# Supplementary material for: Is carotid artery atherosclerosis associated with poor cognitive function assessed using the Mini-Mental State Examination? A systematic review and meta-analysis
Source: BMJ Open. 2022 Apr 18;12(4):e055131. doi: 10.1136/bmjopen-2021-055131 (PMC9020283; doi:10.1136/bmjopen-2021-055131)
Supplement: Supplementary data [file bmjopen-2021-055131supp002.pdf]

Database: Ovid MEDLINE(R) and Epub Ahead of Print, In-Process & Other Non-Indexed Citations and Daily <1946 to February 26, 2020>
Search Strategy:
----
1 cognition/ (94748)
2 cognitive function*.tw. (58742)
3 dementia/ (49736)
4 Alzheimer disease/ (91342)
5 1 or 2 or 3 or 4 (257625)
6 (atheroscolerosis or intima media thickness or plaque).mp. (119020)
7 atherosclerosis/ (35080)
8 6 or 7 (145302)
9 5 and 8 (6774)
10 Carotid Arteries/ (32174)
11 carotid arter*.mp. (102137)
12 10 or 11 (102137)
13 9 and 12 (124)

****

2.

A Cross-Sectional Investigation of Cognition and Ultrasound-Based Vascular Strain Indices.
Meshram NH; Jackson D; Varghese T; Mitchell CC; Wilbrand SM; Dempsey RJ; Hermann BP.
Archives of Clinical Neuropsychology. 35(1):46-55, 2019 Jan 24.
[Journal Article]
UI: 30805597
OBJECTIVE: We examine the relationship between variability in the plaque strain distribution estimated using ultrasound with multiple cognitive domains including executive, language, visuospatial reasoning, and memory function.
METHOD: Asymptomatic (n = 42) and symptomatic (n = 34) patients with significant (>60%) carotid artery stenosis were studied for plaque instability using ultrasound strain imaging and

multiple cognitive domains including executive, language, visuospatial reasoning, and memory function. Correlation and ROC analyses were performed between ultrasound strain indices and cognitive function. Strain indices and cognition scores were also compared between symptomatic and asymptomatic patients to determine whether there are significant group differences.
RESULTS: Association of high-strain distributions with dysexecutive function was observed in both asymptomatic and symptomatic patients. For memory, visuospatial, and language functions, the correlations between strain and cognition were weaker for the asymptomatic compared to symptomatic group.
CONCLUSIONS: Both asymptomatic and symptomatic patients demonstrate a relationship between vessel strain indices and executive function indicating that silent strokes and micro-emboli could initially contribute to a decline in executive function, whereas strokes and transient ischemic attacks may cause the further decline in other cognitive functions.
Copyright © The Author(s) 2019. Published by Oxford University Press. All rights reserved. For permissions, please e-mail: journals.permissions@oup.com.
Version ID
1
Record Owner
From MEDLINE, a database of the U.S. National Library of Medicine.
Status
In-Process
Authors Full Name
Meshram, N H; Jackson, D; Varghese, T; Mitchell, C C; Wilbrand, S M; Dempsey, R J; Hermann, B P.
Institution
Meshram, N H. Department of Medical Physics, University of Wisconsin School of Medicine and Public Health, Madison, WI, USA.   Meshram, N H. Department of Electrical and Computer Engineering, University of Wisconsin-Madison, Madison, WI, USA.
Jackson, D. Department of Neurology, University of Wisconsin School of Medicine and Public Health, Madison, WI, USA.
Varghese, T. Department of Medical Physics, University of Wisconsin School of Medicine and Public Health, Madison, WI, USA.
Varghese, T. Department of Electrical and Computer Engineering, University of Wisconsin-Madison, Madison, WI, USA.
Mitchell, C C. Department of Medicine, University of Wisconsin School of Medicine and Public Health, Madison, WI, USA.
Wilbrand, S M. Department of Neurological Surgery, University of Wisconsin School of Medicine and Public Health, Madison, WI, USA.

Dempsey, R J. Department of Neurological Surgery, University of Wisconsin School of Medicine and Public Health, Madison, WI, USA.
Hermann, B P. Department of Neurology, University of Wisconsin School of Medicine and Public Health, Madison, WI, USA.
Keyword Heading
Carotid stenosis Cerebrovascular disease
Cognitive impairment
Early diagnosis
Executive functioning
Neuropsychological tests.
Year of Publication
2019

Link to the Ovid Full Text or citation:
Click here for full text options

Link to the External Link Resolver:
SFX

4.

Carotid Intima-media Thickness, Cognitive Performance and Cognitive Decline in Stroke-free Middle-aged and Older Adults. The Atahualpa Project.
Del Brutto OH; Mera RM; Recalde BY; Del Brutto VJ.
Journal of Stroke & Cerebrovascular Diseases. 29(2):104576, 2020 Feb.
[Journal Article]
UI: 31839546
BACKGROUND: Little is known on factors influencing cognitive function in rural communities. Using the Atahualpa Project cohort, we aimed to assess whether the carotid intima-media thickness (cIMT) - used as a surrogate of extracranial carotid atherosclerosis - is associated with cognitive performance and further decline in community-dwelling adults living in a rural setting. METHODS: The study included Atahualpa residents aged greater than or equal to 40 years who had ultrasound examination of the extracranial carotid arteries and a baseline Montreal Cognitive

Assessment (MoCA), as well as the subset of individuals who also had a follow-up MoCA at least 1 year after baseline. Relationship between cIMT and cognitive function was assessed by means of generalized linear and longitudinal models, adjusted for relevant covariates. Mediation analysis was utilized to establish the proportion of the effect between increased cIMT and cognitive performance, which is mediated by age.
RESULTS: A total of 561 individuals were included for the cross-sectional study, and 510 of them were assessed for the prospective cohort. Univariate analysis showed a significant association between increased cIMT and worse cognitive performance ($P < .001$), which vanishes after considering the effect of age and low scholarity. Causal mediation analysis confirms that age captures 82.6% (95% C.I.: 63.9% to 100%) of the effect of this association. There was no relationship between increased cIMT and cognitive decline in the follow-up.
CONCLUSIONS: In this rural population, the association between increased cIMT and cognitive dysfunction is mostly mediated by increasing age.
Copyright © 2019 Elsevier Inc. All rights reserved.
Version ID
1
Record Owner
From MEDLINE, a database of the U.S. National Library of Medicine.
Status
MEDLINE
Authors Full Name
Del Brutto, Oscar H; Mera, Robertino M; Recalde, Bettsy Y; Del Brutto, Victor J.
Institution
Del Brutto, Oscar H. School of Medicine, Universidad Espiritu Santo - Ecuador, Samborondon, Ecuador. Electronic address: oscardelbrutto@hotmail.com.   Mera, Robertino M. Department of Epidemiology, Gilead Sciences, Inc., Foster City, California.
Recalde, Bettsy Y. Community Center, the Atahualpa Project, Atahualpa, Ecuador.
Del Brutto, Victor J. Department of Neurology, Miller School of Medicine, University of Miami, Miami, Florida.
MeSH Heading
Adult. Age Factors. Aged. Aged, 80 and over. *Carotid Artery Diseases/dg [Diagnostic Imaging]. Carotid Artery Diseases/ep [Epidemiology]. *Carotid Intima-Media Thickness. *Cognition. *Cognitive Aging/px [Psychology]. Cognitive Dysfunction/di [Diagnosis]. Cognitive Dysfunction/ep [Epidemiology]. *Cognitive Dysfunction/px [Psychology]. Cross-Sectional Studies. Ecuador/ep [Epidemiology]. Female. Humans. Longitudinal Studies. Male. Middle Aged. Predictive Value of Tests. Prognosis. Prospective Studies. Risk Assessment. Risk Factors. *Rural Health. Time Factors.
Keyword Heading

Carotid intima-media thickness aging
cognitive decline
cognitive performance
extracranial carotid atherosclerosis
population study.
Year of Publication
2020

Link to the Ovid Full Text or citation:
[Click here for full text options]

Link to the External Link Resolver:
[SFX]

5.

Association Between Carotid Artery Intima-Media Thickness and Combinations of Mild Cognitive Impairment and Pre-Frailty in Older Adults.
Park J; Park JH; Park H.
International Journal of Environmental Research & Public Health [Electronic Resource]. 16(16), 2019 08 19.
[Journal Article. Research Support, Non-U.S. Gov't]
UI: 31430926
Carotid intima-media thickness (CIMT) has been proposed as a surrogate marker of cardiovascular disease. Mild cognitive impairment (MCI) and pre-frailty are reportedly associated with increased CIMT. As the evidence on the association of CIMT with combinations of MCI and pre-frailty is limited, this association is examined. A total of 231 older adults participated. MCI was defined according to clinical consensus or psychometric criteria by a dementia specialist, and considering detailed neuropsychological assessments. Also, pre-frailty was defined as subjects with frail component of 1 or 2. Carotid variables were measured using a B-mode ultrasound. The analysis of covariance (ANCOVA) was performed to assess independent differences in CIMT among the four groups, according to the cognitive function and frailty status after a multivariate adjustment. Increased CIMT is associated with combinations of MCI and pre-frailty. ANCOVA showed that

CIMTs were significantly different among the four groups according to the cognitive function and frailty status. CIMTmax combined with MCI and pre-frailty was the thickest (1.04 +/- 0.3 mm), whereas the CIMT of no MCI and no pre-frailty was the thinnest (0.82 +/- 0.2 mm). The results suggest that combinations of MCI and pre-frailty are associated with increased CIMT in older adults.

Version ID

1

Record Owner

From MEDLINE, a database of the U.S. National Library of Medicine.

Status

MEDLINE

Author NameID

Park, Jong-Hwan; ORCID: https://orcid.org/0000-0003-2815-7248 Park, Hyuntae; ORCID: https://orcid.org/0000-0002-1976-0005

Authors Full Name

Park, Jinkee; Park, Jong-Hwan; Park, Hyuntae.

Institution

Park, Jinkee. Department of Sport Rehabilitation, Dong-Ju College, Busan 49318, Korea. Park, Jong-Hwan. Health Convergence Medicine Research Group, Biomedical Research Institute, Pusan National University Hospital, 179, Gudeok-Ro, Seo-Gu, Busan 49241, Korea.

Park, Hyuntae. Department of Health Care Science, Dong-A University, Busan 49315, Korea. htpark@dau.ac.kr.

Park, Hyuntae. Institute of Convergence Bio-Health, Dong-A University, Busan 49201, Korea. htpark@dau.ac.kr.

MeSH Heading

Aged. Aged, 80 and over. *Carotid Intima-Media Thickness. *Cognitive Dysfunction/co [Complications]. Cognitive Dysfunction/di [Diagnosis]. Cognitive Dysfunction/pp [Physiopathology]. Cross-Sectional Studies. Female. *Frailty/co [Complications]. Frailty/pp [Physiopathology]. Frailty/px [Psychology]. Humans. Male. Neuropsychological Tests.

Keyword Heading

*cardiovascular disease *carotid intima-media thickness

*mild cognitive impairment

*older adults

*pre-frailty.

Year of Publication

2019

Link to the Ovid Full Text or citation:
[Click here for full text options]

Link to the External Link Resolver:
[SFX]

6.

Subclinical carotid atherosclerosis and neurocognitive function in an urban population.
Wendell CR; Waldstein SR; Evans MK; Zonderman AB.
Atherosclerosis. 249:125-31, 2016 06.
[Journal Article. Research Support, N.I.H., Extramural. Research Support, N.I.H., Intramural]
UI: 27092741

BACKGROUND AND AIMS: Examine age, sex, race, and socioeconomic status as modifiers of the association between carotid intimal medial thickness (IMT) and neurocognitive performance in a socioeconomically diverse, biracial, urban, adult population.
METHODS: Participants were 1712 community-dwelling adults (45% men, 56% African-American, 38% below poverty threshold, aged 30-64 years) enrolled in the Healthy Aging in Neighborhoods of Diversity across the Life Span (HANDLS) study. Participants underwent initial carotid ultrasonography followed by cognitive testing on up to two occasions over 4 years. Mixed-effects regression analyses were adjusted for demographic, behavioral, and biomedical covariates.
RESULTS: Significant cross-sectional IMT x race x poverty interactions were identified for measures of delayed recall memory, auditory-verbal attention, and working memory. An IMT x race interaction also appeared for auditory-verbal learning. Higher IMT was generally associated with worse cognitive performance, but the disadvantage was most pronounced among those with higher socioeconomic status and white participants. No longitudinal associations were identified.
CONCLUSIONS: Carotid IMT-cognition associations differed as a function of race and socioeconomic status and were most compelling for measures of attention, executive function, and memory. These findings highlight the possibility that subclinical atherosclerosis may be differentially informative as a predictor of cognitive performance among varied demographic subgroups.
Copyright © 2016 Elsevier Ireland Ltd. All rights reserved.
Version ID

1

Record Owner

From MEDLINE, a database of the U.S. National Library of Medicine.

Status

MEDLINE

Authors Full Name

Wendell, Carrington R; Waldstein, Shari R; Evans, Michele K; Zonderman, Alan B.

Institution

Wendell, Carrington R. Department of Psychology, University of Maryland, Baltimore County, USA; Laboratory of Epidemiology and Population Sciences, Intramural Research Program, National Institute on Aging, NIH, USA. Electronic address: crwendell@umbc.edu. Waldstein, Shari R. Department of Psychology, University of Maryland, Baltimore County, USA; Division of Gerontology & Geriatric Medicine, Department of Medicine, University of Maryland School of Medicine, USA; Geriatric Research Education and Clinical Center, Baltimore VA Medical Center, USA.

Evans, Michele K. Laboratory of Epidemiology and Population Sciences, Intramural Research Program, National Institute on Aging, NIH, USA.

Zonderman, Alan B. Laboratory of Epidemiology and Population Sciences, Intramural Research Program, National Institute on Aging, NIH, USA.

MeSH Heading

Adult. African Americans. Atherosclerosis/ep [Epidemiology]. Attention. Baltimore. Carotid Artery Diseases/co [Complications]. *Carotid Artery Diseases/di [Diagnosis]. Carotid Artery Diseases/eh [Ethnology]. Carotid Intima-Media Thickness. Cognition. Cognition Disorders/co [Complications]. *Cognition Disorders/di [Diagnosis]. Cognition Disorders/eh [Ethnology]. European Continental Ancestry Group. Female. Humans. Longitudinal Studies. Male. Memory. Middle Aged. Poverty. Prospective Studies. Regression Analysis. Social Class. *Urban Population.

Keyword Heading

*Carotid intimal medial thickness *Cognitive function

*Subclinical cardiovascular disease.

Year of Publication

2016

Link to the Ovid Full Text or citation:

Click here for full text options

Link to the External Link Resolver:

SFX

8.

Carotid artery plaque detected on ultrasound is associated with impaired cognitive state in the elderly: A population-based study in Wakiso district, Uganda.
Mworozi K; Ameda F; Byanyima RK; Nakasujja N.
Journal of Clinical Neuroscience. 68:194-200, 2019 Oct.
[Journal Article]
UI: 31301929
Carotid artery disease which includes carotid artery stenosis, plaques, clots and increased intima media thickness, have been reported by many studies to be associated with dementia. Dementia is an end stage of usually asymptomatic cognitive impairment. Risk factors of carotid artery disease include; age, atherosclerosis, arteriosclerosis, shorter years in school, history of hypertension, diabetes mellitus, stroke and depression. This study set out to determine the prevalence of abnormal carotid ultrasound findings and their association with cognitive function among the adults >=60years in Wakiso district, Uganda in 2018. A total of 210 participants were included. Carotid artery stenosis, presence of plaque, stenosis and intima-media thickness were assessed by ultrasound. Cognitive status was assessed using a Mini Mental State Exam (MMSE) test. The prevalence of plaque was 21.4%. Variables which included; presence of plaque, age, education, gender, marital status, whether participant stayed alone or with someone else, care for self, occupation status, division of staying and history of smoking. The presence of plaque was associated with an abnormal cognitive function at both univariate and multivariate analysis with respective OR=3.8 (95% CI=1.90-7.54, p-value=0.0001) and OR=3.4 (95% CI=1.38-8.15, p-value=0.007). The cognitive function distribution was 43.8%, 19%, 34.3% and 2.9% within the normal, mild, moderate, and severe cognitive function status respectively. This study showed that prevalence of carotid artery plaque was high in this elderly population in Wakiso district Uganda. Also, carotid artery plaque was associated with abnormal cognitive function.
Copyright © 2019 Elsevier Ltd. All rights reserved.
Version ID
1
Record Owner
From MEDLINE, a database of the U.S. National Library of Medicine.
Status

MEDLINE

Authors Full Name

Mworozi, Kenneth; Ameda, Faith; Byanyima, Rosemary K; Nakasujja, Noeline.

Institution

Mworozi, Kenneth. Department of Radiology, School of Medicine, P.O. Box 7062, Kampala, Uganda. Electronic address: mworozikenneth@gmail.com. Ameda, Faith. Department of Radiology, Makerere University College of Health Sciences, P.O. Box 7062, Kampala, Uganda.
Byanyima, Rosemary K. Department of Radiology, Mulago National Referral Hospital, P.O. Box 7072, Kampala, Uganda.
Nakasujja, Noeline. Department of Psychiatry, Makerere University College of Health Sciences, P.O. Box 7062, Kampala, Uganda.

MeSH Heading

Aged. Carotid Intima-Media Thickness/px [Psychology]. *Carotid Stenosis/co [Complications]. Carotid Stenosis/dg [Diagnostic Imaging]. *Carotid Stenosis/ep [Epidemiology]. *Cognitive Dysfunction/et [Etiology]. Female. Humans. Male. Middle Aged. Multivariate Analysis. *Plaque, Atherosclerotic/co [Complications]. Plaque, Atherosclerotic/dg [Diagnostic Imaging]. *Plaque, Atherosclerotic/ep [Epidemiology]. Prevalence. Risk Factors. Uganda/ep [Epidemiology]. Ultrasonography.

Keyword Heading

Abnormal cognitive function Carotid artery disease

Carotid artery plaque

Carotid ultrasound

Elderly

Intima-media thickness

Mini-mental state examination.

Year of Publication

2019

Link to the Ovid Full Text or citation:

Click here for full text options

Link to the External Link Resolver:

SFX

10.

Common Carotid Artery Calcification Impacts on Cognitive Function in Older Patients.
Di Daniele N; Celotto R; Alunni Fegatelli D; Gabriele M; Rovella V; Scuteri A.
High Blood Pressure & Cardiovascular Prevention. 26(2):127-134, 2019 Apr.
[Journal Article]
UI: 30779026
INTRODUCTION: Cognitive impairment and dementia represent an emerging health problem. Cardiovascular (CV) risk factors contribute to cognitive impairment.
AIM: To investigate the effect of vascular calcification on cognitive impairment and dementia, independently of plaque and traditional CV risk factors.
METHODS: Four hundred and sixty-nine patients (age of 78.6 +/- 6.1 years, 74.4% women) were studied. Traditional CV risk factors levels, cognitive function (MMSE), brain CT scan, and other vascular parameters were measured. Common Carotid Artery (CCA) plaque and calcification were evaluated by ultrasound.
RESULTS: CCA calcification was associated with a lower MMSE score than in subjects with no CCA calcification (23.7 +/- 0.3 versus 25.5 +/- 0.8; p = 0.015), after controlling for age, sex, education, blood pressure levels, diabetes, creatinine, lipid lowering therapy, neuroimaging alteration, and CCA plaque. Similarly, CCA calcification was associated with higher odds of dementia regardless of the presence of CCA plaque (OR 1.70, 95% CI 1.01-2.94, p < 0.05). This trend was not observed when stratifying patients according to the presence of CCA plaque.
CONCLUSION: CCA calcification is associated with cognitive impairment and dementia, independently of established CV risk factors and CCA plaque. The impact of arterial calcification on cognition seems largely independent of arterial stiffness.
Version ID
1
Record Owner
From MEDLINE, a database of the U.S. National Library of Medicine.
Status
MEDLINE
Authors Full Name
Di Daniele, Nicola; Celotto, Roberto; Alunni Fegatelli, Danilo; Gabriele, Marco; Rovella, Valentina; Scuteri, Angelo.
Institution
Di Daniele, Nicola. Hypertension and Nephrology Unit, Department of Medicine, Policinico Tor Vergata, Universita'di Roma Tor Vergata, Rome, Italy.   Celotto, Roberto. Hypertension and

Nephrology Unit, Department of Medicine, Policinico Tor Vergata, Universita'di Roma Tor Vergata, Rome, Italy.
Alunni Fegatelli, Danilo. Department of Public Health and Infectious Disease, University "La Sapienza", Rome, Italy.
Gabriele, Marco. Hypertension and Nephrology Unit, Department of Medicine, Policinico Tor Vergata, Universita'di Roma Tor Vergata, Rome, Italy.
Rovella, Valentina. Hypertension and Nephrology Unit, Department of Medicine, Policinico Tor Vergata, Universita'di Roma Tor Vergata, Rome, Italy.
Scuteri, Angelo. Department of Medical, Surgical, and Experimental Sciences, University of Sassari, Sassari, Italy. d341elefante@virgilio.it.
MeSH Heading
Age Factors. Aged. Aged, 80 and over. *Carotid Artery Diseases/co [Complications]. Carotid Artery Diseases/dg [Diagnostic Imaging]. Carotid Artery Diseases/pp [Physiopathology]. Carotid Artery, Common/dg [Diagnostic Imaging]. Carotid Artery, Common/pp [Physiopathology]. *Carotid Artery, Common. *Cognition. Cognition Disorders/di [Diagnosis]. *Cognition Disorders/et [Etiology]. Cognition Disorders/px [Psychology]. *Cognitive Aging. Dementia/di [Diagnosis]. *Dementia/et [Etiology]. Dementia/px [Psychology]. Female. Humans. Male. Mental Status and Dementia Tests. Plaque, Atherosclerotic. Pulse Wave Analysis. Risk Factors. Tomography, X-Ray Computed. Ultrasonography. *Vascular Calcification/co [Complications]. Vascular Calcification/dg [Diagnostic Imaging]. Vascular Calcification/pp [Physiopathology]. Vascular Stiffness.
Keyword Heading
Arterial stiffness Cognitive impairment
Dementia
Vascular aging
Vascular calcification.
Year of Publication
2019

Link to the Ovid Full Text or citation:
Click here for full text options

Link to the External Link Resolver:
SFX

11.

Cortical Cerebral Microinfarcts on 3T Magnetic Resonance Imaging in Patients With Carotid Artery Stenosis.
Takasugi J; Miwa K; Watanabe Y; Okazaki S; Todo K; Sasaki T; Sakaguchi M; Mochizuki H.
Stroke. 50(3):639-644, 2019 03.
[Journal Article. Research Support, Non-U.S. Gov't]
UI: 30744544
Background and Purpose - Carotid artery stenosis is common in the elderly and contributes to cognitive impairment and dementia. Cortical cerebral microinfarcts (CMIs) play an important role in vascular cognitive impairment and dementia. We aimed to investigate the association between CMIs on 3T magnetic resonance imaging and clinical and radiological features, including plaque morphology, and cognitive function in patients with carotid stenosis. Methods- Eighty-nine patients with >30% carotid stenosis on ultrasound were prospectively enrolled, and underwent brain and carotid artery magnetic resonance imaging. CMIs were rated according to predetermined criteria based on 3D-double inversion recovery and fluid-attenuated inversion recovery images. Results - CMIs were identified in 26 patients (29%; median number 0, range 0-9). Poisson regression models adjusted for age and sex revealed that CMIs were associated with intraplaque hemorrhage (rate ratio, 1.95; 95% CI, 1.26-3.18), lacunar infarcts (rate ratio, 1.54; 95% CI, 1.00-2.44), and cortical infarcts (rate ratio, 3.22; 95% CI, 2.20-5.00). These associations were also observed in asymptomatic patients (n=64). Of 81 patients with unilateral carotid stenosis, the prevalence and number of CMIs were significantly higher in the hemisphere ipsilateral to the carotid stenosis than in the contralateral hemisphere (P=0.005 and P<0.001, respectively). The presence of CMIs was associated with poor cognitive function. Conclusions - Our results indicate that vulnerable carotid plaque increases the risk of CMIs and subsequent cognitive impairment. Carotid atherosclerosis could be a potential therapeutic target for cognitive impairment.
Version ID
1
Record Owner
From MEDLINE, a database of the U.S. National Library of Medicine.
Status
MEDLINE
Authors Full Name
Takasugi, Junji; Miwa, Kaori; Watanabe, Yoshiyuki; Okazaki, Shuhei; Todo, Kenichi; Sasaki, Tsutomu; Sakaguchi, Manabu; Mochizuki, Hideki.
Institution

Takasugi, Junji. From the Department of Neurology (J.T., K.M., S.O., K.T., T.S., M.S., H.M.), Osaka University Graduate School of Medicine, Japan. Miwa, Kaori. From the Department of Neurology (J.T., K.M., S.O., K.T., T.S., M.S., H.M.), Osaka University Graduate School of Medicine, Japan.
Miwa, Kaori. Department of Cerebrovascular Medicine, National Cerebral and Cardiovascular Center, Osaka, Japan (K.W.).
Watanabe, Yoshiyuki. Department of Diagnostic and Interventional Radiology (Y.W.), Osaka University Graduate School of Medicine, Japan.
Okazaki, Shuhei. From the Department of Neurology (J.T., K.M., S.O., K.T., T.S., M.S., H.M.), Osaka University Graduate School of Medicine, Japan.
Todo, Kenichi. From the Department of Neurology (J.T., K.M., S.O., K.T., T.S., M.S., H.M.), Osaka University Graduate School of Medicine, Japan.
Sasaki, Tsutomu. From the Department of Neurology (J.T., K.M., S.O., K.T., T.S., M.S., H.M.), Osaka University Graduate School of Medicine, Japan.
Sakaguchi, Manabu. From the Department of Neurology (J.T., K.M., S.O., K.T., T.S., M.S., H.M.), Osaka University Graduate School of Medicine, Japan.
Mochizuki, Hideki. From the Department of Neurology (J.T., K.M., S.O., K.T., T.S., M.S., H.M.), Osaka University Graduate School of Medicine, Japan.
MeSH Heading
Aged. Aged, 80 and over. *Carotid Stenosis/dg [Diagnostic Imaging]. Carotid Stenosis/ep [Epidemiology]. Carotid Stenosis/px [Psychology]. *Cerebral Cortex/dg [Diagnostic Imaging]. *Cerebral Infarction/dg [Diagnostic Imaging]. Cerebral Infarction/ep [Epidemiology]. Cerebral Infarction/px [Psychology]. Cognition. Female. Humans. Image Processing, Computer-Assisted. Intracranial Arteriosclerosis/dg [Diagnostic Imaging]. Intracranial Arteriosclerosis/ep [Epidemiology]. Magnetic Resonance Imaging. Male. Mental Status and Dementia Tests. Neuropsychological Tests. Prevalence. Prospective Studies. Ultrasonography.
Keyword Heading
*carotid stenosis *cognition
*dementia
*embolism
*magnetic resonance imaging.
Year of Publication
2019

Link to the Ovid Full Text or citation:
Click here for full text options

Link to the External Link Resolver:
SFX

12.

Healthy Lifestyle During the Midlife Is Prospectively Associated With Less Subclinical Carotid Atherosclerosis: The Study of Women's Health Across the Nation.
Wang D; Jackson EA; Karvonen-Gutierrez CA; Elliott MR; Harlow SD; Hood MM; Derby CA; Sternfeld B; Janssen I; Crawford SL; Huang MH; El Khoudary SR; Chae CU; Baylin A.
Journal of the American Heart Association. 7(23):e010405, 2018 12 04.
[Journal Article. Research Support, N.I.H., Extramural]
UI: 30482079
Background Measures of subclinical atherosclerosis are predictors of future cardiovascular outcomes as well as of physical and cognitive functioning. The menopausal transition is associated with accelerated progression of atherosclerosis in women. The prospective association between a healthy lifestyle during the midlife and subclinical atherosclerosis is unclear. Methods and Results Self-reported data on smoking, diet, and physical activity from 1143 women in the Study of Women's Health Across the Nation were used to construct a 10-year average Healthy Lifestyle Score (HLS) during the midlife. Markers of subclinical atherosclerosis were measured 14 years after baseline and included common carotid artery intima-media thickness (CCA - IMT), adventitial diameter (CCA - AD), and carotid plaque. The associations of average HLS with CCA - IMT and CCA - AD were estimated using linear models; the association of average HLS with carotid plaque was estimated using cumulative logit models. Average HLS was associated with smaller CCA - IMT and CCA - AD in the fully adjusted models (P=0.0031 and <0.001, respectively). Compared with participants in the lowest HLS level, those in the highest level had 0.024 mm smaller CCA - IMT (95% confidence interval: -0.048, 0.000), which equals 17% of the SD of CCA - IMT , and 0.16 mm smaller CCA - AD (95% confidence interval: -0.27, -0.04), which equals 24% of the SD of CCA - AD . Among the 3 components of the HLS , abstinence from smoking had the strongest association with subclinical atherosclerosis. Conclusions Healthy lifestyle during the menopausal transition is associated with less subclinical atherosclerosis, highlighting the growing recognition that the midlife is a critical window for cardiovascular prevention in women.
Version ID
1
Record Owner

From MEDLINE, a database of the U.S. National Library of Medicine.

Status

MEDLINE

Authors Full Name

Wang, Dongqing; Jackson, Elizabeth A; Karvonen-Gutierrez, Carrie A; Elliott, Michael R; Harlow, Sioban D; Hood, Michelle M; Derby, Carol A; Sternfeld, Barbara; Janssen, Imke; Crawford, Sybil L; Huang, Mei-Hua; El Khoudary, Samar R; Chae, Claudia U; Baylin, Ana.

Institution

Wang, Dongqing. 1 Department of Epidemiology University of Michigan Ann Arbor MI. Jackson, Elizabeth A. 2 Division of Cardiovascular Disease University of Alabama at Birmingham AL.

Karvonen-Gutierrez, Carrie A. 1 Department of Epidemiology University of Michigan Ann Arbor MI.

Elliott, Michael R. 3 Department of Biostatistics University of Michigan Ann Arbor MI.

Elliott, Michael R. 4 Survey Research Center University of Michigan Ann Arbor MI.

Harlow, Sioban D. 1 Department of Epidemiology University of Michigan Ann Arbor MI.

Hood, Michelle M. 1 Department of Epidemiology University of Michigan Ann Arbor MI.

Derby, Carol A. 5 Department of Neurology Albert Einstein College of Medicine Bronx NY.

Derby, Carol A. 6 Department of Epidemiology and Population Health Albert Einstein College of Medicine Bronx NY.

Sternfeld, Barbara. 7 Division of Research Kaiser Permanente Oakland CA.

Janssen, Imke. 8 Department of Preventive Medicine Rush University Medical Center Chicago IL.

Crawford, Sybil L. 9 Medical School University of Massachusetts Worcester MA.

Huang, Mei-Hua. 10 Division of Geriatrics University of California Los Angeles Los Angeles CA.

El Khoudary, Samar R. 11 Department of Epidemiology University of Pittsburgh PA.

Chae, Claudia U. 12 Cardiology Division Massachusetts General Hospital Boston MA.

Baylin, Ana. 1 Department of Epidemiology University of Michigan Ann Arbor MI.

Baylin, Ana. 13 Department of Nutritional Sciences University of Michigan Ann Arbor MI.

Comments

Erratum in (EIN)

MeSH Heading

Asymptomatic Diseases. Carotid Artery Diseases/ep [Epidemiology]. Carotid Artery Diseases/et [Etiology]. *Carotid Artery Diseases/pc [Prevention & Control]. Diet/ae [Adverse Effects]. Exercise. Female. Healthy Lifestyle/ph [Physiology]. *Healthy Lifestyle. Humans. Middle Aged. Prospective Studies. Risk Factors. Smoking/ae [Adverse Effects]. United States/ep [Epidemiology].

Keyword Heading

*atherosclerosis *cardiovascular disease

*lifestyle

*risk factors

*women.
Year of Publication
2018

Link to the Ovid Full Text or citation:
Click here for full text options

Link to the External Link Resolver:
SFX

13.

Pulse Wave Velocity Is Associated With Greater Risk of Dementia in Mild Cognitive Impairment Patients.
Rouch L; Cestac P; Sallerin B; Andrieu S; Bailly H; Beunardeau M; Cohen A; Dubail D; Hernandorena I; Seux ML; Vidal JS; Hanon O.
Hypertension. 72(5):1109-1116, 2018 11.
[Journal Article]
UI: 30354804
To investigate the association between pulse wave velocity, intima-media thickness, carotid artery diameter, carotid plaques, and conversion from mild cognitive impairment to dementia. Three hundred and seventy-five elderly ambulatory subjects with mild cognitive impairment were followed yearly to examine potential conversion to dementia. Vascular function was assessed by carotid-femoral pulse wave velocity. Vascular structure was evaluated by intima-media thickness, carotid artery diameter, and carotid plaques using an ultrasonographic assessment of carotid arteries. One hundred and five patients (28%) converted to dementia during a mean follow-up period of 4.5 years. Higher pulse wave velocity was associated with greater risk of conversion to dementia (1-SD increase of pulse wave velocity: hazard ratio, 1.33; 95% CI, 1.04-1.71; P=0.02) independently of age, sex, educational level, systolic blood pressure, cardiovascular diseases, body mass index, calcium channel blockers intake, Mini-Mental State Examination at baseline, and apoE epsilon4 status. Intima-media thickness, carotid plaques, and carotid artery diameter did not predict conversion to dementia (1-SD increase of intima-media thickness: hazard ratio, 0.93; 95% CI, 0.73-1.18; P=0.55; presence of carotid plaques: hazard ratio, 1.08; 95% CI, 0.62-1.87; P=0.79; 1-

SD increase of carotid artery diameter: hazard ratio, 1.08; 95% CI, 0.89-1.31; P=0.44). Pulse wave velocity was associated with conversion to dementia, whereas intima-media thickness, carotid plaques, or carotid artery diameter were not after controlling for age and other confounding factors. Arterial stiffness could identify mild cognitive impairment patients at higher risk of dementia and may be a therapeutic target to delay or prevent the onset of dementia.

Version ID

1

Record Owner

From MEDLINE, a database of the U.S. National Library of Medicine.

Status

MEDLINE

Authors Full Name

Rouch, Laure; Cestac, Philippe; Sallerin, Brigitte; Andrieu, Sandrine; Bailly, Henri; Beunardeau, Maelle; Cohen, Adrien; Dubail, Delphine; Hernandorena, Intza; Seux, Marie-Laure; Vidal, Jean-Sebastien; Hanon, Olivier.

Institution

Rouch, Laure. From the EA 4468, Universite Paris Descartes, Sorbonne Paris Cite, France (L.R., H.B., M.B., A.C., D.D., I.H., M.-L.S., J.-S.V., O.H.). Cestac, Philippe. Unite INSERM 1027, Toulouse, France (P.C., S.A.).

Cestac, Philippe. University Paul Sabatier Toulouse III, France (P.C., B.S., S.A.).

Cestac, Philippe. Pole Pharmacie, Centre Hospitalier Universitaire de Toulouse, France (P.C., B.S.).

Sallerin, Brigitte. University Paul Sabatier Toulouse III, France (P.C., B.S., S.A.).

Sallerin, Brigitte. Pole Pharmacie, Centre Hospitalier Universitaire de Toulouse, France (P.C., B.S.).

Sallerin, Brigitte. Unite INSERM 1048, Toulouse, France (B.S.).

Andrieu, Sandrine. Unite INSERM 1027, Toulouse, France (P.C., S.A.).

Andrieu, Sandrine. University Paul Sabatier Toulouse III, France (P.C., B.S., S.A.).

Andrieu, Sandrine. Unite INSERM 1048, Toulouse, France (B.S.).

Andrieu, Sandrine. Pole Epidemiologie et Sante Publique, Centre Hospitalier Universitaire de Toulouse, France (S.A.).

Bailly, Henri. From the EA 4468, Universite Paris Descartes, Sorbonne Paris Cite, France (L.R., H.B., M.B., A.C., D.D., I.H., M.-L.S., J.-S.V., O.H.).

Bailly, Henri. Service de geriatrie, Hopital Broca, AP-HP, Hopitaux Universitaires Paris Centre, France (H.B., M.B., A.C., D.D., I.H., M.-L.S., J.-S.V., O.H.).

Beunardeau, Maelle. From the EA 4468, Universite Paris Descartes, Sorbonne Paris Cite, France (L.R., H.B., M.B., A.C., D.D., I.H., M.-L.S., J.-S.V., O.H.).

Beunardeau, Maelle. Service de geriatrie, Hopital Broca, AP-HP, Hopitaux Universitaires Paris Centre, France (H.B., M.B., A.C., D.D., I.H., M.-L.S., J.-S.V., O.H.).

Cohen, Adrien. From the EA 4468, Universite Paris Descartes, Sorbonne Paris Cite, France (L.R., H.B., M.B., A.C., D.D., I.H., M.-L.S., J.-S.V., O.H.).
Cohen, Adrien. Service de geriatrie, Hopital Broca, AP-HP, Hopitaux Universitaires Paris Centre, France (H.B., M.B., A.C., D.D., I.H., M.-L.S., J.-S.V., O.H.).
Dubail, Delphine. From the EA 4468, Universite Paris Descartes, Sorbonne Paris Cite, France (L.R., H.B., M.B., A.C., D.D., I.H., M.-L.S., J.-S.V., O.H.).
Dubail, Delphine. Service de geriatrie, Hopital Broca, AP-HP, Hopitaux Universitaires Paris Centre, France (H.B., M.B., A.C., D.D., I.H., M.-L.S., J.-S.V., O.H.).
Hernandorena, Intza. From the EA 4468, Universite Paris Descartes, Sorbonne Paris Cite, France (L.R., H.B., M.B., A.C., D.D., I.H., M.-L.S., J.-S.V., O.H.).
Hernandorena, Intza. Service de geriatrie, Hopital Broca, AP-HP, Hopitaux Universitaires Paris Centre, France (H.B., M.B., A.C., D.D., I.H., M.-L.S., J.-S.V., O.H.).
Seux, Marie-Laure. From the EA 4468, Universite Paris Descartes, Sorbonne Paris Cite, France (L.R., H.B., M.B., A.C., D.D., I.H., M.-L.S., J.-S.V., O.H.).
Seux, Marie-Laure. Service de geriatrie, Hopital Broca, AP-HP, Hopitaux Universitaires Paris Centre, France (H.B., M.B., A.C., D.D., I.H., M.-L.S., J.-S.V., O.H.).
Vidal, Jean-Sebastien. From the EA 4468, Universite Paris Descartes, Sorbonne Paris Cite, France (L.R., H.B., M.B., A.C., D.D., I.H., M.-L.S., J.-S.V., O.H.).
Vidal, Jean-Sebastien. Service de geriatrie, Hopital Broca, AP-HP, Hopitaux Universitaires Paris Centre, France (H.B., M.B., A.C., D.D., I.H., M.-L.S., J.-S.V., O.H.).
Hanon, Olivier. From the EA 4468, Universite Paris Descartes, Sorbonne Paris Cite, France (L.R., H.B., M.B., A.C., D.D., I.H., M.-L.S., J.-S.V., O.H.).
Hanon, Olivier. Service de geriatrie, Hopital Broca, AP-HP, Hopitaux Universitaires Paris Centre, France (H.B., M.B., A.C., D.D., I.H., M.-L.S., J.-S.V., O.H.).
Comments
Comment in (CIN)
MeSH Heading
Aged. Aged, 80 and over. *Blood Flow Velocity/ph [Physiology]. *Carotid Arteries/pp [Physiopathology]. Carotid Intima-Media Thickness. Cognitive Dysfunction/co [Complications]. *Cognitive Dysfunction/pp [Physiopathology]. Dementia/co [Complications]. *Dementia/pp [Physiopathology]. Female. Humans. Male. Middle Aged. Pulse Wave Analysis. Ultrasonography.
Keyword Heading
*blood pressure *dementia
*patients
*pulse wave analysis
*risk
*vascular stiffness.

Year of Publication
2018

Link to the Ovid Full Text or citation:
Click here for full text options

Link to the External Link Resolver:
SFX

15.

Association between carotid atheroma and cerebral cortex structure at age 73 years.
Alhusaini S; Karama S; Nguyen TV; Thiel A; Bernhardt BC; Cox SR; Corley J; Taylor A; Evans AC; Star JM; Bastin ME; Wardlaw JM; Deary IJ; Ducharme S.
Annals of Neurology. 84(4):576-587, 2018 10.
[Journal Article. Research Support, Non-U.S. Gov't]
UI: 30179274
OBJECTIVE: To examine the relationship between carotid atherosclerosis and cerebral cortical thickness and investigate whether cortical thickness mediates the association between carotid atheroma and relative cognitive decline.
METHODS: We assessed 554 community-dwelling subjects (male/female: 296/258) from the Lothian Birth Cohort 1936 who underwent brain magnetic resonance imaging and carotid Doppler ultrasound studies at age 73 years. The relationship between carotid atherosclerosis markers (internal carotid artery stenosis, intima-media thickness, velocity, pulsatility, and resistivity indexes) and vertex-wide cerebral cortical thickness was examined cross-sectionally, controlling for gender, extensive vascular risk factors (VRFs), and intelligence quotient at age 11 (IQ-11). We also determined the association between carotid stenosis and a composite measure of fluid intelligence at age 73 years. A mediation model was applied to examine whether cortical thickness mediated the relationship between carotid stenosis and cognitive function.
RESULTS: A widespread negative association was identified between carotid stenosis (median = 15%) and cerebral cortical thickness at age 73 years, independent of the side of carotid stenosis, other carotid measures, VRFs, and IQ-11. This association increased in an almost dose-response relationship from mild to severe degrees of carotid stenosis, across the anterior and posterior

circulation territories. A negative association was also noted between carotid stenosis and fluid intelligence (standardized beta coefficient = -0.151, p = 0.001), which appeared partly (approximately 22%) mediated by carotid stenosis-related thinning of the cerebral cortex. INTERPRETATION: The findings suggest that carotid stenosis represents a marker of processes that accelerate aging of the cerebral cortex and cognition that is in part independent of measurable VRFs. Cortical thinning within the anterior and posterior circulation territories partially mediated the relationship between carotid atheroma and fluid intelligence. Ann Neurol 2018;84:576-587. Copyright © 2018 The Authors. Annals of Neurology published by Wiley Periodicals, Inc. on behalf of American Neurological Association.

Version ID

1

Record Owner

From MEDLINE, a database of the U.S. National Library of Medicine.

Status

MEDLINE

Author NameID

Wardlaw, Joanna M; ORCID: https://orcid.org/0000-0002-9812-6642

Authors Full Name

Alhusaini, Saud; Karama, Sherif; Nguyen, Tuong-Vi; Thiel, Alexander; Bernhardt, Boris C; Cox, Simon R; Corley, Janie; Taylor, Adele; Evans, Alan C; Star, John M; Bastin, Mark E; Wardlaw, Joanna M; Deary, Ian J; Ducharme, Simon.

Institution

Alhusaini, Saud. Department of Neurology and Neurosurgery, Montreal Neurological Institute and Hospital, McGill University, Montreal, Quebec, Canada.   Karama, Sherif. McConnell Brain Imaging Centre, Montreal Neurological Institute, McGill University, Montreal, Quebec, Canada.

Karama, Sherif. Department of Psychiatry, Douglas Mental Health University Institute, McGill University, Montreal, Quebec, Canada.

Nguyen, Tuong-Vi. Department of Psychiatry, McGill University Health Centre, McGill University, Montreal, Quebec, Canada.

Nguyen, Tuong-Vi. Department of Obstetrics-Gynecology, McGill University Health Centre, McGill University, Montreal, Quebec, Canada.

Thiel, Alexander. Department of Neurology and Neurosurgery, Montreal Neurological Institute and Hospital, McGill University, Montreal, Quebec, Canada.

Thiel, Alexander. Department of Neurology, Jewish General Hospital, Lady Davis Institute for Medical Research, Montreal, Quebec, Canada.

Bernhardt, Boris C. McConnell Brain Imaging Centre, Montreal Neurological Institute, McGill University, Montreal, Quebec, Canada.

Cox, Simon R. Centre for Cognitive Ageing and Cognitive Epidemiology, Department of Psychology, University of Edinburgh, Edinburgh, United Kingdom.
Corley, Janie. Centre for Cognitive Ageing and Cognitive Epidemiology, Department of Psychology, University of Edinburgh, Edinburgh, United Kingdom.
Taylor, Adele. Centre for Cognitive Ageing and Cognitive Epidemiology, Department of Psychology, University of Edinburgh, Edinburgh, United Kingdom.
Evans, Alan C. McConnell Brain Imaging Centre, Montreal Neurological Institute, McGill University, Montreal, Quebec, Canada.
Star, John M. Centre for Cognitive Ageing and Cognitive Epidemiology, Department of Psychology, University of Edinburgh, Edinburgh, United Kingdom.
Star, John M. Alzheimer Scotland Dementia Research Centre, Department of Psychology, University of Edinburgh, Edinburgh, United Kingdom.
Bastin, Mark E. Centre for Cognitive Ageing and Cognitive Epidemiology, Department of Psychology, University of Edinburgh, Edinburgh, United Kingdom.
Bastin, Mark E. Brain Research Imaging Centre, Centre for Clinical Brain Sciences, University of Edinburgh, Edinburgh, United Kingdom.
Wardlaw, Joanna M. Centre for Cognitive Ageing and Cognitive Epidemiology, Department of Psychology, University of Edinburgh, Edinburgh, United Kingdom.
Wardlaw, Joanna M. Brain Research Imaging Centre, Centre for Clinical Brain Sciences, University of Edinburgh, Edinburgh, United Kingdom.
Wardlaw, Joanna M. UK Dementia Research Institute at the University of Edinburgh, Edinburgh, United Kingdom.
Deary, Ian J. Centre for Cognitive Ageing and Cognitive Epidemiology, Department of Psychology, University of Edinburgh, Edinburgh, United Kingdom.
Deary, Ian J. Alzheimer Scotland Dementia Research Centre, Department of Psychology, University of Edinburgh, Edinburgh, United Kingdom.
Ducharme, Simon. Department of Neurology and Neurosurgery, Montreal Neurological Institute and Hospital, McGill University, Montreal, Quebec, Canada.
Ducharme, Simon. McConnell Brain Imaging Centre, Montreal Neurological Institute, McGill University, Montreal, Quebec, Canada.
Ducharme, Simon. Department of Psychiatry, McGill University Health Centre, McGill University, Montreal, Quebec, Canada.
MeSH Heading
Aged. *Carotid Artery Diseases/dg [Diagnostic Imaging]. *Carotid Artery Diseases/px [Psychology]. *Carotid Artery, Internal/dg [Diagnostic Imaging]. *Cerebral Cortex/dg [Diagnostic Imaging]. Cohort Studies. Cross-Sectional Studies. Female. Humans. Magnetic Resonance Imaging/td

[Trends]. Male. *Mental Status and Dementia Tests. Organ Size. Scotland/ep [Epidemiology]. Ultrasonography, Doppler/td [Trends].
Year of Publication
2018

Link to the Ovid Full Text or citation:
Click here for full text options

Link to the External Link Resolver:
SFX

17.

Carotid circumferential wall stress is not associated with cognitive performance among individuals in late middle age: The Maastricht Study.
Geijselaers SL; Sep SJ; Schram MT; van Boxtel MP; van Sloten TT; Op Het Roodt J; Henry RM; Reesink KD; Schaper NC; Dagnelie PC; van der Kallen CJ; Biessels GJ; Stehouwer CD.
Atherosclerosis. 276:15-22, 2018 09.
[Journal Article. Research Support, Non-U.S. Gov't]
UI: 30006323
BACKGROUND AND AIMS: Arterial remodelling aims at normalising circumferential wall stress (CWS). Greater CWS in the carotid artery has previously been associated with the prevalence and severity of cerebral small vessel disease, a major cause of ageing-related cognitive decline. Here we test the hypothesis that greater carotid CWS is associated with poorer cognitive performance. METHODS: We studied 722 individuals (60+/-8 years, 55% men, 42.5% highly educated, blood pressure 137+/-19/77+/-11mmHg, n=197 with type 2 diabetes) who completed a neuropsychological assessment and underwent vascular ultrasound to measure the intima-media thickness (IMT) and interadventitial diameter (IAD) of the left common carotid artery at a plaque-free site. From IMT and IAD, lumen diameter (LD) was calculated. These structural measures were then combined with local carotid pulse pressure and brachial mean arterial pressure to obtain a measure of pulsatile (CWSpulsatile) and average (CWSmean) mechanical load on the vessel wall. Cognitive domains assessed were memory, executive function and attention, and processing speed.

RESULTS: After adjustment for age, sex, and education, regression analyses showed that neither CWSpulsatile nor CWSmean were associated with measures of cognitive performance (p-values >=0.31). This null association did not differ by age or educational level, and was observed in both individuals with and without carotid plaque, diabetes and/or hypertension. In addition, none of the individual measures of carotid structure (i.e. IMT, IAD, and LD) was related to cognitive performance.
CONCLUSIONS: The present cross-sectional study shows that carotid CWS is not associated with cognitive performance, at least not among relatively highly educated individuals in late middle age with adequately controlled cardiovascular risk factors.
Copyright © 2018 The Authors. Published by Elsevier B.V. All rights reserved.
Version ID
1
Record Owner
From MEDLINE, a database of the U.S. National Library of Medicine.
Status
MEDLINE
Authors Full Name
Geijselaers, Stefan Lc; Sep, Simone Js; Schram, Miranda T; van Boxtel, Martin Pj; van Sloten, Thomas T; Op Het Roodt, Jos; Henry, Ronald Ma; Reesink, Koen D; Schaper, Nicolaas C; Dagnelie, Pieter C; van der Kallen, Carla Jh; Biessels, Geert Jan; Stehouwer, Coen DA.
Institution
Geijselaers, Stefan Lc. Department of Internal Medicine, Maastricht University Medical Centre +, Maastricht, the Netherlands; CARIM School for Cardiovascular Diseases, Maastricht University, Maastricht, the Netherlands; Department of Neurology, Brain Centre Rudolf Magnus, University Medical Centre Utrecht, Utrecht, the Netherlands. Electronic address: stefan.geijselaers@gmail.com. Sep, Simone Js. Department of Internal Medicine, Maastricht University Medical Centre +, Maastricht, the Netherlands; CARIM School for Cardiovascular Diseases, Maastricht University, Maastricht, the Netherlands.
Schram, Miranda T. Department of Internal Medicine, Maastricht University Medical Centre +, Maastricht, the Netherlands; CARIM School for Cardiovascular Diseases, Maastricht University, Maastricht, the Netherlands.
van Boxtel, Martin Pj. Department of Psychiatry and Neuropsychology and MHeNS School for Mental Health and Neuroscience, Maastricht University Medical Centre +, Maastricht, the Netherlands.
van Sloten, Thomas T. Department of Internal Medicine, Maastricht University Medical Centre +, Maastricht, the Netherlands; CARIM School for Cardiovascular Diseases, Maastricht University, Maastricht, the Netherlands.

Op Het Roodt, Jos. Department of Internal Medicine, Maastricht University Medical Centre +, Maastricht, the Netherlands; CARIM School for Cardiovascular Diseases, Maastricht University, Maastricht, the Netherlands.
Henry, Ronald Ma. Department of Internal Medicine, Maastricht University Medical Centre +, Maastricht, the Netherlands; CARIM School for Cardiovascular Diseases, Maastricht University, Maastricht, the Netherlands.
Reesink, Koen D. Department of Biomedical Engineering, Maastricht University Medical Centre +, Maastricht, the Netherlands.
Schaper, Nicolaas C. Department of Internal Medicine, Maastricht University Medical Centre +, Maastricht, the Netherlands; CARIM School for Cardiovascular Diseases, Maastricht University, Maastricht, the Netherlands; CAPHRI School for Public Health and Primary Care, Maastricht University, Maastricht, the Netherlands.
Dagnelie, Pieter C. CARIM School for Cardiovascular Diseases, Maastricht University, Maastricht, the Netherlands; CAPHRI School for Public Health and Primary Care, Maastricht University, Maastricht, the Netherlands; Department of Epidemiology, Maastricht University, Maastricht, the Netherlands.
van der Kallen, Carla Jh. Department of Internal Medicine, Maastricht University Medical Centre +, Maastricht, the Netherlands; CARIM School for Cardiovascular Diseases, Maastricht University, Maastricht, the Netherlands.
Biessels, Geert Jan. Department of Neurology, Brain Centre Rudolf Magnus, University Medical Centre Utrecht, Utrecht, the Netherlands.
Stehouwer, Coen DA. Department of Internal Medicine, Maastricht University Medical Centre +, Maastricht, the Netherlands; CARIM School for Cardiovascular Diseases, Maastricht University, Maastricht, the Netherlands.
MeSH Heading
Age Factors. Aged. Attention. Blood Pressure. Carotid Arteries/dg [Diagnostic Imaging]. *Carotid Arteries/pp [Physiopathology]. Carotid Artery Diseases/dg [Diagnostic Imaging]. *Carotid Artery Diseases/pp [Physiopathology]. *Carotid Artery Diseases/px [Psychology]. Carotid Intima-Media Thickness. *Cognition. Cross-Sectional Studies. Educational Status. Executive Function. Female. Humans. Male. Memory. Middle Aged. Netherlands. Neuropsychological Tests. Risk Factors. Stress, Mechanical. *Vascular Remodeling.
Year of Publication
2018

Link to the Ovid Full Text or citation:
Click here for full text options

Link to the External Link Resolver:
SFX

18.

Cognitive function of patients with rheumatoid arthritis is associated with disease activity but not carotid atherosclerotic changes.
Lee JH; Kim GT; Kim YK; Lee SG.
Clinical & Experimental Rheumatology. 36(5):856-861, 2018 Sep-Oct.
[Journal Article]
UI: 29652660
OBJECTIVES: Although the relationship between atherosclerosis and cognitive impairment has been studied and replicated, whether cognitive deficits in RA can be attributed to atherosclerotic changes is not well understood. This study investigated cognitive function in patients with RA and evaluated whether cognitive function was affected by carotid arterial atherosclerosis.
METHODS: We examined 70 RA patients and 40 healthy controls. RA activity was assessed by disease activity score with 28 joint-erythrocyte sedimentation rate (DAS28-ESR). Cognitive function was assessed by the Korean version of the Consortium to Establish a Registry for Alzheimer's disease (CERAD-K) neuropsychological battery. Carotid arteries were scanned for the presence of plaques and to assess intima-media thickness (IMT). We assessed potential risk factors of cognitive impairment in RA patients using regression analyses.
RESULTS: There was a significant difference between RA patients and healthy controls in the verbal fluency (p=0.004) and Boston naming test (p=0.035). Carotid ultrasound revealed significantly more plaque in RA patients than in healthy controls (p=0.017). RA patients with memory impairment had significantly higher DAS28-ESR scores (p<0.001), age (p=0.009), and mean cIMT (p=0.027) than RA patients without memory impairment. In multivariable regression analysis, CERAD-K total score showed a significant negative correlation with age (beta=-0.415, p<0.001) or DAS28-ESR (beta=-4.685, p<0.001), but no correlation was found between CERAD-K total score and presence of plaque or cIMT.
CONCLUSIONS: Our results indicate that disease activity of RA and aging contribute to cognitive dysfunction, but there was no association between cognitive function and carotid atherosclerotic changes in RA patients.
Version ID

1

Record Owner

From MEDLINE, a database of the U.S. National Library of Medicine.

Status

MEDLINE

Authors Full Name

Lee, Ji Hyun; Kim, Geun-Tae; Kim, Yun-Kyung; Lee, Seung-Geun.

Institution

Lee, Ji Hyun. Division of Rheumatology, Department of Internal Medicine, Maryknoll Medical Center, Busan, Republic of Korea. Kim, Geun-Tae. Division of Rheumatology, Department of Internal Medicine, Kosin University College of Medicine, Busan, Republic of Korea. gtah@hanmail.net.

Kim, Yun-Kyung. Division of Rheumatology, Department of Internal Medicine, Kosin University College of Medicine, Busan, Republic of Korea.

Lee, Seung-Geun. Division of Rheumatology, Department of Internal Medicine, Pusan National University Hospital, Busan, Republic of Korea.

MeSH Heading

Age Factors. Aged. Aging/px [Psychology]. *Arthritis, Rheumatoid/co [Complications]. Arthritis, Rheumatoid/di [Diagnosis]. Arthritis, Rheumatoid/px [Psychology]. *Carotid Artery Diseases/co [Complications]. Carotid Artery Diseases/dg [Diagnostic Imaging]. Carotid Artery Diseases/px [Psychology]. Carotid Intima-Media Thickness. Case-Control Studies. *Cognition. Cognition Disorders/di [Diagnosis]. *Cognition Disorders/et [Etiology]. Cognition Disorders/px [Psychology]. Female. Humans. Male. Memory. Middle Aged. Neuropsychological Tests. Plaque, Atherosclerotic. Risk Factors. Verbal Behavior.

Year of Publication

2018

Link to the Ovid Full Text or citation:

Click here for full text options

Link to the External Link Resolver:

SFX

22.

Association of subclinical carotid atherosclerosis with immediate memory and other cognitive functions.
Matsumoto L; Suzuki K; Mizuno Y; Ohike Y; Ozeki A; Ono S; Takanashi M; Sawaki D; Suzuki T; Yamazaki T; Tsuji S; Iwata A.
Geriatrics & gerontology international. 18(1):65-71, 2018 Jan.
[Journal Article]
UI: 28776906
AIM: To clarify whether carotid atherosclerosis and its risk factors are associated with cognitive decline.
METHODS: We evaluated 206 individuals who visited our center for health screening. We carried out physical examinations, blood tests, intima-media thickness (IMT) measurement by carotid ultrasonography, brain magnetic resonance imaging scanning and cognitive function assessments. A total of 30 individuals, who had significant cerebrovascular lesions detected in magnetic resonance imaging scans, were excluded. To detect early cognitive decline, we defined "cognitive impairment (CI)" when a patient satisfied at least one of three criteria. These were Mini-Mental State Examination score <24, clock-drawing test score <4 coexisting with forgetfulness and Wechsler Memory Scale-revised delayed recall score below the normal range for the duration of education (>16 years of education: >=9, 10-15 years: >=5, 0-9 years: >=3).
RESULTS: Among 176 individuals, 27 were placed in the CI group. IMT was significantly higher in the CI group as compared with the non-CI group (mean +/- SD: 2.0 +/- 1.0 vs 1.7 +/- 0.7, P = 0018 by Student's t-test). Other atherosclerotic risk factors, such as blood pressure, low-density lipoprotein cholesterol, and hemoglobin A1c, were not significantly different between the two groups. In multivariate analysis, maximum IMT was associated with impaired immediate recall score on Wechsler Memory Scale-revised, independent of the presence of deep white matter hyperintensities on the magnetic resonance imaging scan.
CONCLUSIONS: Subclinical carotid atherosclerosis, defined as thickened IMT, could be a marker for early stages of CI, especially for immediate memory recall. The impairment is presumably caused by inducing cerebral microvascular dysfunction in the frontal lobe. Geriatr Gerontol Int 2018; 18: 65-71.
Copyright © 2017 Japan Geriatrics Society.
Version ID
1
Record Owner
From MEDLINE, a database of the U.S. National Library of Medicine.
Status

MEDLINE
Author NameID
Iwata, Atsushi; ORCID: http://orcid.org/0000-0001-7308-5314
Authors Full Name
Matsumoto, Lumine; Suzuki, Kazushi; Mizuno, Yoshiko; Ohike, Yumiko; Ozeki, Atsuko; Ono, Satoshi; Takanashi, Mikio; Sawaki, Daigo; Suzuki, Toru; Yamazaki, Tsutomu; Tsuji, Shoji; Iwata, Atsushi.
Institution
Matsumoto, Lumine. Department of Neurology, The University of Tokyo Hospital, Tokyo, Japan.
Matsumoto, Lumine. Center for Epidemiology and Preventive Medicine, The University of Tokyo Hospital, Tokyo, Japan.
Suzuki, Kazushi. Department of Neurology, The University of Tokyo Hospital, Tokyo, Japan.
Suzuki, Kazushi. Center for Epidemiology and Preventive Medicine, The University of Tokyo Hospital, Tokyo, Japan.
Mizuno, Yoshiko. Center for Epidemiology and Preventive Medicine, The University of Tokyo Hospital, Tokyo, Japan.
Ohike, Yumiko. Center for Epidemiology and Preventive Medicine, The University of Tokyo Hospital, Tokyo, Japan.
Ozeki, Atsuko. Center for Epidemiology and Preventive Medicine, The University of Tokyo Hospital, Tokyo, Japan.
Ono, Satoshi. Center for Epidemiology and Preventive Medicine, The University of Tokyo Hospital, Tokyo, Japan.
Takanashi, Mikio. Center for Epidemiology and Preventive Medicine, The University of Tokyo Hospital, Tokyo, Japan.
Sawaki, Daigo. Center for Epidemiology and Preventive Medicine, The University of Tokyo Hospital, Tokyo, Japan.
Suzuki, Toru. Center for Epidemiology and Preventive Medicine, The University of Tokyo Hospital, Tokyo, Japan.
Yamazaki, Tsutomu. Center for Epidemiology and Preventive Medicine, The University of Tokyo Hospital, Tokyo, Japan.
Tsuji, Shoji. Department of Neurology, The University of Tokyo Hospital, Tokyo, Japan.
Iwata, Atsushi. Department of Neurology, The University of Tokyo Hospital, Tokyo, Japan.
MeSH Heading
Carotid Artery Diseases/di [Diagnosis]. *Carotid Artery Diseases/px [Psychology]. *Carotid Intima-Media Thickness. Cognition Disorders/ep [Epidemiology]. Humans. Memory Disorders/ep [Epidemiology]. Memory, Short-Term. Risk Factors.
Keyword Heading

atherosclerosis cognitive function
immediate memory
intima-media thickness.
Year of Publication
2018

Link to the Ovid Full Text or citation:
[Click here for full text options]

Link to the External Link Resolver:
[SFX]

23.

Carotid atherosclerotic plaque instability and cognition determined by ultrasound-measured plaque strain in asymptomatic patients with significant stenosis.
Dempsey RJ; Varghese T; Jackson DC; Wang X; Meshram NH; Mitchell CC; Hermann BP; Johnson SC; Berman SE; Wilbrand SM.
Journal of Neurosurgery. 128(1):111-119, 2018 01.
[Clinical Trial. Journal Article. Research Support, N.I.H., Extramural]
UI: 28298048
OBJECTIVE This article describes the use of ultrasound measurements of physical strain within carotid atherosclerotic plaques as a measure of instability and the potential for vascular cognitive decline, microemboli, and white matter changes. METHODS Asymptomatic patients with significant (> 60%) carotid artery stenosis were studied for dynamic measures of plaque instability, presence of microemboli, white matter changes, and vascular cognitive decline in comparison with normative controls and premorbid state. RESULTS Although classically asymptomatic, these patients showed vascular cognitive decline. The degree of strain instability measured within the atherosclerotic plaque directly predicted vascular cognitive decline in these patients thought previously to be asymptomatic according to classic criteria. Furthermore, 26% of patients showed microemboli, and patients had twice as much white matter hyperintensity as controls. CONCLUSIONS These data show that physical measures of plaque instability are possible through interpretation of ultrasound strain data during pulsation, which may be more clinically relevant

than solely measuring degree of stenosis. The data also highlight the importance of understanding that the definition of symptoms should not be limited to motor, speech, and vision function but underscore the role of vascular cognitive decline in the pathophysiology of carotid atherosclerotic disease. Clinical trial registration no.: NCT02476396 (clinicaltrials.gov).

Version ID

1

Record Owner

From MEDLINE, a database of the U.S. National Library of Medicine.

Status

MEDLINE

Authors Full Name

Dempsey, Robert J; Varghese, Tomy; Jackson, Daren C; Wang, Xiao; Meshram, Nirvedh H; Mitchell, Carol C; Hermann, Bruce P; Johnson, Sterling C; Berman, Sara E; Wilbrand, Stephanie M.

Institution

Dempsey, Robert J. Departments of1Neurological Surgery and. Varghese, Tomy. 2Medical Physics, University of Wisconsin School of Medicine and Public Health.

Jackson, Daren C. 3Wisconsin Surgical Outcomes Research Program, Department of Surgery, University of Wisconsin School of Medicine and Public Health, Madison, Wisconsin.

Wang, Xiao. 4Rutgers Cancer Institute of New Jersey, New Brunswick, New Jersey.

Meshram, Nirvedh H. 2Medical Physics, University of Wisconsin School of Medicine and Public Health.

Mitchell, Carol C. 5Department of Medicine, Cardiovascular Medicine Division.

Hermann, Bruce P. 6Department of Neurology, University of Wisconsin School of Medicine and Public Health; and.

Johnson, Sterling C. 7Alzheimer's Disease Research Center, University of Wisconsin School of Medicine and Public Health, Waisman Laboratory for Brain Injury and Behavior, University of Wisconsin-Madison & Geriatric Research Education & Clinical Center, William S. Middleton Veterans Hospital, Madison, Wisconsin.

Berman, Sara E. 7Alzheimer's Disease Research Center, University of Wisconsin School of Medicine and Public Health, Waisman Laboratory for Brain Injury and Behavior, University of Wisconsin-Madison & Geriatric Research Education & Clinical Center, William S. Middleton Veterans Hospital, Madison, Wisconsin.

Wilbrand, Stephanie M. Departments of1Neurological Surgery and.

Comments

Comment in (CIN)

MeSH Heading

Aged. Aged, 80 and over. Biomechanical Phenomena. *Carotid Stenosis/dg [Diagnostic Imaging]. *Carotid Stenosis/px [Psychology]. *Cognition. Cognitive Dysfunction/dg [Diagnostic Imaging]. Cognitive Dysfunction/px [Psychology]. Constriction, Pathologic/dg [Diagnostic Imaging]. Constriction, Pathologic/px [Psychology]. Dementia, Vascular/dg [Diagnostic Imaging]. Dementia, Vascular/px [Psychology]. Female. Humans. Image Interpretation, Computer-Assisted. Magnetic Resonance Imaging. Male. Middle Aged. Neuropsychological Tests. *Plaque, Atherosclerotic/dg [Diagnostic Imaging]. *Plaque, Atherosclerotic/px [Psychology]. Prodromal Symptoms. Severity of Illness Index. *Ultrasonography. White Matter/dg [Diagnostic Imaging].

Keyword Heading

*CA = carotid artery   *CAS = CA stenosis

*HITS = high-intensity transient signal

*MCA = middle cerebral artery

*TCD = transcranial Doppler

*TIA = transient ischemic attack

*WMH = white matter hyperintensity

*carotid atherosclerosis

*plaque instability

*stroke

*ultrasound

*ultrasound strain

*vascular cognitive decline

*vascular disorders.

Year of Publication

2018

Link to the Ovid Full Text or citation:

Click here for full text options

Link to the External Link Resolver:

SFX

24.

Carotid Atherosclerosis and Cognitive Impairment in Nonstroke Patients. [Review]
Chen WH; Jin W; Lyu PY; Liu Y; Li R; Hu M; Xiao XJ.
Chinese Medical Journal. 130(19):2375-2379, 2017 Oct 05.
[Journal Article. Review]
UI: 28937045
OBJECTIVE: As a vascular risk factor, carotid atherosclerosis is crucial to cognitive impairment. While carotid intima-media thickness, carotid artery plaque, and carotid stenosis can reflect carotid atherosclerosis in different stages, this review aimed to explore researches on the role of carotid intima-media thickness, carotid artery plaque, and carotid stenosis in the progress of cognitive impairment in nonstroke patients and tried to illustrate the possible mechanisms.
DATA SOURCES: We searched the PubMed database for recently published research articles up to July 2017, with the key words of "carotid atherosclerosis," "carotid intima-media thickness," "carotid plaque," "carotid stenosis," "nonstroke," and "cognitive impairment."
STUDY SELECTION: Articles were obtained and reviewed to analyze the role of carotid atherosclerosis such as carotid intima-thickness, carotid plaque, and carotid stenosis in the progress of cognitive impairment in nonstroke patients and the possible mechanisms.
RESULTS: In recent years, most studies proved that by evaluating carotid atherosclerosis with ultrasonography, carotid atherosclerosis accounts for the development of cognitive decline in nonstroke patients. Carotid atherosclerosis not only impairs the subtle general cognitive function but also decreases the specific domains of cognitive function, such as memory, motor function, visual perception, attention, and executive function. But, it is still controversial. The possible mechanisms of cognitive impairment in nonstroke patients with carotid atherosclerosis can be classified as systemic global cerebrovascular function, small-vessel diseases, and the mixed lesions.
CONCLUSIONS: Carotid atherosclerosis can be used to predict the risk of cognitive impairment. Furthermore, diagnosing and treating carotid atherosclerosis at early stage might help clinicians prevent and treat vascular cognitive impairment in nonstroke patients.
Version ID
1
Record Owner
From MEDLINE, a database of the U.S. National Library of Medicine.
Status
MEDLINE
Authors Full Name
Chen, Wei-Hong; Jin, Wei; Lyu, Pei-Yuan; Liu, Yang; Li, Rui; Hu, Ming; Xiao, Xiang-Jian.
Institution

Chen, Wei-Hong. Graduate School, Hebei Medical University; Department of Neurology, Hebei General Hospital, Shijiazhuang, Hebei 050051, China. Jin, Wei. Department of Neurology, Hebei General Hospital, Shijiazhuang, Hebei 050051, China.
Lyu, Pei-Yuan. Graduate School, Hebei Medical University; Department of Neurology, Hebei General Hospital, Shijiazhuang, Hebei 050051, China.
Liu, Yang. Graduate School, Hebei Medical University; Department of Neurology, Hebei General Hospital, Shijiazhuang, Hebei 050051, China.
Li, Rui. Graduate School, Hebei Medical University; Department of Neurology, Hebei General Hospital, Shijiazhuang, Hebei 050051, China.
Hu, Ming. Department of Neurology, Hebei General Hospital, Shijiazhuang, Hebei 050051, China.
Xiao, Xiang-Jian. Department of Neurology, Hebei General Hospital, Shijiazhuang, Hebei 050051, China.
MeSH Heading
*Carotid Artery Diseases/pp [Physiopathology]. Carotid Intima-Media Thickness. Carotid Stenosis/pp [Physiopathology]. Cognition/ph [Physiology]. Cognitive Dysfunction/pp [Physiopathology]. Female. Humans. Male.
Year of Publication
2017

Link to the Ovid Full Text or citation:
Click here for full text options

Link to the External Link Resolver:
SFX

25.

Asymptomatic carotid stenosis is associated with cognitive impairment.
Lal BK; Dux MC; Sikdar S; Goldstein C; Khan AA; Yokemick J; Zhao L.
Journal of Vascular Surgery. 66(4):1083-1092, 2017 10.
[Journal Article. Research Support, N.I.H., Extramural. Research Support, U.S. Gov't, Non-P.H.S.]
UI: 28712815

BACKGROUND: Cerebrovascular risk factors (eg, hypertension, coronary artery disease) and stroke can lead to vascular cognitive impairment. The Asymptomatic Carotid Stenosis and Cognitive Function study evaluated the isolated impact of asymptomatic carotid stenosis (no prior ipsilateral or contralateral stroke or transient ischemic attack) on cognitive function. Cerebrovascular hemodynamic and carotid plaque characteristics were analyzed to elucidate potential mechanisms affecting cognition.
METHODS: There were 82 patients with >=50% asymptomatic carotid stenosis and 62 controls without stenosis but matched for vascular comorbidities who underwent neurologic, National Institutes of Health Stroke Scale, and comprehensive neuropsychological examination. Overall cognitive function and five domain-specific scores were computed. Duplex ultrasound with Doppler waveform and B-mode imaging defined the degree of stenosis, least luminal diameter, plaque area, and plaque gray-scale median. Breath-holding index (BHI) and microembolization were measured using transcranial Doppler. We assessed cognitive differences between stenosis patients and control patients and of stenosis patients with low vs high BHI and correlated cognitive function with microembolic counts and plaque characteristics.
RESULTS: Stenosis and control patients did not differ in vascular risk factors, education, estimated intelligence, or depressive symptoms. Stenosis patients had worse composite cognitive scores (P = .02; Cohen's d = 0.43) and domain-specific scores for learning/memory (P = .02; d = 0.42) and motor/processing speed (P = .01; d = 0.65), whereas scores for executive function were numerically lower (P = .08). Approximately 49.4% of all stenosis patients were impaired in at least two cognitive domains. Precisely 50% of stenosis patients demonstrated a reduced BHI. Stenosis patients with reduced BHI performed worse on the overall composite cognitive score (t = -2.1; P = .02; d = 0.53) and tests for learning/memory (t = -2.7; P = .01; d = 0.66). Cognitive function did not correlate with measures of plaque burden (degree of stenosis, least luminal diameter, and plaque area) or with plaque gray-scale median.
CONCLUSIONS: Asymptomatic carotid stenosis is associated with cognitive impairment independent of known vascular risk factors for vascular cognitive impairment. Approximately 49.4% of these patients demonstrate impairment in at least two neuropsychological domains. The deficit is driven primarily by reduced motor/processing speed and learning/memory and is mild to moderate in severity. The mechanism for impairment is likely to be hemodynamic as evidenced by reduced cerebrovascular reserve and the likely result of hypoperfusion from a pressure drop across the stenosis in the presence of inadequate collateralization.
Copyright © 2017 Society for Vascular Surgery. Published by Elsevier Inc. All rights reserved.
Version ID
1
Record Owner
From MEDLINE, a database of the U.S. National Library of Medicine.

Status

MEDLINE

Authors Full Name

Lal, Brajesh K; Dux, Moira C; Sikdar, Siddhartha; Goldstein, Carly; Khan, Amir A; Yokemick, John; Zhao, Limin.

Institution

Lal, Brajesh K. Department of Vascular Surgery, University of Maryland School of Medicine, Baltimore, Md; Vascular Service, Veterans Affairs Medical Center, Baltimore, Md. Electronic address: blal@som.umaryland.edu. Dux, Moira C. Neuropsychology Section, Veterans Affairs Medical Center, Baltimore, Md.

Sikdar, Siddhartha. Department of Bioengineering, George Mason University, Fairfax, Va.

Goldstein, Carly. Department of Vascular Surgery, University of Maryland School of Medicine, Baltimore, Md; Vascular Service, Veterans Affairs Medical Center, Baltimore, Md.

Khan, Amir A. Department of Vascular Surgery, University of Maryland School of Medicine, Baltimore, Md; Department of Bioengineering, George Mason University, Fairfax, Va.

Yokemick, John. Department of Vascular Surgery, University of Maryland School of Medicine, Baltimore, Md.

Zhao, Limin. Department of Vascular Surgery, University of Maryland School of Medicine, Baltimore, Md; Vascular Service, Veterans Affairs Medical Center, Baltimore, Md.

MeSH Heading

Aged. Asymptomatic Diseases. Attention. Carotid Arteries/dg [Diagnostic Imaging]. Carotid Arteries/pp [Physiopathology]. *Carotid Arteries. *Carotid Stenosis/co [Complications]. Carotid Stenosis/dg [Diagnostic Imaging]. Carotid Stenosis/pp [Physiopathology]. Carotid Stenosis/px [Psychology]. Case-Control Studies. Cerebrovascular Circulation. *Cognition. Cognition Disorders/di [Diagnosis]. *Cognition Disorders/et [Etiology]. Cognition Disorders/pp [Physiopathology]. Cognition Disorders/px [Psychology]. Executive Function. Female. Humans. Intracranial Embolism/dg [Diagnostic Imaging]. *Intracranial Embolism/et [Etiology]. Intracranial Embolism/pp [Physiopathology]. Intracranial Embolism/px [Psychology]. Male. Memory. Middle Aged. Motor Activity. Neurologic Examination. Neuropsychological Tests. Plaque, Atherosclerotic. Prospective Studies. Risk Factors. Severity of Illness Index. Ultrasonography, Doppler, Duplex. Ultrasonography, Doppler, Transcranial.

Year of Publication

2017

Link to the Ovid Full Text or citation:

Click here for full text options

Link to the External Link Resolver:
SFX

29.

Carotid disease at age 73 and cognitive change from age 70 to 76 years: A longitudinal cohort study.
Wardlaw JM; Allerhand M; Eadie E; Thomas A; Corley J; Pattie A; Taylor A; Shenkin SD; Cox S; Gow A; Starr JM; Deary IJ.
Journal of Cerebral Blood Flow & Metabolism. 37(8):3042-3052, 2017 Aug.
[Journal Article]
UI: 28155579
Cognitive decline and carotid artery atheroma are common at older ages. In community-dwelling subjects, we assessed cognition at ages 70, 73 and 76 and carotid Doppler ultrasound at age 73, to determine whether carotid stenosis was related to cognitive decline. We used latent growth curve models to examine associations between four carotid measures (internal carotid artery stenosis, velocity, pulsatility and resistivity indices) and four cognitive ability domains (memory, visuospatial function, crystallised intelligence, processing speed) adjusted for cognitive ability at age 11, current age, gender and vascular risk factors. Amongst 866 participants, carotid stenosis (median 12.96%) was not associated with cognitive abilities at age 70 or cognitive decline from age 70 to 76. Increased ICA pulsatility and resistivity indices were associated with slower processing speed (both P < 0.001) and worse visuospatial function (P = 0.036, 0.031, respectively) at age 70, and declining crystallised intelligence from ages 70 to 76 (P = 0.008, 0.006, respectively). The findings suggest that vascular stiffening, rather than carotid luminal narrowing, adversely influences cognitive ageing and provides a potential target for ameliorating age-related cognitive decline.
Version ID
1
Record Owner
From MEDLINE, a database of the U.S. National Library of Medicine.
Status
MEDLINE
Authors Full Name

Wardlaw, Joanna M; Allerhand, Michael; Eadie, Elizabeth; Thomas, Avril; Corley, Janey; Pattie, Alison; Taylor, Adele; Shenkin, Susan D; Cox, Simon; Gow, Alan; Starr, John M; Deary, Ian J.

Institution

Wardlaw, Joanna M. 1 Brain Research Imaging Centre, Centre for Clinical Brain Sciences, University of Edinburgh, Edinburgh, UK. Wardlaw, Joanna M. 2 Department of Neuroradiology, NHS Lothian, Western General Hospital, Edinburgh, UK.

Wardlaw, Joanna M. 3 Centre for Cognitive Ageing and Cognitive Epidemiology, University of Edinburgh, Edinburgh, UK.

Allerhand, Michael. 3 Centre for Cognitive Ageing and Cognitive Epidemiology, University of Edinburgh, Edinburgh, UK.

Eadie, Elizabeth. 2 Department of Neuroradiology, NHS Lothian, Western General Hospital, Edinburgh, UK.

Thomas, Avril. 2 Department of Neuroradiology, NHS Lothian, Western General Hospital, Edinburgh, UK.

Corley, Janey. 3 Centre for Cognitive Ageing and Cognitive Epidemiology, University of Edinburgh, Edinburgh, UK.

Pattie, Alison. 3 Centre for Cognitive Ageing and Cognitive Epidemiology, University of Edinburgh, Edinburgh, UK.

Taylor, Adele. 3 Centre for Cognitive Ageing and Cognitive Epidemiology, University of Edinburgh, Edinburgh, UK.

Shenkin, Susan D. 3 Centre for Cognitive Ageing and Cognitive Epidemiology, University of Edinburgh, Edinburgh, UK.

Shenkin, Susan D. 4 Geriatric Medicine, University of Edinburgh, Royal Infirmary, Edinburgh, UK.

Cox, Simon. 3 Centre for Cognitive Ageing and Cognitive Epidemiology, University of Edinburgh, Edinburgh, UK.

Gow, Alan. 3 Centre for Cognitive Ageing and Cognitive Epidemiology, University of Edinburgh, Edinburgh, UK.

Gow, Alan. 5 Department of Psychology, Heriot-Watt University, Edinburgh, UK.

Starr, John M. 3 Centre for Cognitive Ageing and Cognitive Epidemiology, University of Edinburgh, Edinburgh, UK.

Deary, Ian J. 3 Centre for Cognitive Ageing and Cognitive Epidemiology, University of Edinburgh, Edinburgh, UK.

MeSH Heading

Aged. Carotid Stenosis/dg [Diagnostic Imaging]. Carotid Stenosis/en [Enzymology]. *Carotid Stenosis/px [Psychology]. *Cognition/ph [Physiology]. Cognition Disorders/ep [Epidemiology]. *Cognition Disorders/px [Psychology]. Cognitive Aging/ph [Physiology]. *Cognitive Aging/px [Psychology]. Female. Humans. Longitudinal Studies. Male. Models, Neurological. Plaque,

Atherosclerotic/dg [Diagnostic Imaging]. Plaque, Atherosclerotic/ep [Epidemiology]. *Plaque, Atherosclerotic/px [Psychology]. Ultrasonography, Doppler, Color.

Keyword Heading

Carotid stenosis ageing

cognition

vascular risk factors

white matter hyperintensities.

Year of Publication

2017

Link to the Ovid Full Text or citation:

Click here for full text options

Link to the External Link Resolver:

SFX

35.

Atherosclerotic carotid stenosis and cognitive function. [Review]

Wang T; Mei B; Zhang J.

Clinical Neurology & Neurosurgery. 146:64-70, 2016 Jul.

[Journal Article. Review]

UI: 27152468

Atherosclerosis carotid stenosis is associated with stroke and cognitive impairment. Progressive cognitive decline may be an even greater problem than stroke, but it has not been widely recognized and therefore must be adequately addressed. Although both Carotid Endarterectomy (CEA) and Carotid Artery Stenting (CAS) have been proven can prevent future stroke in patients with atherosclerotic carotid stenosis, the influence of CEA and CAS on cognitive function is not clear. In the first part of this review, we evaluated the literature concerning carotid stenosis and the risk of cognitive impairment. Studies have suggested that both symptomatic and asymptomatic carotid stenosis are associated with cognitive impairment. In the second part, we reviewed the impact of CEA and CAS on cognitive function, some studies have shown benefits, but others have not.

Copyright © 2016 Elsevier B.V. All rights reserved.
Version ID
1
Record Owner
From MEDLINE, a database of the U.S. National Library of Medicine.
Status
MEDLINE
Authors Full Name
Wang, Tao; Mei, Bin; Zhang, Junjian.
Institution
Wang, Tao. Department of Neurology and Neuropsychological Research Center, Zhongnan Hospital, Wuhan University, Wuhan, China. Mei, Bin. Department of Neurology and Neuropsychological Research Center, Zhongnan Hospital, Wuhan University, Wuhan, China. Zhang, Junjian. Department of Neurology and Neuropsychological Research Center, Zhongnan Hospital, Wuhan University, Wuhan, China. Electronic address: 851895243@qq.com.
MeSH Heading
*Atherosclerosis/co [Complications]. *Carotid Stenosis/co [Complications]. *Cognitive Dysfunction/et [Etiology]. Humans.
Keyword Heading
Carotid artery stenting Carotid endarterectomy
Carotid stenosis
Cognitive function
Revascularization.
Year of Publication
2016

Link to the Ovid Full Text or citation:
Click here for full text options

Link to the External Link Resolver:
SFX

37.

Carotid atherosclerosis, cytomegalovirus infection, and cognitive decline in the very old: a community-based prospective cohort study.
Kawasaki M; Arai Y; Takayama M; Hirata T; Takayama M; Abe Y; Niimura H; Mimura M; Takebayashi T; Hirose N.
Age. 38(2):29, 2016 Apr.
[Journal Article. Multicenter Study. Observational Study. Research Support, Non-U.S. Gov't]
UI: 26886582
To investigate various risk factors of cognitive decline in the very old, we studied 494 subjects over 85 years old without diagnosis of dementia at baseline from the Tokyo Oldest Old Survey on Total Health, an ongoing, community-based cohort in Japan. Cognitive function was assessed at baseline and at 3-year follow-up using Mini-Mental State Examination (MMSE). Plasma samples were assayed for levels of cytomegalovirus (CMV) immunoglobulin G (IgG) antibodies, tumor necrosis factor-alpha, interleukin-6, and blood chemistry. Carotid artery plaques were measured using an ultrasonography. In the cross-sectional analyses using Tobit regression, individuals with high carotid artery plaque score (>=5.0) had MMSE scores that were 1.08 points lower compared to those with no plaque (95 % confidence interval (CI) -1.95 to -0.20; p = 0.016), adjusted for age, sex, and education. Individuals with CMV IgG titers in the highest quartile had MMSE scores that were 1.47 points lower compared to individuals in the lowest quartile (95 % CI -2.44 to -0.50; p = 0.003). CMV and carotid atherosclerosis showed evidence of an interaction, where the association between CMV and MMSE was present only in subjects with carotid artery plaque. In the longitudinal analyses using linear regression, carotid atherosclerosis, smoking, low grip strength, and poor activities of daily living (ADL) status were associated with faster cognitive decline, adjusted for age, sex, education, and baseline cognitive function. Our findings suggest that carotid atherosclerosis is consistently associated with low cognitive function in the very old and modifies the association between latent CMV infection and cognition.
Version ID
1
Record Owner
From MEDLINE, a database of the U.S. National Library of Medicine.
Status
MEDLINE
Authors Full Name
Kawasaki, Midori; Arai, Yasumichi; Takayama, Michiyo; Hirata, Takumi; Takayama, Midori; Abe, Yukiko; Niimura, Hidehito; Mimura, Masaru; Takebayashi, Toru; Hirose, Nobuyoshi.
Institution

Kawasaki, Midori. Center for Supercentenarian Medical Research, Keio University School of Medicine, 35 Shinanomachi, Shinjuku-ku, Tokyo, 160-8582, Japan. Arai, Yasumichi. Center for Supercentenarian Medical Research, Keio University School of Medicine, 35 Shinanomachi, Shinjuku-ku, Tokyo, 160-8582, Japan. yasumich@keio.jp.
Takayama, Michiyo. Center for Preventive Medicine, Keio University School of Medicine, 35 Shinanomachi, Shinjuku-ku, Tokyo, 160-8582, Japan.
Hirata, Takumi. Center for Supercentenarian Medical Research, Keio University School of Medicine, 35 Shinanomachi, Shinjuku-ku, Tokyo, 160-8582, Japan.
Takayama, Midori. Faculty of Science and Technology, Keio University, 4-1-1 Hiyoshi, Kohoku-ku, Yokohama, Kanagawa, 252-8520, Japan.
Abe, Yukiko. Center for Supercentenarian Medical Research, Keio University School of Medicine, 35 Shinanomachi, Shinjuku-ku, Tokyo, 160-8582, Japan.
Niimura, Hidehito. Department of Neuropsychiatry, Keio University School of Medicine, 35 Shinanomachi, Shinjuku-ku, Tokyo, 160-8582, Japan.
Mimura, Masaru. Department of Neuropsychiatry, Keio University School of Medicine, 35 Shinanomachi, Shinjuku-ku, Tokyo, 160-8582, Japan.
Takebayashi, Toru. Department of Preventative Medicine and Public Health, Keio University School of Medicine, 35 Shinanomachi, Shinjuku-ku, Tokyo, 160-8582, Japan.
Hirose, Nobuyoshi. Center for Supercentenarian Medical Research, Keio University School of Medicine, 35 Shinanomachi, Shinjuku-ku, Tokyo, 160-8582, Japan.
MeSH Heading
Aged, 80 and over. *Aging. Carotid Artery Diseases/di [Diagnosis]. *Carotid Artery Diseases/ep [Epidemiology]. Cognition. Cognition Disorders/di [Diagnosis]. *Cognition Disorders/ep [Epidemiology]. Cross-Sectional Studies. Cytomegalovirus Infections/di [Diagnosis]. *Cytomegalovirus Infections/ep [Epidemiology]. Female. Follow-Up Studies. Humans. Incidence. Japan/ep [Epidemiology]. Male. Neuropsychological Tests. *Population Surveillance. Prospective Studies. Risk Factors. Time Factors.
Keyword Heading
Atherosclerosis Cognitive decline
Cytomegalovirus
Inflammation
Very old.
Year of Publication
2016

Link to the Ovid Full Text or citation:

Click here for full text options

Link to the External Link Resolver:
SFX

38.

Low carotid artery wall shear stress is independently associated with brain white-matter hyperintensities and cognitive impairment in older patients.
Liu Z; Zhao Y; Wang X; Zhang H; Cui Y; Diao Y; Xiu J; Sun X; Jiang G.
Atherosclerosis. 247:78-86, 2016 Apr.
[Journal Article. Research Support, Non-U.S. Gov't]
UI: 26868512
BACKGROUND & AIMS: Brain white-matter lesions and cognitive impairment are increasing because of the increasing number of patients aged >=80 y. Wall shear stress (WSS) plays a pivotal role as a fluid mechanical mediator in vascular reactivity and atherosclerosis. In this study, we investigated the associations among common carotid artery (CCA) WSS, white-matter lesions, and cognitive impairment in patients aged >=80 y
METHODS: We enrolled 384 patients aged >=80 y. All subjects had CCA-WSS, brain white-matter hyperintensities (WMH), and Mini-Mental State Examination (MMSE) assessments and were divided into three groups using tertiles of mean and peak CCA-WSS.
RESULTS: For groups classified by the tertile of mean CCA-WSS, WMH, and WMH fraction were decreased; the MMSE score increased from low to high in the respective groups. Differences in WMH, WMH fraction, and the MMSE score were significant between any two groups (all adjusted $p < 0.001$). Groups classified by the tertile of peak CCA-WSS had the same pattern. Mean and peak CCA-WSS were significantly and inversely correlated with WMH ($r = -0.575$ and -0.570, respectively; $p < 0.001$) and WMH fraction ($r = -0.574$ and -0.569, respectively; $p < 0.001$) but positively correlated with the MMSE score ($r = 0.390$ and 0.278, respectively; $p < 0.001$). Multiple linear backward stepwise regression indicated the mean and peak CCA-WSS were significantly and independently associated with WMH, WMH fraction, and the MMSE score (all adjusted $p < 0.001$).
CONCLUSION: Carotid artery WSS was independently associated with brain white-matter lesions and cognitive impairment in patients aged >=80 y.
Copyright © 2016 Elsevier Ireland Ltd. All rights reserved.
Version ID

1
Record Owner
From MEDLINE, a database of the U.S. National Library of Medicine.
Status
MEDLINE
Authors Full Name
Liu, Zhendong; Zhao, Yingxin; Wang, Xidi; Zhang, Hua; Cui, Yi; Diao, Yutao; Xiu, Jianchao; Sun, Xiaolin; Jiang, Guosheng.
Institution
Liu, Zhendong. Cardio-Cerebrovascular Control and Research Center, Institute of Basic Medicine, Shandong Academy of Medical Sciences, Jinan, Shandong, 250062, China. Electronic address: zhendongliu876@126.com. Zhao, Yingxin. Cardio-Cerebrovascular Control and Research Center, Institute of Basic Medicine, Shandong Academy of Medical Sciences, Jinan, Shandong, 250062, China.
Wang, Xidi. Department of Neurology, Zhangqiu People's Hospital, Zhangqiu, Shandong, 250200, China.
Zhang, Hua. Cardio-Cerebrovascular Control and Research Center, Institute of Basic Medicine, Shandong Academy of Medical Sciences, Jinan, Shandong, 250062, China.
Cui, Yi. Department of Radiology, Qilu Hospital of Shandong University, Jinan, Shandong, 250012, China.
Diao, Yutao. Cardio-Cerebrovascular Control and Research Center, Institute of Basic Medicine, Shandong Academy of Medical Sciences, Jinan, Shandong, 250062, China.
Xiu, Jianchao. Cardio-Cerebrovascular Control and Research Center, Institute of Basic Medicine, Shandong Academy of Medical Sciences, Jinan, Shandong, 250062, China.
Sun, Xiaolin. Cardio-Cerebrovascular Control and Research Center, Institute of Basic Medicine, Shandong Academy of Medical Sciences, Jinan, Shandong, 250062, China.
Jiang, Guosheng. Cardio-Cerebrovascular Control and Research Center, Institute of Basic Medicine, Shandong Academy of Medical Sciences, Jinan, Shandong, 250062, China. Electronic address: jiangguosh@163.com.
MeSH Heading
Age Factors. Aged, 80 and over. Blood Flow Velocity. *Carotid Artery Diseases/co [Complications]. Carotid Artery Diseases/dg [Diagnostic Imaging]. Carotid Artery Diseases/pp [Physiopathology]. Carotid Artery, Common/dg [Diagnostic Imaging]. *Carotid Artery, Common/pp [Physiopathology]. Carotid Intima-Media Thickness. *Cognition. Cognition Disorders/di [Diagnosis]. *Cognition Disorders/et [Etiology]. Cognition Disorders/px [Psychology]. Cross-Sectional Studies. Female. Humans. Leukoencephalopathies/dg [Diagnostic Imaging]. *Leukoencephalopathies/et [Etiology]. Linear Models. Magnetic Resonance Imaging. Male. Multivariate Analysis. Psychiatric

Status Rating Scales. Regional Blood Flow. Risk Factors. Stress, Mechanical. Ultrasonography, Doppler, Duplex.

Keyword Heading

Atherosclerosis   Cognitive function

Older patients

Wall shear stress

White matter lesions.

Year of Publication

2016

Link to the Ovid Full Text or citation:

Click here for full text options

Link to the External Link Resolver:

SFX

39.

Classification of Symptomatic and Asymptomatic Patients with and without Cognitive Decline Using Non-invasive Carotid Plaque Strain Indices as Biomarkers.

Wang X; Jackson DC; Mitchell CC; Varghese T; Wilbrand SM; Rocque BG; Hermann BP; Dempsey RJ.

Ultrasound in Medicine & Biology. 42(4):909-18, 2016 Apr.

[Journal Article. Research Support, N.I.H., Extramural. Research Support, Non-U.S. Gov't]

UI: 26778288

Vascular cognitive decline may be caused by micro-emboli generated by carotid plaque instability. We previously found that maximum strain indices in carotid plaque were significantly correlated with cognitive function. In the work described here, we examined these associations with a larger sample size, as well as evaluated the performance of these maximum strain indices in predicting cognitive impairment. Ultrasound-based strain imaging and cognition assessment were conducted on 75 human patients. Patients underwent one of two standardized cognitive test batteries, either the Repeatable Battery for the Assessment of Neuropsychological Status (RBANS) or the National Institute of Neurologic Disorder and Stroke-Canadian Stroke Network (NINDS-CSN) Vascular

Cognitive Impairment Harmonization Standards (60 min). Scores were standardized within each battery to allow these data to be combined across all participants. Radiofrequency signals for ultrasound strain imaging were acquired on the carotid arteries using either a Siemens Antares with a VFX 13-5 linear array transducer or a Siemens S2000 with an 18 L6 linear array transducer. The same hierarchical block-matching motion tracking algorithm developed in our laboratory was used to estimate accumulated axial, lateral, and shear strain indices in carotid plaque, with inclusion of adventitia regardless of the ultrasound system and transducer used. Associations between cognitive z-scores and maximum strain indices were examined using Pearson's correlation coefficients. Maximum strain indices were also employed to predict cognitive impairment using receiver operating characteristic analysis. All correlations between maximum strain indices and total cognition were statistically significant ($p < 0.05$), indicating that these indices have good utility in predicting cognitive impairment. Maximum lateral strain indices provided an area under the curve of 0.85 for symptomatic patients and 0.68 for asymptomatic patients. Our results indicate the important relationship of maximum strain indices to cognitive function and the feasibility of using maximum strain indices to predict cognitive decline with inclusion of the adventitia layer into the segmentation of plaque.

Copyright © 2016 World Federation for Ultrasound in Medicine & Biology. Published by Elsevier Inc. All rights reserved.

Version ID

1

Record Owner

From MEDLINE, a database of the U.S. National Library of Medicine.

Status

MEDLINE

Authors Full Name

Wang, Xiao; Jackson, Daren C; Mitchell, Carol C; Varghese, Tomy; Wilbrand, Stephanie M; Rocque, Brandon G; Hermann, Bruce P; Dempsey, Robert J.

Institution

Wang, Xiao. Department of Medical Physics, University of Wisconsin School of Medicine and Public Health, University of Wisconsin-Madison, Madison, Wisconsin, USA. Electronic address: xwang235@wisc.edu. Jackson, Daren C. Department of Neurology, University of Wisconsin School of Medicine and Public Health, University of Wisconsin-Madison, Madison, Wisconsin, USA.

Mitchell, Carol C. Department of Medicine, University of Wisconsin School of Medicine and Public Health, University of Wisconsin-Madison, Madison, Wisconsin, USA.

Varghese, Tomy. Department of Medical Physics, University of Wisconsin School of Medicine and Public Health, University of Wisconsin-Madison, Madison, Wisconsin, USA.

Wilbrand, Stephanie M. Department of Neurological Surgery, University of Wisconsin School of Medicine and Public Health, University of Wisconsin-Madison, Madison, Wisconsin, USA.
Rocque, Brandon G. Department of Neurological Surgery, University of Wisconsin School of Medicine and Public Health, University of Wisconsin-Madison, Madison, Wisconsin, USA.
Hermann, Bruce P. Department of Neurology, University of Wisconsin School of Medicine and Public Health, University of Wisconsin-Madison, Madison, Wisconsin, USA.
Dempsey, Robert J. Department of Neurological Surgery, University of Wisconsin School of Medicine and Public Health, University of Wisconsin-Madison, Madison, Wisconsin, USA.
MeSH Heading
Adult. Aged. Aged, 80 and over. Algorithms. Asymptomatic Diseases. *Carotid Stenosis/co [Complications]. *Carotid Stenosis/dg [Diagnostic Imaging]. *Cognitive Dysfunction/co [Complications]. *Cognitive Dysfunction/dg [Diagnostic Imaging]. Diagnosis, Differential. *Elasticity Imaging Techniques/mt [Methods]. Female. Humans. Male. Middle Aged. *Pattern Recognition, Automated/mt [Methods]. Reproducibility of Results. Sensitivity and Specificity.
Keyword Heading
Adventitia Carotid Plaque
Elasticity imaging
Elastography
Motion tracking
Multi-level
Strain imaging
Vascular cognitive impairment.
Year of Publication
2016

Link to the Ovid Full Text or citation:
Click here for full text options

Link to the External Link Resolver:
SFX

41.

Improved Correlation of Strain Indices with Cognitive Dysfunction with Inclusion of Adventitial Layer with Carotid Plaque.
Wang X; Mitchell CC; Varghese T; Jackson DC; Rocque BG; Hermann BP; Dempsey RJ.
Ultrasonic Imaging. 38(3):194-208, 2016 May.
[Journal Article. Research Support, N.I.H., Extramural. Research Support, Non-U.S. Gov't. Research Support, U.S. Gov't, Non-P.H.S.]
UI: 26025578
Plaque instability may lead to chronic embolization, which in turn may contribute to progressive cognitive decline. Accumulated strain tensor indices over a cardiac cycle within a pulsating carotid plaque may be viable biomarkers for the diagnosis of plaque instability. Using plaque-only carotid artery segmentations, we recently demonstrated that impaired cognitive function correlated significantly with maximum axial and lateral strain indices within a localized region of interest in plaque. Inclusion of the adventitial layer focuses our strain or instability measures on the vessel wall-plaque interface hypothesized to be a region with increased shearing forces and measureable instability. A hierarchical block-matching motion tracking algorithm developed in our laboratory was used to estimate accumulated axial, lateral, and shear strain distribution in plaques identified with the plaque-with-adventitia segmentation. Correlations of strain indices to the Repeatable Battery for the Assessment of Neuropsychological Status Total score were performed and compared with previous results. Overall, correlation coefficients (r) and significance (p) values improved for axial, lateral, and shear strain indices. Shear strain indices, however, demonstrated the largest improvement. The Pearson correlation coefficients for maximum shear strain and cognition improved from the previous plaque-only analyses of -0.432 and -0.345 to -0.795 and -0.717 with the plaque-with-adventitia segmentation for the symptomatic group and for all patients combined, respectively. Our results demonstrate the advantage of including adventitia for ultrasound carotid strain imaging providing improved association to parameters assessing cognitive impairment in patients. This supports theories of the importance of the vessel wall plaque interface in the pathophysiology of embolic disease.
Copyright © The Author(s) 2015.
Version ID
1
Record Owner
From MEDLINE, a database of the U.S. National Library of Medicine.
Status
MEDLINE
Authors Full Name
Wang, X; Mitchell, C C; Varghese, T; Jackson, D C; Rocque, B G; Hermann, B P; Dempsey, R J.
Institution

Wang, X. Department of Medical Physics, University of Wisconsin-Madison School of Medicine and Public Health, Madison, WI, USA. Mitchell, C C. Department of Medicine, University of Wisconsin-Madison, Madison School of Medicine and Public Health, WI, USA.
Varghese, T. Department of Medical Physics, University of Wisconsin-Madison School of Medicine and Public Health, Madison, WI, USA tvarghese@wisc.edu.
Jackson, D C. Department of Neurology, University of Wisconsin-Madison, Madison School of Medicine and Public Health, WI, USA.
Rocque, B G. Department of Neurological Surgery, University of Wisconsin-Madison School of Medicine and Public Health, Madison, WI, USA.
Hermann, B P. Department of Neurology, University of Wisconsin-Madison, Madison School of Medicine and Public Health, WI, USA.
Dempsey, R J. Department of Neurological Surgery, University of Wisconsin-Madison School of Medicine and Public Health, Madison, WI, USA.
MeSH Heading
Adult. *Adventitia/dg [Diagnostic Imaging]. Aged. *Carotid Stenosis/dg [Diagnostic Imaging]. *Cognitive Dysfunction/et [Etiology]. Elasticity Imaging Techniques. Female. Humans. *Image Interpretation, Computer-Assisted/mt [Methods]. Male. Middle Aged. Neuropsychological Tests.
Keyword Heading
carotid plaque elasticity imaging
motion tracking
strain
vascular cognitive dementia.
Year of Publication
2016

Link to the Ovid Full Text or citation:
Click here for full text options

Link to the External Link Resolver:
SFX

44.

Subclinical carotid artery atherosclerosis and performance on cognitive tests in middle-aged adults: Baseline results from the ELSA-Brasil.
Suemoto CK; Santos IS; Bittencourt MS; Pereira AC; Goulart AC; Rundek T; Passos VM; Lotufo P; Bensenor IM.
Atherosclerosis. 243(2):510-5, 2015 Dec.
[Journal Article. Multicenter Study. Research Support, Non-U.S. Gov't]
UI: 26520907
BACKGROUND AND AIMS: Carotid artery intima-media thickness (CIMT) may be used as a biomarker for early cognitive impairment. However, the results of the association between CIMT and cognitive function in middle-aged subjects are mixed. We aimed to investigate this association in a large Brazilian sample with no history of stroke at baseline. Additionally, we tested the effect of interactions between CIMT and cardiovascular risk factors on cognitive performance.
METHODS: In this cross-sectional study, cognition was evaluated using the delayed word recall (DWRT), the category fluency, and the trail making tests (TMT). CIMT was measured at the common carotid artery. The association between CIMT and cognitive tests was investigated using linear regression models, adjusted for an extensive set of possible confounding variables. We also included interaction terms with selected risk factors.
RESULTS: The mean age of the 8208 participants was 49.6 +/- 7.3 years, 44% were male, and 56% White. Increase in CIMT was associated with worse performance on the DWRT (beta = -0.433, 95%CI = -0.724;-0.142, p = 0.004). We found effect modification of the association between cognitive function and CMIT by self-reported heart failure and alcohol intake. Participants had worse performance in the TMT if they had greater CIMT and current alcohol use (p < 0.0001). The interaction between CIMT and heart failure on TMT performance was not significant after adjustment for multiple comparisons (p = 0.07).
CONCLUSIONS: In this sample of middle-aged adults, CIMT was inversely associated with memory function. Additionally, the presence of alcohol use resulted in a stronger association of CIMT with worse performance on an executive function test.
Copyright © 2015 Elsevier Ireland Ltd. All rights reserved.
Version ID
1
Record Owner
From MEDLINE, a database of the U.S. National Library of Medicine.
Status
MEDLINE
Authors Full Name
Suemoto, Claudia K; Santos, Itamar S; Bittencourt, Marcio S; Pereira, Alexandre C; Goulart, Alessandra C; Rundek, Tatjana; Passos, Valeria M; Lotufo, Paulo; Bensenor, Isabela M.

Institution

Suemoto, Claudia K. Division of Geriatrics, University of Sao Paulo Medical School, Sao Paulo, Brazil; Department of Global Health and Population, Harvard School of Public Health, Boston, USA. Electronic address: cksuemoto@usp.br. Santos, Itamar S. Department of Internal Medicine, University of Sao Paulo Medical School, Sao Paulo, Brazil; Center for Clinical and Epidemiological Research, Hospital Universitario, University of Sao Paulo, Sao Paulo, Brazil.

Bittencourt, Marcio S. Center for Clinical and Epidemiological Research, Hospital Universitario, University of Sao Paulo, Sao Paulo, Brazil.

Pereira, Alexandre C. Laboratory of Genetics and Molecular Cardiology, Division of Cardiology, University of Sao Paulo Medical School, Sao Paulo, Brazil.

Goulart, Alessandra C. Center for Clinical and Epidemiological Research, Hospital Universitario, University of Sao Paulo, Sao Paulo, Brazil.

Rundek, Tatjana. Departments of Neurology and Public Health Sciences, University of Miami, Miller School of Medicine, Miami, FL, USA.

Passos, Valeria M. Department of Internal Medicine, Federal University of Minas Gerais Medical School, Minas Gerais, Brazil.

Lotufo, Paulo. Department of Internal Medicine, University of Sao Paulo Medical School, Sao Paulo, Brazil; Center for Clinical and Epidemiological Research, Hospital Universitario, University of Sao Paulo, Sao Paulo, Brazil.

Bensenor, Isabela M. Department of Internal Medicine, University of Sao Paulo Medical School, Sao Paulo, Brazil; Center for Clinical and Epidemiological Research, Hospital Universitario, University of Sao Paulo, Sao Paulo, Brazil.

MeSH Heading

Adult. Alcohol Drinking/ae [Adverse Effects]. Alcohol Drinking/px [Psychology]. Asymptomatic Diseases. Brazil. *Carotid Artery Diseases/co [Complications]. Carotid Artery Diseases/dg [Diagnostic Imaging]. Carotid Artery Diseases/px [Psychology]. Carotid Artery, Common/dg [Diagnostic Imaging]. *Carotid Artery, Common. Carotid Intima-Media Thickness. *Cognition. Cognition Disorders/di [Diagnosis]. *Cognition Disorders/et [Etiology]. Cognition Disorders/px [Psychology]. Cross-Sectional Studies. Executive Function. Female. Heart Failure/co [Complications]. Heart Failure/px [Psychology]. Humans. Linear Models. Male. Mental Recall. Middle Aged. Multivariate Analysis. *Plaque, Atherosclerotic. Predictive Value of Tests. Risk Factors. Trail Making Test.

Keyword Heading

Atherosclerosis Carotid intima media thickness

Cognition disorders.

Year of Publication

2015

Link to the Ovid Full Text or citation:
Click here for full text options

Link to the External Link Resolver:
SFX

46.

Subclinical Atherosclerosis Is Inversely Associated With Gray Matter Volume in African Americans With Type 2 Diabetes.
Freedman BI; Divers J; Whitlow CT; Bowden DW; Palmer ND; Smith SC; Xu J; Register TC; Carr JJ; Wagner BC; Williamson JD; Sink KM; Maldjian JA.
Diabetes Care. 38(11):2158-65, 2015 Nov.
[Journal Article. Research Support, N.I.H., Extramural]
UI: 26370382
OBJECTIVE: Relative to European Americans, African Americans manifest lower levels of computed tomography-based calcified atherosclerotic plaque (CP), a measure of subclinical cardiovascular disease (CVD). Potential relationships between CP and cerebral structure are poorly defined in the African American population. We assessed associations among glycemic control, inflammation, and CP with cerebral structure on MRI and with cognitive performance in 268 high-risk African Americans with type 2 diabetes.
RESEARCH DESIGN AND METHODS: Associations among hemoglobin A1c (HbA1c), C-reactive protein (CRP), and CP in coronary arteries, carotid arteries, and the aorta with MRI volumetric analysis (white matter volume, gray matter volume [GMV], cerebrospinal fluid volume, and white matter lesion volume) were assessed using generalized linear models adjusted for age, sex, African ancestry proportion, smoking, BMI, use of statins, HbA1c, hypertension, and prior CVD.
RESULTS: Participants were 63.4% female with mean (SD) age of 59.8 years (9.2), diabetes duration of 14.5 years (7.6), HbA1c of 7.95% (1.9), estimated glomerular filtration rate of 86.6 mL/min/1.73 m(2) (24.6), and coronary artery CP mass score of 215 mg (502). In fully adjusted models, GMV was inversely associated with coronary artery CP (parameter estimate [beta] -0.47 [SE 0.15], P = 0.002; carotid artery CP (beta -1.92 [SE 0.62], P = 0.002; and aorta CP [beta -0.10 [SE 0.03] P = 0.002),

whereas HbA1c and CRP did not associate with cerebral volumes. Coronary artery CP also associated with poorer global cognitive function on the Montreal Cognitive Assessment. CONCLUSIONS: Subclinical atherosclerosis was associated with smaller GMV and poorer cognitive performance in African Americans with diabetes. Cardioprotective strategies could preserve GMV and cognitive function in high-risk African Americans with diabetes.

Copyright © 2015 by the American Diabetes Association. Readers may use this article as long as the work is properly cited, the use is educational and not for profit, and the work is not altered.

Version ID

1

Record Owner

From MEDLINE, a database of the U.S. National Library of Medicine.

Status

MEDLINE

Authors Full Name

Freedman, Barry I; Divers, Jasmin; Whitlow, Christopher T; Bowden, Donald W; Palmer, Nicholette D; Smith, S Carrie; Xu, Jianzhao; Register, Thomas C; Carr, J Jeffrey; Wagner, Benjamin C; Williamson, Jeff D; Sink, Kaycee M; Maldjian, Joseph A.

Institution

Freedman, Barry I. Department of Internal Medicine, Section on Nephrology, Wake Forest School of Medicine, Winston-Salem, NC Center for Diabetes Research and Center for Genomics and Personalized Medicine Research, Wake Forest School of Medicine, Winston-Salem, NC bfreedma@wakehealth.edu. Divers, Jasmin. Division of Public Health Sciences, Department of Biostatistical Sciences, Wake Forest School of Medicine, Winston-Salem, NC.

Whitlow, Christopher T. Advanced Neuroscience Imaging Research Laboratory, Department of Radiologic Sciences, Wake Forest School of Medicine, Winston-Salem, NC.

Bowden, Donald W. Center for Diabetes Research and Center for Genomics and Personalized Medicine Research, Wake Forest School of Medicine, Winston-Salem, NC Department of Biochemistry, Wake Forest School of Medicine, Winston-Salem, NC.

Palmer, Nicholette D. Center for Diabetes Research and Center for Genomics and Personalized Medicine Research, Wake Forest School of Medicine, Winston-Salem, NC Department of Biochemistry, Wake Forest School of Medicine, Winston-Salem, NC.

Smith, S Carrie. Department of Biochemistry, Wake Forest School of Medicine, Winston-Salem, NC.

Xu, Jianzhao. Center for Diabetes Research and Center for Genomics and Personalized Medicine Research, Wake Forest School of Medicine, Winston-Salem, NC.

Register, Thomas C. Department of Pathology, Wake Forest School of Medicine, Winston-Salem, NC.

Carr, J Jeffrey. Department of Radiology, Vanderbilt University School of Medicine, Nashville, TN.

Wagner, Benjamin C. Advanced Neuroscience Imaging Research Laboratory, Department of Radiologic Sciences, Wake Forest School of Medicine, Winston-Salem, NC.
Williamson, Jeff D. Section on Gerontology and Geriatric Medicine, Wake Forest School of Medicine, Department of Internal Medicine, Winston-Salem, NC.
Sink, Kaycee M. Section on Gerontology and Geriatric Medicine, Wake Forest School of Medicine, Department of Internal Medicine, Winston-Salem, NC.
Maldjian, Joseph A. Advanced Neuroscience Imaging Research Laboratory, Department of Radiologic Sciences, Wake Forest School of Medicine, Winston-Salem, NC.
MeSH Heading
*African Americans. Aged. *Atherosclerosis/co [Complications]. Atherosclerosis/di [Diagnosis]. Atherosclerosis/eh [Ethnology]. Blood Glucose/an [Analysis]. Cognition. Cognition Disorders/et [Etiology]. *Cognition Disorders/pa [Pathology]. *Diabetes Mellitus, Type 2/co [Complications]. Diabetes Mellitus, Type 2/eh [Ethnology]. Female. *Gray Matter/pa [Pathology]. Humans. Magnetic Resonance Imaging. Male. Middle Aged. Organ Size. Plaque, Atherosclerotic/dg [Diagnostic Imaging]. Plaque, Atherosclerotic/pa [Pathology]. Radiography.
Registry Number/Name of Substance
0 (Blood Glucose).
Year of Publication
2015

Link to the Ovid Full Text or citation:
Click here for full text options

Link to the External Link Resolver:
SFX

47.

Intima-Media Thickness and Cognitive Function in Stroke-Free Middle-Aged Adults: Findings From the Coronary Artery Risk Development in Young Adults Study.
Zeki Al Hazzouri A; Vittinghoff E; Sidney S; Reis JP; Jacobs DR Jr; Yaffe K.
Stroke. 46(8):2190-6, 2015 Aug.
[Journal Article. Research Support, N.I.H., Extramural. Research Support, Non-U.S. Gov't]

UI: 26106116

BACKGROUND AND PURPOSE: The relationship between carotid artery intima-media thickness (IMT) and cognitive function in midlife remains relatively unexplored. We examined the association between IMT and cognitive function in a middle-aged epidemiological cohort of 2618 stroke-free participants.

METHODS: At the year 20 visit (our study baseline), participants from the Coronary Artery Risk Development in Young Adults study had IMT measured by ultrasound at the common carotid artery. Five years later, participants completed a cognitive battery consisting of the Rey Auditory-Verbal Learning Test of verbal memory, the Digit Symbol Substitution Test of processing speed, and the Stroop test of executive function. We transformed cognitive scores into standardized z scores, with negative values indicating worse performance.

RESULTS: Mean age at baseline was 45.3 years (SD, 3.6). Greater IMT (per 1 SD difference of 0.12 mm) was significantly associated with worse performance on all cognitive tests (z scores) in unadjusted linear regression models (verbal memory, -0.16; 95% confidence interval [CI], -0.20 to -0.13; processing speed, -0.23; 95% CI, -0.27 to -0.19; and executive function, -0.17; 95% CI, -0.20 to -0.13). In models adjusted for sociodemographics and vascular risk factors that lie earlier in the causal pathway, greater IMT remained negatively associated with processing speed (-0.06; 95% CI, -0.09 to -0.02; P, 0.003) and borderline associated with executive function (-0.03; 95% CI, -0.07 to 0.00; P, 0.07) but not with verbal memory.

CONCLUSIONS: We observed an association between greater IMT and worse processing speed-a key component of cognitive functioning-at middle age above and beyond traditional vascular risk factors. Efforts targeted at preventing early stages of atherosclerosis may modify the course of cognitive aging.

Copyright © 2015 American Heart Association, Inc.

Version ID

1

Record Owner

From MEDLINE, a database of the U.S. National Library of Medicine.

Status

MEDLINE

Authors Full Name

Zeki Al Hazzouri, Adina; Vittinghoff, Eric; Sidney, Stephen; Reis, Jared P; Jacobs, David R Jr; Yaffe, Kristine.

Institution

Zeki Al Hazzouri, Adina. From the Division of Epidemiology and Population Health Sciences, Department of Public Health Sciences, University of Miami, FL (A.Z.A.H.); Departments of Epidemiology and Biostatistics (E.V., K.Y.), Psychiatry (K.Y.), and Neurology (K.Y.), University of

California San Francisco; San Francisco Veterans Affairs Medical Center, San Francisco, CA (K.Y.); Kaiser Permanente Division of Research, Oakland, CA (S.S.); Division of Cardiovascular Sciences, National Heart, Lung, and Blood Institute, National Institutes of Health, Bethesda, MD (J.P.R.); and Division of Epidemiology and Community Health, School of Public Health, University of Minnesota, Minneapolis (D.R.J.). axz122@miami.edu. Vittinghoff, Eric. From the Division of Epidemiology and Population Health Sciences, Department of Public Health Sciences, University of Miami, FL (A.Z.A.H.); Departments of Epidemiology and Biostatistics (E.V., K.Y.), Psychiatry (K.Y.), and Neurology (K.Y.), University of California San Francisco; San Francisco Veterans Affairs Medical Center, San Francisco, CA (K.Y.); Kaiser Permanente Division of Research, Oakland, CA (S.S.); Division of Cardiovascular Sciences, National Heart, Lung, and Blood Institute, National Institutes of Health, Bethesda, MD (J.P.R.); and Division of Epidemiology and Community Health, School of Public Health, University of Minnesota, Minneapolis (D.R.J.).
Sidney, Stephen. From the Division of Epidemiology and Population Health Sciences, Department of Public Health Sciences, University of Miami, FL (A.Z.A.H.); Departments of Epidemiology and Biostatistics (E.V., K.Y.), Psychiatry (K.Y.), and Neurology (K.Y.), University of California San Francisco; San Francisco Veterans Affairs Medical Center, San Francisco, CA (K.Y.); Kaiser Permanente Division of Research, Oakland, CA (S.S.); Division of Cardiovascular Sciences, National Heart, Lung, and Blood Institute, National Institutes of Health, Bethesda, MD (J.P.R.); and Division of Epidemiology and Community Health, School of Public Health, University of Minnesota, Minneapolis (D.R.J.).
Reis, Jared P. From the Division of Epidemiology and Population Health Sciences, Department of Public Health Sciences, University of Miami, FL (A.Z.A.H.); Departments of Epidemiology and Biostatistics (E.V., K.Y.), Psychiatry (K.Y.), and Neurology (K.Y.), University of California San Francisco; San Francisco Veterans Affairs Medical Center, San Francisco, CA (K.Y.); Kaiser Permanente Division of Research, Oakland, CA (S.S.); Division of Cardiovascular Sciences, National Heart, Lung, and Blood Institute, National Institutes of Health, Bethesda, MD (J.P.R.); and Division of Epidemiology and Community Health, School of Public Health, University of Minnesota, Minneapolis (D.R.J.).
Jacobs, David R Jr. From the Division of Epidemiology and Population Health Sciences, Department of Public Health Sciences, University of Miami, FL (A.Z.A.H.); Departments of Epidemiology and Biostatistics (E.V., K.Y.), Psychiatry (K.Y.), and Neurology (K.Y.), University of California San Francisco; San Francisco Veterans Affairs Medical Center, San Francisco, CA (K.Y.); Kaiser Permanente Division of Research, Oakland, CA (S.S.); Division of Cardiovascular Sciences, National Heart, Lung, and Blood Institute, National Institutes of Health, Bethesda, MD (J.P.R.); and Division of Epidemiology and Community Health, School of Public Health, University of Minnesota, Minneapolis (D.R.J.).
Yaffe, Kristine. From the Division of Epidemiology and Population Health Sciences, Department of Public Health Sciences, University of Miami, FL (A.Z.A.H.); Departments of Epidemiology and Biostatistics (E.V., K.Y.), Psychiatry (K.Y.), and Neurology (K.Y.), University of California San Francisco; San Francisco Veterans Affairs Medical Center, San Francisco, CA (K.Y.); Kaiser Permanente Division

of Research, Oakland, CA (S.S.); Division of Cardiovascular Sciences, National Heart, Lung, and Blood Institute, National Institutes of Health, Bethesda, MD (J.P.R.); and Division of Epidemiology and Community Health, School of Public Health, University of Minnesota, Minneapolis (D.R.J.).

MeSH Heading

Adult. *Carotid Artery Diseases/dg [Diagnostic Imaging]. Carotid Artery Diseases/px [Psychology]. *Carotid Artery, Common/dg [Diagnostic Imaging]. Carotid Intima-Media Thickness/px [Psychology]. *Carotid Intima-Media Thickness. *Cognition Disorders/dg [Diagnostic Imaging]. Cognition Disorders/px [Psychology]. Cohort Studies. *Coronary Artery Disease/dg [Diagnostic Imaging]. Coronary Artery Disease/px [Psychology]. Female. Humans. Male. Middle Aged. Prospective Studies. Risk Factors. *Stroke/dg [Diagnostic Imaging]. Stroke/px [Psychology]. Young Adult.

Keyword Heading

carotid intima-media thickness cognition

epidemiology

risk factors

stroke.

Year of Publication

2015

Link to the Ovid Full Text or citation:

[Click here for full text options]

Link to the External Link Resolver:

[SFX]

48.

Carotid and vertebral arterial variations in Alzheimer's disease.

Zhou R; Liu D; Yu K; Chen Y; Li L; Xu J; Zhou H.

Current Alzheimer Research. 12(4):368-76, 2015.

[Journal Article]

UI: 25817257

The effects of carotid and vertebral arterial morphological variations on cognitive function impairment remain unclear. We investigated the association between extracranial carotid and vertebral arterial variations and the risk of Alzheimer's disease (AD). A prospective study with a 5-year followup was conducted from July 2008 to June 2013. A total of 1741 subjects (50 years of age and older) were examined for carotid and vertebral arterial variations using computed tomography angiography (CTA) and completed the study follow-up. Variations of the carotid and vertebral arteries were classified as tortuosity, kinking and coiling, according to the Weibel and Fields criteria. Cognitive function was assessed using the Mini-Mental State Examination and the Activities of Daily Living scale. We analyzed the association between arterial variations and the risk of AD by using multivariate Cox proportional-hazards models. The prevalence of carotid arterial variations was 38.4%, and the prevalence of vertebral arterial variations was 86.6%. Among the 1741 subjects who completed the study follow-up, 134 AD cases were detected. The subjects diagnosed with AD displayed greater kinking and coiling in the carotid artery (P<0.01) and vertebral artery (P<0.05) than the subjects without AD. After adjusting for potential confounders, kinking and coiling (hazard ratio [HR]=1.93, 95% confidence interval [CI], 1.37 to 2.86, P<0.01) in the carotid artery were significantly associated with AD. Additionally, after adjusting for potential confounders, kinking and coiling (HR=1.73, 95% CI, 1.25 to 2.31, P<0.01) in the vertebral artery were significantly associated with the risk of AD. We determined that age, hypertension and smoking status were significant predictors of AD in the multivariable models with carotid and vertebral arterial variation. The results of the current study indicate that severe carotid and vertebral arterial variations are associated with a significantly increased risk of AD. Further investigation into the association between these variations and AD would be useful for preventing AD.
Version ID
1
Record Owner
From MEDLINE, a database of the U.S. National Library of Medicine.
Status
MEDLINE
Authors Full Name
Zhou, Rui; Liu, Dong; Yu, Ke; Chen, Yang; Li, Ling; Xu, Jianzhong; Zhou, Huadong.
Institution
Zhou, Huadong. Department of Neurology, Daping hospital, Third Military Medical University, Chongqing, 400038, P.R. China. zhouhuad@163.com.
MeSH Heading
Aged. Aged, 80 and over. Alzheimer Disease/ep [Epidemiology]. *Alzheimer Disease/pa [Pathology]. Angiography. *Carotid Arteries/pa [Pathology]. Carotid Intima-Media Thickness. Disease-Free Survival. Female. Follow-Up Studies. Humans. Incidence. Male. Middle Aged.

Multivariate Analysis. Neuropsychological Tests. Proportional Hazards Models. Sex Characteristics. Tomography, X-Ray Computed. *Vertebral Artery/pa [Pathology].
Year of Publication
2015

Link to the Ovid Full Text or citation:
[Click here for full text options]

Link to the External Link Resolver:
[SFX]

50.

Markers for the risk of progression from mild cognitive impairment to Alzheimer's disease.
Buratti L; Balestrini S; Altamura C; Viticchi G; Falsetti L; Luzzi S; Provinciali L; Vernieri F; Silvestrini M.
Journal of Alzheimer's Disease. 45(3):883-90, 2015.
[Journal Article]
UI: 25633680
BACKGROUND: Defining reliable markers of conversion to dementia could be the first step in order to identify appropriate treatment strategies for mild cognitive impairment (MCI) patients.
OBJECTIVE: To develop a tool able to predict the risk of progression from MCI to Alzheimer's disease (AD).
METHODS: 406 MCI patients were included and followed for a one-year period. Demographic characteristics, vascular risk factors, extent of cerebrovascular lesions, markers of carotid atherosclerosis investigated with an ultrasonographic assessment (plaque index and intima-media thickness) and cerebrovascular reactivity to apnea (breath-holding index) were considered as potential predictors of conversion.
RESULTS: 106 (26%) MCI patients showed a conversion to AD. Plaque index, intima-media thickness, and breath-holding index were relevant predictors of conversion ($p = 0.042$; $p = 0.003$; $p < 0.001$, multivariate logistic regression analysis). A simplified scoring system was devised based on the magnitude of the estimated multinomial logistic regression beta coefficient results. A total score was calculated as the sum of each predictive factor which resulted in a 0-5 range. The optimal cut-off score was >=3 (sensitivity, 23.6%, 95% CI 15.9%-32.8%; specificity, 97.7%, 95% CI

95.3%-99.1%; positive likelihood ratio, 10.1, 95% CI 4.5%-22.7%; negative likelihood ratio, 0.78, 95% CI 0.70%-0.87%). The AUC was 0.71 (95% CI, 0.65-0.77).
CONCLUSIONS: Our findings show the possibility to obtain a predictive indicator of the risk of conversion from MCI to dementia by considering the presence of both atherosclerotic changes in the carotid district and impairment of cerebral hemodynamics. Such an approach may allow us to formulate a correct prognosis in more than 70% of patients with amnesic MCI.
Version ID
1
Record Owner
From MEDLINE, a database of the U.S. National Library of Medicine.
Status
MEDLINE
Authors Full Name
Buratti, Laura; Balestrini, Simona; Altamura, Claudia; Viticchi, Giovanna; Falsetti, Lorenzo; Luzzi, Simona; Provinciali, Leandro; Vernieri, Fabrizio; Silvestrini, Mauro.
Institution
Buratti, Laura. Neurological Clinic, Marche Polytechnic University, Ancona, Italy. Balestrini, Simona. Neurological Clinic, Marche Polytechnic University, Ancona, Italy.
Altamura, Claudia. Neurology Unit, Campus Bio-Medico University, Rome, Italy.
Viticchi, Giovanna. Neurological Clinic, Marche Polytechnic University, Ancona, Italy.
Falsetti, Lorenzo. Internal and Subintensive Medicine, Ospedali Riuniti Ancona, Italy.
Luzzi, Simona. Neurological Clinic, Marche Polytechnic University, Ancona, Italy.
Provinciali, Leandro. Neurological Clinic, Marche Polytechnic University, Ancona, Italy.
Vernieri, Fabrizio. Neurology Unit, Campus Bio-Medico University, Rome, Italy.
Silvestrini, Mauro. Neurological Clinic, Marche Polytechnic University, Ancona, Italy.
MeSH Heading
Aged. Alzheimer Disease/co [Complications]. Alzheimer Disease/ep [Epidemiology]. Alzheimer Disease/px [Psychology]. *Alzheimer Disease. Biomarkers. Carotid Intima-Media Thickness. Cerebrovascular Disorders. Cognition Disorders/ep [Epidemiology]. *Cognition Disorders/et [Etiology]. Cognition Disorders/px [Psychology]. Disease Progression. Female. Humans. Logistic Models. Longitudinal Studies. Male. Neuropsychological Tests. ROC Curve. Risk Factors.
Keyword Heading
Alzheimer's disease atherosclerosis
carotid arteries
cerebral hemodynamics
mild cognitive impairment
ultrasonography.

Registry Number/Name of Substance
0 (Biomarkers).
Year of Publication
2015

Link to the Ovid Full Text or citation:
Click here for full text options

Link to the External Link Resolver:
SFX

51.

Association of postural instability with asymptomatic cerebrovascular damage and cognitive decline: the Japan Shimanami health promoting program study.
Tabara Y; Okada Y; Ohara M; Uetani E; Kido T; Ochi N; Nagai T; Igase M; Miki T; Matsuda F; Kohara K.
Stroke. 46(1):16-22, 2015 Jan.
[Journal Article. Research Support, Non-U.S. Gov't]
UI: 25523051
BACKGROUND AND PURPOSE: Asymptomatic cerebral small-vessel disease (cSVD) in elderly individuals are potent risk factors for stroke. In addition to common clinical risk factors, postural instability has been postulated to be associated with cSVD in older frail patients. Here, we conducted a cross-sectional study to understand the possible link between postural instability and asymptomatic cSVD further, namely periventricular hyperintensity, lacunar infarction, and microbleeds, as well as cognitive function, in a middle-aged to elderly general population (n=1387).
METHODS: Postural instability was assessed based on one-leg standing time (OLST) and posturography findings. cSVD was evaluated by brain MRI. Mild cognitive impairment was assessed using a computer-based questionnaire, and carotid intima-media thickness as an index of atherosclerosis was measured via ultrasonography.
RESULTS: Frequency of short OLST, in particular <20 s, increased linearly with severity of cSVD (lacunar infarction lesion: none, 9.7%; 1, 16.0%; >2, 34.5%; microbleeds lesion: none, 10.1%; 1, 15.3%;

>2, 30.0%; periventricular hyperintensity grade: 0, 5.7%; 1, 11.5%; >2, 23.7%). The association of short OLST with lacunar infarction and microbleeds but not periventricular hyperintensity remained significant even after adjustment for possible covariates (lacunar infarction, P=0.009; microbleeds, P=0.003; periventricular hyperintensity, P=0.601). In contrast, no significant association was found between posturographic parameters and cSVD, whereas these parameters were linearly associated with OLST. Short OLST was also significantly associated with reduced cognitive function independent of covariates, including cSVD (P=0.002).
CONCLUSIONS: Postural instability was found to be associated with early pathological changes in the brain and functional decline, even in apparently healthy subjects.
Copyright © 2014 American Heart Association, Inc.
Version ID
1
Record Owner
From MEDLINE, a database of the U.S. National Library of Medicine.
Status
MEDLINE
Authors Full Name
Tabara, Yasuharu; Okada, Yoko; Ohara, Maya; Uetani, Eri; Kido, Tomoko; Ochi, Namiko; Nagai, Tokihisa; Igase, Michiya; Miki, Tetsuro; Matsuda, Fumihiko; Kohara, Katsuhiko.
Institution
Tabara, Yasuharu. From the Center for Genomic Medicine, Kyoto University Graduate School of Medicine, Sakyo-ku, Kyoto, Japan (Y.T., T.M., F.M.); and Department of Geriatric Medicine, Ehime University Graduate School of Medicine, Toon City, Ehime, Japan (Y.T., Y.O., M.O., E.U., T.K., N.O., T.N., M.I., T.M., K.K.). tabara@genome.med.kyoto-u.ac.jp. Okada, Yoko. From the Center for Genomic Medicine, Kyoto University Graduate School of Medicine, Sakyo-ku, Kyoto, Japan (Y.T., T.M., F.M.); and Department of Geriatric Medicine, Ehime University Graduate School of Medicine, Toon City, Ehime, Japan (Y.T., Y.O., M.O., E.U., T.K., N.O., T.N., M.I., T.M., K.K.).
Ohara, Maya. From the Center for Genomic Medicine, Kyoto University Graduate School of Medicine, Sakyo-ku, Kyoto, Japan (Y.T., T.M., F.M.); and Department of Geriatric Medicine, Ehime University Graduate School of Medicine, Toon City, Ehime, Japan (Y.T., Y.O., M.O., E.U., T.K., N.O., T.N., M.I., T.M., K.K.).
Uetani, Eri. From the Center for Genomic Medicine, Kyoto University Graduate School of Medicine, Sakyo-ku, Kyoto, Japan (Y.T., T.M., F.M.); and Department of Geriatric Medicine, Ehime University Graduate School of Medicine, Toon City, Ehime, Japan (Y.T., Y.O., M.O., E.U., T.K., N.O., T.N., M.I., T.M., K.K.).
Kido, Tomoko. From the Center for Genomic Medicine, Kyoto University Graduate School of Medicine, Sakyo-ku, Kyoto, Japan (Y.T., T.M., F.M.); and Department of Geriatric Medicine, Ehime

University Graduate School of Medicine, Toon City, Ehime, Japan (Y.T., Y.O., M.O., E.U., T.K., N.O., T.N., M.I., T.M., K.K.).
Ochi, Namiko. From the Center for Genomic Medicine, Kyoto University Graduate School of Medicine, Sakyo-ku, Kyoto, Japan (Y.T., T.M., F.M.); and Department of Geriatric Medicine, Ehime University Graduate School of Medicine, Toon City, Ehime, Japan (Y.T., Y.O., M.O., E.U., T.K., N.O., T.N., M.I., T.M., K.K.).
Nagai, Tokihisa. From the Center for Genomic Medicine, Kyoto University Graduate School of Medicine, Sakyo-ku, Kyoto, Japan (Y.T., T.M., F.M.); and Department of Geriatric Medicine, Ehime University Graduate School of Medicine, Toon City, Ehime, Japan (Y.T., Y.O., M.O., E.U., T.K., N.O., T.N., M.I., T.M., K.K.).
Igase, Michiya. From the Center for Genomic Medicine, Kyoto University Graduate School of Medicine, Sakyo-ku, Kyoto, Japan (Y.T., T.M., F.M.); and Department of Geriatric Medicine, Ehime University Graduate School of Medicine, Toon City, Ehime, Japan (Y.T., Y.O., M.O., E.U., T.K., N.O., T.N., M.I., T.M., K.K.).
Miki, Tetsuro. From the Center for Genomic Medicine, Kyoto University Graduate School of Medicine, Sakyo-ku, Kyoto, Japan (Y.T., T.M., F.M.); and Department of Geriatric Medicine, Ehime University Graduate School of Medicine, Toon City, Ehime, Japan (Y.T., Y.O., M.O., E.U., T.K., N.O., T.N., M.I., T.M., K.K.).
Matsuda, Fumihiko. From the Center for Genomic Medicine, Kyoto University Graduate School of Medicine, Sakyo-ku, Kyoto, Japan (Y.T., T.M., F.M.); and Department of Geriatric Medicine, Ehime University Graduate School of Medicine, Toon City, Ehime, Japan (Y.T., Y.O., M.O., E.U., T.K., N.O., T.N., M.I., T.M., K.K.).
Kohara, Katsuhiko. From the Center for Genomic Medicine, Kyoto University Graduate School of Medicine, Sakyo-ku, Kyoto, Japan (Y.T., T.M., F.M.); and Department of Geriatric Medicine, Ehime University Graduate School of Medicine, Toon City, Ehime, Japan (Y.T., Y.O., M.O., E.U., T.K., N.O., T.N., M.I., T.M., K.K.).
Comments
Comment in (CIN) Erratum in (EIN)
MeSH Heading
Aged. Asymptomatic Diseases. *Brain/pa [Pathology]. Carotid Artery Diseases/dg [Diagnostic Imaging]. *Carotid Artery Diseases/ep [Epidemiology]. Carotid Intima-Media Thickness. *Cerebral Hemorrhage/ep [Epidemiology]. Cerebral Hemorrhage/pa [Pathology]. Cerebral Small Vessel Diseases/ep [Epidemiology]. Cerebral Small Vessel Diseases/pa [Pathology]. Cerebrovascular Disorders/ep [Epidemiology]. Cerebrovascular Disorders/pa [Pathology]. Cognition Disorders/ep [Epidemiology]. Cognition Disorders/pa [Pathology]. *Cognitive Dysfunction/ep [Epidemiology]. Cognitive Dysfunction/pa [Pathology]. Cross-Sectional Studies. Female. Humans. Japan/ep

[Epidemiology]. Magnetic Resonance Imaging. Male. Middle Aged. *Postural Balance. *Sensation Disorders/ep [Epidemiology]. *Stroke, Lacunar/ep [Epidemiology]. Stroke, Lacunar/pa [Pathology].
Keyword Heading
lacunar stroke.
Year of Publication
2015

Link to the Ovid Full Text or citation:
Click here for full text options

Link to the External Link Resolver:
SFX

52.

Carotid plaque as a predictor of dementia in older adults: the Three-City Study.
Carcaillon L; Plichart M; Zureik M; Rouaud O; Majed B; Ritchie K; Tzourio C; Dartigues JF; Empana JP.
Alzheimer's & Dementia. 11(3):239-48, 2015 Mar.
[Journal Article. Multicenter Study. Research Support, Non-U.S. Gov't]
UI: 25510384
BACKGROUND: The contribution of carotid atherosclerosis to incident dementia remains unclear. We examined the association between carotid plaques (CP) and common carotid intima media thickness (CCA-IMT) with incident dementia and its subtypes, and their added value for dementia risk prediction.
METHODS: At baseline, 6025 dementia-free subjects aged 65-86 years underwent bilateral carotid ultrasonography measures of CP and plaque-free CCA-IMT. Subjects were followed-up over 7 years for the detection of dementia.
RESULTS: After a mean 5.4 years of follow-up, 421 subjects developed dementia including 272 Alzheimer's disease and 83 vascular/mixed dementia (VaD). Only CP were independently related to VaD (HR(>=2 sites with plaques) = 1.92; 95% confidence interval or CI = 1.13-3.22) and improved VaD risk prediction (continuous Net Reclassification Index = 30.1%; 95% CI = 8.4-51.7) beyond

known dementia risk factors. Accounting for stroke or competing risk by death marginally modified the results.
CONCLUSION: In older adults, CP are independent predictors of incident VaD and may improve VaD risk prediction.
Copyright © 2015 The Alzheimer's Association. Published by Elsevier Inc. All rights reserved.
Version ID
1
Record Owner
From MEDLINE, a database of the U.S. National Library of Medicine.
Status
MEDLINE
Authors Full Name
Carcaillon, Laure; Plichart, Matthieu; Zureik, Mahmoud; Rouaud, Olivier; Majed, Bilal; Ritchie, Karen; Tzourio, Christophe; Dartigues, Jean-Francois; Empana, Jean-Philippe.
Institution
Carcaillon, Laure. Inserm, CESP Centre for Research in Epidemiology and Population Health, UMR-S1018, Hormones and Cardiovascular Disease, University Paris Sud, Villejuif, France. Plichart, Matthieu. Inserm, UMR-S 970, Paris Descartes University, Sorbonne Paris Cite, Paris Cardiovascular Research Center, Paris, France; Assistance Publique - Hopitaux de Paris, Hopital Broca, Paris, France. Electronic address: matthieu.plichart@inserm.fr.
Zureik, Mahmoud. Inserm U700, Paris, France.
Rouaud, Olivier. CMMR CHU Dijon, Dijon, France.
Majed, Bilal. Inserm, UMR-S 970, Paris Descartes University, Sorbonne Paris Cite, Paris Cardiovascular Research Center, Paris, France; Epidemiology and Clinical Research Unit, Arras General Hospital, Arras, France.
Ritchie, Karen. Inserm U1061, Neuropsychiatry: Epidemiological and Clinical Research, Hopital La Colombiere, Montpellier, France; Imperial College, Faculty of Medicine, London, United Kingdom; University of Montpellier 1, Faculty of Medicine, Montpellier, France.
Tzourio, Christophe. Inserm U708, Neuroepidemiology, Bordeaux, France; University of Victor Segalen Bordeaux2, Bordeaux, France.
Dartigues, Jean-Francois. University of Victor Segalen Bordeaux2, Bordeaux, France; Inserm U897, Epidemiology and Neuropsychology of Brain Aging, Bordeaux, France.
Empana, Jean-Philippe. Inserm, UMR-S 970, Paris Descartes University, Sorbonne Paris Cite, Paris Cardiovascular Research Center, Paris, France.
MeSH Heading
Aged. Aged, 80 and over. *Carotid Arteries/dg [Diagnostic Imaging]. *Carotid Artery Diseases/dg [Diagnostic Imaging]. Carotid Artery Diseases/ep [Epidemiology]. Carotid Intima-Media Thickness.

*Dementia/di [Diagnosis]. *Dementia/dg [Diagnostic Imaging]. Dementia/ep [Epidemiology]. Female. Follow-Up Studies. Humans. Incidence. Male. *Plaque, Atherosclerotic/dg [Diagnostic Imaging]. Plaque, Atherosclerotic/ep [Epidemiology]. Prognosis. Prospective Studies. Risk. Risk Factors. Sensitivity and Specificity.

Keyword Heading

Aging Atherosclerosis

Dementia

Epidemiology

Risk factors.

Year of Publication

2015

Link to the Ovid Full Text or citation:

Click here for full text options

Link to the External Link Resolver:

SFX

53.

Atherosclerotic calcification is related to a higher risk of dementia and cognitive decline.

Bos D; Vernooij MW; de Bruijn RF; Koudstaal PJ; Hofman A; Franco OH; van der Lugt A; Ikram MA.

Alzheimer's & Dementia. 11(6):639-47.e1, 2015 Jun.

[Journal Article. Research Support, Non-U.S. Gov't]

UI: 25150731

BACKGROUND: Longitudinal data on the role of atherosclerosis in different vessel beds in the etiology of cognitive impairment and dementia are scarce and inconsistent.

METHODS: Between 2003-2006, 2364 nondemented persons underwent computed tomography of the coronaries, aortic arch, extracranial, and intracranial carotid arteries to quantify atherosclerotic calcification. Participants were followed for incident dementia (n = 90) until April 2012. At baseline and follow-up participants also underwent a cognitive test battery.

RESULTS: Larger calcification volume in all vessels, except in the coronaries, was associated with a higher risk of dementia. After adjustment for relevant confounders, extracranial carotid artery

calcification remained significantly associated with a higher risk of dementia [hazard ratio per standard deviation increase in calcification volume: 1.37 (1.05, 1.79)]. Additional analyses for Alzheimer's disease only or censoring for stroke showed similar results. Larger calcification volumes were also associated with cognitive decline.
CONCLUSIONS: Atherosclerosis, in particular in the extracranial carotid arteries, is related to a higher risk of dementia and cognitive decline.
Copyright © 2015 The Alzheimer's Association. Published by Elsevier Inc. All rights reserved.
Version ID
1
Record Owner
From MEDLINE, a database of the U.S. National Library of Medicine.
Status
MEDLINE
Authors Full Name
Bos, Daniel; Vernooij, Meike W; de Bruijn, Renee F A G; Koudstaal, Peter J; Hofman, Albert; Franco, Oscar H; van der Lugt, Aad; Ikram, M Arfan.
Institution
Bos, Daniel. Department of Radiology, Erasmus Medical Center, Rotterdam, The Netherlands; Department of Epidemiology, Erasmus Medical Center, Rotterdam, The Netherlands. Vernooij, Meike W. Department of Radiology, Erasmus Medical Center, Rotterdam, The Netherlands; Department of Epidemiology, Erasmus Medical Center, Rotterdam, The Netherlands.
de Bruijn, Renee F A G. Department of Epidemiology, Erasmus Medical Center, Rotterdam, The Netherlands; Department of Neurology, Erasmus Medical Center, Rotterdam, The Netherlands.
Koudstaal, Peter J. Department of Neurology, Erasmus Medical Center, Rotterdam, The Netherlands.
Hofman, Albert. Department of Epidemiology, Erasmus Medical Center, Rotterdam, The Netherlands.
Franco, Oscar H. Department of Epidemiology, Erasmus Medical Center, Rotterdam, The Netherlands.
van der Lugt, Aad. Department of Radiology, Erasmus Medical Center, Rotterdam, The Netherlands.
Ikram, M Arfan. Department of Radiology, Erasmus Medical Center, Rotterdam, The Netherlands; Department of Epidemiology, Erasmus Medical Center, Rotterdam, The Netherlands; Department of Neurology, Erasmus Medical Center, Rotterdam, The Netherlands. Electronic address: m.a.ikram@erasmusmc.nl.
MeSH Heading

Aged. Aorta, Thoracic/dg [Diagnostic Imaging]. Atherosclerosis/dg [Diagnostic Imaging]. *Atherosclerosis/ep [Epidemiology]. Atherosclerosis/px [Psychology]. Calcinosis/dg [Diagnostic Imaging]. *Calcinosis/ep [Epidemiology]. Calcinosis/px [Psychology]. Carotid Arteries/dg [Diagnostic Imaging]. Cognition Disorders/dg [Diagnostic Imaging]. *Cognition Disorders/ep [Epidemiology]. Coronary Vessels/dg [Diagnostic Imaging]. Dementia/dg [Diagnostic Imaging]. *Dementia/ep [Epidemiology]. Female. Follow-Up Studies. Humans. Longitudinal Studies. Male. Netherlands/ep [Epidemiology]. Prospective Studies. Risk. Stroke/dg [Diagnostic Imaging]. Stroke/ep [Epidemiology]. Stroke/px [Psychology]. Tomography, X-Ray Computed.

Keyword Heading

Arterial calcification Atherosclerosis

Cognitive decline

Dementia

Epidemiology

Imaging.

Year of Publication

2015

Link to the Ovid Full Text or citation:

Click here for full text options

Link to the External Link Resolver:

SFX

55.

The role of carotid intima-media thickness in predicting longitudinal cognitive function in an older adult cohort.

Frazier DT; Seider T; Bettcher BM; Mack WJ; Jastrzab L; Chao L; Weiner MW; DeCarli C; Reed BR; Mungas D; Chui HC; Kramer JH.

Cerebrovascular Diseases. 38(6):441-7, 2014.

[Journal Article. Research Support, N.I.H., Extramural]

UI: 25502351

BACKGROUND AND PURPOSE: Carotid atherosclerosis is a risk factor for cerebrovascular disease in older adults. Although age-related cognitive decline has been associated with cerebrovascular disease, not much is known about the consequences of carotid atherosclerosis on longitudinal cognitive function. This study examines the longitudinal relationship between atherosclerosis and cognition in a sample of non-demented older subjects using baseline measurements of carotid intima media thickness (CIMT) and annual cognitive measures of executive function (EXEC) and verbal memory (MEM).
METHODS: Baseline measurements included CIMT derived from B-mode carotid artery ultrasound, structural T1-weighted images of white matter hypointensities (WMH), white matter lesions (WML), and cerebral infarct. Hypertension, low-density lipoprotein (LDL), diabetes, and waist to hip ratios (WHR) were included as covariates in our models to control for cerebrovascular risks and central adiposity. Annual composite scores of EXEC and MEM functions were derived from item response theory. Linear mixed models were used to model longitudinal cognitive change.
RESULTS: A significant inverse relationship was found between baseline CIMT and annual EXEC score, but not annual MEM score. Subjects included in the highest 4th quartile of CIMT showed a rate of annual decline in EXEC score that was significant relative to subjects in lower quartile groups ($p<0.01$). The relationship between the 4th quartile of CIMT and annual EXEC score remained significant after independently adjusting for imaging measures of white matter injury and cerebral infarct.
CONCLUSIONS: Older adult subjects with the highest index of CIMT showed an annual decline in EXEC scores that was significant relative to subjects with lower quartile measurements of CIMT, independent of our measures of white matter injury and cerebral infarct. Our findings suggest that elevated measures of CIMT may mark an atherosclerotic state, resulting in a decline in executive function and not memory in non-demented older adults.
Copyright © 2014 S. Karger AG, Basel.
Version ID
1
Record Owner
From MEDLINE, a database of the U.S. National Library of Medicine.
Status
MEDLINE
Authors Full Name
Frazier, Darvis T; Seider, Talia; Bettcher, Brianne M; Mack, Wendy J; Jastrzab, Laura; Chao, Linda; Weiner, Michael W; DeCarli, Charles; Reed, Bruce R; Mungas, Dan; Chui, Helena C; Kramer, Joel H.
Institution
Frazier, Darvis T. Memory and Aging Center, San Francisco, Calif., USA.
MeSH Heading

Aged. Aged, 80 and over. Brain/pa [Pathology]. Carotid Artery Diseases/dg [Diagnostic Imaging]. *Carotid Artery Diseases/ep [Epidemiology]. *Carotid Intima-Media Thickness. *Cognition. *Cognition Disorders/ep [Epidemiology]. Cognition Disorders/pa [Pathology]. Executive Function. Female. Humans. Longitudinal Studies. Magnetic Resonance Imaging. Male. Memory. Middle Aged. Neuropsychological Tests. Prospective Studies. *White Matter/pa [Pathology].
Year of Publication
2014

Link to the Ovid Full Text or citation:
Click here for full text options

Link to the External Link Resolver:
SFX

59.

Differential impact of subclinical carotid artery disease on cerebral structure and functioning in type 1 diabetes patients with versus those without proliferative retinopathy.
van Duinkerken E; Ijzerman RG; van der Zijl NJ; Barkhof F; Pouwels PJ; Schoonheim MM; Moll AC; Boerop J; Wessels AM; Klein M; Snoek FJ; Diamant M.
Cardiovascular Diabetology. 13:58, 2014 Mar 12.
[Comparative Study. Journal Article. Research Support, Non-U.S. Gov't]
UI: 24620788
BACKGROUND: Type 1 diabetes mellitus (T1DM) is associated with cerebral compromise, typically found in patients with microangiopathy. Associations between subclinical macroangiopathy and the brain, whether or not in the presence of microangiopathy, have not been fully explored in T1DM. We hypothesized that subclinical macroangiopathy in adult T1DM may affect the brain and interacts with microangiopathy.
METHODS: In 51 asymptomatic T1DM patients with, 53 without proliferative retinopathy and 51 controls, right common carotid artery ultrasound was used to assess intima media thickness (cIMT) and distensibility (cD). Neuropsychological tests for cognitive functions, and magnetic resonance imagining for white matter integrity and functional connectivity, i.e. neuronal communication, were used.

RESULTS: After correction for confounders, cIMT was borderline significantly increased in all T1DM patients (P = 0.071), whereas cD was not statistically significantly altered (P = 0.45). Patients with proliferative retinopathy showed the largest increase in cIMT and decrease in cD. In all participants, after adjustment for confounders, increased cIMT was related to decreased white matter integrity (beta = -0.198 P = 0.041) and decreased functional connectivity in visual areas (beta = -0.195 P = 0.046). For cognition, there was a significant interaction between cIMT and the presence of proliferative retinopathy after adjustment for confounding factors (all P < 0.05). Increased cIMT was associated with lower general cognitive ability (beta = -0.334; P = 0.018), information processing speed (beta = -0.361; P = 0.010) and attention (beta = -0.394; P = 0.005) scores in patients without, but not in patients with proliferative retinopathy.
CONCLUSIONS: These findings suggest that subclinical macroangiopathy may be a factor in the development of diabetes-related cognitive changes in uncomplicated T1DM, whereas in patients with advanced T1DM, proliferative retinopathy may rather be the driving force of cerebral compromise.
Version ID
1
Record Owner
From MEDLINE, a database of the U.S. National Library of Medicine.
Status
MEDLINE
Authors Full Name
van Duinkerken, Eelco; Ijzerman, Richard G; van der Zijl, Nynke J; Barkhof, Frederik; Pouwels, Petra J W; Schoonheim, Menno M; Moll, Annette C; Boerop, Jeannette; Wessels, Alette M; Klein, Martin; Snoek, Frank J; Diamant, Michaela.
Institution
van Duinkerken, Eelco. Diabetes Center/Department of Internal Medicine, VU University Medical Center, De Boelelaan 1117 - Room MF-G417, 1081 HV, Amsterdam, The Netherlands. e.vanduinkerken@vumc.nl.
MeSH Heading
Adolescent. Adult. *Carotid Artery Diseases/dg [Diagnostic Imaging]. Carotid Artery Diseases/ep [Epidemiology]. Carotid Artery Diseases/pp [Physiopathology]. *Carotid Intima-Media Thickness. Cell Proliferation. *Cerebrovascular Circulation/ph [Physiology]. Cross-Sectional Studies. *Diabetes Mellitus, Type 1/dg [Diagnostic Imaging]. Diabetes Mellitus, Type 1/ep [Epidemiology]. Diabetes Mellitus, Type 1/pp [Physiopathology]. *Diabetic Retinopathy/dg [Diagnostic Imaging]. Diabetic Retinopathy/ep [Epidemiology]. Diabetic Retinopathy/pp [Physiopathology]. Female. Humans. Male. Middle Aged. Single-Blind Method. Young Adult.
Year of Publication

2014

Link to the Ovid Full Text or citation:
[Click here for full text options]

Link to the External Link Resolver:
[SFX]

60.

Visit-to-visit blood pressure variability in the elderly: associations with cognitive impairment and carotid artery remodeling.
Nagai M; Hoshide S; Nishikawa M; Masahisa S; Kario K.
Atherosclerosis. 233(1):19-26, 2014 Mar.
[Journal Article]
UI: 24529116
OBJECTIVE: Recently, visit-to-visit blood pressure (BP) variability has been shown to be associated with vascular remodeling and cognitive dysfunction. However, there have been no studies that focused on the relationship between visit-to-visit BP variability and cognitive dysfunction in relation to vascular remodeling. In this study, we investigated the relationships among visit-to-visit BP measures, carotid artery remodeling and cognitive function in the elderly at high risk of cardiovascular disease.
METHODS: The cognitive function was evaluated using a Mini-Mental State Examination (MMSE) and global deterioration scale (GDS) in 201 elderly subjects at high risk of cardiovascular disease (79.9 +/- 6.4 years old; female 75%). Based on 12 visits (once a month), visit-to-visit BP variability (expressed as the coefficient of variation [CV] and as delta [maximum--minimum] BP) were measured. Carotid ultrasound was performed to measure intima-media thickness (IMT) and the stiffness parameter beta.
RESULTS: The patients having both high delta systolic BP (SBP) and high IMT had significantly higher prevalence of low MMSE score than those with both low delta SBP and low IMT ($p < 0.05$), and the patients having both high delta SBP and high stiffness parameter beta also had significantly higher prevalence of low MMSE score than those with both low delta SBP and low stiffness parameter beta ($p < 0.01$). In the logistic regression analysis adjusted for age, calcium

channel blocker use, low density lipoprotein, average heart rate, and average SBP level, a significant interaction was found between delta SBP and stiffness parameter beta for the low MMSE score ($p < 0.05$).
CONCLUSIONS: In the high risk elderly, exaggerated visit-to-visit BP variability and advanced carotid artery remodeling have a synergetic association with cognitive dysfunction.
Copyright © 2013 Elsevier Ireland Ltd. All rights reserved.
Version ID
1
Record Owner
From MEDLINE, a database of the U.S. National Library of Medicine.
Status
MEDLINE
Authors Full Name
Nagai, Michiaki; Hoshide, Satoshi; Nishikawa, Mami; Masahisa, Shimpo; Kario, Kazuomi.
Institution
Nagai, Michiaki. Division of Cardiovascular Medicine, Department of Medicine, Jichi Medical University School of Medicine, Yakushiji 3311-1, Shimotsuke, Tochigi 329-0498, Japan. Hoshide, Satoshi. Division of Cardiovascular Medicine, Department of Medicine, Jichi Medical University School of Medicine, Yakushiji 3311-1, Shimotsuke, Tochigi 329-0498, Japan.
Nishikawa, Mami. Division of Cardiovascular Medicine, Department of Medicine, Jichi Medical University School of Medicine, Yakushiji 3311-1, Shimotsuke, Tochigi 329-0498, Japan.
Masahisa, Shimpo. Division of Cardiovascular Medicine, Department of Medicine, Jichi Medical University School of Medicine, Yakushiji 3311-1, Shimotsuke, Tochigi 329-0498, Japan.
Kario, Kazuomi. Division of Cardiovascular Medicine, Department of Medicine, Jichi Medical University School of Medicine, Yakushiji 3311-1, Shimotsuke, Tochigi 329-0498, Japan. Electronic address: kkario@jichi.ac.jp.
MeSH Heading
Aged. Aged, 80 and over. Blood Pressure/de [Drug Effects]. *Blood Pressure. Carotid Arteries/dg [Diagnostic Imaging]. *Carotid Intima-Media Thickness. Cognition Disorders/ep [Epidemiology]. *Cognition Disorders/et [Etiology]. Female. Humans. Japan/ep [Epidemiology]. Male. *Vascular Resistance/ph [Physiology].
Keyword Heading
Carotid artery remodeling   Cognitive impairment
The elderly
Visit-to-visit blood pressure variability.
Year of Publication
2014

Link to the Ovid Full Text or citation:
Click here for full text options

Link to the External Link Resolver:
SFX

61.

Association of cognitive dysfunction with cardiovascular disease events in elderly hypertensive patients.
Yano Y; Bakris GL; Inokuchi T; Ohba Y; Tamaki N; Nagata M; Kuwabara M; Yokota N; Eto T; Kuroki M; Shimada K; Kario K.
Journal of Hypertension. 32(2):423-31, 2014 Feb.
[Journal Article. Research Support, Non-U.S. Gov't]
UI: 24351802
OBJECTIVES: This study assesses whether presence of cognitive dysfunction can be a marker associated with the development of cardiovascular disease (CVD) events independent of ambulatory blood pressure (BP) or other indices of target organ damage (TOD) in elderly hypertensive patients.
METHODS: We recruited 585 hypertensive patients (mean age, 73 years; 41% men) who were ambulatory, lived independently, and were without clinically overt dementia. Cognitive function was assessed by Mini-Mental State Examination (MMSE) at baseline, and CVD events (coronary artery disease, stroke, congestive heart failure, and sudden death) were prospectively ascertained. Cognitive dysfunction was defined as the lowest quartile of MMSE scores (n = 183, median 24 points).
RESULTS: CVD events occurred in 42 people over an average of 2.8 years (1644 person-years). The prevalence of cognitive dysfunction was higher in patients with CVD events than those without (57 vs. 29%; both P <0.001) at baseline. Cognitive dysfunction was associated with CVD events, after adjustment for nocturnal SBP and evidence of TOD [i.e. albuminuria, cardiac hypertrophy, and carotid-artery intima-media thickness (IMT)], hazard ratio 2.5-2.9 (all P <0.01). Incorporation of MMSE in the risk model (including age, estimated glomerular filtration rate, and preexisting CVD) improved the C-statistics (from 0.691 to 0.741) and resulted in a net reclassification improvement of

17.6% (P = 0.02). In contrast, incorporation of albuminuria, cardiac hypertrophy, and high carotid-artery IMT added little further improvement in the risk prediction.
CONCLUSION: Cognitive dysfunction is an independent marker associated with increased risk of CVD events in elderly hypertensive patients.
Version ID
1
Record Owner
From MEDLINE, a database of the U.S. National Library of Medicine.
Status
MEDLINE
Authors Full Name
Yano, Yuichiro; Bakris, George L; Inokuchi, Takashi; Ohba, Yusuke; Tamaki, Noboru; Nagata, Masahiko; Kuwabara, Masachika; Yokota, Naoto; Eto, Takuma; Kuroki, Munetoshi; Shimada, Kazuyuki; Kario, Kazuomi.
Institution
Yano, Yuichiro. aAmerican Society of Hypertension Comprehensive Hypertension Center, Section of Endocrinology, Diabetes and Metabolism, Department of Medicine, University of Chicago Medicine, Chicago, Illinois, USA bDivision of Cardiovascular Medicine, Department of Medicine, Jichi Medical University School of Medicine, Tochigi cDepartment of Orthopedics, Chikamorikai Medical Group, Kochi dTamaki Clinic eKijo Clinic fKuwabara Clinic gYokota Naika hEto Cardiology Clinic iKuroki Naika, Miyazaki, Japan.
MeSH Heading
Aged. Aged, 80 and over. Blood Pressure. *Cardiovascular Diseases/et [Etiology]. Cardiovascular Diseases/pp [Physiopathology]. Cardiovascular Diseases/px [Psychology]. *Cognition Disorders/co [Complications]. Cognition Disorders/px [Psychology]. Cohort Studies. Female. Follow-Up Studies. Humans. *Hypertension/co [Complications]. Hypertension/pp [Physiopathology]. Hypertension/px [Psychology]. Male. Mental Status Schedule. Middle Aged. Prognosis. Proportional Hazards Models. Risk Factors.
Year of Publication
2014

Link to the Ovid Full Text or citation:
Click here for full text options

Link to the External Link Resolver:
SFX

63.

Correlation of cognitive function with ultrasound strain indices in carotid plaque.
Wang X; Jackson DC; Varghese T; Mitchell CC; Hermann BP; Kliewer MA; Dempsey RJ.
Ultrasound in Medicine & Biology. 40(1):78-89, 2014 Jan.
[Journal Article. Research Support, N.I.H., Extramural]
UI: 24120415

Instability in carotid vulnerable plaque can generate cerebral micro-emboli, which may be related to both stroke and eventual cognitive abnormality. Strain imaging to detect plaque vulnerability based on regions with large strain fluctuations, with arterial pulsation, may be able to determine the risk of cognitive impairment. Plaque instability may be characterized by increased strain variations over a cardiac cycle. Radiofrequency signals for ultrasound strain imaging were acquired from the carotid arteries of 24 human patients using a Siemens Antares with a VFX 13-5 linear array transducer. These patients underwent standardized cognitive assessment (Repeatable Battery for the Assessment of Neuropsychological Status [RBANS]). Plaque regions were segmented by a radiologist at end-diastole using the Medical Imaging Interaction Toolkit. A hierarchical block-matching motion tracking algorithm was used to estimate the cumulated axial, lateral and shear strains within the imaging plane. The maximum, minimum and peak-to-peak strain indices in the plaque computed from the mean cumulated strain over a small region of interest in the plaque with large deformations were obtained. The maximum and peak-to-peak mean cumulated strain indices over the entire plaque region were also computed. All strain indices were then correlated with RBANS Total performance. Overall cognitive performance (RBANS Total) was negatively associated with values of the maximum strain and the peak-to-peak for axial and lateral strains, respectively. There was no significant correlation between the RBANS Total score and shear strain and strain indices averaged over the entire identified plaque for this group of patients. However, correlation of maximum lateral strain was higher for symptomatic patients ($r = -0.650$, $p = 0.006$) than for asymptomatic patients ($r = -0.115$, $p = 0.803$). On the other hand, correlation of maximum axial strain averaged over the entire plaque region was significantly higher for asymptomatic patients ($r = -0.817$, $p = 0.016$) than for symptomatic patients ($r = -0.224$, $p = 0.402$). The results reveal a direct relationship between the maximum axial and lateral strain indices in carotid plaque and cognitive impairment.

Copyright © 2014 World Federation for Ultrasound in Medicine & Biology. Published by Elsevier Inc. All rights reserved.

Version ID
1
Record Owner
From MEDLINE, a database of the U.S. National Library of Medicine.
Status
MEDLINE
Authors Full Name
Wang, Xiao; Jackson, Daren C; Varghese, Tomy; Mitchell, Carol C; Hermann, Bruce P; Kliewer, Mark A; Dempsey, Robert J.
Institution
Wang, Xiao. Department of Medical Physics, University of Wisconsin-Madison, Madison, Wisconsin, USA.
MeSH Heading
Adult. Aged. *Algorithms. *Carotid Stenosis/co [Complications]. *Carotid Stenosis/dg [Diagnostic Imaging]. Carotid Stenosis/pp [Physiopathology]. *Cognition Disorders/dg [Diagnostic Imaging]. *Cognition Disorders/et [Etiology]. Cognition Disorders/pp [Physiopathology]. Elastic Modulus. Elasticity Imaging Techniques/mt [Methods]. Female. Humans. Image Enhancement/mt [Methods]. *Image Interpretation, Computer-Assisted/mt [Methods]. Male. Middle Aged. Plaque, Atherosclerotic/co [Complications]. *Plaque, Atherosclerotic/dg [Diagnostic Imaging]. Prognosis. Reproducibility of Results. Risk Assessment. Sensitivity and Specificity. Statistics as Topic. Stress, Mechanical.
Keyword Heading
Carotid plaque   Displacement
Elasticity imaging
Elastography
Motion tracking
Multi-level
Strain
Vascular cognitive dementia.
Year of Publication
2014

Link to the Ovid Full Text or citation:
[Click here for full text options]

Link to the External Link Resolver:

SFX

65.

Albuminuria and carotid atherosclerosis as predictors of cognitive function in a general population.
Rogne SO; Solbu MD; Arntzen KA; Herder M; Mathiesen EB; Schirmer H.
European Neurology. 70(5-6):340-8, 2013.
[Journal Article]
UI: 24158160
BACKGROUND/AIMS: Albuminuria and carotid atherosclerosis are predictors of cardiovascular disease and potential predictors of cognitive decline. Our aim was to study whether albuminuria was an early predictor of cognitive function independent of carotid atherosclerosis in a general population.
METHODS: The study population comprised 1,577 adults without self-reported stroke. In 1994 and 2007 all were screened for cardiovascular risk factors, urinary albumin-creatinine ratio (ACR), carotid intima-media thickness and carotid total plaque area (TPA). Endpoints were neuropsychological test results in 2007 from the digit symbol test, the finger-tapping test, the Mini Mental Status Examination and the 12-word test parts 1 and 2. Multivariate linear regression was used to assess associations.
RESULTS: Higher ACR, DELTAACR, intima-media thickness, TPA and DELTATPA independently predicted a lower score on the digit symbol test. Higher DELTAACR and DELTATPA predicted a lower score on the finger-tapping test. Higher TPA predicted a lower score on the 12-word test part 1 (immediate recall). Smoking predicted lower scores on the digit symbol and finger-tapping tests independent of albuminuria and carotid atherosclerosis.
CONCLUSIONS: Our results suggest that albuminuria, carotid atherosclerosis and smoking are independent predictors of executive function and motor tempo.
Copyright © 2013 S. Karger AG, Basel.
Version ID
1
Record Owner
From MEDLINE, a database of the U.S. National Library of Medicine.
Status
MEDLINE
Authors Full Name

Rogne, Sigbjorn O; Solbu, Marit D; Arntzen, Kjell Arne; Herder, Marit; Mathiesen, Ellisiv B; Schirmer, Henrik.
Institution
Rogne, Sigbjorn O. Department of Clinical Medicine, University of Tromso, Tromso, Norway.
MeSH Heading
Albuminuria/dg [Diagnostic Imaging]. *Albuminuria/pp [Physiopathology]. Carotid Artery Diseases/dg [Diagnostic Imaging]. *Carotid Artery Diseases/pp [Physiopathology]. Carotid Intima-Media Thickness/px [Psychology]. *Carotid Intima-Media Thickness. *Cognition/ph [Physiology]. *Cognition Disorders/di [Diagnosis]. Cognition Disorders/pp [Physiopathology]. Cognition Disorders/px [Psychology]. Female. Humans. Male. Middle Aged. Neuropsychological Tests. Predictive Value of Tests. Risk Factors.
Year of Publication
2013

Link to the Ovid Full Text or citation:
Click here for full text options

Link to the External Link Resolver:
SFX

66.

Asymptomatic cervicocerebral atherosclerosis, intracranial vascular resistance and cognition: the AsIA-neuropsychology study.
Lopez-Oloriz J; Lopez-Cancio E; Arenillas JF; Hernandez M; Jimenez M; Dorado L; Barrios M; Soriano-Raya JJ; Miralbell J; Caceres C; Fores R; Pera G; Davalos A; Mataro M.
Atherosclerosis. 230(2):330-5, 2013 Oct.
[Journal Article. Research Support, Non-U.S. Gov't]
UI: 24075765
BACKGROUND AND PURPOSE: Carotid atherosclerosis has emerged as a relevant contributor to cognitive impairment and dementia whereas the role of intracranial stenosis and vascular resistance in cognition remains unknown. This study aims to assess the association of

asymptomatic cervicocerebral atherosclerosis and intracranial vascular resistance with cognitive performance in a large dementia-free population.
METHODS: The Barcelona-AsIA (Asymptomatic Intracranial Atherosclerosis) Neuropsychology Study included 747 Caucasian subjects older than 50 with a moderate-high vascular risk (assessed by REGICOR score) and without history of neither symptomatic vascular disease nor dementia. Extracranial and transcranial color-coded duplex ultrasound examination was performed to assess carotid intima-media thickness (IMT), presence of carotid plaques (ECAD group), intracranial stenosis (ICAD group), and middle cerebral artery pulsatility index (MCA-PI) as a measure of intracranial vascular resistance. Neuropsychological assessment included tests in three cognitive domains: visuospatial skills and speed, verbal memory and verbal fluency.
RESULTS: In univariate analyses, carotid IMT, ECAD and MCA-PI were associated with lower performance in almost all cognitive domains, and ICAD was associated with poor performance in some visuospatial and verbal cognitive tests. After adjustment for age, sex, vascular risk score, years of education and depressive symptoms, ECAD remained associated with poor performance in the three cognitive domains and elevated MCA-PI with worse performance in visuospatial skills and speed.
CONCLUSIONS: Carotid plaques and increased intracranial vascular resistance are independently associated with low cognitive functioning in Caucasian stroke and dementia-free subjects. We failed to find an independent association of intracranial large vessel stenosis with cognitive performance.
Copyright © 2013 Elsevier Ireland Ltd. All rights reserved.
Version ID
1
Record Owner
From MEDLINE, a database of the U.S. National Library of Medicine.
Status
MEDLINE
Authors Full Name
Lopez-Oloriz, Jorge; Lopez-Cancio, Elena; Arenillas, Juan F; Hernandez, Maria; Jimenez, Marta; Dorado, Laura; Barrios, Maite; Soriano-Raya, Juan Jose; Miralbell, Julia; Caceres, Cynthia; Fores, Rosa; Pera, Guillem; Davalos, Antoni; Mataro, Maria.
Institution
Lopez-Oloriz, Jorge. Department of Psychiatry and Clinical Psychobiology, Universitat de Barcelona, 08035 Barcelona, Spain; Institute for Brain, Cognition and Behavior (IR3C), Universitat de Barcelona, 08035 Barcelona, Spain.
MeSH Heading

Aged. Aged, 80 and over. Carotid Arteries/dg [Diagnostic Imaging]. Carotid Arteries/pa [Pathology]. *Carotid Artery Diseases/co [Complications]. Carotid Artery Diseases/pa [Pathology]. Carotid Intima-Media Thickness. *Carotid Stenosis/co [Complications]. Carotid Stenosis/pa [Pathology]. *Cognition/ph [Physiology]. *Cognition Disorders/co [Complications]. Cognition Disorders/pa [Pathology]. Cross-Sectional Studies. Female. Humans. *Intracranial Arteriosclerosis/co [Complications]. Intracranial Arteriosclerosis/pa [Pathology]. Male. Middle Aged. Multivariate Analysis. Neuropsychological Tests. Neuropsychology. Risk Assessment. Stroke/co [Complications]. Stroke/pa [Pathology]. Ultrasonography. *Vascular Resistance.

Keyword Heading

Atherosclerosis Cognitive impairment

Intima-media thickness

Intracranial stenosis

Pulsatility index.

Year of Publication

2013

Link to the Ovid Full Text or citation:

Click here for full text options

Link to the External Link Resolver:

SFX

67.

Carotid artery atherosclerosis is correlated with cognitive impairment in an elderly urban Chinese non-stroke population.

Xiang J; Zhang T; Yang QW; Liu J; Chen Y; Cui M; Yin ZG; Li L; Wang YJ; Li J; Zhou HD.

Journal of Clinical Neuroscience. 20(11):1571-5, 2013 Nov.

[Journal Article]

UI: 23978769

Carotid artery atherosclerosis may cause increased intima-media thickness (IMT), plaque formation, and vessel stenosis or occlusion. However, the association between carotid artery atherosclerosis and cognitive impairment remains uncertain. This study explored the effects of IMT

and carotid artery stenosis on cognitive function in an elderly Chinese non-stroke population. A total of 2015 patients were recruited. The IMT of carotid arteries and the presence of plaques and stenosis in carotid arteries were assessed with B-mode ultrasound examination. Cognitive performance was evaluated with neuropsychological tests. The cross-sectional relationships between cognitive performance and carotid wall characteristics were analyzed. Carotid artery atherosclerosis (IMT>1.0) and stenosis were found in 86% and 51% of patients, respectively. Cognitive impairment was found in 356 (17.7%) patients. After adjustment for possible confounders, IMT (odds ratio [OR]=1.96; 95% confidence interval [CI] 1.23-3.16) and hyperdense plaque (OR=4.72; 95% CI 2.56-11.2) were associated with poor cognitive performance. Patients with severe (>=70%) carotid artery stenosis had a lower Mini-Mental State Examination score compared with the mild to modest (40-70%) carotid artery stenosis group. Cognitive performance differed between patients with left and right carotid artery stenosis, but no differences were observed between patients with severe left and right carotid artery stenosis. This study indicates that carotid artery atherosclerosis is correlated with cognitive impairment in the elderly Chinese population. A larger sample size across multiple centers and a longitudinal study are required to further explore the impact of carotid artery atherosclerosis on cognition in the elderly population.
Copyright © 2013 Elsevier Ltd. All rights reserved.
Version ID
1
Record Owner
From MEDLINE, a database of the U.S. National Library of Medicine.
Status
MEDLINE
Authors Full Name
Xiang, Jing; Zhang, Tao; Yang, Qing-Wu; Liu, Juan; Chen, Yong; Cui, Ming; Yin, Ze-Gang; Li, Ling; Wang, Yan-Jiang; Li, Jing; Zhou, Hua-Dong.
Institution
Xiang, Jing. Department of Neurology, Daping Hospital, The Third Military Medical University, Yangzi River Street, Yuzhong District, Chongqing 400042, China.
MeSH Heading
Aged. Aged, 80 and over. Asian Continental Ancestry Group. *Atherosclerosis/co [Complications]. Atherosclerosis/dg [Diagnostic Imaging]. Carotid Intima-Media Thickness. *Carotid Stenosis/co [Complications]. Carotid Stenosis/dg [Diagnostic Imaging]. *Cognition Disorders/co [Complications]. Cognition Disorders/dg [Diagnostic Imaging]. Female. Humans. Male. Neuropsychological Tests.
Keyword Heading
Carotid artery atherosclerosis   Cognitive impairment

Intima-media thickness
Mini-mental state examination
Neuropsychological tests
Stenosis.
Year of Publication
2013

Link to the Ovid Full Text or citation:
[Click here for full text options]

Link to the External Link Resolver:
[SFX]

68.

Atherosclerosis and physical functioning in older men, a longitudinal study.
den Ouden ME; Schuurmans MJ; Arts IE; Grobbee DE; Bots ML; van den Beld AW; Lamberts SW; van der Schouw YT.
Journal of Nutrition, Health & Aging. 17(1):97-104, 2013 Jan.
[Journal Article. Research Support, Non-U.S. Gov't]
UI: 23299387
OBJECTIVE: Functional decline is a major threat to independency, progressing into functional limitations and eventually leading to disability. Chronic diseases, especially cardiovascular diseases, are important determinants of functional limitations and disability. Vascular damage exits long before it is clinically manifest and can have adverse effects on health, physical and cognitive functioning. The objective was to investigate the association between non-invasive atherosclerosis measures and physical functioning in older men.
DESIGN: Prospective cohort study.
SETTING: The study was conducted in the general community.
PARTICIPANTS: 195 independently living older men.
MEASUREMENTS: Atherosclerosis was measured by intima media thickness (CIMT) of the common carotid artery using ultrasonography and assessment for presence of atherosclerotic plaques.
Physical functioning was measured by isometric handgrip strength and leg extensor strength using

a hand held dynamometer, lower extremity function using the physical performance score and ability to perform activities of daily life using the modified Stanford health assessment questionnaire. Linear regression analysis was performed to estimate the associations between CIMT or plaques and physical functioning.
RESULTS: After adjustment for confounders, higher baseline CIMT was associated with lower isometric handgrip strength at follow up (betaCIMT = -7.21, 95% CI[-13.64;-0.77]). No other associations were found between CIMT and physical functioning. In addition, no associations were found for the presence of plaques and physical functioning either at baseline, or at follow-up.
CONCLUSION: Atherosclerosis, as measured by higher CIMT, is related to a lower isometric handgrip strength at follow-up, but no further associations with physical functioning were found in this longitudinal study among independently living older men.
Version ID
1
Record Owner
From MEDLINE, a database of the U.S. National Library of Medicine.
Status
MEDLINE
Authors Full Name
den Ouden, M E M; Schuurmans, M J; Arts, I E M A; Grobbee, D E; Bots, M L; van den Beld, A W; Lamberts, S W J; van der Schouw, Y T.
Institution
den Ouden, M E M. Julius Center for Health Sciences and Primary Care, STR 6.131, University Medical Center Utrecht, P.O. Box 85500, 3508 GA Utrecht, The Netherlands. M.E.M.denOuden@umcutrecht.nl
MeSH Heading
Aged. Aged, 80 and over. *Atherosclerosis/dg [Diagnostic Imaging]. *Atherosclerosis/pp [Physiopathology]. Carotid Artery, Common/dg [Diagnostic Imaging]. Carotid Artery, Common/pp [Physiopathology]. Carotid Intima-Media Thickness. Cognition/ph [Physiology]. Disability Evaluation. Follow-Up Studies. Hand Strength/ph [Physiology]. Humans. Independent Living. Linear Models. Longitudinal Studies. Male. *Motor Activity. Prospective Studies. Risk Factors. Surveys and Questionnaires.
Year of Publication
2013

Link to the Ovid Full Text or citation:
Click here for full text options

Link to the External Link Resolver:
SFX

70.

Carotid atherosclerosis and prospective risk of dementia.
Wendell CR; Waldstein SR; Ferrucci L; O'Brien RJ; Strait JB; Zonderman AB.
Stroke. 43(12):3319-24, 2012 Dec.
[Journal Article. Research Support, N.I.H., Intramural]
UI: 23103489
BACKGROUND AND PURPOSE: Although vascular risk factors have been implicated in the development of all-cause dementia and Alzheimer disease (AD), few studies have examined the association between subclinical atherosclerosis and prospective risk of dementia.
METHODS: Participants from the Baltimore Longitudinal Study of Aging (n=364; age, 60-95 years; median age, 73; 60% male; 82% white) underwent initial carotid atherosclerosis assessment and subsequently were assessed for dementia and AD annually for up to 14 years (median, 7.0). Cox proportional hazards models predicting all-cause dementia and AD were adjusted for age, sex, race, education, blood pressure, cholesterol, cardiovascular disease, diabetes mellitus, and smoking.
RESULTS: Sixty participants developed dementia, with 53 diagnosed as AD. Raw rates of future dementia and AD among individuals initially in the upper quintile of carotid intimal medial thickness or with bilateral carotid plaque were generally double the rates of individuals with intimal medial thickness in the lower quintiles or no plaque at baseline. Adjusted proportional hazards models revealed >2.5-fold increased risk of dementia and AD among individuals in the upper quintile of carotid intimal medial thickness, and approximately 2.0-fold increased risk of dementia among individuals with bilateral plaque.
CONCLUSIONS: Multiple measures of carotid atherosclerosis are associated with prospective risk of dementia. Individuals in the upper quintile of carotid intimal medial thickness or bilateral carotid plaque were at greatest risk. These findings underscore the possibility that early intervention to reduce atherosclerosis may help delay or prevent onset of dementia and AD.
Version ID
1
Record Owner

From MEDLINE, a database of the U.S. National Library of Medicine.
Status
MEDLINE
Authors Full Name
Wendell, Carrington R; Waldstein, Shari R; Ferrucci, Luigi; O'Brien, Richard J; Strait, James B; Zonderman, Alan B.
Institution
Wendell, Carrington R. Intramural Research Program, National Institute on Aging, NIH, 251 Bayview Blvd, Baltimore, MD 21224, USA. cwendell@jhmi.edu
MeSH Heading
Aged. Aged, 80 and over. Aging. *Alzheimer Disease/ep [Epidemiology]. Carotid Artery Diseases/dg [Diagnostic Imaging]. *Carotid Artery Diseases/ep [Epidemiology]. *Carotid Intima-Media Thickness/sn [Statistics & Numerical Data]. Carotid Stenosis/dg [Diagnostic Imaging]. *Carotid Stenosis/ep [Epidemiology]. *Dementia/ep [Epidemiology]. Female. Follow-Up Studies. Humans. Incidence. Longitudinal Studies. Male. Middle Aged. Predictive Value of Tests. Proportional Hazards Models. Prospective Studies. Risk Factors. Severity of Illness Index.
Year of Publication
2012

Link to the Ovid Full Text or citation:
Click here for full text options

Link to the External Link Resolver:
SFX

71.

Carotid intima-media thickness is associated with cognitive deficiency in hypertensive patients with elevated central systolic blood pressure.
Dias Eda M; Giollo LT Jr; Martinelli DD; Mazeti C; Junior HM; Vilela-Martin JF; Yugar-Toledo JC.
Cardiovascular Ultrasound. 10:41, 2012 Oct 18.
[Comparative Study. Journal Article]
UI: 23078629

BACKGROUND: The role of hypertension in the loss of cognitive function is controversial. Relationships between hypertension and increases in cerebral vascular resistance, diffused lesions and multiple lacunar infarcts of the white matter are well known. Thus, the objectives of this study were: to evaluate the relationship between hypertension and cognitive dysfunction (CD), identify risk factors and determine the association between early markers of vascular disease and CD in hypertensive individuals.
METHODS: Two hundred individuals aged between 40 and 80 years old were evaluated in this cross-sectional prospective study. Fifty participants were controls (CT). The remaining 150 hypertensive patients were subdivided into two groups, those with CD (HCD) and those without CD (HNCD). All participants underwent clinical evaluations and biochemical blood tests were performed. CD was investigated using the Mini Mental State Examination (MMSE) following the guidelines for its use in Brazil. The impact of hypertension on the arterial bed was assessed by identifying and measuring changes in the intima-media thickness (IMT) by vascular ultrasonography of the carotid arteries and analyses of the central blood pressure and Augmentation Index by applanation tonometry of the radial artery.
RESULTS: There were no significant differences in the total cholesterol, high-density lipoprotein cholesterol and triglycerides plasma concentrations between the three groups. The serum creatinine and estimated glomerular filtration rate were within normal ranges for all three groups. A significantly lower MMSE score was recorded for the HCD Group compared to the HNCD and CT Groups (p-value < 0.05). The IMT was significantly different between the HNCD and HCD Groups (p-value = 0.0124). A significant difference in the IMT was also observed between hypertensive patients and the CT Group (p-value < 0.0001). Age, low-density cholesterol, high-density cholesterol, triglycerides and IMT increased the Odds Ratio for cognitive dysfunction. The central systolic pressure was significantly higher in the HCD and HNCD Groups compared to CT Group (p-value < 0.0001).
CONCLUSIONS: Hypertensive patients with CD have changes in the vascular morphology characterized by an increased carotid IMT, enhanced atherosclerotic lipid profile and impaired hemodynamic functional manifested by elevated central systolic blood pressure.
Version ID
1
Record Owner
From MEDLINE, a database of the U.S. National Library of Medicine.
Status
MEDLINE
Authors Full Name
Dias, Eros da Mota; Giollo, Luiz Tadeu Jr; Martinelli, Debora Dada; Mazeti, Camila; Junior, Heitor Moreno; Vilela-Martin, Jose Fernando; Yugar-Toledo, Juan Carlos.

Institution
Dias, Eros da Mota. Hypertension Clinic, Department of Internal Medicine, State Medical School of Sao Jose do Rio Preto, Brazil.
MeSH Heading
Adult. Aged. Aged, 80 and over. *Blood Pressure/ph [Physiology]. Brazil/ep [Epidemiology]. *Carotid Arteries/dg [Diagnostic Imaging]. Carotid Arteries/pp [Physiopathology]. *Carotid Intima-Media Thickness. *Cognition/ph [Physiology]. Cognition Disorders/ep [Epidemiology]. *Cognition Disorders/et [Etiology]. Cognition Disorders/pp [Physiopathology]. Cross-Sectional Studies. Female. Follow-Up Studies. Humans. Hypertension/co [Complications]. *Hypertension/dg [Diagnostic Imaging]. Hypertension/pp [Physiopathology]. Incidence. Male. Middle Aged. Prognosis. Prospective Studies. Risk Factors. Severity of Illness Index. Systole.
Year of Publication
2012

Link to the Ovid Full Text or citation:
Click here for full text options

Link to the External Link Resolver:
SFX

72.

Vascular risk factors in Alzheimer's disease - preliminary report.
Stefanova E; Pavlovic A; Jovanovic Z; Veselinovic N; Despotovic I; Stojkovic T; Sternic N; Kostic V.
Journal of the Neurological Sciences. 322(1-2):166-9, 2012 Nov 15.
[Journal Article. Research Support, Non-U.S. Gov't]
UI: 22938734
BACKGROUND: The vascular risk factors are associated with an increased risk for vascular cognitive decline (VCD), but also with Alzheimer disease (AD).
OBJECTIVE: To investigate vascular risk factors in relation to AD and VCD, with a non-invasive neurosonological methods in a clinical settings.
RESULTS: A total of 296 patients with AD and 237 patients with VCD were included in the study. Hypertension, hyperlipidemia, diabetes mellitus, stroke, and white matter changes ($p<0.001$) were

significantly more prevalent in VCD, although they were also present in AD patients. No statistically significant differences were obtained between groups regarding coronary disease, atrial fibrillation, average degree of carotid artery stenosis and carotid intima-media thickness (cITM). However, the patients with AD had carotid artery stenosis ">50%" (p=0.007) and present plaques (p<0.001) more frequently compared to vascular group. The significant associations between robust cognitive measure and vascular factors, diabetes mellitus, carotid stenosis, cITM, and type of plaques were identified only in VCD, but not in AD group.
CONCLUSIONS: The vascular risk factors were more prevalent in VCD group, although they were also present in AD. With few treatment options available in AD, it may be important not to neglect the vascular risk factors.
Copyright © 2012 Elsevier B.V. All rights reserved.
Version ID
1
Record Owner
From MEDLINE, a database of the U.S. National Library of Medicine.
Status
MEDLINE
Authors Full Name
Stefanova, E; Pavlovic, A; Jovanovic, Z; Veselinovic, N; Despotovic, I; Stojkovic, T; Sternic, N; Kostic, V.
Institution
Stefanova, E. Faculty of Medicine, University of Belgrade, Serbia; Clinic of Neurology CCS, Serbia. steela21@gmail.com
MeSH Heading
Aged. Aged, 80 and over. Alzheimer Disease/di [Diagnosis]. *Alzheimer Disease/ep [Epidemiology]. Carotid Arteries/dg [Diagnostic Imaging]. Carotid Intima-Media Thickness. Cerebrovascular Disorders/di [Diagnosis]. *Cerebrovascular Disorders/ep [Epidemiology]. Female. Humans. Magnetic Resonance Imaging. Male. Mental Status Schedule. Middle Aged. Retrospective Studies. Risk Factors. Statistics, Nonparametric. Tomography, X-Ray Computed.
Year of Publication
2012

Link to the Ovid Full Text or citation:
Click here for full text options

Link to the External Link Resolver:

SFX

73.

Carotid atherosclerosis and 10-year changes in cognitive function.
Zhong W; Cruickshanks KJ; Schubert CR; Acher CW; Carlsson CM; Klein BE; Klein R; Chappell RJ.
Atherosclerosis. 224(2):506-10, 2012 Oct.
[Journal Article. Research Support, N.I.H., Extramural]
UI: 22854188
BACKGROUND: Carotid atherosclerosis has been suggested to be involved in cognitive decline.
METHODS: The Epidemiology of Hearing Loss Study is a longitudinal study of aging among Beaver Dam residents, WI. In 1998-2000, carotid intima-media thickness (IMT) and plaque were measured by ultrasound; cognitive function was measured by the Mini-Mental State Examination (MMSE). Follow-up examinations were conducted in 2003-2005 and 2009-2010. Incidence of cognitive impairment was defined as an MMSE score <24 or reported physician-diagnosed dementia during the follow-up. In the last examination, five additional cognitive tests were added. The associations of carotid atherosclerosis with incident cognitive impairment and cognitive test performance ten years later were evaluated.
RESULTS: A total of 1651 participants (mean age 66.8 years, 41% men) without cognitive impairment at baseline were included in the incidence analysis. IMT was associated with incidence of cognitive impairment after multiple adjustments (hazard ratio: 1.09, p = 0.02 for each 0.1 mm increase in IMT). A total of 1311 participants with atherosclerosis data at baseline had the additional cognitive tests 10 years later. Larger IMT was associated with longer time to complete the Trail-Making Test-part B after multiple adjustments (0.1 mm IMT: 2.3 s longer, p = 0.02). Plaque was not associated with incident cognitive impairment or cognitive test performance 10 years later.
CONCLUSIONS: In this population-based longitudinal study, carotid IMT was associated with a higher risk of developing cognitive impairment during the 10-year follow-up, and was associated with poorer performance in a test of executive function 10 years later.
Copyright Published by Elsevier Ireland Ltd.
Version ID
1
Record Owner
From MEDLINE, a database of the U.S. National Library of Medicine.
Status

MEDLINE
Authors Full Name
Zhong, Wenjun; Cruickshanks, Karen J; Schubert, Carla R; Acher, Charles W; Carlsson, Cynthia M; Klein, Barbara E K; Klein, Ronald; Chappell, Richard J.
Institution
Zhong, Wenjun. University of Wisconsin-Madison, 1036 WARF, 610 Walnut Street, WI 53726, USA. zhongwenjun80@gmail.com
MeSH Heading
Adult. Aged. Aged, 80 and over. Carotid Arteries/dg [Diagnostic Imaging]. Carotid Artery Diseases/dg [Diagnostic Imaging]. *Carotid Artery Diseases/ep [Epidemiology]. Carotid Artery Diseases/px [Psychology]. Carotid Intima-Media Thickness. *Cognition. Cognition Disorders/di [Diagnosis]. *Cognition Disorders/ep [Epidemiology]. Cognition Disorders/px [Psychology]. Female. Humans. Incidence. Linear Models. Logistic Models. Longitudinal Studies. Male. Middle Aged. Multivariate Analysis. Neuropsychological Tests. Odds Ratio. Plaque, Atherosclerotic. Predictive Value of Tests. Psychiatric Status Rating Scales. Risk Assessment. Risk Factors. Time Factors. Wisconsin/ep [Epidemiology].
Year of Publication
2012

Link to the Ovid Full Text or citation:
Click here for full text options

Link to the External Link Resolver:
SFX

74.

Impaired cognitive function in patients with atherosclerotic carotid stenosis and correlation with ultrasound strain measurements.
Rocque BG; Jackson D; Varghese T; Hermann B; McCormick M; Kliewer M; Mitchell C; Dempsey RJ.
Journal of the Neurological Sciences. 322(1-2):20-4, 2012 Nov 15.
[Journal Article. Research Support, N.I.H., Extramural]
UI: 22658531

INTRODUCTION: It has been postulated that up to 11 million "silent" strokes occur annually. While these patients are without classic neurologic deficits, they may exhibit cognitive decline. In this study, we examine the cognitive function of patients with carotid stenosis. Additionally, we evaluate a noninvasive measure of strain in pulsating carotid artery plaques to determine its ability to predict cognitive decline.
METHODS: We administered the Repeatable Battery for the Assessment of Neuropsychological Status (RBANS) to 44 patients with carotid stenosis. All patients had stenosis meeting NASCET or ACAS criteria for endarterectomy, and were classified as symptomatic or asymptomatic as defined by these publications. Age-adjusted scores for each of the 5 RBANS domains (immediate memory, visuospatial ability, language, attention, and delayed memory) were compared between symptomatic and asymptomatic patients. Mean score for each of the 5 domains was then compared to all other domains, regardless of symptom status. From this cohort, 23 patients underwent assessment of carotid plaque strain by tracking displacements in ultrasound radiofrequency data to estimate axial and principal strains over the cardiac cycle.
RESULTS: Thirty symptomatic and 14 asymptomatic patients were studied. Visuospatial scores were significantly lower than any other domain regardless of symptoms ($p<0.05$ for all pairwise comparisons). No other domain score was significantly different from any other. In the language domain, asymptomatic patients scored significantly higher than symptomatic patients ($p<0.05$. For all other domains, no difference was found. Asymptomatic patients showed a relationship between plaque strain and immediate memory (r=-.61, p=ns). Left carotid disease was associated with poorer performance across multiple cognitive domains with increasing accumulated strain. This was not seen in right carotid disease.
CONCLUSION: Patients with large carotid plaques (>70% stenosis) exhibit significant difficulties in mental status whether classically symptomatic or asymptomatic. While language deficits may be a non-specific marker for stroke symptoms, visuospatial deficits are seen before classic symptoms, suggesting that carotid disease may become symptomatic earlier and more subtly than previously suspected. Abnormal strain distribution with pulsation may be related to cognition.
Copyright © 2012 Elsevier B.V. All rights reserved.
Version ID
1
Record Owner
From MEDLINE, a database of the U.S. National Library of Medicine.
Status
MEDLINE
Authors Full Name
Rocque, Brandon G; Jackson, Daren; Varghese, Tomy; Hermann, Bruce; McCormick, Matthew; Kliewer, Mark; Mitchell, Carol; Dempsey, Robert J.

Institution
Rocque, Brandon G. UW-Madison School of Medicine and Public Health, Department of Neurological Surgery, 600 Highland Avenue, K4/866, Madison, WI 53792, United States.
MeSH Heading
Attention. Brain/pa [Pathology]. *Carotid Artery Diseases/co [Complications]. Carotid Artery Diseases/su [Surgery]. *Cognition Disorders/dg [Diagnostic Imaging]. *Cognition Disorders/et [Etiology]. Cohort Studies. Endarterectomy, Carotid/mt [Methods]. Female. Humans. Language. Male. Memory. Neuropsychological Tests. Space Perception. Statistics as Topic. Ultrasonography.
Year of Publication
2012

Link to the Ovid Full Text or citation:
Click here for full text options

Link to the External Link Resolver:
SFX

76.

Atherosclerotic calcification relates to cognitive function and to brain changes on magnetic resonance imaging.
Bos D; Vernooij MW; Elias-Smale SE; Verhaaren BF; Vrooman HA; Hofman A; Niessen WJ; Witteman JC; van der Lugt A; Ikram MA.
Alzheimer's & Dementia. 8(5 Suppl):S104-11, 2012 Oct.
[Journal Article. Research Support, Non-U.S. Gov't]
UI: 22537801
BACKGROUND: Increasing evidence suggests a role of atherosclerosis in the pathogenesis of cognitive impairment and dementia. Calcification volume measured with computed tomography (CT) is a valid marker of atherosclerosis. This study investigates associations of atherosclerosis (measured using CT) at four locations with cognition and brain changes on magnetic resonance imaging (MRI).
METHODS: To quantify calcification volume, 2414 nondemented people from the Rotterdam Study underwent CT of the coronary arteries, aortic arch, extracranial carotid arteries, and intracranial

carotid arteries. To assess global cognition and performance on memory, executive function, information processing speed, and motor speed, they also underwent neuropsychological tests. In a random subgroup of 844 participants, brain MRI was performed. Automated segmentation and quantification of brain MRI scans yielded brain tissue volumes in milliliters. Diffusion tensor imaging was used to measure the microstructural integrity of the white matter. Relationships of atherosclerotic calcification with cognition, brain tissue volumes, and diffusion tensor imaging measures were assessed with linear regression models and adjusted for relevant confounders.
RESULTS: With larger calcification volumes, lower cognitive scores were observed. When calcification volumes were larger, total brain volumes were also smaller. Specifically, larger coronary artery calcification volumes related to smaller gray matter volumes, and extracranial and intracranial carotid calcification volumes related to smaller white matter volumes. Larger calcification volume in all vessel beds was accompanied by worse microstructural integrity of the white matter.
CONCLUSIONS: Larger calcification volume is associated with worse cognitive performance. It also relates to smaller brain tissue volumes and worse white matter microstructural integrity, revealing possible mechanisms through which atherosclerosis may lead to poorer cognition.
Copyright © 2012 The Alzheimer's Association. Published by Elsevier Inc. All rights reserved.
Version ID
1
Record Owner
From MEDLINE, a database of the U.S. National Library of Medicine.
Status
MEDLINE
Authors Full Name
Bos, Daniel; Vernooij, Meike W; Elias-Smale, Suzette E; Verhaaren, Benjamin F J; Vrooman, Henri A; Hofman, Albert; Niessen, Wiro J; Witteman, Jacqueline C M; van der Lugt, Aad; Ikram, M Arfan.
Institution
Bos, Daniel. Department of Radiology, Erasmus MC, Rotterdam, The Netherlands.
MeSH Heading
Aged. Aorta, Thoracic/dg [Diagnostic Imaging]. Aorta, Thoracic/pa [Pathology]. *Atherosclerosis/co [Complications]. Atherosclerosis/dg [Diagnostic Imaging]. Atherosclerosis/pa [Pathology]. Brain/bs [Blood Supply]. Brain/dg [Diagnostic Imaging]. *Brain/pa [Pathology]. *Calcinosis/co [Complications]. Calcinosis/dg [Diagnostic Imaging]. Calcinosis/pa [Pathology]. Carotid Arteries/dg [Diagnostic Imaging]. Carotid Arteries/pa [Pathology]. Cognition Disorders/dg [Diagnostic Imaging]. *Cognition Disorders/et [Etiology]. *Cognition Disorders/pa [Pathology]. Coronary Vessels/dg [Diagnostic Imaging]. Coronary Vessels/pa [Pathology]. Female. Humans. Magnetic Resonance Imaging. Male. Neuropsychological Tests. Tomography, X-Ray Computed.

Year of Publication
2012

Link to the Ovid Full Text or citation:
Click here for full text options

Link to the External Link Resolver:
SFX

77.

Carotid artery plaque progression and cognitive decline: the Tromso Study 1994-2008.
Arntzen KA; Schirmer H; Johnsen SH; Wilsgaard T; Mathiesen EB.
European Journal of Neurology. 19(10):1318-24, 2012 Oct.
[Journal Article. Research Support, Non-U.S. Gov't]
UI: 22537454
BACKGROUND: Carotid atherosclerosis is a risk factor for stroke and cognitive decline, but knowledge on how progression of carotid atherosclerosis affects cognitive function in stroke-free individuals is scarce.
METHODS: In the population-based Tromso study, we calculated the change in ultrasound-assessed carotid plaque number and total plaque area from baseline (survey 4) to follow-up 7 years later (survey 5) in 4274 middle-aged stroke-free subjects. Cognitive function was assessed at follow-up by the verbal memory test, the digit-symbol coding test, and the tapping test and repeated after an additional 6 years in a subgroup of 2042 subjects (survey 6). Associations between the average of survey 4 and survey 5 plaque scores and the progression of plaque scores and cognitive test scores were assessed in regression analyses adjusted for baseline age, sex, education, depression, and cardiovascular risk factors.
RESULTS: Progression of total plaque area was associated with lower scores in the digit-symbol coding test (multivariable adjusted standardized beta, -0.03; 95% CI, -0.05 to -0.00; P = 0.04) and the tapping test (beta, -0.03; 95% CI, -0.06 to -0.00; P = 0.03). Similar results were seen for progression of plaque number. The average plaque scores were associated with lower scores in all cognitive tests (P-values <= 0.01). No association was found between plaque scores and cognitive decline.

CONCLUSIONS: The average plaque scores were associated with lower scores in all cognitive tests. Progression of plaque scores was associated with lower scores in the digit-symbol coding test and the tapping test, but not with the verbal memory test or with cognitive decline.
Copyright © 2012 The Author(s) European Journal of Neurology © 2012 EFNS.
Version ID
1
Record Owner
From MEDLINE, a database of the U.S. National Library of Medicine.
Status
MEDLINE
Authors Full Name
Arntzen, K A; Schirmer, H; Johnsen, S H; Wilsgaard, T; Mathiesen, E B.
Institution
Arntzen, K A. Department of Community Medicine, University of Tromso, Tromso, Norway. kjell.a.arntzen@uit.no
MeSH Heading
Carotid Stenosis/co [Complications]. *Carotid Stenosis/dg [Diagnostic Imaging]. *Cognition. *Cognition Disorders/ep [Epidemiology]. Cognition Disorders/et [Etiology]. Cross-Sectional Studies. Disease Progression. Female. Humans. Male. Middle Aged. Risk Factors. Ultrasonography.
Year of Publication
2012

Link to the Ovid Full Text or citation:
Click here for full text options

Link to the External Link Resolver:
SFX

79.

Carotid atherosclerosis predicts lower cognitive test results: a 7-year follow-up study of 4,371 stroke-free subjects - the Tromso study.
Arntzen KA; Schirmer H; Johnsen SH; Wilsgaard T; Mathiesen EB.

Cerebrovascular Diseases. 33(2):159-65, 2012.
[Journal Article. Research Support, Non-U.S. Gov't]
UI: 22222422
BACKGROUND: Carotid artery atherosclerosis is a major risk factor for stroke and subsequent cognitive impairment. Prospective population studies have shown associations between carotid intima-media thickness (IMT) and stenosis and cognitive decline and dementia in elderly stroke-free persons, whereas results in the middle-aged are conflicting.
METHODS: In this prospective population-based study, 4,371 stroke-free middle-aged participants underwent carotid ultrasound examination and assessment of vascular risk factors at baseline and were tested for cognitive function 7 years later. Associations between IMT, number of plaques and total plaque area and cognitive test scores on verbal memory test, digit symbol-coding test and tapping test were assessed in linear regression models.
RESULTS: In the multivariable analyses adjusted for sex, age, education, depression and vascular risk factors, the presence of plaques was significantly associated with lower test scores on the verbal memory test ($p = 0.01$) and on the digit symbol-coding test ($p = 0.03$). The number of plaques ($p = 0.01$) and the total plaque area ($p = 0.02$) were associated with lower scores on the verbal memory test. No significant association was seen between common carotid artery IMT and cognitive test scores. The tapping test was not associated with the carotid ultrasound variables.
CONCLUSIONS: In this middle-aged general population, subclinical carotid atherosclerosis measured as the presence of plaques, number of plaques and total plaque area were independent long-term predictors of lower cognitive test scores.
Copyright © 2012 S. Karger AG, Basel.
Version ID
1
Record Owner
From MEDLINE, a database of the U.S. National Library of Medicine.
Status
MEDLINE
Authors Full Name
Arntzen, Kjell Arne; Schirmer, Henrik; Johnsen, Stein Harald; Wilsgaard, Tom; Mathiesen, Ellisiv B.
Institution
Arntzen, Kjell Arne. Department of Community Medicine, University of Tromso, Tromso, Norway. kjell.a.arntzen@uit.no
MeSH Heading
Adult. Aged. Attention. Carotid Artery Diseases/dg [Diagnostic Imaging]. *Carotid Artery Diseases/ep [Epidemiology]. Carotid Intima-Media Thickness. *Cognition. *Cognition Disorders/ep [Epidemiology]. Cognition Disorders/px [Psychology]. Female. Follow-Up Studies. Humans. Linear

Models. Male. Memory. Middle Aged. Multivariate Analysis. Neuropsychological Tests. Norway/ep [Epidemiology]. Prospective Studies. Psychomotor Performance. Risk Assessment. Risk Factors. Severity of Illness Index. Time Factors. Wechsler Scales.
Year of Publication
2012

Link to the Ovid Full Text or citation:
Click here for full text options

Link to the External Link Resolver:
SFX

80.

Vascular predictors of cognitive decline in patients with mild cognitive impairment.
Viticchi G; Falsetti L; Vernieri F; Altamura C; Bartolini M; Luzzi S; Provinciali L; Silvestrini M.
Neurobiology of Aging. 33(6):1127.e1-9, 2012 Jun.
[Comparative Study. Journal Article. Research Support, Non-U.S. Gov't]
UI: 22217417
Our aim in this study was to assess the relationship between the state of cerebral vessels and the risk of conversion from mild cognitive impairment (MCI) to Alzheimer's disease (AD). We included 117 MCI patients. They underwent an ultrasonographic assessment of common carotid arteries intima-media thickness (IMT) and carotid plaque index. Cerebrovascular reactivity to hypercapnia in the middle cerebral arteries was calculated with the Breath-Holding Index (BHI). After a 12-month follow-up period, neuropsychological examinations demonstrated a progression to dementia in 21 patients. Pathological values of BHI and IMT significantly increased the risk of conversion (BHI: odds ratio, 5.80; 95% confidence interval, 1.83-18.37, $p < 0.05$; IMT: odds ratio, 3.08; 95% confidence interval, 1.02-9.33; $p < 0.05$, multinomial logistic regression analysis). Comparison between patients with all normal values and those with the simultaneous alteration of the 2 vascular indexes showed an increase in the risk of conversion from 9% to 33% (ordinal regression analysis). Our findings show that alterations of cerebral vessel functional and anatomic status increase the risk of conversion from MCI to dementia.
Copyright © 2012 IBRO. Published by Elsevier Inc. All rights reserved.

Version ID

1

Record Owner

From MEDLINE, a database of the U.S. National Library of Medicine.

Status

MEDLINE

Authors Full Name

Viticchi, Giovanna; Falsetti, Lorenzo; Vernieri, Fabrizio; Altamura, Claudia; Bartolini, Marco; Luzzi, Simona; Provinciali, Leandro; Silvestrini, Mauro.

Institution

Viticchi, Giovanna. Department of Experimental and Clinical Medicine, Marche Polytechnic University, Ancona, Italy.

MeSH Heading

Aged. Aged, 80 and over. *Alzheimer Disease/di [Diagnosis]. Alzheimer Disease/dg [Diagnostic Imaging]. Alzheimer Disease/ep [Epidemiology]. Carotid Artery, Common/dg [Diagnostic Imaging]. *Carotid Artery, Common/pa [Pathology]. Carotid Intima-Media Thickness/td [Trends]. Cognition Disorders/di [Diagnosis]. Cognition Disorders/dg [Diagnostic Imaging]. Cognition Disorders/ep [Epidemiology]. *Cognitive Dysfunction/di [Diagnosis]. Cognitive Dysfunction/dg [Diagnostic Imaging]. Cognitive Dysfunction/ep [Epidemiology]. *Disease Progression. Female. Follow-Up Studies. Humans. Male. Middle Cerebral Artery/dg [Diagnostic Imaging]. *Middle Cerebral Artery/pa [Pathology]. Predictive Value of Tests.

Year of Publication

2012

Link to the Ovid Full Text or citation:

Click here for full text options

Link to the External Link Resolver:

SFX

86.

Atherosclerosis and dementia: a cross-sectional study with pathological analysis of the carotid arteries.
Suemoto CK; Nitrini R; Grinberg LT; Ferretti RE; Farfel JM; Leite RE; Menezes PR; Fregni F; Jacob-Filho W; Pasqualucci CA; Brazilian Aging Brain Study Group.
Stroke. 42(12):3614-5, 2011 Dec.
[Journal Article. Research Support, Non-U.S. Gov't]
UI: 21940957
BACKGROUND AND PURPOSE: Previous ultrasound-based studies have shown an association between carotid artery atherosclerosis and dementia. Our aim was to investigate this association using postmortem examination.
METHODS: Postmortem morphometric measurements of carotid stenosis and intima-media thickness were performed in individuals with dementia (n=112) and control subjects (n=577). Multivariate logistic regression models were applied.
RESULTS: High-grade left internal carotid stenosis (>=70%) was associated with increased odds for dementia (OR, 2.30; 95% CI, 1.14-4.74; P=0.02). Intima-media thickness was not associated with dementia.
CONCLUSIONS: The likelihood of dementia is increased with high-grade left internal carotid artery atherosclerosis after adjusting for demographic and cardiovascular risk factors.
Version ID
1
Record Owner
From MEDLINE, a database of the U.S. National Library of Medicine.
Status
MEDLINE
Authors Full Name
Suemoto, Claudia K; Nitrini, Ricardo; Grinberg, Lea T; Ferretti, Renata E L; Farfel, Jose M; Leite, Renata E P; Menezes, Paulo R; Fregni, Felipe; Jacob-Filho, Wilson; Pasqualucci, Carlos A; Brazilian Aging Brain Study Group.
Institution
Suemoto, Claudia K. Department of Geriatrics, University of Sao Paulo Medical School, Avenida Doutor Arnaldo, 455, sala 1353, Sao Paulo, SP, Brazil. cksuemoto@usp.br
MeSH Heading
Aged. Aged, 80 and over. *Atherosclerosis/pa [Pathology]. *Carotid Arteries/pa [Pathology]. Carotid Intima-Media Thickness. *Carotid Stenosis/pa [Pathology]. Cross-Sectional Studies. *Dementia/pa [Pathology]. Female. Humans. Male. Middle Aged. Tunica Intima/pa [Pathology]. Tunica Media/pa [Pathology].
Year of Publication

2011

Link to the Ovid Full Text or citation:
Click here for full text options

Link to the External Link Resolver:
SFX

87.

Carotid atherosclerosis and cognitive function in midlife: the Beaver Dam Offspring Study.
Zhong W; Cruickshanks KJ; Huang GH; Klein BE; Klein R; Nieto FJ; Pankow JS; Schubert CR.
Atherosclerosis. 219(1):330-3, 2011 Nov.
[Journal Article. Research Support, N.I.H., Extramural]
UI: 21831374
BACKGROUND: Atherosclerosis may be associated with cognitive function; however the studies are few, especially among midlife adults.
METHODS: Participants in the beaver dam offspring study who had cognitive test data and gradable carotid artery ultrasound scans were included (n=2794, mean age: 49 years). Atherosclerosis was measured by carotid intima-media thickness (IMT) and the presence of plaque. Cognitive function was measured by the trail making test (TMT), grooved pegboard test (GPT) and mini-mental state examination (MMSE). Generalized cognitive function was defined by a summary score calculated from the TMT and GPT. Linear regression was used to evaluate the associations between carotid atherosclerosis and cognitive function tests.
RESULTS: Larger IMT was associated with lower GPT, MMSE and the summary score adjusting for multiple factors, the coefficients were: 13.8s (p<0.0001), -0.6 (p=0.007), and 0.47 (p=0.01), respectively for 1mm increase in IMT. Plaque scores were significantly associated with TMT-B, GPT, MMSE, and the summary score adjusting for age, sex and education. The associations remained statistically significant after further adjustments except for the association with TMT-B, which was attenuated and no longer significant.
CONCLUSIONS: Our results show the significant associations between markers of carotid atherosclerosis and cognitive function in a cohort of persons aged 21-84 years. Longitudinal studies are needed to further examine these associations.

Copyright A© 2011 Elsevier Ireland Ltd. All rights reserved.
Version ID
1
Record Owner
From MEDLINE, a database of the U.S. National Library of Medicine.
Status
MEDLINE
Authors Full Name
Zhong, Wenjun; Cruickshanks, Karen J; Huang, Guan-Hua; Klein, Barbara E K; Klein, Ronald; Nieto, F Javier; Pankow, James S; Schubert, Carla R.
Institution
Zhong, Wenjun. University of Wisconsin-Madison, 1036 WARF, 610 Walnut Street, Madison, WI 53726, USA. wzhong@wisc.edu
MeSH Heading
Adult. Aged. Aged, 80 and over. Carotid Artery Diseases/dg [Diagnostic Imaging]. *Carotid Artery Diseases/px [Psychology]. Carotid Intima-Media Thickness. *Cognition. Female. Humans. Male. Middle Aged. Plaque, Atherosclerotic/pa [Pathology].
Year of Publication
2011

Link to the Ovid Full Text or citation:
Click here for full text options

Link to the External Link Resolver:
SFX

96.

A review of carotid atherosclerosis and vascular cognitive decline: a new understanding of the keys to symptomology. [Review]
Dempsey RJ; Vemuganti R; Varghese T; Hermann BP.
Neurosurgery. 67(2):484-93; discussion 493-4, 2010 Aug.
[Journal Article. Research Support, N.I.H., Extramural. Review]

UI: 20644437

This review encourages the reader to consider cerebral vascular disease beyond the traditional clinical end points of major motor and speech strokes and to consider the possible impact of embolic cerebral vascular disease on vascular cognitive decline. This article examines the issue of "silent" strokes in the relationship between the structural stability of atherosclerotic carotid plaque and the development of nonmotor symptomatology, including cognitive decline. It addresses the question of the role of carotid emboli in silent stroke and their cognitive sequelae. In a study of endarterectomy patients, we relate plaque elasticity and its development of mechanical strain features and thinning of stabilizing fibrous cap at the point of these mechanical strain features. The possibility that microemboli from such mechanically unstable carotid plaques could contribute to silent strokes led to a study of cognitive function in such patients. A linear relationship between the process of mechanically unstable areas of carotid plaques and cognitive decline suggests a contributory role for such a process in silent strokes.

Version ID

1

Record Owner

From MEDLINE, a database of the U.S. National Library of Medicine.

Status

MEDLINE

Authors Full Name

Dempsey, Robert J; Vemuganti, Raghu; Varghese, Tomy; Hermann, Bruce P.

Institution

Dempsey, Robert J. University of Wisconsin School of Medicine and Public Health, Department of Neurological Surgery, Madison, Wisconsin 53792, USA. dempsey@neurosurg.wisc.edu

MeSH Heading

*Carotid Artery Diseases/co [Complications]. Carotid Artery Diseases/ge [Genetics]. *Carotid Artery Diseases/px [Psychology]. *Cerebrovascular Disorders/co [Complications]. *Cerebrovascular Disorders/px [Psychology]. *Cognition Disorders/et [Etiology]. *Cognition Disorders/px [Psychology]. Elasticity. Embolization, Therapeutic. Gene Expression. Humans. Intracranial Embolism/et [Etiology]. Intracranial Embolism/pa [Pathology]. Neovascularization, Pathologic/pa [Pathology]. Neuropsychological Tests. Plaque, Atherosclerotic/et [Etiology]. Plaque, Atherosclerotic/pa [Pathology]. Stroke/px [Psychology].

Year of Publication

2010

Link to the Ovid Full Text or citation:

Click here for full text options

Link to the External Link Resolver:
SFX

99.

Vascular characteristics of patients with dementia.
Morovic S; Jurasic MJ; Martinic Popovic I; Seric V; Lisak M; Demarin V.
Journal of the Neurological Sciences. 283(1-2):41-3, 2009 Aug 15.
[Journal Article. Research Support, Non-U.S. Gov't]
UI: 19375085
Arterial beta stiffness index is a potential risk factor for increased stroke occurrence. Vascular component appears to be significant in both Alzheimer's disease (AD) and vascular dementia (VAD). We aimed to further explore vascular characteristics of patients with both types of cognitive decline using non-invasive neurosonological methods. There were 38 patients; 16 diagnosed with AD and 22 with VAD. Vascular risk factors were assessed and ultrasound measurements on common carotid artery (CCA) were performed using Aloka ProSound ALPHA 10 with 13 MHz linear probe. Among AD patients there were 5 with arterial hypertension (AH), 3 with atrial fibrillation (AF), 2 with diabetes mellitus (DM), 6 with hyper lipidemia and 1 smoker. Nineteen VAD patients had AH, 6 had AF, 12 had hyper lipidemia and one was diabetic. We found no statistically significant differences between groups regarding average body mass index (BMI), blood pressure, pulse pressure, intima-media thickness (IMT), CCA diameter or arterial beta stiffness indices. However, the trend of BMI increase, slight blood and pulse pressure decrease, CCA diameter increase and beta stiffness index increase was noted in VAD patients. Even though there was no significant difference found among two explored subgroups of patients with dementia, there was a tendency of greater systolic and diastolic diameters noted in VAD as well as greater stiffness, especially when measured in the right CCA. This indicates that VAD patients may have more prominent vascular changes that may help differentiate the type of dementia and further monitor these individuals. Further studies on a larger number of patients are needed support this evidence.
Version ID
1
Record Owner
From MEDLINE, a database of the U.S. National Library of Medicine.

Status
MEDLINE
Authors Full Name
Morovic, Sandra; Jurasic, Miljenka-Jelena; Martinic Popovic, Irena; Seric, Vesna; Lisak, Marijana; Demarin, Vida.
Institution
Morovic, Sandra. University Department of Neurology, Sestre Milosrdnice University Hospital, Vinogradska 29, Zagreb, Croatia.
MeSH Heading
Aged. *Alzheimer Disease/dg [Diagnostic Imaging]. Alzheimer Disease/ep [Epidemiology]. Alzheimer Disease/pp [Physiopathology]. Atrial Fibrillation/ep [Epidemiology]. Blood Pressure. Body Mass Index. *Carotid Artery, Common/dg [Diagnostic Imaging]. Carotid Artery, Common/pa [Pathology]. Carotid Artery, Common/pp [Physiopathology]. *Dementia, Vascular/dg [Diagnostic Imaging]. Dementia, Vascular/ep [Epidemiology]. Dementia, Vascular/pp [Physiopathology]. Diabetes Mellitus/ep [Epidemiology]. Echoencephalography. Elasticity. Female. Humans. Hyperlipidemias/ep [Epidemiology]. Male. Organ Size. Risk Factors. Smoking/ep [Epidemiology].
Year of Publication
2009

Link to the Ovid Full Text or citation:
Click here for full text options

Link to the External Link Resolver:
SFX

100.

Carotid intima-media thickness as a predictor of response to cholinesterase inhibitors in Alzheimer's disease: an open-label trial.
Modrego PJ; Rios C; Perez Trullen JM; Garcia-Gomara MJ; Errea JM.
CNS Drugs. 23(3):253-60, 2009.
[Clinical Trial. Journal Article]
UI: 19320533

BACKGROUND: Cholinesterase inhibitors are modestly effective in treating patients with Alzheimer's disease. However, there may be important inter-individual variations ranging from no improvement at all to significant improvement and long periods of stabilization. Carotid atherosclerosis is associated with cognitive decline in elderly people.
OBJECTIVE: The objective of this study was to investigate whether carotid intima-media thickness (IMT) predicts response to cholinesterase inhibitors in Alzheimer's disease.
PATIENTS AND METHODS: A series of 54 patients with mild to moderate Alzheimer's disease were enrolled consecutively in an open-label trial. At baseline, all patients were assessed on the following clinical scales: Mini-Mental State Examination, Clinical Dementia Rating, the Hachinski Ischemic Scale, Blessed Dementia Rating Scale, Alzheimer's Disease Assessment Scale-cognitive subscale (ADAS-cog), Neuropsychiatric Inventory (NPI) and a daily-living activities scale (Disability Assessment for Dementia [DAD]). Investigations included magnetic resonance imaging of the brain and a colour echo-Doppler scan of the carotid arteries to measure the maximum IMT. Patients were then commenced on galantamine treatment for 6 months, after which scores on the ADAS-cog, NPI and DAD scales were reassessed.
RESULTS: A total of 50 patients completed the study. Their mean age was 77.78 years (SD 6.51 years); 34 patients were female. Galantamine treatment decreased the mean NPI score from 17.68 to 13.86 points, but this difference was not statistically significant (p=0.07). On the ADAS-cog scale, a modest and nonsignificant mean difference of -0.4 points (p=0.7) was observed. A weak (correlation coefficient r=0.4) but significant correlation between IMT and changes in clinical scale score was found, with low carotid IMT being shown to be a predictor of response on both the ADAS-cog (p=0.003) and NPI (p=0.006) scales; these findings were corroborated in multivariate analysis. For men, the correlation was stronger (r=0.7 and 0.8 for the ADAS-cog and NPI scales, respectively).
CONCLUSION: Although the magnitude of effect was moderate, carotid IMT could be a significant predictor of clinical response to cholinesterase inhibitors in patients with Alzheimer's disease.
Version ID
1
Record Owner
From MEDLINE, a database of the U.S. National Library of Medicine.
Status
MEDLINE
Authors Full Name
Modrego, Pedro J; Rios, Consuelo; Perez Trullen, Jose M; Garcia-Gomara, Maria J; Errea, Jose M.
Institution
Modrego, Pedro J. Department of Neurology, Hospital Universitario Miguel Servet, Zaragoza, Spain. pmodrego@salud.aragon.es

Comments
Comment in (CIN)
MeSH Heading
Activities of Daily Living. Aged. *Alzheimer Disease/dt [Drug Therapy]. Alzheimer Disease/pa [Pathology]. Alzheimer Disease/px [Psychology]. Atherosclerosis/co [Complications]. Atherosclerosis/dg [Diagnostic Imaging]. Atherosclerosis/pa [Pathology]. Carotid Artery Diseases/co [Complications]. *Carotid Artery Diseases/dg [Diagnostic Imaging]. *Carotid Artery Diseases/pa [Pathology]. *Cholinesterase Inhibitors/tu [Therapeutic Use]. Female. *Galantamine/tu [Therapeutic Use]. Humans. Magnetic Resonance Imaging. Male. Middle Aged. Neuropsychological Tests. Predictive Value of Tests. Psychiatric Status Rating Scales. Regression Analysis. Ultrasonography.
Registry Number/Name of Substance
0 (Cholinesterase Inhibitors). 0D3Q044KCA (Galantamine).
Year of Publication
2009

Link to the Ovid Full Text or citation:
Click here for full text options

Link to the External Link Resolver:
SFX

102.

Increased atherogenic lipoproteins are associated with cognitive impairment: effects of statins and subclinical atherosclerosis.
Carlsson CM; Nondahl DM; Klein BE; McBride PE; Sager MA; Schubert CR; Klein R; Cruickshanks KJ.
Alzheimer Disease & Associated Disorders. 23(1):11-7, 2009 Jan-Mar.
[Journal Article. Research Support, N.I.H., Extramural. Research Support, Non-U.S. Gov't]
UI: 19266697
Hypercholesterolemia increases the risk for dementia. Some studies suggest that statins may protect cognition, but findings are conflicting. Unmeasured confounders, including high-density lipoprotein (HDL) cholesterol or subclinical atherosclerosis, may have influenced prior study

outcomes. In older adults participating in a population-based cohort study (n=1711, aged 65 to 97 y), we investigated the relationships of total and HDL cholesterol levels, statin use, and carotid intima-media thickness with the prevalence of cognitive impairment. In adjusted models, participants in the highest quartile of non-HDL (total-HDL) cholesterol had an increased odds of cognitive impairment compared with those in the lowest quartile [odds ratio (OR): 2.06, 95% confidence interval (CI): 1.07-3.98]. Statin use was associated with lower odds of cognitive impairment in unadjusted models (OR: 0.57, 95% CI: 0.36-0.89), but this relationship was not significant after adjusting for vascular and lifestyle factors (OR: 0.84, 95% CI: 0.47-1.49). In this analysis of older adults, increased atherogenic lipoproteins were associated with impaired cognition. Statin use was related to many factors that both negatively and positively affect cognition, but was not associated with better cognitive function. These results suggest that confounding by indication may explain the contradictory findings in studies assessing the association of statins with cognition. Randomized-controlled clinical trials and longitudinal studies are necessary to determine if statins protect against cognitive decline.

Version ID

1

Record Owner

From MEDLINE, a database of the U.S. National Library of Medicine.

Status

MEDLINE

Authors Full Name

Carlsson, Cynthia M; Nondahl, David M; Klein, Barbara E K; McBride, Patrick E; Sager, Mark A; Schubert, Carla R; Klein, Ronald; Cruickshanks, Karen J.

Institution

Carlsson, Cynthia M. Section of Geriatrics and Gerontology, Department of Medicine, University of Wisconsin School of Medicine and Public Health, Madison, WI53705, USA. cmc@medicine.wisc.edu

Comments

Comment in (CIN)

MeSH Heading

Aged. Aged, 80 and over. *Atherosclerosis/co [Complications]. *Carotid Arteries/pa [Pathology]. *Cholesterol/bl [Blood]. *Cholesterol, HDL/bl [Blood]. Cognition Disorders/bl [Blood]. *Cognition Disorders/et [Etiology]. Female. Humans. *Hydroxymethylglutaryl-CoA Reductase Inhibitors/tu [Therapeutic Use]. Hypercholesterolemia/dt [Drug Therapy]. Male. Tunica Intima/pa [Pathology]. Tunica Media/pa [Pathology].

Registry Number/Name of Substance

0 (Cholesterol, HDL). 0 (Hydroxymethylglutaryl-CoA Reductase Inhibitors). 97C5T2UQ7J (Cholesterol).

Year of Publication
2009

Link to the Ovid Full Text or citation:
Click here for full text options

Link to the External Link Resolver:
SFX

103.

Vascular structure and function is correlated to cognitive performance and white matter hyperintensities in older hypertensive patients with subjective memory complaints.
Kearney-Schwartz A; Rossignol P; Bracard S; Felblinger J; Fay R; Boivin JM; Lecompte T; Lacolley P; Benetos A; Zannad F.
Stroke. 40(4):1229-36, 2009 Apr.
[Journal Article. Research Support, Non-U.S. Gov't]
UI: 19246701
BACKGROUND AND PURPOSE: Arterial stiffening and thickening and endothelial dysfunction may be associated with cognitive decline or white matter hyperintensities (WMH) independently of blood pressure level. We aimed to investigate, using an integrative approach, the relative contributions of structural and functional vascular factors to the degree of cognitive impairment (primary outcome) and the severity of WMH (secondary outcome) in elderly hypertensive patients with subjective memory complaints, a group prone to dementia.
METHODS: A prospective, dedicated, cross-sectional population of 198 elderly hypertensive patients (mean age 69.3+/-6.2 years) with subjective memory complaints underwent a full set of cognitive function assessments, brain MRI with semiquantification of WMH, carotid ultrasonography, carotid-femoral pulse wave velocity, brachial endothelial function, and plasma von Willebrand Factor measurements.
RESULTS: After adjustment for the usual cardiovascular risk factors, increased arterial stiffness (as assessed by pulse wave velocity) was significantly and independently associated with memory impairment in men. The severity of WMH was independently associated with increased carotid

intima media thickness and stiffness (as assessed by augmentation index) as well as with increased age and plasma levels of von Willebrand Factor, a biomarker of endothelial dysfunction. CONCLUSIONS: Our data suggest that vascular abnormalities, independently of blood pressure levels, may play a role in the setting of subjective memory complaints as well as of WMH in elderly hypertensive patients. Arterial thickness and stiffness as well as endothelial function should be assessed simultaneously and may represent additional targets for the prevention of subjective memory complaints and WMH.

Version ID

1

Record Owner

From MEDLINE, a database of the U.S. National Library of Medicine.

Status

MEDLINE

Authors Full Name

Kearney-Schwartz, Anna; Rossignol, Patrick; Bracard, Serge; Felblinger, Jacques; Fay, Renaud; Boivin, Jean-Marc; Lecompte, Thomas; Lacolley, Patrick; Benetos, Athanase; Zannad, Faiez.

Institution

Kearney-Schwartz, Anna. Nancy University Hospital, Clinical Investigation Centre, J d'Arc Hospital, Dommartin les Toul, France.

Comments

Comment in (CIN)

MeSH Heading

Aged. Aged, 80 and over. Carotid Artery Diseases/pa [Pathology]. Cerebrovascular Circulation. Cognition Disorders/ep [Epidemiology]. Cognition Disorders/pa [Pathology]. Cognition Disorders/pp [Physiopathology]. Cross-Sectional Studies. Endothelium, Vascular/pa [Pathology]. Endothelium, Vascular/ph [Physiology]. Female. Humans. *Hypertension/ep [Epidemiology]. *Hypertension/pa [Pathology]. Hypertension/pp [Physiopathology]. *Leukoaraiosis/ep [Epidemiology]. *Leukoaraiosis/pa [Pathology]. Leukoaraiosis/pp [Physiopathology]. Magnetic Resonance Imaging. Male. *Memory Disorders/ep [Epidemiology]. *Memory Disorders/pa [Pathology]. Memory Disorders/pp [Physiopathology]. Middle Aged. Nerve Fibers, Myelinated/pa [Pathology]. Prevalence. Prospective Studies. Pulsatile Flow. Risk Factors. Severity of Illness Index.

Year of Publication

2009

Link to the Ovid Full Text or citation:

Click here for full text options

Link to the External Link Resolver:
SFX

104.

Subclinical atherosclerosis is weakly associated with lower cognitive function in healthy hyperhomocysteinemic adults without clinical cardiovascular disease.
Gatto NM; Henderson VW; St John JA; McCleary C; Detrano R; Hodis HN; Mack WJ.
International Journal of Geriatric Psychiatry. 24(4):390-9, 2009 Apr.
[Journal Article. Randomized Controlled Trial. Research Support, N.I.H., Extramural]
UI: 18836986
OBJECTIVE: Atherosclerosis is the most common pathologic process underlying cardiovascular disease (CVD). It is not well known whether subclinical atherosclerosis is an independent risk factor for lower cognitive function among individuals without clinically evident CVD.
METHODS: We examined cross-sectional associations between subclinical atherosclerosis and cognitive function in a community-based sample of otherwise healthy adults with plasma homocysteine >or=8.5 micromol/L enrolled in the BVAIT study (n = 504, mean age 61 years). Carotid artery intima-media thickness (CIMT), coronary artery calcium (CAC) and abdominal aortic calcium (AAC) were used to measure subclinical atherosclerosis. Cognitive function was assessed with a battery of neuropsychological tests. A principal components analysis was used to extract five uncorrelated cognitive factors from scores on individual tests, and a measure of global cognition was derived. Multivariable linear regression was used to examine the association between subclinical atherosclerosis and cognitive function, adjusting for other correlates of cognition.
RESULTS: Increasing thickness of CIMT was associated with significantly lower scores on the verbal learning factor (beta = -0.07 per 0.1 mm increase CIMT [SE(beta) = 0.03], p = 0.01). CAC and AAC were not individually associated with any of the cognitive factors.
CONCLUSIONS: This study provides evidence that increasing CIMT is weakly associated with lower verbal learning abilities but not global cognition in a population of otherwise healthy middle-to-older aged adults with elevated plasma homocysteine levels but without clinically evident CVD. The association between CIMT and poor verbal learning may pertain particularly to men.
Copyright (c) 2008 John Wiley & Sons, Ltd.
Version ID
1

Record Owner

From MEDLINE, a database of the U.S. National Library of Medicine.

Status

MEDLINE

Authors Full Name

Gatto, Nicole M; Henderson, Victor W; St John, Jan A; McCleary, Carol; Detrano, Robert; Hodis, Howard N; Mack, Wendy J.

Institution

Gatto, Nicole M. Department of Preventive Medicine, USC Keck School of Medicine, Los Angeles, CA 90089-9010, USA.

MeSH Heading

Adult. Aged. Aged, 80 and over. *Atherosclerosis/co [Complications]. Atherosclerosis/dg [Diagnostic Imaging]. Atherosclerosis/pa [Pathology]. Carotid Arteries/dg [Diagnostic Imaging]. Carotid Arteries/pa [Pathology]. Carotid Artery Diseases/co [Complications]. Carotid Artery Diseases/pa [Pathology]. Cognition Disorders/dg [Diagnostic Imaging]. *Cognition Disorders/et [Etiology]. Cross-Sectional Studies. Female. Humans. *Hyperhomocysteinemia/co [Complications]. Hyperhomocysteinemia/dg [Diagnostic Imaging]. Hyperhomocysteinemia/pa [Pathology]. Male. Middle Aged. Neuropsychological Tests. Risk Factors. Tomography, X-Ray Computed.

Year of Publication

2009

Link to the Ovid Full Text or citation:

Click here for full text options

Link to the External Link Resolver:

SFX

105.

Carotid atherosclerosis and cognitive decline in patients with Alzheimer's disease.

Silvestrini M; Gobbi B; Pasqualetti P; Bartolini M; Baruffaldi R; Lanciotti C; Cerqua R; Altamura C; Provinciali L; Vernieri F.

Neurobiology of Aging. 30(8):1177-83, 2009 Aug.

[Journal Article. Research Support, Non-U.S. Gov't]
UI: 18077061
Aim of the study was to explore the correlation between the progression of carotid atherosclerosis and the evolution of cognitive impairment in 66 patients with Alzheimer's disease (AD). They underwent cognitive status evaluation and ultrasonography (US) to investigate carotid arteries intima-media thickness (IMT) and plaque index (PI). After a 12-month follow-up period, neuropsychological and US examinations were repeated to assess the progression of carotid atherosclerosis and of cognitive decline [in terms of changes in Mini Mental State Examination (MMSE) scores]. MMSE score changes were related to baseline IMT (p=0.018), changes in IMT (p<0.001) and PI (p=0.006), and "antihypertensive drug intake" (p<0.001). While the first three variables correlated with increased cognitive impairment, the last one was associated with a reduced extent of MMSE score decline. Results show a link between progression of carotid wall changes and of cognitive decline, and suggest a possible protective role of antihypertensive therapy. Given the potential clinical implications, our preliminary findings could stimulate further investigations into the role of vascular impairment in patients with AD.
Version ID
1
Record Owner
From MEDLINE, a database of the U.S. National Library of Medicine.
Status
MEDLINE
Authors Full Name
Silvestrini, Mauro; Gobbi, Beatrice; Pasqualetti, Patrizio; Bartolini, Marco; Baruffaldi, Roberto; Lanciotti, Chiara; Cerqua, Raffaella; Altamura, Claudia; Provinciali, Leandro; Vernieri, Fabrizio.
Institution
Silvestrini, Mauro. Neurological Clinic, Polytechnic University of Marche, Ancona, Italy. m.silvestrini@univpm.it
MeSH Heading
Aged. *Alzheimer Disease/co [Complications]. Alzheimer Disease/dt [Drug Therapy]. Antihypertensive Agents/tu [Therapeutic Use]. Carotid Arteries/dg [Diagnostic Imaging]. Carotid Arteries/de [Drug Effects]. *Carotid Artery Diseases/co [Complications]. Carotid Artery Diseases/dg [Diagnostic Imaging]. Carotid Artery Diseases/dt [Drug Therapy]. Cognition/de [Drug Effects]. *Cognition Disorders/co [Complications]. Cognition Disorders/dt [Drug Therapy]. Disease Progression. Female. Follow-Up Studies. Humans. Linear Models. Male. Neuroprotective Agents/tu [Therapeutic Use]. Severity of Illness Index. Ultrasonography.
Registry Number/Name of Substance
0 (Antihypertensive Agents). 0 (Neuroprotective Agents).

Year of Publication
2009

Link to the Ovid Full Text or citation:
Click here for full text options

Link to the External Link Resolver:
SFX

107.

Socioeconomic status moderates the association between carotid intima-media thickness and cognition in midlife: evidence from the Whitehall II study.
Singh-Manoux A; Britton A; Kivimaki M; Gueguen A; Halcox J; Marmot M.
Atherosclerosis. 197(2):541-8, 2008 Apr.
[Journal Article. Research Support, N.I.H., Extramural. Research Support, Non-U.S. Gov't]
UI: 17854813
BACKGROUND: Common carotid artery intima-media thickness (IMT) is a measure of generalized atherosclerosis and has been shown to be associated with cognitive function. We examine two questions: does socioeconomic status (SES) moderate this association and is IMT more strongly associated with specific aspects of cognitive function?
METHODS: Data are drawn from the Phase 7 (2003-2004) of the Whitehall II study (N=3896). In cross-sectional analyses the association between IMT and six measures of cognition (short-term verbal memory, inductive reasoning, vocabulary, semantic and phonemic fluency and a measure of global cognitive status) was examined in analyses adjusted for previous history of coronary heart disease, health behaviours and other vascular risk measures such as blood pressure, cholesterol and body mass index.
RESULTS: The overall association between IMT and the six measures of cognition was restricted to the low SES group (p=0.02). Within this group, IMT was significantly associated with inductive reasoning (p=0.001), vocabulary (p=0.002), phonemic (p=0.006) and semantic fluency (p=0.02). The covariates examined explained about a quarter of the association between IMT and cognition in the low SES group. The associations with the measure of inductive reasoning (p=0.02), vocabulary (p=0.02) and phonemic fluency (p=0.04) remained after adjustment for all covariates.

CONCLUSIONS: SES is an important modifier of the association between IMT and cognition, an inverse association between the two was observed only in the low SES group. It is possible that high cognitive reserve among the high SES individuals prevents the functional manifestations of atherosclerosis. Verbal memory was not one of the cognitive domains associated with IMT.

Version ID

1

Record Owner

From MEDLINE, a database of the U.S. National Library of Medicine.

Status

MEDLINE

Authors Full Name

Singh-Manoux, Archana; Britton, Annie; Kivimaki, Mika; Gueguen, Alice; Halcox, Julian; Marmot, Michael.

Institution

Singh-Manoux, Archana. INSERM, U687-IFR69, HNSM, 14 rue du Val d'Osne, 94415 Saint-Maurice Cedex, France. Archana.Singh-Manoux@st-maurice.inserm.fr

MeSH Heading

Aged. *Atherosclerosis/co [Complications]. Atherosclerosis/ec [Economics]. Atherosclerosis/pa [Pathology]. *Carotid Artery Diseases/co [Complications]. Carotid Artery Diseases/ec [Economics]. Carotid Artery Diseases/pa [Pathology]. *Carotid Artery, Common/pa [Pathology]. *Cognition. *Cognition Disorders/pp [Physiopathology]. Cross-Sectional Studies. Female. Health Surveys. Humans. Male. Middle Aged. *Social Class. Tunica Intima/pa [Pathology]. Tunica Media/pa [Pathology].

Year of Publication

2008

Link to the Ovid Full Text or citation:

Click here for full text options

Link to the External Link Resolver:

SFX

108.

Carotid intima-media thickness and cognitive function in elderly women: a population-based study.
Komulainen P; Kivipelto M; Lakka TA; Hassinen M; Helkala EL; Patja K; Nissinen A; Rauramaa R.
Neuroepidemiology. 28(4):207-13, 2007.
[Comparative Study. Journal Article. Research Support, Non-U.S. Gov't]
UI: 17851259
OBJECTIVE: Several vascular risk factors have been linked to cognitive decline. However, little is known about the association between the atherosclerotic process and cognitive impairment. We investigated whether carotid intima-media thickness (IMT) predicts the risk of cognitive impairment and whether the putative impairment is specific for some cognitive domains.
METHODS: A 12-year population-based follow-up study was performed for a total of 91 women, aged 60-70 years at baseline. Ultrasonographically assessed carotid artery IMT and the Mini-Mental State Examination test were performed at baseline and 12-year follow-up. A detailed cognitive evaluation for memory and cognitive speed was performed in 2003. The mean of left and right carotid bifurcation IMT was used in the analyses for association with the risk for poor cognitive speed and memory.
RESULTS: Increased IMT at baseline was an independent predictor for poor memory (beta = -5.004, 95% confidence interval = -7.74 to -2.27; p = 0.001) and cognitive speed (beta = 2.562, 95% confidence interval = 1.19-4.94; p = 0.035) at 12-year follow-up after adjustment for age, education, depression, plasma LDL cholesterol, systolic blood pressure, cardiovascular disease, hormone replacement therapy, smoking, alcohol consumption and physical activity. The risk for poor memory (p = 0.023 for linear trend) and cognitive speed (p = 0.070 for linear trend) increased with increasing IMT tertiles.
CONCLUSIONS: Carotid IMT predicts an increased risk for cognitive impairment, particularly poor memory and cognitive speed, in elderly women.
Copyright (c) 2007 S. Karger AG, Basel.
Version ID
1
Record Owner
From MEDLINE, a database of the U.S. National Library of Medicine.
Status
MEDLINE
Authors Full Name
Komulainen, Pirjo; Kivipelto, Miia; Lakka, Timo A; Hassinen, Maija; Helkala, Eeva-Liisa; Patja, Kristiina; Nissinen, Aulikki; Rauramaa, Rainer.
Institution

Komulainen, Pirjo. Kuopio Research Institute of Exercise Medicine, Kuopio, Finland.
pirjo.komulainen@uku.fi
MeSH Heading
Aged. Aged, 80 and over. Atherosclerosis/co [Complications]. Atherosclerosis/pa [Pathology]. *Carotid Artery, Common/pa [Pathology]. Cognition/ph [Physiology]. *Cognition Disorders/et [Etiology]. *Cognition Disorders/pa [Pathology]. Female. Follow-Up Studies. Humans. Middle Aged. Risk Factors. *Tunica Intima/pa [Pathology]. *Tunica Media/pa [Pathology].
Year of Publication
2007

Link to the Ovid Full Text or citation:
[Click here for full text options]

Link to the External Link Resolver:
[SFX]

109.

Cerebrovascular risk factors, vascular disease, and neuropsychological outcomes in adults with major depression.
Smith PJ; Blumenthal JA; Babyak MA; Hoffman BM; Doraiswamy PM; Waugh R; Hinderliter A; Sherwood A.
Psychosomatic Medicine. 69(6):578-86, 2007 Jul-Aug.
[Journal Article. Research Support, N.I.H., Extramural]
UI: 17634564
OBJECTIVE: To investigate the relationship of cerebrovascular risk factors (CVRFs), endothelial function, carotid artery intima medial thickness (IMT), and neuropsychological performance in a sample of 198 middle-aged and older individuals with major depressive disorder (MDD). Neuropsychological deficits are common among adults with MDD, particularly among those with CVRFs and potentially persons with subclinical vascular disease.
METHODS: CVRFs were indexed by the Framingham Stroke Risk Profile (FSRP) and serum cholesterol levels obtained by medical history and physical examination. Patients completed a neuropsychological test battery including measures of executive functioning, working memory,

and verbal recall. Vascular function was indexed by carotid artery IMT and brachial artery flow mediated dilation (FMD). Hierarchical multiple regression analyses were used to investigate the association between CVRFs, vascular disease, and neurocognitive performance.
RESULTS: Greater FSRP scores were associated with poorer executive functioning (b = -0.86; p = .041) and working memory (b = -0.90; p = .024). Lower high-density lipoprotein levels also were associated with poorer executive functioning (b = 1.03; p = .035). Higher IMT (b = -0.83; p = .028) and lower FMD (b = 1.29; p = .032) were associated with poorer executive functioning after controlling for CVRFs. Lower FMD was also associated with poorer working memory (b = 1.58; p = .015).
CONCLUSIONS: Greater CVRFs were associated with poorer neuropsychological performance. Vascular dysfunction also was associated with neuropsychological decrements independent of traditional CVRFs.
Version ID
1
Record Owner
From MEDLINE, a database of the U.S. National Library of Medicine.
Status
MEDLINE
Authors Full Name
Smith, Patrick J; Blumenthal, James A; Babyak, Michael A; Hoffman, Benson M; Doraiswamy, P Murali; Waugh, Robert; Hinderliter, Alan; Sherwood, Andrew.
Institution
Smith, Patrick J. Department of Psychiatry and Behavioral Sciences, Duke University Medical Center, Durham, NC 27710, USA. Smith562@mc.duke.edu
MeSH Heading
Atherosclerosis/ep [Epidemiology]. *Atherosclerosis/pp [Physiopathology]. Cerebrovascular Disorders/ep [Epidemiology]. *Cerebrovascular Disorders/pp [Physiopathology]. *Cognition. Cross-Sectional Studies. Depressive Disorder, Major/ep [Epidemiology]. *Depressive Disorder, Major/pp [Physiopathology]. *Endothelium, Vascular/pp [Physiopathology]. Factor Analysis, Statistical. Female. Humans. Male. Middle Aged. Multivariate Analysis. Regression Analysis. Risk Factors. United States/ep [Epidemiology].
Year of Publication
2007

Link to the Ovid Full Text or citation:
Click here for full text options

Link to the External Link Resolver:
SFX

111.

Atherosclerosis and risk for dementia.
van Oijen M; de Jong FJ; Witteman JC; Hofman A; Koudstaal PJ; Breteler MM.
Annals of Neurology. 61(5):403-10, 2007 May.
[Journal Article. Research Support, Non-U.S. Gov't]
UI: 17328068
OBJECTIVE: Atherosclerosis has been implicated in the development of dementia and its major subtypes, Alzheimer's disease and vascular dementia. However, support for this association mainly comes from cross-sectional studies. We investigated the association of atherosclerosis with dementia and subtypes of dementia during long follow-up, with various noninvasive measures of atherosclerosis.
METHODS: This study was based on 6,647 participants in the Rotterdam Study, a population-based prospective cohort study among 7,983 elderly subjects. At baseline (1990-1993) and at the third survey (1997-1999), common carotid intima media thickness, carotid plaques, and peripheral arterial disease (measured as ankle-brachial index) were measured. During follow-up (mean, 9.0 years), 678 subjects developed dementia. We estimated the associations of different measures of atherosclerosis with risk for dementia and subtypes of dementia by means of Cox proportional hazard models. Analyses were repeated and stratified on duration of follow-up. To evaluate competing risk for mortality, we examined the association between measures of atherosclerosis and risk for dementia or mortality by combining the two in a single outcome measure.
RESULTS: We found that atherosclerosis, predominantly carotid atherosclerosis, was associated with an increased risk for dementia during short follow-up. This association attenuated with longer follow-up, likely because of the strong association between atherosclerosis and mortality. The associations did not differ across apolipoprotein E genotypes.
INTERPRETATION: Our findings suggest that atherosclerosis is associated with an increased risk for dementia. Stronger associations between atherosclerosis and mortality may attenuate the association between atherosclerosis and dementia in prospective cohort studies with long follow-up periods.
Version ID

1

Record Owner

From MEDLINE, a database of the U.S. National Library of Medicine.

Status

MEDLINE

Authors Full Name

van Oijen, Marieke; de Jong, Frank Jan; Witteman, Jacqueline C M; Hofman, Albert; Koudstaal, Peter J; Breteler, Monique M B.

Institution

van Oijen, Marieke. Department of Epidemiology and Biostatistics, Erasmus Medical Center, Rotterdam, the Netherlands.

Comments

Comment in (CIN) Comment in (CIN)

MeSH Heading

Aged. Apolipoproteins E/ge [Genetics]. *Atherosclerosis/co [Complications]. Atherosclerosis/dg [Diagnostic Imaging]. *Atherosclerosis/ep [Epidemiology]. Carotid Arteries/dg [Diagnostic Imaging]. Cohort Studies. Data Interpretation, Statistical. Dementia/di [Diagnosis]. *Dementia/ep [Epidemiology]. Dementia/px [Psychology]. Female. Follow-Up Studies. Genotype. Humans. Male. Netherlands/ep [Epidemiology]. Neuropsychological Tests. Population. Prospective Studies. Risk. Risk Factors. Ultrasonography.

Registry Number/Name of Substance

0 (Apolipoproteins E).

Year of Publication

2007

Link to the Ovid Full Text or citation:

Click here for full text options

Link to the External Link Resolver:

SFX

112.

Carotid artery intima-media thickness and cognition in cardiovascular disease.
Haley AP; Forman DE; Poppas A; Hoth KF; Gunstad J; Jefferson AL; Paul RH; Ler AS; Sweet LH; Cohen RA.
International Journal of Cardiology. 121(2):148-54, 2007 Oct 01.
[Comparative Study. Journal Article. Research Support, N.I.H., Extramural]
UI: 17196687
BACKGROUND: Increased carotid artery intima-media thickness (IMT) is a non-invasive marker of systemic arterial disease. Increased IMT has been associated with atherosclerosis, abnormal arterial mechanics, myocardial infarction, and stroke. Given evidence of a relationship between cardiovascular health and attention-executive-psychomotor functioning, the purpose of this study was to examine IMT in relation to neuropsychological test performance in patients with a variety of cardiovascular diagnoses.
METHODS: One hundred and nine participants, ages 55 to 85, underwent neuropsychological assessment and B-mode ultrasound of the left common carotid artery. IMT was calculated using an automated algorithm based on a validated edge-detection technique. The relationship between IMT and measures of language, memory, visual-spatial abilities and attention-executive-psychomotor functioning was modeled using hierarchical linear regression analyses adjusted for age, education, sex, cardiovascular risk, current systolic blood pressure, and history of coronary artery disease (CAD).
RESULTS: Increased IMT was associated with significantly lower performance in the attention-executive-psychomotor domain (IMT beta=-0.26, p<.01), independent of age, education, sex, cardiovascular risk, current systolic blood pressure, and CAD (F(10,100)=3.61, p<.001). IMT was not significantly related to language, memory, or visual-spatial abilities.
CONCLUSIONS: Our findings suggest that, in patients with cardiovascular disease, IMT may be associated with the integrity of frontal subcortical networks responsible for attention-executive-psychomotor performance. Future studies are needed to clarify the mechanisms by which IMT affects cognition and examine potential interactions between increased IMT and other measures of cardiovascular health such as blood pressure variability, cardiac systolic performance, and systemic perfusion.
Version ID
1
Record Owner
From MEDLINE, a database of the U.S. National Library of Medicine.
Status
MEDLINE
Authors Full Name

Haley, Andreana P; Forman, Daniel E; Poppas, Athena; Hoth, Karin F; Gunstad, John; Jefferson, Angela L; Paul, Robert H; Ler, Albert S H; Sweet, Lawrence H; Cohen, Ronald A.
Institution
Haley, Andreana P. Department of Psychiatry and Human Behavior, Brown Medical School, Providence, RI, United States. aphaley@brown.edu <aphaley@brown.edu>
MeSH Heading
Aged. Aged, 80 and over. Cardiovascular Diseases/co [Complications]. *Cardiovascular Diseases/pa [Pathology]. Cardiovascular Diseases/px [Psychology]. Carotid Artery Diseases/co [Complications]. *Carotid Artery Diseases/pa [Pathology]. Carotid Artery Diseases/px [Psychology]. *Carotid Artery, Common/pa [Pathology]. *Cognition. Cognition Disorders/et [Etiology]. Cognition Disorders/pa [Pathology]. Cognition Disorders/px [Psychology]. Female. Humans. Male. Middle Aged. Neuropsychological Tests. *Tunica Intima/pa [Pathology].
Year of Publication
2007

Link to the Ovid Full Text or citation:
[Click here for full text options]

Link to the External Link Resolver:
[SFX]

114.

Cognitive impairment and carotid atherosclerosis in a general Italian midlife and old population.
Prati P; Casaroli M; Bignamini A; Scotti S; Canciani L; Ruscio M; Balestrieri M; Bornstein N; Zanetti O; Tosetto A; Castellani S; Pantoni L; Touboul PJ; Inzitari D.
Neuroepidemiology. 27(1):33-8, 2006.
[Journal Article. Research Support, Non-U.S. Gov't]
UI: 16804332
The authors describe the design and the general, ultrasonographic, neuropsychological methodology of an observational epidemiological population survey, named REMEMBER (Registry Evaluation Memory in Buttrio e Remanzacco) conducted in the northeast of Italy in a randomized stratified sample of 1,026 subjects (554 F and 472 M) aged 55-98 years. The study was planned as

cross-sectional and longitudinal survey of cognitive impairment, cardiovascular risk factors, carotid atherosclerosis in a midlife and older Italian population sample. The objectives of the first phase are to assess the prevalence of the different types of dementia, the cognitive impairment non-dementia, the cardiovascular risk factors, the carotid intima-media thickness and arterial distensibility, and of depression. The conclusions of this study will make it possible to organize preventive and interventional strategies for these epidemic conditions.

Version ID

1

Record Owner

From MEDLINE, a database of the U.S. National Library of Medicine.

Status

MEDLINE

Authors Full Name

Prati, P; Casaroli, M; Bignamini, A; Scotti, S; Canciani, L; Ruscio, M; Balestrieri, M; Bornstein, N; Zanetti, O; Tosetto, A; Castellani, S; Pantoni, L; Touboul, P J; Inzitari, D.

Institution

Prati, P. Department of Neurology, Gervasutta Hospital, Udine, Italy. patrizio.prati@tin.it

MeSH Heading

Aged. Aged, 80 and over. *Alzheimer Disease/ep [Epidemiology]. Carotid Artery Diseases/dg [Diagnostic Imaging]. *Carotid Artery Diseases/ep [Epidemiology]. *Cognition Disorders/ep [Epidemiology]. Female. Humans. Incidence. Italy/ep [Epidemiology]. Male. Mass Screening/mt [Methods]. Middle Aged. Prevalence. Risk Factors. Surveys and Questionnaires. Ultrasonography.

Year of Publication

2006

Link to the Ovid Full Text or citation:

[Click here for full text options]

Link to the External Link Resolver:

[SFX]

119.

Carotid intima-media thickness and cognitive decline: what does it mean for prevention of dementia?.
Spence JD.
Journal of the Neurological Sciences. 223(2):103-5, 2004 Aug 30.
[Comment. Editorial]
UI: 15337609
Version ID
1
Record Owner
From MEDLINE, a database of the U.S. National Library of Medicine.
Status
MEDLINE
Authors Full Name
Spence, J David.
Comments
Comment on (CON)
MeSH Heading
Aged. Aged, 80 and over. Angiotensin-Converting Enzyme Inhibitors/tu [Therapeutic Use]. Carotid Artery, Common/pa [Pathology]. Cognition Disorders/dt [Drug Therapy]. *Cognition Disorders/pa [Pathology]. Dementia/co [Complications]. *Dementia/pa [Pathology]. *Dementia/pc [Prevention & Control]. Humans. Tunica Intima/de [Drug Effects]. *Tunica Intima/pa [Pathology]. Tunica Media/de [Drug Effects]. *Tunica Media/pa [Pathology].
Registry Number/Name of Substance
0 (Angiotensin-Converting Enzyme Inhibitors).
Year of Publication
2004

Link to the Ovid Full Text or citation:
Click here for full text options

Link to the External Link Resolver:
SFX

121.

Small dense low-density lipoprotein and carotid atherosclerosis in relation to vascular dementia.
Watanabe T; Koba S; Kawamura M; Itokawa M; Idei T; Nakagawa Y; Iguchi T; Katagiri T.
Metabolism: Clinical & Experimental. 53(4):476-82, 2004 Apr.
[Comparative Study. Journal Article]
UI: 15045695
Vascular dementia (VaD) and Alzheimer's disease (AD) are the most common causes of dementia in the elderly. The aim of this study was to investigate carotid atherosclerosis, serum lipid profiles, and atherogenic hormone levels in nondiabetic Japanese men with VaD or AD. Carotid artery intima-media thickness (IMT) and plaque, serum lipid and lipoprotein profiles, including low-density lipoprotein (LDL) particle size, as well as insulin-like growth factor-I (IGF-I, somatomedin C) and testosterone levels, were determined in 34 patients with AD, 37 patients with VaD, and 63 healthy male controls. Age, body mass index, systolic and diastolic blood pressure, and fasting plasma glucose, hemoglobin A(1c) (HbA(1c)), triglyceride, high-density lipoprotein (HDL)-cholesterol, and apolipoproteins (apo) A-I, B, and E levels did not differ significantly among the 3 groups. However, the mean value of carotid IMT, the frequency of atherosclerotic plaque deposition, the serum levels of LDL-cholesterol, lipoprotein(a), and lipid peroxides, and the incidence of small dense LDL (particle diameter </= 25.5 nm) were increased significantly in VaD patients compared with AD patients or controls. VaD patients had a close reverse correlation between carotid IMT and LDL particle diameter, which were statistically proven independent risk factors for VaD. In contrast, AD patients had significantly lower serum levels of IGF-I and testosterone than either VaD patients or controls. Our results indicate that VaD is associated with atherogenic dyslipidemia, in particular, small dense LDL and carotid atherosclerosis, whereas AD is associated with hyposomatomedinemia and hypogonadism rather than atherosclerosis.
Version ID
1
Record Owner
From MEDLINE, a database of the U.S. National Library of Medicine.
Status
MEDLINE
Authors Full Name
Watanabe, Takuya; Koba, Shinji; Kawamura, Mitsuharu; Itokawa, Masashi; Idei, Tsunenori; Nakagawa, Yukiko; Iguchi, Takashi; Katagiri, Takashi.
Institution
Watanabe, Takuya. Third Department of Internal Medicine, Showa University School of Medicine, Division of Internal Medicine, Showa University Karasuyama Hospital, Tokyo, Japan.

MeSH Heading
Aged. Aged, 80 and over. Alzheimer Disease/bl [Blood]. Alzheimer Disease/me [Metabolism]. *Carotid Artery Diseases/bl [Blood]. Carotid Artery Diseases/dg [Diagnostic Imaging]. *Dementia, Vascular/bl [Blood]. Dementia, Vascular/ep [Epidemiology]. Dementia, Vascular/me [Metabolism]. Humans. Insulin-Like Growth Factor I/me [Metabolism]. Lipid Peroxides/bl [Blood]. Lipoprotein(a)/bl [Blood]. *Lipoproteins, LDL/bl [Blood]. Male. Middle Aged. Particle Size. Risk Factors. Testosterone/bl [Blood]. Tunica Media/me [Metabolism]. Tunica Media/pa [Pathology]. Ultrasonography.
Registry Number/Name of Substance
0 (Lipid Peroxides). 0 (Lipoprotein(a)). 0 (Lipoproteins, LDL). 3XMK78S47O (Testosterone). 67763-96-6 (Insulin-Like Growth Factor I).
Year of Publication
2004

Link to the Ovid Full Text or citation:
Click here for full text options

Link to the External Link Resolver:
SFX

124.

Ultrasonographic assessment of carotid wall characteristics and cognitive functions in a community sample of 59- to 71-year-olds. The EVA Study Group.
Auperin A; Berr C; Bonithon-Kopp C; Touboul PJ; Ruelland I; Ducimetiere P; Alperovitch A.
Stroke. 27(8):1290-5, 1996 Aug.
[Journal Article]
UI: 8711788
BACKGROUND AND PURPOSE: This study was aimed at analyzing cross-sectional relationships between cognitive performance and ultrasonographic assessment of carotid wall characteristics. METHODS: A cohort of 1279 subjects (men, 41%) aged 59 to 71 years was recruited from the electoral rolls of the city of Nantes (western France). Cognitive performances were evaluated with the Mini-Mental State Examination (MMSE) and seven neuropsychological tests assessing

attention, psychomotor rapidity, verbal abilities, memory, and visuospatial perception. For each test, subjects were classified into three performance levels with a quartile distribution: 25% highest, 25% lowest, and 50% middle. The intima-media thickness of common carotid arteries and the presence of plaques in the carotid arteries were assessed with B-mode ultrasound examination.
RESULTS: Only 28% of men and 17% of women had carotid plaques inducing moderate stenosis of the lumen (< 40%). After adjustment for possible confounders, odds ratios for poor cognitive performance associated with plaques were above 1 for all cognitive tests in men. This association was statistically significant for the MMSE and another test assessing attention skills. There was a slight association between increase of the common carotid intima-media thickness and poor cognitive scores in men with plaques. In women, no association was found between cognitive functions and presence of plaques or intima-media thickness.
CONCLUSIONS: This study indicated a moderate association between atherosclerosis of the carotid arteries and poor cognitive functioning in men aged 59 to 71 years. In view of these moderate cross-sectional results, further studies are required to better assess the relationship between carotid atherosclerosis and cognitive impairment.
Version ID
1
Record Owner
From MEDLINE, a database of the U.S. National Library of Medicine.
Status
MEDLINE
Authors Full Name
Auperin, A; Berr, C; Bonithon-Kopp, C; Touboul, P J; Ruelland, I; Ducimetiere, P; Alperovitch, A.
Institution
Auperin, A. INSERM U360, Hopital de la Salpetriere, Nantes, France.
MeSH Heading
Aged. *Aging/ph [Physiology]. Arteriosclerosis/dg [Diagnostic Imaging]. Arteriosclerosis/ep [Epidemiology]. Arteriosclerosis/pa [Pathology]. *Carotid Arteries/dg [Diagnostic Imaging]. Carotid Arteries/pa [Pathology]. *Cognition/ph [Physiology]. Cross-Sectional Studies. Female. Humans. Longitudinal Studies. Male. Middle Aged. Multivariate Analysis. Prevalence. Sex Factors. Ultrasonography.
Year of Publication
1996

Link to the Ovid Full Text or citation:
Click here for full text options

Link to the External Link Resolver:

SFX
