# Supplementary material for: Is carotid artery atherosclerosis associated with poor cognitive function assessed using the Mini-Mental State Examination? A systematic review and meta-analysis
Source: BMJ Open. 2022 Apr 18;12(4):e055131. doi: 10.1136/bmjopen-2021-055131 (PMC9020283; doi:10.1136/bmjopen-2021-055131)
Supplement: Supplementary data [file bmjopen-2021-055131supp003.pdf]

Eligibility Form: IS ASYMPTOMATIC ATHEROSCLEROSIS IN CAROTID ARTERIES ASSOCIATED WITH COGNITIVE FUNCTION?

A SYSTEMATIC REVIEW AND META-ANALYSIS

**1. Reference details**							
Reference citation							
First author							
Year of publication							
Title of the paper							
Assessor's identifier	1		2				
**2. Study eligibility**							
Inclusion of the study	Yes	√	No	√	Unclear r		
**Eligibility criteria**	**Reason for exclusion (if excluded)**					Yes	No
ALL studies except : review articles, conference abstract, letters to the editor, case reports; pilot study,	☐ no MMSE cognitive test					☐	☐
cIMT measured by ultrasound	☐ No IMT Assessment					√	☐
	☐ Other imaging modality not ultrasound					☐	☐
	☐ review articles, conference abstract, letters to the editor, case reports					☐	☐
Notes:	☐ Duplicate					☐	☐

**Additional information**
Notes:

DO NOT PROCEED IF PAPER EXCLUDED FROM REVIEW

Data Extraction Form

**3. Study details**		
Study (cohort) name	The EVA Study	
Study design	longitudinal study	
Region/country	Nantes (western France).	
Sample size	1279	
Mean (range) age	Mean ± SD : mean age, 65.0+/-3.0 years	Range (IQR):
Sex	Male, n (%): 526 men	Female, n (%): 753 women
Follow-up duration		
Clinical variables (n, %)		
Hypertension	n./a	
Diabetes mellitus	n/a	
**4. Exposure details**		
Hardware: Ultrasound Machine	an Aloka SSD-650 with a 7.5-MHz transducer.	
Method and site	The ultrasound examination involved scanning the common carotid arteries, the carotid bifurcations, and the first 2 cm of the internal carotid arteries. The IMT (distance between the media-adventitia interface and the lumen-intima interface) was automatically measured twice on longitudinal B-mode images of the far wall of each common carotid artery at plaque-free sites. The common carotid IMT was defined as the mean of four measurements. All segments of the carotid arteries were scanned longitudinally and transversely to assess the presence of plaques, and a localized echo-structure protruding into the lumen was considered to be a plaque if the distance between the media-adventitia interface and the internal side of the lesion was >or=to1 mm. The total number of plaques was recorded. For each, maximum thickness was measured perpendicular to the vessel wall. When several plaques were present in the same carotid segment (ie, common carotid artery or bifureation/origin of the internal carotid artery), plaque thickness measurement was made only for that with the greatest protrusion into the lumen. On the basis of transverse views, the degree of carotid stenosis was defined as (1-residual area/vessel area) times 100%.	

Notes:

**5. Outcomes' details**	
Type has of outcomes used in this review	Outcomes reported in this study (n)
**Primary outcome(s)**<br>Global cognitive function	neuropsychological battery that included seven tests assessing different cognitive functions and a global test (MMSE) of 18 items roughly assessing various cognitive skills with scores ranging from 0 to 30.
Notes:	

**6. Available number of participants**		
		Number
Baseline sample size	Yes ☐ No ☐	
follow-up	Yes ☐ No ☐	
Total number included in the analysis	Yes ☐ No ☐	
All subjects accounted for	Yes ☐ No ☐	
Notes:		

**7. Statistical analysis**	
Statistical method used	Nominal polychotomous logistic regressions [13] (BMDP statistical software) were used to analyze the relationships between each cognitive score distribution of the eight neuropsychological tests and carotid characteristics (presence of plaques, IMT), adjusting for age, education, depressive symptomatology, systolic blood pressure, body mass index, smoking, and alcohol consumption.
