# Supplementary material for: Is carotid artery atherosclerosis associated with poor cognitive function assessed using the Mini-Mental State Examination? A systematic review and meta-analysis
Source: BMJ Open. 2022 Apr 18;12(4):e055131. doi: 10.1136/bmjopen-2021-055131 (PMC9020283; doi:10.1136/bmjopen-2021-055131)
Supplement: Supplementary data [file bmjopen-2021-055131supp004.pdf]

BMJ Publishing Group Limited (BMJ) disclaims all liability and responsibility arising from any reliance placed on this supplemental material which has been supplied by the author(s)

A Modified Newcastle-Ottawa Quality Assessment for Observational Studies

The quality of included studies was assessed using a modified seven-point criteria derived from Newcastle-Ottawa scale (Wells et al., 2000). Each of the following criteria (A to G) was assigned 1 point if met.

**Title and reference :**				
**Criteria**				
**Sample**	**Yes**	**No**	**NR**	**Note**
A) Representativeness of the target sample<br>Yes = sampling designed to ensure adequate representativeness<br>No = inappropriately selected sample, or no description of sample selection				
B) Non-response satisfactorily dealt with<br>Yes = comparability between respondent and non-respondent characteristics established and response rate is satisfactory<br>No = the response rate is unsatisfactory, or the comparability between respondents and non-respondents is unsatisfactory, or no description of the response rate or the characteristics of the responders and the non-responders.				
**Exposure**	**Yes**	**No**	**NR**	**Note**
C) Validated measurement tool<br>Yes = high-resolution ultrasound system with linear ultrasound transducers at frequencies >7 MHz and measurement protocol for cIMT and plaque in accord[*] with American Society Echocardiography/Mannheim Consensus Guidelines. Data analysis performed independently and blinded using validated analysis system<br>No = inadequate ultrasound system, protocol, or analysis process, or not described				

[*] ‘In accord’ needs to be interpreted reasonably since some studies will precede these guidelines and others, while deviating slightly may not deviate sufficiently to be out of accord with the guidelines.

D) Relevant confounders measured[†]<br>Yes = sex, age, smoking, hypertension/blood pressure, diabetes, hyperlipidemia/blood lipids/body mass index<br>No = potential confounders not measured or not reported				
**Outcome**	**Yes**	**No**	**NR**	**Note**
E) Assessment of outcome<br>Yes = independent blinded assessment of cognitive status using a validated instrument<br>No = unvalidated instrument, or not described				
F) Statistical analysis<br>Yes = statistical analysis clearly described and appropriate: estimate of the central tendency of the outcome with a measure of precision is provided (e.g. mean/median (95% confidence interval/standard deviation/range)).<br>No = the statistical analysis is inappropriate, missing or inadequately described				
G) Missing data<br>Yes = extent of missing data reported and the methods for addressing missing data described<br>No = extent of missing data not reported and/or no description of how missing data were dealt with				
TOTAL (maximum 7)				

[†] relevant confounders listed here were selected on the basis of various components of the Framingham Cardiovascular risk score (https://framinghamheartstudy.org/fhs-risk-functions/cardiovascular-disease-10-year-risk/).

**Title and reference :**				
**Criteria**				
**Sample**	**Yes**	**No**	**NR**	**Note**
A) Representativeness of the target sample<br>Yes = sampling designed to ensure adequate representativeness<br>No = inappropriately selected sample, or no description of sample selection				
B) Non-response satisfactorily dealt with<br>Yes = comparability between respondent and non-respondent characteristics established and response rate is satisfactory<br>No = the response rate is unsatisfactory, or the comparability between respondents and non-respondents is unsatisfactory, or no description of the response rate or the characteristics of the responders and the non-responders.				
**Exposure**	**Yes**	**No**	**NR**	**Note**
C) Validated measurement tool<br>Yes = high-resolution ultrasound system with linear ultrasound transducers at frequencies >7 MHz and measurement protocol for cIMT and plaque in accord[‡] with American Society Echocardiography/Mannheim Consensus Guidelines. Data analysis performed independently and blinded using validated analysis system<br>No = inadequate ultrasound system, protocol, or analysis process, or not described				

[‡] 'In accord' needs to be interpreted reasonably since some studies will precede these guidelines and others, while deviating slightly may not deviate sufficiently to be out of accord with the guidelines.

D) Relevant confounders measured[§]<br>Yes = sex, age, smoking, hypertension/blood pressure, diabetes, hyperlipidemia/blood lipids/body mass index<br>No = potential confounders not measured or not reported				
**Outcome**	**Yes**	**No**	**NR**	**Note**
E) Assessment of outcome<br>Yes = independent blinded assessment of cognitive status using a validated instrument<br>No = unvalidated instrument, or not described				
F) Statistical analysis<br>Yes = statistical analysis clearly described and appropriate: estimate of the central tendency of the outcome with a measure of precision is provided (e.g. mean/median (95% confidence interval/standard deviation/range)).<br>No = the statistical analysis is inappropriate, missing or inadequately described				
G) Missing data<br>Yes = extent of missing data reported and the methods for addressing missing data described<br>No = extent of missing data not reported and/or no description of how missing data were dealt with				
TOTAL (maximum 7)				

[§] relevant confounders listed here were selected on the basis of various components of the Framingham Cardiovascular risk score (https://framinghamheartstudy.org/fhs-risk-functions/cardiovascular-disease-10-year-risk/).

**Title and reference :**				
**Criteria**				
**Sample**	**Yes**	**No**	**NR**	**Note**
A) Representativeness of the target sample<br>Yes = sampling designed to ensure adequate representativeness<br>No = inappropriately selected sample, or no description of sample selection				
B) Non-response satisfactorily dealt with<br>Yes = comparability between respondent and non-respondent characteristics established and response rate is satisfactory<br>No = the response rate is unsatisfactory, or the comparability between respondents and non-respondents is unsatisfactory, or no description of the response rate or the characteristics of the responders and the non-responders.				
**Exposure**	**Yes**	**No**	**NR**	**Note**
C) Validated measurement tool<br>Yes = high-resolution ultrasound system with linear ultrasound transducers at frequencies >7 MHz and measurement protocol for cIMT and plaque in accord[**] with American Society Echocardiography/Mannheim Consensus Guidelines. Data analysis performed independently and blinded using validated analysis system<br>No = inadequate ultrasound system, protocol, or analysis process, or not described				
D) Relevant confounders measured[††]<br>Yes = sex, age, smoking, hypertension/blood pressure, diabetes, hyperlipidemia/blood lipids/body mass index<br>No = potential confounders not measured or not reported				

[**] ‘In accord’ needs to be interpreted reasonably since some studies will precede these guidelines and others, while deviating slightly may not deviate sufficiently to be out of accord with the guidelines.

[††] relevant confounders listed here were selected on the basis of various components of the Framingham Cardiovascular risk score (https://framinghamheartstudy.org/fhs-risk-functions/cardiovascular-disease-10-year-risk/).

**Outcome**	**Yes**	**No**	**NR**	**Note**
E) Assessment of outcome<br>Yes = independent blinded assessment of cognitive status using a validated instrument<br>No = unvalidated instrument, or not described				
F) Statistical analysis<br>Yes = statistical analysis clearly described and appropriate: estimate of the central tendency of the outcome with a measure of precision is provided (e.g. mean/median (95% confidence interval/standard deviation/range)).<br>No = the statistical analysis is inappropriate, missing or inadequately described				
G) Missing data<br>Yes = extent of missing data reported and the methods for addressing missing data described<br>No = extent of missing data not reported and/or no description of how missing data were dealt with				
TOTAL (maximum 7)				

**Title and reference :**				
**Criteria**				
**Sample**	**Yes**	**No**	**NR**	**Note**
A) Representativeness of the target sample<br>Yes = sampling designed to ensure adequate representativeness<br>No = inappropriately selected sample, or no description of sample selection				
B) Non-response satisfactorily dealt with<br>Yes = comparability between respondent and non-respondent characteristics established and response rate is satisfactory<br>No = the response rate is unsatisfactory, or the comparability between respondents and non-respondents is unsatisfactory, or no description of the response rate or the characteristics of the responders and the non-responders.				
**Exposure**	**Yes**	**No**	**NR**	**Note**
C) Validated measurement tool<br>Yes = high-resolution ultrasound system with linear ultrasound transducers at frequencies >7 MHz and measurement protocol for cIMT and plaque in accord[‡‡] with American Society Echocardiography/Mannheim Consensus Guidelines. Data analysis performed independently and blinded using validated analysis system<br>No = inadequate ultrasound system, protocol, or analysis process, or not described				
D) Relevant confounders measured[§§]<br>Yes = sex, age, smoking, hypertension/blood pressure, diabetes, hyperlipidemia/blood lipids/body mass index<br>No = potential confounders not measured or not reported				

[‡‡] 'In accord' needs to be interpreted reasonably since some studies will precede these guidelines and others, while deviating slightly may not deviate sufficiently to be out of accord with the guidelines.

[§§] relevant confounders listed here were selected on the basis of various components of the Framingham Cardiovascular risk score (https://framinghamheartstudy.org/fhs-risk-functions/cardiovascular-disease-10-year-risk/).

**Outcome**	**Yes**	**No**	**NR**	**Note**
E) Assessment of outcome<br>Yes = independent blinded assessment of cognitive status using a validated instrument<br>No = unvalidated instrument, or not described				
F) Statistical analysis<br>Yes = statistical analysis clearly described and appropriate: estimate of the central tendency of the outcome with a measure of precision is provided (e.g. mean/median (95% confidence interval/standard deviation/range)).<br>No = the statistical analysis is inappropriate, missing or inadequately described				
G) Missing data<br>Yes = extent of missing data reported and the methods for addressing missing data described<br>No = extent of missing data not reported and/or no description of how missing data were dealt with				
TOTAL (maximum 7)				

**Title and reference :**				
**Criteria**				
**Sample**	**Yes**	**No**	**NR**	**Note**
A) Representativeness of the target sample<br>Yes = sampling designed to ensure adequate representativeness<br>No = inappropriately selected sample, or no description of sample selection				
B) Non-response satisfactorily dealt with<br>Yes = comparability between respondent and non-respondent characteristics established and response rate is satisfactory<br>No = the response rate is unsatisfactory, or the comparability between respondents and non-respondents is unsatisfactory, or no description of the response rate or the characteristics of the responders and the non-responders.				
**Exposure**	**Yes**	**No**	**NR**	**Note**
C) Validated measurement tool<br>Yes = high-resolution ultrasound system with linear ultrasound transducers at frequencies >7 MHz and measurement protocol for cIMT and plaque in accord[***] with American Society Echocardiography/Mannheim Consensus Guidelines. Data analysis performed independently and blinded using validated analysis system<br>No = inadequate ultrasound system, protocol, or analysis process, or not described				
D) Relevant confounders measured[†††]<br>Yes = sex, age, smoking, hypertension/blood pressure, diabetes, hyperlipidemia/blood lipids/body mass index<br>No = potential confounders not measured or not reported				

[***] 'In accord' needs to be interpreted reasonably since some studies will precede these guidelines and others, while deviating slightly may not deviate sufficiently to be out of accord with the guidelines.
[†††] relevant confounders listed here were selected on the basis of various components of the Framingham Cardiovascular risk score (https://framinghamheartstudy.org/fhs-risk-functions/cardiovascular-disease-10-year-risk/).

Outcome	Yes	No	NR	Note
E) Assessment of outcome<br>Yes = independent blinded assessment of cognitive status using a validated instrument<br>No = unvalidated instrument, or not described				
F) Statistical analysis<br>Yes = statistical analysis clearly described and appropriate: estimate of the central tendency of the outcome with a measure of precision is provided (e.g. mean/median (95% confidence interval/standard deviation/range)).<br>No = the statistical analysis is inappropriate, missing or inadequately described				
G) Missing data<br>Yes = extent of missing data reported and the methods for addressing missing data described<br>No = extent of missing data not reported and/or no description of how missing data were dealt with				
TOTAL (maximum 7)				

**Title and reference :**				
**Criteria**				
**Sample**	**Yes**	**No**	**NR**	**Note**
A) Representativeness of the target sample<br>Yes = sampling designed to ensure adequate representativeness<br>No = inappropriately selected sample, or no description of sample selection				
B) Non-response satisfactorily dealt with<br>Yes = comparability between respondent and non-respondent characteristics established and response rate is satisfactory<br>No = the response rate is unsatisfactory, or the comparability between respondents and non-respondents is unsatisfactory, or no description of the response rate or the characteristics of the responders and the non-responders.				
**Exposure**	**Yes**	**No**	**NR**	**Note**
C) Validated measurement tool<br>Yes = high-resolution ultrasound system with linear ultrasound transducers at frequencies >7 MHz and measurement protocol for cIMT and plaque in accord[‡‡‡] with American Society Echocardiography/Mannheim Consensus Guidelines. Data analysis performed independently and blinded using validated analysis system<br>No = inadequate ultrasound system, protocol, or analysis process, or not described				
D) Relevant confounders measured[§§§]<br>Yes = sex, age, smoking, hypertension/blood pressure, diabetes, hyperlipidemia/blood lipids/body mass index<br>No = potential confounders not measured or not reported				

[‡‡‡] 'In accord' needs to be interpreted reasonably since some studies will precede these guidelines and others, while deviating slightly may not deviate sufficiently to be out of accord with the guidelines.
[§§§] relevant confounders listed here were selected on the basis of various components of the Framingham Cardiovascular risk score (https://framinghamheartstudy.org/fhs-risk-functions/cardiovascular-disease-10-year-risk/).

**Outcome**	**Yes**	**No**	**NR**	**Note**
E) Assessment of outcome<br>Yes = independent blinded assessment of cognitive status using a validated instrument<br>No = unvalidated instrument, or not described				
F) Statistical analysis<br>Yes = statistical analysis clearly described and appropriate: estimate of the central tendency of the outcome with a measure of precision is provided (e.g. mean/median (95% confidence interval/standard deviation/range)).<br>No = the statistical analysis is inappropriate, missing or inadequately described				
G) Missing data<br>Yes = extent of missing data reported and the methods for addressing missing data described<br>No = extent of missing data not reported and/or no description of how missing data were dealt with				
TOTAL (maximum 7)				

**Title and reference :**				
**Criteria**				
**Sample**	**Yes**	**No**	**NR**	**Note**
A) Representativeness of the target sample<br>Yes = sampling designed to ensure adequate representativeness<br>No = inappropriately selected sample, or no description of sample selection				
B) Non-response satisfactorily dealt with<br>Yes = comparability between respondent and non-respondent characteristics established and response rate is satisfactory<br>No = the response rate is unsatisfactory, or the comparability between respondents and non-respondents is unsatisfactory, or no description of the response rate or the characteristics of the responders and the non-responders.				
**Exposure**	**Yes**	**No**	**NR**	**Note**
C) Validated measurement tool<br>Yes = high-resolution ultrasound system with linear ultrasound transducers at frequencies >7 MHz and measurement protocol for cIMT and plaque in accord[****] with American Society Echocardiography/Mannheim Consensus Guidelines. Data analysis performed independently and blinded using validated analysis system<br>No = inadequate ultrasound system, protocol, or analysis process, or not described				
D) Relevant confounders measured[††††]<br>Yes = sex, age, smoking, hypertension/blood pressure, diabetes, hyperlipidemia/blood lipids/body mass index<br>No = potential confounders not measured or not reported				

[****] 'In accord' needs to be interpreted reasonably since some studies will precede these guidelines and others, while deviating slightly may not deviate sufficiently to be out of accord with the guidelines.
[††††] relevant confounders listed here were selected on the basis of various components of the Framingham Cardiovascular risk score (https://framinghamheartstudy.org/fhs-risk-functions/cardiovascular-disease-10-year-risk/).

**Outcome**	**Yes**	**No**	**NR**	**Note**
E) Assessment of outcome<br>Yes = independent blinded assessment of cognitive status using a validated instrument<br>No = unvalidated instrument, or not described				
F) Statistical analysis<br>Yes = statistical analysis clearly described and appropriate: estimate of the central tendency of the outcome with a measure of precision is provided (e.g. mean/median (95% confidence interval/standard deviation/range)).<br>No = the statistical analysis is inappropriate, missing or inadequately described				
G) Missing data<br>Yes = extent of missing data reported and the methods for addressing missing data described<br>No = extent of missing data not reported and/or no description of how missing data were dealt with				
TOTAL (maximum 7)				

**Title and reference :**				
**Criteria**				
**Sample**	**Yes**	**No**	**NR**	**Note**
A) Representativeness of the target sample<br>Yes = sampling designed to ensure adequate representativeness<br>No = inappropriately selected sample, or no description of sample selection				
B) Non-response satisfactorily dealt with<br>Yes = comparability between respondent and non-respondent characteristics established and response rate is satisfactory<br>No = the response rate is unsatisfactory, or the comparability between respondents and non-respondents is unsatisfactory, or no description of the response rate or the characteristics of the responders and the non-responders.				
**Exposure**	**Yes**	**No**	**NR**	**Note**
C) Validated measurement tool<br>Yes = high-resolution ultrasound system with linear ultrasound transducers at frequencies >7 MHz and measurement protocol for cIMT and plaque in accord[‡‡‡‡] with American Society Echocardiography/Mannheim Consensus Guidelines. Data analysis performed independently and blinded using validated analysis system<br>No = inadequate ultrasound system, protocol, or analysis process, or not described				
D) Relevant confounders measured[§§§§]<br>Yes = sex, age, smoking, hypertension/blood pressure, diabetes, hyperlipidemia/blood lipids/body mass index<br>No = potential confounders not measured or not reported				

[‡‡‡‡] 'In accord' needs to be interpreted reasonably since some studies will precede these guidelines and others, while deviating slightly may not deviate sufficiently to be out of accord with the guidelines.
[§§§§] relevant confounders listed here were selected on the basis of various components of the Framingham Cardiovascular risk score (https://framinghamheartstudy.org/fhs-risk-functions/cardiovascular-disease-10-year-risk/).

**Outcome**	**Yes**	**No**	**NR**	**Note**
E) Assessment of outcome<br>Yes = independent blinded assessment of cognitive status using a validated instrument<br>No = unvalidated instrument, or not described				
F) Statistical analysis<br>Yes = statistical analysis clearly described and appropriate: estimate of the central tendency of the outcome with a measure of precision is provided (e.g. mean/median (95% confidence interval/standard deviation/range)).<br>No = the statistical analysis is inappropriate, missing or inadequately described				
G) Missing data<br>Yes = extent of missing data reported and the methods for addressing missing data described<br>No = extent of missing data not reported and/or no description of how missing data were dealt with				
TOTAL (maximum 7)				

**Title and reference :**				
**Criteria**				
**Sample**	**Yes**	**No**	**NR**	**Note**
A) Representativeness of the target sample<br>Yes = sampling designed to ensure adequate representativeness<br>No = inappropriately selected sample, or no description of sample selection				
B) Non-response satisfactorily dealt with<br>Yes = comparability between respondent and non-respondent characteristics established and response rate is satisfactory<br>No = the response rate is unsatisfactory, or the comparability between respondents and non-respondents is unsatisfactory, or no description of the response rate or the characteristics of the responders and the non-responders.				
**Exposure**	**Yes**	**No**	**NR**	**Note**
C) Validated measurement tool<br>Yes = high-resolution ultrasound system with linear ultrasound transducers at frequencies >7 MHz and measurement protocol for cIMT and plaque in accord[*****] with American Society Echocardiography/Mannheim Consensus Guidelines. Data analysis performed independently and blinded using validated analysis system<br>No = inadequate ultrasound system, protocol, or analysis process, or not described				
D) Relevant confounders measured[†††††]<br>Yes = sex, age, smoking, hypertension/blood pressure, diabetes, hyperlipidemia/blood lipids/body mass index<br>No = potential confounders not measured or not reported				

[*****] 'In accord' needs to be interpreted reasonably since some studies will precede these guidelines and others, while deviating slightly may not deviate sufficiently to be out of accord with the guidelines.
[†††††] relevant confounders listed here were selected on the basis of various components of the Framingham Cardiovascular risk score (https://framinghamheartstudy.org/fhs-risk-functions/cardiovascular-disease-10-year-risk/).

**Outcome**	**Yes**	**No**	**NR**	**Note**
E) Assessment of outcome<br>Yes = independent blinded assessment of cognitive status using a validated instrument<br>No = unvalidated instrument, or not described				
F) Statistical analysis<br>Yes = statistical analysis clearly described and appropriate: estimate of the central tendency of the outcome with a measure of precision is provided (e.g. mean/median (95% confidence interval/standard deviation/range)).<br>No = the statistical analysis is inappropriate, missing or inadequately described				
G) Missing data<br>Yes = extent of missing data reported and the methods for addressing missing data described<br>No = extent of missing data not reported and/or no description of how missing data were dealt with				
TOTAL (maximum 7)				

References

Wells, G. et al. (2000) The Newcastle-Ottawa Scale (NOS) for assessing the quality of nonrandomised studies in meta-analyses, Ottawa, ON: Ottawa Hospital Research Institute.

**Title and reference :**				
**Criteria**				
**Sample**	**Yes**	**No**	**NR**	**Note**
A) Representativeness of the target sample<br>Yes = sampling designed to ensure adequate representativeness<br>No = inappropriately selected sample, or no description of sample selection				
B) Non-response satisfactorily dealt with<br>Yes = comparability between respondent and non-respondent characteristics established and response rate is satisfactory<br>No = the response rate is unsatisfactory, or the comparability between respondents and non-respondents is unsatisfactory, or no				

description of the response rate or the characteristics of the responders and the non-responders.				
**Exposure**	**Yes**	**No**	**NR**	**Note**
C) Validated measurement tool<br>Yes = high-resolution ultrasound system with linear ultrasound transducers at frequencies >7 MHz and measurement protocol for cIMT and plaque in accord[‡‡‡‡‡] with American Society Echocardiography/Mannheim Consensus Guidelines. Data analysis performed independently and blinded using validated analysis system<br>No = inadequate ultrasound system, protocol, or analysis process, or not described				
D) Relevant confounders measured[§§§§§]<br>Yes = sex, age, smoking, hypertension/blood pressure, diabetes, hyperlipidemia/blood lipids/body mass index<br>No = potential confounders not measured or not reported				
**Outcome**	**Yes**	**No**	**NR**	**Note**
E) Assessment of outcome<br>Yes = independent blinded assessment of cognitive status using a validated instrument<br>No = unvalidated instrument, or not described				
F) Statistical analysis<br>Yes = statistical analysis clearly described and appropriate: estimate of the central tendency of the outcome with a measure of precision is provided (e.g. mean/median (95% confidence interval/standard deviation/range)).<br>No = the statistical analysis is inappropriate, missing or inadequately described				

[‡‡‡‡‡] 'In accord' needs to be interpreted reasonably since some studies will precede these guidelines and others, while deviating slightly may not deviate sufficiently to be out of accord with the guidelines.
[§§§§§] relevant confounders listed here were selected on the basis of various components of the Framingham Cardiovascular risk score (https://framinghamheartstudy.org/fhs-risk-functions/cardiovascular-disease-10-year-risk/).

G) Missing data<br>Yes = extent of missing data reported and the methods for addressing missing data described<br>No = extent of missing data not reported and/or no description of how missing data were dealt with				
TOTAL (maximum 7)				

**Title and reference :**				
**Criteria**				
**Sample**	**Yes**	**No**	**NR**	**Note**
A) Representativeness of the target sample<br>Yes = sampling designed to ensure adequate representativeness<br>No = inappropriately selected sample, or no description of sample selection				
B) Non-response satisfactorily dealt with<br>Yes = comparability between respondent and non-respondent characteristics established and response rate is satisfactory<br>No = the response rate is unsatisfactory, or the comparability between respondents and non-respondents is unsatisfactory, or no description of the response rate or the characteristics of the responders and the non-responders.				
**Exposure**	**Yes**	**No**	**NR**	**Note**
C) Validated measurement tool<br>Yes = high-resolution ultrasound system with linear ultrasound transducers at frequencies >7 MHz and measurement protocol for cIMT and plaque in accord[******] with American Society Echocardiography/Mannheim Consensus Guidelines. Data analysis performed independently and blinded using validated analysis system<br>No = inadequate ultrasound system, protocol, or analysis process, or not described				

[******] 'In accord' needs to be interpreted reasonably since some studies will precede these guidelines and others, while deviating slightly may not deviate sufficiently to be out of accord with the guidelines.

D) Relevant confounders measured[††††††]<br>Yes = sex, age, smoking, hypertension/blood pressure, diabetes, hyperlipidemia/blood lipids/body mass index<br>No = potential confounders not measured or not reported				
**Outcome**	**Yes**	**No**	**NR**	**Note**
E) Assessment of outcome<br>Yes = independent blinded assessment of cognitive status using a validated instrument<br>No = unvalidated instrument, or not described				
F) Statistical analysis<br>Yes = statistical analysis clearly described and appropriate: estimate of the central tendency of the outcome with a measure of precision is provided (e.g. mean/median (95% confidence interval/standard deviation/range)).<br>No = the statistical analysis is inappropriate, missing or inadequately described				
G) Missing data<br>Yes = extent of missing data reported and the methods for addressing missing data described<br>No = extent of missing data not reported and/or no description of how missing data were dealt with				
TOTAL (maximum 7)				

**Title and reference :**				
**Criteria**				
**Sample**	**Yes**	**No**	**NR**	**Note**
A) Representativeness of the target sample<br>Yes = sampling designed to ensure adequate representativeness<br>No = inappropriately selected sample, or no description of sample selection				
B) Non-response satisfactorily dealt with				

[††††††] relevant confounders listed here were selected on the basis of various components of the Framingham Cardiovascular risk score (https://framinghamheartstudy.org/fhs-risk-functions/cardiovascular-disease-10-year-risk/).

Yes = comparability between respondent and non-respondent characteristics established and response rate is satisfactory<br>No = the response rate is unsatisfactory, or the comparability between respondents and non-respondents is unsatisfactory, or no description of the response rate or the characteristics of the responders and the non-responders.				
**Exposure**	**Yes**	**No**	**NR**	**Note**
C) Validated measurement tool<br>Yes = high-resolution ultrasound system with linear ultrasound transducers at frequencies >7 MHz and measurement protocol for cIMT and plaque in accord[‡‡‡‡‡‡] with American Society Echocardiography/Mannheim Consensus Guidelines. Data analysis performed independently and blinded using validated analysis system<br>No = inadequate ultrasound system, protocol, or analysis process, or not described				
D) Relevant confounders measured[§§§§§§]<br>Yes = sex, age, smoking, hypertension/blood pressure, diabetes, hyperlipidemia/blood lipids/body mass index<br>No = potential confounders not measured or not reported				
**Outcome**	**Yes**	**No**	**NR**	**Note**
E) Assessment of outcome<br>Yes = independent blinded assessment of cognitive status using a validated instrument<br>No = unvalidated instrument, or not described				

[‡‡‡‡‡‡] ‘In accord’ needs to be interpreted reasonably since some studies will precede these guidelines and others, while deviating slightly may not deviate sufficiently to be out of accord with the guidelines.

[§§§§§§] relevant confounders listed here were selected on the basis of various components of the Framingham Cardiovascular risk score (https://framinghamheartstudy.org/fhs-risk-functions/cardiovascular-disease-10-year-risk/).

F) Statistical analysis<br>Yes = statistical analysis clearly described and appropriate: estimate of the central tendency of the outcome with a measure of precision is provided (e.g. mean/median (95% confidence interval/standard deviation/range)).<br>No = the statistical analysis is inappropriate, missing or inadequately described				
G) Missing data<br>Yes = extent of missing data reported and the methods for addressing missing data described<br>No = extent of missing data not reported and/or no description of how missing data were dealt with				
TOTAL (maximum 7)				

**Title and reference :**				
**Criteria**				
**Sample**	**Yes**	**No**	**NR**	**Note**
A) Representativeness of the target sample<br>Yes = sampling designed to ensure adequate representativeness<br>No = inappropriately selected sample, or no description of sample selection				
B) Non-response satisfactorily dealt with<br>Yes = comparability between respondent and non-respondent characteristics established and response rate is satisfactory<br>No = the response rate is unsatisfactory, or the comparability between respondents and non-respondents is unsatisfactory, or no description of the response rate or the characteristics of the responders and the non-responders.				
**Exposure**	**Yes**	**No**	**NR**	**Note**

C) Validated measurement tool<br>Yes = high-resolution ultrasound system with linear ultrasound transducers at frequencies >7 MHz and measurement protocol for cIMT and plaque in accord[*******] with American Society Echocardiography/Mannheim Consensus Guidelines. Data analysis performed independently and blinded using validated analysis system<br>No = inadequate ultrasound system, protocol, or analysis process, or not described				
D) Relevant confounders measured[†††††††]<br>Yes = sex, age, smoking, hypertension/blood pressure, diabetes, hyperlipidemia/blood lipids/body mass index<br>No = potential confounders not measured or not reported				
**Outcome**	**Yes**	**No**	**NR**	**Note**
E) Assessment of outcome<br>Yes = independent blinded assessment of cognitive status using a validated instrument<br>No = unvalidated instrument, or not described				
F) Statistical analysis<br>Yes = statistical analysis clearly described and appropriate: estimate of the central tendency of the outcome with a measure of precision is provided (e.g. mean/median (95% confidence interval/standard deviation/range)).<br>No = the statistical analysis is inappropriate, missing or inadequately described				

[*******] 'In accord' needs to be interpreted reasonably since some studies will precede these guidelines and others, while deviating slightly may not deviate sufficiently to be out of accord with the guidelines.
[†††††††] relevant confounders listed here were selected on the basis of various components of the Framingham Cardiovascular risk score (https://framinghamheartstudy.org/fhs-risk-functions/cardiovascular-disease-10-year-risk/).

G) Missing data<br>Yes = extent of missing data reported and the methods for addressing missing data described<br>No = extent of missing data not reported and/or no description of how missing data were dealt with				
TOTAL (maximum 7)				

**Title and reference :**				
**Criteria**				
**Sample**	**Yes**	**No**	**NR**	**Note**
A) Representativeness of the target sample<br>Yes = sampling designed to ensure adequate representativeness<br>No = inappropriately selected sample, or no description of sample selection				
B) Non-response satisfactorily dealt with<br>Yes = comparability between respondent and non-respondent characteristics established and response rate is satisfactory<br>No = the response rate is unsatisfactory, or the comparability between respondents and non-respondents is unsatisfactory, or no description of the response rate or the characteristics of the responders and the non-responders.				
**Exposure**	**Yes**	**No**	**NR**	**Note**
C) Validated measurement tool<br>Yes = high-resolution ultrasound system with linear ultrasound transducers at frequencies >7 MHz and measurement protocol for cIMT and plaque in accord[‡‡‡‡‡‡‡] with American Society Echocardiography/Mannheim Consensus Guidelines. Data analysis performed independently and blinded using validated analysis system<br>No = inadequate ultrasound system, protocol, or analysis process, or not described				

[‡‡‡‡‡‡‡] 'In accord' needs to be interpreted reasonably since some studies will precede these guidelines and others, while deviating slightly may not deviate sufficiently to be out of accord with the guidelines.

D) Relevant confounders measured[§§§§§§§]<br>Yes = sex, age, smoking, hypertension/blood pressure, diabetes, hyperlipidemia/blood lipids/body mass index<br>No = potential confounders not measured or not reported				
**Outcome**	**Yes**	**No**	**NR**	**Note**
E) Assessment of outcome<br>Yes = independent blinded assessment of cognitive status using a validated instrument<br>No = unvalidated instrument, or not described				
F) Statistical analysis<br>Yes = statistical analysis clearly described and appropriate: estimate of the central tendency of the outcome with a measure of precision is provided (e.g. mean/median (95% confidence interval/standard deviation/range)).<br>No = the statistical analysis is inappropriate, missing or inadequately described				
G) Missing data<br>Yes = extent of missing data reported and the methods for addressing missing data described<br>No = extent of missing data not reported and/or no description of how missing data were dealt with				
TOTAL (maximum 7)				

**Title and reference :**				
**Criteria**				
**Sample**	**Yes**	**No**	**NR**	**Note**
A) Representativeness of the target sample<br>Yes = sampling designed to ensure adequate representativeness<br>No = inappropriately selected sample, or no description of sample selection				
B) Non-response satisfactorily dealt with				

[§§§§§§§] relevant confounders listed here were selected on the basis of various components of the Framingham Cardiovascular risk score (https://framinghamheartstudy.org/fhs-risk-functions/cardiovascular-disease-10-year-risk/).

Yes = comparability between respondent and non-respondent characteristics established and response rate is satisfactory<br>No = the response rate is unsatisfactory, or the comparability between respondents and non-respondents is unsatisfactory, or no description of the response rate or the characteristics of the responders and the non-responders.				
**Exposure**	**Yes**	**No**	**NR**	**Note**
C) Validated measurement tool<br>Yes = high-resolution ultrasound system with linear ultrasound transducers at frequencies >7 MHz and measurement protocol for cIMT and plaque in accord[********] with American Society Echocardiography/Mannheim Consensus Guidelines. Data analysis performed independently and blinded using validated analysis system<br>No = inadequate ultrasound system, protocol, or analysis process, or not described				
D) Relevant confounders measured[††††††††]<br>Yes = sex, age, smoking, hypertension/blood pressure, diabetes, hyperlipidemia/blood lipids/body mass index<br>No = potential confounders not measured or not reported				
**Outcome**	**Yes**	**No**	**NR**	**Note**
E) Assessment of outcome<br>Yes = independent blinded assessment of cognitive status using a validated instrument<br>No = unvalidated instrument, or not described				

[********] 'In accord' needs to be interpreted reasonably since some studies will precede these guidelines and others, while deviating slightly may not deviate sufficiently to be out of accord with the guidelines.
[††††††††] relevant confounders listed here were selected on the basis of various components of the Framingham Cardiovascular risk score (https://framinghamheartstudy.org/fhs-risk-functions/cardiovascular-disease-10-year-risk/).

F) Statistical analysis<br>Yes = statistical analysis clearly described and appropriate: estimate of the central tendency of the outcome with a measure of precision is provided (e.g. mean/median (95% confidence interval/standard deviation/range)).<br>No = the statistical analysis is inappropriate, missing or inadequately described				
G) Missing data<br>Yes = extent of missing data reported and the methods for addressing missing data described<br>No = extent of missing data not reported and/or no description of how missing data were dealt with				
TOTAL (maximum 7)				

**Title and reference :**				
**Criteria**				
**Sample**	**Yes**	**No**	**NR**	**Note**
A) Representativeness of the target sample<br>Yes = sampling designed to ensure adequate representativeness<br>No = inappropriately selected sample, or no description of sample selection				
B) Non-response satisfactorily dealt with<br>Yes = comparability between respondent and non-respondent characteristics established and response rate is satisfactory<br>No = the response rate is unsatisfactory, or the comparability between respondents and non-respondents is unsatisfactory, or no description of the response rate or the characteristics of the responders and the non-responders.				
**Exposure**	**Yes**	**No**	**NR**	**Note**

C) Validated measurement tool<br>Yes = high-resolution ultrasound system with linear ultrasound transducers at frequencies >7 MHz and measurement protocol for cIMT and plaque in accord[‡‡‡‡‡‡‡‡] with American Society Echocardiography/Mannheim Consensus Guidelines. Data analysis performed independently and blinded using validated analysis system<br>No = inadequate ultrasound system, protocol, or analysis process, or not described				
D) Relevant confounders measured[§§§§§§§§]<br>Yes = sex, age, smoking, hypertension/blood pressure, diabetes, hyperlipidemia/blood lipids/body mass index<br>No = potential confounders not measured or not reported				
**Outcome**	**Yes**	**No**	**NR**	**Note**
E) Assessment of outcome<br>Yes = independent blinded assessment of cognitive status using a validated instrument<br>No = unvalidated instrument, or not described				
F) Statistical analysis<br>Yes = statistical analysis clearly described and appropriate: estimate of the central tendency of the outcome with a measure of precision is provided (e.g. mean/median (95% confidence interval/standard deviation/range)).<br>No = the statistical analysis is inappropriate, missing or inadequately described				

[‡‡‡‡‡‡‡‡] ‘In accord’ needs to be interpreted reasonably since some studies will precede these guidelines and others, while deviating slightly may not deviate sufficiently to be out of accord with the guidelines.
[§§§§§§§§] relevant confounders listed here were selected on the basis of various components of the Framingham Cardiovascular risk score (https://framinghamheartstudy.org/fhs-risk-functions/cardiovascular-disease-10-year-risk/).

G) Missing data<br>Yes = extent of missing data reported and the methods for addressing missing data described<br>No = extent of missing data not reported and/or no description of how missing data were dealt with				
TOTAL (maximum 7)				

**Title and reference :**				
**Criteria**				
**Sample**	**Yes**	**No**	**NR**	**Note**
A) Representativeness of the target sample<br>Yes = sampling designed to ensure adequate representativeness<br>No = inappropriately selected sample, or no description of sample selection				
B) Non-response satisfactorily dealt with<br>Yes = comparability between respondent and non-respondent characteristics established and response rate is satisfactory<br>No = the response rate is unsatisfactory, or the comparability between respondents and non-respondents is unsatisfactory, or no description of the response rate or the characteristics of the responders and the non-responders.				
**Exposure**	**Yes**	**No**	**NR**	**Note**
C) Validated measurement tool<br>Yes = high-resolution ultrasound system with linear ultrasound transducers at frequencies >7 MHz and measurement protocol for cIMT and plaque in accord[*********] with American Society Echocardiography/Mannheim Consensus Guidelines. Data analysis performed independently and blinded using validated analysis system<br>No = inadequate ultrasound system, protocol, or analysis process, or not described				

[*********] 'In accord' needs to be interpreted reasonably since some studies will precede these guidelines and others, while deviating slightly may not deviate sufficiently to be out of accord with the guidelines.

D) Relevant confounders measured[†††††††††]<br>Yes = sex, age, smoking, hypertension/blood pressure, diabetes, hyperlipidemia/blood lipids/body mass index<br>No = potential confounders not measured or not reported				
**Outcome**	**Yes**	**No**	**NR**	**Note**
E) Assessment of outcome<br>Yes = independent blinded assessment of cognitive status using a validated instrument<br>No = unvalidated instrument, or not described				
F) Statistical analysis<br>Yes = statistical analysis clearly described and appropriate: estimate of the central tendency of the outcome with a measure of precision is provided (e.g. mean/median (95% confidence interval/standard deviation/range)).<br>No = the statistical analysis is inappropriate, missing or inadequately described				
G) Missing data<br>Yes = extent of missing data reported and the methods for addressing missing data described<br>No = extent of missing data not reported and/or no description of how missing data were dealt with				
TOTAL (maximum 7)				

**Title and reference :**				
**Criteria**				
**Sample**	**Yes**	**No**	**NR**	**Note**
A) Representativeness of the target sample<br>Yes = sampling designed to ensure adequate representativeness<br>No = inappropriately selected sample, or no description of sample selection				
B) Non-response satisfactorily dealt with				

[†††††††††] relevant confounders listed here were selected on the basis of various components of the Framingham Cardiovascular risk score (https://framinghamheartstudy.org/fhs-risk-functions/cardiovascular-disease-10-year-risk/).

Yes = comparability between respondent and non-respondent characteristics established and response rate is satisfactory<br>No = the response rate is unsatisfactory, or the comparability between respondents and non-respondents is unsatisfactory, or no description of the response rate or the characteristics of the responders and the non-responders.				
**Exposure**	**Yes**	**No**	**NR**	**Note**
C) Validated measurement tool<br>Yes = high-resolution ultrasound system with linear ultrasound transducers at frequencies >7 MHz and measurement protocol for cIMT and plaque in accord[‡‡‡‡‡‡‡‡‡] with American Society Echocardiography/Mannheim Consensus Guidelines. Data analysis performed independently and blinded using validated analysis system<br>No = inadequate ultrasound system, protocol, or analysis process, or not described				
D) Relevant confounders measured[§§§§§§§§§]<br>Yes = sex, age, smoking, hypertension/blood pressure, diabetes, hyperlipidemia/blood lipids/body mass index<br>No = potential confounders not measured or not reported				
**Outcome**	**Yes**	**No**	**NR**	**Note**
E) Assessment of outcome<br>Yes = independent blinded assessment of cognitive status using a validated instrument<br>No = unvalidated instrument, or not described				

‡‡‡‡‡‡‡‡‡ 'In accord' needs to be interpreted reasonably since some studies will precede these guidelines and others, while deviating slightly may not deviate sufficiently to be out of accord with the guidelines.
§§§§§§§§§ relevant confounders listed here were selected on the basis of various components of the Framingham Cardiovascular risk score (https://framinghamheartstudy.org/fhs-risk-functions/cardiovascular-disease-10-year-risk/).

F) Statistical analysis<br>Yes = statistical analysis clearly described and appropriate: estimate of the central tendency of the outcome with a measure of precision is provided (e.g. mean/median (95% confidence interval/standard deviation/range)).<br>No = the statistical analysis is inappropriate, missing or inadequately described				
G) Missing data<br>Yes = extent of missing data reported and the methods for addressing missing data described<br>No = extent of missing data not reported and/or no description of how missing data were dealt with				
TOTAL (maximum 7)				

**Title and reference :**				
**Criteria**				
**Sample**	**Yes**	**No**	**NR**	**Note**
A) Representativeness of the target sample<br>Yes = sampling designed to ensure adequate representativeness<br>No = inappropriately selected sample, or no description of sample selection				
B) Non-response satisfactorily dealt with<br>Yes = comparability between respondent and non-respondent characteristics established and response rate is satisfactory<br>No = the response rate is unsatisfactory, or the comparability between respondents and non-respondents is unsatisfactory, or no description of the response rate or the characteristics of the responders and the non-responders.				
**Exposure**	**Yes**	**No**	**NR**	**Note**

C) Validated measurement tool<br>Yes = high-resolution ultrasound system with linear ultrasound transducers at frequencies >7 MHz and measurement protocol for cIMT and plaque in accord[**********] with American Society Echocardiography/Mannheim Consensus Guidelines. Data analysis performed independently and blinded using validated analysis system<br>No = inadequate ultrasound system, protocol, or analysis process, or not described				
D) Relevant confounders measured[††††††††††]<br>Yes = sex, age, smoking, hypertension/blood pressure, diabetes, hyperlipidemia/blood lipids/body mass index<br>No = potential confounders not measured or not reported				
**Outcome**	**Yes**	**No**	**NR**	**Note**
E) Assessment of outcome<br>Yes = independent blinded assessment of cognitive status using a validated instrument<br>No = unvalidated instrument, or not described				
F) Statistical analysis<br>Yes = statistical analysis clearly described and appropriate: estimate of the central tendency of the outcome with a measure of precision is provided (e.g. mean/median (95% confidence interval/standard deviation/range)).<br>No = the statistical analysis is inappropriate, missing or inadequately described				

[**********] 'In accord' needs to be interpreted reasonably since some studies will precede these guidelines and others, while deviating slightly may not deviate sufficiently to be out of accord with the guidelines.
[††††††††††] relevant confounders listed here were selected on the basis of various components of the Framingham Cardiovascular risk score (https://framinghamheartstudy.org/fhs-risk-functions/cardiovascular-disease-10-year-risk/).

G) Missing data<br>Yes = extent of missing data reported and the methods for addressing missing data described<br>No = extent of missing data not reported and/or no description of how missing data were dealt with				
TOTAL (maximum 7)				

**Title and reference :**				
**Criteria**				
**Sample**	**Yes**	**No**	**NR**	**Note**
A) Representativeness of the target sample<br>Yes = sampling designed to ensure adequate representativeness<br>No = inappropriately selected sample, or no description of sample selection				
B) Non-response satisfactorily dealt with<br>Yes = comparability between respondent and non-respondent characteristics established and response rate is satisfactory<br>No = the response rate is unsatisfactory, or the comparability between respondents and non-respondents is unsatisfactory, or no description of the response rate or the characteristics of the responders and the non-responders.				
**Exposure**	**Yes**	**No**	**NR**	**Note**
C) Validated measurement tool<br>Yes = high-resolution ultrasound system with linear ultrasound transducers at frequencies >7 MHz and measurement protocol for cIMT and plaque in accord[‡‡‡‡‡‡‡‡‡‡] with American Society Echocardiography/Mannheim Consensus Guidelines. Data analysis performed independently and blinded using validated analysis system<br>No = inadequate ultrasound system, protocol, or analysis process, or not described				

[‡‡‡‡‡‡‡‡‡‡] 'In accord' needs to be interpreted reasonably since some studies will precede these guidelines and others, while deviating slightly may not deviate sufficiently to be out of accord with the guidelines.

D) Relevant confounders measured[§§§§§§§§§§§]<br>Yes = sex, age, smoking, hypertension/blood pressure, diabetes, hyperlipidemia/blood lipids/body mass index<br>No = potential confounders not measured or not reported				
**Outcome**	**Yes**	**No**	**NR**	**Note**
E) Assessment of outcome<br>Yes = independent blinded assessment of cognitive status using a validated instrument<br>No = unvalidated instrument, or not described				
F) Statistical analysis<br>Yes = statistical analysis clearly described and appropriate: estimate of the central tendency of the outcome with a measure of precision is provided (e.g. mean/median (95% confidence interval/standard deviation/range)).<br>No = the statistical analysis is inappropriate, missing or inadequately described				
G) Missing data<br>Yes = extent of missing data reported and the methods for addressing missing data described<br>No = extent of missing data not reported and/or no description of how missing data were dealt with				
TOTAL (maximum 7)				

**Title and reference :**				
**Criteria**				
**Sample**	**Yes**	**No**	**NR**	**Note**
A) Representativeness of the target sample<br>Yes = sampling designed to ensure adequate representativeness<br>No = inappropriately selected sample, or no description of sample selection				
B) Non-response satisfactorily dealt with				

[§§§§§§§§§§§] relevant confounders listed here were selected on the basis of various components of the Framingham Cardiovascular risk score (https://framinghamheartstudy.org/fhs-risk-functions/cardiovascular-disease-10-year-risk/).

Yes = comparability between respondent and non-respondent characteristics established and response rate is satisfactory<br>No = the response rate is unsatisfactory, or the comparability between respondents and non-respondents is unsatisfactory, or no description of the response rate or the characteristics of the responders and the non-responders.				
**Exposure**	**Yes**	**No**	**NR**	**Note**
C) Validated measurement tool<br>Yes = high-resolution ultrasound system with linear ultrasound transducers at frequencies >7 MHz and measurement protocol for cIMT and plaque in accord[***********] with American Society Echocardiography/Mannheim Consensus Guidelines. Data analysis performed independently and blinded using validated analysis system<br>No = inadequate ultrasound system, protocol, or analysis process, or not described				
D) Relevant confounders measured[†††††††††††]<br>Yes = sex, age, smoking, hypertension/blood pressure, diabetes, hyperlipidemia/blood lipids/body mass index<br>No = potential confounders not measured or not reported				
**Outcome**	**Yes**	**No**	**NR**	**Note**
E) Assessment of outcome<br>Yes = independent blinded assessment of cognitive status using a validated instrument<br>No = unvalidated instrument, or not described				

*********** 'In accord' needs to be interpreted reasonably since some studies will precede these guidelines and others, while deviating slightly may not deviate sufficiently to be out of accord with the guidelines.

††††††††††† relevant confounders listed here were selected on the basis of various components of the Framingham Cardiovascular risk score (https://framinghamheartstudy.org/fhs-risk-functions/cardiovascular-disease-10-year-risk/).

F) Statistical analysis<br>Yes = statistical analysis clearly described and appropriate: estimate of the central tendency of the outcome with a measure of precision is provided (e.g. mean/median (95% confidence interval/standard deviation/range)).<br>No = the statistical analysis is inappropriate, missing or inadequately described				
G) Missing data<br>Yes = extent of missing data reported and the methods for addressing missing data described<br>No = extent of missing data not reported and/or no description of how missing data were dealt with				
TOTAL (maximum 7)				

**Title and reference :**				
**Criteria**				
**Sample**	**Yes**	**No**	**NR**	**Note**
A) Representativeness of the target sample<br>Yes = sampling designed to ensure adequate representativeness<br>No = inappropriately selected sample, or no description of sample selection				
B) Non-response satisfactorily dealt with<br>Yes = comparability between respondent and non-respondent characteristics established and response rate is satisfactory<br>No = the response rate is unsatisfactory, or the comparability between respondents and non-respondents is unsatisfactory, or no description of the response rate or the characteristics of the responders and the non-responders.				
**Exposure**	**Yes**	**No**	**NR**	**Note**

C) Validated measurement tool<br>Yes = high-resolution ultrasound system with linear ultrasound transducers at frequencies >7 MHz and measurement protocol for cIMT and plaque in accord[‡‡‡‡‡‡‡‡‡‡‡‡] with American Society Echocardiography/Mannheim Consensus Guidelines. Data analysis performed independently and blinded using validated analysis system<br>No = inadequate ultrasound system, protocol, or analysis process, or not described				
D) Relevant confounders measured[§§§§§§§§§§§§]<br>Yes = sex, age, smoking, hypertension/blood pressure, diabetes, hyperlipidemia/blood lipids/body mass index<br>No = potential confounders not measured or not reported				
**Outcome**	**Yes**	**No**	**NR**	**Note**
E) Assessment of outcome<br>Yes = independent blinded assessment of cognitive status using a validated instrument<br>No = unvalidated instrument, or not described				
F) Statistical analysis<br>Yes = statistical analysis clearly described and appropriate: estimate of the central tendency of the outcome with a measure of precision is provided (e.g. mean/median (95% confidence interval/standard deviation/range)).<br>No = the statistical analysis is inappropriate, missing or inadequately described				

[‡‡‡‡‡‡‡‡‡‡‡‡] ‘In accord’ needs to be interpreted reasonably since some studies will precede these guidelines and others, while deviating slightly may not deviate sufficiently to be out of accord with the guidelines.
[§§§§§§§§§§§§] relevant confounders listed here were selected on the basis of various components of the Framingham Cardiovascular risk score (https://framinghamheartstudy.org/fhs-risk-functions/cardiovascular-disease-10-year-risk/).

G) Missing data<br>Yes = extent of missing data reported and the methods for addressing missing data described<br>No = extent of missing data not reported and/or no description of how missing data were dealt with				
TOTAL (maximum 7)				

**Title and reference :**				
**Criteria**				
**Sample**	**Yes**	**No**	**NR**	**Note**
A) Representativeness of the target sample<br>Yes = sampling designed to ensure adequate representativeness<br>No = inappropriately selected sample, or no description of sample selection				
B) Non-response satisfactorily dealt with<br>Yes = comparability between respondent and non-respondent characteristics established and response rate is satisfactory<br>No = the response rate is unsatisfactory, or the comparability between respondents and non-respondents is unsatisfactory, or no description of the response rate or the characteristics of the responders and the non-responders.				
**Exposure**	**Yes**	**No**	**NR**	**Note**
C) Validated measurement tool<br>Yes = high-resolution ultrasound system with linear ultrasound transducers at frequencies >7 MHz and measurement protocol for cIMT and plaque in accord[************] with American Society Echocardiography/Mannheim Consensus Guidelines. Data analysis performed independently and blinded using validated analysis system<br>No = inadequate ultrasound system, protocol, or analysis process, or not described				

[************] 'In accord' needs to be interpreted reasonably since some studies will precede these guidelines and others, while deviating slightly may not deviate sufficiently to be out of accord with the guidelines.

D) Relevant confounders measured[††††††††††††]<br>Yes = sex, age, smoking, hypertension/blood pressure, diabetes, hyperlipidemia/blood lipids/body mass index<br>No = potential confounders not measured or not reported				
**Outcome**	**Yes**	**No**	**NR**	**Note**
E) Assessment of outcome<br>Yes = independent blinded assessment of cognitive status using a validated instrument<br>No = unvalidated instrument, or not described				
F) Statistical analysis<br>Yes = statistical analysis clearly described and appropriate: estimate of the central tendency of the outcome with a measure of precision is provided (e.g. mean/median (95% confidence interval/standard deviation/range)).<br>No = the statistical analysis is inappropriate, missing or inadequately described				
G) Missing data<br>Yes = extent of missing data reported and the methods for addressing missing data described<br>No = extent of missing data not reported and/or no description of how missing data were dealt with				
TOTAL (maximum 7)				

**Title and reference :**				
**Criteria**				
**Sample**	**Yes**	**No**	**NR**	**Note**
A) Representativeness of the target sample<br>Yes = sampling designed to ensure adequate representativeness<br>No = inappropriately selected sample, or no description of sample selection				
B) Non-response satisfactorily dealt with				

[††††††††††††] relevant confounders listed here were selected on the basis of various components of the Framingham Cardiovascular risk score (https://framinghamheartstudy.org/fhs-risk-functions/cardiovascular-disease-10-year-risk/).

Yes = comparability between respondent and non-respondent characteristics established and response rate is satisfactory<br>No = the response rate is unsatisfactory, or the comparability between respondents and non-respondents is unsatisfactory, or no description of the response rate or the characteristics of the responders and the non-responders.				
**Exposure**	**Yes**	**No**	**NR**	**Note**
C) Validated measurement tool<br>Yes = high-resolution ultrasound system with linear ultrasound transducers at frequencies >7 MHz and measurement protocol for cIMT and plaque in accord[‡‡‡‡‡‡‡‡‡‡‡‡] with American Society Echocardiography/Mannheim Consensus Guidelines. Data analysis performed independently and blinded using validated analysis system<br>No = inadequate ultrasound system, protocol, or analysis process, or not described				
D) Relevant confounders measured[§§§§§§§§§§§§§]<br>Yes = sex, age, smoking, hypertension/blood pressure, diabetes, hyperlipidemia/blood lipids/body mass index<br>No = potential confounders not measured or not reported				
**Outcome**	**Yes**	**No**	**NR**	**Note**
E) Assessment of outcome<br>Yes = independent blinded assessment of cognitive status using a validated instrument<br>No = unvalidated instrument, or not described				

[‡‡‡‡‡‡‡‡‡‡‡‡] 'In accord' needs to be interpreted reasonably since some studies will precede these guidelines and others, while deviating slightly may not deviate sufficiently to be out of accord with the guidelines.

[§§§§§§§§§§§§§] relevant confounders listed here were selected on the basis of various components of the Framingham Cardiovascular risk score (https://framinghamheartstudy.org/fhs-risk-functions/cardiovascular-disease-10-year-risk/).

F) Statistical analysis<br>Yes = statistical analysis clearly described and appropriate: estimate of the central tendency of the outcome with a measure of precision is provided (e.g. mean/median (95% confidence interval/standard deviation/range)).<br>No = the statistical analysis is inappropriate, missing or inadequately described				
G) Missing data<br>Yes = extent of missing data reported and the methods for addressing missing data described<br>No = extent of missing data not reported and/or no description of how missing data were dealt with				
TOTAL (maximum 7)				
