# Supplementary material for: Is carotid artery atherosclerosis associated with poor cognitive function assessed using the Mini-Mental State Examination? A systematic review and meta-analysis
Source: BMJ Open. 2022 Apr 18;12(4):e055131. doi: 10.1136/bmjopen-2021-055131 (PMC9020283; doi:10.1136/bmjopen-2021-055131)
Supplement: Supplementary data [file bmjopen-2021-055131supp005.pdf]

Supplementary Table S1A. Longitudinal studies

Reference	Location	Sampling frame / Health conditions	Sample size	Age (years±SD)	Female (%)	Presence of Plaque (n)	cIMT measurement (mm±SD)	MMSE (score±SD)	Major relevant finding
Auperin, et al. [1]	France	Community-based sample	Male: 521<br>Female: 753	65±3	60	Male: Yes (358)<br>Female: Yes (217)	Male: 0.69±0.14<br>Female: 0.65±0.11	Male: 28.3±2<br>Female: 27.9±2.3	No evidence of an association between cIMT and MMSE in either sex. Association between presence of carotid plaques and impaired MMSE in men. No evidence of an association in women

Watanabe, et al. [2]	Japan	Patients with vascular dementia (VaD) and age-matched controls	Control: 63<br><br>VaD: 37<br><br>AD: 34	C: 72±11<br>VaD: 75±8<br>AD: 76±9	0	Control: Yes (15)<br><br>VaD: Yes (28)<br><br>AD: Yes (18)	Control : 0.68±0.11<br>VaD: 0.94±0.2<br>AD: 0.85±0.14	Control : 26±3<br>VaD: 13±6<br>AD: 10±5	Comparing VaD patients with controls. MMSE was lower, cIMT was greater and carotid plaques more frequent
Haley, et al. [3]	USA	Cardiology patients and volunteers	109	69.18±7.43	43	N/A	0.88±0.13	28.55±1.59	cIMT was not independently related to performance on measures of global cognitive functioning. Increased cIMT was significantly associated with poorer performan

										ce in the attention-executive-psychomotor domain independent of risk factors.
Muller, et al. [4]	Netherlands	Community-based sample, age-stratified selection and further stratified by cardiovascular disease status	No CVD: 217 Subclinical CVD: 125 Prevalent CVD: 54	No CVD: 54±10.3 Subclinical CVD: 66.8±8.1 Prevalent CVD: 67.7±8.8	0	N/A	No CVD: 0.77±0.01 Subclinical CVD: 0.89±0.01 Prevalent CVD: 0.89±0.02	No CVD: >28 (n=100, 63%) Subclinical CVD: >28 (n=49, 31%) Prevalent CVD: >28 (n=11, 7%)	No CVD: ≤28 (n=117, 50%) Subclinical CVD: ≤28 (n=73, 31%) Prevalent CVD: ≤28 (n=43, 18%)	Association of cIMT with MMSE not presented. Thicker cIMT was associated with lower scores on memory functioning.
Singh-Manoux, et al. [5]	UK	Occupational sample excluding individuals with stroke	High SES, Male: 1190 High SES, Female: 185 Intermediate SES, Male: 1477 Intermediate SES, Female:490	High SES, Male: 62.32±5.61 High SES, Female: 59.8±5.49 Intermediate SES, Male: 60±71 Intermediate SES, Female: 60±5.93	High SES: 17 Intermediate SES: 45 Low SES: 38	N/A	N/A	High SES, Male: 0.8±0.16 High SES, Female: 0.76±0.13 Intermediate SES, Male: 0.79±0.16 Intermediate SES, Female: 0.77±0.13	High SES, Male: 28.9±1.19 High SES, Female: 29.16±1.04 Intermediate SES, Male: 28.71±1.31 Intermediate SES,	An inverse association between cIMT and cognition was observed only in the low SES group. Evidence that SES

			Low SES, Male: 141 Low SES, F: 413	Low SES, Male: 60.84±6.51 Low SES, Female: 62±5.73				Low SES, Male: 0.82±0.18 Low SES, Female: 0.79±0.14	Female: 28.88±1.24 Low SES, Male: 27.86±1.7 Low SES, Female: 28.18±1.55	modifies the associatio n between cIMT and cognition.
Carlsson, et al. [6]	USA	Population-based sample	No cognitive impairment : 1358 Cognitive impairment : 180	No cognitive impairment: 74.8±6.6 Cognitive impairment:80 ±7.1	No cognitive impairment : 62 Cognitive impairment : 54	N/A	N/A	No cognitive impairment: 0.95±0.23 Cognitive impairment: 1.00 ± 0.25	No cognitive impairment : 27.6 ± 1.7 Cognitive impairment : 21.1 ± 2.9	cIMT higher in people with cognitive impairmen t defined as MMSE <24 or proxy-reported dementia
El-Kattan, et al. [7]	Egypt	Vascular surgery patients and age- sex-matched healthy controls / PAD (66.6%)	Control: 10 PAD without CVD: 10 PAD with CVD: 10	Control: 53.9±9.55 PAD without CVD: 54.8±8.96 PAD with CVD: 56.1±9.62	Control: 20 PAD without CVD: 10 PAD with CVD: 20	N/A	Control: No (0) PAD without CVD: Yes (1) PAD with CVD: Yes (5)	Control: 0.7±0.1 PAD without CVD: 0.9±0.2 PAD with CVD: 1.07±0.13	Control: 30±0 PAD without CVD: 28±1.4 PAD with CVD: 26±1.5	Patients with peripheral arterial disease had higher cIMT and lower MMSE compared to healthy controls

Kearney-Schwartz, et al. [8]	France	Hypertensive patients with subjective memory complaints but excluding individuals with MMSW ≤24	198	69.3±6.2	53	N/A	Yes (50)	0.68±0.09	28.3±1.4	No evidence of an association between cIMT and impaired memory function implied, although data for MMSE or cognition not presented.
Zhong, et al. [9]	USA	Population-based sample	2794	49±9.8	54	N/A	Yes (2665)	0.65±0.15	28.7±1.3	Higher Carotid IMT and plaque were associated with lower MMSE score
Dias, et al. [10]	Brazil	HTN patients and non-HTN, non-cognitively impaired controls identified from	Controls: 48 HTN without cognitive impairment: 108 HTN with cognitive	Controls: 44.5±7.9 HTN without cognitive impairment: 55.75±8.6 HTN with cognitive	C 62.5 HTN without 53.7 HTN with 60.5	N/A		Controls: 0.69±1 HTN without cognitive impairment: 0.89±0.2 HTN with cognitive	Controls: 27.77±4.4 HTN without cognitive impairment: 28.44±1.3 HTN with cognitive	Higher cIMT was associated with higher odds ratio of MMSE score ≤24

		patients referred for investigation of high blood pressure. People with carotid plaques excluded.	impairment: 42	impairment: 63.3±9.3				impairment: 0.99±0.2	impairment: 22.02±2.4	
Stefanova, et al. [11]	Serbia	Patients with VCD or AD / VCD: Hyperlipidemia (36%) HTN (29%) DM (11%) AD: Hyperlipidemia (62%) HTN (88%) DM (28%)	VCD: 237 AD: 197	VCD: 67.4±8.18 AD: 68.5±9.36	VCD: 52.3 AD: 49	N/A N/A	VCD: Yes (120) AD: Yes (155)	VCD: 1.11±0.2 AD: 1.15±0.18	VCD: 23.81±2.33 AD: 16±5.9	Higher cIMT and plaque type was correlated with poorer MMSE in VCD. No evidence of a correlation between MMSE and cIMT or plaque in AD group.
**Rogne, et al. [12]**	Norway	Population-based sample / DM (1%)	1,577	Median (range): 57 (52-61)	48	N/A	N/A	Median (range): 0.78 (0.69–0.89)	N/A	No evidence of association between

		CHD (5.3%)								cIMT and MMSE.
**Xiang, et al.** [13]	China	Neurology patients / HTN (55%) DM (35%) CHD (10%)	No cognitive impairment : 1659 Cognitive impairment : 356	No cognitive impairment: 68.1±7.2 Cognitive impairment: 73.2±7.8	No cognitive impairment : 48 Cognitive impairment : 52	N/A N/A	Yes (1377) Yes (299)	No cognitive impairment: 0.76±0.14 Cognitive Impairment: 1.57±0.15	No cognitive impairment : ≥24 Cognitive impairment : <24	cIMT was associated with a higher odds ratio of cognitive impairment (MMSE <24)
**Nagai, et al.** [14]	Japan	Patients at high risk of cardiovascular disease / HTN (75.9%) DM (13.9%)	201	79.9±6.4	75	N/A	N/A	1.03±0.3	25.8±4.69	Compared with those with both low blood pressure variability and low IMT, patients with high blood pressure variability and high IMT had lower MMSE score or higher

										prevalence of cognitive impairment (based on a MMSE score ≤24)
Yano, et al. [15]	Japan	Hypertensive patients / HTN (100%)	587	73±8.1	59.0	N/A	N/A	NCD, median(range): 0.87(0.75-1.01) CD, median(range): 0.96 (0.82-1.06)	NCD, median(range): 28(27-29) CD, median(range): 24(22-24)	cIMT higher in patients with cognitive impairment (defined as MMSE score ≤ 24)
Liu, et al. [16]	China	Community-based sample / HTN (71%) DM (13%)	384	84.65±2.3	67	N/A	N/A	1.45 ± 0.30	Median (range): 24 (22-24)	cIMT inversely related to MMSE score.
Alhusaini, et al. [17]	Scotland	Population-based cohort HTN (47.3%) DM (10.1%)	518	72.7±0.73	46.6	N/A	Yes (77)	0.84±0.17	28.8±1.3	Data relating cIMT to MMSE not presented. Carotid stenosis was related to lower fluid

										intelligence.
Matsumoto, et al. [18]	Japan	Patients undergoing health screening	176	No cognitive impairment: 64.6 ±9.6 Cognitive impairment: 67.7±12.3	No cognitive impairment: 59.1 Cognitive impairment: 37	N/A	N/A	No cognitive impairment:1.7 ±0.7 Cognitive impairment: 2.0±1	N/A	No evidence of an association between cIMT and MMSE in a model adjusted for sex, age and years of education. cIMT higher in patients with cognitive impairment.
Muela, et al. [19]	Brazil	Patients. Individuals with cerebrovascular disease excluded / HTN (67%)	Normotensive: 69 HTN stage 1: 83 HTN stage 2: 59	Normotensive: 52.1±13.9 HTN stage 1: 52.1±13 HTN stage 2: 51.3±10.1	Normotensive: 55.1 HTN stage 1: 55.8 HTN stage 2: 53.6	N/A	N/A	Normotensive: 0.7±0.1 HTN stage 1: 0.8±0.1 HTN stage 2: 0.8±0.1	Normotensive: 28.03±1.92 HTN stage 1: 27.43±2.01	cIMT showed a weak negative correlation with MMSE consistent

									HTN stage 2: 26.66±2.07	with null. Negative correlations of cIMT with other cognitive measures were observed.
Mworozi, et al. [20]	Uganda	Community-based study HTN (25%) DM (5%)	210	69.9±7.76	71.4	N/A	Yes (45)	0.9±0.2	N/A	Presence of carotid artery plaque was associated with abnormal cognitive function defined as MMSE ≤24.

Supplementary Table S1B. Longitudinal studies

Reference	Location	Sampling frame / Health conditions	Sample size	Age, year (mean±SD)	Female, %	Mean follow-up (years)	Presence of Plaque (n)	cIMT measurement (mm±SD)	MMSE (score±SD)	Major relevant finding
Silvestrini, et al. [21]	Italy	Dementia clinic outpatients	66	72.7±6.1	55	1	Yes (52)	1.1±0.3	18.1±3	Higher baseline cIMT was associated with greater decline in MMSE scores
Viticchi, et al. [22]	Italy	Patients referred to Dementia clinic / Hyperlipidemia (55.6%) HTN (65%) DM (29.1%)	Cognitively stable: 96 Developed dementia: 21	Cognitively stable: 75±6 Developed dementia: 77.2±4.28	Cognitively stable:44.8 Developed dementia: 66.7	1	Cognitively stable: Yes (30) Developed dementia: Yes (9)	Cognitively stable : 0.92±0.15 Developed dementia: 1.04±0.16	Cognitively stable 27.14±1.76 Developed dementia: 27±1.56	Patients with abnormal cIMT (>1mm) had higher odds ratio of progressing from mild cognitive impairment to AD dementia
Zhong, et al. [23]	USA	Population-based sample	1311	66.8	59	5 and 10	Yes (682)	0.86±0.21	N/A	Higher cIMT was associated with higher risk of incident

										cognitive impairment (MMSE <24). Plaque was not associated with incident cognitive impairment or cognitive test performanc e.
Buratti, et al. [24]	Italy	Patients with high vascular risk	159 Normal cIMT: 68 Pathologic al cIMT: 91)	Normal cIMT:69.7±3.6 PcIMT:70.2±3.8	NcIMT:39.7 PcIMT:37.4	3	Normal cIMT:Yes (68) PcIMT:Yes (91)	Normal cIMT: < 1 PcIMT: ≥ 1	Normal cIMT baseline: 26.75±1.05 Normal cIMT follow-up:24.86±1.85 Pathological cIMT baseline: 27±1.25 Pathological cIMT follow-up: 25.27±1.86	No evidence of an association between cIMT and change in MMSE score

Buratti, et al. [25]	Italy	Patients referred to Dementia clinic	MCI: 300<br>AD: 106	MCI, meadian (range): 72 (67-76)<br>AD, meadian (range): 72 (68-76)	MCI: 37<br>AD: 43	1	MCI: Yes (114)<br>AD: Yes (53)	N/A	MCI: 27±1<br>AD: 27±1	cIMT >1mm and plaque associated with increased odds ratio of developme nt of Alzheimer disease over 12-month follow-up
Wendell, et al. [26]	USA	Community-based sample	1696	46.9±9.3	55	4	N/A	0.69±0.13	N/A	Weak negative association between cIMT and MMSE in fully adjusted models, consistent with null.

Falsetti, et al. [27]	Italy	Patients from neurological clinic with mild to moderate cognitive impairment	310	76.86±7.49	62.9	2	N/A	pNVAF(-) : 0.97±0.2 pNVAF(+) : 1.11±0.16	pNVAF(-) : 18.62±5.22 pNVAF(+): 15.8±5.17	cIMT weakly associated with progression to probably Alzheimer Disease but results compatible with null. Patients with non-valvular atrial fibrillation (pNVAF(+)) had lower MMSE and higher cIMT compared to pNVAF(-).

Rouch, et al. [28]	N/A	Consecutive patients attending a memory clinic	363	75.2±7	65.6	4.5	No conversion to dementia: Yes (50) Conversion to dementia: Yes (40)	No conversion to dementia: 0.85±0.13 Conversion to dementia : 0.82±0.13	No conversion to dementia: 28.3±1.6 Conversion to dementia: 26.6±2.6	Higher cIMT and plaque associated with progression from MCI to dementia based on MMSE score.

Supplementary Table S1C. Cross-sectional plus longitudinal studies

Reference	Location	Sampling frame / Health conditions	Sample size	Age (years±SD)	Female (%)	Follow-up (years)	Presence of Plaque (n)	cIMT measurement (mm±SD)	MMSE (score±SD)	Major relevant finding
Komulainen, et al. [29]	Finland	Population-based sample	91 (CS) 47 (LT)	63.5±3.1 (CS) 75±3.2 (LT)	100	12	N/A	1.02±0.26 (Cross-sectional) 1.25±0.33 (LTl)	28.9±1.6 (Cross-sectional) 26.4±2.01 (LT)	There was no evidence of an association between IMT and MMSE score cross-sectionally or after 12-year follow-up, although confidence intervals of estimates were very wide. Associations between high cIMT and poor memory were seen both cross-sectionally and longitudinally.

Carcaillon, et al. [30]	France	Population-based sample	5798	73.4±4.8	60.5	7	Yes (3038)	0.71±0.12	N/A	Association between baseline carotid plaques and incident dementia. No association between cIMT and incident dementia
Kawasaki, et al. [31]	Japan	Population-based sample	494	Median (range): 87.2 (86.1–88.7)	55	3	Yes (328)	N/A	N/A	Presence of higher carotid artery plaque score was associated with lower MMSE scores. Weak evidence of an association between plaque score and increase rate of decline in MMSE compatible with null.

Abbreviations: AD: Alzheimer's disease, cIMT: carotid intima-media thickness, CD: cognitive dysfunction, CHD: coronary heart disease, CS: cross-sectional, CVD: cardiovascular disease, DM: Diabetes mellitus, HTN: Hypertensive, ICA: internal carotid artery, LT: longitudinal, MMSE: Mini Mental State Examination, MCI: mild cognitive impairment, N/A: not available, PAD: peripheral arterial disease, pNVAF: permanent non-valvular atrial fibrillation, SES; socioeconomic status, VaD: vascular dementia, VCD: vascular cognitive decline.

Supplementary Table S2. Exposure measures of the included papers.

Reference	IMT Measurement sites	Scanning techniques	Intra-observer reproducibility, blinded
Auperin, et al. [1]	Bilateral CCA, including the carotid bifurcation, and the first 2 cm of the ICA	Scanned longitudinally and transversely to assess the presence of plaques	Inter-reader agreement regarding the presence of plaques was excellent (κ coefficient=.90).
Watanabe, et al. [2]	Bilateral CCA including the carotid bifurcation and from 10 mm below the bifurcation,	Anterolateral and posterolateral angles of CCA with beam focused on the far wall	All measurements were determined by the same examiner, who was blinded to clinical history or risk factor profile.
Komulainen, et al. [29]	Bilateral far wall of CCA and bifurcation	N/A	sonographers were blinded to the randomization status of the study participants
Haley, et al. [3]	Left, far wall CCA 1 cm proximal to the carotid bulb	N/A	N/A
Muller, et al. [4]	Bilateral distal CCA	N/A	the intra-class correlation coefficient (ICC) for repeated IMT-measurement was 84%
Singh-Manoux, et al. [5]	Bilateral CCA	Longitudinal images triggered on the R-wave of the ECG	N/A
Silvestrini, et al. [21]	Bilateral CCA	of IMT taken as the thickest plaque-free region on the near and far walls in longitudinal images	N/A
Carlsson, et al. [6]	Bilateral, distal CCA, bifurcation, and the proximal portion of ICA	near and far walls scanned in each vessel segment (total of 12 sites)	N/A

El-Kattan, et al. [7]	No details provided	Arteries assessed using longitudinal views and checked for the state of arterial wall and the presence of thrombi	N/A
Kearney-Schwartz, et al. [8]	Bilateral CCA	Arteries assessed using longitudinal views	N/A
Zhong, et al. [9]	Bilateral, near and far walls of CCA, bifurcation and ICA	N/A	The reproducibility of IMT and plaque assessment was good. In a 10% sample (n=280) of participant scans that were re-graded the mean difference in IMT was 0.0019 mm and kappa statistics for plaque assessment ranged from 0.58 (ICA) to 0.71(bifurcation) with 97.3% agreement within ±1 for the number of sites with plaque.
Dias, et al. [10]	Bilateral CCA	The intima-media thickness was measured in near and far walls over a 1-cm segment of the artery located approximately 0.5 cm below the carotid-artery bulb and considered not to contain any plaque	The variability between IMT measurements less than 2%,; analysis performed by a physician blinded to the patient's clinical data
Zhong, et al. [23]	Bilateral CCA	Near and far walls	The reproducibility of IMT and plaque assessment was good. The mean inter-grader difference in IMT was 0.03 mm; and for plaque, the kappa coefficient was 0.76 and percent agreement was 90%

Viticchi, et al. [22]	Bilateral, distal segment of CCA	Measurement of near and far wall IMT on longitudinal image of 1.5cm segment of CCA that precedes the carotid bifurcation. Measurement made with an automated system at the thickest point where there were no plaques	N/A
Stefanova, et al. [11]	Far wall of CCA, ICA during diastole	during diastole measurements were done in a supine position with head elevated up to 45°, and tilted to the either side for 30°, depending on the side examined	The inter-rater correlation reliability assessed for 50 randomly selected patients from both groups was excellent (0.932), Physicians that performed ultrasound examinations were blinded to clinical data
Rogne, et al. [12]	Right, far and near wall CCA, bulb	measurement of IMT was performed in 10-mm segments of the far and near wall of the common carotid artery in the most proximal 10-mm segment of the bulb. The CCA, the bifurcation and the internal carotid artery were examined for plaque presence.	N/A
Xiang, et al. [13]	Bilateral, far wall CCA	Measurement of far wall cIMT in longitudinal B-mode images of the CCA, the carotid bifurcations, and the first 2 cm of the ICA at plaque-free sites	N/A
Nagai, et al. [14]	Bilateral, far wall CCA	CCA scanned bilaterally in longitudinal and transverse projections. The image was focused on the far wall of the artery	N/A
Yano, et al. [15]	Bilaterally at CCA, the bulb, and ICA	CCA, the bulb, and ICA measured from both transverse and longitudinal orientations, Region with the thickest IMT measured.	Coefficient of variation within 10%. Scan performed blind to patient's data
Buratti, et al. [24]	Bilateral CCA	N/A	inter-reader correlation coefficient of 0.88
Buratti, et al. [25]	Bilateral CCA	A longitudinal image of the distal segment of common carotid arteries was taken, and the measurement was obtained with an automatic system at the thickest point where there were no plaques on the proximal and distal wall	N/A

Carcaillon, et al. [30]	Bilateral CCA, bulb, and ICA	scanned longitudinally and transversally to detect plaques.	N/A
Kawasaki, et al. [31]	Bilateral CCA, ICA	N/A	All examinations were performed by a single physician blinded to the subject’s clinical information
Liu, et al. [16]	Bilateral CCA	Three B-mode images were obtained using anterior, lateral, and medial angles. Maximum IMT in the right or left CCA used.	Scanning done by certified ultrasonographer who was unaware of the subjects' clinical details
Wendell, et al. [26]	Left CCA	Far wall IMT measured over a region 1.5 cm proximal to the carotid bifurcation	Intraobserver correlation between repeated carotid IMT measurements on 10 participants was 0.96 (p <0.001).
Rouch, et al. [28]	Bilateral CCA	Near and far wall IMT measured in longitudinal images. Longitudinal and transverse images examined for plaques	N/A
Alhusaini, et al. [17]	Far walls of CCA, and bulb*	Far wall cIMT measured as the mean of 3 caliper measurements over a 1cm-long segment of the CCA and carotid bulb	N/A
Matsumoto, et al. [18]	Bilateral, far wall CCA, ICA	far wall maximum cIMT of the bilateral CCA,ICA measured at end-diastole in longitudinal images	Certified sonographers who were blinded with cognitive test results carried out the carotid ultrasonography
Muela, et al. [19]	Left CCA	Near and far wall cIMT was measured at the thickest point of the distal CCA, not including plaques using a computer program	an experienced observer who were blinded with Clinical condition carried out the carotid ultrasonography
Falsetti, et al. [27]	Bilateral CCA	cIMT measurements made on 1.5 cm segment of CCA artery preceding carotid bifurcation in a longitudinal image using a semiautomatic system	N/A
Mworozi, et al. [20]	CCA, ICA*	Participants scanned in both supine and semi recumbent positions, with the head slightly hyperextended and rotated 45 degrees away from the side being examined	N/A

Abbreviations: (ICA) internal carotid artery, (ECA) the external carotid artery, (CCA) common carotid artery , (CIMT) carotid intima-media thickness , (N/A) not applicable. *not clear whether measured bilaterally.

Supplementary Table S3. The quality of included studies assessed using a modified seven-point criteria derived from Newcastle-Ottawa scale [32].

**Reference**	**Quality score Newcastle-Ottawa scale (0-7)**
Auperin, et al. [1]	3
Watanabe, et al. [2]	5
Komulainen, et al. [29]	4
Haley, et al. [3]	4
Muller, et al. [4]	4
Singh-Manoux, et al. [5]	4
Silvestrini, et al. [21]	4
Carlsson, et al. [6]	4
El-Kattan, et al. [7]	4
Kearney-Schwartz, et al. [8]	4
Zhong, et al. [9]	4
Dias, et al. [10]	4
Zhong, et al. [23]	4
Viticchi, et al. [22]	6
Stefanova, et al. [11]	4
Rogne, et al. [12]	4
Xiang, et al. [13]	4
Nagai, et al. [14]	4
Yano, et al. [15]	3

Buratti, et al. [24]	5
Buratti, et al. [25]	4
Carcaillon, et al. [30]	5
Kawasaki, et al. [31]	5
Liu, et al. [16]	4
Wendell, et al. [26]	4
Rouch, et al. [28]	5
Alhusaini, et al. [17]	4
Matsumoto, et al. [18]	2
Muela, et al. [19]	3
Falsetti, et al. [27]	4
Mworozi, et al. [20]	3

References

1. Auperin A, Berr C, Bonithon-Kopp C, et al. Ultrasonographic assessment of carotid wall characteristics and cognitive functions in a community sample of 59- to 71-year-olds. The EVA Study Group. *Stroke* 1996;27(8):1290-5. doi: 10.1161/01.str.27.8.1290 [published Online First: 1996/08/01]
2. Watanabe T, Koba S, Kawamura M, et al. Small dense low-density lipoprotein and carotid atherosclerosis in relation to vascular dementia. *Metabolism, clinical and experimental* 2004;53(4):476-82. doi: 10.1016/j.metabol.2003.11.020
3. Haley AP, Forman DE, Poppas A, et al. Carotid artery intima-media thickness and cognition in cardiovascular disease. *International Journal of Cardiology* 2007;121(2):148-54.
4. Muller M, Grobbee DE, Aleman A, et al. Cardiovascular disease and cognitive performance in middle-aged and elderly men. *Atherosclerosis* 2007;190(1):143-49. doi: 10.1016/j.atherosclerosis.2006.01.005
5. Singh-Manoux A, Britton A, Kivimaki M, et al. Socioeconomic status moderates the association between carotid intima-media thickness and cognition in midlife: Evidence from the Whitehall II study. *Atherosclerosis* 2008;197(2):541-48.
6. Carlsson CM, Nondahl DM, Klein BE, et al. Increased atherogenic lipoproteins are associated with cognitive impairment: effects of statins and subclinical atherosclerosis. *Alzheimer Dis Assoc Disord* 2009;23(1):11-7. doi: 10.1097/wad.0b013e3181850188 [published Online First: 2009/03/10]
7. El-Kattan MM, Zakaria YA, El-Fayomy N, et al. Peripheral arterial disease and cognition. *Egyptian Journal of Neurology, Psychiatry and Neurosurgery* 2009;46(2):311-22.
8. Kearney-Schwartz A, Rossignol P, Bracard S, et al. Vascular Structure and Function Is Correlated to Cognitive Performance and White Matter Hyperintensities in Older Hypertensive Patients With Subjective Memory Complaints. *Stroke* 2009;40(4):1229-36.
9. Zhong W, Cruickshanks KJ, Huang GH, et al. Carotid atherosclerosis and cognitive function in midlife: the Beaver Dam Offspring Study. *Atherosclerosis* 2011;219(1):330-3. doi: 10.1016/j.atherosclerosis.2011.07.013 [published Online First: 2011/08/13]
10. Dias EDM, Giollo LT, Martinelli DD, et al. Carotid intima-media thickness is associated with cognitive deficiency in hypertensive patients with elevated central systolic blood pressure. *Cardiovascular Ultrasound* 2012;10(1)
11. Stefanova E, Pavlovic A, Jovanovic Z, et al. Vascular risk factors in Alzheimer's disease - preliminary report. *J Neurol Sci* 2012;322(1-2):166-9. doi: 10.1016/j.jns.2012.07.065 [published Online First: 2012/09/04]
12. Rogne SO, Solbu MD, Arntzen KA, et al. Albuminuria and carotid atherosclerosis as predictors of cognitive function in a general population. *European Neurology* 2013;70(5-6):340-8.
13. Xiang J, Zhang T, Yang QW, et al. Carotid artery atherosclerosis is correlated with cognitive impairment in an elderly urban Chinese non-stroke population. *Journal of Clinical Neuroscience* 2013;20(11):1571-75.
14. Nagai M, Hoshide S, Nishikawa M, et al. Visit-to-visit blood pressure variability in the elderly: associations with cognitive impairment and carotid artery remodeling. *Atherosclerosis* 2014;233(1):19-26. doi: 10.1016/j.atherosclerosis.2013.11.071 [published Online First: 2014/02/18]

15. Yano Y, Bakris GL, Inokuchi T, et al. Association of cognitive dysfunction with cardiovascular disease events in elderly hypertensive patients. *J Hypertens* 2014;32(2):423-31. doi: 10.1097/HJH.0000000000000025 [published Online First: 2013/12/20]
16. Liu Z, Zhao Y, Wang X, et al. Low carotid artery wall shear stress is independently associated with brain white-matter hyperintensities and cognitive impairment in older patients. *Atherosclerosis* 2016;247:78-86. doi: 10.1016/j.atherosclerosis.2016.02.003 [published Online First: 2016/02/13]
17. Alhusaini S, Karama S, Nguyen TV, et al. Association between carotid atheroma and cerebral cortex structure at age 73 years. *Ann Neurol* 2018;84(4):576-87. doi: 10.1002/ana.25324 [published Online First: 2018/09/05]
18. Matsumoto L, Suzuki K, Mizuno Y, et al. Association of subclinical carotid atherosclerosis with immediate memory and other cognitive functions. *Geriatrics & gerontology international* 2018;18(1):65-71.
19. Muela HCS, Costa-Hong VA, Yassuda MS, et al. Higher arterial stiffness is associated with lower cognitive performance in patients with hypertension. *Journal of Clinical Hypertension* 2018;20(1):22-30.
20. Mworozi K, Ameda F, Byanyima RK, et al. Carotid artery plaque detected on ultrasound is associated with impaired cognitive state in the elderly: A population-based study in Wakiso district, Uganda. *Journal of Clinical Neuroscience* 2019;68:194-200.
21. Silvestrini M, Gobbi B, Pasqualetti P, et al. Carotid atherosclerosis and cognitive decline in patients with Alzheimer's disease. *Neurobiology of Aging* 2009;30(8):1177-83.
22. Viticchi G, Falsetti L, Vernieri F, et al. Vascular predictors of cognitive decline in patients with mild cognitive impairment. *Neurobiology of Aging* 2012;33(6):1127e1-27e9.
23. Zhong W, Cruickshanks KJ, Schubert CR, et al. Carotid atherosclerosis and 10-year changes in cognitive function. *Atherosclerosis* 2012;224(2):506-10. doi: 10.1016/j.atherosclerosis.2012.07.024 [published Online First: 2012/08/03]
24. Buratti L, Balucani C, Viticchi G, et al. Cognitive deterioration in bilateral asymptomatic severe carotid stenosis. *Stroke* 2014;45(7):2072-77.
25. Buratti L, Balestrini S, Altamura C, et al. Markers for the Risk of Progression from Mild Cognitive Impairment to Alzheimer's Disease. *Journal of Alzheimer's Disease* 2015;45(3):883-90.
26. Wendell CR, Waldstein SR, Evans MK, et al. Subclinical carotid atherosclerosis and neurocognitive function in an urban population. *Atherosclerosis* 2016;249:125-31. doi: 10.1016/j.atherosclerosis.2016.04.009 [published Online First: 2016/04/20]
27. Falsetti L, Viticchi G, Buratti L, et al. Interactions between Atrial Fibrillation, Cardiovascular Risk Factors, and ApoE Genotype in Promoting Cognitive Decline in Patients with Alzheimer's Disease: A Prospective Cohort Study. *Journal of Alzheimer's Disease* 2018;62(2):713-25.
28. Rouch L, Cestac P, Sallerin B, et al. Pulse wave velocity is associated with greater risk of dementia in mild cognitive impairment patients. *Journal of Hypertension* 2018;36 (Supplement 3):e340.
29. Komulainen P, Kivipelto M, Lakka TA, et al. Carotid intima-media thickness and cognitive function in elderly women: A population-based study. *Neuroepidemiology* 2007;28(4):207-13.
30. Carcaillon L, Plichart M, Zureik M, et al. Carotid plaque as a predictor of dementia in older adults: the Three-City Study. *Alzheimer's & Dementia* 2015;11(3):239-48.
31. Kawasaki M, Arai Y, Hirata T, et al. Carotid atherosclerosis, cytomegalovirus infection, and cognitive decline in the very old: a community-based prospective cohort study. *Age* 2016;38(2):1-13.

32. Wells G, Shea B, O'Connell D, et al. The Newcastle–Ottawa Scale (NOS) for Assessing the Quality of Non-Randomized Studies in Meta-Analysis. , 2000.
