# Supplementary material for: Is carotid artery atherosclerosis associated with poor cognitive function assessed using the Mini-Mental State Examination? A systematic review and meta-analysis
Source: BMJ Open. 2022 Apr 18;12(4):e055131. doi: 10.1136/bmjopen-2021-055131 (PMC9020283; doi:10.1136/bmjopen-2021-055131)
Supplement: Author's manuscript [file bmjopen-2021-055131.draft_revisions.pdf]

BMJ Open

BMJ Open is committed to open peer review. As part of this commitment we make the peer review history of every article we publish publicly available.

When an article is published we post the peer reviewers' comments and the authors' responses online. We also post the versions of the paper that were used during peer review. These are the versions that the peer review comments apply to.

The versions of the paper that follow are the versions that were submitted during the peer review process. They are not the versions of record or the final published versions. They should not be cited or distributed as the published version of this manuscript.

BMJ Open is an open access journal and the full, final, typeset and author-corrected version of record of the manuscript is available on our site with no access controls, subscription charges or pay-per-view fees (http://bmjopen.bmj.com).

If you have any questions on BMJ Open's open peer review process please email info.bmjopen@bmj.com

Is Carotid Artery Atherosclerosis Associated with Poor Cognitive Function Assessed using the Mini-Mental State Examination? a systematic review and meta-analysis

Journal:	*BMJ Open*
Manuscript ID	bmjopen-2021-055131
Article Type:	Original research
Date Submitted by the Author:	04-Jul-2021

Complete List of Authors:	Anbar, Rayan; King Abdulaziz University, Diagnostic Radiology, Faculty of Applied Medical Sciences; University College London, MRC Unit for Lifelong Health and Aging, UCL Institute of Cardiovascular Science, Faculty of Population Health Sciences<br>Sultan, Salahaden; King Abdulaziz University, Diagnostic Radiology, Faculty of Applied Medical Sciences<br>Al Saikhan, Lamia; Imam Abdulrahman Bin Faisal University, College of Applied Medial Sciences<br>Alkharaiji, Mohammed ; Saudi Electronic University, Department of Public Health<br>Hardy, Rebecca; University College London, Social Research Institute, UCL Institute of Education<br>Richards, M; University College London, MRC Unit for Lifelong Health and Aging, UCL Institute of Cardiovascular Science<br>Chaturvedi, Nishi; University College London, MRC Unit for Lifelong Health and Aging, UCL Institute of Cardiovascular Science<br>Hughes, A; University College London, MRC Unit for Lifelong Health and Aging, UCL Institute of Cardiovascular Science

Keywords:	Ultrasound < RADIOLOGY & IMAGING, Cardiovascular imaging < RADIOLOGY & IMAGING, Dementia < NEUROLOGY, VASCULAR MEDICINE

SCHOLARONE™
Manuscripts

I, the Submitting Author has the right to grant and does grant on behalf of all authors of the Work (as defined in the below author licence), an exclusive licence and/or a non-exclusive licence for contributions from authors who are: i) UK Crown employees; ii) where BMJ has agreed a CC-BY licence shall apply, and/or iii) in accordance with the terms applicable for US Federal Government officers or employees acting as part of their official duties; on a worldwide, perpetual, irrevocable, royalty-free basis to BMJ Publishing Group Ltd ("BMJ") its licensees and where the relevant Journal is co-owned by BMJ to the co-owners of the Journal, to publish the Work in this journal and any other BMJ products and to exploit all rights, as set out in our licence.

The Submitting Author accepts and understands that any supply made under these terms is made by BMJ to the Submitting Author unless you are acting as an employee on behalf of your employer or a postgraduate student of an affiliated institution which is paying any applicable article publishing charge ("APC") for Open Access articles. Where the Submitting Author wishes to make the Work available on an Open Access basis (and intends to pay the relevant APC), the terms of reuse of such Open Access shall be governed by a Creative Commons licence – details of these licences and which Creative Commons licence will apply to this Work are set out in our licence referred to above.

Other than as permitted in any relevant BMJ Author's Self Archiving Policies, I confirm this Work has not been accepted for publication elsewhere, is not being considered for publication elsewhere and does not duplicate material already published. I confirm all authors consent to publication of this Work and authorise the granting of this licence.

For peer review only

Is Carotid Artery Atherosclerosis Associated with Poor Cognitive Function Assessed using the Mini-Mental State Examination? a systematic review and meta-analysis

Rayan Anbar[1,2], Salahaden R. Sultan[1], Lamia Al Saikhan[3], Mohammed Alkharaiji[4], Nish Chaturvedi[2], Rebecca Hardy[5], Marcus Richards[2], Alun D. Hughes[2]

1. Department of Diagnostic Radiology, Faculty of Applied Medical Sciences, King Abdulaziz University, Jeddah, Saudi Arabia

2. MRC Unit for Lifelong Health and Aging, UCL Institute of Cardiovascular Science, Faculty of Population Health Sciences, University College London, London, UK

3. Department of Cardiac Technology, College of Applied Medial Sciences, Imam Abdulrahman Bin Faisal University, 2835 King Faisal Street, Dammam 34212, Kingdom of Saudi Arabia

4. Faculty of Public Health, College of Health Science, The Saudi Electronic University, Riyadh, Saudi Arabia

5. Social Research Institute, UCL Institute of Education, University College London, London, UK

Corresponding author:

Prof Alun Hughes, MRC Unit for Lifelong Health and Aging, UCL Institute of Cardiovascular Science, Faculty of Population Health Sciences, University College London, London, UK. Email: alun.hughes@ucl.ac.uk

For peer review only

ABSTRACT

Objectives: To determine associations between carotid atherosclerosis assessed by ultrasound and the mini-mental state examination (MMSE), a measure of global cognitive function.

Design: Systematic review and meta-analysis.

Methods: MEDLINE and EMBASE databases were searched up to 01/05/2020 to identify studies assessed the associations between asymptomatic carotid atherosclerosis and the MMSE. Studies reporting odds ratios (OR) for associations between carotid plaque or intima-media thickness (cIMT) and dichotomised MMSE were meta-analysed. Publication bias of included studies was assessed.

Results: A total of 31 of 378 reviewed articles met the inclusion criteria; together they included 27,738 participants (age 35 to 95 years). Fifteen studies reported some evidence of a positive association between measures of atherosclerosis and poorer cognitive performance in either cross-sectional or longitudinal studies. The remaining 16 studies found no evidence of an association. Seven cross-sectional studies provided data suitable for meta-analysis. Meta-analysis of three studies that assessed carotid plaque (n= 3,549) showed an association between the presence of plaque and impaired MMSE with pooled estimate for the OR (95% CI) being 2.72 (0.85, 4.59). An association between cIMT and impaired MMSE was reported in six studies (n=4,443) with a pooled estimate for the OR (95% CI) being 1.13 (1.04, 1.22). Heterogeneity across studies was moderate to small (carotid plaque with MMSE, I^2=40.9%; cIMT with MMSE, I^2=4.9%). There was evidence of publication bias for carotid plaque studies (p=0.02), but not cIMT studies (p=0.2).

Conclusions: There is some, limited cross-sectional evidence indicating an association between cIMT and poorer global cognitive function assessed with MMSE. Estimates of the association between plaques and poor cognition are too imprecise to draw firm conclusions and evidence from studies of longitudinal associations between carotid atherosclerosis and MMSE is limited.

Keywords: atherosclerosis, cognitive function, carotid artery, ultrasound, systematic review, Meta-analysis, Mini Mental State Examination, intima-media thickness.

For peer review only

Strengths and limitations of this study

- We performed a systematic review on 31 published studies assessed the association between carotid atherosclerosis and global cognition.
- The association between carotid atherosclerosis and global cognition was meta-analysed on 7,992 asymptomatic patients.
- There was some evidence from cross-sectional studies that carotid plaque and cIMT were associated with impaired MMSE.
- Methods used and characteristics of participants across studies were different which makes it difficult to compare all studies.
- This review only included studies which used MMSE as a cognitive function test which may result in bias or an underestimation of decline in cognitive state.

Competing interests' statement

Rayan Anbar – none

Salahaden R Sultan – none

Lamia Al Saikhan – none

Mohammed Alkharaiji – none

Nish Chaturvedi – NC has received personal fees from Astra Zeneca outside of the submitted work.

Rebecca Hardy – none

Marcus Richards – none

Alun Hughes – none

For peer review only

INTRODUCTION

Improvements in life expectancy have led to a dramatic increase in the worldwide burden of age-related diseases, notably atherosclerotic cardiovascular disease, and cognitive decline and dementia[1].

Atherosclerosis is a chronic inflammatory disease, which begins in childhood[2]. With time and progression of disease, fibro-fatty plaques develop which may result in alterations in the elastic properties of the artery, narrowing of the lumen, or thromboembolic consequences. Atherosclerosis affects most large arteries, including the common carotid artery (CCA) and its major branch the internal carotid artery[3]. Carotid artery atherosclerosis is common [4] and may be an important cause of cerebral ischaemia and cognitive impairment. While assessment of carotid disease can be performed using a range of imaging methods, (e.g. magnetic resonance imaging (MRI), computed tomography (CT), angiography, and ultrasound [5]), ultrasound is the most commonly used [6-9], as it is inexpensive, non-invasive and widely available.

A decline in cognitive function is common with advancing age,[10] and severe cognitive impairment and dementia are increasingly frequent. Cognitive function refers to internal mental processes which help people to think, make decisions and solve problems[11], and cognitive function decline can affect patients' health, daily routine, learning new things, speech and writing abilities, and to live independently. Mild Cognitive Impairment (MCI), dementia and Alzheimer's are the most common types of cognitive dysfunction[12]. A comprehensive assessment of neuropsychological cognitive performance at the domain level is time consuming and most studies use brief cognitive screening tools[13]. The most widely-used tool for cognitive testing is the MMSE[14-16]. MMSE contains 11 tasks and covers 7 domains: visuospatial skills, language, concentration, working memory, memory recall, and orientation and is scored out of a maximum 30 points.[17] The commonest cut-points to detect dementia with the MMSE are ≤23 or ≤24, although higher and lower cut-points have been used in some studies[15 16]. While it is relatively easy to use, the MMSE has a number of limitations, including non-linearity, a floor effect in advanced dementia, a ceiling effect in very mild disease, and bias in people with little formal education or in non-English speaking groups[14-16].

Several studies have used a combination of carotid ultrasound and MMSE to examine the associations between carotid atherosclerosis and cognitive performance, either cross-sectionally or longitudinally; however, these studies have yielded conflicting results. For

For peer review only

example, the Rotterdam Study [18] reported that common carotid artery intima-media thickness (cIMT) was associated with increased risk of Alzheimer's disease, but not vascular dementia, whereas carotid plaques were not associated with either Alzheimer's disease or vascular dementia. In contrast, the Framingham Offspring Cohort study [19] reported that cIMT was not associated with any measure of cognition, although there was evidence of an association between cIMT and impaired verbal memory and nonverbal memory, and that carotid stenosis ≥50% was associated with impaired executive function, but not verbal and non-verbal memory.

The aim of this study therefore was to systematically review evidence of an association between carotid atherosclerosis assessed by ultrasound and global cognitive function assessed using the MMSE, also to perform a meta-analysis of quantitative measures of association between carotid atherosclerosis and global cognitive function.

METHODS

This study was conducted according to the 'Preferred Reporting Items for Systematic Reviews and Meta-Analyses'(PRISMA) statement[20] and registered in the international prospective register of systematic reviews (PROSPERO) (registration number CRD42021240077). Patients or the public were not involved in the design, or conduct, or reporting, or dissemination plans of our research.

Search strategy

The search was conducted systematically (up to 01/05/2020) by two authors independently in two online databases: MEDLINE and EMBASE Classic + EMBASE. A combination of synonyms and related words and text word searching was used to comprehensively extract all relevant articles. A combination of indexed (MesH) terms and keyword searches in titles and abstracts was used - (1) cognition, cognitive function, dementia, Alzheimer disease; (2) atherosclerosis, intima media thickness, plaque; (3) carotid arteries;(4) All searches were limited to (English language and humans). The results from databases were exported to an Endnote library, and any duplicates results were identified and removed before screening the records. The records of non-relevant articles were excluded by screening titles and abstracts by three authors working independently. The remaining publications were assessed by screening the full texts for eligibility and were retrieved and double screened. Discrepancies were reviewed and resolved through consensus.

For peer review only - http://bmjopen.bmj.com/site/about/guidelines.xhtml

Inclusion criteria

Relevant studies were required to include the following: (1) Adults (2) Carotid intima media thickness and/or carotid artery plaque measured using ultrasound and (3) Mini-mental state examination (MMSE) as a global cognitive function test.

Exclusion criteria

The exclusion criteria as follows: (1) Adults following stroke (if results in stroke patients were reported separately these were excluded, or if the study only included participants with stroke the entire study was excluded); (2) Patients who had undergone carotid surgical and non-surgical intervention; (3) Studies performed in animals; (4) Studies that used medical imaging modalities other than ultrasound to assess the presence of plaque and to measure carotid atherosclerosis; (5) Review articles, conference abstracts, case reports, letters to the editor, or commentary articles.

Data extraction

Data extraction from the included studies were performed by three researchers independently using standardized forms; any discrepancies were resolved by consensus. The extracted data from the eligible studies consisted of study characteristics such as author, year of publication, sample size, characteristics of participants (age, and gender), study region, MMSE cognitive function test score, and cIMT measurements (Tables 1,A,B,C). Means and standard deviations and sample size of cIMT and MMSE were extracted from the included papers. If mean and standard deviation were not provided, median and range or other measures of central tendency and dispersion were extracted. Maximum cIMT was extracted if the mean value was not reported in the studies. If necessary, odds ratios for cIMT were converted to be per 1mm IMT. If the percentage of people with plaques was reported, the total number was calculated. For studies in which stroke sufferers were included in the sample size, the total number of subjects were re-calculated by removing subjects with stroke. The sample size was extracted of which cIMT and MMSE were measured. If different numbers of subject underwent cIMT and MMSE assessments, the lower number was recorded. The percentage of female subjects was extracted. If the total number of females was provided, the percentage of females from the overall sample size was calculated. The data was collected from baseline values or nearest value to the baseline if baseline was not available. If there were data from the article that was deficient, the authors were contacted by email.

Meta-analysis

A meta-analysis was planned using odds ratios (OR) with 95% confidence intervals (CI) as the estimates of the strength of association between carotid atherosclerosis and MMSE, with MMSE categorised into normal or impaired on the basis of a cut-point of 24. This choice was based on the commonest mode of analysis in the papers identified. If other measures of dispersion were reported these were converted into 95% CI. To facilitate comparison of cross-sectional hazard ratios or risk ratios from longitudinal studies were converted to OR using the equation described by Grant[21]. Meta-analysis was performed using Stata/SE Statistical Software version 16.1 (StataCorp., College Station, TX). Q-test and I^2 statistics were used to assessed heterogeneity between studies. We used the Egger's test and funnel plots to look for the possibility of publication bias.

Quality Assessment

The quality of included studies was scored using a modified seven-point criteria were derived from Newcastle-Ottawa scale[22]. Scores covered the 1) representativeness of the target sample, 2) non-response satisfactorily dealt with, 3) use of a validated measurement tool, 4) relevant confounders measured, 5) appropriate assessment of outcome, 6) appropriate statistical analysis clearly described and 7) reporting missing data if present. Each criterion was assigned one point if met. The quality assessment was performed by two researchers independently using standardized forms and any discrepancies were resolved by consensus .

RESULTS

The PRISMA flowchart showing the study selection process is shown in Fig 1. We identified 378 potential studies from the electronic databases (Medline & Embase) after exclusion of duplicates. Title and abstract screening resulted in 48 records remaining to be reviewed for eligibility in full text. Of those studies, 17 were excluded with reasons. The reasons were: no cIMT measurement (n = 4), or no MMSE test data (n = 9) or use of a modified version of MMSE (n = 3), review articles, conference abstract, letters to the editor or case reports (n = 1).

For peer review only

Characteristics studies included in the systematic review

The total number of participants in the 31 included studies was 26,178, with an age range from 35 to 95 years. Twenty of the studies were cross-sectional (Table 1A); eight were longitudinal (Table 1C). The remaining 3 studies included a mixture of cross-sectional and longitudinal data (Table 1B). The geographical distribution of the studies was diverse: with 5 from Italy, 5 from Japan, 5 from USA, 3 from France, 2 from Brazil, 2 from China, 3 from UK, and 1 from each of Egypt, Netherlands, Norway, Serbia, Sweden, and Uganda. One study failed to mention the geographical location. Only one study [23] failed to report the patients' health conditions and risk factors, such as diabetes mellitus, hypertension, and chronic kidney disease. Two studies included only male participants [24 25], and one study included only female participants [26], the remaining studies included both male and female participants. Details of the key exposure measures are shown in Table 2. 29 studies reported the mean of carotid intima media thickness measurement (Table 1)[23-51]. 14 studies did not mention the presence of carotid plaques[24 26-29 34 38 41 42 44 45 47-49]. Seven studies did not provide MMSE scores[37 38 43 45 48 51 52].

Associations between atherosclerosis measures and MMSE score

Cross-sectional associations

Presence of carotid plaques was associated with poorer cognitive function in 3 studies [33 51 52]. Auperin et al. [23] reported an association between higher prevalence of carotid plaque and lower MMSE in men, but not in women. Another study [28] concluded that the association between cIMT and cognitive function was observed only in a low socioeconomic status group. Another study [35] found a significant correlation between higher cIMT and plaques with poor MMSE score in people with evidence of vascular cognitive decline, but not in a group of people with Alzheimer's disease and Watanabe et al. [25] reported that vascular dementia patients were more likely to have low MMSE score with thicker cIMT and frequent presences of carotid plaques. Ten cross-sectional studies [27 29 30 34 36 39 41 44 49 53] reported that cIMT was not associated with changes in MMSE, although some of these studies reported associations with a selected aspect of cognitive performance.

For peer review only

Longitudinal associations

Zhong et al. reported an association between higher cIMT and a reduction in MMSE score after 10 years of follow-up but not after 5-years; a similar association between presence of carotid plaque and change in MMSE was not observed at any follow-up interval[37]. Rouch et al. found that cIMT and carotid plaques were associated with progression to dementia in univariable analyses, but neither predicted progression after multivariable adjustment [50]. Another study found that the plaque index, and cIMT were predictive of an increased risk of conversion of mild cognitive impairment to dementia including after multivariable adjustment using a backwards stepwise method[54]. While Carcaillon et al. reported that there was an association between presence of carotid plaque in two sites or more and incidence of dementia, they observed no association between cIMT measured at plaque-free sites with incident dementia[43]. Silvestrini et al. found that baseline cIMT and increase in cIMT adjusted for baseline were associated with decline in MMSE[32]. Wendell et al.found no association between baseline cIMT and change in MMSE, but observed that higher cIMT was associated with future changes in selected aspects of cognitive performance[45]. One study did not provide quantitative data about possible associations between carotid plaque or cIMT and MMSE [47], while 4 other studies found no evidence that cIMT was associated with future global cognitive impairment [26 38 40 48].

Quality

One study received quality scores of 6 out of 7 [36]; five received a quality score of 5 out of 7 [25 40 43 50 52]; twenty a quality score of 4 out of 7 [24 26-35 37-39 41 44-47 54]; four a quality score of 3 out of 7 [23 42 49 51]; and one a quality score of 2 out of 7 [48]. Among the 31 included studies, 24 studies were adequately designed to represent the target sample. 9 publications used validated ultrasound measurement tools, 6 reported blinding assessment of outcome and blinding of outcome measurements, 29 described the statistical analysis satisfactorily.

Publication Bias

Egger`s test showed evidence of bias in studies assessing between carotid plaque and MMSE (p=0.02), but not in cIMT with MMSE (p=0.2) (Figure 4 A&B).

Meta-analysis

Seven of the 31 studies reported data that were suitable for inclusion in a meta-analysis [23 24 34 37 39 48 51]. All included studies were cross-sectional, and all but one drew from population samples. Of all studies, 3 reported associations between carotid plaque and impaired MMSE [23 39 55], and 6 that reported the association between cIMT and impaired MMSE [23 24 34 39 48 54]. There was only one suitable longitudinal study[37], so a meta-analysis was not attempted for this category of study.

Carotid plaque and MMSE meta-analysis

Meta-analysis showed an association between presence of carotid plaque and impaired MMSE. The overall OR for the association between carotid plaque and impaired MMSE was 2.06 (95% CI: 1.16, 2.96) for presence of plaque, and with Z-score = 4.49, P < 0.01 (Figure 2).

Association between cIMT and MMSE

Meta-analysis showed an association between cIMT and impaired MMSE. The overall OR for the association between cIMT and impaired MMSE was 1.13 (95% CI: 1.04, 1.22) per 1mm cIMT, with Z-score = 24.7, P < 0.01 (Figure 3).

DISCUSSION

This systematic review examined and quantitated the evidence regarding a possible association between measures of atherosclerosis in the carotid arteries by ultrasound and generalised impairment of cognitive function assessed using the MMSE. From the limited studies available, our analysis showed there is some evidence of a cross-sectional association between carotid artery atherosclerosis and poorer MMSE, with the majority of studies finding an association. However a sizeable proportion of studies did not find evidence for an association between cIMT and MMSE score. These discrepancies could be due to inadequate sample size, sample selection, or the low sensitivity of MMSE for detection of mild cognitive impairment.

For peer review only

Risk factors for CVD are related with changes in cognitive funcation[56-59] and many factors could link carotid atherosclerosis with cognition. These include thromboembolic consequences of carotid disease, small vessel disease and cerebral perfusion abnormalities, shared genetic predisposition, or shared risk factors over the life course. A recent study that observed a relationship between cIMT and MMSE, memory and executive function, also found an association between cIMT and decrease in brain matter volumes and cerebral hypoperfusion[60].

There is little information regarding factors, such as age, gender, socioeconomic state, and pre-existing CVD, that might modify any relationship between carotid atherosclerosis and cognitive impairment. One study reported that there was a moderate association between presence of plaques and impaired MMSE in men, but no association in women[23]. In contrast, many studies failed to report evidence of sex differences in the association between carotid atherosclerosis and cognitive impairment,[61-63] although it was often not clear whether such an interaction was sought. Limited evidence suggests that socioeconomic position (SEP), which includes income, education, and social supports may modify the association between atherosclerosis and cognitive performance. In Whitehall study of UK-based civil servants, [28] an association between higher cIMT and poorer cognitive function was only observed in individuals classified as low SES. Another study done in USA, [45] reported that the association between cIMT and measures of cognition differed as a function of race and socioeconomic status.

For peer review only

Limitations

Our systematic review has several limitations. Restricting the search to only literature published in the English language only may have reduced the number of citations identified. The primary aim of some the studies reviewed was not to assess the association between atherosclerosis in carotid arteries and cognitive function. It is also worth noting that there was significant heterogeneity with regards to atherosclerosis measurements and scanning techniques and, most of studies reviewed did not perform a comprehensive carotid scan. The use of B-mode ultrasound to detect carotid atherosclerosis has well-recognised limitations[64 65] and more advanced ultrasound imaging including 3D ultrasound[66], or other imaging techniques, such as MRI[67] or PET[68], that better quantify and characterise vulnerable atherosclerosis may have advantages. Similarly, use of the MMSE which has several limitations, as discussed above, provides only a limited insight into the complex process of cognitive decline and use of a wider range of tools would be worthwhile.

CONCLUSION

This meta-analysis and systematic review provide some evidence in favour of an association between cIMT and cognitive function in cross-sectional studies. Estimates of the association between plaques and poor cognition in cross-sectional studies were too imprecise to draw firm conclusions. While evidence from studies of longitudinal associations between carotid atherosclerosis and cognitive function is limited.

For peer review only

Table 1A: Cross-sectional studies

Reference	Location	Sampling frame / Health conditions	Sample size	Age (years±SD)	Female (%)	Presence of Plaque (n)	cIMT measurement (mm±SD)	MMSE (score±SD)	Major relevant finding
(Auperin et al., 1996)	France	Community-based sample	Male: 521 Female: 753	65±3	60	Male: Yes (358) Female: Yes (217)	Male: 0.69±0.14 Female: 0.65±0.11	Male: 28.3±2 Female: 27.9±2.3	No evidence of an association between cIMT and MMSE in either sex. Association between presence of carotid plaques and impaired MMSE in men. No evidence of an association in women
(Watanabe et al., 2004)	Japan	Patients with vascular dementia (VaD) and age-matched controls	Control: 63 VaD: 37 AD: 34	C: 72±11 VaD: 75±8 AD: 76±9	0	Control: Yes (15) VaD: Yes (28) AD: Yes (18)	Control : 0.68±0.11 VaD: 0.94±0.2 AD: 0.85±0.14	Control : 26±3 VaD: 13±6 AD: 10±5	Comparing VaD patients with controls. MMSE was lower, cIMT was greater and carotid plaques more frequent

(Haley et al., 2007)	USA	Cardiology patients and volunteers	109	69.18±7.43	43	N/A	0.88±0.13	28.55±1.59		cIMT was not independently related to performance on measures of global cognitive functioning. Increased cIMT was significantly associated with poorer performance in the attention-executive-psychomotor domain independent of risk factors.
(Muller et al., 2007)	Netherlands	Community-based sample, age-stratified selection and further stratified by cardiovascular disease status	No CVD: 217 Subclinical CVD: 125 Prevalent CVD: 54	No CVD: 54±10.3 Subclinical CVD: 66.8±8.1 Prevalent CVD: 67.7±8.8	0	N/A	No CVD: 0.77±0.01 Subclinical CVD: 0.89±0.01 Prevalent CVD: 0.89±0.02	No CVD: >28 (n=100, 63%) SCVD: >28 (n=49, 31%) PCVD: >28 (n=11, 7%)	No CVD: ≤28 (n=117, 50%) SCVD: ≤28 (n=73, 31%) PCVD: ≤28 (n=43, 18%)	Association of cIMT with MMSE not presented. Thicker cIMT was associated with lower scores on memory functioning.

(Singh-Manoux et al., 2008)	UK	Occupational sample excluding individuals with stroke	High SES,M: 1190 High SES, F: 185 Intermediate SES, M: 1477 Intermediate SES, F:490 Low SES, M: 141 Low SES, F: 413	High SES, M: 62.32±5.61 High SES, F: 59.8±5.49 Intermediate SES, M: 60±71 Intermediate SES, F: 60±5.93 Low SES, M: 60.84±6.51 Low SES, F: 62±5.73	High SES: 17 Intermediate SES: 45 Low SES: 38	N/A	N/A	High SES, M: 0.8±0.16 High SES, F: 0.76±0.13 Intermediate SES, M: 0.79±0.16 Intermediate SES, F: 0.77±0.13 Low SES, M: 0.82±0.18 Low SES, F: 0.79±0.14	High SES, M: 28.9±1.19 High SES, F: 29.16±1.04 Intermediate SES, M: 28.71±1.31 Intermediate SES, F: 28.88±1.24 Low SES, M: 27.86±1.7 Low SES, F: 28.18±1.55	An inverse association between cIMT and cognition was observed only in the low SES group. Evidence that SES modifies the association between cIMT and cognition.
(Carlsson et al., 2009)	USA	Population-based sample	No CI: 1358 CI: 180	No CI: 74.8±6.6 CI:80±7.1	No CI: 62 CI: 54	N/A	N/A	No CI: 0.95±0.23 CI: 1.00 ± 0.25	No CI: 27.6 ± 1.7 CI: 21.1 ± 2.9	cIMT higher in people with cognitive impairment defined as MMSE <24 or proxy-reported dementia
(El-Kattan et al., 2009)	Egypt	Vascular surgery patients and age- sex-matched healthy controls / PAD (66.6%)	C: 10 PAD wo CVD: 10 PAD w CVD: 10	C: 53.9±9.55 PAD wo CVD: 54.8±8.96 PAD w CVD: 56.1±9.62	C: 20 PAD wo CVD: 10 PAD w CVD: 20	N/A	C: No (0) PAD wo CVD: Yes (1) PAD w CVD: Yes (5)	C: 0.7±0.1 PAD wo CVD: 0.9±0.2 PAD w CVD: 1.07±0.13	C: 30±0 PAD wo CVD: 28±1.4 PAD w CVD: 26±1.5	Patients with peripheral arterial disease had higher cIMT and lower MMSE compared to healthy controls

(Kearney-Schwartz et al., 2009)	France	Hypertensive patients with subjective memory complaints but excluding individuals with MMSW ≤24	198	69.3±6.2	53	N/A	Yes (50)	0.68±0.09	28.3±1.4	No evidence of an association between cIMT and impaired memory function implied, although data for MMSE or cognition not presented.
(Zhong et al., 2011)	USA	Population-based sample	2794	49±9.8	54	N/A	Yes (2665)	0.65±0.15	28.7±1.3	Higher Carotid IMT and plaque were associated with lower MMSE score
(Dias et al., 2012)	Brazil	Hypertensive patients and non-hypertensive, non-cognitively impaired controls identified from patients referred for investigation of high blood pressure. People with carotid plaques excluded.	Controls: 48<br>Hypertensive without cognitive impairment: 108<br>Hypertensive with cognitive impairment: 42	Controls: 44.5±7.9<br><br>Hypertensive without cognitive impairment: 55.75±8.6<br>Hypertensive with cognitive impairment: 63.3±9.3	C 62.5<br><br>H without 53.7<br>H with 60.5	N/A		Controls: 0.69±1<br>Hypertensive without cognitive impairment: 0.89±0.2<br>Hypertensive with cognitive impairment: 0.99±0.2	Controls: 27.77±4.4<br>Hypertensive without cognitive impairment: 28.44±1.3<br>Hypertensive with cognitive impairment: 22.02±2.4	Higher cIMT was associated with higher odds ratio of MMSE score ≤24

(Stefanova et al., 2012)	Serbia	Patients with VCD or AD / VCD: HYL (36%) HTN (29%) DM (11%) AD: HYL(62%) HTN (88%) DM (28%)	VCD: 237 AD: 197	VCD: 67.4±8.18 AD: 68.5±9.36	VCD: 52.3 AD: 49	N/A N/A	VCD: Yes (120) AD: Yes (155)	VCD: 1.11±0.2 AD: 1.15±0.18	VCD: 23.81±2.33 AD: 16±5.9	Higher cIMT and plaque type was correlated with poorer MMSE in VCD. No evidence of a correlation between MMSE and cIMT or plaque in AD group.
(Rogne et al., 2013)	Norway	Population-based sample / DM (1%) CHD (5.3%)	1,577	Median (range): 57 (52-61)	48	N/A	N/A	Median (range): 0.78 (0.69–0.89)	N/A	No evidence of association between cIMT and MMSE.
(Xiang et al., 2013)	China	Neurology patients / HTN (55%) DM (35%) CHD (10%)	No cognitive impairment: 1659 Cognitive impairment: 356	No cognitive impairment: 68.1±7.2 Cognitive impairment: 73.2±7.8	No cognitive impairment : 48 Cognitive impairment: 52	N/A N/A	Yes (1377) Yes (299)	No cognitive impairment: 0.76±0.14 Cognitive Impairment: 1.57±0.15	No cognitive impairment: ≥24 Cognitive impairment: <24	cIMT was associated with a higher odds ratio of cognitive impairment (MMSE <24)
(Nagai et al., 2014)	Japan	Patients at high risk of cardiovascular disease / HTN (75.9%) DM (13.9%)	201	79.9±6.4	75	N/A	N/A	1.03±0.3	25.8±4.69	Compared with those with both low blood pressure variability and low IMT, patients with high blood pressure variability and high IMT had lower MMSE score

										or higher prevalence of cognitive impairment (based on a MMSE score ≤24)
(Yano et al., 2014)	Japan	Hypertensive patients / HTN (100%)	587	73±8.1	59.0	N/A	N/A	NCD, median(range): 0.87(0.75-1.01) CD, median(range): 0.96 (0.82-1.06)	NCD, median(range): 28(27-29) CD, median(range): 24(22-24)	cIMT higher in patients with cognitive impairment (defined as MMSE score ≤ 24)
(Liu et al., 2016)	China	Community-based sample / HTN (71%) DM (13%)	384	84.65±2.3	67	N/A	N/A	1.45 ± 0.30	Median (range): 24 (22-24)	cIMT inversely related to MMSE score.
(Alhusaini et al., 2018)	Scotland	Population-based cohort HTN (47.3%) DM (10.1%)	518	72.7±0.73	46.6	N/A	Yes (77)	0.84±0.17	28.8±1.3	Data relating cIMT to MMSE not presented. Carotid stenosis was related to lower fluid intelligence.
(Matsumoto et al., 2018)	Japan	Patients undergoing health screening	176	NCI: 64.6 ±9.6 CI: 67.7±12.3	NCI: 59.1 CI: 37	N/A	N/A	NCI :1.7±0.7 CI: 2.0±1	N/A	No evidence of an association between cIMT and MMSE in a model adjusted for sex, age and years of education. cIMT higher in patients

										with cognitive impairment.
(Muela et al., 2018)	Brazil	Patients. Individuals with cerebrovascular disease excluded / HTN (67%)	Normotensive: 69 Hypertensive stage 1: 83 Hypertensive stage 2: 59	Normotensive: 52.1±13.9 Hypertensive stage 1: 52.1±13 Hypertensive stage 2: 51.3±10.1	Normotensive: 55.1 Hypertensive stage 1: 55.8 Hypertensive stage 2: 53.6	N/A	N/A	Normotensive: 0.7±0.1 Hypertensive stage 1: 0.8±0.1 Hypertensive stage 2: 0.8±0.1	Normotensive: 28.03±1.92 Hypertensive stage 1: 27.43±2.01 Hypertensive stage 2: 26.66±2.07	cIMT showed a weak negative correlation with MMSE consistent with null. Negative correlations of cIMT with other cognitive measures were observed.
(Mworozi et al., 2019)	Uganda	Community-based study / HTN (25%) DM (5%)	210	69.9±7.76	71.4	N/A	Yes (45)	0.9±0.2	N/A	Presence of carotid artery plaque was associated with abnormal cognitive function defined as MMSE ≤24.

For peer review only

Table 1B. Cross-sectional plus longitudinal studies

Reference	Location	Sampling frame / Health conditions	Sample size	Age (years±SD)	Female (%)	Follow-up (years)	Presence of Plaque (n)	cIMT measurement (mm±SD)	MMSE (score±SD)	Major relevant finding
(Komulainen et al., 2007)	Finland	Population-based sample	91 (CS) 47 (LT)	63.5±3.1 (CS) 75±3.2 (LT)	100	12	N/A	1.02±0.26 (Cross-sectional) 1.25±0.33 (Longitudinal)	28.9±1.6 (Cross-sectional) 26.4±2.01 (Longitudinal)	There was no evidence of an association between IMT and MMSE score cross-sectionally or after 12-year follow-up, although confidence intervals of estimates were very wide. Associations between high cIMT and poor memory were seen both cross-sectionally and longitudinally.
(Carcaillon et al., 2015)	France	Population-based sample	5798	73.4±4.8	60.5	7	Yes (3038)	0.71±0.12	N/A	Association between baseline carotid plaques and incident dementia. No association between cIMT and incident dementia

(Kawasaki et al., 2016)	Japan	Population-based sample	494	Median (range): 87.2 (86.1–88.7)	55	3	Yes (328)	N/A	N/A	Presence of higher carotid artery plaque score was associated with lower MMSE scores. Weak evidence of an association between plaque score and increase rate of decline in MMSE compatible with null.

For peer review only

Table 1C. Longitudinal studies

Reference	Location	Sampling frame / Health conditions	Sample size	Age, year (mean±SD)	Female, %	Mean follow-up (years)	Presence of Plaque (n)	cIMT measurement (mm±SD)	MMSE (score±SD)	Major relevant finding
(Silvestrini et al., 2009)	Italy	Dementia clinic outpatients	66	72.7±6.1	55	1	Yes (52)	1.1±0.3	18.1±3	Higher baseline cIMT was associated with greater decline in MMSE scores
(Viticchi et al., 2012)	Italy	Patients referred to Dementia clinic / HYL (55.6%) HTN (65%) DM (29.1%)	IC: 96 DE: 21	IC: 75±6 DE: 77.2±4.28	IC:44.8 DE: 66.7	1	IC: Yes (30) DE: Yes (9)	IC: 0.92±0.15 DE: 1.04±0.16	IC 27.14±1.76 DE: 27±1.56	Patients with abnormal cIMT (>1mm) had higher odds ratio of progressing from mild cognitive impairment to AD dementia
(Zhong et al., 2012)	USA	Population-based sample	1311	66.8	59	5 and 10	Yes (682)	0.86±0.21	N/A	Higher cIMT was associated with higher risk of incident cognitive impairment (MMSE <24). Plaque was not associated with incident

										cognitive impairment or cognitive test performance.
(Buratti et al., 2014)	Italy	Patients with high vascular risk	159 (NcIMT: 68 PcIMT: 91)	NcIMT:69.7±3.6 PcIMT:70.2±3.8	NcIMT:39.7 PcIMT:37.4	3	NcIMT:Yes (68) PcIMT:Yes (91)	NcIMT: < 1 PcIMT: ≥ 1	NcIMT BL: 26.75±1.05 NcIMT:24.86±1.85 (3 y from BL) PcIMT BL: 27±1.25 PcIMT FU: 25.27±1.86 (3 y from BL)	No evidence of an association between cIMT and change in MMSE score
(Buratti et al., 2015)	Italy	Patients referred to Dementia clinic	MCI: 300 AD: 106	MCI, meadian (range): 72 (67-76) AD, meadian (range): 72 (68-76)	MCI: 37 AD: 43	1	MCI: Yes (114) AD: Yes (53)	N/A	MCI: 27±1 AD: 27±1	cIMT >1mm and plaque associated with increased odds ratio of development of Alzheimer disease over 12-month follow-up
(Wendell et al., 2016)	USA	Community-based sample	1696	46.9±9.3	55	4	N/A	0.69±0.13	N/A	Weak negative association between cIMT and MMSE in fully adjusted models, consistent with null.

(Falsetti et al., 2018)	Italy	Patients from neurological clinic with mild to moderate cognitive impairment	310	76.86±7.49	62.9	2	N/A	pNVAF- : 0.97±0.2 pNVAF+ : 1.11±0.16	pNVAF- : 18.62±5.22 pNVAF+ : 15.8±5.17	cIMT weakly associated with progression to probably Alzheimer Disease but results compatible with null. Patients with non-valvular atrial fibrillation (pNVAF+) had lower MMSE and higher cIMT compared to pNVAF-.
(Rouch et al., 2018)	N/A	Consecutive patients attending a memory clinic	363	75.2±7	65.6	4.5	NCTD: Yes (50) CTD: Yes (40)	NCTD: 0.85±0.13 CTD: 0.82±0.13	NCTD: 28.3±1.6 CTD: 26.6±2.6	Higher cIMT and plaque associated with progression from MCI to dementia based on MMSE score.

Abbreviations: AD:Alzheimer's disease, cIMT :carotid intima-media thickness,CD:cognitive dysfunction, ICA:internal carotid artery, M:men, MMSE :Mini Mental State Examination, MCI:mild cognitive impairment, pNVAF: permanent non-valvular atrial fibrillation, VaD:vascular dementia, VCD:vascular cognitive decline.

For peer review only

Table 2. Exposure measures of the included papers.

Reference	Ultrasound machine / transducer	IMT Measurement sites	Scanning techniques	Intra-observer reproducibility, blinded
(Auperin et al., 1996)	Aloka SSD-650 7.5-MHz transducer	Bilateral CCA, including the carotid bifurcation, and the first 2 cm of the ICA	Scanned longitudinally and transversely to assess the presence of plaques	Inter-reader agreement regarding the presence of plaques was excellent (κ coefficient=.90).
(Watanabe et al., 2004)	PLE-705S, Toshiba 7.5-MHz linear-array transducer	Bilateral CCA including the carotid bifurcation and from 10 mm below the bifurcation,	Anterolateral and posterolateral angles of CCA with beam focused on the far wall	All measurements were determined by the same examiner, who was blinded to clinical history or risk factor profile.
(Komulainen et al., 2007)	10-MHz transducer	Bilateral far wall of CCA and bifurcation	N/A	sonographers were blinded to the randomization status of the study participants
(Haley et al., 2007)	Agilent 5500 7.5-MHz transducer	Left, far wall CCA 1 cm proximal to the carotid bulb	N/A	N/A
(Muller et al., 2007)	Acuson Aspen	Bilateral distal CCA	N/A	the intra-class

For peer review only - http://bmjopen.bmj.com/site/about/guidelines.xhtml

	7.5 MHz linear array transducer			correlation coefficient (ICC) for repeated IMT-measurement was 84%
(Singh-Manoux et al., 2008)	Aloka 5500 7.5-MHz transducer	Bilateral CCA	Longitudinal images triggered on the R-wave of the ECG	N/A
(Silvestrini et al., 2009)	Aspen color-coded duplex sonography	Bilateral CCA	of IMT taken as the thickest plaque-free region on the near and far walls in longitudinal images	N/A
(Carlsson et al., 2009)	AU4 Biosound 7.5MHz transducer	Bilateral, distal CCA, bifurcation, and the proximal portion of ICA	near and far walls scanned in each vessel segment (total of 12 sites)	N/A
(El-Kattan et al., 2009)	Siemens Elegra 7.5 MHz transducer	No details provided	Arteries assessed using longitudinal views and checked for the state of arterial wall and the presence of thrombi	N/A
(Kearney-Schwartz et al., 2009)	ATL Apogee 800+ 7.5-MHz linear array transducer	Bilateral CCA	Arteries assessed using longitudinal views	N/A

For peer review only - http://bmjopen.bmj.com/site/about/guidelines.xhtml

For peer review only

(Zhong et al., 2011)	Biosound AU4<br>7.5MHz transducer	Bilateral, near and far walls of CCA, bifurcation and ICA	N/A	The reproducibility of IMT and plaque assessment was good. In a 10% sample (n=280) of participant scans that were re-graded the mean difference in IMT was 0.0019 mm and kappa statistics for plaque assessment ranged from 0.58 (ICA) to 0.71(bifurcation) with 97.3% agreement within ±1 for the number of sites with plaque.
(Dias et al., 2012)	Philips HD 11 XE<br><br>7–12 MHz linear transducer	Bilateral CCA	The intima-media thickness was measured in near and far walls over a 1-cm segment of the artery located approximately 0.5 cm below the carotid-artery bulb and considered not to contain any plaque	The variability between IMT measurements less than 2%,; analysis performed by a physician blinded to the patient's clinical

For peer review only - http://bmjopen.bmj.com/site/about/guidelines.xhtml

				data
(Zhong et al., 2012)	Biosound AU4 7.5MHz transducer	Bilateral CCA	Near and far walls	The reproducibility of IMT and plaque assessment was good. The mean inter-grader difference in IMT was 0.03 mm; and for plaque, the kappa coefficient was 0.76 and percent agreement was 90%
(Viticchi et al., 2012)	iU22 Philips 7.5MHz transducer	Bilateral, distal segment of CCA	Measurement of near and far wall IMT on longitudinal image of 1.5cm segment of CCA that precedes the carotid bifurcation. Measurement made with an automated system at the thickest point where there were no plaques	N/A
(Stefanova et al., 2012)	Aloka ProSound ALPHA 10 13 MHz linear multifrequency transducer	Far wall of CCA, ICA during diastole	during diastole measurements were done in a supine position with head elevated up to 45°, and tilted to the either side for 30°, depending on the side examined	The inter-rater correlation reliability assessed for 50 randomly selected patients from both groups was excellent (0.932), Physicians

For peer review only

				that performed ultrasound examinations were blinded to clinical data
(Rogne et al., 2013)	F/U (GE Vivid 7) 12-MHz transducer	Right, far and near wall CCA, bulb	measurement of IMT was performed in 10-mm segments of the far and near wall of the common carotid artery in the most proximal 10-mm segment of the bulb. The CCA, the bifurcation and the internal carotid artery were examined for plaque presence.	N/A
(Xiang et al., 2013)	Hitachi Aloka SSD-650 7.5 and 10 MHz transducer	Bilateral, far wall CCA	Measurement of far wall cIMT in longitudinal B-mode images of the CCA, the carotid bifurcations, and the first 2 cm of the ICA at plaque-free sites	N/A
(Nagai et al., 2014)	SSA-660A Xario; Toshiba 7.5-MHz transducer with border detection	Bilateral, far wall CCA	CCA scanned bilaterally in longitudinal and transverse projections. The image was focused on the far wall of the artery	N/A
(Yano et al., 2014)	7.5-MHz transducer	Bilaterally at CCA, the bulb, and ICA	CCA, the bulb, and ICA measured from both transverse and longitudinal orientations, Region with the thickest IMT measured.	Coefficient of variation within 10%. Scan performed blind to patient's data
(Buratti et al., 2014)	iU22 Philips	Bilateral CCA	N/A	inter-reader correlation coefficient of 0.88

For peer review only - http://bmjopen.bmj.com/site/about/guidelines.xhtml

(Buratti et al., 2015)	iU22 Philips 7.5MHz transducer	Bilateral CCA	A longitudinal image of the distal segment of common carotid arteries was taken, and the measurement was obtained with an automatic system at the thickest point where there were no plaques on the proximal and distal wall	N/A
(Carcaillon et al., 2015)	N/A	Bilateral CCA, bulb, and ICA	scanned longitudinally and transversally to detect plaques.	N/A
(Kawasaki et al., 2016)	Hitachi EUB- 525 10-MHz linear transducer	Bilateral CCA, ICA	N/A	All examinations were performed by a single physician blinded to the subject's clinical information
(Liu et al., 2016)	Vivid i, GE Medical Systems 7.5-MHz linear array transducer	Bilateral CCA	Three B-mode images were obtained using anterior, lateral, and medial angles. Maximum IMT in the right or left CCA used.	Scanning done by certified ultrasonographer who was unaware of the subjects' clinical details
(Wendell et al., 2016)	Acuson CV 70, Siemens standard transducer 5.OL45	Left CCA	Far wall IMT measured over a region 1.5 cm proximal to the carotid bifurcation	Intraobserver correlation between repeated carotid IMT measurements on 10 participants was 0.96 (p <0.001).

For peer review only

For peer review only - http://bmjopen.bmj.com/site/about/guidelines.xhtml

(Rouch et al., 2018)	Sigma 110 Kontron device<br>7.5 MHz transducer	Bilateral CCA	Near and far wall IMT measured in longitudinal images. Longitudinal and transverse images examined for plaques	N/A
(Alhusaini et al., 2018)	Siemens<br>7.5 MHz transducer	Far walls of CCA, and bulb*	Far wall cIMT measured as the mean of 3 caliper measurements over a 1cm-long segment of the CCA and carotid bulb	N/A
(Matsumoto et al., 2018)	Xario SSA-660A; TOSHIBA	Bilateral, far wall CCA, ICA	far wall maximum cIMT of the bilateral CCA,ICA measured at end-diastole in longitudinal images	Certified sonographers who were blinded with cognitive test results carried out the carotid ultrasonography
(Muela et al., 2018)	Sigma 44 Kontrom<br>7.5- MHz transducer	Left CCA	Near and far wall cIMT was measured at the thickest point of the distal CCA, not including plaques using a computer program	an experienced observer who were blinded with Clinical condition carried out the carotid ultrasonography
(Falsetti et al., 2018)	iU22 Philips	Bilateral CCA	cIMT measurements made on 1.5 cm segment of CCA artery preceding carotid bifurcation in a longitudinal image using a semiautomatic system	N/A
(Mworozi et al., 2019)	Shantou Institute of ultrasonic instrument 5300	CCA, ICA*	Participants scanned in both supine and semi recumbent positions, with the head slightly hyperextended and rotated 45 degrees away	N/A

For peer review only - http://bmjopen.bmj.com/site/about/guidelines.xhtml

For peer review only

	5–12 MHz frequency linear transducer		from the side being examined	

Abbreviations: (ICA) internal carotid artery, (ECA) the external carotid artery, (CCA) common carotid artery , (CIMT) carotid intima-media thickness , (N/A) not applicable. *not clear whether measured bilaterally.

For peer review only

For peer review only - http://bmjopen.bmj.com/site/about/guidelines.xhtml

Contributors: RA, AH, RH and MR developed the idea and designed the study protocol. RA, LA, and SS designed and wrote the search strategy and data extraction. RA, MA, and SS planned the statistical analysis and results interpretation. NC, RA, RH, MR, and AH provided critical insights. All authors have approved and contributed to the final written manuscript.

Funding:

RA is supported by a PhD scholarship grant from King Abdulaziz University.

NC received support from the National Institute for Health Research University College London Hospitals Biomedical Research Centre and works in a unit that receives support from the UK Medical Research Council.

AH receives support from the British Heart Foundation, the Economic and Social Research Council (ESRC), the Horizon 2020 Framework Programme of the European Union, the National Institute on Aging, the National Institute for Health Research University College London Hospitals Biomedical Research Centre, the UK Medical Research Council, the Wellcome Trust, and works in a unit that receives support from the UK Medical Research Council.

MR is supported by UK Medical Research Council grants MC_UU_12019/1 (Enhancing NSHD) and /3 (Mental Ageing).

Ethical approval statement: This study is for conducting a systematic review and meta-analysis hence no ethical approval is required.

Competing interests: None declared.

Patient consent for publication: Not required.

Provenance and peer review: Not commissioned, externally peer reviewed.

Data availability: Data are available upon reasonable request.

For peer review only

REFERENCES

1. Naghavi M, Abajobir AA, Abbafati C, et al. Global, regional, and national age-sex specific mortality for 264 causes of death, 1980–2016: a systematic analysis for the Global Burden of Disease Study 2016. *The Lancet* 2017;390(10100):1151-210. doi: 10.1016/s0140-6736(17)32152-9
2. McGill HC, Jr., McMahan CA, Gidding SS. Preventing heart disease in the 21st century: implications of the Pathobiological Determinants of Atherosclerosis in Youth (PDAY) study. *Circulation* 2008;117(9):1216-27. doi: 10.1161/CIRCULATIONAHA.107.717033
3. Sobieszczyk P, Beckman J. Carotid Artery Disease. *Circulation* 2006;114(7):e244-e47. doi: 10.1161/circulationaha.105.542860
4. Song P, Fang Z, Wang H, et al. Global and regional prevalence, burden, and risk factors for carotid atherosclerosis: a systematic review, meta-analysis, and modelling study. *The Lancet Global Health* 2020;8(5):e721-e29. doi: 10.1016/s2214-109x(20)30117-0
5. Davis PH, Dawson JD, Riley WA, et al. Carotid intimal-medial thickness is related to cardiovascular risk factors measured from childhood through middle age: The Muscatine Study. *Circulation* 2001;104(23):2815-9. doi: 10.1161/hc4601.099486 [published Online First: 2001/12/06]
6. Breteler MMB, Claus JJ, Grobbee DE, et al. Cardiovascular disease and distribution of cognitive function in elderly people: The Rotterdam study. *British Medical Journal* 1994;308(6944):1604-08.
7. Stein JH, Korcarz CE, Hurst RT, et al. Use of Carotid Ultrasound to Identify Subclinical Vascular Disease and Evaluate Cardiovascular Disease Risk: A Consensus Statement from the American Society of Echocardiography Carotid Intima-Media Thickness Task Force Endorsed by the Society for Vascular Medicine. *Journal of the American Society of Echocardiography* 2008;21(2):93-111. doi: 10.1016/j.echo.2007.11.011
8. O'Leary DH, Bots ML. Imaging of atherosclerosis: carotid intima–media thickness. *European Heart Journal* 2010;31(14):1682-89. doi: 10.1093/eurheartj/ehq185
9. Touboul PJ, Hennerici MG, Meairs S, et al. Mannheim carotid intima-media thickness and plaque consensus (2004-2006-2011). An update on behalf of the advisory board of the 3rd, 4th and 5th watching the risk symposia, at the 13th, 15th and 20th European Stroke Conferences, Mannheim, Germany, 2004, Brussels, Belgium, 2006, and Hamburg, Germany, 2011. *Cerebrovasc Dis* 2012;34(4):290-96. doi: 10.1159/000343145 [published Online First: 2012/11/01]
10. Chaytor N, Schmitter-Edgecombe M. The ecological validity of neuropsychological tests: a review of the literature on everyday cognitive skills. *Neuropsychol Rev* 2003;13(4):181-97. doi: 10.1023/b:nerv.0000009483.91468.fb [published Online First: 2004/03/06]
11. Roy E. Cognitive Function. In: Gellman MD, Turner JR, eds. Encyclopedia of Behavioral Medicine. New York, NY: Springer New York 2013:448-49.
12. Knopman DS, Petersen RC. Mild cognitive impairment and mild dementia: a clinical perspective. *Mayo Clin Proc* 2014;89(10):1452-9. doi: 10.1016/j.mayocp.2014.06.019 [published Online First: 2014/10/06]

For peer review only - http://bmjopen.bmj.com/site/about/guidelines.xhtml

13. Faber RA. The Neuropsychiatric Mental Status Examination. *SEMIN NEUROL* 2009;29(3):185-93. doi: 10.1055/s-0029-1223874
14. Tsoi KKF, Chan JYC, Hirai HW, et al. Cognitive Tests to Detect Dementia: A Systematic Review and Meta-analysis. *JAMA Internal Medicine* 2015;175(9):1450-58. doi: 10.1001/jamainternmed.2015.2152
15. Mitchell AJ. A meta-analysis of the accuracy of the mini-mental state examination in the detection of dementia and mild cognitive impairment. *Journal of Psychiatric Research* 2009;43(4):411-31. doi: https://doi.org/10.1016/j.jpsychires.2008.04.014
16. Patnode CD, Perdue LA, Rossom RC, et al. Screening for Cognitive Impairment in Older Adults: An Evidence Update for the U.S. Preventive Services Task Force. Rockville (MD): Agency for Healthcare Research and Quality 2020.
17. Meyers CA, Wefel JS. The use of the mini-mental state examination to assess cognitive functioning in cancer trials: no ifs, ands, buts, or sensitivity. *J Clin Oncol* 2003;21(19):3557-8. doi: 10.1200/JCO.2003.07.080 [published Online First: 2003/08/13]
18. van Oijen M, Jan de Jong F, Witteman JC, et al. Atherosclerosis and risk for dementia. *Annals of Neurology: Official Journal of the American Neurological Association and the Child Neurology Society* 2007;61(5):403-10.
19. Romero JR, Beiser A, Seshadri S, et al. Carotid artery atherosclerosis, MRI indices of brain ischemia, aging, and cognitive impairment: the Framingham study. *Stroke* 2009;40(5):1590-6. doi: 10.1161/STROKEAHA.108.535245 [published Online First: 2009/03/07]
20. Moher D, Liberati A, Tetzlaff J, et al. Preferred reporting items for systematic reviews and meta-analyses: the PRISMA statement. *PLoS Med* 2009;6(7):e1000097.
21. Grant RL. Converting an odds ratio to a range of plausible relative risks for better communication of research findings. *Br Med J* 2014;348(jan24 1):f7450-f50. doi: 10.1136/bmj.f7450
22. Wells G, Shea B, O'Connell D, et al. The Newcastle–Ottawa Scale (NOS) for Assessing the Quality of Non-Randomized Studies in Meta-Analysis. , 2000.
23. Auperin A, Berr C, Bonithon-Kopp C, et al. Ultrasonographic assessment of carotid wall characteristics and cognitive functions in a community sample of 59- to 71-year-olds. The EVA Study Group. *Stroke* 1996;27(8):1290-5. doi: 10.1161/01.str.27.8.1290 [published Online First: 1996/08/01]
24. Muller M, Grobbee DE, Aleman A, et al. Cardiovascular disease and cognitive performance in middle-aged and elderly men. *Atherosclerosis* 2007;190(1):143-49. doi: 10.1016/j.atherosclerosis.2006.01.005
25. Watanabe T, Koba S, Kawamura M, et al. Small dense low-density lipoprotein and carotid atherosclerosis in relation to vascular dementia. *Metabolism, clinical and experimental* 2004;53(4):476-82. doi: 10.1016/j.metabol.2003.11.020
26. Komulainen P, Kivipelto M, Lakka TA, et al. Carotid intima-media thickness and cognitive function in elderly women: A population-based study. *Neuroepidemiology* 2007;28(4):207-13.
27. Haley AP, Forman DE, Poppas A, et al. Carotid artery intima-media thickness and cognition in cardiovascular disease. *International Journal of Cardiology* 2007;121(2):148-54.
28. Singh-Manoux A, Britton A, Kivimaki M, et al. Socioeconomic status moderates the association between carotid intima-media thickness and cognition in midlife: Evidence from the Whitehall II study. *Atherosclerosis* 2008;197(2):541-48.
29. Carlsson CM, Nondahl DM, Klein BE, et al. Increased atherogenic lipoproteins are associated with cognitive impairment: effects of statins and subclinical

For peer review only - http://bmjopen.bmj.com/site/about/guidelines.xhtml

atherosclerosis. *Alzheimer Dis Assoc Disord* 2009;23(1):11-7. doi: 10.1097/wad.0b013e3181850188 [published Online First: 2009/03/10]

30. El-Kattan MM, Zakaria YA, El-Fayomy N, et al. Peripheral arterial disease and cognition. *Egyptian Journal of Neurology, Psychiatry and Neurosurgery* 2009;46(2):311-22.
31. Kearney-Schwartz A, Rossignol P, Bracard S, et al. Vascular Structure and Function Is Correlated to Cognitive Performance and White Matter Hyperintensities in Older Hypertensive Patients With Subjective Memory Complaints. *Stroke* 2009;40(4):1229-36.
32. Silvestrini M, Gobbi B, Pasqualetti P, et al. Carotid atherosclerosis and cognitive decline in patients with Alzheimer's disease. *Neurobiology of Aging* 2009;30(8):1177-83.
33. Zhong W, Cruickshanks KJ, Huang GH, et al. Carotid atherosclerosis and cognitive function in midlife: the Beaver Dam Offspring Study. *Atherosclerosis* 2011;219(1):330-3. doi: 10.1016/j.atherosclerosis.2011.07.013 [published Online First: 2011/08/13]
34. Dias EDM, Giollo LT, Martinelli DD, et al. Carotid intima-media thickness is associated with cognitive deficiency in hypertensive patients with elevated central systolic blood pressure. *Cardiovascular Ultrasound* 2012;10(1)
35. Stefanova E, Pavlovic A, Jovanovic Z, et al. Vascular risk factors in Alzheimer's disease - preliminary report. *J Neurol Sci* 2012;322(1-2):166-9. doi: 10.1016/j.jns.2012.07.065 [published Online First: 2012/09/04]
36. Viticchi G, Falsetti L, Vernieri F, et al. Vascular predictors of cognitive decline in patients with mild cognitive impairment. *Neurobiology of Aging* 2012;33(6):1127e1-27e9.
37. Zhong W, Cruickshanks KJ, Schubert CR, et al. Carotid atherosclerosis and 10-year changes in cognitive function. *Atherosclerosis* 2012;224(2):506-10. doi: 10.1016/j.atherosclerosis.2012.07.024 [published Online First: 2012/08/03]
38. Rogne SO, Solbu MD, Arntzen KA, et al. Albuminuria and carotid atherosclerosis as predictors of cognitive function in a general population. *European Neurology* 2013;70(5-6):340-8.
39. Xiang J, Zhang T, Yang QW, et al. Carotid artery atherosclerosis is correlated with cognitive impairment in an elderly urban Chinese non-stroke population. *Journal of Clinical Neuroscience* 2013;20(11):1571-75.
40. Buratti L, Balucani C, Viticchi G, et al. Cognitive deterioration in bilateral asymptomatic severe carotid stenosis. *Stroke* 2014;45(7):2072-77.
41. Nagai M, Hoshide S, Nishikawa M, et al. Visit-to-visit blood pressure variability in the elderly: associations with cognitive impairment and carotid artery remodeling. *Atherosclerosis* 2014;233(1):19-26. doi: 10.1016/j.atherosclerosis.2013.11.071 [published Online First: 2014/02/18]
42. Yano Y, Bakris GL, Inokuchi T, et al. Association of cognitive dysfunction with cardiovascular disease events in elderly hypertensive patients. *J Hypertens* 2014;32(2):423-31. doi: 10.1097/HJH.0000000000000025 [published Online First: 2013/12/20]
43. Carcaillon L, Plichart M, Zureik M, et al. Carotid plaque as a predictor of dementia in older adults: the Three-City Study. *Alzheimer's & Dementia* 2015;11(3):239-48.
44. Liu Z, Zhao Y, Wang X, et al. Low carotid artery wall shear stress is independently associated with brain white-matter hyperintensities and cognitive impairment in older patients. *Atherosclerosis* 2016;247:78-86. doi: 10.1016/j.atherosclerosis.2016.02.003 [published Online First: 2016/02/13]

For peer review only - http://bmjopen.bmj.com/site/about/guidelines.xhtml

45. Wendell CR, Waldstein SR, Evans MK, et al. Subclinical carotid atherosclerosis and neurocognitive function in an urban population. *Atherosclerosis* 2016;249:125-31. doi: 10.1016/j.atherosclerosis.2016.04.009 [published Online First: 2016/04/20]
46. Alhusaini S, Karama S, Nguyen TV, et al. Association between carotid atheroma and cerebral cortex structure at age 73 years. *Ann Neurol* 2018;84(4):576-87. doi: 10.1002/ana.25324 [published Online First: 2018/09/05]
47. Falsetti L, Viticchi G, Buratti L, et al. Interactions between Atrial Fibrillation, Cardiovascular Risk Factors, and ApoE Genotype in Promoting Cognitive Decline in Patients with Alzheimer's Disease: A Prospective Cohort Study. *Journal of Alzheimer's Disease* 2018;62(2):713-25.
48. Matsumoto L, Suzuki K, Mizuno Y, et al. Association of subclinical carotid atherosclerosis with immediate memory and other cognitive functions. *Geriatrics & gerontology international* 2018;18(1):65-71.
49. Muela HCS, Costa-Hong VA, Yassuda MS, et al. Higher arterial stiffness is associated with lower cognitive performance in patients with hypertension. *Journal of Clinical Hypertension* 2018;20(1):22-30.
50. Rouch L, Cestac P, Sallerin B, et al. Pulse wave velocity is associated with greater risk of dementia in mild cognitive impairment patients. *Journal of Hypertension* 2018;36 (Supplement 3):e340.
51. Mworozi K, Ameda F, Byanyima RK, et al. Carotid artery plaque detected on ultrasound is associated with impaired cognitive state in the elderly: A population-based study in Wakiso district, Uganda. *Journal of Clinical Neuroscience* 2019;68:194-200.
52. Kawasaki M, Arai Y, Hirata T, et al. Carotid atherosclerosis, cytomegalovirus infection, and cognitive decline in the very old: a community-based prospective cohort study. *Age* 2016;38(2):1-13.
53. Wendell CR, Zonderman AB, Metter EJ, et al. Carotid intimal medial thickness predicts cognitive decline among adults without clinical vascular disease. *Stroke* 2009;40(10):3180-5. doi: 10.1161/STROKEAHA.109.557280 [published Online First: 2009/08/01]
54. Buratti L, Balestrini S, Altamura C, et al. Markers for the Risk of Progression from Mild Cognitive Impairment to Alzheimer's Disease. *Journal of Alzheimer's Disease* 2015;45(3):883-90.
55. Mworozi K, Ameda F, Byanyima RK, et al. Carotid artery plaque detected on ultrasound is associated with impaired cognitive state in the elderly: A population-based study in Wakiso district, Uganda. *J Clin Neurosci* 2019;68:194-200. doi: 10.1016/j.jocn.2019.06.011 [published Online First: 2019/07/16]
56. Breteler MM. [New York, NY] :.
57. Grodstein F. Cardiovascular risk factors and cognitive function. *Alzheimer's & dementia* 2007;3(2):S16-S22. doi: 10.1016/j.jalz.2007.01.001
58. Skoog I. Status of Risk Factors for Vascular Dementia. *Neuroepidemiology* 1998;17(1):2-9. doi: 10.1159/000026147
59. Lai MMY, Sharman MJ, Ames DJ, et al. Relationship of established cardiovascular risk factors and peripheral biomarkers on cognitive function in adults at risk of cognitive deterioration. *Journal of Alzheimer's disease* 2020;74(1):163-71. doi: 10.3233/JAD-190953
60. Zheng K, Qian Y, Lin T, et al. Carotid intima–media thickness relative to cognitive impairment in dialysis patients, and their relationship with brain volume and cerebral small vessel disease. *Therapeutic Advances in Chronic Disease* 2020;11:2040622320953352. doi: 10.1177/2040622320953352

For peer review only - http://bmjopen.bmj.com/site/about/guidelines.xhtml

61. Van den Berg JF, Dogge B, Kist N, et al. Gender Differences in Cognitive Functioning in Older Alcohol-Dependent Patients. *SUBST USE MISUSE* 2017;52(5):574-80. doi: 10.1080/10826084.2016.1245341
62. Aartsen MJ, Martin M, Zimprich D. Gender Differences in Level and Change in Cognitive Functioning: Results from the Longitudinal Aging Study Amsterdam. *Gerontology* 2004;50(1):35-8.
63. Novella R, Olivera J. Gender Differences in Cognitive Abilities Among the Elderly Poor of Peru. *Economía , 42 (83) pp 95-109 (2019)* 2019
64. Touboul PJ, Hennerici MG, Meairs S, et al. Mannheim carotid intima-media thickness and plaque consensus (2004-2006-2011). An update on behalf of the advisory board of the 3rd, 4th and 5th watching the risk symposia, at the 13th, 15th and 20th European Stroke Conferences, Mannheim, Germany, 2004, Brussels, Belgium, 2006, and Hamburg, Germany, 2011. *Cerebrovasc Dis* 2012;34(4):290-6. doi: 10.1159/000343145 [published Online First: 2012/11/07]
65. Stein JH, Korcarz CE, Hurst RT, et al. Use of carotid ultrasound to identify subclinical vascular disease and evaluate cardiovascular disease risk: a consensus statement from the American Society of Echocardiography Carotid Intima-Media Thickness Task Force. Endorsed by the Society for Vascular Medicine. *J Am Soc Echocardiogr* 2008;21(2):93-111. doi: 10.1016/j.echo.2007.11.011
66. Cires-Drouet RS, Mozafarian M, Ali A, et al. Imaging of high-risk carotid plaques: ultrasound. *Semin Vasc Surg* 2017;30(1):44-53. doi: 10.1053/j.semvascsurg.2017.04.010
67. Gupta A, Baradaran H, Schweitzer AD, et al. Carotid Plaque MRI and Stroke Risk. *Stroke* 2013;44(11):3071-77. doi: 10.1161/strokeaha.113.002551
68. Piri R, Gerke O, Høilund-Carlsen PF. Molecular imaging of carotid artery atherosclerosis with PET: a systematic review. *Eur J Nucl Med Mol Imaging* 2020;47(8):2016-25. doi: 10.1007/s00259-019-04622-y

For peer review only

For peer review only - http://bmjopen.bmj.com/site/about/guidelines.xhtml

For peer review only

For peer review only - http://bmjopen.bmj.com/site/about/guidelines.xhtml

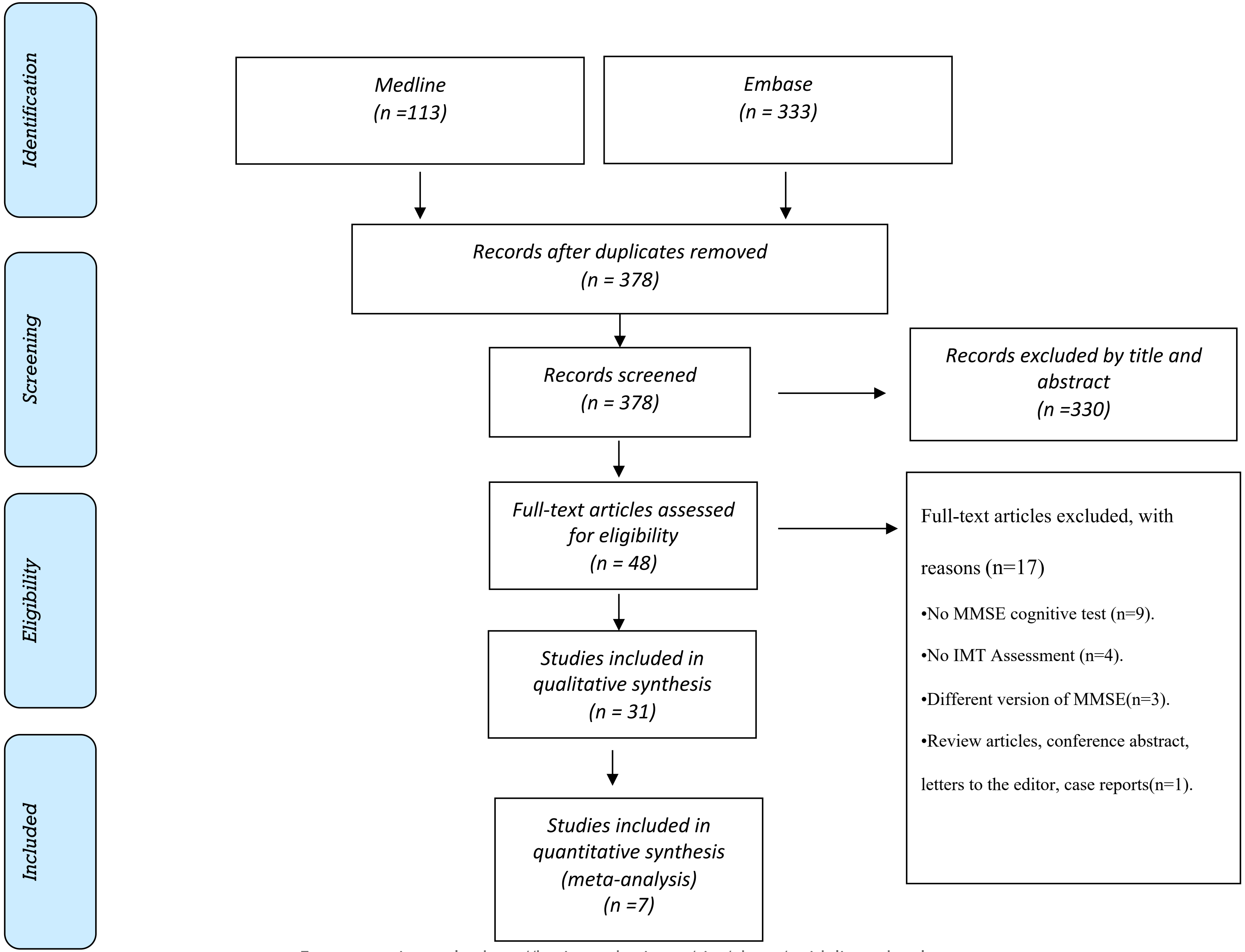
Identification
Screening
Eligibility
Included
Medline
(n =113)
Embase
(n = 333)
Records after duplicates removed
(n = 378)
Records screened
(n = 378)
Records excluded by title and abstract
(n =330)
Full-text articles assessed for eligibility
(n = 48)
Full-text articles excluded, with reasons (n=17)
•No MMSE cognitive test (n=9).
•No IMT Assessment (n=4).
•Different version of MMSE(n=3).
•Review articles, conference abstract, letters to the editor, case reports(n=1).
Studies included in qualitative synthesis
(n = 31)
Studies included in quantitative synthesis (meta-analysis)
(n =7)

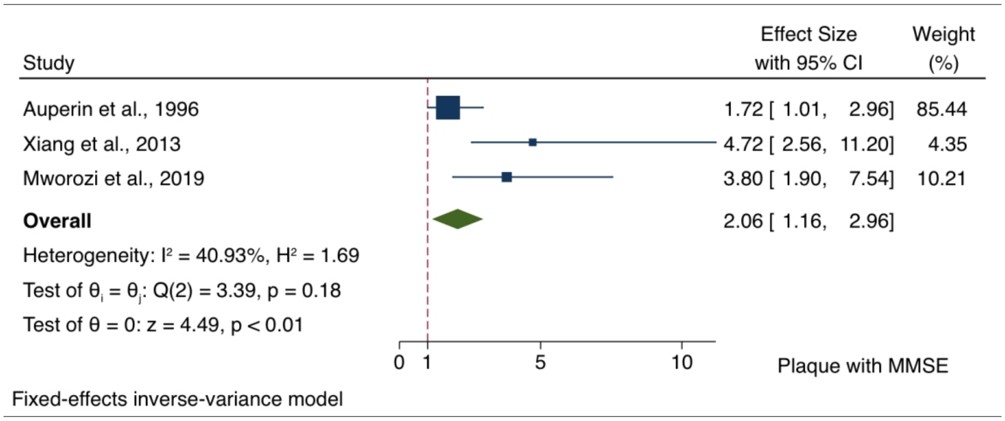

FIGURE 2. Forest plots of the association between carotid plaques and mini-mental state exam (MMSE).

564x233mm (72 x 72 DPI)

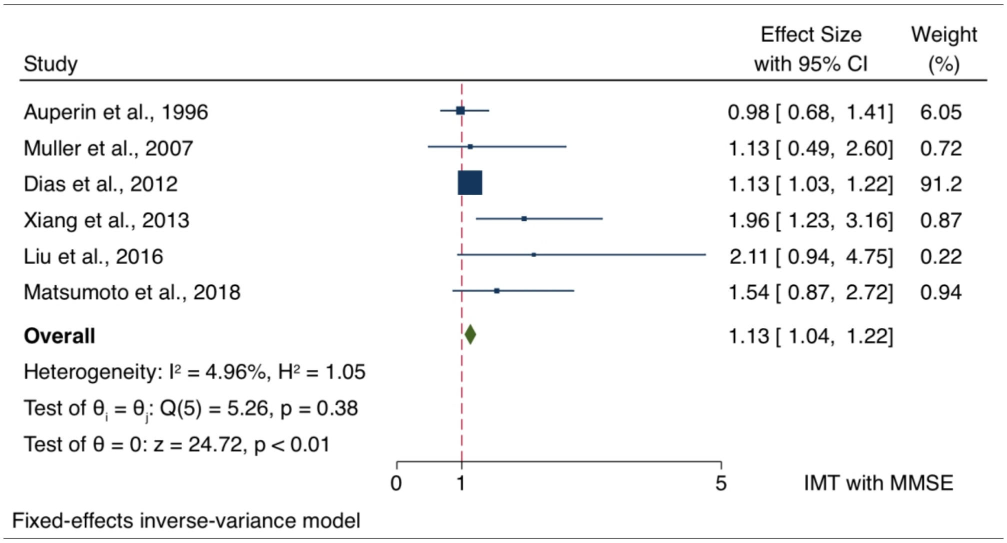

FIGURE 3. Forest plots of the association between carotid intima-media thickness (cIMT) and mini-mental state exam (MMSE).

564x301mm (72 x 72 DPI)

A, Carotid plaques **B, IMT**

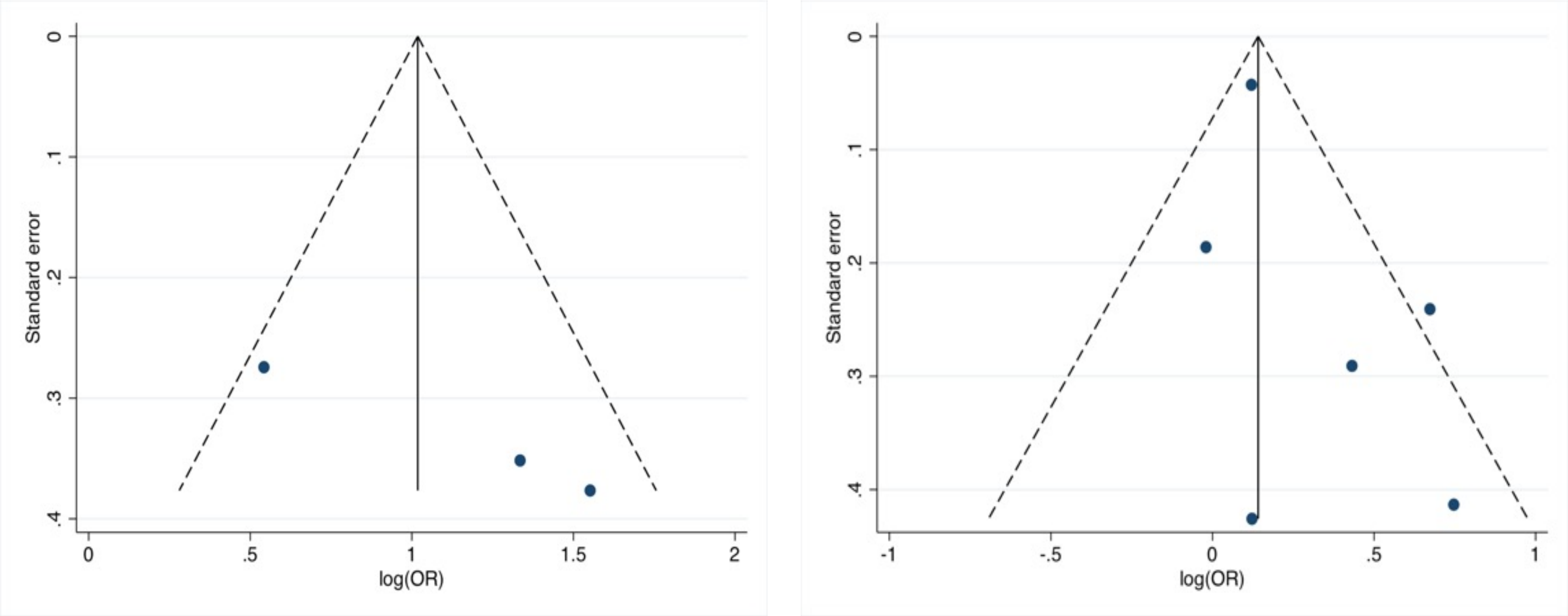

BMJ Open

Is Carotid Artery Atherosclerosis Associated with Poor Cognitive Function Assessed using the Mini-Mental State Examination? a systematic review and meta-analysis

Journal:	*BMJ Open*
Manuscript ID	bmjopen-2021-055131.R1
Article Type:	Original research
Date Submitted by the Author:	15-Mar-2022

Complete List of Authors:	Anbar, Rayan; King Abdulaziz University, Diagnostic Radiology, Faculty of Applied Medical Sciences; University College London, MRC Unit for Lifelong Health and Aging, UCL Institute of Cardiovascular Science, Faculty of Population Health Sciences<br>Sultan, Salahaden; King Abdulaziz University, Diagnostic Radiology, Faculty of Applied Medical Sciences<br>Al Saikhan, Lamia; Imam Abdulrahman Bin Faisal University, College of Applied Medial Sciences<br>Alkharaiji, Mohammed ; Saudi Electronic University, Department of Public Health<br>Chaturvedi, Nishi; University College London, MRC Unit for Lifelong Health and Aging, UCL Institute of Cardiovascular Science<br>Hardy, Rebecca; University College London, Social Research Institute, UCL Institute of Education<br>Richards, M; University College London, MRC Unit for Lifelong Health and Aging, UCL Institute of Cardiovascular Science<br>Hughes, A; University College London, MRC Unit for Lifelong Health and Aging, UCL Institute of Cardiovascular Science

**Primary Subject Heading**:	Mental health
Secondary Subject Heading:	Cardiovascular medicine, Diagnostics
Keywords:	Ultrasound < RADIOLOGY & IMAGING, Cardiovascular imaging < RADIOLOGY & IMAGING, Dementia < NEUROLOGY, VASCULAR MEDICINE

SCHOLARONE™
Manuscripts

I, the Submitting Author has the right to grant and does grant on behalf of all authors of the Work (as defined in the below author licence), an exclusive licence and/or a non-exclusive licence for contributions from authors who are: i) UK Crown employees; ii) where BMJ has agreed a CC-BY licence shall apply, and/or iii) in accordance with the terms applicable for US Federal Government officers or employees acting as part of their official duties; on a worldwide, perpetual, irrevocable, royalty-free basis to BMJ Publishing Group Ltd ("BMJ") its licensees and where the relevant Journal is co-owned by BMJ to the co-owners of the Journal, to publish the Work in this journal and any other BMJ products and to exploit all rights, as set out in our licence.

The Submitting Author accepts and understands that any supply made under these terms is made by BMJ to the Submitting Author unless you are acting as an employee on behalf of your employer or a postgraduate student of an affiliated institution which is paying any applicable article publishing charge ("APC") for Open Access articles. Where the Submitting Author wishes to make the Work available on an Open Access basis (and intends to pay the relevant APC), the terms of reuse of such Open Access shall be governed by a Creative Commons licence – details of these licences and which Creative Commons licence will apply to this Work are set out in our licence referred to above.

Other than as permitted in any relevant BMJ Author's Self Archiving Policies, I confirm this Work has not been accepted for publication elsewhere, is not being considered for publication elsewhere and does not duplicate material already published. I confirm all authors consent to publication of this Work and authorise the granting of this licence.

For peer review only

Is Carotid Artery Atherosclerosis Associated with Poor Cognitive Function Assessed Using the Mini-Mental State Examination? A Systematic Review and Meta-analysis

Rayan Anbar[1,2], Salahaden R Sultan[1], Lamia Al Saikhan [3], Mohammed Alkharaiji[4], Nish Chaturvedi[2], Rebecca Hardy[5], Marcus Richards[2], Alun D Hughes[2]

1. Department of Diagnostic Radiology, Faculty of Applied Medical Sciences, King Abdulaziz University, Jeddah, Saudi Arabia

2. MRC Unit for Lifelong Health and Aging, UCL Institute of Cardiovascular Science, Faculty of Population Health Sciences, University College London, London, UK

3. Department of Cardiac Technology, College of Applied Medial Sciences, Imam Abdulrahman Bin Faisal University, 2835 King Faisal Street, Dammam 34212, Kingdom of Saudi Arabia

4. Faculty of Public Health, College of Health Science, The Saudi Electronic University, Riyadh, Saudi Arabia

5. Social Research Institute, UCL Institute of Education, University College London, London, UK

Corresponding author:

Prof Alun Hughes, MRC Unit for Lifelong Health and Aging, UCL Institute of Cardiovascular Science, Faculty of Population Health Sciences, University College London, London, UK. Email: alun.hughes@ucl.ac.uk

For peer review only

ABSTRACT

Objectives: To determine associations between carotid atherosclerosis assessed by ultrasound and the mini-mental state examination (MMSE), a measure of global cognitive function.

Design: Systematic review and meta-analysis.

Methods: MEDLINE and EMBASE databases were searched up to 01/05/2020 to identify studies assessed the associations between asymptomatic carotid atherosclerosis and the MMSE. Studies reporting odds ratios (OR) for associations between carotid plaque or intima-media thickness (cIMT) and dichotomised MMSE were meta-analysed. Publication bias of included studies was assessed. This study is registered on the international prospective register of systematic reviews (PROSPERO) (registration number CRD42021240077).

Results: A total of 31 of 378 reviewed articles met the inclusion criteria; together they included 27,738 participants (age 35 to 95 years). Fifteen studies reported some evidence of a positive association between measures of atherosclerosis and poorer cognitive performance in either cross-sectional or longitudinal studies. The remaining 16 studies found no evidence of an association. Seven cross-sectional studies provided data suitable for meta-analysis. Meta-analysis of three studies that assessed carotid plaque (n= 3,549) showed an association between the presence of plaque and impaired MMSE with pooled estimate for the OR (95% CI) being 2.72 (0.85, 4.59). An association between cIMT and impaired MMSE was reported in six studies (n=4,443) with a pooled estimate for the OR (95% CI) being 1.13 (1.04, 1.22). Heterogeneity across studies was moderate to small (carotid plaque with MMSE, I^2=40.9%; cIMT with MMSE, I^2=4.9%). There was evidence of publication bias for carotid plaque studies (p=0.02), but not cIMT studies (p=0.2).

Conclusions: There is some, limited cross-sectional evidence indicating an association between cIMT and poorer global cognitive function assessed with MMSE. Estimates of the association between plaques and poor cognition are too imprecise to draw firm conclusions and evidence from studies of longitudinal associations between carotid atherosclerosis and MMSE is limited.

For peer review only

Funding:

RA is supported by a PhD scholarship grant from King Abdulaziz University.

NC received support from the National Institute for Health Research University College London Hospitals Biomedical Research Centre and works in a unit that receives support from the UK Medical Research Council.

AH receives support from the British Heart Foundation, the Economic and Social Research Council (ESRC), the Horizon 2020 Framework Programme of the European Union, the National Institute on Aging, the National Institute for Health Research University College London Hospitals Biomedical Research Centre, the UK Medical Research Council, the Wellcome Trust, and works in a unit that receives support from the UK Medical Research Council.

MR is supported by UK Medical Research Council grants MC_UU_12019/1 (Enhancing NSHD) and /3 (Mental Ageing).

Keywords: atherosclerosis, cognitive function, carotid artery, ultrasound, systematic review, Meta-analysis, Mini Mental State Examination, intima-media thickness.

For peer review only

Strengths and limitations of this study

- We performed a systematic review on 31 published studies assessed the association between carotid atherosclerosis and global cognition.
- The association between carotid atherosclerosis and global cognition was meta-analysed on 7,992 asymptomatic patients.
- There was some evidence from cross-sectional studies that carotid plaque and cIMT were associated with impaired MMSE.
- Methods used and characteristics of participants across studies were different which makes it difficult to compare all studies.
- This review only included studies which used MMSE as a cognitive function test which may result in bias or an underestimation of decline in cognitive state.

Competing interests statements

Rayan Anbar – none

Salahaden R Sultan – none

Lamia Al Saikhan – none

Mohammed Alkharaiji – none

Nish Chaturvedi – NC has received personal fees from Astra Zeneca outside of the submitted work.

Rebecca Hardy – none

Marcus Richards – none

Alun Hughes – none

For peer review only

INTRODUCTION

Improvements in life expectancy have led to a dramatic increase in the worldwide burden of age-related diseases, notably atherosclerotic cardiovascular disease, and cognitive decline and dementia[1].

Atherosclerosis is a chronic inflammatory disease, which begins in childhood[2]. With time and progression of disease, fibro-fatty plaques develop which may result in alterations in the elastic properties of the artery, narrowing of the lumen and resultant effects on haemodynamic, or thromboembolic consequences. Atherosclerosis affects most large arteries, including the common carotid artery (CCA) and its major branch, the internal carotid artery[3]. Carotid artery atherosclerosis is common[4] and may be an important cause of cerebral ischaemia and cognitive impairment. In carotid stenosis, embolization and hypoperfusion are thought to be the major processes that cause cognitive impairment,[5] but alterations in cerebral haemodynamics secondary to atherosclerosis could also make a contribution that is potentially reversible.[6 7]The assessment of carotid disease can be performed using a range of imaging methods, (e.g. magnetic resonance imaging (MRI), computed tomography (CT), angiography, and ultrasound [8]), ultrasound is the most commonly used [9-12], as it is inexpensive, non-invasive and widely available.

Cognitive function refers to internal mental processes which help people to think, make decisions and solve problems[13], and a decline in cognitive function can affect patients' health, daily routine, learning new things, speech and writing abilities, and their ability to live independently. A decline in cognitive function is common with advancing age,[14] and severe cognitive impairment and dementia are placing an increasingly heavy social, emotional and economic burden on society.[15] Dementia is a syndrome that results from diseases of the brain which are usually chronic or progressive.[15] Dementia is characterised by severe deficits in cognition that have a substantial effect on the activities of daily life. These impairments in cognition are also often accompanied by changes in emotional control, social behaviour and motivation. [15] Mild cognitive impairment (MCI) is defined as an impairment of cognition that is greater than expected based on an individual's age and education which does not appreciably affect daily functioning. Nevertheless, despite its denomination as mild, it is well established that people with MCI are at a high risk of developing dementia, particularly of the Alzheimer type[16]A comprehensive assessment of neuropsychological cognitive performance at the domain level is time consuming and most studies use brief cognitive screening tools[17].

For peer review only - http://bmjopen.bmj.com/site/about/guidelines.xhtml

The most widely-used tool for cognitive testing is the MMSE[18-20]. MMSE contains 11 tasks and covers 7 domains: visuospatial skills, language, concentration, working memory, memory recall, and orientation and is scored out of a maximum 30 points.[21] The commonest cut-points to detect dementia with the MMSE are ≤23 or ≤24, although higher and lower cut-points have been used in some studies[19 20].

Several studies have used a combination of carotid ultrasound and MMSE to examine the associations between carotid atherosclerosis and cognitive performance, either cross-sectionally or longitudinally; however, these studies have yielded conflicting results. For example, the Rotterdam Study [22] reported that common carotid artery intima-media thickness (cIMT) was associated with increased risk of Alzheimer's disease, but not vascular dementia, whereas carotid plaques were not associated with either Alzheimer's disease or vascular dementia. In contrast, the Framingham Offspring Cohort study [23] reported that cIMT was not associated with any measure of cognition, although there was evidence of an association between cIMT and impaired verbal memory and nonverbal memory, and that carotid stenosis ≥50% was associated with impaired executive function, but not verbal and non-verbal memory. The aim of this study therefore was to systematically review evidence of an association between carotid atherosclerosis assessed by ultrasound and global cognitive function assessed using the MMSE, also to perform a meta-analysis of quantitative measures of association between carotid atherosclerosis and global cognitive function.

METHODS

This study was conducted according to the 'Preferred Reporting Items for Systematic Reviews and Meta-Analyses'(PRISMA) statement[24] and registered in the international prospective register of systematic reviews (PROSPERO) (registration number CRD42021240077). Patients or the public were not involved in the design, or conduct, or reporting, or dissemination plans of our research.

Search strategy

The search was conducted systematically (up to 01/05/2020) by two authors independently in two online databases: MEDLINE and EMBASE Classic + EMBASE following training and support from a information specialist. A combination of synonyms and related words and text word searching was used to comprehensively extract all relevant articles. A combination of indexed (MesH) terms and keyword searches in titles and abstracts was used - (1) cognition,

For peer review only - http://bmjopen.bmj.com/site/about/guidelines.xhtml

cognitive function, dementia, Alzheimer disease; (2) atherosclerosis, intima media thickness, plaque; (3) carotid arteries;(4) All searches were limited to (English language and humans). The results from databases were exported to an Endnote library, and any duplicates results were identified and removed before screening the records. The records of non-relevant articles were excluded by screening titles and abstracts by three authors working independently. The remaining publications were assessed by screening the full texts for eligibility and were retrieved and double screened. Discrepancies were reviewed and resolved through consensus. full search strategies for all databases available **Supplementary files 1&2**.

Inclusion criteria

Relevant studies were required to include the following: (1) Human adults (i.e. a person older than 19 years of age)[25] (2) Carotid intima media thickness and/or carotid artery plaque measured using ultrasound and (3) Mini-mental state examination (MMSE) as a global cognitive function test.

Exclusion criteria

The exclusion criteria as follows: (1) Adults following stroke (if results in stroke patients were reported separately these were excluded, or if the study only included participants with stroke the entire study was excluded); (2) Patients who had undergone carotid surgical and non-surgical intervention; (3) Studies that used medical imaging modalities other than ultrasound to assess the presence of plaque and to measure carotid atherosclerosis; (4) Review articles, conference abstracts, case reports, letters to the editor, or commentary articles.

Data extraction

Data extraction from the included studies were performed by three researchers independently using standardized forms; any discrepancies were resolved by consensus. The extracted data from the eligible studies consisted of study characteristics such as author, year of publication, sample size, characteristics of participants (age, and gender), study region, MMSE cognitive function test score, and cIMT measurements (Tables 1,A,B,C). Means and standard deviations and sample size of cIMT and MMSE were extracted from the included papers. If mean and standard deviation were not provided, median and range or other measures of central tendency and dispersion were extracted. Maximum cIMT was extracted if the mean value was not reported in the studies. If necessary, odds ratios for cIMT were converted to be per 1mm IMT. If the percentage of people with plaques was reported, the total number was calculated. For

For peer review only - http://bmjopen.bmj.com/site/about/guidelines.xhtml

studies in which stroke sufferers were included in the sample size, the total number of subjects were re-calculated by removing subjects with stroke. The sample size was extracted of which cIMT and MMSE were measured. If different numbers of subject underwent cIMT and MMSE assessments, the lower number was recorded. The percentage of female subjects was extracted. If the total number of females was provided, the percentage of females from the overall sample size was calculated. The data was collected from baseline values or nearest value to the baseline if baseline was not available. If there were data from the article that was deficient, the authors were contacted by email. The form for eligibility papers is available in **Supplementary files 3**.

Patient and Public Involvement

This study does not involve human participants and did not require ethical review.

Meta-analysis

A meta-analysis was planned using odds ratios (OR) with 95% confidence intervals (CI) as the estimates of the strength of association between carotid atherosclerosis and MMSE. If other measures of dispersion were reported these were converted into 95% CI. Odds ratios were chosen as recommended by Borenstein et al [26].As this was the effect size measure most commonly presented in the identified studies and also has the advantage of symmetry for outcome definition[27]. The two measures of carotid atherosclerosis used for meta-analysis were presence of carotid plaque and cIMT as these were the most common exposures examined in identified studies. The outcome, MMSE was categorised into normal or impaired based on a cut-point of 24, since this was commonest threshold used for analysis in the papers identified. To facilitate comparison of effect sizes from longitudinal studies with those reported by cross-sectional studies hazard ratios or risk ratios were converted to OR using the equation described by Grant.[28] Random effects meta-analysis was performed using Stata/SE Statistical Software version 16.1 (StataCorp., College Station, TX) using the meta suite of commands; weighting was by the inverse variance method. Q-test and I^2 statistics were used to assessed heterogeneity between studies. We used the Egger's test and funnel plots to look for the possibility of publication bias.

Quality Assessment

The quality of included studies was scored using a modified seven-point criteria were derived from Newcastle-Ottawa scale.[29] Scores covered the 1) representativeness of the target sample,

For peer review only - http://bmjopen.bmj.com/site/about/guidelines.xhtml

2) non-response satisfactorily dealt with, 3) use of a validated measurement tool, 4) relevant confounders measured, 5) appropriate assessment of outcome, 6) appropriate statistical analysis clearly described and 7) reporting missing data if present. Each criterion was assigned one point if met. The quality assessment was performed by two researchers independently using standardized forms and any discrepancies were resolved by consensus. The Newcastle-Ottawa scale is provided in **Supplementary files 4**.

RESULTS

The PRISMA flowchart showing the study selection process is shown in Fig 1. We identified 378 potential studies from the electronic databases (Medline & Embase) after exclusion of duplicates. Title and abstract screening resulted in 48 records remaining to be reviewed for eligibility in full text. Of those studies, 17 were excluded with reasons. The reasons were: no cIMT measurement (n = 4), or no MMSE test data (n = 9) or use of a modified version of MMSE (n = 3), review articles, conference abstract, letters to the editor or case reports (n = 1).

Characteristics studies included in the systematic review

The total number of participants in the 31 included studies was 26,178, with an age range from 35 to 95 years. Twenty of the studies were cross-sectional **(Supplementary Table S1A**); eight were longitudinal **(Supplementary Table S1B**). The remaining 3 studies included a mixture of cross-sectional and longitudinal data **(Supplementary Table S1C**). The geographical distribution of the studies was diverse: with 5 from Italy, 5 from Japan, 5 from USA, 3 from France, 2 from Brazil, 2 from China, 3 from UK, and 1 from each of Egypt, Netherlands, Norway, Serbia, Sweden, and Uganda. One study failed to mention the geographical location. Only one study [30] failed to report the patients' health conditions and risk factors, such as diabetes mellitus, hypertension, and chronic kidney disease. Two studies included only male participants [31 32], and one study included only female participants [33], the remaining studies included both male and female participants. Details of the key exposure measures are shown in **Supplementary Table S2**. Twenty-nine studies reported the mean of carotid intima media thickness measurement[30-58]. Fourteen studies did not mention the presence of carotid plaques[31 33-36 41 45 48 49 51 52 54-56]. Seven studies did not provide MMSE scores[44 45 50 52 55 58 59].

Associations between atherosclerosis measures and MMSE score

Cross-sectional associations

For peer review only - http://bmjopen.bmj.com/site/about/guidelines.xhtml

Presence of carotid plaques was associated with poorer cognitive function in 3 studies [40 58 59]. Auperin et al. [30] reported an association between higher prevalence of carotid plaque and lower MMSE in men, but not in women. Another study [35] concluded that the association between cIMT and cognitive function was observed only in a low socioeconomic status group. Another study [42] found a significant correlation between higher cIMT and plaques with poor MMSE score in people with evidence of vascular cognitive decline, but not in a group of people with Alzheimer's disease and Watanabe et al. [32] reported that vascular dementia patients were more likely to have low MMSE score with thicker cIMT and frequent presences of carotid plaques. Ten cross-sectional studies [34 36 37 41 43 46 48 51 56 60] reported that cIMT was not associated with changes in MMSE, although some of these studies reported associations with a selected aspect of cognitive performance.

Longitudinal associations

Zhong et al. reported an association between higher cIMT and a reduction in MMSE score after 10 years of follow-up but not after 5-years; a similar association between presence of carotid plaque and change in MMSE was not observed at any follow-up interval[44]. Rouch et al. found that cIMT and presence of carotid plaques were associated with progression to dementia in univariable analyses, but neither predicted progression after multivariable adjustment [57]. Another study found that the plaque index, and cIMT were predictive of an increased risk of conversion of mild cognitive impairment to dementia including after multivariable adjustment using a backwards stepwise method[61]. While Carcaillon et al. reported that there was an association between presence of carotid plaque in two sites or more and incidence of dementia, they observed no association between cIMT measured at plaque-free sites with incident dementia[50]. Silvestrini et al. found that baseline cIMT and increase in cIMT adjusted for baseline were associated with decline in MMSE[39]. Wendell et al.found no association between baseline cIMT and change in MMSE, but observed that higher cIMT was associated with future changes in selected aspects of cognitive performance[52]. One study did not provide quantitative data about possible associations between carotid plaque or cIMT and MMSE [54], while 4 other studies found no evidence that cIMT was associated with future global cognitive impairment [33 45 47 55].

Quality

One study received quality scores of 6 out of 7 [43]; five received a quality score of 5 out of 7 [32 47 50 57 59]; twenty a quality score of 4 out of 7 [31 33-42 44-46 48 51-54 61]; four a quality score of 3 out

For peer review only - http://bmjopen.bmj.com/site/about/guidelines.xhtml

of 7 [30 49 56 58]; and one a quality score of 2 out of 7 [55]. Among the 31 included studies, 24 studies were adequately designed to represent the target sample. 9 publications used validated ultrasound measurement tools, 6 reported blinding assessment of outcome and blinding of outcome measurements, 29 described the statistical analysis satisfactorily. Further details are provided in **Supplementary Table S3.**

Meta-analysis

Seven of the 31 studies reported data that were suitable for inclusion in a meta-analysis [30 31 41 44 46 55 58]. All included studies were cross-sectional, and all but one drew from population samples. Of all studies, 3 reported associations between carotid plaque and impaired MMSE [30 46 62], and 6 that reported the association between cIMT and impaired MMSE [30 31 41 46 55 61]. There was only one suitable longitudinal study[44], so a meta-analysis was not attempted for this category of study.

Carotid plaque and MMSE meta-analysis

Meta-analysis showed an association between presence of carotid plaque and impaired MMSE. The overall OR for the association between carotid plaque and impaired MMSE was 2.06 (95% CI: 1.16, 2.96) for presence of plaque, and with Z-score = 4.49, P < 0.01 (Figure 2).

Association between cIMT and MMSE

Meta-analysis showed an association between cIMT and impaired MMSE. The overall OR for the association between cIMT and impaired MMSE was 1.13 (95% CI: 1.04, 1.22) per 1mm cIMT, with Z-score = 24.7, P < 0.01 (Figure 3).

Publication Bias

Egger`s test showed evidence of bias in studies assessing between carotid plaque and MMSE (p=0.02), but not in cIMT with MMSE (p=0.2) (Figure 4 A&B).

DISCUSSION

This systematic review examined and quantitated the evidence regarding a possible association between measures of atherosclerosis in the carotid arteries by ultrasound and generalised impairment of cognitive function assessed using the MMSE. From the limited studies available,

For peer review only - http://bmjopen.bmj.com/site/about/guidelines.xhtml

our analysis showed there is some evidence of a cross-sectional association between carotid artery atherosclerosis and poorer MMSE, with the majority of studies finding an association. However, a sizeable proportion of studies did not find evidence for an association between cIMT and MMSE score. These discrepancies could be due to inadequate sample size, sample selection, or the low sensitivity of MMSE for detection of mild cognitive impairment.

Risk factors for CVD are related with changes in cognitive funcation[63-66] and many factors could link carotid atherosclerosis with cognition. These include thromboembolic consequences of carotid disease, small vessel disease and cerebral perfusion abnormalities, shared genetic predisposition, or shared risk factors over the life course. A recent study that observed a relationship between cIMT and MMSE, memory and executive function, also found an association between cIMT and decrease in brain matter volumes and cerebral hypoperfusion[67].

Few studies provided information regarding factors, such as age, gender, socioeconomic position (SEP), or pre-existing CVD, that might modify any relationship between carotid atherosclerosis and cognitive impairment, so it was not possible to formally examine effect modification by meta-analysis. One study reported that there was a moderate association between presence of plaques and impaired MMSE in men, but no association in women[30]. In contrast, many studies failed to report evidence of sex differences in the association between carotid atherosclerosis and cognitive impairment,[68-70] although it was often not clear whether such an interaction was sought. Limited evidence suggests that SEP, which may include income, education, and social supports may modify the association between atherosclerosis and cognitive performance. In Whitehall study of UK-based civil servants, [35] an association between higher cIMT and poorer cognitive function was only observed in individuals classified as low SEP. Another study done in USA, [52] reported that the association between cIMT and measures of cognition differed as a function of race and socioeconomic status.

Limitations

Our systematic review has several limitations. Restricting the search to only literature published in the English language only may have reduced the number of citations identified. The search was performed to cover publications up to 01/05/2020 and information published after that date will not have been included. The studies included in the review were drawn from

For peer review only - http://bmjopen.bmj.com/site/about/guidelines.xhtml

diverse geographical locations, but while there was no obvious difference by location it was not possible to formally examine this question. Relatedly most studies did not report on the ethnicity of the participants, and it was therefore not possible to conclude anything about potential ethnic differences in associations. Similarly the lack of availability of individual participant data and the limitation of the summary data available restricted the analyses that could be performed. The primary aim of some the studies reviewed was not to assess the association between atherosclerosis in carotid arteries and cognitive function. Adjustment for potential confounders was quite variable and inconsistent across studies and it is likely that this will contribute to heterogeneity between studies. There was also substantial heterogeneity with regards to atherosclerosis measurements; for example, some studies provided more information on plaque burden (e.g. number, thickness or location of plaques) but this analysis was too infrequent and inconsistent across studies to allow synthesis of these data and consequently presence of plaque was chosen as the exposure of interest. There were also difference in scanning techniques between studies, and most of studies reviewed did not perform a comprehensive carotid scan. The use of B-mode ultrasound to detect carotid atherosclerosis has well-recognised limitations[71 72] and more advanced ultrasound imaging including 3D ultrasound[73], or other imaging techniques, such as MRI[74] or PET[75], that better quantify and characterise vulnerable atherosclerosis may have advantages. Similarly, use of the MMSE has several limitations, including non-linearity, a floor effect in advanced dementia, a ceiling effect in very mild disease, and bias in people with little formal education or in non-English speaking groups[18-20]. Also, MMSE only provides only a limited insight into the complex process of cognitive decline and use of a wider range of tools, for example assessment of a wider range of cognitive domains including executive functioning and psychomotor speed, would be worthwhile in future studies.

CONCLUSION

This meta-analysis and systematic review provide some evidence in favour of an association between cIMT and cognitive function in cross-sectional studies. Estimates of the association between plaques and poor cognition in cross-sectional studies were too imprecise to draw firm conclusions; while evidence from studies of longitudinal associations between carotid atherosclerosis and cognitive function is limited.

For peer review only - http://bmjopen.bmj.com/site/about/guidelines.xhtml

Contributors: RA, AH, RH and MR developed the idea and designed the study protocol. RA, LA, and SS designed and wrote the search strategy and data extraction. RA, MA, AH, RH and SS planned and performed the statistical analysis and results interpretation. NC, RA, RH, MR, and AH provided critical insights. All authors have approved and contributed to the final written manuscript.

Funding:

RA is supported by a PhD scholarship grant from King Abdulaziz University.

NC received support from the National Institute for Health Research University College London Hospitals Biomedical Research Centre and works in a unit that receives support from the UK Medical Research Council.

AH receives support from the British Heart Foundation, the Economic and Social Research Council (ESRC), the Horizon 2020 Framework Programme of the European Union, the National Institute on Aging, the National Institute for Health Research University College London Hospitals Biomedical Research Centre, the UK Medical Research Council, the Wellcome Trust, and works in a unit that receives support from the UK Medical Research Council.

MR is supported by UK Medical Research Council grants MC_UU_12019/1 (Enhancing NSHD) and /3 (Mental Ageing).

Ethical approval statement: This study is for conducting a systematic review and meta-analysis hence no ethical approval was required.

Competing interests: None declared.

Patient consent for publication: Not required.

Provenance and peer review: Not commissioned, externally peer reviewed.

Data availability: Data are available upon reasonable request.

For peer review only - http://bmjopen.bmj.com/site/about/guidelines.xhtml

REFERENCES

1. Naghavi M, Abajobir AA, Abbafati C, et al. Global, regional, and national age-sex specific mortality for 264 causes of death, 1980-2016: a systematic analysis for the Global Burden of Disease Study 2016. Lancet 2017;390(10100):1151-210. doi: Doi 10.1016/S0140-6736(17)32152-9
2. McGill HC, Jr., McMahan CA, Gidding SS. Preventing heart disease in the 21st century: implications of the Pathobiological Determinants of Atherosclerosis in Youth (PDAY) study. Circulation 2008;117(9):1216-27. doi: 10.1161/CIRCULATIONAHA.107.717033
3. Sobieszczyk P, Beckman J. Carotid Artery Disease. Circulation 2006;114(7):e244-e47. doi: 10.1161/circulationaha.105.542860
4. Song P, Fang Z, Wang H, et al. Global and regional prevalence, burden, and risk factors for carotid atherosclerosis: a systematic review, meta-analysis, and modelling study. Lancet Global Health 2020;8(5):e721-e29. doi: 10.1016/s2214-109x(20)30117-0
5. Dutra AP. Cognitive function and carotid stenosis: Review of the literature. Dement Neuropsychol 2012;6(3):127-30. doi: 10.1590/S1980-57642012DN06030003
6. Marshall RS. Effects of altered cerebral hemodynamics on cognitive function. J ALZHEIMERS DIS 2012;32(3):633-42. doi: 10.3233/JAD-2012-120949
7. Silvestrini M, Paolino I, Vernieri F, et al. Cerebral hemodynamics and cognitive performance in patients with asymptomatic carotid stenosis. NEUROLOGY 2009;72(12):1062-68. doi: 10.1212/01.wnl.0000345015.35520.52
8. Davis PH, Dawson JD, Riley WA, et al. Carotid intimal-medial thickness is related to cardiovascular risk factors measured from childhood through middle age: The Muscatine Study. Circulation 2001;104(23):2815-9. doi: 10.1161/hc4601.099486 [published Online First: 2001/12/06]
9. Breteler MMB, Claus JJ, Grobbee DE, et al. Cardiovascular disease and distribution of cognitive function in elderly people: The Rotterdam study. British Medical Journal 1994;308(6944):1604-08.
10. Stein JH, Korcarz CE, Hurst RT, et al. Use of Carotid Ultrasound to Identify Subclinical Vascular Disease and Evaluate Cardiovascular Disease Risk: A Consensus Statement from the American Society of Echocardiography Carotid Intima-Media Thickness Task Force Endorsed by the Society for Vascular Medicine. Journal of the American Society of Echocardiography 2008;21(2):93-111. doi: 10.1016/j.echo.2007.11.011
11. O'Leary DH, Bots ML. Imaging of atherosclerosis: carotid intima–media thickness. European Heart Journal 2010;31(14):1682-89. doi: 10.1093/eurheartj/ehq185
12. Touboul PJ, Hennerici MG, Meairs S, et al. Mannheim carotid intima-media thickness and plaque consensus (2004-2006-2011). An update on behalf of the advisory board of the 3rd, 4th and 5th watching the risk symposia, at the 13th, 15th and 20th European Stroke Conferences, Mannheim, Germany, 2004, Brussels, Belgium, 2006, and Hamburg, Germany, 2011. Cerebrovasc Dis 2012;34(4):290-96. doi: 10.1159/000343145 [published Online First: 2012/11/01]
13. Roy E. Cognitive Function. In: Gellman MD, Turner JR, eds. Encyclopedia of Behavioral Medicine. New York, NY: Springer New York 2013:448-49.
14. Chaytor N, Schmitter-Edgecombe M. The ecological validity of neuropsychological tests: a review of the literature on everyday cognitive skills. Neuropsychol Rev 2003;13(4):181-97. doi: 10.1023/b:nerv.0000009483.91468.fb [published Online First: 2004/03/06]
15. Dening T, Sandilyan MB. Dementia: definitions and types. Nursing Standard (2014+) 2015;29(37):37.

For peer review only - http://bmjopen.bmj.com/site/about/guidelines.xhtml

16. Gauthier S, Reisberg B, Zaudig M, et al. Mild cognitive impairment. The Lancet 2006;367(9518):1262-70. doi: https://doi.org/10.1016/S0140-6736(06)68542-5
17. Faber RA. The Neuropsychiatric Mental Status Examination. SEMIN NEUROL 2009;29(3):185-93. doi: 10.1055/s-0029-1223874
18. Tsoi KKF, Chan JYC, Hirai HW, et al. Cognitive Tests to Detect Dementia: A Systematic Review and Meta-analysis. JAMA Internal Medicine 2015;175(9):1450-58. doi: 10.1001/jamainternmed.2015.2152
19. Mitchell AJ. A meta-analysis of the accuracy of the mini-mental state examination in the detection of dementia and mild cognitive impairment. Journal of Psychiatric Research 2009;43(4):411-31. doi: https://doi.org/10.1016/j.jpsychires.2008.04.014
20. Patnode CD, Perdue LA, Rossom RC, et al. Screening for Cognitive Impairment in Older Adults: An Evidence Update for the U.S. Preventive Services Task Force. Rockville (MD): Agency for Healthcare Research and Quality 2020.
21. Meyers CA, Wefel JS. The use of the mini-mental state examination to assess cognitive functioning in cancer trials: no ifs, ands, buts, or sensitivity. J Clin Oncol 2003;21(19):3557-8. doi: 10.1200/JCO.2003.07.080 [published Online First: 2003/08/13]
22. van Oijen M, Jan de Jong F, Witteman JC, et al. Atherosclerosis and risk for dementia. Annals of Neurology: Official Journal of the American Neurological Association and the Child Neurology Society 2007;61(5):403-10.
23. Romero JR, Beiser A, Seshadri S, et al. Carotid artery atherosclerosis, MRI indices of brain ischemia, aging, and cognitive impairment: the Framingham study. Stroke 2009;40(5):1590-6. doi: 10.1161/STROKEAHA.108.535245 [published Online First: 2009/03/07]
24. Moher D, Liberati A, Tetzlaff J, et al. Preferred reporting items for systematic reviews and meta-analyses: the PRISMA statement. PLoS Med 2009;6(7):e1000097.
25. Canêo LF, Neirotti R. The Importance of the Proper Definition of Adulthood: What is and What is Not Included in a Scientific Publication. Braz J Cardiovasc Surg 2017;32(1):60-60. doi: 10.21470/1678-9741-2016-0049
26. Borenstein M, Hedges LV, Higgins JP, et al. Introduction to meta-analysis: John Wiley & Sons 2021.
27. Cummings P. The relative merits of risk ratios and odds ratios. Archives of pediatrics & adolescent medicine 2009;163(5):438-45.
28. Grant RL. Converting an odds ratio to a range of plausible relative risks for better communication of research findings. Br Med J 2014;348(jan24 1):f7450-f50. doi: 10.1136/bmj.f7450
29. Wells G, Shea B, O'Connell D, et al. The Newcastle–Ottawa Scale (NOS) for Assessing the Quality of Non-Randomized Studies in Meta-Analysis. , 2000.
30. Auperin A, Berr C, Bonithon-Kopp C, et al. Ultrasonographic assessment of carotid wall characteristics and cognitive functions in a community sample of 59- to 71-year-olds. The EVA Study Group. Stroke 1996;27(8):1290-5. doi: 10.1161/01.str.27.8.1290 [published Online First: 1996/08/01]
31. Muller M, Grobbee DE, Aleman A, et al. Cardiovascular disease and cognitive performance in middle-aged and elderly men. Atherosclerosis 2007;190(1):143-49. doi: 10.1016/j.atherosclerosis.2006.01.005
32. Watanabe T, Koba S, Kawamura M, et al. Small dense low-density lipoprotein and carotid atherosclerosis in relation to vascular dementia. Metabolism, clinical and experimental 2004;53(4):476-82. doi: 10.1016/j.metabol.2003.11.020

For peer review only - http://bmjopen.bmj.com/site/about/guidelines.xhtml

33. Komulainen P, Kivipelto M, Lakka TA, et al. Carotid intima-media thickness and cognitive function in elderly women: A population-based study. Neuroepidemiology 2007;28(4):207-13.
34. Haley AP, Forman DE, Poppas A, et al. Carotid artery intima-media thickness and cognition in cardiovascular disease. International Journal of Cardiology 2007;121(2):148-54.
35. Singh-Manoux A, Britton A, Kivimaki M, et al. Socioeconomic status moderates the association between carotid intima-media thickness and cognition in midlife: Evidence from the Whitehall II study. Atherosclerosis 2008;197(2):541-48.
36. Carlsson CM, Nondahl DM, Klein BE, et al. Increased atherogenic lipoproteins are associated with cognitive impairment: effects of statins and subclinical atherosclerosis. Alzheimer Dis Assoc Disord 2009;23(1):11-7. doi: 10.1097/wad.0b013e3181850188 [published Online First: 2009/03/10]
37. El-Kattan MM, Zakaria YA, El-Fayomy N, et al. Peripheral arterial disease and cognition. Egyptian Journal of Neurology, Psychiatry and Neurosurgery 2009;46(2):311-22.
38. Kearney-Schwartz A, Rossignol P, Bracard S, et al. Vascular Structure and Function Is Correlated to Cognitive Performance and White Matter Hyperintensities in Older Hypertensive Patients With Subjective Memory Complaints. Stroke 2009;40(4):1229-36.
39. Silvestrini M, Gobbi B, Pasqualetti P, et al. Carotid atherosclerosis and cognitive decline in patients with Alzheimer's disease. Neurobiology of Aging 2009;30(8):1177-83.
40. Zhong W, Cruickshanks KJ, Huang GH, et al. Carotid atherosclerosis and cognitive function in midlife: the Beaver Dam Offspring Study. Atherosclerosis 2011;219(1):330-3. doi: 10.1016/j.atherosclerosis.2011.07.013 [published Online First: 2011/08/13]
41. Dias EDM, Giollo LT, Martinelli DD, et al. Carotid intima-media thickness is associated with cognitive deficiency in hypertensive patients with elevated central systolic blood pressure. Cardiovascular Ultrasound 2012;10(1)
42. Stefanova E, Pavlovic A, Jovanovic Z, et al. Vascular risk factors in Alzheimer's disease - preliminary report. J Neurol Sci 2012;322(1-2):166-9. doi: 10.1016/j.jns.2012.07.065 [published Online First: 2012/09/04]
43. Viticchi G, Falsetti L, Vernieri F, et al. Vascular predictors of cognitive decline in patients with mild cognitive impairment. Neurobiology of Aging 2012;33(6):1127e1-27e9.
44. Zhong W, Cruickshanks KJ, Schubert CR, et al. Carotid atherosclerosis and 10-year changes in cognitive function. Atherosclerosis 2012;224(2):506-10. doi: 10.1016/j.atherosclerosis.2012.07.024 [published Online First: 2012/08/03]
45. Rogne SO, Solbu MD, Arntzen KA, et al. Albuminuria and carotid atherosclerosis as predictors of cognitive function in a general population. European Neurology 2013;70(5-6):340-8.
46. Xiang J, Zhang T, Yang QW, et al. Carotid artery atherosclerosis is correlated with cognitive impairment in an elderly urban Chinese non-stroke population. Journal of Clinical Neuroscience 2013;20(11):1571-75.
47. Buratti L, Balucani C, Viticchi G, et al. Cognitive deterioration in bilateral asymptomatic severe carotid stenosis. Stroke 2014;45(7):2072-77.
48. Nagai M, Hoshide S, Nishikawa M, et al. Visit-to-visit blood pressure variability in the elderly: associations with cognitive impairment and carotid artery remodeling. Atherosclerosis 2014;233(1):19-26. doi: 10.1016/j.atherosclerosis.2013.11.071 [published Online First: 2014/02/18]

For peer review only - http://bmjopen.bmj.com/site/about/guidelines.xhtml

49. Yano Y, Bakris GL, Inokuchi T, et al. Association of cognitive dysfunction with cardiovascular disease events in elderly hypertensive patients. J Hypertens 2014;32(2):423-31. doi: 10.1097/HJH.0000000000000025 [published Online First: 2013/12/20]
50. Carcaillon L, Plichart M, Zureik M, et al. Carotid plaque as a predictor of dementia in older adults: the Three-City Study. Alzheimer's & Dementia 2015;11(3):239-48.
51. Liu Z, Zhao Y, Wang X, et al. Low carotid artery wall shear stress is independently associated with brain white-matter hyperintensities and cognitive impairment in older patients. Atherosclerosis 2016;247:78-86. doi: 10.1016/j.atherosclerosis.2016.02.003 [published Online First: 2016/02/13]
52. Wendell CR, Waldstein SR, Evans MK, et al. Subclinical carotid atherosclerosis and neurocognitive function in an urban population. Atherosclerosis 2016;249:125-31. doi: 10.1016/j.atherosclerosis.2016.04.009 [published Online First: 2016/04/20]
53. Alhusaini S, Karama S, Nguyen TV, et al. Association between carotid atheroma and cerebral cortex structure at age 73 years. Ann Neurol 2018;84(4):576-87. doi: 10.1002/ana.25324 [published Online First: 2018/09/05]
54. Falsetti L, Viticchi G, Buratti L, et al. Interactions between Atrial Fibrillation, Cardiovascular Risk Factors, and ApoE Genotype in Promoting Cognitive Decline in Patients with Alzheimer's Disease: A Prospective Cohort Study. Journal of Alzheimer's Disease 2018;62(2):713-25.
55. Matsumoto L, Suzuki K, Mizuno Y, et al. Association of subclinical carotid atherosclerosis with immediate memory and other cognitive functions. Geriatrics & gerontology international 2018;18(1):65-71.
56. Muela HCS, Costa-Hong VA, Yassuda MS, et al. Higher arterial stiffness is associated with lower cognitive performance in patients with hypertension. Journal of Clinical Hypertension 2018;20(1):22-30.
57. Rouch L, Cestac P, Sallerin B, et al. Pulse wave velocity is associated with greater risk of dementia in mild cognitive impairment patients. Journal of Hypertension 2018;36 (Supplement 3):e340.
58. Mworozi K, Ameda F, Byanyima RK, et al. Carotid artery plaque detected on ultrasound is associated with impaired cognitive state in the elderly: A population-based study in Wakiso district, Uganda. Journal of Clinical Neuroscience 2019;68:194-200.
59. Kawasaki M, Arai Y, Hirata T, et al. Carotid atherosclerosis, cytomegalovirus infection, and cognitive decline in the very old: a community-based prospective cohort study. Age 2016;38(2):1-13.
60. Wendell CR, Zonderman AB, Metter EJ, et al. Carotid intimal medial thickness predicts cognitive decline among adults without clinical vascular disease. Stroke 2009;40(10):3180-5. doi: 10.1161/STROKEAHA.109.557280 [published Online First: 2009/08/01]
61. Buratti L, Balestrini S, Altamura C, et al. Markers for the Risk of Progression from Mild Cognitive Impairment to Alzheimer's Disease. Journal of Alzheimer's Disease 2015;45(3):883-90.
62. Mworozi K, Ameda F, Byanyima RK, et al. Carotid artery plaque detected on ultrasound is associated with impaired cognitive state in the elderly: A population-based study in Wakiso district, Uganda. J Clin Neurosci 2019;68:194-200. doi: 10.1016/j.jocn.2019.06.011 [published Online First: 2019/07/16]
63. Breteler MM. [New York, NY] :.
64. Grodstein F. Cardiovascular risk factors and cognitive function. Alzheimer's & dementia 2007;3(2):S16-S22. doi: 10.1016/j.jalz.2007.01.001

For peer review only - http://bmjopen.bmj.com/site/about/guidelines.xhtml

65. Skoog I. Status of Risk Factors for Vascular Dementia. Neuroepidemiology 1998;17(1):2-9. doi: 10.1159/000026147
66. Lai MMY, Sharman MJ, Ames DJ, et al. Relationship of established cardiovascular risk factors and peripheral biomarkers on cognitive function in adults at risk of cognitive deterioration. Journal of Alzheimer's disease 2020;74(1):163-71. doi: 10.3233/JAD-190953
67. Zheng K, Qian Y, Lin T, et al. Carotid intima–media thickness relative to cognitive impairment in dialysis patients, and their relationship with brain volume and cerebral small vessel disease. Therapeutic Advances in Chronic Disease 2020;11:2040622320953352. doi: 10.1177/2040622320953352
68. Van den Berg JF, Dogge B, Kist N, et al. Gender Differences in Cognitive Functioning in Older Alcohol-Dependent Patients. SUBST USE MISUSE 2017;52(5):574-80. doi: 10.1080/10826084.2016.1245341
69. Aartsen MJ, Martin M, Zimprich D. Gender Differences in Level and Change in Cognitive Functioning: Results from the Longitudinal Aging Study Amsterdam. Gerontology 2004;50(1):35-8.
70. Novella R, Olivera J. Gender Differences in Cognitive Abilities Among the Elderly Poor of Peru. Economía , 42 (83) pp 95-109 (2019) 2019
71. Touboul PJ, Hennerici MG, Meairs S, et al. Mannheim carotid intima-media thickness and plaque consensus (2004-2006-2011). An update on behalf of the advisory board of the 3rd, 4th and 5th watching the risk symposia, at the 13th, 15th and 20th European Stroke Conferences, Mannheim, Germany, 2004, Brussels, Belgium, 2006, and Hamburg, Germany, 2011. Cerebrovasc Dis 2012;34(4):290-6. doi: 10.1159/000343145 [published Online First: 2012/11/07]
72. Stein JH, Korcarz CE, Hurst RT, et al. Use of carotid ultrasound to identify subclinical vascular disease and evaluate cardiovascular disease risk: a consensus statement from the American Society of Echocardiography Carotid Intima-Media Thickness Task Force. Endorsed by the Society for Vascular Medicine. J Am Soc Echocardiogr 2008;21(2):93-111. doi: 10.1016/j.echo.2007.11.011
73. Cires-Drouet RS, Mozafarian M, Ali A, et al. Imaging of high-risk carotid plaques: ultrasound. Semin Vasc Surg 2017;30(1):44-53. doi: 10.1053/j.semvascsurg.2017.04.010 [published Online First: 2017/08/19]
74. Gupta A, Baradaran H, Schweitzer AD, et al. Carotid plaque MRI and stroke risk: a systematic review and meta-analysis. Stroke 2013;44(11):3071-7. doi: 10.1161/STROKEAHA.113.002551 [published Online First: 2013/08/31]
75. Piri R, Gerke O, Hoilund-Carlsen PF. Molecular imaging of carotid artery atherosclerosis with PET: a systematic review. Eur J Nucl Med Mol Imaging 2020;47(8):2016-25. doi: 10.1007/s00259-019-04622-y [published Online First: 2019/12/02]

For peer review only - http://bmjopen.bmj.com/site/about/guidelines.xhtml

Figure legends

Figure 1. PRISMA Flow chart of the strategy used to select articles for review

Figure 2. Forest plots of the association between carotid plaques and mini-mental state exam (MMSE).

Figure 3. Forest plots of the association between carotid intima-media thickness (cIMT) and mini-mental state exam (MMSE).

Figure 4. Funnel plots for each outcome evaluating publication bias. A. Studies assessed the association between carotid plaque and mini-mental state examination (MMSE) shows evidence of bias (p=0.02). B. Studies assessed the association between carotid intima-media thickness (IMT) and MMSE shows no evidence of bias (p=0.2).

For peer review only

For peer review only - http://bmjopen.bmj.com/site/about/guidelines.xhtml

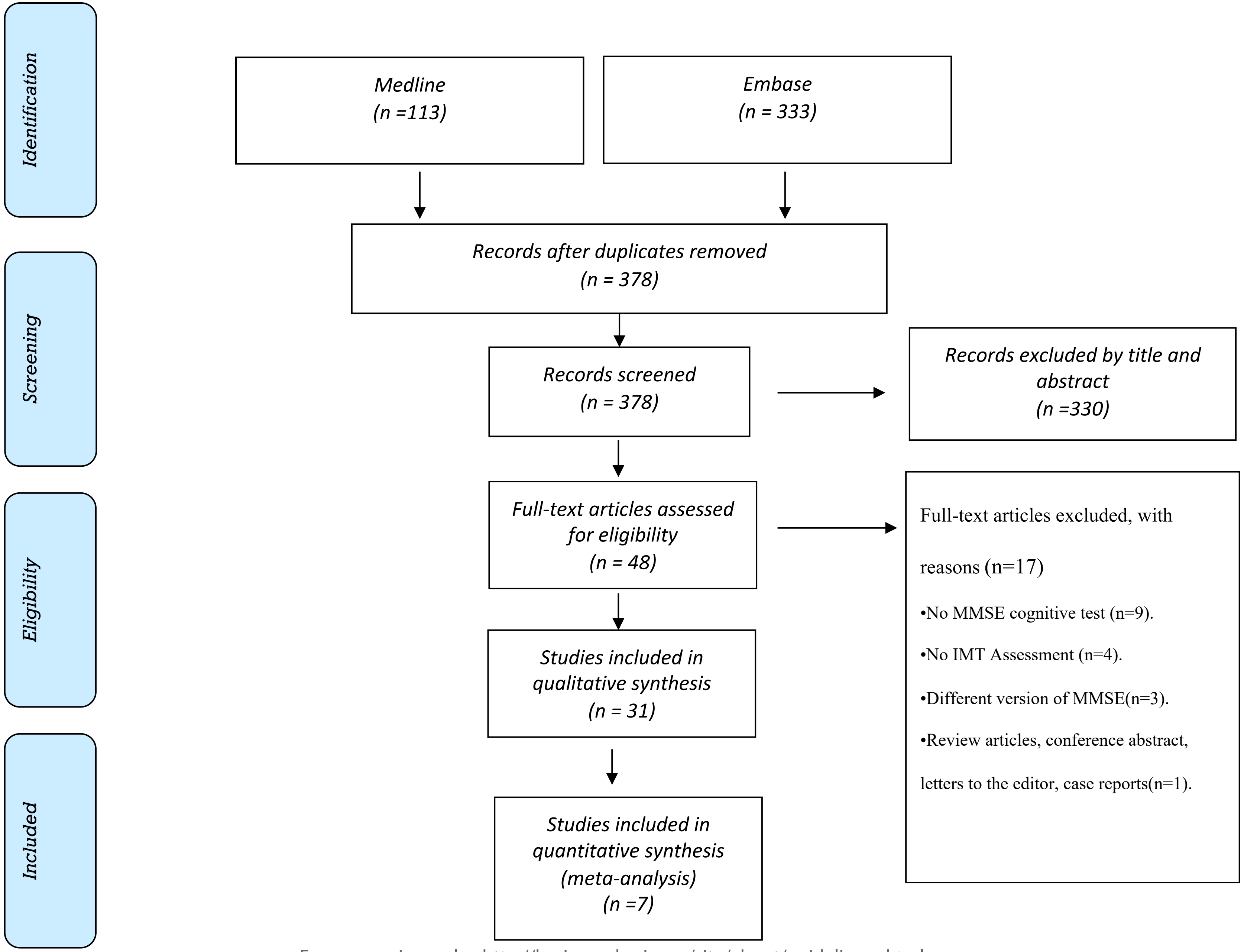

For peer review only - http://bmjopen.bmj.com/site/about/guidelines.xhtml

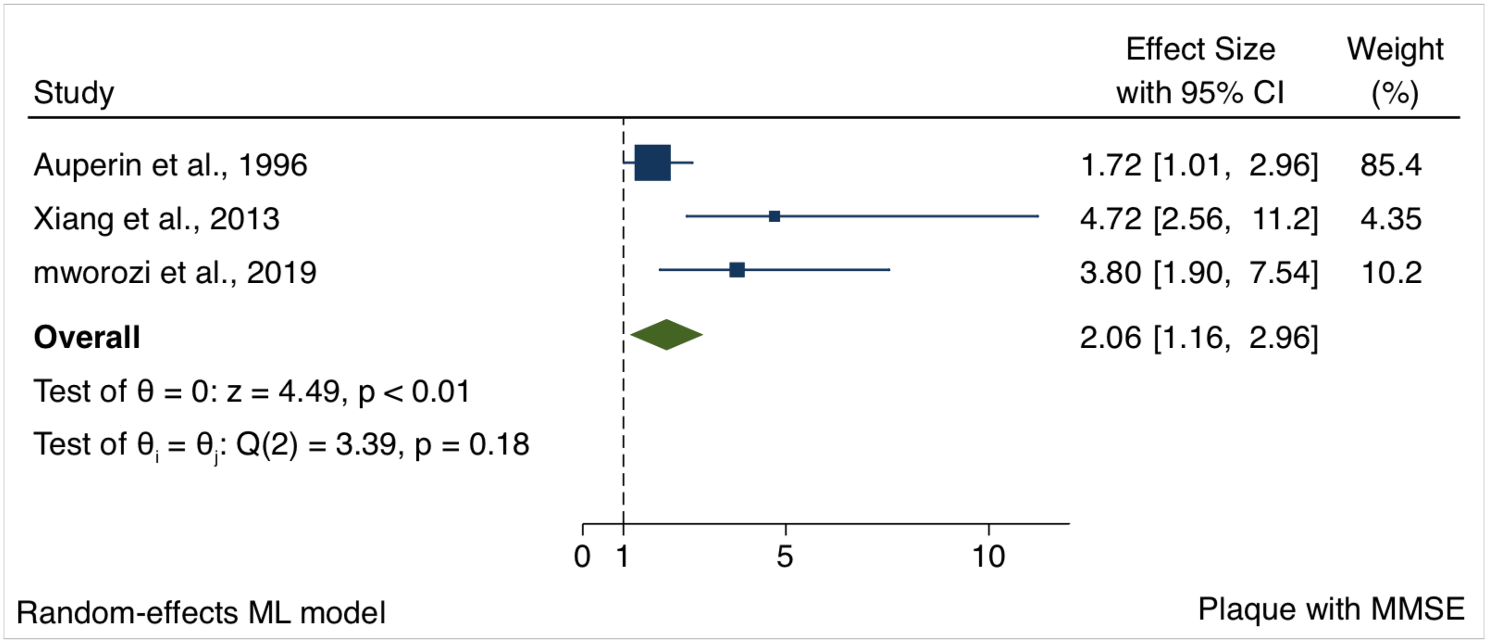
Study
Effect Size with 95% CI
Weight (%)
Auperin et al., 1996
1.72 [1.01, 2.96]
85.4
Xiang et al., 2013
4.72 [2.56, 11.2]
4.35
mworozi et al., 2019
3.80 [1.90, 7.54]
10.2
Overall
2.06 [1.16, 2.96]
Test of θ = 0: z = 4.49, p < 0.01
Test of θi = θj: Q(2) = 3.39, p = 0.18
0
1
5
10
Random-effects ML model
Plaque with MMSE

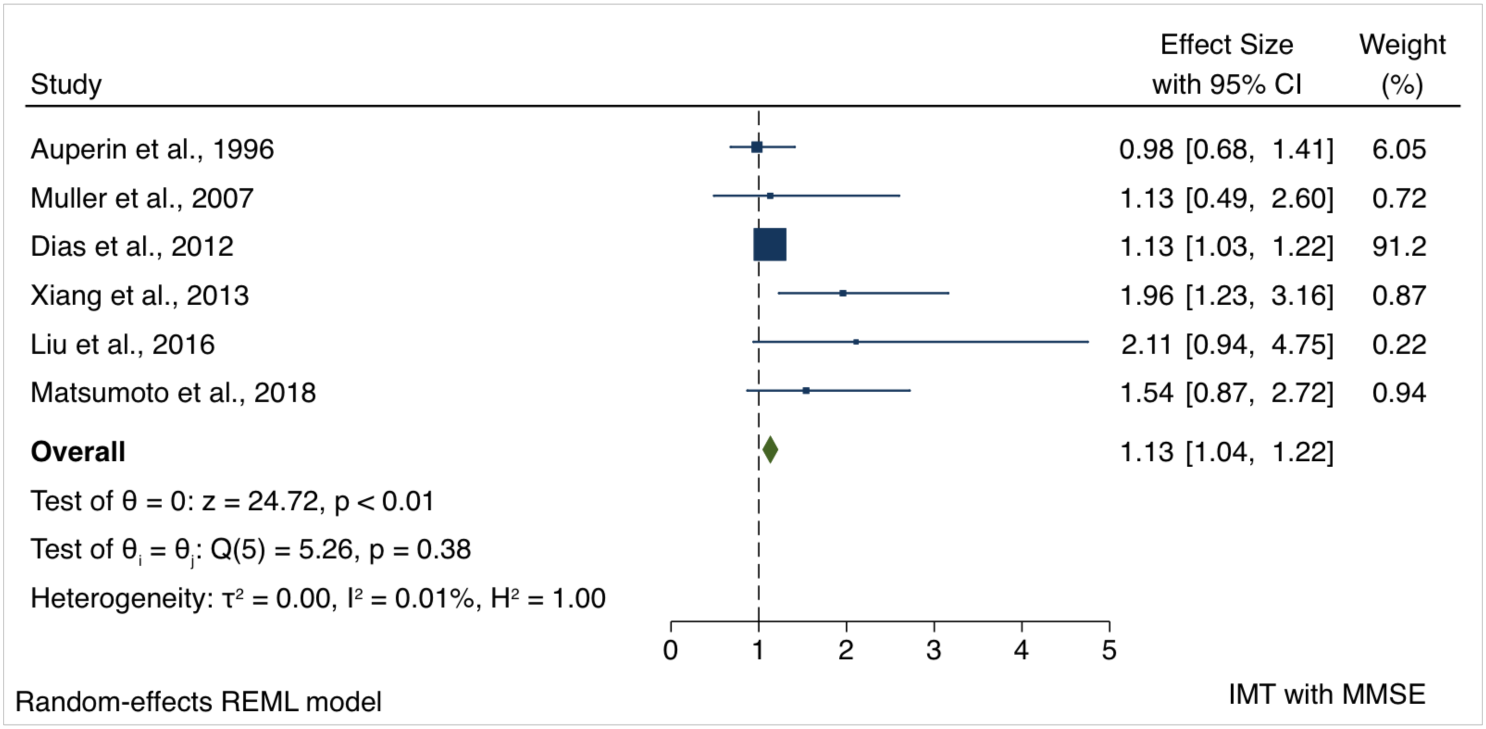
Study
Effect Size with 95% CI
Weight (%)
Auperin et al., 1996 0.98 [0.68, 1.41] 6.05
Muller et al., 2007 1.13 [0.49, 2.60] 0.72
Dias et al., 2012 1.13 [1.03, 1.22] 91.2
Xiang et al., 2013 1.96 [1.23, 3.16] 0.87
Liu et al., 2016 2.11 [0.94, 4.75] 0.22
Matsumoto et al., 2018 1.54 [0.87, 2.72] 0.94
Overall 1.13 [1.04, 1.22]
Test of θ = 0: z = 24.72, p < 0.01
Test of θi = θj: Q(5) = 5.26, p = 0.38
Heterogeneity: τ² = 0.00, I² = 0.01%, H² = 1.00
0 1 2 3 4 5
Random-effects REML model
IMT with MMSE

A, Carotid plaques **B, IMT**

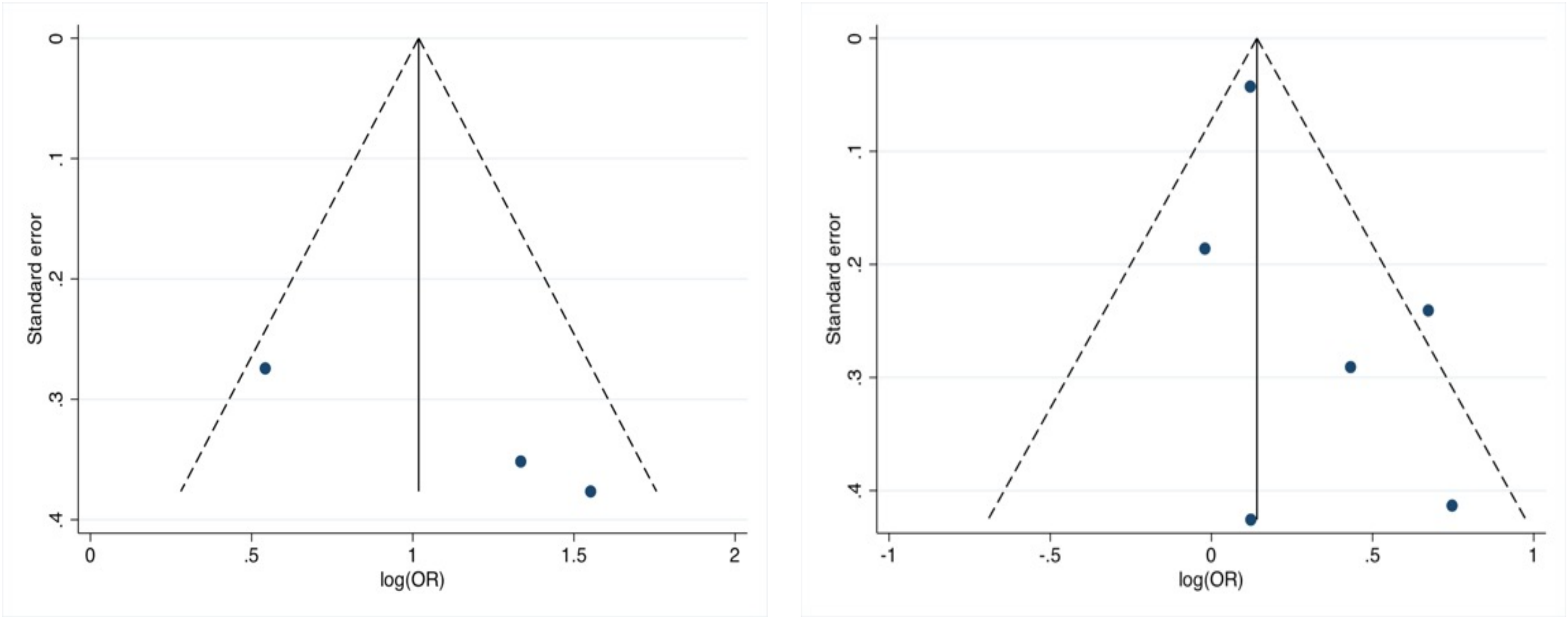

Database: Embase <1974 to 2020 February 28>
Search Strategy:
----
1 cognition/ (229751)
2 cognitive function*.tw. (86107)
3 dementia/ (113610)
4 Alzheimer disease/ (195937)
5 1 or 2 or 3 or 4 (508101)
6 (atheroscolerosis or intima media thickness or plaque).mp. (166750)
7 atherosclerosis/ (145056)
8 6 or 7 (287040)
9 5 and 8 (18670)
10 Carotid Arteries/ (47931)
11 carotid arter*.mp. (149782)
12 10 or 11 (149782)
13 9 and 12 (409)

****

2.

A Cross-Sectional Investigation of Cognition and Ultrasound-Based Vascular Strain Indices.
Meshram N.H., Jackson D., Varghese T., Mitchell C.C., Wilbrand S.M., Dempsey R.J., Hermann B.P.
Archives of clinical neuropsychology : the official journal of the National Academy of Neuropsychologists. 35 (1) (pp 46-55), 2019. Date of Publication: 24 Jan 2019.
AN: 630722963
OBJECTIVE: We examine the relationship between variability in the plaque strain distribution estimated using ultrasound with multiple cognitive domains including executive, language, visuospatial reasoning, and memory function.
METHOD(S): Asymptomatic (n = 42) and symptomatic (n = 34) patients with significant (>60%) carotid artery stenosis were studied for plaque instability using ultrasound strain imaging and multiple cognitive domains including executive, language, visuospatial reasoning, and memory

function. Correlation and ROC analyses were performed between ultrasound strain indices and cognitive function. Strain indices and cognition scores were also compared between symptomatic and asymptomatic patients to determine whether there are significant group differences.
RESULT(S): Association of high-strain distributions with dysexecutive function was observed in both asymptomatic and symptomatic patients. For memory, visuospatial, and language functions, the correlations between strain and cognition were weaker for the asymptomatic compared to symptomatic group.
CONCLUSION(S): Both asymptomatic and symptomatic patients demonstrate a relationship between vessel strain indices and executive function indicating that silent strokes and micro-emboli could initially contribute to a decline in executive function, whereas strokes and transient ischemic attacks may cause the further decline in other cognitive functions.
Copyright © The Author(s) 2019. Published by Oxford University Press. All rights reserved. For permissions, please e-mail: journals.permissions@oup.com.
PMID
30805597 [http://www.ncbi.nlm.nih.gov/pubmed/?term=30805597]
Institution
(Meshram, Varghese) Department of Medical Physics, University of Wisconsin School of Medicine and Public Health, WI, Madison, United States (Meshram, Varghese) Department of Electrical and Computer Engineering, University of Wisconsin-Madison, WI, Madison, United States
(Jackson, Hermann) Department of Neurology, University of Wisconsin School of Medicine and Public Health, WI, Madison, United States
(Mitchell) Department of Medicine, University of Wisconsin School of Medicine and Public Health, WI, Madison, United States
(Wilbrand, Dempsey) Department of Neurological Surgery, University of Wisconsin School of Medicine and Public Health, WI, Madison, United States
Publisher
NLM (Medline)
Emtree Heading
adult; article; *carotid artery obstruction; clinical article; *cognitive defect; controlled study; correlation analysis; *early diagnosis; *executive function; female; human; language; male; memory; microembolism; *neuropsychological test; reasoning; receiver operating characteristic; *transient ischemic attack; *ultrasound.
Other Index Terms
adult [m]; article [m]; *carotid artery obstruction [m]; clinical article [m]; *cognitive defect [m]; controlled study [m]; correlation analysis [m]; *early diagnosis [m]; *executive function [m]; female [m]; human [m]; language [m]; male [m]; memory [m]; microembolism [m]; *neuropsychological

test [m]; reasoning [m]; receiver operating characteristic [m]; *transient ischemic attack [m]; *ultrasound [m].

Link to the Ovid Full Text or citation:
Click here for full text options

Link to the External Link Resolver:
SFX

4.

Association of blood lipids, atherosclerosis and statin use with dementia and cognitive impairment after stroke: A systematic review and meta-analysis.
Yang Z., Wang H., Edwards D., Ding C., Yan L., Brayne C., Mant J.
Ageing Research Reviews. 57 (no pagination), 2020. Article Number: 100962. Date of Publication: January 2020.
AN: 2003369983
Background: Trial and observational evidence is conflicting in terms of the association of blood lipids, atherosclerosis and statin use with dementia and cognitive impairment in the general population. It is uncertain whether the associations occur in stroke patients, who are at known higher risk of cognitive decline. This systematic review was to synthesize the evidence for these associations among stroke patients.
Method(s): MEDLINE, EMBASE, the Cochrane Library and trial registries were searched. We included randomized controlled trials (RCTs) or observational cohort studies conducted among patients with stroke and reported on the association of blood lipids, atherosclerosis or statin use with dementia or cognitive impairment. Meta-analysis was conducted separately for crude and maximally adjusted odds ratios (ORs) and hazard ratios (HRs).
Result(s): Of 18,026 records retrieved, 56 studies (one RCT and 55 cohort studies) comprising 38,423 stroke patients were included. For coronary heart disease, the pooled OR of dementia and cognitive impairment was 1.32 (95%CI 1.10-1.58, n = 15 studies, I2 = 0%) and 1.23 (95%CI 0.99-1.54, n = 14, I2 = 26.9%), respectively. Peripheral artery disease was associated with dementia (OR 3.59, 95%CI 1.47-8.76, n = 2, I2 = 0%) and cognitive impairment (OR 2.70, 95%CI 1.09-6.69, n = 1). For carotid stenosis, the pooled OR of dementia and cognitive impairment was 2.67 (95%CI 0.83-8.62,

For peer review only - http://bmjopen.bmj.com/site/about/guidelines.xhtml

n = 3, I2 = 77.9%) and 3.34 (95%CI 0.79-14.1, n = 4, I2 = 96.6%), respectively. For post-stroke statin use, the pooled OR of dementia and cognitive impairment was 0.89 (95%CI 0.65-1.21, n = 1) and 0.56 (95%CI 0.46-0.69, n = 3, I2 = 0%), respectively. No association was observed for hypercholesterolemia. These results were mostly consistent with adjusted ORs or HRs, which were reported from limited evidence.

Conclusion(s): Atherosclerosis was associated with an increased risk of post-stroke dementia. Post-stroke statin use was associated with decreased risk of cognitive impairment. To confirm whether or not statins confer advantages in the post-stroke population in terms of preventing cognitive decline over and above their known effectiveness in reducing risk of further vascular events, further stroke trials including cognitive assessment and observational analyses adjusted for key confounders, focusing on key subgroups or statin use patterns are required.

Copyright © 2019 Elsevier B.V.

PMID

31505259 [http://www.ncbi.nlm.nih.gov/pubmed/?term=31505259]

Institution

(Yang, Edwards, Mant) Primary Care Unit, Department of Public Health and Primary Care, School of Clinical Medicine, University of Cambridge, Cambridge, United Kingdom (Wang, Brayne) Institute of Public Health, School of Clinical Medicine, University of Cambridge, Cambridge, United Kingdom

(Ding) Research Department of Epidemiology and Public Health, University College London, London, United Kingdom

(Yan) Department of Epidemiology and Biostatistics, School of Public Health, Imperial College London, London, United Kingdom

(Yan) MRC-PHE Centre for Environment and Health, King's College London, London, United Kingdom

Publisher

Elsevier Ireland Ltd

Emtree Heading

*atherosclerosis; carotid artery obstruction; *cerebrovascular accident; Cochrane Library; *cognitive defect; cohort analysis; *dementia; disease association; Embase; human; hypercholesterolemia; ischemic heart disease; *lipid blood level; Medline; meta analysis; observational study; peripheral occlusive artery disease; randomized controlled trial (topic); review; stroke patient; systematic review; *hydroxymethylglutaryl coenzyme A reductase inhibitor; *lipid/ec [Endogenous Compound].

Drug Index Terms

*hydroxymethylglutaryl coenzyme A reductase inhibitor; *lipid / *endogenous compound.

Other Index Terms

For peer review only - http://bmjopen.bmj.com/site/about/guidelines.xhtml

*atherosclerosis; carotid artery obstruction; *cerebrovascular accident; Cochrane Library; *cognitive defect; cohort analysis; *dementia; disease association; Embase; human; hypercholesterolemia; ischemic heart disease; *lipid blood level; Medline; meta analysis; observational study; peripheral occlusive artery disease; randomized controlled trial (topic); Review; stroke patient; systematic review.

Link to the Ovid Full Text or citation:
Click here for full text options

Link to the External Link Resolver:
SFX

6.

Carotid Intima-media Thickness, Cognitive Performance and Cognitive Decline in Stroke-free Middle-aged and Older Adults. The Atahualpa Project.
Del Brutto O.H., Mera R.M., Recalde B.Y., Del Brutto V.J.
Journal of Stroke and Cerebrovascular Diseases. 29 (2) (no pagination), 2020. Article Number: 104576. Date of Publication: February 2020.
AN: 2004217925
Background: Little is known on factors influencing cognitive function in rural communities. Using the Atahualpa Project cohort, we aimed to assess whether the carotid intima-media thickness (cIMT) - used as a surrogate of extracranial carotid atherosclerosis - is associated with cognitive performance and further decline in community-dwelling adults living in a rural setting.
Method(s): The study included Atahualpa residents aged greater than or equal to 40 years who had ultrasound examination of the extracranial carotid arteries and a baseline Montreal Cognitive Assessment (MoCA), as well as the subset of individuals who also had a follow-up MoCA at least 1 year after baseline. Relationship between cIMT and cognitive function was assessed by means of generalized linear and longitudinal models, adjusted for relevant covariates. Mediation analysis was utilized to establish the proportion of the effect between increased cIMT and cognitive performance, which is mediated by age.
Result(s): A total of 561 individuals were included for the cross-sectional study, and 510 of them were assessed for the prospective cohort. Univariate analysis showed a significant association

For peer review only

between increased cIMT and worse cognitive performance (P < .001), which vanishes after considering the effect of age and low scholarity. Causal mediation analysis confirms that age captures 82.6% (95% C.I.: 63.9% to 100%) of the effect of this association. There was no relationship between increased cIMT and cognitive decline in the follow-up.
Conclusion(s): In this rural population, the association between increased cIMT and cognitive dysfunction is mostly mediated by increasing age.
Copyright © 2019 Elsevier Inc.
PMID
31839546 [http://www.ncbi.nlm.nih.gov/pubmed/?term=31839546]
Author NameID
Del Brutto, Oscar H.; ORCID: http://orcid.org/0000-0003-1917-8805
Institution
(Del Brutto) School of Medicine, Universidad Espiritu Santo - Ecuador, Samborondon, Ecuador
(Mera) Department of Epidemiology, Gilead Sciences, Inc., Foster City, CA, United States
(Recalde) Community Center, the Atahualpa Project, Atahualpa, Ecuador
(Del Brutto) Department of Neurology, Miller School of Medicine, University of Miami, Miami, FL, United States
Publisher
W.B. Saunders
Emtree Heading
adult; age; aged; *arterial wall thickness; article; carotid artery; *cerebrovascular accident; *cognition; *cognitive defect; cohort analysis; cross-sectional study; Ecuador; female; follow up; human; longitudinal study; major clinical study; male; Montreal cognitive assessment; priority journal; univariate analysis.
Other Index Terms
adult; age; aged; *arterial wall thickness; Article; carotid artery; *cerebrovascular accident; *cognition; *cognitive defect; cohort analysis; cross-sectional study; Ecuador; female; follow up; human; longitudinal study; major clinical study; male; Montreal cognitive assessment; priority journal; univariate analysis.

Link to the Ovid Full Text or citation:
Click here for full text options

Link to the External Link Resolver:
SFX

For peer review only

8.

Cerebral hemodynamics and cognitive function in patients with atherosclerotic lesions of brachiocephalic arteries.

Mushba A., Vinogradov O., Kuznetsov A., Vachromeeva M., Kankia-Denisenko E., Batrashov V.

Journal of the Neurological Sciences. Conference: World Congress of Neurology (WCN 2019). United Arab Emirates. 405 (Supplement) (pp 71), 2019. Date of Publication: 15 October 2019.

AN: 2003654554

Introduction: Steno-occlusive atherosclerotic lesions of extra- and intracranial cerebral arteries is regarded as the most significant factor for ischemic stroke risk. The greater the percentage of stenosis, the higher the risk of stroke. However it is unclear the impact of macroangiopathy (stenos in the carotid arteries) in the inhibition of cognitive functions.

Material(s) and Method(s): In study were included 2 groups of patients: group 1 - patients with asymptomatic carotid stenosis >50% (30 patients); group 2 - patients with carotid stenosis <50% (30 patients). In the group with asymptomatic carotid stenosis >50% were 22 (73.3%) male and 8 (26.7%) women; mean age of patients was 62.5 +/- 1.3 years. In the group with carotid stenosis <50% - 19 (63.3%) male and 11 (36.6%) women; mean age of patients was 64.5+/-1.4 years. All patients underwent assessment of scale of cognitive impairment (MMSE, MoSa), cognitive evoked potentials (P-300), duplex scanning of brachiocephalic arteries, transcranial duplex scan, brain MRI and Single-photon emission computed tomography (SPECT) with 99mTc-HMPAO.

Result(s): Cerebral perfusion according to SPECT in groups 1 and 2 were 76.2% and 79.2%, respectively; differences were not statistically significant (p > 0.05). MMSE and MoCa in groups 1 and 2 appeared to be 25.7 +/- 0.5 and 26.8 +/- 0.5 (p > 0.05); 26.5 +/- 0.4 and 27.5 +/- 0.4 (p > 0.05), respectively. According to cognitive evoked potentials, latencyP-300 in groups were 406 +/- 0.6 ms and 371 +/- 0.6 ms, respectively (p > 0.05). No correlation between severity of stenosis and cognitive functions in groups 1 and 2 were seen (r < 0.3).

Conclusion(s): Macroangiopathy of cerebral arteries (carotid stenosis) is not associated with cognitive deterioration.

Copyright © 2019

Institution

(Mushba, Vinogradov, Kuznetsov, Vachromeeva, Kankia-Denisenko, Batrashov) National Center of Cerebrovascular Disorders, The National Pirogov Medical Surgical Center, Moscow, Russian Federation

Publisher

Elsevier B.V.
Emtree Heading
adult; *atherosclerosis; *brachiocephalic trunk; brain artery; brain ischemia; brain perfusion; carotid artery obstruction; clinical article; *cognitive defect; controlled study; evoked response; female; *hemodynamics; human; male; mental deterioration; middle aged; Mini Mental State Examination; nuclear magnetic resonance imaging; single photon emission computed tomography; polyacrylamide; conference abstract.
Candidate Terms
conference abstract [other term].
Drug Index Terms
polyacrylamide.
Other Index Terms
adult; *atherosclerosis; *brachiocephalic trunk; brain artery; brain ischemia; brain perfusion; carotid artery obstruction; clinical article; *cognitive defect; controlled study; evoked response; female; *hemodynamics; human; male; mental deterioration; middle aged; Mini Mental State Examination; nuclear magnetic resonance imaging; single photon emission computed tomography.

Link to the Ovid Full Text or citation:
Click here for full text options

Link to the External Link Resolver:
SFX

11.

Cortical cerebral microinfarcts on 3t magnetic resonance imaging in patients with carotid artery stenosis.
Takasugi J., Miwa K., Watanabe Y., Okazaki S., Todo K., Sasaki T., Sakaguchi M., Mochizuki H.
Stroke. 50 (3) (pp 639-644), 2019. Date of Publication: 01 Mar 2019.
AN: 627432469
Background and Purpose - Carotid artery stenosis is common in the elderly and contributes to cognitive impairment and dementia. Cortical cerebral microinfarcts (CMIs) play an important role in vascular cognitive impairment and dementia. We aimed to investigate the association between

For peer review only - http://bmjopen.bmj.com/site/about/guidelines.xhtml

CMIs on 3T magnetic resonance imaging and clinical and radiological features, including plaque morphology, and cognitive function in patients with carotid stenosis. Methods - Eighty-nine patients with >30% carotid stenosis on ultrasound were prospectively enrolled, and underwent brain and carotid artery magnetic resonance imaging. CMIs were rated according to predetermined criteria based on 3D-double inversion recovery and fluid-attenuated inversion recovery images. Results - CMIs were identified in 26 patients (29%; median number 0, range 0-9). Poisson regression models adjusted for age and sex revealed that CMIs were associated with intraplaque hemorrhage (rate ratio, 1.95; 95% CI, 1.26-3.18), lacunar infarcts (rate ratio, 1.54; 95% CI, 1.00-2.44), and cortical infarcts (rate ratio, 3.22; 95% CI, 2.20-5.00). These associations were also observed in asymptomatic patients (n=64). Of 81 patients with unilateral carotid stenosis, the prevalence and number of CMIs were significantly higher in the hemisphere ipsilateral to the carotid stenosis than in the contralateral hemisphere (P=0.005 and P<0.001, respectively). The presence of CMIs was associated with poor cognitive function. Conclusions - Our results indicate that vulnerable carotid plaque increases the risk of CMIs and subsequent cognitive impairment. Carotid atherosclerosis could be a potential therapeutic target for cognitive impairment.
Copyright © 2019 American Heart Association, Inc.
PMID
30744544 [http://www.ncbi.nlm.nih.gov/pubmed/?term=30744544]
Institution
(Takasugi, Miwa, Okazaki, Todo, Sasaki, Sakaguchi, Mochizuki) Department of Neurology, Osaka University, Graduate School of Medicine, 2-2 Yamadaoka, Suita, Osaka 565-0871, Japan
(Watanabe) Department of Diagnostic and Interventional Radiology, Osaka University, Graduate School of Medicine, Japan
(Miwa) Department of Cerebrovascular Medicine, National Cerebral and Cardiovascular Center, Osaka, Japan
Publisher
Lippincott Williams and Wilkins (E-mail: kathiest.clai@apta.org)
Emtree Heading
aged; article; atherosclerotic plaque; atrial fibrillation; brain function; *brain infarction/co [Complication]; *brain infarction/di [Diagnosis]; carotid artery; *carotid artery obstruction; carotid atherosclerosis; cognitive defect; dementia; diabetes mellitus; female; human; hyperlipidemia; hypertension; ischemic heart disease; major clinical study; male; morphology; neuroimaging; neuropsychological test; *nuclear magnetic resonance imaging; priority journal; transient ischemic attack; ultrasound; antithrombocytic agent; hydroxymethylglutaryl coenzyme A reductase inhibitor; nuclear magnetic resonance scanner; *cortical cerebral microinfarct/co [Complication]; *cortical cerebral microinfarct/di [Diagnosis].
Candidate Terms

For peer review only

*cortical cerebral microinfarct / *complication / *diagnosis [other term].

Device Index Terms

nuclear magnetic resonance scanner.

Drug Index Terms

antithrombocytic agent; hydroxymethylglutaryl coenzyme A reductase inhibitor.

Other Index Terms

aged; Article; atherosclerotic plaque; atrial fibrillation; brain function; *brain infarction / *complication / *diagnosis; carotid artery; *carotid artery obstruction; carotid atherosclerosis; cognitive defect; dementia; diabetes mellitus; female; human; hyperlipidemia; hypertension; ischemic heart disease; major clinical study; male; morphology; neuroimaging; neuropsychological test; *nuclear magnetic resonance imaging; priority journal; transient ischemic attack; ultrasound.

Link to the Ovid Full Text or citation:

Click here for full text options

Link to the External Link Resolver:

SFX

14.

Carotid atherosclerosis and dementia - Inflammatory markers and marker of macrophage activation. MIAZDZYCA TETNIC SZYJNYCH A OTEPIENIE - CZYNNIKI ZAPALNE I WSKAZNIK AKTYWACJI MAKROFAGOW <MIAZDZYCA TETNIC SZYJNYCH A OTEPIENIE - CZYNNIKI ZAPALNE I WSKAZNIK AKTYWACJI MAKROFAGOW.>

Wehr H., Lugowska A., Graban A., Wisniewska A., Hetmanczyk-Sawicka K., Witkowski G., Ryglewicz D., Lipczynska-Lojkowska W., Bochynska A., Gugala-Iwaniuk M., Szirkowiec W., Bednarska-Makaruk M.E.

Postepy Psychiatrii i Neurologii. 28 (3) (pp 169-175), 2019. Date of Publication: 2019.

AN: 2003669181

Purpose: To assess the relationship between serum inflammatory markers (interleukin 6, high sensitivity C-reactive protein [hsCRP] and chitotriosidase activity) and the extent of carotid atherosclerotic lesions in subjects with various types of dementia.

For peer review only

Method(s): Four hundreds persons with dementia (166 diagnosed as probable Alzheimer's disease, 85 as vascular dementia [VaD], 149 as mixed dementia [MD] and 180 controls) were observed. In all persons carotid intima-media thickness (IMT) was measured and all were subjected to a general medical and neurological evaluation, neuroimaging examination (computed tomography and magnetic resonance) and comprehensive neuropsychological examination. The pro-inflammatory markers interleukin-6 (IL-6) and hsCRP, and anti-inflammatory markers (paraoxonase-1 activity and HDL cholesterol level), were determined in blood serum. Chitotriosidase activity - an indicator of chronic macrophage activation - was also determined.
Result(s): A higher frequency of carotid atherosclerosis was observed in the whole group of dementia and in the VaD and MD groups as compared to the controls. A significant positive correlation of IMT with the inflammatory indicators IL-6 and hsCRP was found. A negative correlation of IMT with inflammatory markers (paraoxonase-1 activity and HDL cholesterol level) was observed. Chitotriosidase activity was significantly elevated, as compared with the controls, in the whole group with dementia and in the MD group, and depended on the degree of carotid stenosis.
Conclusion(s): Serum IL-6, hsCRP and chitotriosidase activity can be considered as markers of the extent of carotid arteriosclerosis in dementia, especially in patients with dementia with vascular lesions. High chitotriosidase activity may indicate chronic macrophage activation in the course of dementia development.
Copyright © 2019 Termedia Publishing House Ltd.. All rights reserved.
Institution
(Wehr, Lugowska, Wisniewska, Hetmanczyk-Sawicka, Szirkowiec, Bednarska-Makaruk) Department of Genetics, Institute of Psychiatry and Neurology, Warsaw, Poland (Graban, Witkowski, Ryglewicz, Lipczynska-Lojkowska, Bochynska, Gugala-Iwaniuk) First Department of Neurology, Institute of Psychiatry and Neurology, Warsaw, Poland
Publisher
Termedia Publishing House Ltd. (Kleeberqa St.2, Poznan 61-615, Poland)
Emtree Heading
adult; aged; Alzheimer disease; arterial wall thickness; article; carotid artery bifurcation; carotid artery obstruction; *carotid atherosclerosis; clinical assessment; computer assisted tomography; controlled study; *dementia; DSM-IV; enzyme activity; female; high density lipoprotein cholesterol level; human; ICD-10; *macrophage activation; major clinical study; male; Mini Mental State Examination; multiinfarct dementia; neuroimaging; neuropsychological test; nuclear magnetic resonance imaging; very elderly; *aryldialkylphosphatase 1/ec [Endogenous Compound]; *C reactive protein/ec [Endogenous Compound]; *chitotriosidase/ec [Endogenous Compound]; *high density lipoprotein cholesterol/ec [Endogenous Compound]; *interleukin 6/ec [Endogenous Compound].

Drug Index Terms
*aryldialkylphosphatase 1 / *endogenous compound; *C reactive protein / *endogenous compound; *chitotriosidase / *endogenous compound; *high density lipoprotein cholesterol / *endogenous compound; *interleukin 6 / *endogenous compound.
Other Index Terms
adult; aged; Alzheimer disease; arterial wall thickness; Article; carotid artery bifurcation; carotid artery obstruction; *carotid atherosclerosis; clinical assessment; computer assisted tomography; controlled study; *dementia; DSM-IV; enzyme activity; female; high density lipoprotein cholesterol level; human; ICD-10; *macrophage activation; major clinical study; male; Mini Mental State Examination; multiinfarct dementia; neuroimaging; neuropsychological test; nuclear magnetic resonance imaging; very elderly.

Link to the Ovid Full Text or citation:
Click here for full text options

Link to the External Link Resolver:
SFX

15.

Assessment of cognitive function in female rheumatoid arthritis patients: associations with cerebrovascular pathology, depression and anxiety.
Olah C., Kardos Z., Andrejkovics M., Szarka E., Hodosi K., Domjan A., Sepsi M., Sas A., Kostyal L., Fazekas K., Florian A., Lukacs K., Miksi A., Barath Z., Kerekes G., Pentek M., Valikovics A., Tamasi L., Bereczki D., Szekanecz Z.
Rheumatology International. (no pagination), 2019. Date of Publication: 2019.
AN: 2003469015
We assessed cognitive function of female rheumatoid arthritis (RA) patients and analyze the determinants, with special focus on cerebrovascular morphology. Sixty methotrexate (MTX-) or biologic-treated RA patients and 39 healthy controls were included in a cross-sectional study. Smoking habits, alcohol intake and time spent in education were recorded. Standard measures were performed to assess cognitive function (Montreal Cognitive Assessment, MOCA; Trail Making Test, TMT; Victoria Stroop Test, VST; Wechsler Adult Intelligence Scale, WAIS; Benton Visual

For peer review only

Retention test, BVRT), depression (Beck Depression Inventory, BDI), anxiety (State-Trait Anxiety Inventory, STAIT/S) and general health status (Short Form 36, SF-36). Mean disease activity (28-joint Disease Activity Score, mDAS28; erythrocyte sedimentation rate, mESR; C-reactive protein, mCRP) of the past 12 months was calculated; anti-cyclic citrullinated peptide (CCP) and rheumatoid factor (RF) were assessed. Cerebral vascular lesions and atrophy, carotid intima-media thickness (cIMT) and plaques, as well as median cerebral artery (MCA) circulatory reserve capacity (CRC) were assessed by brain magnetic resonance imaging (MRI), carotid ultrasound and transcranial Doppler, respectively. Cognitive function tests showed impairment in RA vs controls. Biologic- vs MTX-treated subgroups differed in TMT-A. Correlations were identified between cognitive function and depression/anxiety tests. WAIS, STAIS, STAIT and BDI correlated with most SF-36 domains. Numerous cognitive tests correlated with age and lower education. Some also correlated with disease duration, mESR and mDAS28. Regarding vascular pathophysiology, cerebral vascular lesions were associated with VST-A, carotid plaques with multiple cognitive parameters, while MCA and CRC with MOCA, BVRT and BDI. RA patients have significant cognitive impairment. Cognitive dysfunction may occur together with or independently of depression/anxiety. Older patients and those with lower education are at higher risk to develop cognitive impairment. Cognitive screening might be a useful tool to identify subgroups to be further investigated for cerebrovascular pathologies.

Copyright © 2019, The Author(s).

PMID

31555886 [http://www.ncbi.nlm.nih.gov/pubmed/?term=31555886]

Institution

(Olah, Szarka) Department of Neurosurgery, Borsod County Teaching Hospital, Miskolc, Hungary

(Kardos, Fazekas, Florian, Lukacs, Miksi, Barath, Tamasi) Department of Rheumatology, Borsod County Teaching Hospital, Miskolc, Hungary

(Andrejkovics) Institute of Behavioural Sciences, Faculty of Public Health, University of Debrecen, Debrecen, Hungary

(Andrejkovics) Department of Oncoradiology, Szabolcs-Szatmar-Bereg County Hospitals, Josa Andras Teaching Hospital, Nyiregyhaza, Hungary

(Hodosi, Domjan, Szekanecz) Department of Rheumatology, Faculty of Medicine, University of Debrecen, Debrecen, Hungary

(Sepsi, Kostyal) Department of Radiology, Borsod County Teaching Hospital, Miskolc, Hungary

(Sas, Valikovics) Department of Neurology, Borsod County Teaching Hospital, Miskolc, Hungary

(Kerekes) Department of Angiology, Faculty of Medicine, University of Debrecen, Debrecen, Hungary

(Pentek) Department of Health Economics, Corvinus University, Budapest, Hungary

(Pentek) Department of Rheumatology, Flor Ferenc County Hospital, Kistarcsa, Hungary

For peer review only - http://bmjopen.bmj.com/site/about/guidelines.xhtml

(Bereczki) Department of Neurology, Semmelweis University, Budapest, Hungary

Publisher
Springer Verlag (E-mail: service@springer.de)

Emtree Heading

adult; age; alcohol consumption; *arterial wall thickness; article; atrophy; Beck Depression Inventory; Benton visual retention test; brain artery; *carotid atherosclerosis; *cognitive defect; controlled study; cross-sectional study; DAS28; disease course; drug therapy; education; erythrocyte sedimentation rate; female; human; major clinical study; Montreal cognitive assessment; nuclear magnetic resonance imaging; quality of life; *rheumatoid arthritis; Short Form 36; smoking habit; *State Trait Anxiety Inventory; Stroop test; trail making test; *transcranial doppler; Wechsler adult intelligence scale; C reactive protein; cyclic citrullinated peptide antibody; endogenous compound; *methotrexate; rheumatoid factor.

Drug Index Terms

C reactive protein [m]; cyclic citrullinated peptide antibody [m]; endogenous compound [m]; *methotrexate [m]; rheumatoid factor [m].

Other Index Terms

adult [m]; age [m]; alcohol consumption [m]; *arterial wall thickness [m]; article [m]; atrophy [m]; Beck Depression Inventory [m]; Benton visual retention test [m]; brain artery [m]; *carotid atherosclerosis [m]; *cognitive defect [m]; controlled study [m]; cross-sectional study [m]; DAS28 [m]; disease course [m]; drug therapy [m]; education [m]; erythrocyte sedimentation rate [m]; female [m]; human [m]; major clinical study [m]; Montreal cognitive assessment [m]; nuclear magnetic resonance imaging [m]; quality of life [m]; *rheumatoid arthritis [m]; Short Form 36 [m]; smoking habit [m]; *State Trait Anxiety Inventory [m]; Stroop test [m]; trail making test [m]; *transcranial doppler [m]; Wechsler adult intelligence scale [m].

Link to the Ovid Full Text or citation:

Click here for full text options

Link to the External Link Resolver:

SFX

22.

For peer review only

Carotid atherosclerotic plaque instability and cognition determined by ultrasound-measured plaque strain in asymptomatic patients with signifcant stenosis.

Dempsey R.J., Varghese T., Jackson D.C., Wang X., Meshram N.H., Mitchell C.C., Hermann B.P., Johnson S.C., Berman S.E., Wilbrand S.M.

Journal of Neurosurgery. 128 (1) (pp 111-119), 2018. Date of Publication: January 2018.

AN: 620124652

OBJECTIVE This article describes the use of ultrasound measurements of physical strain within carotid atherosclerotic plaques as a measure of instability and the potential for vascular cognitive decline, microemboli, and white matter changes. METHODS Asymptomatic patients with signifcant (>*60%) carotid artery stenosis were studied for dynamic measures of plaque instability, presence of microemboli, white matter changes, and vascular cognitive decline in comparison with normative controls and premorbid state. RESULTS Although classically asymptomatic, these patients showed vascular cognitive decline. The degree of strain instability measured within the atherosclerotic plaque directly predicted vascular cognitive decline in these patients thought previously to be asymptomatic according to classic criteria. Furthermore, 26% of patients showed microemboli, and patients had twice as much white matter hyperintensity as controls. CONCLUSIONS These data show that physical measures of plaque instability are possible through interpretation of ultrasound strain data during pulsation, which may be more clinically relevant than solely measuring degree of stenosis. The data also highlight the importance of understanding that the defnition of symptoms should not be limited to motor, speech, and vision function but underscore the role of vascular cognitive decline in the pathophysiology of carotid atherosclerotic disease.

Copyright © AANS 2018.

PMID

28298048 [http://www.ncbi.nlm.nih.gov/pubmed/?term=28298048]

Institution

(Dempsey, Wilbrand) Department of Neurological Surgery, University of Wisconsin School of Medicine and Public Health, 600 Highland Ave., Madison, WI 53792, United States (Varghese, Meshram) Department of Medical Physics, University of Wisconsin School of Medicine and Public Health, United States

(Jackson) Wisconsin Surgical Outcomes Research Program, Department of Surgery, University of Wisconsin School of Medicine and Public Health, Madison, WI, United States

(Wang) Rutgers Cancer Institute of New Jersey, New Brunswick, NJ, United States

(Mitchell) Department of Medicine, Cardiovascular Medicine Division, United States

(Hermann) Department of Neurology, University of Wisconsin School of Medicine and Public Health, United States

For peer review only - http://bmjopen.bmj.com/site/about/guidelines.xhtml

(Johnson, Berman) Alzheimer's Disease Research Center, University of Wisconsin School of Medicine and Public Health, Waisman Laboratory for Brain Injury and Behavior, University of Wisconsin-Madison, Geriatric Research Education and Clinical Center, William S. Middleton Veterans Hospital, Madison, WI, United States

Publisher

American Association of Neurological Surgeons

Emtree Heading

adult; aged; article; *asymptomatic disease/di [Diagnosis]; *carotid artery obstruction/di [Diagnosis]; *carotid atherosclerosis/di [Diagnosis]; clinical article; *cognition; controlled clinical trial; controlled study; female; human; male; mental deterioration; microembolism; middle cerebral artery occlusion/di [Diagnosis]; nuclear magnetic resonance imaging; patient identification; priority journal; transcranial Doppler ultrasonography; very elderly; white matter; nuclear magnetic resonance scanner; transcranial doppler; SONORA Digital Bilateral Systems.

Candidate Terms

SONORA Digital Bilateral Systems [device term].

Device Index Terms

nuclear magnetic resonance scanner; transcranial doppler.

Other Index Terms

adult; aged; Article; *asymptomatic disease / *diagnosis; *carotid artery obstruction / *diagnosis; *carotid atherosclerosis / *diagnosis; clinical article; *cognition; controlled clinical trial; controlled study; female; human; male; mental deterioration; microembolism; middle cerebral artery occlusion / diagnosis; nuclear magnetic resonance imaging; patient identification; priority journal; transcranial Doppler ultrasonography; very elderly; white matter.

Link to the Ovid Full Text or citation:

Click here for full text options

Link to the External Link Resolver:

SFX

23.

For peer review only

Association between carotid artery intima-media thickness and combinations of mild cognitive impairment and pre-frailty in older adults.

Park J., Park J.-H., Park H.

International Journal of Environmental Research and Public Health. 16 (16) (no pagination), 2019. Article Number: 2978. Date of Publication: 02 Aug 2019.

AN: 2002471160

Carotid intima-media thickness (CIMT) has been proposed as a surrogate marker of cardiovascular disease. Mild cognitive impairment (MCI) and pre-frailty are reportedly associated with increased CIMT. As the evidence on the association of CIMT with combinations of MCI and pre-frailty is limited, this association is examined. A total of 231 older adults participated. MCI was defined according to clinical consensus or psychometric criteria by a dementia specialist, and considering detailed neuropsychological assessments. Also, pre-frailty was defined as subjects with frail component of 1 or 2. Carotid variables were measured using a B-mode ultrasound. The analysis of covariance (ANCOVA) was performed to assess independent differences in CIMT among the four groups, according to the cognitive function and frailty status after a multivariate adjustment. Increased CIMT is associated with combinations of MCI and pre-frailty. ANCOVA showed that CIMTs were significantly different among the four groups according to the cognitive function and frailty status. CIMTmax combined with MCI and pre-frailty was the thickest (1.04 +/- 0.3 mm), whereas the CIMT of no MCI and no pre-frailty was the thinnest (0.82 +/- 0.2 mm). The results suggest that combinations of MCI and pre-frailty are associated with increased CIMT in older adults.

Copyright © 2019 by the authors. Licensee MDPI, Basel, Switzerland.

PMID

31430926 [http://www.ncbi.nlm.nih.gov/pubmed/?term=31430926]

Institution

(Park) Department of Sport Rehabilitation, Dong-Ju College, Busan 49318, South Korea (Park) Health Convergence Medicine Research Group, Biomedical Research Institute, Pusan National University Hospital, 179, Gudeok-Ro, Seo-Gu, Busan 49241, South Korea

(Park) Department of Health Care Science, Dong-A University, Busan 49315, South Korea

(Park) Institute of Convergence Bio-Health, Dong-A University, Busan 49201, South Korea

Publisher

MDPI AG (Postfach, Basel CH-4005, Switzerland. E-mail: indexing@mdpi.com)

Emtree Heading

aged; *arterial wall thickness; article; B scan; body mass; body weight; cognition; controlled study; cross-sectional study; fat mass; female; *frailty; grip strength; human; major clinical study; male; *mild cognitive impairment; Mini Mental State Examination; physical activity; systolic blood pressure; waist circumference; walking distance; walking speed.

For peer review only - http://bmjopen.bmj.com/site/about/guidelines.xhtml

Other Index Terms

aged; *arterial wall thickness; Article; B scan; body mass; body weight; cognition; controlled study; cross-sectional study; fat mass; female; *frailty; grip strength; human; major clinical study; male; *mild cognitive impairment; Mini Mental State Examination; physical activity; systolic blood pressure; waist circumference; walking distance; walking speed.

Link to the Ovid Full Text or citation:

Click here for full text options

Link to the External Link Resolver:

SFX

24.

Carotid Atherosclerosis Predicts Lower Cognitive Test Results: A 7-Year Follow-Up Study of 4,371 Stroke-Free Subjects - The Tromso Study.

Arntzen K.A., Schirmer H., Johnsen S.H., Wilsgaard T., Mathiesen E.B.

Cerebrovascular Diseases. 33 (2) (pp 159-165), 2012. Date of Publication: 05 Jan 2012.

AN: 51802572

Background: Carotid artery atherosclerosis is a major risk factor for stroke and subsequent cognitive impairment. Prospective population studies have shown associations between carotid intima-media thickness (IMT) and stenosis and cognitive decline and dementia in elderly stroke-free persons, whereas results in the middle-aged are conflicting.

Method(s): In this prospective population-based study, 4,371 stroke-free middle-aged participants underwent carotid ultrasound examination and assessment of vascular risk factors at baseline and were tested for cognitive function 7 years later. Associations between IMT, number of plaques and total plaque area and cognitive test scores on verbal memory test, digit symbol-coding test and tapping test were assessed in linear regression models.

Result(s): In the multivariable analyses adjusted for sex, age, education, depression and vascular risk factors, the presence of plaques was significantly associated with lower test scores on the verbal memory test ($p = 0.01$) and on the digit symbol-coding test ($p = 0.03$). The number of plaques ($p = 0.01$) and the total plaque area ($p = 0.02$) were associated with lower scores on the

For peer review only - http://bmjopen.bmj.com/site/about/guidelines.xhtml

verbal memory test. No significant association was seen between common carotid artery IMT and cognitive test scores. The tapping test was not associated with the carotid ultrasound variables. Conclusion(s): In this middle-aged general population, subclinical carotid atherosclerosis measured as the presence of plaques, number of plaques and total plaque area were independent long-term predictors of lower cognitive test scores. Copyright © 2012 S. Karger AG, Basel.

Institution

(Arntzen) Department of Community Medicine, University of Tromso, Tromso, Norway

Publisher

S. Karger AG

Emtree Heading

*stroke; *carotid atherosclerosis; *follow up; middle aged; verbal memory; human; risk factor; ultrasound; population; carotid artery; cognition; linear regression analysis; model; education; common carotid artery; cognitive defect; population research; arterial wall thickness; stenosis; dementia; aged; examination.

Other Index Terms

*stroke; *carotid atherosclerosis; *follow up; middle aged; verbal memory; human; risk factor; ultrasound; population; carotid artery; cognition; linear regression analysis; model; education; common carotid artery; cognitive defect; population research; arterial wall thickness; stenosis; dementia; aged; examination.

Link to the Ovid Full Text or citation:

Click here for full text options

Link to the External Link Resolver:

SFX

25.

Effects of Carotid Endarterectomy on the Dynamics of Cognitive Impairments in Patients with Atherosclerotic Stenosis of the Carotid Arteries.

Yakhno N.N., Fedorova T.S., Damulin I.V., Shcherbyuk A.N., Vinogradov O.A., Lavrentiev A.V.

Neuroscience and Behavioral Physiology. (pp 1-7), 2012. Date of Publication: 2012.

AN: 52135697

For peer review only

The clinical and neurochemical characteristics of non-dementia cognitive disorders were studied in 102 patients with atherosclerotic carotid sclerosis, with assessment of their dynamics after carotid endarterectomy (CEAE). Mild cognitive disorders were seen in 37 patients (36.3 %) and moderate cognitive disorders in 36 patients (35.3 %). Moderate cognitive impairments were significantly more common in patients with symptoms of carotid stenosis, dominated by structural changes in the brain on neuroimaging (leukoaraiosis and infarcts); unstable atherosclerotic plaques, with a predominance of the hypodense component, were also more frequent. This suggests that cognitive dysfunction in patients with atherosclerotic carotid stenosis results not only from decreased perfusion, but also from arterio-arterial microembolism. CEAE was found to have favorable effects on cognitive functions. Positive changes were marked in patients with asymptomatic carotid stenosis. However, CEAE could also have adverse influences on cognitive functions in patients with moderate cognitive disorders of dysmnestic type and symptoms of carotid stenosis. © 2012 Springer Science+Business Media, Inc.

Institution

(Yakhno, Fedorova, Damulin) Department of Nervous Diseases, I. M. Sechenov First Moscow, State Medical University, Moscow, Russian Federation (Shcherbyuk, Vinogradov, Lavrentiev) Department of Vascular Surgery, University Clinical Hospital No. 1, I. M. Sechenov First Moscow State Medical University, Moscow, Russian Federation

Publisher

Springer US

Emtree Heading

*cognitive defect; *carotid endarterectomy; *human; *carotid artery obstruction; *dynamics; *patient; *carotid artery; *stenosis; *atherosclerosis; cognition; perfusion; atherosclerotic plaque; infarction; leukoaraiosis; neuroimaging; brain; sclerosis; dementia; commercial phenomena; microembolism.

Other Index Terms

*cognitive defect; *carotid endarterectomy; *human; *carotid artery obstruction; *dynamics; *patient; *carotid artery; *stenosis; *atherosclerosis; cognition; perfusion; atherosclerotic plaque; infarction; leukoaraiosis; neuroimaging; brain; sclerosis; dementia; commercial phenomena; microembolism.

Link to the Ovid Full Text or citation:

Click here for full text options

Link to the External Link Resolver:

SFX

For peer review only - http://bmjopen.bmj.com/site/about/guidelines.xhtml

27.

Atherosclerosis and dementia: A cross-sectional study with pathological analysis of the carotid arteries.

Suemoto C.K., Nitrini R., Grinberg L.T., Ferretti R.E.L., Farfel J.M., Leite R.E.P., Menezes P.R., Fregni F., Jacob-Filho W., Pasqualucci C.A.

Stroke A Journal of Cerebral Circulation. (no pagination), 2011. Date of Publication: 22 Sep 2011.

AN: 51631125

BACKGROUND AND PURPOSE: Previous ultrasound-based studies have shown an association between carotid artery atherosclerosis and dementia. Our aim was to investigate this association using postmortem examination.

METHOD(S): Postmortem morphometric measurements of carotid stenosis and intima-media thickness were performed in individuals with dementia (n=112) and control subjects (n=577). Multivariate logistic regression models were applied.

RESULT(S): High-grade left internal carotid stenosis (>=70%) was associated with increased odds for dementia (OR, 2.30; 95% CI, 1.14-4.74; P=0.02). Intima-media thickness was not associated with dementia.

CONCLUSION(S): The likelihood of dementia is increased with high-grade left internal carotid artery atherosclerosis after adjusting for demographic and cardiovascular risk factors.

Emtree Heading

*dementia; *carotid artery; *atherosclerosis; *cross-sectional study; carotid artery obstruction; arterial wall thickness; carotid atherosclerosis; cardiovascular risk; ultrasound; autopsy; logistic regression analysis; model; internal carotid artery.

Other Index Terms

*dementia; *carotid artery; *atherosclerosis; *cross-sectional study; carotid artery obstruction; arterial wall thickness; carotid atherosclerosis; cardiovascular risk; ultrasound; autopsy; logistic regression analysis; model; internal carotid artery.

Link to the Ovid Full Text or citation:

Click here for full text options

Link to the External Link Resolver:

For peer review only - http://bmjopen.bmj.com/site/about/guidelines.xhtml

SFX

31.

Carotid atherosclerosis and a reduced likelihood for lowered cognitive Performance in a Canadian first nations population.

Fergenbaum J.H., Bruce S., Spence J.D., Lou W., Hanley A.J.G., Greenwood C., Young T.K.

Neuroepidemiology. 33 (4) (pp 321-328), 2009. Date of Publication: December 2009.

AN: 50693794

Background: We investigated the associations among cardiovascular risk factors, carotid atherosclerosis and cognitive function in a Canadian First Nations population.

Method(s): Individuals aged >=18 years, without stroke, nonpreg- nant and with First Nations status were assessed by the Trail Making Test Parts A and B. Results were combined into a Trail Making Test executive function score (TMT-exec). Doppler ultrasonography assessed carotid stenosis and plaque volume. Anthropometric, vascular and metabolic risk factors were assessed by interview, clinical examinations and blood tests.

Result(s): For 190 individuals with TMT-exec scores, the median age of the population was 39 years. Compared to the reference group, individuals with elevated levels of left carotid stenosis (LCS) and total carotid stenosis (TCS) were less likely to demonstrate lowered cognitive performance [LCS, odds ratio (OR): 0.47, 95% confidence interval (CI): 0.24-0.96; TCS, OR: 0.40, 95% CI: 0.20-0.80]. No effect was shown for plaque volume. In structural equation modeling, we found that for every 1-unit change in the anthropometric factor in kg/m2, there was a 0.86-fold decrease in the percent of TCS ($p < 0.05$).

Conclusion(s): Individuals with elevated levels of LCS and TCS were less likely to demonstrate lowered performance. There was some suggestion that TCS mediates the effect of anthropometric risk factors on cognitive function. Copyright © 2009 S. Karger AG, Basel.

PMID

19887837 [http://www.ncbi.nlm.nih.gov/pubmed/?term=19887837]

Institution

(Fergenbaum, Lou, Hanley, Greenwood, Young) University of Toronto, Toronto, ON, Canada

(Bruce) University of Manitoba, Winnipeg, MB, Canada

(Spence) University of Western Ontario, London, ON, Canada

Publisher

S. Karger AG

For peer review only - http://bmjopen.bmj.com/site/about/guidelines.xhtml

Emtree Heading
adult; aged; anthropometric parameters; *atherosclerosis; blood examination; cardiovascular risk; carotid artery obstruction; *carotid atherosclerosis; clinical assessment; clinical examination; *cognition; conference paper; confidence interval; controlled study; cross-sectional study; Doppler echography; female; human; major clinical study; male; scoring system; structural equation modeling.
Other Index Terms
adult; aged; anthropometric parameters; *atherosclerosis; blood examination; cardiovascular risk; carotid artery obstruction; *carotid atherosclerosis; clinical assessment; clinical examination; *cognition; conference paper; confidence interval; controlled study; cross-sectional study; Doppler echography; female; human; major clinical study; male; scoring system; structural equation modeling.

Link to the Ovid Full Text or citation:
Click here for full text options

Link to the External Link Resolver:
SFX

32.

Carotid artery plaque detected on ultrasound is associated with impaired cognitive state in the elderly: A population-based study in Wakiso district, Uganda.
Mworozi K., Ameda F., Byanyima R.K., Nakasujja N.
Journal of Clinical Neuroscience. 68 (pp 194-200), 2019. Date of Publication: October 2019.
AN: 2002253229
Carotid artery disease which includes carotid artery stenosis, plaques, clots and increased intima media thickness, have been reported by many studies to be associated with dementia. Dementia is an end stage of usually asymptomatic cognitive impairment. Risk factors of carotid artery disease include; age, atherosclerosis, arteriosclerosis, shorter years in school, history of hypertension, diabetes mellitus, stroke and depression. This study set out to determine the prevalence of abnormal carotid ultrasound findings and their association with cognitive function among the adults >=60 years in Wakiso district, Uganda in 2018. A total of 210 participants were included.

For peer review only - http://bmjopen.bmj.com/site/about/guidelines.xhtml

Carotid artery stenosis, presence of plaque, stenosis and intima-media thickness were assessed by ultrasound. Cognitive status was assessed using a Mini Mental State Exam (MMSE) test. The prevalence of plaque was 21.4%. Variables which included; presence of plaque, age, education, gender, marital status, whether participant stayed alone or with someone else, care for self, occupation status, division of staying and history of smoking. The presence of plaque was associated with an abnormal cognitive function at both univariate and multivariate analysis with respective OR = 3.8 (95% CI = 1.90-7.54, p-value = 0.0001) and OR = 3.4 (95% CI = 1.38-8.15, p-value = 0.007). The cognitive function distribution was 43.8%, 19%, 34.3% and 2.9% within the normal, mild, moderate, and severe cognitive function status respectively. This study showed that prevalence of carotid artery plaque was high in this elderly population in Wakiso district Uganda. Also, carotid artery plaque was associated with abnormal cognitive function.

Copyright © 2019 Elsevier Ltd

PMID

31301929 [http://www.ncbi.nlm.nih.gov/pubmed/?term=31301929]

Institution

(Mworozi) Department of Radiology, School of Medicine, P.O. Box 7062, Kampala, Uganda

(Ameda) Department of Radiology, Makerere University College of Health Sciences, P.O. Box 7062, Kampala, Uganda

(Byanyima) Department of Radiology, Mulago National Referral Hospital, P.O. Box 7072, Kampala, Uganda

(Nakasujja) Department of Psychiatry, Makerere University College of Health Sciences, P.O. Box 7062, Kampala, Uganda

Publisher

Churchill Livingstone

Emtree Heading

age; aged; arterial wall thickness; article; *carotid artery disease/di [Diagnosis]; carotid artery obstruction/di [Diagnosis]; *cognitive defect; *disease association; *echography; education; female; gender; human; major clinical study; male; marriage; Mini Mental State Examination; occupation; population research; prevalence; priority journal; self care; smoking; Uganda; very elderly; *carotid artery plaque/di [Diagnosis].

Candidate Terms

*carotid artery plaque / *diagnosis [other term].

Other Index Terms

age; aged; arterial wall thickness; Article; *carotid artery disease / *diagnosis; carotid artery obstruction / diagnosis; *cognitive defect; *disease association; *echography; education; female; gender; human; major clinical study; male; marriage; Mini Mental State Examination; occupation; population research; prevalence; priority journal; self care; smoking; Uganda; very elderly.

For peer review only

Link to the Ovid Full Text or citation:

Click here for full text options

Link to the External Link Resolver:

SFX

For peer review only

33.

Association between carotid atheroma and cerebral cortex structure at age 73 years.
Alhusaini S., Karama S., Nguyen T.-V., Thiel A., Bernhardt B.C., Cox S.R., Corley J., Taylor A., Evans A.C., Star J.M., Bastin M.E., Wardlaw J.M., Deary I.J., Ducharme S.
Annals of Neurology. 84 (4) (pp 576-587), 2018. Date of Publication: October 2018.
AN: 624251907
Objective: To examine the relationship between carotid atherosclerosis and cerebral cortical thickness and investigate whether cortical thickness mediates the association between carotid atheroma and relative cognitive decline.
Method(s): We assessed 554 community-dwelling subjects (male/female: 296/258) from the Lothian Birth Cohort 1936 who underwent brain magnetic resonance imaging and carotid Doppler ultrasound studies at age 73 years. The relationship between carotid atherosclerosis markers (internal carotid artery stenosis, intima-media thickness, velocity, pulsatility, and resistivity indexes) and vertex-wide cerebral cortical thickness was examined cross-sectionally, controlling for gender, extensive vascular risk factors (VRFs), and intelligence quotient at age 11 (IQ-11). We also determined the association between carotid stenosis and a composite measure of fluid intelligence at age 73 years. A mediation model was applied to examine whether cortical thickness mediated the relationship between carotid stenosis and cognitive function.
Result(s): A widespread negative association was identified between carotid stenosis (median = 15%) and cerebral cortical thickness at age 73 years, independent of the side of carotid stenosis, other carotid measures, VRFs, and IQ-11. This association increased in an almost dose-response relationship from mild to severe degrees of carotid stenosis, across the anterior and posterior circulation territories. A negative association was also noted between carotid stenosis and fluid intelligence (standardized beta coefficient = -0.151, p = 0.001), which appeared partly (approximately 22%) mediated by carotid stenosis-related thinning of the cerebral cortex.

Interpretation(s): The findings suggest that carotid stenosis represents a marker of processes that accelerate aging of the cerebral cortex and cognition that is in part independent of measurable VRFs. Cortical thinning within the anterior and posterior circulation territories partially mediated the relationship between carotid atheroma and fluid intelligence. Ann Neurol 2018;84:576-587.
Copyright © 2018 The Authors. Annals of Neurology published by Wiley Periodicals, Inc. on behalf of American Neurological Association.
PMID
30179274 [http://www.ncbi.nlm.nih.gov/pubmed/?term=30179274]
Author NameID
Wardlaw, Joanna M.; ORCID: http://orcid.org/0000-0002-9812-6642
Institution
(Alhusaini, Thiel, Ducharme) Department of Neurology and Neurosurgery, Montreal Neurological Institute and Hospital, McGill University, Montreal, QC, Canada (Karama, Bernhardt, Evans, Ducharme) McConnell Brain Imaging Centre, Montreal Neurological Institute, McGill University, Montreal, QC, Canada
(Karama) Department of Psychiatry, Douglas Mental Health University Institute, McGill University, Montreal, QC, Canada
(Nguyen, Ducharme) Department of Psychiatry, McGill University Health Centre, McGill University, Montreal, QC, Canada
(Nguyen) Department of Obstetrics-Gynecology, McGill University Health Centre, McGill University, Montreal, QC, Canada
(Thiel) Department of Neurology, Jewish General Hospital, Lady Davis Institute for Medical Research, Montreal, QC, Canada
(Cox, Corley, Taylor, Star, Bastin, Wardlaw, Deary) Centre for Cognitive Ageing and Cognitive Epidemiology, Department of Psychology, University of Edinburgh, Edinburgh, United Kingdom
(Star, Deary) Alzheimer Scotland Dementia Research Centre, Department of Psychology, University of Edinburgh, Edinburgh, United Kingdom
(Bastin, Wardlaw) Brain Research Imaging Centre, Centre for Clinical Brain Sciences, University of Edinburgh, Edinburgh, United Kingdom
(Wardlaw) UK Dementia Research Institute at the University of Edinburgh, Edinburgh, United Kingdom
Publisher
John Wiley and Sons Inc. (P.O.Box 18667, Newark NJ 07191-8667, United States)
Emtree Heading
aged; arterial wall thickness; article; brain circulation; cardiovascular risk; carotid artery obstruction; *carotid atherosclerosis; clinical assessment; cognition; *cognitive defect; cohort analysis; *cortical thickness (brain); disease association; disease severity; Doppler ultrasonography; female; human;

For peer review only

intelligence quotient; major clinical study; male; neuroimaging; nuclear magnetic resonance imaging; patient risk; priority journal; pulsatility index; risk assessment; risk factor; *carotid atheroma.

Candidate Terms

*carotid atheroma [other term].

Other Index Terms

aged; arterial wall thickness; Article; brain circulation; cardiovascular risk; carotid artery obstruction; *carotid atherosclerosis; clinical assessment; cognition; *cognitive defect; cohort analysis; *cortical thickness (brain); disease association; disease severity; Doppler ultrasonography; female; human; intelligence quotient; major clinical study; male; neuroimaging; nuclear magnetic resonance imaging; patient risk; priority journal; pulsatility index; risk assessment; risk factor.

Link to the Ovid Full Text or citation:

Click here for full text options

Link to the External Link Resolver:

SFX

37.

Carotid circumferential wall stress is not associated with cognitive performance among individuals in late middle age: The Maastricht Study.

Geijselaers S.L., Sep S.J., Schram M.T., van Boxtel M.P., van Sloten T.T., op het Roodt J., Henry R.M., Reesink K.D., Schaper N.C., Dagnelie P.C., van der Kallen C.J., Biessels G.J., Stehouwer C.D.

Atherosclerosis. 276 (pp 15-22), 2018. Date of Publication: September 2018.

AN: 2000935317

Background and aims: Arterial remodelling aims at normalising circumferential wall stress (CWS). Greater CWS in the carotid artery has previously been associated with the prevalence and severity of cerebral small vessel disease, a major cause of ageing-related cognitive decline. Here we test the hypothesis that greater carotid CWS is associated with poorer cognitive performance.

Method(s): We studied 722 individuals (60 +/- 8 years, 55% men, 42.5% highly educated, blood pressure 137 +/- 19/77 +/- 11 mmHg, n = 197 with type 2 diabetes) who completed a neuropsychological assessment and underwent vascular ultrasound to measure the intima-media

For peer review only - http://bmjopen.bmj.com/site/about/guidelines.xhtml

thickness (IMT) and interadventitial diameter (IAD) of the left common carotid artery at a plaque-free site. From IMT and IAD, lumen diameter (LD) was calculated. These structural measures were then combined with local carotid pulse pressure and brachial mean arterial pressure to obtain a measure of pulsatile (CWSpulsatile) and average (CWSmean) mechanical load on the vessel wall. Cognitive domains assessed were memory, executive function and attention, and processing speed.
Result(s): After adjustment for age, sex, and education, regression analyses showed that neither CWSpulsatile nor CWSmean were associated with measures of cognitive performance (p-values >=0.31). This null association did not differ by age or educational level, and was observed in both individuals with and without carotid plaque, diabetes and/or hypertension. In addition, none of the individual measures of carotid structure (i.e. IMT, IAD, and LD) was related to cognitive performance.
Conclusion(s): The present cross-sectional study shows that carotid CWS is not associated with cognitive performance, at least not among relatively highly educated individuals in late middle age with adequately controlled cardiovascular risk factors.
Copyright © 2018 The Authors
PMID
30006323 [http://www.ncbi.nlm.nih.gov/pubmed/?term=30006323]
Author NameID
op het Roodt, Jos; ORCID: http://orcid.org/0000-0003-3535-6894 Schaper, Nicolaas C.; ORCID: http://orcid.org/0000-0002-2128-8029
Institution
(Geijselaers, Sep, Schram, van Sloten, op het Roodt, Henry, Schaper, van der Kallen, Stehouwer) Department of Internal Medicine, Maastricht University Medical Centre +, Maastricht, Netherlands
(Geijselaers, Sep, Schram, van Sloten, op het Roodt, Henry, Schaper, Dagnelie, van der Kallen, Stehouwer) CARIM School for Cardiovascular Diseases, Maastricht University, Maastricht, Netherlands
(Geijselaers, Biessels) Department of Neurology, Brain Centre Rudolf Magnus, University Medical Centre Utrecht, Utrecht, Netherlands
(van Boxtel) Department of Psychiatry and Neuropsychology and MHeNS School for Mental Health and Neuroscience, Maastricht University Medical Centre +, Maastricht, Netherlands
(Reesink) Department of Biomedical Engineering, Maastricht University Medical Centre +, Maastricht, Netherlands
(Schaper, Dagnelie) CAPHRI School for Public Health and Primary Care, Maastricht University, Maastricht, Netherlands
(Dagnelie) Department of Epidemiology, Maastricht University, Maastricht, Netherlands
Publisher

Elsevier Ireland Ltd

Emtree Heading

adult; aged; arterial wall thickness; article; atherosclerotic plaque; attention; brachial artery; cardiovascular disease/dt [Drug Therapy]; *carotid artery; carotid artery pulse; *carotid atherosclerosis/di [Diagnosis]; *carotid atherosclerosis/dt [Drug Therapy]; *cognition; *cognitive defect; controlled study; cross-sectional study; depression; diabetic patient; echography; educational status; executive function; female; human; hypertension/dt [Drug Therapy]; information processing; left common carotid artery; major clinical study; male; mean arterial pressure; memory; middle aged; neuropsychological test; non insulin dependent diabetes mellitus; null hypothesis; priority journal; pulse pressure; regression analysis; *wall stress; antihypertensive agent/dt [Drug Therapy]; antithrombocytic agent/dt [Drug Therapy]; beta adrenergic receptor blocking agent/dt [Drug Therapy]; calcium antagonist/dt [Drug Therapy]; diuretic agent/dt [Drug Therapy]; renin inhibitor/dt [Drug Therapy]; *carotid circumferential wall stress.

Candidate Terms

*carotid circumferential wall stress [other term].

Drug Index Terms

antihypertensive agent / drug therapy; antithrombocytic agent / drug therapy; beta adrenergic receptor blocking agent / drug therapy; calcium antagonist / drug therapy; diuretic agent / drug therapy; renin inhibitor / drug therapy.

Other Index Terms

adult; aged; arterial wall thickness; Article; atherosclerotic plaque; attention; brachial artery; cardiovascular disease / drug therapy; *carotid artery; carotid artery pulse; *carotid atherosclerosis / *diagnosis / *drug therapy; *cognition; *cognitive defect; controlled study; cross-sectional study; depression; diabetic patient; echography; educational status; executive function; female; human; hypertension / drug therapy; information processing; left common carotid artery; major clinical study; male; mean arterial pressure; memory; middle aged; neuropsychological test; non insulin dependent diabetes mellitus; null hypothesis; priority journal; pulse pressure; regression analysis; *wall stress.

Link to the Ovid Full Text or citation:

Click here for full text options

Link to the External Link Resolver:

SFX

For peer review only - http://bmjopen.bmj.com/site/about/guidelines.xhtml

40.

The preservation of cognition 1 year after carotid endarterectomy in patients with prior cognitive decline.

Dempsey R.J., Jackson D.C., Wilbrand S.M., Mitchell C.C., Berman S.E., Johnson S.C., Meshram N.H., Varghese T., Hermann B.P.

Neurosurgery. 82 (3) (pp 322-328), 2018. Date of Publication: 01 Mar 2018.

AN: 623354114

BACKGROUND: Vascular cognitive decline is critically important in the course of atherosclerosis and stroke.

OBJECTIVE(S): To explore the hypothesis that carotid endarterectomy (CEA) by removing an unstable plaque may slow the course of vascular cognitive decline in both symptomatic and asymptomatic patients.

METHOD(S): Patients with clinically significant (>60%) carotid stenosis were studied preop and 1 yr post-CEA for clinical symptoms, vascular cognitive decline, instability of carotid plaque-presence of microemboli, brain white matter changes, and medical risk factors.

RESULT(S): Forty-six percent were classically symptomatic. All patients showed vascular cognitive decline at presentation which correlatedwith degree of plaque instability. Significant white matter hyperintensity changes (48.7%) and cerebral emboli (25%) were also seen at baseline in both classically symptomatic and asymptomatic. One year after CEA, both groups showed no decline in cognitive function and significant improvement in 2 tests (P = .028 and P = .013). Brain white matter hyperintensities were unchanged. Microemboliwere reduced but remained present (17.86%). Improvementwas predicted by the presence of hypertension (P = .001), or less advanced cognitive decline preoperatively (P = .009).

CONCLUSION(S): This study demonstrates the importance of vascular cognitive decline in atherosclerotic disease. This is a function of the degree of instability of the atherosclerotic plaque more than the presence of stroke symptoms. It further suggests that atherosclerotic vascular cognitive decline need not be inevitable, and may be modified by treating hypertension and removal of the unstable plaque. This highlights the need for continued research on the cognitive effects of cerebrovascular disease and the synergistic benefits of intensive medical and surgical therapy.

Copyright © 2017 by the Congress of Neurological Surgeons.

Institution

(Dempsey, Wilbrand) Department of Neurological Surgery, University of Wisconsin, School of Medicine and Public Health, 600 Highland Avenue, Madison, WI 53792, United States (Jackson)

For peer review only - http://bmjopen.bmj.com/site/about/guidelines.xhtml

Wisconsin Surgical Outcomes, Center Research Program, Department of Surgery, University of Wisconsin, School of Medicine and Public Health, Madison, WI, United States
(Mitchell) Department of Medicine, Cardiovascular Medicine Division, University of Wisconsin, School of Medicine and Public Health, Madison, WI, United States
(Berman, Johnson) Alzheimer's Disease Research Center, Uiversity of Wisconsin, School of Medicine and Public Health, Madison, WI, United States
(Meshram, Varghese) Department of Medical Physics, University of Wisconsin, School of Medicine and Public Health, Madison, WI, United States
(Meshram) Department of Electrical and Computer Engineering, University of Wisconsin-Madison, Madison, WI, United States
(Hermann) Department of Neurology, University of Wisconsin, School of Medicine and Public Health, Centennial Building, Madison, WI, United States
Publisher
Oxford University Press
Emtree Heading
aged; article; asymptomatic disease; brain embolism; carotid artery obstruction/su [Surgery]; carotid atherosclerosis; *carotid endarterectomy; cerebrovascular accident; cerebrovascular disease; clinical article; clinical outcome; *cognition; *cognitive defect; cohort analysis; diabetes mellitus; disease association; disease course; exploratory research; female; human; hyperlipidemia; hypertension; hypothesis; male; microembolism; preoperative evaluation; priority journal; risk factor; smoking; transient ischemic attack; white matter; white matter lesion.
Other Index Terms
aged; Article; asymptomatic disease; brain embolism; carotid artery obstruction / surgery; carotid atherosclerosis; *carotid endarterectomy; cerebrovascular accident; cerebrovascular disease; clinical article; clinical outcome; *cognition; *cognitive defect; cohort analysis; diabetes mellitus; disease association; disease course; exploratory research; female; human; hyperlipidemia; hypertension; hypothesis; male; microembolism; preoperative evaluation; priority journal; risk factor; smoking; transient ischemic attack; white matter; white matter lesion.

Link to the Ovid Full Text or citation:
Click here for full text options

Link to the External Link Resolver:
SFX

For peer review only

41.

Response to "carotid flow velocities and endothelial function in cognitive ability of hypertension".

Chuang S.-Y., Cheng H.-M.

American Journal of Hypertension. 32 (6) (pp E9), 2019. Date of Publication: 2019.

AN: 628237346

PMID

30984973 [http://www.ncbi.nlm.nih.gov/pubmed/?term=30984973]

Institution

(Chuang) Institute of Population Health Science, National Health Research Institutes, Miaoli, Taiwan (Republic of China) (Cheng) Department of Medicine, National Yang-Ming University, Taipei, Taiwan (Republic of China)

(Cheng) Institute of Public Health and Community, Medicine Research Center, National Yang-Ming University, Taipei, Taiwan (Republic of China)

(Cheng) Center for Evidence-based Medicine, Taipei Veterans General Hospital, Taipei, Taiwan (Republic of China)

Publisher

Oxford University Press

Emtree Heading

arterial wall thickness; atherosclerosis; *blood flow velocity; blood vessel tone; brain tissue; cardiovascular risk; carotid artery; *carotid artery flow; *cognition; cognitive defect; diastolic blood pressure; disease association; *endothelial dysfunction; *endothelium; heart tissue; hemodynamic parameters; human; *hypertension; letter; Mini Mental State Examination; peak systolic velocity; priority journal; risk assessment; risk factor; systolic blood pressure; vascular endothelium; white matter.

Other Index Terms

arterial wall thickness; atherosclerosis; *blood flow velocity; blood vessel tone; brain tissue; cardiovascular risk; carotid artery; *carotid artery flow; *cognition; cognitive defect; diastolic blood pressure; disease association; *endothelial dysfunction; *endothelium; heart tissue; hemodynamic parameters; human; *hypertension; Letter; Mini Mental State Examination; peak systolic velocity; priority journal; risk assessment; risk factor; systolic blood pressure; vascular endothelium; white matter.

Link to the Ovid Full Text or citation:

For peer review only

Click here for full text options

Link to the External Link Resolver:
SFX

42.

Higher arterial stiffness is associated with lower cognitive performance in patients with hypertension.
Muela H.C.S., Costa-Hong V.A., Yassuda M.S., Moraes N.C., Memoria C.M., Machado M.F., Bor-Seng-Shu E., Nogueira R.C., Mansur A.J., Massaro A.R., Nitrini R., Macedo T.A., Bortolotto L.A.
Journal of Clinical Hypertension. 20 (1) (pp 22-30), 2018. Date of Publication: January 2018.
AN: 619150646
Cognitive impairment and elevated arterial stiffness have been described in patients with arterial hypertension, but their association has not been well studied. We evaluated the correlation of arterial stiffness and different cognitive domains in patients with hypertension compared with those with normotension. We evaluated 211 patients (69 with normotension and 142 with hypertension). Patients were age matched and distributed according to their blood pressure: normotension, hypertension stage 1, and hypertension stage 2. Cognitive function was assessed using the Mini-Mental State Examination, Montreal Cognitive Assessment, and a battery of neuropsychological evaluations that assessed six main cognitive domains. Pulse wave velocity was measured using a Complior device, and carotid properties were assessed by radiofrequency ultrasound. Central arterial pressure and augmentation index were obtained using applanation tonometry. The hypertension stage 2 group had higher arterial stiffness and worse performance either by Mini-Mental State Examination (26.8+/-2.1 vs 27.3+/-2.1 vs 28.0+/-2.0, P=.003) or the Montreal Cognitive Assessment test (23.4+/-3.5 vs 24.9+/-2.9 vs 25.6+/-3.0, P<.001). On multivariable regression analysis, augmentation index, intima-media thickness, and pulse wave velocity were the variables mainly associated with lower cognitive performance at different cognitive domains. Cognitive impairment in different domains was associated with higher arterial stiffness.
Copyright ©2017 Wiley Periodicals, Inc.
PMID
29106057 [http://www.ncbi.nlm.nih.gov/pubmed/?term=29106057]
Author NameID

For peer review only

Muela, Henrique C.S.; ORCID: http://orcid.org/0000-0002-0071-9555
Institution
(Muela, Costa-Hong, Mansur, Macedo, Bortolotto) Hypertension Unit, Heart Institute (InCor), University of Sao Paulo Medical School, Sao Paulo, Brazil (Muela) Department of Physiology, Faculty of Medicine, Agostinho Neto University, Luanda, Angola
(Yassuda, Moraes, Memoria, Machado, Bor-Seng-Shu, Nogueira, Massaro, Nitrini) Department of Neurology, University of Sao Paulo Medical School, Sao Paulo, Brazil
Publisher
Blackwell Publishing Inc. (E-mail: subscrip@blackwellpub.com)
Emtree Heading
adult; age; arterial pressure; *arterial stiffness; arterial wall thickness; article; augmentation index; blood pressure; carotid artery; *cognition; cognitive defect/di [Diagnosis]; controlled study; cross-sectional study; *disease association; disease severity; echography; female; human; *hypertension/di [Diagnosis]; major clinical study; male; middle aged; Mini Mental State Examination; Montreal cognitive assessment; neuropsychological test; priority journal; pulse wave; radiofrequency; regression analysis; tonometry; cardiovascular equipment; applanation tonometry; Complior device.
Candidate Terms
applanation tonometry [other term]; Complior device [other term].
Device Index Terms
cardiovascular equipment.
Other Index Terms
adult; age; arterial pressure; *arterial stiffness; arterial wall thickness; Article; augmentation index; blood pressure; carotid artery; *cognition; cognitive defect / diagnosis; controlled study; cross-sectional study; *disease association; disease severity; echography; female; human; *hypertension / *diagnosis; major clinical study; male; middle aged; Mini Mental State Examination; Montreal cognitive assessment; neuropsychological test; priority journal; pulse wave; radiofrequency; regression analysis; tonometry.

Link to the Ovid Full Text or citation:
Click here for full text options

Link to the External Link Resolver:
SFX

For peer review only

44.

Pulse wave velocity is associated with greater risk of dementia in mild cognitive impairment patients.

Rouch L., Cestac P., Sallerin B., Andrieu S., Bailly H., Beunardeau M., Cohen A., Dubail D., Hernandorena I., Seux M.-L., Vidal J.-S., Hanon O.

Hypertension. 72 (5) (pp 1109-1116), 2018. Date of Publication: 2018.

AN: 627080314

To investigate the association between pulse wave velocity, intima-media thickness, carotid artery diameter, carotid plaques, and conversion from mild cognitive impairment to dementia. Three hundred and seventy-five elderly ambulatory subjects with mild cognitive impairment were followed yearly to examine potential conversion to dementia. Vascular function was assessed by carotid-femoral pulse wave velocity. Vascular structure was evaluated by intima-media thickness, carotid artery diameter, and carotid plaques using an ultrasonographic assessment of carotid arteries. One hundred and five patients (28%) converted to dementia during a mean follow-up period of 4.5 years. Higher pulse wave velocity was associated with greater risk of conversion to dementia (1-SD increase of pulse wave velocity: Hazard ratio, 1.33; 95% CI, 1.04-1.71; P=0.02) independently of age, sex, educational level, systolic blood pressure, cardiovascular diseases, body mass index, calcium channel blockers intake, Mini-Mental State Examination at baseline, and apoE a4 status. Intima-media thickness, carotid plaques, and carotid artery diameter did not predict conversion to dementia (1-SD increase of intima-media thickness: Hazard ratio, 0.93; 95% CI, 0.73-1.18; P=0.55; presence of carotid plaques: Hazard ratio, 1.08; 95% CI, 0.62-1.87; P=0.79; 1-SD increase of carotid artery diameter: Hazard ratio, 1.08; 95% CI, 0.89-1.31; P=0.44). Pulse wave velocity was associated with conversion to dementia, whereas intima-media thickness, carotid plaques, or carotid artery diameter were not after controlling for age and other confounding factors. Arterial stiffness could identify mild cognitive impairment patients at higher risk of dementia and may be a therapeutic target to delay or prevent the onset of dementia.

Copyright © 2018 American Heart Association, Inc.

PMID

30354804 [http://www.ncbi.nlm.nih.gov/pubmed/?term=30354804]

Institution

(Rouch, Bailly, Beunardeau, Cohen, Dubail, Hernandorena, Seux, Vidal, Hanon) Universite Paris Descartes, Sorbonne Paris Cite, France (Cestac, Sallerin, Andrieu) Unite INSERM, Toulouse 1027, France

(Cestac, Sallerin, Andrieu) University Paul Sabatier Toulouse, France

For peer review only - http://bmjopen.bmj.com/site/about/guidelines.xhtml

(Cestac, Sallerin, Cohen) Centre Hospitalier Universitaire de, Toulouse, France
(Bailly, Beunardeau, Cohen, Dubail, Hernandorena, Seux, Vidal, Hanon) Hopital Broca, Hopitaux Universitaires, Paris, France

Publisher

Lippincott Williams and Wilkins (E-mail: kathiest.clai@apta.org)

Emtree Heading

adult; aged; arterial stiffness; arterial wall thickness; artery diameter; article; body mass; cardiovascular disease; carotid artery; *dementia; diastolic blood pressure; *disease association; echography; educational status; female; follow up; human; major clinical study; male; *mild cognitive impairment; Mini Mental State Examination; multiinfarct dementia; priority journal; *pulse wave; *risk factor; systolic blood pressure; angiotensin receptor antagonist; antihypertensive agent; apolipoprotein E4/ec [Endogenous Compound]; beta adrenergic receptor blocking agent; calcium channel blocking agent; dipeptidyl carboxypeptidase inhibitor; diuretic agent.

Drug Index Terms

angiotensin receptor antagonist; antihypertensive agent; apolipoprotein E4 / endogenous compound; beta adrenergic receptor blocking agent; calcium channel blocking agent; dipeptidyl carboxypeptidase inhibitor; diuretic agent.

Other Index Terms

adult; aged; arterial stiffness; arterial wall thickness; artery diameter; Article; body mass; cardiovascular disease; carotid artery; *dementia; diastolic blood pressure; *disease association; echography; educational status; female; follow up; human; major clinical study; male; *mild cognitive impairment; Mini Mental State Examination; multiinfarct dementia; priority journal; *pulse wave; *risk factor; systolic blood pressure.

Link to the Ovid Full Text or citation:

Click here for full text options

Link to the External Link Resolver:

SFX

45.

Vascular, cognitive, and psychomental survey on elderly recycling volunteers in northern Taiwan.

For peer review only

Chen G.-C., Chen P.-Y., Su Y.-C., Hsiao C.-L., Yang F.-Y., Hsu P.-J., Lin S.-K.

Frontiers in Neurology. 10 (JAN) (no pagination), 2019. Article Number: 1176. Date of Publication: 2019.

AN: 627647510

Background: Stroke and dementia represent frequent causes of psychophysical and socioeconomic burdens. We conducted a vascular, cognitive, and psychomental survey involving elderly volunteers at community-based recycling stations in Northern Taiwan.

Method(s): Recycling volunteers aged >=60 years were surveyed. We recorded seven parameters, namely (1) body mass index (BMI), (2) fasting glucose, (3) fasting cholesterol, (4) ankle-brachial index (ABI), (5) carotid duplex sonography, (6) five-item Brief Symptom Rating Scale (BSRS-5) score, and (7) eight-item Interview to Differentiate Aging and Dementia (AD8). During the carotid duplex study, we measured the carotid intima-media thickness (CIMT) and the carotid total plaque score (CTPS) of the common and internal carotid arteries.

Result(s): In total, 985 subjects (mean age: 70.8 years) participated in this study. Among these, 81% were women, and 52% were vegetarians. The average ABI, CIMT, and CTPS were higher in men, whereas women had higher cholesterol levels and BSRS-5 scores. Obesity, hypertension, hyperglycemia, and hyperlipidemia were present in 21, 38, 9, and 27% of all subjects, respectively. Carotid plaques with mild (CTPS 1-5), moderate (CTPS 5.1-10), and severe (CTPS > 10) atherosclerosis were detected in 45, 16, and 7% of the subjects, respectively. Mild cognitive impairment (AD8 > 2) was observed in 13% of the subjects, whereas moderate mood disorder (BSRS-510) was observed in only 1% of subjects. Vegetarians had a lower BMI, systolic blood pressure (SBP), cholesterol, CIMT, and CTPS than did non-vegetarians. Substantial predictors of severe atherosclerosis were advanced age (>70 years), male sex, history of heart disease, hyperlipidemia, and currently elevated SBP and cholesterol levels. Predictors of mild cognitive impairment were illiteracy, history of hypertension, hyperlipidemia, and moderate mood disorder.

Conclusion(s): Subclinical carotid atherosclerosis was common in elderly recycling volunteers, with 23% having moderate to severe stenosis. Vegetarians had a reduced risk of atherosclerosis. The low incidence of moderate mood disorder might indicate that recycling work enhances psychomental health. In addition, a healthier lifestyle, better mood condition, and vegetarian diet might contribute to lower incidence of mild cognitive impairment.

Copyright © 2019 Chen, Chen, Su, Hsiao, Yang, Hsu and Lin.

Institution

(Chen, Chen, Su, Hsiao, Yang, Hsu, Lin) Stroke Center and Department of Neurology, Taipei Tzu Chi Hospital, Buddhist Tzu Chi Medical Foundation, New Taipei City, Taiwan (Republic of China)

(Su, Lin) School of Medicine, Tzu Chi University, Hualien, Taiwan (Republic of China)

Publisher

Frontiers Media S.A. (E-mail: info@frontiersin.org)

For peer review only - http://bmjopen.bmj.com/site/about/guidelines.xhtml

Emtree Heading
age; aged; ankle brachial index; arterial wall thickness; article; body mass; carotid atherosclerosis; cholesterol blood level; *cognition; common carotid artery; disease severity; echography; female; gender; glucose blood level; human; hyperglycemia; hyperlipidemia; hypertension; internal carotid artery; major clinical study; male; mild cognitive impairment; mood disorder; obesity; prospective study; rating scale; systolic blood pressure; Taiwan; vegetarian; *volunteer; cholesterol/ec [Endogenous Compound]; glucose/ec [Endogenous Compound]; Brief Symptom Rating Scale; carotid duplex sonography; Differentiate Aging and Dementia Scale; *recycling volunteer.
Candidate Terms
Brief Symptom Rating Scale [other term]; carotid duplex sonography [other term]; Differentiate Aging and Dementia Scale [other term]; *recycling volunteer [other term].
Drug Index Terms
cholesterol / endogenous compound; glucose / endogenous compound.
Other Index Terms
age; aged; ankle brachial index; arterial wall thickness; Article; body mass; carotid atherosclerosis; cholesterol blood level; *cognition; common carotid artery; disease severity; echography; female; gender; glucose blood level; human; hyperglycemia; hyperlipidemia; hypertension; internal carotid artery; major clinical study; male; mild cognitive impairment; mood disorder; obesity; prospective study; rating scale; systolic blood pressure; Taiwan; vegetarian; *volunteer.

Link to the Ovid Full Text or citation:
Click here for full text options

Link to the External Link Resolver:
SFX

47.

Common Carotid Artery Calcification Impacts on Cognitive Function in Older Patients.
Di Daniele N., Celotto R., Alunni Fegatelli D., Gabriele M., Rovella V., Scuteri A.
High Blood Pressure and Cardiovascular Prevention. 26 (2) (pp 127-134), 2019. Date of Publication: 01 Apr 2019.
AN: 627071939

For peer review only

Introduction: Cognitive impairment and dementia represent an emerging health problem. Cardiovascular (CV) risk factors contribute to cognitive impairment.
Aim(s): To investigate the effect of vascular calcification on cognitive impairment and dementia, independently of plaque and traditional CV risk factors.
Method(s): Four hundred and sixty-nine patients (age of 78.6 +/- 6.1 years, 74.4% women) were studied. Traditional CV risk factors levels, cognitive function (MMSE), brain CT scan, and other vascular parameters were measured. Common Carotid Artery (CCA) plaque and calcification were evaluated by ultrasound.
Result(s): CCA calcification was associated with a lower MMSE score than in subjects with no CCA calcification (23.7 +/- 0.3 versus 25.5 +/- 0.8; p = 0.015), after controlling for age, sex, education, blood pressure levels, diabetes, creatinine, lipid lowering therapy, neuroimaging alteration, and CCA plaque. Similarly, CCA calcification was associated with higher odds of dementia regardless of the presence of CCA plaque (OR 1.70, 95% CI 1.01-2.94, p < 0.05). This trend was not observed when stratifying patients according to the presence of CCA plaque.
Conclusion(s): CCA calcification is associated with cognitive impairment and dementia, independently of established CV risk factors and CCA plaque. The impact of arterial calcification on cognition seems largely independent of arterial stiffness.
Copyright © 2019, Italian Society of Hypertension.
PMID
30779026 [http://www.ncbi.nlm.nih.gov/pubmed/?term=30779026]
Institution
(Di Daniele, Celotto, Gabriele, Rovella) Hypertension and Nephrology Unit, Department of Medicine, Policinico Tor Vergata, Universita'di Roma Tor Vergata, Rome, Italy (Alunni Fegatelli) Department of Public Health and Infectious Disease, University "La Sapienza", Rome, Italy (Scuteri) Department of Medical, Surgical, and Experimental Sciences, University of Sassari, Sassari, Italy
Publisher
Springer International Publishing
Emtree Heading
aged; albumin blood level; arterial stiffness; *artery calcification; article; blood pressure; cholesterol blood level; *cognition; *cognitive defect; *common carotid artery; creatinine blood level; dementia; diabetes mellitus; education; female; human; hypotension; major clinical study; male; Mini Mental State Examination; neuroimaging; priority journal; pulse wave; white matter lesion; x ray computed tomography; albumin/ec [Endogenous Compound]; antidiabetic agent/pv [Special Situation for Pharmacovigilance]; antihypertensive agent/pv [Special Situation for Pharmacovigilance]; antilipemic agent; antithrombocytic agent/pv [Special Situation for Pharmacovigilance]; creatinine/ec [Endogenous Compound]; high density lipoprotein

For peer review only - http://bmjopen.bmj.com/site/about/guidelines.xhtml

cholesterol/ec [Endogenous Compound]; hydroxymethylglutaryl coenzyme A reductase inhibitor/pv [Special Situation for Pharmacovigilance]; low density lipoprotein cholesterol/ec [Endogenous Compound]; nitric acid derivative/pv [Special Situation for Pharmacovigilance]; triacylglycerol/ec [Endogenous Compound].

Drug Index Terms

albumin / endogenous compound; antidiabetic agent / special situation for pharmacovigilance; antihypertensive agent / special situation for pharmacovigilance; antilipemic agent; antithrombocytic agent / special situation for pharmacovigilance; creatinine / endogenous compound; high density lipoprotein cholesterol / endogenous compound; hydroxymethylglutaryl coenzyme A reductase inhibitor / special situation for pharmacovigilance; low density lipoprotein cholesterol / endogenous compound; nitric acid derivative / special situation for pharmacovigilance; triacylglycerol / endogenous compound.

Other Index Terms

aged; albumin blood level; arterial stiffness; *artery calcification; Article; blood pressure; cholesterol blood level; *cognition; *cognitive defect; *common carotid artery; creatinine blood level; dementia; diabetes mellitus; education; female; human; hypotension; major clinical study; male; Mini Mental State Examination; neuroimaging; priority journal; pulse wave; white matter lesion; x-ray computed tomography.

Link to the Ovid Full Text or citation:

Click here for full text options

Link to the External Link Resolver:

SFX

51.

Study on the correlation between vascular lesions and cognitive function in native tibetan with h-type hypertension in plateau.

Li Y., Wu S.

High Altitude Medicine and Biology. Conference: 12th World Congress on Mountain Medicine. Nepal. 19 (4) (pp A451), 2018. Date of Publication: December 2018.

AN: 627024764

For peer review only - http://bmjopen.bmj.com/site/about/guidelines.xhtml

Objective: To explore characteristics of vascular lesions and the correlation between vascular lesions and cognitive function in native Tibetan patients with H-type hypertension in Qinghai-Tibetan Plateau of China.
Method(s): Sixty-two cases with H-type hypertension and 178 cases with non-H-type hypertension were enrolled in native Tibetan patients at high altitude (Yushu city, Qinghai Province, China; 3800 m). All the subjects detected carotid artery atheroselerotic plaques and carotid intima-media thickness (CIMT) with the color Doppler ultrasound, examined the cognitive function with the minimental state examination (MMSE), and measured the brachial-ankle pulse wave velocity (BaPWV), and ankle brachial index (ABI) with the Colin-VP1000 type artery stiff diagnosis apparatus.
Result(s): Comparing with non-H-type hypertension, the incidence of carotid artery atheroselerotic plaques, CIMT, and BaPWV was significantly higher in the native Tibetan with H-type hypertension ($p < 0.05$), whereas MMSE and ABI were significantly decreased ($p < 0.05$). MMSE were positively correlated with BaPWV, CIMT, and plaques, MMSE was negatively correlated with ABI and plaques.
Conclusion(s): The patients with H-type hypertension are more likely to have the peripheral artery structure and function impairments and more severe the cognition impaired, which closely correlate with high altitude and hypoxia. Hypoxia and special national diet (less vegetables and more meat) affect the metabolism of homocysteine. Elevated plasma Hcy level may aggravate vascular lesions and cognitive impairment in native Tibetan patients with high blood pressure in the plateau.
Institution
(Li, Wu) Qinghai Provincial People's Hospital, Xining, China
Publisher
Mary Ann Liebert Inc.
Emtree Heading
adult; *altitude disease; ankle brachial index; arterial wall thickness; carotid atherosclerosis; China; *cognitive defect; color Doppler flowmetry; controlled study; diet; female; human; human tissue; *hypertension; incidence; major clinical study; male; metabolism; plasma; pulse wave; structure activity relation; *vascular lesion; vegetable; homocysteine; conference abstract.
Candidate Terms
conference abstract [other term].
Drug Index Terms
homocysteine.
Other Index Terms
adult; *altitude disease; ankle brachial index; arterial wall thickness; carotid atherosclerosis; China; *cognitive defect; color Doppler flowmetry; controlled study; diet; female; human; human tissue; *hypertension; incidence; major clinical study; male; metabolism; plasma; pulse wave; structure activity relation; *vascular lesion; vegetable.

For peer review only - http://bmjopen.bmj.com/site/about/guidelines.xhtml

Link to the Ovid Full Text or citation:

Click here for full text options

Link to the External Link Resolver:

SFX

52.

Correlation between vascular aging and functional decline in exceptionally long-lived families.
Kuipers A.L., Minster R.L., Barinas-Mitchell E.J., Cristensen K., Feitosa M., Cosentino S., Andersen-Toomey S.L., Newman A.B.
Circulation. Conference: 2018 American Heart Association Scientific Sessions. United States. 138 (Supplement 1) (no pagination), 2018. Date of Publication: November 2018.
AN: 626955898
Atherosclerosis occurs with age and has been associated with increased risk of cognitive decline, dementia, and declines in physical function. We aimed to estimate both the phenotypic and genetic correlation between vascular disease, and cognitive and physical functioning in 2060 participants in the Long Life Family Study (LLFS), which recruited families with at least 2 long-lived siblings. Participants underwent B-mode carotid artery ultrasound to assess common carotid artery intimamedia thickness (IMT; mm) and interadventitial diameter (mm), as well as, multiple tests of cognitive and physical functioning: digit symbol substitution test (DSST), semantic fluency score, trail making time (s), working memory/attention score, time to complete 5 chair stands (s), gait speed from 4- meter walk (m/s), and maximum grip strength (kg). We tested for phenotypic and genetic correlation between each vascular and functional measure using the variance covariance methods implemented in SOLAR. All models were adjusted for age, age , sex, site, height, weight, hypertension, diabetes, smoking, and physical activity. Greater carotid IMT and diameter were correlated with lower grip strength, and greater carotid diameter was correlated with poorer chair stand test performance (all phenotypic P < 0.05). While there was no phenotypic correlation between vascular measures and cognitive function, there was significant genetic correlation between working memory and carotid IMT. The current analysis provides further evidence for an association between vascular aging and functional decline, and also highlights the possibility of a

For peer review only - http://bmjopen.bmj.com/site/about/guidelines.xhtml

shared genetic link between atherosclerosis and cognitive decline in exceptionally long-lived families.

Institution

(Kuipers, Minster, Barinas-Mitchell, Cristensen, Feitosa, Cosentino, Andersen-Toomey, Newman) 1Epidemiology, Univ of Pittsburgh, Pittsburgh, PA2Human Genetics, Univ of Pittsburgh, Pittsburgh, PA3Epidemiology, Biostatistics and Biodemography, Danish Aging Rsch Cntr, Odense C, Denmark4Genetics, Washington Univ of St. Louis, St. Louis, MO5Neuropsychology, Columbia Univ, New York, NY6Medicine, Boston Univ Sch of Medicine, Boston, MA

Publisher

Lippincott Williams and Wilkins

Emtree Heading

adult; *aging; arterial wall thickness; atherosclerosis; attention; cognitive defect; common carotid artery; controlled study; covariance; diabetes mellitus; digit symbol substitution test; female; genetic correlation; grip strength; height; human; hypertension; major clinical study; male; sibling; smoking; task performance; ultrasound; walking speed; working memory; conference abstract.

Candidate Terms

conference abstract [other term].

Other Index Terms

adult; *aging; arterial wall thickness; atherosclerosis; attention; cognitive defect; common carotid artery; controlled study; covariance; diabetes mellitus; digit symbol substitution test; female; genetic correlation; grip strength; height; human; hypertension; major clinical study; male; sibling; smoking; task performance; ultrasound; walking speed; working memory.

Link to the Ovid Full Text or citation:

Click here for full text options

Link to the External Link Resolver:

SFX

53.

For peer review only

Impact of carotid artery revascularization on the cognitive and functional outcome, as well as cerebral flow on TCD and brain MRI in patients with symptomatic carotid artery stenosis: A preliminary report.

Badacz R., Kablak-Ziembicka A., Urbanczyk-Zawadzka M., Banys R., Musialek P., Pieniazek P., Trystula M., Mleczko S., Roslawiecka A., Rzeznik D., Brzychczy A., Zmudka K., Przewlocki T.

Kardiologia Polska. Conference: 20th International Congress of the Polish Cardiac Society. Poland. 74 (Supplement 4) (pp 260-261), 2016. Date of Publication: September 2016.

AN: 614983380

BACKGROUND About one third of patients develop cognitive dementia following cerebral ischemic event (CIE), while 20-50% of subjects with symptomatic carotid artery stenosis (CAS) suffer from CIE recurrence during 6 months. On the other hand, prompt carotid artery revascularization (CAR) may prevent CIE recurrence, however, at the cost of cognitive function decline or new acute micro embolic lesions (MES) on brain diffusion-weighted magnetic resonance imaging (DWI-MRI). We investigated whether CAR in recent survivors of CIE related to high risk CAS may contribute to functional and cognitive outcomes. METHODS Thirteen consecutive patients (mean age 67+/-7.8y.o., 8 male) with recent CIE (18.3+/-10.5 days to CAR) related to severe CAS (mean stenosis degree: 89.8+/-7.9%, range 80-99%) were prospectively assessed with transcranial doppler (TCD) of the Willis Circle, DWI-MRI, cognitive outcome using the Montreal Cognitive Assessment (MoCA), Mini Mental Skills Examination (MMSE), and functional outcome using the modified Rankin Scale (Rs) and National Institutes of Health Stroke Scale (NIHSS) at 24 hours before CAR, at 48-72 hours and at 1 month following CAR. RESULTS Most plaques were high risk: thrombotic in 5, lipid-rich in 1, lipid-fibrotic in 5 and calcified in 2 patients, including string stenosis in 5 and ulcerations in 9 subjects, as evidenced by ultrasonography and post-CAR histological assessment of plaque debris captured by the filter in 9 subjects or plaques removed during endarterectomy in 4 subjects. One (7.7%) minor stroke was observed following CAR. Acute and subacute multiple cerebral ischemic lesions were observed in all subjects before CAR (mean lesion size of 13.5+/-10mm, range 4-37mm), while new MES following CAR were found in 5 (38.5%) subjects. After 1 month, MES persisted in 3 (23%), resolved in 2 (15.4%), decreased in 8 (61.5%) patients respectively. There was a significant increase of cerebral flow velocity in the middle and the anterior cerebral artery on the site of CAR following intervention (from 72+/-20 to 106+/-24cm/s, p=0.0008 and from 75+/-23.4 to 94+/-18.2 cm/s, p=0.056; respectively). This flow increase was correlated with cerebral perfusion increase on MRI. NIHSS and Rs significantly improved after CAR (from 3.0+/-1.2 to 1.8+/-1.1 p=0.013 and from 1.5+/-0.8 to 0.7+/-0.9 p=0.034 respectively). There was no cognitive decline as assessed by MMSE and MoCA at 1 month vs before procedure (MMSE: 27.7+/-2.5, range 22-30, vs 26.8+/-1.9, range 26-29, p=0.027; MoCa: 23.5+/-3.5, range 19-28 vs, 23.3+/-2.5, range 20-27, p=0.356). Improvement of cognitive function was found in 9 (69.2%) by MMSE and in 7 (53.8%) subjects by MoCa. CONCLUSIONS This

For peer review only - http://bmjopen.bmj.com/site/about/guidelines.xhtml

preliminary pilot study concerning many aspects of CAR following CIE indicated immediate improvement of cerebral flow seen on TCD and perfusion MRI. Furthermore, we did not find cognitive decline when urgent CAR was performed for secondary stroke prevention in this high risk group. Large studies are necessary.

Institution

(Badacz, Kablak-Ziembicka, Pieniazek, Trystula, Mleczko, Roslawiecka, Rzeznik, Zmudka, Przewlocki) Klinika Kardiologii Interwencyjnej IK UJ CM, Krakowski Szpital Specjalistyczny im. Jana Pawla II, Pradnicka 80, Krakow, Poland (Urbanczyk-Zawadzka, Banys) Osrodek Diagnostyki, Prewencji I Telemedycyny, Krakowski Szpital Specjalistyczny im. Jana Pawla II, Pradnicka 80, Krakow, Poland

(Musialek) Klinika Chorob Serca I Naczyn, Instytut Kardiologii, CM UJ, Pradnicka 80, Krakow, Poland

(Brzychczy) Oddzial Chirurgii Naczyn z Pododdzialem Zabiegow, Endowaskularnych Krakowski Szpital Specjalistyczny im. Jana Pawla II, Pradnicka 80, Krakow, Poland

Publisher

Via Medica

Emtree Heading

aged; *angiogenesis; *anterior cerebral artery; brain circulus arteriosus; brain perfusion; cancer size; *carotid artery obstruction; cerebrovascular accident; clinical article; cognitive defect; controlled study; *diffusion weighted imaging; endarterectomy; filter; flow rate; high risk population; human; ischemia; male; Montreal cognitive assessment; National Institutes of Health Stroke Scale; perfusion weighted imaging; pilot study; prevention; Rankin scale; skill; survivor; thrombosis; transcranial doppler; ulcer; lipid.

Drug Index Terms

lipid.

Other Index Terms

aged; *angiogenesis; *anterior cerebral artery; brain circulus arteriosus; brain perfusion; cancer size; *carotid artery obstruction; cerebrovascular accident; clinical article; cognitive defect; controlled study; *diffusion weighted imaging; endarterectomy; filter; flow rate; high risk population; human; ischemia; male; Montreal cognitive assessment; National Institutes of Health Stroke Scale; perfusion weighted imaging; pilot study; prevention; Rankin scale; skill; survivor; thrombosis; transcranial doppler; ulcer.

Link to the Ovid Full Text or citation:

Click here for full text options

Link to the External Link Resolver:

For peer review only - http://bmjopen.bmj.com/site/about/guidelines.xhtml

SFX

54.

Cognitive function and cerebrovascular reserve in patients with severe stenoocclusive disease of an internal carotid artery or a middle cerebral artery.

Ishikawa M., Saito H., Soma N., Yamaguro T., Ikoda M., Ebihara A., Kusaka G., Tanaka Y.

Journal of Cerebral Blood Flow and Metabolism. Conference: 27th International Symposium on Cerebral Blood Flow, Metabolism and Function and 12th International Conference on Quantification of Brain Function with PET. Canada. 36 (Supplement 1) (pp 204-205), 2016. Date of Publication: June 2016.

AN: 611615325

Objectives: Patients with severe steno-occlusive disease of a main cerebral artery may demonstrate cognitive impairment without focal neurological deficits and without identification of causative lesions on magnetic resonance imaging (MRI), but the pathophysiology of this condition has not been characterized. We investigated whether cognitive impairment in these patients is associated with cerebral blood flow (CBF), cerebrovascular reserve (CVR), leukoaraiosis, and risk factors of atherosclerosis and whether the CVR decreases widespread-nonselectively on both sides.

Method(s): In 65 patients with severe steno-occlusive disease of an internal carotid artery (ICA) or a middle cerebral artery (MCA), we examined cognitive function with COGNISTAT (the Japanese version of the neurobehavioral cognitive status examination), grades of periventricular hyperintensity (PVH) and deep subcortical white matter hyperintensity (DSWMH) as measured by MRI and cerebral blood flow (CBF) and cerebral vascular reserve (CVR) as calculated by iodine-123-N-isopropyl-p-iodoamphetamine single photon emission computed tomography (123IMP-SPECT) and blood data (hemoglobin A1c [HbA1c], total cholesterol, triglycerides, low-density lipoprotein [LDL] cholesterol, high-density lipoprotein [HDL] cholesterol). In 15 patients who underwent superficial temporal artery (STA)-middle cerebral artery (MCA) anastomosis, the measured values were compared with those collected postoperatively.

Result(s): Logistic regression analysis revealed that both CVR and DWFMH correlated with cognitive impairment. There was no significant difference in CBF, CVR, or COGNISTAT score when comparing the left side and right side. There were good correlations between CBF or CVR of the ipsilateral MCA area and those of all other areas. For example, in the CBFs of ipsilateral MCA area and contralateral MCA area at the anterior horn level of the lateral ventricle, the regression equation was $Y = 0.70x + 14.3$, the correlation coefficient was 0.81, and the p value as < 0.0001. In

For peer review only - http://bmjopen.bmj.com/site/about/guidelines.xhtml

the CVRs between ipsilateral MCA area and contralateral MCA area at the anterior horn level of the lateral ventricle, the regression equation was Y = 0.52x + 33.4, the correlation coefficient was 0.64 and the p value was < 0.0001. In patients who underwent STA-MCA anastomosis, both postoperative CVR and cognitive impairment improved, and the correlations between CBF or CVR of the ipsilateral MCA area and those of all other areas were maintained. However, the COGNISTAT score did not change in the matched control group (without STA-MCA anastomosis) Conclusion(s): Cognitive impairment is associated with CVR in the whole brain, and nonselective widespread disconnections may be a reason for cognitive impairment in patients with severe steno-occlusive disease of a main cerebral artery. Cognitive impairment and CVR improved after STA-MCA anastomosis, compared to preoperative values.

Institution

(Ishikawa, Saito, Soma, Yamaguro, Ikoda, Ebihara, Kusaka, Tanaka) Neurosurgery, Saitama Medical Center, Jichi Medical University, Saitama, Japan

Publisher

Nature Publishing Group

Emtree Heading

artery anastomosis; atherosclerosis; brain blood flow; cholesterol blood level; *cognitive defect; control group; controlled study; correlation coefficient; human; *internal carotid artery; lateral brain ventricle; leukoaraiosis; logistic regression analysis; major clinical study; *middle cerebral artery; nuclear magnetic resonance imaging; risk factor; single photon emission computer tomography; spinal cord ventral horn; statistical significance; superficial temporal artery; surgery; white matter; cholesterol; hemoglobin A1c; high density lipoprotein; low density lipoprotein; triacylglycerol.

Drug Index Terms

cholesterol; hemoglobin A1c; high density lipoprotein; low density lipoprotein; triacylglycerol.

Other Index Terms

artery anastomosis; atherosclerosis; brain blood flow; cholesterol blood level; *cognitive defect; control group; controlled study; correlation coefficient; human; *internal carotid artery; lateral brain ventricle; leukoaraiosis; logistic regression analysis; major clinical study; *middle cerebral artery; nuclear magnetic resonance imaging; risk factor; single photon emission computer tomography; spinal cord ventral horn; statistical significance; superficial temporal artery; surgery; white matter.

Link to the Ovid Full Text or citation:

Click here for full text options

Link to the External Link Resolver:

SFX

For peer review only - http://bmjopen.bmj.com/site/about/guidelines.xhtml

57.

The preservation of cognition 1 year after carotid endarterectomy in patients with prior cognitive decline.

Dempsey R.J., Jackson D.C., Wilbrand S.M., Mitchell C.C., Berman S.E., Johnson S.C., Meshram N.H., Varghese T., Hermann B.P.

Clinical Neurosurgery. 82 (3) (pp 322-328), 2018. Date of Publication: 01 Mar 2018.

AN: 621480677

BACKGROUND: Vascular cognitive decline is critically important in the course of atherosclerosis and stroke.

OBJECTIVE(S): To explore the hypothesis that carotid endarterectomy (CEA) by removing an unstable plaque may slow the course of vascular cognitive decline in both symptomatic and asymptomatic patients.

METHOD(S): Patients with clinically significant (>60%) carotid stenosis were studied preop and 1 yr post-CEA for clinical symptoms, vascular cognitive decline, instability of carotid plaque-presence of microemboli, brain white matter changes, and medical risk factors.

RESULT(S): Forty-six percent were classically symptomatic. All patients showed vascular cognitive decline at presentation which correlated with degree of plaque instability. Significant white matter hyperintensity changes (48.7%) and cerebral emboli (25%) were also seen at baseline in both classically symptomatic and asymptomatic. One year after CEA, both groups showed no decline in cognitive function and significant improvement in 2 tests (P = .028 and P = .013). Brain white matter hyperintensities were unchanged. Microemboli were reduced but remained present (17.86%). Improvement was predicted by the presence of hypertension (P = .001), or less advanced cognitive decline preoperatively (P = .009).

CONCLUSION(S): This study demonstrates the importance of vascular cognitive decline in atherosclerotic disease. This is a function of the degree of instability of the atherosclerotic plaque more than the presence of stroke symptoms. It further suggests that atherosclerotic vascular cognitive decline need not be inevitable, and may be modified by treating hypertension and removal of the unstable plaque. This highlights the need for continued research on the cognitive effects of cerebrovascular disease and the synergistic benefits of intensive medical and surgical therapy.

PMID

28575478 [http://www.ncbi.nlm.nih.gov/pubmed/?term=28575478]

For peer review only

For peer review only - http://bmjopen.bmj.com/site/about/guidelines.xhtml

Institution
(Dempsey, Wilbrand) Department of Neurological Surgery, University of Wisconsin School of Medicine and Public Health, 600 Highland Avenue, Madison, WI 53792, United States (Jackson) Wisconsin Surgical Outcomes Center Research Program, Department of Surgery, University of Wisconsin School of Medicine and Public Health, Madison, WI, United States
(Mitchell) Department of Medicine, Cardiovascular Medicine Division, University of Wisconsin School of Medicine and Public Health, Madison, WI, United States
(Berman, Johnson) Alzheimer's Disease Research Center, University of Wisconsin School of Medicine and Public Health, Madison, WI, United States
(Meshram, Varghese) Department of Medical Physics, University of Wisconsin School of Medicine and Public Health, Madison, WI, United States
(Meshram) Department of Electrical and Computer Engineering, University of Wisconsin-Madison, Madison, WI, United States
(Hermann) Department of Neurology, University of Wisconsin School of Medicine and Public Health, Centennial Building, Madison, WI, United States
Publisher
Oxford University Press
Emtree Heading
aged; attention; brain embolism; carotid artery obstruction/su [Surgery]; carotid atherosclerosis/su [Surgery]; *carotid endarterectomy; cerebrovascular accident; clinical article; *cognition; *cognitive defect; diabetes mellitus; executive function; female; gray matter; heart cycle; human; hyperlipidemia; hypertension; intelligence quotient; male; neuropsychological test; nuclear magnetic resonance imaging; preoperative evaluation; priority journal; review; symptom; transient ischemic attack; verbal memory; white matter; working memory; transcranial doppler.
Device Index Terms
transcranial doppler.
Other Index Terms
aged; attention; brain embolism; carotid artery obstruction / surgery; carotid atherosclerosis / surgery; *carotid endarterectomy; cerebrovascular accident; clinical article; *cognition; *cognitive defect; diabetes mellitus; executive function; female; gray matter; heart cycle; human; hyperlipidemia; hypertension; intelligence quotient; male; neuropsychological test; nuclear magnetic resonance imaging; preoperative evaluation; priority journal; Review; symptom; transient ischemic attack; verbal memory; white matter; working memory.

Link to the Ovid Full Text or citation:
Click here for full text options

For peer review only - http://bmjopen.bmj.com/site/about/guidelines.xhtml

Link to the External Link Resolver:
SFX

60.

Association between Carotid Plaque and Cognitive Impairment in Chinese Stroke Population: The SOS-Stroke Study.
Wang A., Liu X., Chen G., Hao H., Wang Y.
Scientific reports. 7 (1) (pp 3066), 2017. Date of Publication: 08 Jun 2017.
AN: 625842339
We aimed to investigate the association between carotid plaques and cognitive impairment among patients with acute ischemic stroke, and to assess key clinical implications. In the Acute Ischemic Stroke Study, patients who received a cognitive testing and underwent complete carotid artery ultrasound scans were included. Cognitive function was measured by the mini-mental state examination. The cross-sectional relationships between cognitive impairment and carotid plaques were evaluated using multivariate logistic regression analysis. Of the 3116 patients included in this study, 826 (26.51%) patients were diagnosed with cognitive impairment. After adjusting for potential confounders, patients with >=2 carotid plaques (odds ratio [OR]=1.47; 95% confidence interval [CI]: 1.19-1.82), patients with >=2 number of carotid arteries with plaque (OR=1.48; 95% CI: 1.19-1.84) and patients with hypoechoic plaque (OR=2.05; 95% CI: 1.24-3.38) are more likely to have cognitive impairment. In this acute ischemic stroke population, the number of carotid plaques, the number of carotid arteries with plaque and plaque stability are all associated with cognitive impairment.
PMID
28596524 [http://www.ncbi.nlm.nih.gov/pubmed/?term=28596524]
Institution
(Wang, Wang, Wang) Department of Neurology, Beijing Tiantan Hospital, Capital Medical University, Beijing, China (Wang, Wang, Wang) China National Clinical Research Center for Neurological Diseases, Beijing, China
(Wang, Wang, Wang) Center of Stroke, Beijing Institute for Brain Disorders, Beijing, China
(Wang, Wang, Wang) Beijing Key Laboratory of Translational Medicine for Cerebrovascular Disease, Beijing, China
(Liu) Department of Cardiology, Tangshan People's Hospital, Tangshan, China

For peer review only - http://bmjopen.bmj.com/site/about/guidelines.xhtml

(Chen) Department of Neurology, Tangshan Gongren Hospital, North China University of Science and Technology, Tangshan, China
(Hao) Department of Neurology, Peking University First Hospital, Beijing, China

Emtree Heading
aged; carotid artery disease/ep [Epidemiology]; cerebrovascular accident/ep [Epidemiology]; China; cognitive defect/ep [Epidemiology]; complication; female; human; male; middle aged.
Other Index Terms
aged; carotid artery disease / epidemiology; cerebrovascular accident / epidemiology; China; cognitive defect / epidemiology; complication; female; human; male; middle aged.

Link to the Ovid Full Text or citation:
Click here for full text options

Link to the External Link Resolver:
SFX

61.

Cognitive function of patients with rheumatoid arthritis is associated with disease activity but not carotid atherosclerotic changes.
Lee J.H., Kim G.-T., Kim Y.-K., Lee S.-G.
Clinical and Experimental Rheumatology. 36 (5) (pp 856-861), 2018. Date of Publication: 01 Sep 2018.
AN: 624109476
>Objective Although the relationship between atherosclerosis and cognitive impairment has been studied and replicated, whether cognitive deficits in RA can be attributed to atherosclerotic changes is not well understood. This study investigated cognitive function in patients with RA and evaluated whether cognitive function was affected by carotid arterial atherosclerosis. Methods We examined 70 RA patients and 40 healthy controls. RA activity was assessed by disease activity score with 28 joint-erythrocyte sedimentation rate (DAS28-ESR). Cognitive function was assessed by the Korean version of the Consortium to Establish a Registry for Alzheimer's disease (CERAD-K) neuropsychological battery. Carotid arteries were scanned for the presence of plaques and to assess intima-media thickness (IMT). We assessed potential risk factors of cognitive impairment in

For peer review only

RA patients using regression analyses. Results There was a significant difference between RA patients and healthy controls in the verbal fluency (p=0.004) and Boston naming test (p=0.035). Carotid ultrasound revealed significantly more plaque in RA patients than in healthy controls (p=0.017). RA patients with memory impairment had significantly higher DAS28-ESR scores (p<0.001), age (p=0.009), and mean cIMT (p=0.027) than RA patients without memory impairment. In multivariable regression analysis, CERAD-K total score showed a significant negative correlation with age (beta=-0.415, p<0.001) or DAS28-ESR (beta=-4.685, p<0.001), but no correlation was found between CERAD-K total score and presence of plaque or cIMT. Conclusion Our results indicate that disease activity of RA and aging contribute to cognitive dysfunction, but there was no association between cognitive function and carotid atherosclerotic changes in RA patients.

© Copyright CliniCal and ExpErimEntal rhEumatology 2018.

PMID

29652660 [http://www.ncbi.nlm.nih.gov/pubmed/?term=29652660]

Institution

(Lee) Division of Rheumatology, Department of Internal Medicine, Maryknoll Medical Center, Busan, South Korea (Kim, Kim) Division of Rheumatology, Department of Internal Medicine, Kosin University, College of Medicine, Busan, South Korea

(Lee) Division of Rheumatology, Department of Internal Medicine, Pusan National University Hospital, Busan, South Korea

Publisher

Clinical and Experimental Rheumatology S.A.S. (via Santa Maria 31, Pisa 56126, Italy. E-mail: info@clinexprheumatol.org)

Emtree Heading

adult; age; aged; arterial wall thickness; article; atherosclerotic plaque; Boston naming test; *carotid atherosclerosis; *cognitive defect; controlled study; DAS28; *disease activity; echography; female; human; major clinical study; male; memory disorder; middle aged; neuropsychological test; priority journal; prospective study; *rheumatoid arthritis; risk factor; trail making test; C reactive protein/ec [Endogenous Compound].

Drug Index Terms

C reactive protein / endogenous compound.

Other Index Terms

adult; age; aged; arterial wall thickness; Article; atherosclerotic plaque; Boston naming test; *carotid atherosclerosis; *cognitive defect; controlled study; DAS28; *disease activity; echography; female; human; major clinical study; male; memory disorder; middle aged; neuropsychological test; priority journal; prospective study; *rheumatoid arthritis; risk factor; trail making test.

For peer review only

Link to the Ovid Full Text or citation:

Click here for full text options

Link to the External Link Resolver:

SFX

71.

Pulse wave velocity is associated with greater risk of dementia in mild cognitive impairment patients.

Rouch L., Cestac P., Sallerin B., Andrieu S., Bailly H., Beunardeau M., Cohen A., Dubail D., Hernandorena I., Seux M.L., Vidal J.S., Hanon O.

Journal of Hypertension. Conference: 27th Scientific Meeting of the International Society of Hypertension, ISH 2018. China. 36 (Supplement 3) (pp e340), 2018. Date of Publication: October 2018.

AN: 625308676

Objectives: To investigate the association between pulse wave velocity (PWV), intima-media thickness (IMT), carotid artery diameter (CAD), carotid plaques (CP) and conversion from mild cognitive impairment (MCI) to dementia.

Method(s): 375 elderly ambulatory subjects with a diagnosis of MCI were followed yearly to examine potential conversion to dementia up to 6 years. Vascular function was assessed by PWV considered to be the gold standard measurement of aortic stiffness. Vascular structure was evaluated by IMT, CAD and CP using an ultra-sonographic assessment of carotid arteries.

Result(s): 105 patients (28%) converted from MCI to dementia during a mean follow-up period of 4.5 years. Higher PWV was associated with greater risk of conversion from MCI to dementia (1-SD increase of PWV: HR = 1.31; 95% CI [1.03-1.67]; p = 0.03). This relationship was independent of age, sex, educational level, systolic blood pressure, cardiovascular diseases, Mini Mental State Examination at baseline and Apolipoproteine 4 status. IMT, CP and CAD did not predict conversion from MCI to dementia (1-SD increase of IMT: HR = 0.96; 95% CI [0.76-1.21]; p = 0.74; presence of CP: HR = 1.08; 95% CI [0.63-1.86]; p = 0.77; 1-SD increase of CAD: HR = 1.08; 95% CI [0.89-1.31]; p = 0.44).

Conclusion(s): In the present study, arterial stiffness, measured as PWV, predicted conversion from MCI to dementia whereas IMT, CP nor CAD did not after controlling for age and other

For peer review only

confounding factors. Arterial stiffness could help to better predict MCI patients at higher risk of dementia and may be a therapeutic target to delay or prevent the onset of dementia.

Institution

(Rouch, Sallerin, Bailly, Cohen, Dubail, Hernandorena, Seux, Vidal, Hanon) Geriatrics Broca Hospital, France (Cestac) Pharmacy, Toulouse University Hospital, France

(Andrieu) Epidemiology and Public Health INSERM 1027, France

(Rouch, Beunardeau) Geriatrics BROCA HOSPITAL, France

Publisher

Lippincott Williams and Wilkins

Emtree Heading

aged; *arterial stiffness; *arterial wall thickness; *artery diameter; cardiovascular disease; controlled study; *dementia; diagnosis; female; follow up; gold standard; human; major clinical study; male; *mild cognitive impairment; Mini Mental State Examination; prevention; *pulse wave; *risk assessment; systolic blood pressure; conference abstract.

Candidate Terms

conference abstract [other term].

Other Index Terms

aged; *arterial stiffness; *arterial wall thickness; *artery diameter; cardiovascular disease; controlled study; *dementia; diagnosis; female; follow up; gold standard; human; major clinical study; male; *mild cognitive impairment; Mini Mental State Examination; prevention; *pulse wave; *risk assessment; systolic blood pressure.

Link to the Ovid Full Text or citation:

Click here for full text options

Link to the External Link Resolver:

SFX

72.

Impact of the hypertension and carotid intima-media thickness to the infarcts volume in the stroke patients.

Lobjanidze N., Chitauri N.

For peer review only - http://bmjopen.bmj.com/site/about/guidelines.xhtml

Journal of Hypertension. Conference: 27th Scientific Meeting of the International Society of Hypertension, ISH 2018. China. 36 (Supplement 3) (pp e319-e320), 2018. Date of Publication: October 2018.

AN: 625308484

Objectives: Cognitive deficit among hypertensive elderly patients with brain in-farcts was established during last years, represents the basis of vascular dementia in late life still, but their link with intima media thickness (IMT) remains controversially as well as the stroke volume The aim of this study was assesment of IMT among hypertensive poststroke patients(PSL) with lacunar lesions(MSL) Methods: Prospective study of 147(mean age 71,4) patients was carried out. The patients were divided in two groups: I group(85)-moderate stroke patients without hypertension.Type, side and site of stroke was assesed by conventional MRI. II group(62)-moderate/severe hypertensive stroke patients, who had multiple lacunar lesions in MRI. Cognitive function was investigated in both groups by neuropsy-chological battey (letter fluency, Stroop test, Wisconsin Card Sorting Test, digit span, letter number sequensing), MMSE. Ultrasonographically assessed carotid artery IMT.They were compaired due to vascular risk factors, clinical, demographic and radiological variables. Statistical evaluation was perform by SPSS Results: From 85 PSL patients dementia was diagnosed in13(15,2%). cases, depression in 24(28,2%).This group often had damage of the left hemisphere, prevalence of ischemic with basal ganglia lesions. No one from the 65 MSL patients had dementia, but 29(44,6%) had mild cognitive impairment and 36(55.3%) had depression. Atherogenic index and Crotid Intima-Media Sickness was significantly higher in patients with multiple lacunar infarction lesion and associated with hypertension, age and female gender

Conclusion(s): Carotid IMT predicts an increased risk for cognitive impairment, particularly poor memory and cognitive speed, in elderly women, and leads to multiple lacunar silent infarct lesions.

Institution

(Lobjanidze, Chitauri) Neurology, S.Khechinashvili Medical University Hospital, Georgia

Publisher

Lippincott Williams and Wilkins

Emtree Heading

aged; amnesia; *arterial wall thickness; basal ganglion; cardiovascular risk; diagnosis; female; gender; heart stroke volume; human; *hypertension; *lacunar stroke; left hemisphere; major clinical study; male; mild cognitive impairment; Mini Mental State Examination; *multiinfarct dementia; nerve biopsy; nuclear magnetic resonance imaging; prevalence; prospective study; risk assessment; *stroke patient; Stroop test; velocity; Wisconsin Card Sorting Test; conference abstract.

Candidate Terms

conference abstract [other term].

Other Index Terms

For peer review only - http://bmjopen.bmj.com/site/about/guidelines.xhtml

aged; amnesia; *arterial wall thickness; basal ganglion; cardiovascular risk; diagnosis; female; gender; heart stroke volume; human; *hypertension; *lacunar stroke; left hemisphere; major clinical study; male; mild cognitive impairment; Mini Mental State Examination; *multiinfarct dementia; nerve biopsy; nuclear magnetic resonance imaging; prevalence; prospective study; risk assessment; *stroke patient; Stroop test; velocity; Wisconsin Card Sorting Test.

Link to the Ovid Full Text or citation:
Click here for full text options

Link to the External Link Resolver:
SFX

75.

Study on the correlation of vascular lesions and cognitive function in native Tibetan with h-type hypertension in plateau.
Li Y.L., Lou M.Y., Feng J.H., Yang Y., Xu L.F.
Journal of the American Geriatrics Society. Conference: 2018 Annual Scientific Meeting of the American Geriatrics Society, AGS 2018. United States. 66 (Supplement 3) (pp S501-S502), 2018.
Date of Publication: September 2018.
AN: 623841410
Objective: To explore characteristics of Vascular Lesions, the correlation of vascular lesions and cognitive function in native Tibetan patients with H-type hypertension (Hcy >= 15mumol/L) in Qinghai Plateau of China.
Method(s): Sixty-two cases with H-type hypertension (Hcy >= 15 mumol /L) and 178 cases with non-H-type hypertension (Hcy < 15 mumol /L) were enrolled from native Tibetan patients at high altitude (Yushu city, Qinghai Province, 3800m). All the subjects were detected with the color Doppler ultrasound, the Colin-VP1000 type artery stiff diagnosis apparatus and the mini-mental state examination (MMSE) for the Carotid artery atherosclerotic plaques, carotid intima-media thickness (CIMT), the brachial-ankle pulse wave velocity (BaPWV) and ankle brachial index (ABI).
Result(s): The incidence of Carotid artery atherosclerotic plaques, CIMT and BaPWV were significantly higher in the group with H-type as compared with non-H-type hypertension (chi2 = 21.35, P < .05), while MMSE and ABI were significantly decreased (P < .05). MMSE were positively

For peer review only - http://bmjopen.bmj.com/site/about/guidelines.xhtml

For peer review only

correlated with BaPWV, CIMT and plaques amounts, and MMSE was negatively correlated with ABI and plaques amounts.

Conclusion(s): The patients with H-type hypertension are more likely to have the peripheral artery structure and function impairments and have more severe cognition impairment. which closely correlate with high altitude and hypoxia. Hypoxia and special national diet (less vegetables and more meat) affect the metabolism of homocysteine. Elevated plasma Hcy level may aggravate vascular lesions and cognitive impairment in native Tibetan patients with high blood pressure in plateau. (Table Presented).

Institution

(Li, Lou, Feng, Yang, Xu) General Department, Qinghai Provincial People's Hospital, Xining, Qinghai 810007, China

Publisher

Blackwell Publishing Inc.

Emtree Heading

adult; altitude disease; ankle brachial index; arterial wall thickness; atherosclerotic plaque; *cognitive defect; color Doppler flowmetry; controlled study; diagnosis; diet; female; human; human tissue; *hypertension; incidence; major clinical study; male; metabolism; Mini Mental State Examination; plasma; pulse wave; structure activity relation; *vascular lesion; vegetable; homocysteine; conference abstract.

Candidate Terms

conference abstract [other term].

Drug Index Terms

homocysteine.

Other Index Terms

adult; altitude disease; ankle brachial index; arterial wall thickness; atherosclerotic plaque; *cognitive defect; color Doppler flowmetry; controlled study; diagnosis; diet; female; human; human tissue; *hypertension; incidence; major clinical study; male; metabolism; Mini Mental State Examination; plasma; pulse wave; structure activity relation; *vascular lesion; vegetable.

Link to the Ovid Full Text or citation:

Click here for full text options

Link to the External Link Resolver:

SFX

For peer review only

76.

Carotid plaque MRI features embolic complications and effects on cognition after carotid artery stenting.

Eraslan C., Cinar C., Guler A., Dogan O.S., Akkus D.E., Kitis O., Calli M.C., Oran I.

Neuroradiology. Conference: 41st European Society of Neuroradiology Diagnostic and Interventional Annual Meeting - ESNR 2018, , the 25th Advanced Course in Diagnostic Neuroradiology and the 10th Advanced Course in Endovascular and Interventional Neuroradiology. Netherlands. 60 (Supplement 2) (pp S450), 2018. Date of Publication: September 2018.

AN: 623700019

Aim To put forth the relationship between the plaque tissue features assessed with MRI before carotid artery stenting(CAS) and the embolic complications-cognitive changes that develop after the procedure. Methods Thirtyone patients for whom CAS was planned were included in the study. Conventional plaque MRI, susceptibility weighted imaging(SWI) for plaque and difusion weighted images(DWI) for both plaque and cranium were obtained in 3T MR system before the procedure. In the first day after CAS, cranial DWI was repeated to search the existence of new appearing ischemia. Cognitive tests were conducted to all patients before the procedure, in the first 48 hours and three months after the procedure. Plaques which has partially or completely rich fat content or the ones have intraplaque haemorrhage was accepted as vulnerable. Results Thirteen patients had vulnerable plaques according to the MRI findings. Acute ischemic changes of the patients with vulnerable plaque were found to be significantly higher than the patients with stable plaque after the procedure($p<0,001$). A significant difference was also found in the patient group with vulnerable plaque between the cognitive tests conducted before and after CAS and in the third month (p:0,011 and p:0,006 respectively). In the patient group with stable plaque, there was no statistically significant difference. Conclusion According to the results of our study, through assessing the plaque features with plaque MRI obtained with SWI and DWI before CAS procedure, it is possible to predict the development of embolic complications and cognitive changes that can arise after the procedure.

Institution

(Eraslan, Cinar, Kitis, Calli, Oran) Ege University, Department of Radiology, Izmir, Turkey (Guler, Dogan, Akkus) Ege University, Department of Neurology, Izmir, Turkey

Publisher

Springer Verlag

Emtree Heading

adult; bleeding; *carotid artery stenting; clinical article; *cognition; complication; controlled study; fat content; female; human; ischemia; male; *susceptibility weighted imaging; conference abstract.
Candidate Terms
conference abstract [other term].
Other Index Terms
adult; bleeding; *carotid artery stenting; clinical article; *cognition; complication; controlled study; fat content; female; human; ischemia; male; *susceptibility weighted imaging.

Link to the Ovid Full Text or citation:
Click here for full text options

Link to the External Link Resolver:
SFX

77.

The association between carotid flow and cognitive function in the community elderly population.
Chuang S.Y., Cheng H.M., Yip B.S., Chen C.H., Pan W.H.
Journal of Hypertension. Conference: 28th Scientific Meeting of the European Society of Hypertension, ESH 2018. Spain. 36 (Supplement 1) (pp e90), 2018. Date of Publication: June 2018.
AN: 623097110
Objective: Carotid hemodynamics, such as intima-media thickness and carotid fl ow velocity were associated with stroke events. However, the association between carotid hemodynamics and cognitive function remains not fully clear. We aimed to investigate the relationship between carotid fl ow velocity and cognitive function. Design and method: A total of 744 elderly (more than 60 years) subjects completed the baseline and followed ultrasound examinations and those were evaluated cognitive function. Cognitive function was evaluated by MMSE and cognitive function impairment was defined by the MMSE less than 26. The peak-systolic velocity, end-diastolic velocity were measured in the common carotid arteries. Logistic regression was used to evaluate the association between carotid fl ow velocities, carotid diameters and cognitive function. Result(s): A total of 744 elderly subjects completed all examinations during the followed period. The prevalence Cognitive function impairment (MMSE less than 26) was 13.3% (n = 99). The peak systolic velocity (PSV) and diastolic end velocity (EDV) were lower in those with cognitive function

For peer review only - http://bmjopen.bmj.com/site/about/guidelines.xhtml

impairment (60.5 vs. 65.5 cm/sec, p-value < 0.001 for PSV and 19.7 vs. 22.1 cm/sec, p-value < 0.001 for EDV), and only peak-systolic velocity remains significant in the multivariable models. Moreover, those with lower carotid fl ow velocities (the lowest 10th of peak systolic velocity) had 9.69 fold risk (95% confidence intervals: 2.75-34.21) of cognitive function impairment, compared to those with the highest 10th of peak systolic velocity. The significant association remains in the multivariable model by adjusting for age, gender, education, brachial systolic BP, fasting glucose, and low density lipoprotein cholesterol.

Conclusion(s): Low carotid fl ow velocity, especially peak-systolic velocity was associated with cognitive function impairment. Lower carotid fl ow may involve the pathogen of cognitive function impairment in the general elderly population.

Institution

(Chuang) Institute of Population Health Sciences, National Health Research Institutes, Miaoli, Taiwan (Republic of China) (Cheng, Chen) Department of Medicine, Taipei Veterans General Hospital, Taipei, Taiwan (Republic of China)

(Yip) Department of Neurology, National Taiwan University Hospital, Hsin- Chu Branch, Hsin-Chu city, Taiwan (Republic of China)

(Pan) Institute of BioMedical Science, Academia Sinica, Taipei, Taiwan (Republic of China)

Publisher

Lippincott Williams and Wilkins

Emtree Heading

aged; *carotid artery flow; clinical assessment; *cognition; common carotid artery; controlled study; education; female; gender; glucose blood level; human; human experiment; infectious agent; major clinical study; male; Mini Mental State Examination; nonhuman; peak systolic velocity; prevalence; statistical significance; systolic blood pressure; ultrasound; low density lipoprotein cholesterol; conference abstract.

Candidate Terms

conference abstract [other term].

Drug Index Terms

low density lipoprotein cholesterol.

Other Index Terms

aged; *carotid artery flow; clinical assessment; *cognition; common carotid artery; controlled study; education; female; gender; glucose blood level; human; human experiment; infectious agent; major clinical study; male; Mini Mental State Examination; nonhuman; peak systolic velocity; prevalence; statistical significance; systolic blood pressure; ultrasound.

Link to the Ovid Full Text or citation:

For peer review only - http://bmjopen.bmj.com/site/about/guidelines.xhtml

Click here for full text options

Link to the External Link Resolver:
SFX

85.

Asymptomatic carotid stenosis is associated with cognitive impairment.
Lal B.K., Dux M.C., Sikdar S., Goldstein C., Khan A.A., Yokemick J., Zhao L.
Journal of Vascular Surgery. 66 (4) (pp 1083-1092), 2017. Date of Publication: October 2017.
AN: 617342339

Background Cerebrovascular risk factors (eg, hypertension, coronary artery disease) and stroke can lead to vascular cognitive impairment. The Asymptomatic Carotid Stenosis and Cognitive Function study evaluated the isolated impact of asymptomatic carotid stenosis (no prior ipsilateral or contralateral stroke or transient ischemic attack) on cognitive function. Cerebrovascular hemodynamic and carotid plaque characteristics were analyzed to elucidate potential mechanisms affecting cognition. Methods There were 82 patients with >=50% asymptomatic carotid stenosis and 62 controls without stenosis but matched for vascular comorbidities who underwent neurologic, National Institutes of Health Stroke Scale, and comprehensive neuropsychological examination. Overall cognitive function and five domain-specific scores were computed. Duplex ultrasound with Doppler waveform and B-mode imaging defined the degree of stenosis, least luminal diameter, plaque area, and plaque gray-scale median. Breath-holding index (BHI) and microembolization were measured using transcranial Doppler. We assessed cognitive differences between stenosis patients and control patients and of stenosis patients with low vs high BHI and correlated cognitive function with microembolic counts and plaque characteristics. Results Stenosis and control patients did not differ in vascular risk factors, education, estimated intelligence, or depressive symptoms. Stenosis patients had worse composite cognitive scores (P =.02; Cohen's d = 0.43) and domain-specific scores for learning/memory (P =.02; d = 0.42) and motor/processing speed (P =.01; d = 0.65), whereas scores for executive function were numerically lower (P =.08). Approximately 49.4% of all stenosis patients were impaired in at least two cognitive domains. Precisely 50% of stenosis patients demonstrated a reduced BHI. Stenosis patients with reduced BHI performed worse on the overall composite cognitive score (t = -2.1; P =.02; d = 0.53) and tests for learning/memory (t = -2.7; P =.01; d = 0.66). Cognitive function did not correlate with measures of plaque burden (degree of stenosis, least luminal diameter, and plaque area) or with plaque gray-

For peer review only - http://bmjopen.bmj.com/site/about/guidelines.xhtml

scale median. Conclusions Asymptomatic carotid stenosis is associated with cognitive impairment independent of known vascular risk factors for vascular cognitive impairment. Approximately 49.4% of these patients demonstrate impairment in at least two neuropsychological domains. The deficit is driven primarily by reduced motor/processing speed and learning/memory and is mild to moderate in severity. The mechanism for impairment is likely to be hemodynamic as evidenced by reduced cerebrovascular reserve and the likely result of hypoperfusion from a pressure drop across the stenosis in the presence of inadequate collateralization.

Copyright © 2017 Society for Vascular Surgery

PMID

28712815 [http://www.ncbi.nlm.nih.gov/pubmed/?term=28712815]

Institution

(Lal, Goldstein, Khan, Yokemick, Zhao) Department of Vascular Surgery, University of Maryland School of Medicine, Baltimore, Md, United States (Lal, Goldstein, Zhao) Vascular Service, Veterans Affairs Medical Center, Baltimore, Md, United States

(Dux) Neuropsychology Section, Veterans Affairs Medical Center, Baltimore, Md, United States

(Sikdar, Khan) Department of Bioengineering, George Mason University, Fairfax, Va, United States

Publisher

Mosby Inc. (E-mail: customerservice@mosby.com)

Emtree Heading

aged; artery embolism; *asymptomatic disease; atherosclerotic plaque; carotid artery; *carotid artery obstruction; cognition; *cognitive defect; cognitive function test; comorbidity; conference paper; controlled study; depression; disease association; echography; education; executive function; female; hemodynamics; human; intelligence; learning; major clinical study; male; medical parameters; memory; motor performance; National Institutes of Health Stroke Scale; priority journal; prospective study; risk factor; transcranial doppler; transcranial Doppler ultrasonography; ultrasound transducer; breath holding index; SonixMDP system; ST3 TCD machine.

Candidate Terms

breath holding index [other term]; SonixMDP system [device term]; ST3 TCD machine [device term].

Other Index Terms

aged; artery embolism; *asymptomatic disease; atherosclerotic plaque; carotid artery; *carotid artery obstruction; cognition; *cognitive defect; cognitive function test; comorbidity; Conference Paper; controlled study; depression; disease association; echography; education; executive function; female; hemodynamics; human; intelligence; learning; major clinical study; male; medical parameters; memory; motor performance; National Institutes of Health Stroke Scale; priority journal; prospective study; risk factor; transcranial doppler; transcranial Doppler ultrasonography; ultrasound transducer.

For peer review only

Link to the Ovid Full Text or citation:

Click here for full text options

Link to the External Link Resolver:

SFX

86.

Carotid atherosclerosis and cognitive impairment in nonstroke patients.

Chen W.-H., Jin W., Lyu P.-Y., Liu Y., Li R., Hu M., Xiao X.-J.

Chinese Medical Journal. 130 (19) (pp 2375-2379), 2017. Date of Publication: 05 Oct 2017.

AN: 618496993

Objective: As a vascular risk factor, carotid atherosclerosis is crucial to cognitive impairment. While carotid intima-media thickness, carotid artery plaque, and carotid stenosis can reflect carotid atherosclerosis in different stages, this review aimed to explore researches on the role of carotid intima-media thickness, carotid artery plaque, and carotid stenosis in the progress of cognitive impairment in nonstroke patients and tried to illustrate the possible mechanisms.

Data Sources: We searched the PubMed database for recently published research articles up to July 2017, with the key words of "carotid atherosclerosis," "carotid intima-media thickness," "carotid plaque," "carotid stenosis," "nonstroke," and "cognitive impairment." Study Selection: Articles were obtained and reviewed to analyze the role of carotid atherosclerosis such as carotid intima-thickness, carotid plaque, and carotid stenosis in the progress of cognitive impairment in nonstroke patients and the possible mechanisms.

Result(s): In recent years, most studies proved that by evaluating carotid atherosclerosis with ultrasonography, carotid atherosclerosis accounts for the development of cognitive decline in nonstroke patients. Carotid atherosclerosis not only impairs the subtle general cognitive function but also decreases the specific domains of cognitive function, such as memory, motor function, visual perception, attention, and executive function. But, it is still controversial. The possible mechanisms of cognitive impairment in nonstroke patients with carotid atherosclerosis can be classified as systemic global cerebrovascular function, small-vessel diseases, and the mixed lesions.

For peer review only - http://bmjopen.bmj.com/site/about/guidelines.xhtml
For peer review only

Conclusion(s): Carotid atherosclerosis can be used to predict the risk of cognitive impairment. Furthermore, diagnosing and treating carotid atherosclerosis at early stage might help clinicians prevent and treat vascular cognitive impairment in nonstroke patients.

Copyright © 2017 Chinese Medical Journal.

PMID

28937045 [http://www.ncbi.nlm.nih.gov/pubmed/?term=28937045]

Institution

(Chen, Lyu, Liu, Li) Graduate School, Hebei Medical University, Shijiazhuang, Hebei 050017, China

(Chen, Jin, Lyu, Liu, Li, Hu, Xiao) Department of Neurology, Hebei General Hospital, Shijiazhuang, Hebei 050051, China

Publisher

Chinese Medical Association (B9, Kanara Business Centre, off Link Road, Ghatkopar (E), Mumbai 400 075, India)

Emtree Heading

arterial wall thickness; article; attention; brain function; cardiovascular function; carotid artery disease; carotid artery obstruction; *carotid atherosclerosis; cerebrovascular accident; cerebrovascular disease; *cognitive defect/et [Etiology]; degenerative disease; disease association; disease severity; executive function; human; memory; motor performance; nonhuman; pathogenesis; risk assessment; vision; carotid artery plaque; cerebrovascular function.

Candidate Terms

carotid artery plaque [other term]; cerebrovascular function [other term].

Other Index Terms

arterial wall thickness; Article; attention; brain function; cardiovascular function; carotid artery disease; carotid artery obstruction; *carotid atherosclerosis; cerebrovascular accident; cerebrovascular disease; *cognitive defect / *etiology; degenerative disease; disease association; disease severity; executive function; human; memory; motor performance; nonhuman; pathogenesis; risk assessment; vision.

Link to the Ovid Full Text or citation:

Click here for full text options

Link to the External Link Resolver:

SFX

For peer review only

87.

Reduced Cardiovascular Functions in Patients with Alzheimer's Disease.

Jin W.-S., Bu X.-L., Wang Y.-R., Li L., Li W.-W., Liu Y.-H., Zhu C., Yao X.-Q., Chen Y., Gao C.-Y., Zhang T., Zhou H.-D., Zeng F., Wang Y.-J.

Journal of Alzheimer's Disease. 58 (3) (pp 919-925), 2017. Date of Publication: 2017.

AN: 616714109

Previous studies have suggested that cardiovascular functions might play a critical role in Alzheimer's disease (AD) pathogenesis. However, the relationship among heart function, blood flow of cerebral vessels, and AD remains unclear. In the present study, AD patients (n=34) and age- and gender-matched cognitively normal controls (n=34) were recruited. Demographic and comorbidity information was collected. The ejection fraction was measured using echocardiography, and the mean velocity, pulsatility index (PI), and resistance index (RI) of the basilar artery (BA), left terminal internal carotid artery (LTICA), and right terminal internal carotid artery (RTICA) were measured using transcranial Doppler. The data of lacunae, white matter changes, and plaques in the aortic arch and carotid arteries were collected from brain magnetic resonance imaging and computed tomography angiography images. Compared with normal controls, AD patients had lower ejection fractions and cerebral blood flow velocities and higher RI and PI in the BA, LTICA, and RTICA, as well as more plaques in the aortic and carotid arteries. In the multivariate logistic regression analysis, the ejection fraction and the mean velocity of the BA and LTICA were independently associated with AD after adjusting for age, gender, education, vascular risk factors, arterial plaques, and brain ischemic lesions detected in the brain images. These findings suggest that heart function and vascular condition may play important roles in AD pathogenesis. Improving cardiovascular functions could be a promising approach for the prevention and treatment of AD.

Copyright © 2017 - IOS Press and the authors. All rights reserved.

PMID

28505975 [http://www.ncbi.nlm.nih.gov/pubmed/?term=28505975]

Institution

(Jin, Bu, Wang, Li, Li, Liu, Zhu, Yao, Chen, Gao, Zhang, Zhou, Zeng, Wang) Department of Neurology, Center for Clinical Neuroscience, Daping Hospital, Third Military Medical University, 10 Changjiang Branch Road, Yuzhong District, Chongqing 400042, China

Publisher

IOS Press (Nieuwe Hemweg 6B, Amsterdam 1013 BG, Netherlands)

Emtree Heading

aged; *Alzheimer disease; amyloid plaque; aortic arch; artery resistance; article; basilar artery; brain blood flow; *cardiovascular function; carotid artery; clinical article; clinical assessment; computed tomographic angiography; controlled study; echocardiography; female; heart ejection fraction; human; internal carotid artery; male; measurement; multivariate logistic regression analysis; neuroimaging; neuropathology; nuclear magnetic resonance imaging; priority journal; pulsatility index; transcranial doppler; white matter.

Other Index Terms

aged; *Alzheimer disease; amyloid plaque; aortic arch; artery resistance; Article; basilar artery; brain blood flow; *cardiovascular function; carotid artery; clinical article; clinical assessment; computed tomographic angiography; controlled study; echocardiography; female; heart ejection fraction; human; internal carotid artery; male; measurement; multivariate logistic regression analysis; neuroimaging; neuropathology; nuclear magnetic resonance imaging; priority journal; pulsatility index; transcranial doppler; white matter.

Link to the Ovid Full Text or citation:

Click here for full text options

Link to the External Link Resolver:

SFX

93.

Cognitive function of patients with rheumatoid arthritis is associated with disease activity but not carotid atherosclerotic changes.

Kim G.-T., Tag H.-S., Kim Y.-K., Lee S.-G., Park E.-K., Park J.-H., Lee J.-W., Kim S.-H., Lee J.-H.

Annals of the Rheumatic Diseases. Conference: Annual European Congress of Rheumatology, EULAR 2017. Spain. 76 (Supplement 2) (pp 255), 2017. Date of Publication: June 2017.

AN: 621421856

Background: Rheumatoid arthritis (RA) is a complex inflammatory disease that has features of atherosclerosis and cognitive decline. Although the relationship between atherosclerosis and cognitive impairment has been studied and replicated, whether cognitive deficits in RA can be attributed to their atherosclerotic changes is not well understood.

For peer review only - http://bmjopen.bmj.com/site/about/guidelines.xhtml

Objective(s): This study investigated the cognitive function in patients with RA using the Korean version of the Consortium to Establish a Registry for Alzheimer's disease (CERAD-K) neuropsychological battery and evaluated whether cognitive function was affected by the carotid arterial atherosclerosis.

Method(s): We examined seventy RA patients and forty healthy controls. RA activity was assessed by disease activity score with 28 joints-erythrocyte sedimentation rate (DAS28-ESR) and objective memory impairment was defined as a performance score of 1.5 standard deviations below the respective agespecific, education-specific, and sex-specific normative means for at least one of the four episodic memory tests in CERAD-K; the Word List Memory, Word List Recall, Word List Recognition, and Constructional Recall tests [1]. Carotid arteries were scanned for the presence of plaque and intima-media thickness (IMT). We assessed potential risk factors of cognitive impairment in RA patients using regression analyses.

Result(s): Of the CERAD-K subtests, there were a significant difference between the RA patients and healthy controls in verbal fluency (12.97+/-3.73 vs 15.48+/-4.57, respectively; p=0.004) and Boston Naming Test (11.50+/-2.08 vs 12.30+/-1.77, respectively; p=0.035). Carotid ultrasound revealed significantly more plaques in the RA patients than in the healthy controls (39% vs 15%, respectively; p=0.017). RA patients with memory impairment have significantly higher score of DAS28-ESR (4.14+/-0.99 vs 2.60+/-0.88, respectively; p<0.001), age (65.71+/-7.71 vs 58.50+/-11.33, respectively; p=0.009), and mean cIMT (0.56+/-0.10 vs 0.50+/-0.08, respectively; p=0.027) compared to RA patients without memory impairment. In multivariable regression analysis, CERAD-K total score showed a significant negative correlation with age (beta=-0.415, p<0.001) or DAS28-ESR (beta=-4.685, p<0.001), but no correlation was found between CERAD-K total score and presence of plaque or cIMT (Table 1).

Conclusion(s): Our results indicate that disease activity of RA and aging contribute to cognitive impairment, but there was no association between cognitive function and clinical or subclinical carotid atherosclerotic changes in RA patients.

Institution

(Kim, Tag, Kim) Department of Internal Medicine, Kosin University College of Medicine, Busan, South Korea (Lee, Park, Park) Department of Internal Medicine, Pusan National University Hospital, Busan, South Korea

(Lee) Department of Internal Medicine, Busan St. Mary's Hospital, Busan, South Korea

(Kim) Department of Internal Medicine, Inje University College of Medicine, Busan, South Korea

(Lee) Department of Internal Medicine, Maryknoll Medical Center, Busan, South Korea

Publisher

BMJ Publishing Group

Emtree Heading

For peer review only - http://bmjopen.bmj.com/site/about/guidelines.xhtml

adult; aging; Alzheimer disease; *arterial wall thickness; *atherosclerosis; Boston naming test; clinical assessment; controlled study; disease activity score; education; *episodic memory; erythrocyte sedimentation rate; female; human; joint; major clinical study; male; memory disorder; register; regression analysis; *rheumatoid arthritis; risk assessment; risk factor; ultrasound; word list recall; conference abstract.

Candidate Terms

conference abstract [other term].

Other Index Terms

adult; aging; Alzheimer disease; *arterial wall thickness; *atherosclerosis; Boston naming test; clinical assessment; controlled study; disease activity score; education; *episodic memory; erythrocyte sedimentation rate; female; human; joint; major clinical study; male; memory disorder; register; regression analysis; *rheumatoid arthritis; risk assessment; risk factor; ultrasound; word list recall.

Link to the Ovid Full Text or citation:

Click here for full text options

Link to the External Link Resolver:

SFX

101.

Asymptomatic carotid stenosis might worsen cognitive functions in hypertensive patients.

Mineva P., Tsoneva V., Talalaev D.

European Journal of Neurology. Conference: 3rd Congress of the European Academy of Neurology. Netherlands. 24 (Supplement 1) (pp 239), 2017. Date of Publication: July 2017.

AN: 617492551

Background and aims: Systolic arterial hypertension (SAH) in midlife is a risk factor for cognitive impairment (CI) but the relationship of asymptomatic carotid stenosis (ACS>=50%) to CI is still a matter of debate. The aim of this epidemiological study is to estimate the significance of ACS>=50% for CI in hypertensive and non-hypertensive persons without signs and symptoms of stroke or TIA.

For peer review only

Method(s): A total of 500 volunteers, aged 50-79 years, were enrolled and followed-up for cognitive performance. CI has been defined as a score between 24 and 27 of MMSE. A battery of additional neuropsychological tests has also been conducted.
Result(s): Multiple logistic regression analysis has shown that ACS>=50% attributes to CI (OR=10.7; 95%CI: 3.36-34.14; p=0.0001) only in hypertensive patients with SAH but not in normotensives. Logistic regression analysis has revealed that the abnormal scores of neuropsychological tests (MMSE, DFS, DBS and VF) are significantly associated with ACS>=50% (OR 2.121; 95%CI: 1.048-4.292; p=0.036). The strongest relationship has been established between ACS>=50% and DBS (OR 10.818; 95%CI: 1.165-100.439; p=0.037). CI has presented as an executive dysfunction and decline of attention, verbal fluency and working memory.
Conclusion(s): ACS>=50% might be attributable to CI in patients with SAH. This suggests a complexity of a large and small artery dysfunction, caused by both atherosclerosis and hypertension, underlying the CI pathogenesis.
Institution
(Mineva, Tsoneva) Trakia University, Stara Zagora, Bulgaria (Talalaev) Medical University, Sofia, Bulgaria
Publisher
Blackwell Publishing Ltd
Emtree Heading
adult; aortic regurgitation; atherosclerosis; *attention; *carotid artery obstruction; cerebrovascular accident; cognitive defect; disease free survival; female; human; *hypertension; major clinical study; male; middle aged; Mini Mental State Examination; multivariate logistic regression analysis; neuropsychological test; symptom; volunteer; working memory.
Other Index Terms
adult; aortic regurgitation; atherosclerosis; *attention; *carotid artery obstruction; cerebrovascular accident; cognitive defect; disease free survival; female; human; *hypertension; major clinical study; male; middle aged; Mini Mental State Examination; multivariate logistic regression analysis; neuropsychological test; symptom; volunteer; working memory.

Link to the Ovid Full Text or citation:
Click here for full text options

Link to the External Link Resolver:
SFX

For peer review only

111.

Carotid atherosclerosis, cytomegalovirus infection, and cognitive decline in the very old: a community-based prospective cohort study.

Kawasaki M., Arai Y., Hirata T., Takayama M., Abe Y., Niimura H., Mimura M., Takebayashi T., Hirose N.

Age. 38 (2) (pp 1-13), 2016. Article Number: 29. Date of Publication: 01 Apr 2016.

AN: 608461844

To investigate various risk factors of cognitive decline in the very old, we studied 494 subjects over 85 years old without diagnosis of dementia at baseline from the Tokyo Oldest Old Survey on Total Health, an ongoing, community-based cohort in Japan. Cognitive function was assessed at baseline and at 3-year follow-up using Mini-Mental State Examination (MMSE). Plasma samples were assayed for levels of cytomegalovirus (CMV) immunoglobulin G (IgG) antibodies, tumor necrosis factor-alpha, interleukin-6, and blood chemistry. Carotid artery plaques were measured using an ultrasonography. In the cross-sectional analyses using Tobit regression, individuals with high carotid artery plaque score (>=5.0) had MMSE scores that were 1.08 points lower compared to those with no plaque (95 % confidence interval (CI) -1.95 to -0.20; p = 0.016), adjusted for age, sex, and education. Individuals with CMV IgG titers in the highest quartile had MMSE scores that were 1.47 points lower compared to individuals in the lowest quartile (95 % CI -2.44 to -0.50; p = 0.003). CMV and carotid atherosclerosis showed evidence of an interaction, where the association between CMV and MMSE was present only in subjects with carotid artery plaque. In the longitudinal analyses using linear regression, carotid atherosclerosis, smoking, low grip strength, and poor activities of daily living (ADL) status were associated with faster cognitive decline, adjusted for age, sex, education, and baseline cognitive function. Our findings suggest that carotid atherosclerosis is consistently associated with low cognitive function in the very old and modifies the association between latent CMV infection and cognition.

Copyright © 2016, American Aging Association.

Institution

(Kawasaki, Arai, Hirata, Abe, Hirose) Center for Supercentenarian Medical Research, Keio University School of Medicine, 35 Shinanomachi, Shinjuku-ku, Tokyo 160-8582, Japan (Takayama) Center for Preventive Medicine, Keio University School of Medicine, 35 Shinanomachi, Shinjuku-ku, Tokyo 160-8582, Japan

(Takayama) Keio University, 4-1-1 Hiyoshi, Kohoku-ku, Yokohama, Kanagawa 252-8520, Japan

(Niimura, Mimura) Department of Neuropsychiatry, Keio University School of Medicine, 35 Shinanomachi, Shinjuku-ku, Tokyo 160-8582, Japan

For peer review only - http://bmjopen.bmj.com/site/about/guidelines.xhtml

(Takebayashi) Department of Preventative Medicine and Public Health, Keio University School of Medicine, 35 Shinanomachi, Shinjuku-ku, Tokyo 160-8582, Japan

Publisher

Springer Netherlands

Emtree Heading

aged; aging; antibody titer; article; atherosclerotic plaque; body mass; cardiovascular disease; *carotid atherosclerosis; chronic kidney disease; cognition; *cognitive defect; cohort analysis; controlled study; cross- sectional study; *cytomegalovirus infection; daily life activity; diabetes mellitus; female; glomerulus filtration rate; grip strength; human; hypertension; Japan; longitudinal study; major clinical study; male; Mini Mental State Examination; prospective study; risk factor; very elderly; Cytomegalovirus antibody/ec [Endogenous Compound]; high density lipoprotein cholesterol/ec [Endogenous Compound]; immunoglobulin G antibody/ec [Endogenous Compound]; interleukin 6/ec [Endogenous Compound]; low density lipoprotein cholesterol/ec [Endogenous Compound]; Tumor necrosis factor alpha/ec [Endogenous Compound].

Drug Index Terms

Cytomegalovirus antibody / endogenous compound; high density lipoprotein cholesterol / endogenous compound; immunoglobulin G antibody / endogenous compound; interleukin 6 / endogenous compound; low density lipoprotein cholesterol / endogenous compound; tumor necrosis factor alpha / endogenous compound.

Other Index Terms

aged; aging; antibody titer; Article; atherosclerotic plaque; body mass; cardiovascular disease; *carotid atherosclerosis; chronic kidney disease; cognition; *cognitive defect; cohort analysis; controlled study; cross-sectional study; *cytomegalovirus infection; daily life activity; diabetes mellitus; female; glomerulus filtration rate; grip strength; human; hypertension; Japan; longitudinal study; major clinical study; male; Mini Mental State Examination; prospective study; risk factor; very elderly.

Link to the Ovid Full Text or citation:

Click here for full text options

Link to the External Link Resolver:

SFX

For peer review only

112.

Classification of Symptomatic and Asymptomatic Patients with and without Cognitive Decline Using Non-invasive Carotid Plaque Strain Indices as Biomarkers.

Wang X., Jackson D.C., Mitchell C.C., Varghese T., Wilbrand S.M., Rocque B.G., Hermann B.P., Dempsey R.J.

Ultrasound in Medicine and Biology. 42 (4) (pp 909-918), 2016. Date of Publication: 01 Apr 2016.

AN: 607555070

Vascular cognitive decline may be caused by micro-emboli generated by carotid plaque instability. We previously found that maximum strain indices in carotid plaque were significantly correlated with cognitive function. In the work described here, we examined these associations with a larger sample size, as well as evaluated the performance of these maximum strain indices in predicting cognitive impairment. Ultrasound-based strain imaging and cognition assessment were conducted on 75 human patients. Patients underwent one of two standardized cognitive test batteries, either the Repeatable Battery for the Assessment of Neuropsychological Status (RBANS) or the National Institute of Neurologic Disorder and Stroke-Canadian Stroke Network (NINDS-CSN) Vascular Cognitive Impairment Harmonization Standards (60 min). Scores were standardized within each battery to allow these data to be combined across all participants. Radiofrequency signals for ultrasound strain imaging were acquired on the carotid arteries using either a Siemens Antares with a VFX 13-5 linear array transducer or a Siemens S2000 with an 18 L6 linear array transducer. The same hierarchical block-matching motion tracking algorithm developed in our laboratory was used to estimate accumulated axial, lateral, and shear strain indices in carotid plaque, with inclusion of adventitia regardless of the ultrasound system and transducer used. Associations between cognitive z-scores and maximum strain indices were examined using Pearson's correlation coefficients. Maximum strain indices were also employed to predict cognitive impairment using receiver operating characteristic analysis. All correlations between maximum strain indices and total cognition were statistically significant ($p < 0.05$), indicating that these indices have good utility in predicting cognitive impairment. Maximum lateral strain indices provided an area under the curve of 0.85 for symptomatic patients and 0.68 for asymptomatic patients. Our results indicate the important relationship of maximum strain indices to cognitive function and the feasibility of using maximum strain indices to predict cognitive decline with inclusion of the adventitia layer into the segmentation of plaque.

Copyright © 2016 World Federation for Ultrasound in Medicine & Biology.

Institution

(Wang, Varghese) Department of Medical Physics, University of Wisconsin School of Medicine and Public Health, University of Wisconsin-Madison, Madison, WI, United States (Jackson, Hermann)

Department of Neurology, University of Wisconsin School of Medicine and Public Health, University of Wisconsin-Madison, Madison, WI, United States
(Mitchell) Department of Medicine, University of Wisconsin School of Medicine and Public Health, University of Wisconsin-Madison, Madison, WI, United States
(Wilbrand, Rocque, Dempsey) Department of Neurological Surgery, University of Wisconsin School of Medicine and Public Health, University of Wisconsin-Madison, Madison, WI, United States
Publisher
Elsevier USA
Emtree Heading
adventitia; article; *carotid atherosclerosis/di [Diagnosis]; cognition assessment; *cognitive defect; controlled study; disease association; disease classification; *echography; female; human; image analysis; image display; major clinical study; male; priority journal.
Other Index Terms
adventitia; Article; *carotid atherosclerosis / *diagnosis; cognition assessment; *cognitive defect; controlled study; disease association; disease classification; *echography; female; human; image analysis; image display; major clinical study; male; priority journal.

Link to the Ovid Full Text or citation:
Click here for full text options

Link to the External Link Resolver:
SFX

115.

Improved Correlation of Strain Indices with Cognitive Dysfunction with Inclusion of Adventitial Layer with Carotid Plaque.

Wang X., Mitchell C.C., Varghese T., Jackson D.C., Rocque B.G., Hermann B.P., Dempsey R.J.
Ultrasonic imaging. 38 (3) (pp 194-208), 2016. Date of Publication: 01 May 2016.
AN: 615862406
Plaque instability may lead to chronic embolization, which in turn may contribute to progressive cognitive decline. Accumulated strain tensor indices over a cardiac cycle within a pulsating carotid plaque may be viable biomarkers for the diagnosis of plaque instability. Using plaque-only carotid

For peer review only

artery segmentations, we recently demonstrated that impaired cognitive function correlated significantly with maximum axial and lateral strain indices within a localized region of interest in plaque. Inclusion of the adventitial layer focuses our strain or instability measures on the vessel wall-plaque interface hypothesized to be a region with increased shearing forces and measureable instability. A hierarchical block-matching motion tracking algorithm developed in our laboratory was used to estimate accumulated axial, lateral, and shear strain distribution in plaques identified with the plaque-with-adventitia segmentation. Correlations of strain indices to the Repeatable Battery for the Assessment of Neuropsychological Status Total score were performed and compared with previous results. Overall, correlation coefficients (r) and significance (p) values improved for axial, lateral, and shear strain indices. Shear strain indices, however, demonstrated the largest improvement. The Pearson correlation coefficients for maximum shear strain and cognition improved from the previous plaque-only analyses of -0.432 and -0.345 to -0.795 and -0.717 with the plaque-with-adventitia segmentation for the symptomatic group and for all patients combined, respectively. Our results demonstrate the advantage of including adventitia for ultrasound carotid strain imaging providing improved association to parameters assessing cognitive impairment in patients. This supports theories of the importance of the vessel wall plaque interface in the pathophysiology of embolic disease.

Copyright © The Author(s) 2015.

PMID

26025578 [http://www.ncbi.nlm.nih.gov/pubmed/?term=26025578]

Institution

(Wang, Varghese) Department of Medical Physics, University of Wisconsin-Madison School of Medicine and Public Health, Madison, WI, USA (Mitchell) Department of Medicine, University of Wisconsin-Madison, Madison School of Medicine and Public Health, WI, USA

(Jackson, Hermann) Department of Neurology, University of Wisconsin-Madison, Madison School of Medicine and Public Health, WI, USA

(Rocque, Dempsey) Department of Neurological Surgery, University of Wisconsin-Madison School of Medicine and Public Health, Madison, WI, USA

Emtree Heading

adult; adventitia; aged; carotid artery obstruction; computer assisted diagnosis; *diagnostic imaging; elastography; female; human; male; middle aged; neuropsychological test; *procedures; Cognitive Dysfunction/et [Etiology].

Candidate Terms

Cognitive Dysfunction / etiology [other term].

Other Index Terms

adult; adventitia; aged; carotid artery obstruction; computer assisted diagnosis; *diagnostic imaging; elastography; female; human; male; middle aged; neuropsychological test; *procedures.

For peer review only - http://bmjopen.bmj.com/site/about/guidelines.xhtml

Link to the Ovid Full Text or citation:

Click here for full text options

Link to the External Link Resolver:

SFX

116.

Impact of carotid artery revascularization on the cognitive and functional outcome and cerebral flow on TCD and brain MRI in patients with symptomatic carotid artery stenosis: A preliminary report.

Badacz R., Kablak-Ziembicka A., Urbanczyk-Zawadzka M., Banys R.P., Musialek P., Pieniazek P., Mleczko S., Zmudka K., Przewlocki T.

European Heart Journal Cardiovascular Imaging. Conference: 20th Annual Meeting of the European Association of Echocardiography, EUROECHO 2016. Germany. 17 (Supplement 2) (pp ii23), 2016. Date of Publication: December 2016.

AN: 624570711

Background. About one third of patients develop cognitive dementia following cerebral ischemic event (CIE), while 20-50% of subjects with symptomatic carotid artery stenosis (CAS) suffer from CIE recurrence during 6 months. On the other hand, prompt carotid artery revascularization (CAR) may prevent CIE recurrence, however, at the cost of cognitive function decline or new acute micro embolic lesions (MES) on brain diffusion-weighted magnetic resonance imaging (DWI-MRI). We investigated whether CAR in recent survivors of CIE related to high risk CAS may contribute to functional and cognitive outcomes. Methods. Thirteen consecutive patients (mean age 67+/-7.8y.o., 8 male) with recent CIE (18.3+/-10.5 days to CAR) related to severe CAS (mean stenosis degree: 89.8+/-7.9%, range 80-99%) were prospectively assessed with transcranial doppler (TCD) of the Willis Circle, DWI-MRI, cognitive outcome using the Montreal Cognitive Assessment (MoCA), Mini Mental Skills Examination (MMSE), and functional outcome using the modified Rankin Scale (Rs) and National Institutes of Health Stroke Scale (NIHSS) at 24 hours before CAR, at 48-72 hours and at 1 month following CAR. Results. Most plaques were high risk: thrombotic in 5, lipid-rich in 1, lipid-fibrotic in 5 and calcified in 2 patients, including string stenosis in 5 and ulcerations in 9 subjects, as evidenced by ultrasonography and post-CAR histological assessment of plaque debris

For peer review only - http://bmjopen.bmj.com/site/about/guidelines.xhtml

captured by the filter in 9 subjects or plaques removed during endarterectomy in 4 subjects. One (7.7%) minor stroke was observed following CAR. Acute and subacute multiple cerebral ischemic lesions were observed in all subjects before CAR (mean lesion size of 13.5+/-10mm, range 4-37mm), while new MES following CAR were found in 5 (38.5%) subjects. After 1 month, MES persisted in 3 (23%), resolved in 2 (15.4%), decreased in 8 (61.5%) patients respectively. There was a significant increase of cerebral flow velocity in the middle and the anterior cerebral artery on the site of CAR following intervention (from 72+/-20 to 106+/-24cm/s, p=0.0008 and from 75+/-23.4 to 94+/-18.2 cm/s, p=0.056 respectively). This flow increase was correlated with cerebral perfusion increase on MRI. NIHSS and Rs significantly improved after CAR (from 3.0+/-1.2 to 1.8+/-1.1 p=0.013 and from 1.5+/-0.8 to 0.7+/-0.9 p=0.034 respectively). There was no cognitive decline as assessed by MMSE and MoCA at 1 month vs before procedure (MMSE: 27.7+/-2.5, range 22-30, vs 26.8+/-1.9, range 26-29, p=0.027 MoCa: 23.5+/-3.5, range 19-28 vs, 23.3+/-2.5, range 20-27, p=0.356). Improvement of cognitive function was found in 9 (69.2%) by MMSE and in 7 (53.8%) subjects by MoCa. Conclusions. This preliminary pilot study concerning many aspects of CAR following CIE indicated immediate improvement of cerebral flow seen on TCD and perfusion MRI. Furthermore, we did not find cognitive decline when urgent CAR was performed for secondary stroke prevention in this high risk group. Large studies are necessary.

Institution

(Badacz, Kablak-Ziembicka, Pieniazek, Mleczko, Zmudka, Przewlocki) Jagiellonian University Medical College, Department of Interventional Cardiology, Krakow, Poland (Urbanczyk-Zawadzka, Banys) John Paul II Hospital, Department of Radiology and Diagnostic Imaging, Krakow, Poland (Musialek) Jagiellonian University Medical College, Department of Cardiac and Vascular Diseases, John Paul II Hospital, Krakow, Poland

Publisher

Oxford University Press

Emtree Heading

aged; *anterior cerebral artery; *artery formation (physiology); brain circulus arteriosus; brain perfusion; *carotid artery obstruction; case report; cerebrovascular accident; clinical article; cognitive defect; *diffusion weighted imaging; endarterectomy; filter; flow rate; high risk population; histopathology; human; ischemia; male; Montreal cognitive assessment; National Institutes of Health Stroke Scale; perfusion weighted imaging; pilot study; prevention; prospective study; Rankin scale; risk assessment; skill; survivor; thrombosis; transcranial doppler; ulcer; lipid; conference abstract.

Candidate Terms

conference abstract [other term].

Drug Index Terms

lipid.

For peer review only - http://bmjopen.bmj.com/site/about/guidelines.xhtml

Other Index Terms

aged; *anterior cerebral artery; *artery formation (physiology); brain circulus arteriosus; brain perfusion; *carotid artery obstruction; case report; cerebrovascular accident; clinical article; cognitive defect; *diffusion weighted imaging; endarterectomy; filter; flow rate; high risk population; histopathology; human; ischemia; male; Montreal cognitive assessment; National Institutes of Health Stroke Scale; perfusion weighted imaging; pilot study; prevention; prospective study; Rankin scale; risk assessment; skill; survivor; thrombosis; transcranial doppler; ulcer.

Link to the Ovid Full Text or citation:

Click here for full text options

Link to the External Link Resolver:

SFX

120.

Cerebral perfusion and cognitive functions in patients after carotid endarterectomy for symptomatic carotid stenosis.

Mushba A., Vinogradov O., Tsvetkova A., Kuznetsov A.

European Stroke Journal. Conference: 2nd European Stroke Organisation Conference, ESOC 2016. Spain. 1 (1 Supplement 1) (pp 519-520), 2016. Date of Publication: May 2016.

AN: 616987885

Background: 25% of ischemic strokes are associated with atherosclerosis of extracranial and intracranial arteries. Carotid endarterectomy (CEA) is used for secondary prophylaxis of ischemic strokes in patients with symptomatic stenosis of internal carotid artery over 70%. Nevertheless, it is still unknown whether the removal of atherosclerotic plaque from internal carotid artery (ICA) improves perfusion of hemisphere and, as a consequence, cognitive function, or not.

Method(s): 30 patients with atherotrombotic type of ischemic stroke who undergone CEA were included in study. 22 (73.3%) of them were male and 8 (26.7%) were women; mean age of patients was 62.5 +/- 1.3 years. Preoperatively and 10-14 days postoperatively patients were studied with cognitive impairment scales (MMSE, MoCa), cognitive evoked potentials (P-300), duplex scanning of brachiocephalic arteries, transcranial duplex scan, brain MRI and Single-photon emission computed tomography (SPECT)with 99mTc-HMPAO.

For peer review only

Result(s): Improvement of cerebral perfusion in affected hemisphere after CEA was demonstrated: SPECT showed increasing of perfusion in medial cerebral artery system from 87.3% to 92.1% (o < 0.05). Improvement of cerebral perfusion did not result to improvement of cognitive functions: mean MMSE score pre-op and post-op were 25.1 +/-0.5 and 25.9 +/-0.2, respectively (p > 0.05); MoCa score pre-op and post-op were 26.3 +/- 0.4 and 26.7 +/-0.3, respectively (p > 0.05). Differences in neuro-funcional data pre- and postoperatively were not statistically significant. Cognitive evoked potentials showed pre-op and post-op latency of P-300 399.7 +/-8.7 msec and 360.3 +/- 9.6 MC msec, respectively (p > 0.05).

Conclusion(s): CEA for symptomatic stenosis of ICA improves cerebral perfusion in affected hemisphere but do not improve cognitive functions.

Institution

(Mushba) National Pirogov Centre of Therapy and Surgery, Moscow, Russian Federation

(Vinogradov, Tsvetkova, Kuznetsov) National Pirogov Centre of Therapy and Surgery, National Center of Cerebrovascular Disorders, Moscow, Russian Federation

Publisher

SAGE Publications Ltd

Emtree Heading

adult; brachiocephalic trunk; brain ischemia; *brain perfusion; *carotid artery obstruction; *carotid endarterectomy; clinical article; *cognitive defect; evoked response; female; human; internal carotid artery; male; middle aged; middle cerebral artery; Mini Mental State Examination; nuclear magnetic resonance imaging; single photon emission computed tomography; hexamethylpropylene amine oxime technetium tc 99m; polyacrylamide.

Drug Index Terms

hexamethylpropylene amine oxime technetium tc 99m; polyacrylamide.

Other Index Terms

adult; brachiocephalic trunk; brain ischemia; *brain perfusion; *carotid artery obstruction; *carotid endarterectomy; clinical article; *cognitive defect; evoked response; female; human; internal carotid artery; male; middle aged; middle cerebral artery; Mini Mental State Examination; nuclear magnetic resonance imaging; single photon emission computed tomography.

Link to the Ovid Full Text or citation:

Click here for full text options

Link to the External Link Resolver:

SFX

For peer review only - http://bmjopen.bmj.com/site/about/guidelines.xhtml

122.

Lower carotid flow velocities were associated with impaired cognitive function in a community-based elderly population.

Chuang S.-Y., Cheng H.-M., Hwang A.-C., Chen L.-K., Chen C.-H., Wang P.N.

European Heart Journal. Conference: European Society of Cardiology, ESC Congress 2016. Italy. 37 (Supplement 1) (pp 740), 2016. Date of Publication: August 2016.

AN: 612283991

Background: Carotid atherosclerosis (high intima-media thickness) was recognized to associate with stroke and cognitive function impairment. However, few studies investigated the association between carotid flow velocities and cognitive function. This study investigated the association between carotid flow velocities and cognitive function.

Material(s) and Method(s): A total of 1684 seniors aged more than 65 years and without dementia was recruited for this aging study. All seniors were receiving the physical and mental examinations and drawing fasting blood sample for testing biochemical markers. The Mini-Mental State Examination (MMSE) was used to evaluate the global cognitive function. We classified the subjects with poor (MMSE<24), normal (MMSE: 24-27) and well (MMSE: >=28) cognitive function. We used the linear regression and logistic regression to evaluate the association between carotid flow velocity and cognitive function. Multivariate linear regression and ordinal logistic regression were used to evaluate the association between carotid flow velocities and cognitive function.

Result(s): Old age, women gender, low education and high systolic blood pressure, poor nutritional status, worse glucose control and inflammation status were associated with cognitive function. We also found that the peak systolic velocity (PSV) in common carotid artery and in internal carotid artery (ICA), and end-diastolic velocity in the ICA were positively associated with well cognitive function. The multivariate linear regression showed low systolic blood pressure (beta=-0.010, pvalue= 0.0145) and high CCA PSV (beta = 0.026, p-value=0.026, p-value = 0.014) were independently associated with higher MMSE, after controlling the age, sex, education, nutritional status and smoking. Compared to the referent group with higher CCA-PSV (>=68 cm/sec), the group with lowest CCA-PSV (<60 cm/sec) significantly increased 54% risk (OR=1.54; 95% CI: 1.14-2.08), and those seniors with middle CCA-PWV slightly increased 27% risk (OR=1.28; 95% CI: 0.998- 1.63) for impaired cognitive function in the multivariate logistic regression.

Conclusion(s): Low carotid flow velocities were significantly associated with impaired cognitive function and this relationship needs further prospective studies to confirm.

Institution

For peer review only - http://bmjopen.bmj.com/site/about/guidelines.xhtml

(Chuang) National Health Research Institutes, Miaoli County, Taiwan (Republic of China) (Cheng, Hwang, Chen) Taipei Veterans General Hospital, Taipei, Taiwan (Republic of China)
(Chen, Wang) National Yang Ming University, Taipei, Taiwan (Republic of China)
Publisher
Oxford University Press
Emtree Heading
aged; aging; blood glucose monitoring; *carotid artery flow; *cognition; common carotid artery; controlled study; dementia; diet restriction; drawing; female; *flow rate; gender; human; inflammation; internal carotid artery; linear regression analysis; logistic regression analysis; male; Mini Mental State Examination; nutritional status; peak systolic velocity; prospective study; sexual education; smoking; statistical significance; systolic blood pressure.
Other Index Terms
aged; aging; blood glucose monitoring; *carotid artery flow; *cognition; common carotid artery; controlled study; dementia; diet restriction; drawing; female; *flow rate; gender; human; inflammation; internal carotid artery; linear regression analysis; logistic regression analysis; male; Mini Mental State Examination; nutritional status; peak systolic velocity; prospective study; sexual education; smoking; statistical significance; systolic blood pressure.

Link to the Ovid Full Text or citation:
Click here for full text options

Link to the External Link Resolver:
SFX

123.

Carotid intima-media thickness, plaque, and cognition: The Northern manhattan study.
Caunca M.R., Gardener H., Dong C., Gervasi-Franklin P., Cheung Y.K., Elkind M.S.V., Sacco R.L., Rundek T., Wright C.B.
Annals of Neurology. Conference: 141st Annual Meeting of the American Neurological Association, ANA 2016. United States. 80 (Supplement 20) (pp S168-S169), 2016. Date of Publication: October 2016.
AN: 612892399

For peer review only

Carotid artery intima media thickness (cIMT) and nonstenotic carotid plaque are suggested markers of carotid atherosclerosis and may be related to cognition in the elderly. We hypothesized that individuals with greater cIMT or with carotid plaques would exhibit worse cognition at baseline and have greater cognitive decline. Stroke-free Northern Manhattan Study (NOMAS) participants had carotid ultrasound and repeated neuropsychological (NP) testing. Carotid IMT and plaques were imaged with standardized B-mode ultrasound protocols and analyzed by a certified sonographer. We used multivariable linear regression to examine cIMT, plaque presence, and plaque area as correlates of domain-specific NP Zscores cross-sectionally and after six years follow-up. We also investigated effect modification by APOE e4 allele status. Neuropsychological testing was performed among 1166 participants at baseline and among 826 participants at follow-up (mean=6.2 years). The mean cIMT was 0.93+/-0.09 mm (mean age= 71+/-9 years; 60% women; 15% white; 18% black; 67% Hispanic white). Participants with greater cIMT had worse episodic memory at baseline after adjustment for demographics and vascular risk factors (beta=-0.60, P=0.04). APOE e4 carriers with greater cIMT exhibited worse episodic memory (beta=-1.26, P=0.04), semantic memory (beta=-1.35, P=0.01), and processing speed (beta=-1.22, P=0.02) at baseline. Participants with greater cIMT at baseline did not exhibit cognitive decline in episodic or semantic memory, but did exhibit decline in executive function and processing speed, though these associations did not reach significance. The APOE e4 allele was a significant effect modifier: participants without an APOE e4 allele who had greater cIMT at baseline exhibited more decline in executive function (beta=-1.03, P=0.05). Neither plaque presence nor area was significantly associated with cognitive performance in any domain in cross-sectional or longitudinal analyses. Results remained similar after restricting the analysis to those who were categorized as cognitively unimpaired at baseline based on their Mini-Mental Status Examination score. Our cross-sectional findings in this race/ethnically diverse community-based urban sample suggest that being at elevated genetic risk of Alzheimer disease as well as having a greater vascular disease burden may have cognitive consequences. A greater burden of vascular disease may have domain-specific cognitive consequences in the absence of genetic Alzheimer disease risk. Atherosclerotic lesions may be less important in the pathology of vascular cognitive changes, but larger studies are needed. Arterial wall thickening due to compensatory and inflammatory arterial remodeling are mechanisms that should be explored in future studies.

Institution

(Caunca, Gardener, Dong, Gervasi-Franklin, Cheung, Elkind, Sacco, Rundek, Wright) Miami, FL and New York, NY

Publisher

John Wiley and Sons Inc.

Emtree Heading

For peer review only

aged; Alzheimer disease; arterial wall thickening; *arterial wall thickness; atherosclerosis; cardiovascular risk; cerebrovascular accident; cognitive defect; episodic memory; *executive function; female; follow up; gene frequency; genetic predisposition; genetic risk; Hispanic; human; linear regression analysis; major clinical study; male; mental health; neuropsychological test; race; semantic memory; ultrasound; vascular disease; velocity; apolipoprotein E; endogenous compound.

Drug Index Terms

apolipoprotein E; endogenous compound.

Other Index Terms

aged; Alzheimer disease; arterial wall thickening; *arterial wall thickness; atherosclerosis; cardiovascular risk; cerebrovascular accident; cognitive defect; episodic memory; *executive function; female; follow up; gene frequency; genetic predisposition; genetic risk; Hispanic; human; linear regression analysis; major clinical study; male; mental health; neuropsychological test; race; semantic memory; ultrasound; vascular disease; velocity.

Link to the Ovid Full Text or citation:

Click here for full text options

Link to the External Link Resolver:

SFX

125.

Subclinical carotid atherosclerosis associates with impairment in immediate memory.

Matsumoto L., Suzuki K., Mizuno Y., Ohike Y., Ozeki A., Ono S., Takanashi M., Sawaki D., Suzuki T., Yamazaki T., Tsuji S., Iwata A.

Neurology. Conference: 68th American Academy of Neurology Annual Meeting, AAN 2016. Vancouver, BC Canada. Conference Publication: (var.pagings). 86 (16 SUPPL. 1) (no pagination), 2016. Date of Publication: 05 Apr 2016.

AN: 72252214

Objective: To clarify whether carotid atherosclerosis and its risk factors contribute to cognitive function.

For peer review only

Background(s): Both of two major types of dementia: vascular dementia and Alzheimers disease shares pathogenesis related to atherosclerosis. Carotid intima-media thickness (IMT) is widely used for assessing atherosclerosis. Some study has reported association of increased carotid IMT and cognitive function, but it remains controversial.
Method(s): Two hundred and six individuals who visited our center for health screening were evaluated with physical examination, blood test, carotid ultrasonography, brain MRI scanning, and cognitive function tests. Cognitive function assessment included Mini-Mental State Examination (MMSE), Clock Drawing Test (CDT), and logical memory of Wechsler Memory Scale (WMS-R). Thirty individuals with cerebrovascular lesions by MRI scanning were excluded. To detect early cognitive decline, we defined "cognitive impairment (CI)" when an individual satisfied at least one of three criteria; 1)MMSE score under 24, 2)CDT score under 4 coexistent with forgetfulness, and 3)WMS-R delayed recall score under normal range for each educational length (over 16 years: 9, 10-15 years: 5, 0-9 years: 3), according to Alzheimer's Disease Neuroimaging Initiative (ADNI) late MCI criteria.
Result(s): Among 176 individuals, 27 were classified as CI group. Maximum IMT was significantly increased in CI group than in non-CI group (meanSD: 2.01.0 vs 1.70.7, p=0.018) by student f-test. Other atherosclerotic risk factors; blood pressure, LDL-cholesterol, and HbA1c were not significantly different between two groups. In multivariate analysis, no single risk factor of atherosclerosis contributed to each cognitive function assessment. However, carotid IMT was associated with impaired immediate recall score of WMS-R independently to deep white matter hyperintensity on MRI scan.
Conclusion(s): Subclinical carotid atherosclerosis assessed as thickened IMT can be a marker of early stage of cognitive impairment especially immediate memory recall presumably by cerebral microvascular dysfunction in frontal lobe.
Publisher
Lippincott Williams and Wilkins
Emtree Heading
*carotid atherosclerosis; *short term memory; *American; *neurology; Wechsler memory scale; cognition; atherosclerosis; carotid artery; recall; risk factor; multiinfarct dementia; cognitive defect; human; Alzheimer disease; Mini Mental State Examination; nuclear magnetic resonance imaging; blood; multivariate analysis; physical examination; mass screening; blood pressure; student; neuroimaging; cerebrovascular disease; memory; clock drawing test; echography; arterial wall thickness; white matter; cognitive function test; frontal lobe; brain; pathogenesis; hemoglobin A1c; low density lipoprotein cholesterol; marker; low density lipoprotein.
Drug Index Terms
hemoglobin A1c; low density lipoprotein cholesterol; marker; low density lipoprotein.
Other Index Terms

For peer review only

*carotid atherosclerosis; *short term memory; *American; *neurology; Wechsler memory scale; cognition; atherosclerosis; carotid artery; recall; risk factor; multiinfarct dementia; cognitive defect; human; Alzheimer disease; Mini Mental State Examination; nuclear magnetic resonance imaging; blood; multivariate analysis; physical examination; mass screening; blood pressure; student; neuroimaging; cerebrovascular disease; memory; clock drawing test; echography; arterial wall thickness; white matter; cognitive function test; frontal lobe; brain; pathogenesis.

Link to the Ovid Full Text or citation:
Click here for full text options

Link to the External Link Resolver:
SFX

128.

Arterial stiffness and pressure amplification are associated with lower cognition among older Caucasian adults: The atherosclerosis risk in communities (ARIC) study.
Wei J., Palta P., Meyer M., Tanaka H., Deal J., Jack C., Knopman D., Wright J., Griswold M., Mosley T.H., Heiss G.
Circulation. Conference: American Heart Association's Epidemiology and Prevention/Lifestyle and Cardiometabolic Health 2016 Scientific Sessions. Phoenix, AZ United States. Conference Publication: (var.pagings). 133 (SUPPL. 1) (no pagination), 2016. Date of Publication: 01 Mar 2016.
AN: 72231686
Introduction: Accelerated cognitive decline is influenced by vascular aging. Arterial stiffness and pressure amplification, measures of vascular aging, are associated with lower cognitive performance though their association with cognitive domains has been understudied Hypothesis: Arterial stiffness and pressure amplification are associated with lower global and domain-specific cognition among a sample of Caucasian and African American (AA) older adults Methods: In a cross-sectional study of 4618 members from visit 5 (2011-2013) of ARIC (mean age: 75 years, 41% men, 20% AA), we measured arterial stiffness (carotid-femoral pulse wave velocity (cfPWV)) and pressure amplification (pulse pressure amplification (PPA), central pulse pressure (cPP) and carotid systolic blood pressure (cSBP)) using the Omron VP-1000 Plus device. Race-specific 25th percentile cut points were estimated for each measure. Domain-specific cognitive function was examined

For peer review only - http://bmjopen.bmj.com/site/about/guidelines.xhtml

using the Delayed Word Recall Test (memory), Digit Symbol Substitution Test (executive function/processing speed), and Word Fluency Test (language). Test-specific z scores were calculated from sample means and standard deviations. A global cognition z score was generated by averaging the test-specific z scores. Linear regression was used to estimate the associations between race-specific 25th percentile dichotomies for arterial stiffness and pressure amplification measures with test-specific and global cognition z scores, adjusted for age, sex, education, ApoE4, heart rate, smoking and body mass index Results: Among AAs, there was no significant association between measures of arterial stiffness and pressure amplification with either global or the domain-specific measures of cognition. Among Caucasians, all measures of arterial stiffness and pressure amplification were associated with lower global cognitive z scores (cfPWV: Beta (beta) =-0.11, 95% Confidence Interval (CI): -0.19, -0.04; PPA: beta=-0.11, 95% CI: -0.19, -0.03; cPP: beta=-0.11, 95% CI: -0.18, -0.04; cSBP: beta=-0.12, 95% CI: -0.20, -0.05). All measures of arterial stiffness and pressure amplification also were associated with lower executive function/processing speed (cfPWV: beta=-0.09, 95% CI: -0.16, -0.03; PPA: beta=-0.15, 95% CI: -0.22, -0.08; cPP: beta=-0.14, 95% CI: -0.21, -0.07; cSBP: beta=-0.13, 95% CI: -0.20, -0.07). High cfPWV was associated with lower memory (beta=-0.12, 95% CI: -0.20, -0.04); and high cSBP was associated with lower language (beta=-0.12, 95% CI: -0.20, -0.04)

Conclusion(s): Arterial stiffness and measures of pressure amplification are inversely associated with global and domain-specific cognition in Caucasian older adults, but not in a smaller sample of AAs. Further study of the role of modifiable components of arterial aging on the preservation of cognition, particularly executive function/psychomotor speed, in older adulthood is warranted.

Institution

(Wei, Palta, Meyer, Heiss) Univ of North Carolina at Chapel Hill, Chapel Hill, NC, United States
(Tanaka) Univ of Texas at Austin, Austin, TX, United States
(Deal) Johns Hopkins Univ, Baltimore, MD, United States
(Jack, Knopman) Mayo Clinic, Rochester, MN, United States
(Wright) National Heart,Lung,and Blood Institute, Bethesda, MD, United States
(Griswold) Univ of Mississippi Med Cntr, Jackson, MS, United States
(Mosley) Univ of Mississippi, Sch of Medicine, Jackson, MS, United States

Publisher

Lippincott Williams and Wilkins

Emtree Heading

*cognition; *arterial stiffness; *community; *adult; *atherosclerosis; *risk; *Caucasian; *medical society; *epidemiology; *health; human; manager; velocity; aging; pulse pressure; carotid artery; memory; language; digit symbol substitution test; word recognition; male; devices; systolic blood pressure; preservation; cross-sectional study; body mass; smoking; heart rate; African American; sexual education; linear regression analysis; pulse wave; hypothesis; confidence interval; adulthood.

For peer review only - http://bmjopen.bmj.com/site/about/guidelines.xhtml

Other Index Terms
*cognition; *arterial stiffness; *community; *adult; *atherosclerosis; *risk; *Caucasian; *medical society; *epidemiology; *health; human; manager; velocity; aging; pulse pressure; carotid artery; memory; language; digit symbol substitution test; word recognition; male; devices; systolic blood pressure; preservation; cross-sectional study; body mass; smoking; heart rate; African American; sexual education; linear regression analysis; pulse wave; hypothesis; confidence interval; adulthood.

Link to the Ovid Full Text or citation:
Click here for full text options

Link to the External Link Resolver:
SFX

129.

Association of arterial stiffness and pressure amplification with mild cognitive impairment and dementia: The atherosclerosis risk in communities study-neurocognitive study (ARIC-NCS).
Meyer M.L., Palta P., Tanaka H., Deal J.A., Wright J., Jack C., Knopman D., Griswold M., Mosley T.H., Heiss G.H.
Circulation. Conference: American Heart Association's Epidemiology and Prevention/Lifestyle and Cardiometabolic Health 2016 Scientific Sessions. Phoenix, AZ United States. Conference Publication: (var.pagings). 133 (SUPPL. 1) (no pagination), 2016. Date of Publication: 01 Mar 2016.
AN: 72231532
Abstract Background: As a high-flow, low-impedance organ, the brain is sensitive to excessive pressure and flow pulsatility. Increased pulsatility and arterial stiffness are hypothesized to contribute to cerebral microvascular damage linked to cognitive impairment. The association of arterial stiffness with mild cognitive impairment (MCI) and dementia in a biethnic population is not well characterized, and the association of pressure amplification with MCI and dementia is relatively unexplored.
Objective(s): To quantify the cross-sectional association of arterial stiffness, measured by aortic pulse wave velocity (PWV), and pressure amplification with MCI and dementia in a biethnic population of older adults.

For peer review only - http://bmjopen.bmj.com/site/about/guidelines.xhtml

Method(s): We included 4,945 adults (2,903 females; 1,069 African Americans; mean age 75 years) from the population-based ARIC-NCS. The Omron VP-1000 plus system was used to measure arterial stiffness (carotid-femoral PWV (cfPWV)) and pressure amplification measures (central systolic blood pressure (cSBP), central pulse pressure (cPP), and pulse pressure amplification (PPA)). A neurologist and neuropsychologist classified MCI and dementia using psychometric assessments, medical history, cerebral magnetic resonance imaging, and physical examinations, with adjudication by a third reviewer. We used multinomial logistic regression to evaluate associations of race-specific 25th percentile cut points of PWV and pressure amplification with normal cognition (reference), MCI and dementia. We stratified by race and adjusted for age, sex, and heart rate, ApoE4, education, smoking status, and study center.
Result(s): There were 760 Caucasians with MCI and 110 with dementia, and 201 African Americans with MCI and 47 with dementia. Among Caucasians, those with lower PPA had a higher prevalence of dementia, odds ratio (OR)=1.84 (95% confidence interval (CI): 1.15, 2.97), comparing participants below the 25th percentile to those above it, and those with higher cSBP had a higher prevalence of MCI, OR=1.33 (95% CI: 1.09, 1.63), comparing participants above the 75th percentile to those below it. Also among Caucasians, those with higher cPP had a higher prevalence of MCI, OR=1.25 (95%CI: 1.01, 1.55), and dementia, OR=1.66 (95% CI: 1.00, 2.73), comparing participants above the 75th percentile to those below it. There were no statistically significant associations with cfPWV, among African Americans, and no evidence for effect modification by hypertension or diabetes.
Conclusion(s): Arterial stiffness and components of pressure amplification were associated with MCI and dementia in Caucasians but not in African Americans, possibly due to the limited sample size. Longitudinal characterization of the observed associations is warranted to determine whether these measures are independent predictors of MCI and dementia among Caucasian and African American older adults.
Institution
(Meyer, Palta, Heiss) Univ of North Carolina at Chapel Hill, Chapel Hill, NC, United States (Tanaka) Univ of Texas at Austin, Austin, TX, United States
(Deal) Johns Hopkins Univ, Baltimore, MD, United States
(Wright) National Heart Lung and Blood Institute, Bethesda, MD, United States
(Jack, Knopman) Mayo Clinic, Rochester, MN, United States
(Griswold, Mosley) Univ of Mississippi, Med Cntr, Jackson, MS, United States
Publisher
Lippincott Williams and Wilkins
Emtree Heading
*cognitive defect; *pulse wave; *aging; *arterial stiffness; *mild cognitive impairment; *dementia; *atherosclerosis; *risk; *community; *medical society; *epidemiology; *health; human; African American; population; prevalence; adult; pulse pressure; impedance; diabetes mellitus;

For peer review only - http://bmjopen.bmj.com/site/about/guidelines.xhtml

hypertension; systolic blood pressure; sample size; confidence interval; smoking; carotid artery; education; heart rate; cognition; logistic regression analysis; female; physical examination; nuclear magnetic resonance imaging; medical history; neurologist; Caucasian; brain.

Other Index Terms

*cognitive defect; *pulse wave; *aging; *arterial stiffness; *mild cognitive impairment; *dementia; *atherosclerosis; *risk; *community; *medical society; *epidemiology; *health; human; African American; population; prevalence; adult; pulse pressure; impedance; diabetes mellitus; hypertension; systolic blood pressure; sample size; confidence interval; smoking; carotid artery; education; heart rate; cognition; logistic regression analysis; female; physical examination; nuclear magnetic resonance imaging; medical history; neurologist; Caucasian; brain.

Link to the Ovid Full Text or citation:

Click here for full text options

Link to the External Link Resolver:

SFX

130.

Carotid intima-media thickness and cognition: The northern manhattan study.

Caunca M.R., Gardener H., Gervasi-Franklin P., Cheung Y.K., Elkind M.S., Sacco R.L., Rundek T., Wright C.B.

Stroke. Conference: American Heart Association/American Stroke Association 2016 International Stroke Conference and State-of-the-Science Stroke Nursing Symposium. Los Angeles, CA United States. Conference Publication: (var.pagings). 47 (SUPPL. 1) (no pagination), 2016. Date of Publication: February 2016.

AN: 72211108

Background/Objective: Carotid artery intima media thickness (cIMT) may be a marker of cerebral atherosclerotic disease and therefore related to cognitive status in the elderly. We hypothesized that those with greater cIMT would exhibit worse cognition at baseline and have greater cognitive decline over time.

Method(s): A sample of 1166 stroke-free community participants from the Northern Manhattan Study (NOMAS) underwent carotid ultrasound and repeated neuropsychological (NP) testing. cIMT

For peer review only

was imaged with a standardized B-mode ultrasound protocol and analyzed by a trained sonographer. We used multivariable linear regression to examine cIMT as a correlate of domain-specific NP Z-scores cross-sectionally and over time; we investigated possible effect modification by APOE epsilon4 allele, adjusting for demographics and vascular risk factors.

Result(s): The mean age of participants was 71+/-9 years, 60% were women, 15% white, 18% black, 67% Hispanic, and mean cIMT was 0.93 +/- 0.09 mm. NP testing was performed among 1166 participants at baseline and among 826 participants with NP follow-up at a mean of 6.2 years apart. Participants with greater cIMT exhibited worse episodic memory at baseline after adjustment for demographics and vascular risk factors (beta=-0.60, P=0.04). APOE epsilon4 allele presence was a significant effect modifier, and after stratification, APOE epsilon4 carriers with greater cIMT exhibited worse episodic memory (beta=-1.26, P=0.04), semantic memory (beta=-1.35, P=0.01), and processing speed (beta=-1.22, P=0.02) at baseline. In longitudinal analysis, participants with greater cIMT at baseline did not exhibit cognitive decline after adjustment for demographics and vascular risk factors, but APOE epsilon4 allele presence was a significant effect modifier. After stratification, APOE epsilon4 non-carriers with greater cIMT at baseline exhibited greater declines in executive function (beta=-1.03, P=0.05).

Conclusion(s): cIMT may be associated with worse cognition and greater cognitive decline in multiple domains among those at high risk for Alzheimer disease, and with worsening executive function in APOE epsilon4 non-carriers. Interventions that target early stages of atherosclerosis may modify the course of cognitive aging and prevent cognitive decline.

Institution

(Caunca, Gardener) Neurology, Univ of Miami Miller Sch of Medicine, Miami, FL, United States

(Gervasi-Franklin) Neurology, Columbia Univ, New York, NY, United States

(Cheung) Biostatistics, Columbia Univ, New York, NY, United States

(Elkind) Neurology, Epidemiology, Columbia Univ, New York, NY, United States

(Sacco) Neurology, Human Genetics, Univ of Miami Miller Sch of Medicine, Miami, FL, United States

(Rundek) Neurology, Public Health Sciences, Univ of Miami Miller Sch of Medicine, Miami, FL, United States

(Wright) Neurology, Public Health Sciences, Neuroscience, Univ of Miami Miller Sch of Medicine, Miami, FL, United States

Publisher

Lippincott Williams and Wilkins

Emtree Heading

*cerebrovascular accident; *ultrasound; *imaging; *cognition; *arterial wall thickness; *American; *heart; *cognitive defect; *nursing; human; risk factor; allele; carotid artery; stratification; executive function; episodic memory; follow up; aged; artery intima; linear regression analysis; Hispanic;

For peer review only - http://bmjopen.bmj.com/site/about/guidelines.xhtml

Alzheimer disease; risk; female; velocity; processing; semantic memory; community; atherosclerosis; aging; *apolipoprotein; marker.

Drug Index Terms

*apolipoprotein; marker.

Other Index Terms

*cerebrovascular accident; *ultrasound; *imaging; *cognition; *arterial wall thickness; *American; *heart; *cognitive defect; *nursing; human; risk factor; allele; carotid artery; stratification; executive function; episodic memory; follow up; aged; artery intima; linear regression analysis; Hispanic; Alzheimer disease; risk; female; velocity; processing; semantic memory; community; atherosclerosis; aging.

Link to the Ovid Full Text or citation:

Click here for full text options

Link to the External Link Resolver:

SFX

137.

Subclinical carotid artery atherosclerosis and performance on cognitive tests in middle-aged adults: Baseline results from the ELSA-Brasil.

Suemoto C.K., Santos I.S., Bittencourt M.S., Pereira A.C., Goulart A.C., Rundek T., Passos V.M., Lotufo P., Bensenor I.M.

Atherosclerosis. 243 (2) (pp 510-515), 2015. Date of Publication: December 01, 2015.

AN: 606735660

Background and aims: Carotid artery intima-media thickness (CIMT) may be used as a biomarker for early cognitive impairment. However, the results of the association between CIMT and cognitive function in middle-aged subjects are mixed. We aimed to investigate this association in a large Brazilian sample with no history of stroke at baseline. Additionally, we tested the effect of interactions between CIMT and cardiovascular risk factors on cognitive performance.

Method(s): In this cross-sectional study, cognition was evaluated using the delayed word recall (DWRT), the category fluency, and the trail making tests (TMT). CIMT was measured at the common carotid artery. The association between CIMT and cognitive tests was investigated using

For peer review only - http://bmjopen.bmj.com/site/about/guidelines.xhtml

linear regression models, adjusted for an extensive set of possible confounding variables. We also included interaction terms with selected risk factors.

Result(s): The mean age of the 8208 participants was 49.6 +/- 7.3 years, 44% were male, and 56% White. Increase in CIMT was associated with worse performance on the DWRT (beta = -0.433, 95%CI = -0.724;-0.142, p = 0.004). We found effect modification of the association between cognitive function and CMIT by self-reported heart failure and alcohol intake. Participants had worse performance in the TMT if they had greater CIMT and current alcohol use (p < 0.0001). The interaction between CIMT and heart failure on TMT performance was not significant after adjustment for multiple comparisons (p = 0.07).

Conclusion(s): In this sample of middle-aged adults, CIMT was inversely associated with memory function. Additionally, the presence of alcohol use resulted in a stronger association of CIMT with worse performance on an executive function test.

Copyright © 2015 Elsevier Ireland Ltd.

PMID

26520907 [http://www.ncbi.nlm.nih.gov/pubmed/?term=26520907]

Author NameID

Suemoto, Claudia K.; ORCID: http://orcid.org/0000-0002-5942-4778 Passos, Valeria M.; ORCID: http://orcid.org/0000-0003-2829-5798

Institution

(Suemoto) Division of Geriatrics, University of Sao Paulo Medical School, Sao Paulo, Brazil

(Suemoto) Department of Global Health and Population, Harvard School of Public Health, Boston, United States

(Santos, Lotufo, Bensenor) Department of Internal Medicine, University of Sao Paulo Medical School, Sao Paulo, Brazil

(Santos, Bittencourt, Goulart, Lotufo, Bensenor) Center for Clinical and Epidemiological Research, Hospital Universitario, University of Sao Paulo, Sao Paulo, Brazil

(Pereira) Laboratory of Genetics and Molecular Cardiology, Division of Cardiology, University of Sao Paulo Medical School, Sao Paulo, Brazil

(Rundek) Departments of Neurology and Public Health Sciences, University of Miami, Miller School of Medicine, Miami, FL, United States

(Passos) Department of Internal Medicine, Federal University of Minas Gerais Medical School, Minas Gerais, Brazil

Publisher

Elsevier Ireland Ltd

Emtree Heading

adult; alcohol consumption; arterial wall thickness; article; Brazilian; cardiovascular risk; carotid artery; *carotid atherosclerosis; cerebrovascular accident; *cognition; cognition assessment;

For peer review only - http://bmjopen.bmj.com/site/about/guidelines.xhtml

common carotid artery; executive function test; female; heart failure; human; linear regression analysis; major clinical study; male; memory; middle aged; priority journal; self report; trail making test; delayed word recall test.

Candidate Terms

delayed word recall test [other term].

Other Index Terms

adult; alcohol consumption; arterial wall thickness; Article; Brazilian; cardiovascular risk; carotid artery; *carotid atherosclerosis; cerebrovascular accident; *cognition; cognition assessment; common carotid artery; executive function test; female; heart failure; human; linear regression analysis; major clinical study; male; memory; middle aged; priority journal; self report; trail making test.

Link to the Ovid Full Text or citation:

Click here for full text options

Link to the External Link Resolver:

SFX

139.

Differential influence of carotid stenosis and white matter disease on motor and cognitive activation.

Polidori M.C., Calistri V., Mainero C., Tinelli E., Aceti A., Pontico M., Tardioli S., Santini M., Fiorelli M., Panico M.A., Speziale F., Caramia M.D., Schulz R.-J., Caramia F.

Current Alzheimer Research. 12 (6) (pp 585-591), 2015. Date of Publication: 01 Jul 2015.

AN: 605531948

Background: Cognitive and motor performance can be supported, especially in older subjects, by different types of brain activations, which can be accurately studied by functional magnetic resonance imaging (fMRI). Vascular risk factors (VRFs) are extremely important in the development of cognitive impairment, but few studies have focused on the fMRI cortical activation characteristics of healthy subjects with and without silent cerebrovascular disease including white matter hyperintensities (WMH) and carotid stenosis (CS) performing cognitive tasks.

For peer review only

Method(s): Thirty-five volunteers with and without asymptomatic unilateral carotid stenosis above 70% and variable degrees of WMH underwent performance of a simple motor and cognitive task during an fMRI session.
Result(s): While the performance of the motor task resulted in a cortical activation dependent of age but not of WMH and carotid stenosis, performance of the cognitive task was accompanied by a significantly increased activation independently correlated with age, presence of WMH as well as of carotid stenosis.
Conclusion(s): in this study, cognitive domains regulating attention and working memory appear to be activated with a pattern influenced by the presence of carotid stenosis as well as by white matter hyperintensities. The impairment of these cognitive abilities is of high relevance in Alzheimer's disease pathology. The fMRI pattern shown in patients with asymptomatic but significant carotid stenosis might be related to chronic cerebrovascular hypoperfusion, a critical pathophysiological mechanisms in AD. In these patients, carotid endoarterectomy should be considered also for AD prevention and might be recommended.
Copyright © 2015 Bentham Science Publishers.
Institution
(Polidori) Geriatrics Department, St. Marienhospital Cologne and University of Cologne, Cologne, Germany (Calistri, Tinelli, Aceti, Pontico, Tardioli, Santini, Fiorelli, Caramia) Department of Neurology and Psychiatry, University La Sapienza, Rome, Italy
(Mainero) Athinoula A. Martinos Center for Biomedical Imaging, Massachusetts General Hospital, Charlestown, MA, United States
(Panico, Speziale) Vascular and Endovascular Surgery Division, Department of Surgery "Paride Stefanini", Policlinico Umberto I, Sapienza University of Rome, Italy
(Caramia) Department of Public Health, University Tor Vergata, Rome, Italy
(Schulz) Department of Geriatric Medicine, St- Marien- Hospital, Cologne, Germany
Publisher
Bentham Science Publishers B.V. (P.O. Box 294, Bussum 1400 AG, Netherlands)
Emtree Heading
adult; age; Alzheimer disease/et [Etiology]; article; attention; *carotid artery obstruction; clinical article; *cognition; continuous performance test; echo planar imaging; female; functional magnetic resonance imaging; functional neuroimaging; human; male; Mini Mental State Examination; *motor performance; nuclear magnetic resonance scanner; pathophysiology; priority journal; task performance; *white matter lesion; working memory.
Other Index Terms
adult; age; Alzheimer disease / etiology; Article; attention; *carotid artery obstruction; clinical article; *cognition; continuous performance test; echo planar imaging; female; functional magnetic resonance imaging; functional neuroimaging; human; male; Mini Mental State Examination; *motor

For peer review only - http://bmjopen.bmj.com/site/about/guidelines.xhtml

performance; nuclear magnetic resonance scanner; pathophysiology; priority journal; task performance; *white matter lesion; working memory.

Link to the Ovid Full Text or citation:
Click here for full text options

Link to the External Link Resolver:
SFX

141.

Atherosclerotic calcification is related to a higher risk of dementia and cognitive decline.
Bos D., Vernooij M.W., De Bruijn R.F.A.G., Koudstaal P.J., Hofman A., Franco O.H., Van Der Lugt A., Ikram M.A.
Alzheimer's and Dementia. 11 (6) (pp 639-647.e1), 2015. Date of Publication: 01 Jun 2015.
AN: 53298505
Background: Longitudinal data on the role of atherosclerosis in different vessel beds in the etiology of cognitive impairment and dementia are scarce and inconsistent.
Method(s): Between 2003-2006, 2364 nondemented persons underwent computed tomography of the coronaries, aortic arch, extracranial, and intracranial carotid arteries to quantify atherosclerotic calcification. Participants were followed for incident dementia (n = 90) until April 2012. At baseline and follow-up participants also underwent a cognitive test battery.
Result(s): Larger calcification volume in all vessels, except in the coronaries, was associated with a higher risk of dementia. After adjustment for relevant confounders, extracranial carotid artery calcification remained significantly associated with a higher risk of dementia [hazard ratio per standard deviation increase in calcification volume: 1.37 (1.05, 1.79)]. Additional analyses for Alzheimer's disease only or censoring for stroke showed similar results. Larger calcification volumes were also associated with cognitive decline.
Conclusion(s): Atherosclerosis, in particular in the extracranial carotid arteries, is related to a higher risk of dementia and cognitive decline.
Copyright © 2015 The Alzheimer's Association. Published by Elsevier Inc. All rights reserved.
Institution

For peer review only - http://bmjopen.bmj.com/site/about/guidelines.xhtml

(Bos, Vernooij, Van Der Lugt, Ikram) Department of Radiology, Erasmus Medical Center, Rotterdam, Netherlands (Bos, Vernooij, De Bruijn, Hofman, Franco, Ikram) Department of Epidemiology, Erasmus Medical Center, Rotterdam, Netherlands
(De Bruijn, Koudstaal, Ikram) Department of Neurology, Erasmus Medical Center, Rotterdam, Netherlands
Publisher
Elsevier Inc. (E-mail: usjcs@elsevier.com)
Emtree Heading
aged; Alzheimer disease; aorta arch; aorta atherosclerosis; *artery calcification; article; *atherosclerosis; carotid atherosclerosis; cerebrovascular accident; cognition assessment; *cognitive defect; computer assisted tomography; coronary artery atherosclerosis; coronary artery calcification; *dementia; disease assessment; female; follow up; human; internal carotid artery; left coronary artery; major clinical study; male; priority journal; *risk factor; carotid artery calcification.
Candidate Terms
carotid artery calcification [other term].
Other Index Terms
aged; Alzheimer disease; aorta arch; aorta atherosclerosis; *artery calcification; Article; *atherosclerosis; carotid atherosclerosis; cerebrovascular accident; cognition assessment; *cognitive defect; computer assisted tomography; coronary artery atherosclerosis; coronary artery calcification; *dementia; disease assessment; female; follow up; human; internal carotid artery; left coronary artery; major clinical study; male; priority journal; *risk factor.

Link to the Ovid Full Text or citation:
Click here for full text options

Link to the External Link Resolver:
SFX

143.

Intima-Media Thickness and Cognitive Function in Stroke-Free Middle-Aged Adults: Findings From the Coronary Artery Risk Development in Young Adults Study.
Zeki Al Hazzouri A., Vittinghoff E., Sidney S., Reis J.P., Jacobs D.R., Yaffe K.

For peer review only - http://bmjopen.bmj.com/site/about/guidelines.xhtml

Stroke; a journal of cerebral circulation. 46 (8) (pp 2190-2196), 2015. Date of Publication: 01 Aug 2015.

AN: 609379497

BACKGROUND AND PURPOSE: The relationship between carotid artery intima-media thickness (IMT) and cognitive function in midlife remains relatively unexplored. We examined the association between IMT and cognitive function in a middle-aged epidemiological cohort of 2618 stroke-free participants. METHODS: At the year 20 visit (our study baseline), participants from the Coronary Artery Risk Development in Young Adults study had IMT measured by ultrasound at the common carotid artery. Five years later, participants completed a cognitive battery consisting of the Rey Auditory-Verbal Learning Test of verbal memory, the Digit Symbol Substitution Test of processing speed, and the Stroop test of executive function. We transformed cognitive scores into standardized z scores, with negative values indicating worse performance.

RESULTS: Mean age at baseline was 45.3 years (SD, 3.6). Greater IMT (per 1 SD difference of 0.12 mm) was significantly associated with worse performance on all cognitive tests (z scores) in unadjusted linear regression models (verbal memory, -0.16; 95% confidence interval [CI], -0.20 to -0.13; processing speed, -0.23; 95% CI, -0.27 to -0.19; and executive function, -0.17; 95% CI, -0.20 to -0.13). In models adjusted for sociodemographics and vascular risk factors that lie earlier in the causal pathway, greater IMT remained negatively associated with processing speed (-0.06; 95% CI, -0.09 to -0.02; P, 0.003) and borderline associated with executive function (-0.03; 95% CI, -0.07 to 0.00; P, 0.07) but not with verbal memory.

CONCLUSIONS: We observed an association between greater IMT and worse processing speed-a key component of cognitive functioning-at middle age above and beyond traditional vascular risk factors. Efforts targeted at preventing early stages of atherosclerosis may modify the course of cognitive aging.

Copyright © 2015 American Heart Association, Inc.

PMID

26106116 [http://www.ncbi.nlm.nih.gov/pubmed/?term=26106116]

Institution

(Zeki Al Hazzouri) From the Division of Epidemiology and Population Health Sciences, Department of Public Health Sciences, University of Miami, FL (A.Z.A.H.); Departments of Epidemiology and Biostatistics (E.V., K.Y.), Psychiatry (K.Y.), and Neurology (K.Y.), University of California San Francisco; San Francisco Veterans Affairs Medical Center, San Francisco, CA (K.Y.); Kaiser Permanente Division of Research, Oakland, CA (S.S.); Division of Cardiovascular Sciences, National Heart, Lung, and Blood Institute, National Institutes of Health, Bethesda, MD (J.P.R.); and Division of Epidemiology and Community Health, School of Public Health, University of Minnesota, Minneapolis (D.R.J.). axz122@miami.edu (Vittinghoff) From the Division of Epidemiology and Population Health Sciences, Department of Public Health Sciences, University of Miami, FL (A.Z.A.H.); Departments of

Epidemiology and Biostatistics (E.V., K.Y.), Psychiatry (K.Y.), and Neurology (K.Y.), University of California San Francisco; San Francisco Veterans Affairs Medical Center, San Francisco, CA (K.Y.); Kaiser Permanente Division of Research, Oakland, CA (S.S.); Division of Cardiovascular Sciences, National Heart, Lung, and Blood Institute, National Institutes of Health, Bethesda, MD (J.P.R.); and Division of Epidemiology and Community Health, School of Public Health, University of Minnesota, Minneapolis (D.R.J.)

(Sidney) From the Division of Epidemiology and Population Health Sciences, Department of Public Health Sciences, University of Miami, FL (A.Z.A.H.); Departments of Epidemiology and Biostatistics (E.V., K.Y.), Psychiatry (K.Y.), and Neurology (K.Y.), University of California San Francisco; San Francisco Veterans Affairs Medical Center, San Francisco, CA (K.Y.); Kaiser Permanente Division of Research, Oakland, CA (S.S.); Division of Cardiovascular Sciences, National Heart, Lung, and Blood Institute, National Institutes of Health, Bethesda, MD (J.P.R.); and Division of Epidemiology and Community Health, School of Public Health, University of Minnesota, Minneapolis (D.R.J.)

(Reis) From the Division of Epidemiology and Population Health Sciences, Department of Public Health Sciences, University of Miami, FL (A.Z.A.H.); Departments of Epidemiology and Biostatistics (E.V., K.Y.), Psychiatry (K.Y.), and Neurology (K.Y.), University of California San Francisco; San Francisco Veterans Affairs Medical Center, San Francisco, CA (K.Y.); Kaiser Permanente Division of Research, Oakland, CA (S.S.); Division of Cardiovascular Sciences, National Heart, Lung, and Blood Institute, National Institutes of Health, Bethesda, MD (J.P.R.); and Division of Epidemiology and Community Health, School of Public Health, University of Minnesota, Minneapolis (D.R.J.)

(Jacobs) From the Division of Epidemiology and Population Health Sciences, Department of Public Health Sciences, University of Miami, FL (A.Z.A.H.); Departments of Epidemiology and Biostatistics (E.V., K.Y.), Psychiatry (K.Y.), and Neurology (K.Y.), University of California San Francisco; San Francisco Veterans Affairs Medical Center, San Francisco, CA (K.Y.); Kaiser Permanente Division of Research, Oakland, CA (S.S.); Division of Cardiovascular Sciences, National Heart, Lung, and Blood Institute, National Institutes of Health, Bethesda, MD (J.P.R.); and Division of Epidemiology and Community Health, School of Public Health, University of Minnesota, Minneapolis (D.R.J.)

(Yaffe) From the Division of Epidemiology and Population Health Sciences, Department of Public Health Sciences, University of Miami, FL (A.Z.A.H.); Departments of Epidemiology and Biostatistics (E.V., K.Y.), Psychiatry (K.Y.), and Neurology (K.Y.), University of California San Francisco; San Francisco Veterans Affairs Medical Center, San Francisco, CA (K.Y.); Kaiser Permanente Division of Research, Oakland, CA (S.S.); Division of Cardiovascular Sciences, National Heart, Lung, and Blood Institute, National Institutes of Health, Bethesda, MD (J.P.R.); and Division of Epidemiology and Community Health, School of Public Health, University of Minnesota, Minneapolis (D.R.J.)

Emtree Heading

For peer review only

adult; *arterial wall thickness; carotid artery disease; cerebrovascular accident; cognitive defect; cohort analysis; common carotid artery; coronary artery disease; *echography; female; human; male; middle aged; prospective study; psychology; risk factor; young adult.
Other Index Terms
adult; *arterial wall thickness; carotid artery disease; cerebrovascular accident; cognitive defect; cohort analysis; common carotid artery; coronary artery disease; *echography; female; human; male; middle aged; prospective study; psychology; risk factor; young adult.

Link to the Ovid Full Text or citation:
Click here for full text options

Link to the External Link Resolver:
SFX

146.

Ankle-brachial index but neither intima media thickness nor coronaryartery calcification is associated with mild cognitive impairment.
Weimar C., Winkler A., Dlugaj M., Lehmann N., Bauer M., Kroeger K., Koalsch H., Mahabadi A.-A., Dragano N., Moebus S., Hoffmann B., Joockel K.-H., Erbel R.
Alzheimer's and Dementia. Conference: Alzheimer's Association International Conference 2015. Washington, DC United States. Conference Publication: (var.pagings). 11 (7 SUPPL. 1) (pp P663-P664), 2015. Date of Publication: July 2015.
AN: 72125287
Background: Several studies have reported an association of atherosclerosis with mild cognitive impairment (MCI) and dementia independent of cardiovascular risk factors. As several clinical indicators of atherosclerosis exist, it is of interest if there is any variation in the association between the different indicators and MCI and its subtypes, amnestic MCI (aMCI) and non-amnestic MCI (naMCI). We therefore compared the cross-sectional association of the anklebrachial index (ABI), intimamedia thickness (IMT), and coronary artery calcification (CAC) with MCI, aMCI and naMCI in the population- based Heinz Nixdorf Recall cohort study.
Method(s): 4086 participants performed a validated brief cognitive assessment at the first follow-up examination (2006-2008).MCI was diagnosed according to previously published criteria. Logistic

For peer review only - http://bmjopen.bmj.com/site/about/guidelines.xhtml

regression models adjusted for age, gender, education, cardiovascular risk factors, and APOE genotype were used to compare the association of the ABI (ratio of the highest ankle artery systolic pressure and the highest systolic pressure measured in the right and left arm), the CAC - Agatston score (computed by summing weighted CAC scores of all foci in the epicardial coronary system) and the IMT (measured by ultrasound of the left and right common carotid artery) with MCI and its subtypes.

Result(s): We identified 490 participants with MCI (mean age 66.1 6 7.8, 46.9%male, aMCI n=249, naMCI n=241) and 1242 cognitively normal participants. A decreasing ABI (per 0.1) was significantly associated with a higher MCI prevalence in fully adjusted models (odds ratio (OR) 1.06 95% confidence interval (CI) 1.01-1.12), whereas an increasing CAC (log(CAC+1)) or IMT (per 0.1 mm) were not significantly associated after adjustment. In middle-aged participants (50-65 years), the association between ABI and both aMCI (1.13 95%CI 1.01-1.28) and naMCI (OR 1.15 95%CI 1.00-1.33) subtypes remained significant after adjustment for age and education but not in fully adjusted models. In old-aged participants (66-80 years), only the association between ABI and naMCI remained significant in fully adjusted models (OR 1.12 95%CI 1.01- 1.23).

Conclusion(s): Our data show that the degree of generalized atherosclerosis as measured by the ABI is associated with MCI and both MCI subtypes in middle-aged participants and with naMCI in old-aged participants.

Institution

(Weimar, Winkler, Dlugaj) Department of Neurology, University Hospital Essen, Essen, Germany

(Lehmann, Moebus, Joockel) Institute for Medical Informatics, Biometry and Epidemiology, University Hospital of Essen, Essen, Germany

(Bauer, Koalsch, Mahabadi, Erbel) Clinic of Cardiology, West German Heart and Vascular Centre, University Hospital of Essen, Essen, Germany

(Kroeger) Department of Angiology, Helios Kliniken Krefeld, Krefeld, Germany

(Dragano, Hoffmann) Institute for Medical Sociology, Centre for Healthy and Society, University of Dousseldorf, Dousseldorf, Germany

Publisher

Elsevier Inc.

Emtree Heading

*ankle brachial index; *arterial wall thickness; *calcification; *mild cognitive impairment; human; model; atherosclerosis; systolic blood pressure; cardiovascular risk; education; middle aged; coronary artery calcification; prevalence; cohort analysis; clinical indicator; arm; Agatston score; coronary artery calcium score; male; follow up; ankle; genotype; gender; recall; logistic regression analysis; population; artery; dementia; examination; ultrasound; right common carotid artery; risk; confidence interval.

Other Index Terms

For peer review only

*ankle brachial index; *arterial wall thickness; *calcification; *mild cognitive impairment; human; model; atherosclerosis; systolic blood pressure; cardiovascular risk; education; middle aged; coronary artery calcification; prevalence; cohort analysis; clinical indicator; arm; Agatston score; coronary artery calcium score; male; follow up; ankle; genotype; gender; recall; logistic regression analysis; population; artery; dementia; examination; ultrasound; right common carotid artery; risk; confidence interval.

Link to the Ovid Full Text or citation:
Click here for full text options

Link to the External Link Resolver:
SFX

147.

Cerebral perfusion and cognitive status before and in early period after carotid endarterectomy for symptomatic internal carotid stenosis.
Mushba A., Tsvetkova A., Vinogradov O., Kuznetsov A.
Journal of the Neurological Sciences. Conference: 22nd World Congress of Neurology, WCN 2015. Santiago Chile. Conference Publication: (var.pagings). 357 (SUPPL. 1) (pp e397-e398), 2015. Date of Publication: 15 Oct 2015.
AN: 72092469
Background: Atherosclerosis of extracranial and intracranial arteries is regarded as an origin of 25% of ischemic strokes. Carotid endarterectomy (CEA) is a routine surgery for secondary prophylaxis of stroke in patients with symptomatic internal carotid artery stenosis. Nevertheless, it is still unclear, if the removal of atherosclerosis plaque from internal carotid artery (ICA) leads to improvement of cerebral perfusion and cognitive functions. Goal of our study was to estimate cerebral perfusion and cognitive status before and after CEA for atherothrombotic ischemic stroke.
Material(s) and Method(s): 23 patients (20 [86%]male, 3 [14%] female, mean age 61.5 +/-6.7) operated by CEA for atherothrombotic ischemic stroke were included in our study. Pre- and postoperatively all patients were studied by cognitive status scale(MMSE, MoCa), cognitive evoke potentials (P-300), ultrasound scan of brachiocephalic arteries, transcranial duplex scan of intracranial arteries, MRI of brain, SPECT with 99mTc-HMPAO.

For peer review only - http://bmjopen.bmj.com/site/about/guidelines.xhtml

Result(s): Improvement of cerebral perfusion in affected hemisphere was demonstrated after CEA. SPECT of affected hemisphere showed that perfusion index in gyrus temporale superior, gyrus temporale medium and thalamus increased from 70.73% to 78.66% (p=0.01), from 82.20% to 87.37% (p=0.04), and from 58.7% to 68.99% (p=0.009), respectively. Improvement of cerebral perfusion in our study has not resulted in cognitive improvement: mean MMSE pre- and postoperatively appeared to be 26.1+/-2.24 and 26.1+/-1.14 (p > 0.05), respectively; mean MoCa pre- and postoperatively were 27+/- 2 and 27.3+/- 1,1 (p > 0.05), respectively. This data corresponded results of neurofunctional study: P- 300 latency of cognitive evoke potentials pre- and postoperatively were 392.1+/-49.5 msec and 37.8+/-46.1 msec (p > 0.05), respectively.
Conclusion(s): CEA for symptomatic ICA stenosis results in improvement of cerebral perfusion in affected hemisphere, but not associated with cognitive improvement in early period after surgery.

Institution

(Mushba, Tsvetkova, Vinogradov, Kuznetsov) National Center of Cerebrovascular Disorders, National Pirogov Centre of Therapy and Surgery, Moscow, Russian Federation

Publisher

Elsevier

Emtree Heading

*brain perfusion; *carotid endarterectomy; *carotid artery obstruction; *neurology; human; patient; brain ischemia; hemisphere; internal carotid artery; artery; atherosclerosis; surgery; brachiocephalic trunk; ultrasound; single photon emission computer tomography; cognition; female; cerebrovascular accident; prophylaxis; stenosis; latent period; thalamus; perfusion; male; brain; Mini Mental State Examination; nuclear magnetic resonance imaging; carcinoembryonic antigen; technetium 99m.

Drug Index Terms

carcinoembryonic antigen; technetium 99m.

Other Index Terms

*brain perfusion; *carotid endarterectomy; *carotid artery obstruction; *neurology; human; patient; brain ischemia; hemisphere; internal carotid artery; artery; atherosclerosis; surgery; brachiocephalic trunk; ultrasound; single photon emission computer tomography; cognition; female; cerebrovascular accident; prophylaxis; stenosis; latent period; thalamus; perfusion; male; brain; Mini Mental State Examination; nuclear magnetic resonance imaging.

Link to the Ovid Full Text or citation:

Click here for full text options

Link to the External Link Resolver:

For peer review only - http://bmjopen.bmj.com/site/about/guidelines.xhtml

SFX

149.

Dynamics of cognitive functions condition in patients after bilateral carotid endarterectomy.
Ataniyazov M., Tadjenov M., Rakhimbaeva G.
Atherosclerosis. Conference: 83rd European Atherosclerosis Society Congress, EAS 2015. Glasgow United Kingdom. Conference Publication: (var.pagings). 241 (1) (pp e214), 2015. Date of Publication: July 2015.
AN: 71970727
Aim: The primary aim of our study was to establish cerebral hemodynamics and cognitive functions in patients with total carotid artery stenosis who underwent carotid endarterectomy (CEA).
Method(s): We examined 117 patients with different level of chronic vascular cerebral insufficiency (CVCI). All patients had a bilateral hemodynamically significant stenosis of the carotid artery bifurcation and varying degrees of cognitive impairment. The average age of the patients was 62,1 +/- 4,2 years. Among the comorbidities of IHD was observed 68.5% of the patients, 15.6% of patients with early myocardial infarction, hypertension was present in 78.5% of patients, diabetes mellitus in 11.2%, atherosclerotic lesions of extremity arteries - 21.4%.
Result(s): Before CEA, MMSE score was 18,3+/-0,64; 3 months after the first CEA it was 22,2 +/- 0,78. 3 months after the second CEAMMSE average score increased to 26,4 +/- 0,54. Before surgery, the patient's linear blood flow velocity (BFV) of ACA at TCD was 42,14 +/- 3,6 cm/s, MCA - 54,1 +/- 2,3 cm/sec and PCA - 50,8 +/- 1,2 cm/sec. 3 months after the first operation, BFV in ACA increased to 52,4 +/- 3,4 cm/sec, in MCA to 66,1 +/- 5,3 cm/sec and in PCA to 64,6+/-2,3cm/sec. 3 months after the second operation CEA, BFV in ACA increased to 62,4+/-2,1cm/sec, in MCA to 78,1+/-6,3cm/sec and in PCA to 72,4+/-4,1cm/sec.
Conclusion(s): To sum up, bilateral hemodynamically significant stenotic lesion of carotid artery contributes to the development of moderate to severe cognitive impairment.
Institution
(Ataniyazov, Tadjenov, Rakhimbaeva) Neurology, Tashkent Medical Academy, Tashkent, Uzbekistan
Publisher
Elsevier Ireland Ltd
Emtree Heading

For peer review only - http://bmjopen.bmj.com/site/about/guidelines.xhtml

*cognition; *patient; *human; *carotid endarterectomy; *European; *atherosclerosis; *society; *dynamics; cognitive defect; brain dysfunction; diabetes mellitus; carotid artery obstruction; hypertension; heart infarction; hemodynamics; artery; surgery; blood flow velocity; carotid artery bifurcation; carotid artery; stenosis; Mini Mental State Examination; carcinoembryonic antigen.

Drug Index Terms

carcinoembryonic antigen.

Other Index Terms

*cognition; *patient; *human; *carotid endarterectomy; *European; *atherosclerosis; *society; *dynamics; cognitive defect; brain dysfunction; diabetes mellitus; carotid artery obstruction; hypertension; heart infarction; hemodynamics; artery; surgery; blood flow velocity; carotid artery bifurcation; carotid artery; stenosis; Mini Mental State Examination.

Link to the Ovid Full Text or citation:

Click here for full text options

Link to the External Link Resolver:

SFX

150.

Training and environmental enrichment to counteract cognitive decline: Train the brain effects on carotid structure and function.

Stea F., Bruno R., Ghiadoni L., Faita F., Di Lascio N., Del Turco S., Maffei L., Tognoni G., Taddei S., Picano E., Sicari R.

Journal of Hypertension. Conference: 25th European Meeting on Hypertension and Cardiovascular Protection, ESH 2015. Milan Italy. Conference Publication: (var.pagings). 33 (SUPPL. 1) (pp e96), 2015. Date of Publication: June 2015.

AN: 71934878

Objective: Physical activity is beneficial to vascular health; on the other hand, vascular damage is associated with cognitive impairment. Both physical activity and a cognitively stimulating environment are known to delay the onset of dementia. The Train The Brain study evaluates the effectiveness of a comprehensive program of physical training and mental activity in delaying cognitive decline in elderly people with mild cognitive impairment, at the same time investigating

For peer review only

the relationship between physical, vascular, neurological, and cognitive fitness Design and method: Elders age 65-89 were recruited with the help of family physicians and territorial services. All participants underwent a neurological and cardiologic evaluation. In the vascular study, carotid pressure was measured with the SphygmoCor system (AtCor, Australia); longitudinal ultrasound scans of the common carotid were performed and 10-second video clips were recorded to be analyzed offline through the Cardiovascular Suite software (Quipu srl, Italy), with the computation of diameter, intima-media thickness, wall cross-sectional area, distensibility coefficient, compliance, stiffness, and elastic modulus. Subjects classified as mild cognitive impairment at the neurological examination were randomized either to standard care, or a 7-month program of physical training and environmental stimulation (lectures, games, music, social activities) three hours a week. The evaluation was then repeated.

Result(s): Data were obtained for 57 patients who underwent training (T) and 30 controls (C). The only significant difference at baseline was in the distensibility coefficient (p = 0.045). (Table Presented) Vessel diameter increased in C and decreased in T; distensibility decreased in C; all carotid parameters were influenced by the combination of time and treatment, in a diverging trend, at a statistically significant level, while there was no effect on pressure. Introducing arterial pressures as covariates did not affect the findings.

Conclusion(s): There was a significant difference in behavior in time of the two groups as for vessel enlargement, wall thickening and arterial stiffening. The proposed program of physical training and environmental enrichening seems to oppose the typical harmful effects of aging on the wall of the common carotid in elderly people with mild cognitive impairment.

Institution

(Stea, Bruno, Faita, Di Lascio, Del Turco, Picano, Sicari) Institute of Clinical Physiology, National Research Council, Pisa, Italy (Ghiadoni, Taddei) Department of Clinical and Experimental Medicine, University of Pisa, Pisa, Italy

(Maffei) Institute of Neuroscience, National Research Council, Pisa, Italy

(Tognoni) Neurology Unit, University Hospital of Pisa, Pisa, Italy

Publisher

Lippincott Williams and Wilkins

Emtree Heading

*environmental enrichment; *brain; *carotid artery; *European; *hypertension; *protection; human; mild cognitive impairment; training; compliance (physical); aged; physical activity; videorecording; Australia; dementia; fitness; environment; health; parameters; social behavior; cognitive defect; arterial pressure; general practitioner; ultrasound; music; clip; stimulation; neurologic examination; Young modulus; rigidity; blood vessel injury; arterial wall thickness; Italy; computer program; patient; mental performance; arterial stiffness; aging.

Other Index Terms

For peer review only - http://bmjopen.bmj.com/site/about/guidelines.xhtml

*environmental enrichment; *brain; *carotid artery; *European; *hypertension; *protection; human; mild cognitive impairment; training; compliance (physical); aged; physical activity; videorecording; Australia; dementia; fitness; environment; health; parameters; social behavior; cognitive defect; arterial pressure; general practitioner; ultrasound; music; clip; stimulation; neurologic examination; Young modulus; rigidity; blood vessel injury; arterial wall thickness; Italy; computer program; patient; mental performance; arterial stiffness; aging.

Link to the Ovid Full Text or citation:
[Click here for full text options]

Link to the External Link Resolver:
[SFX]

152.

Subclinical atherosclerosis and 20-year cognitive decline: The atherosclerosis risk in communities (ARIC) neurocognitive study.
Love S.-A.M., Palta P., Kalbaugh C.A., Sharrett A.R., Gross A.L., Alonso A., Wruck L.M., Snyder M.L., Mosley T.H., Heiss G.
Circulation. Conference: American Heart Association's Epidemiology and Prevention/Lifestyle and Cardiometabolic Health 2015 Scientific Sessions. Baltimore, MD United States. Conference Publication: (var.pagings). 131 (SUPPL. 1) (no pagination), 2015. Date of Publication: 10 Mar 2015.
AN: 71819426
Introduction: Cardiovascular risk factors are reportedly predictive of cognitive decline and dementia but the association between the extent and severity of subclinical atherosclerosis with cognitive decline remains understudied. Hypothesis: The systemic burden of atherosclerosis measured non-invasively is associated with the rate of decline in domain-specific (memory, executive function and language) and global cognition from mid-life to late life.
Method(s): Members of the ARIC cohort (N=12313; 58% women, 24% African American (AA), 76% white) aged 46-70 years at their 1990-1992 examination were followed through 2011-2013. Participants with prevalent stroke, myocardial infarction or coronary heart disease were excluded. Atherosclerosis at baseline (n=5217) was assessed by carotid artery b-mode ultrasound (presence and number of plaques, bilaterally) and by ankle-brachial index <0.9 measured with an

For peer review only - http://bmjopen.bmj.com/site/about/guidelines.xhtml

oscillometric device. Tests of memory (Delayed Word Recall Test), executive function (Digit Symbol Substitution Test), and language (Word Fluency Test) were administered in 1990-92, 1996-98 and 2011-13. Test-specific z scores were calculated at each exam based on the means and standard deviations at baseline. A global cognition z score was estimated by averaging the 3 test-specific z scores and standardizing to baseline. Race-stratified linear random effects regression was used to estimate the association between subclinical atherosclerosis and 20-year declines in domain-specific cognition and global cognition. We adjusted for age, sex and level of education. Inverse probability weighting (IPW) was used to limit bias due to attrition.

Result(s): In AA, the presence of carotid plaque and/or ABI <0.90 (n=490) was associated with a lower memory z score (Beta=-0.10, 95% confidence interval, CI: -0.18, -0.02), a lower language z score (Beta=-0.07, 95% CI: -0.14,-0.002) and a lower global cognition z score at baseline (Beta=-0.09, 95% CI: -0.16, -0.02), but not with rates of change in any cognitive score. Among whites at baseline, individuals with subclinical atherosclerosis (n=4099) exhibited lower executive function (Beta=-0.05, 95% CI: -0.08, -0.02) and global cognition (Beta=-0.04, 95% CI: -0.07, -0.01). White participants with subclinical atherosclerosis had a greater 20-year rate of decline in global cognition (Beta=-0.06, 95% CI: -0.10, -0.00) compared to those without subclinical atherosclerosis.

Conclusion(s): Baseline memory, language, and global cognition in AA and executive function and global cognition in whites were lower among those with non-invasively ascertained atherosclerosis compared to those without, independent of covariates in the model. Among whites, subclinical clinical measures of atherosclerosis in mid-life may be indicative of modest, but measurable declines in cognition after additional adjustment for potential bias due to attrition.

Institution

(Love, Palta, Kalbaugh, Wruck, Snyder, Heiss) Univ of North Carolina at Chapel Hill, Chapel Hill, NC, United States (Sharrett) Johns Hopkins Univ, Baltimore, MD, United States

(Gross) Johns Hopkins Univeristy, Baltimore, MD, United States

(Alonso) Univ of Minnesota, Minneapolis, MN, United States

(Mosley) Univ of Mississippi Med Cntr, Jackson, MS, United States

Publisher

Lippincott Williams and Wilkins

Emtree Heading

*atherosclerosis; *community; *medical society; *epidemiology; *health; *risk; cognition; memory; language; executive function; human; carotid artery; ultrasound; ischemic heart disease; dementia; heart infarction; cardiovascular risk; cerebrovascular accident; confidence interval; examination; tracheobronchial stent; African American; digit symbol substitution test; word recognition; hypothesis; devices; ankle brachial index; female; education; model.

Other Index Terms

For peer review only - http://bmjopen.bmj.com/site/about/guidelines.xhtml

*atherosclerosis; *community; *medical society; *epidemiology; *health; *risk; cognition; memory; language; executive function; human; carotid artery; ultrasound; ischemic heart disease; dementia; heart infarction; cardiovascular risk; cerebrovascular accident; confidence interval; examination; tracheobronchial stent; African American; digit symbol substitution test; word recognition; hypothesis; devices; ankle brachial index; female; education; model.

Link to the Ovid Full Text or citation:
Click here for full text options

Link to the External Link Resolver:
SFX

156.

Correlation of cognitive function with ultrasound strain indices in carotid plaque.
Wang X., Jackson D.C., Varghese T., Mitchell C.C., Hermann B.P., Kliewer M.A., Dempsey R.J.
Ultrasound in Medicine and Biology. 40 (1) (pp 78-89), 2014. Date of Publication: January 2014.
AN: 52807319
Instability in carotid vulnerable plaque can generate cerebral micro-emboli, which may be related to both stroke and eventual cognitive abnormality. Strain imaging to detect plaque vulnerability based on regions with large strain fluctuations, with arterial pulsation, may be able to determine the risk of cognitive impairment. Plaque instability may be characterized by increased strain variations over a cardiac cycle. Radiofrequency signals for ultrasound strain imaging were acquired from the carotid arteries of 24 human patients using a Siemens Antares with a VFX 13-5 linear array transducer. These patients underwent standardized cognitive assessment (Repeatable Battery for the Assessment of Neuropsychological Status [RBANS]). Plaque regions were segmented by a radiologist at end-diastole using the Medical Imaging Interaction Toolkit. A hierarchical block-matching motion tracking algorithm was used to estimate the cumulated axial, lateral and shear strains within the imaging plane. The maximum, minimum and peak-to-peak strain indices in the plaque computed from the mean cumulated strain over a small region of interest in the plaque with large deformations were obtained. The maximum and peak-to-peak mean cumulated strain indices over the entire plaque region were also computed. All strain indices were then correlated with RBANS Total performance. Overall cognitive performance (RBANS Total) was negatively

For peer review only - http://bmjopen.bmj.com/site/about/guidelines.xhtml
For peer review only

associated with values of the maximum strain and the peak-to-peak for axial and lateral strains, respectively. There was no significant correlation between the RBANS Total score and shear strain and strain indices averaged over the entire identified plaque for this group of patients. However, correlation of maximum lateral strain was higher for symptomatic patients (r=-0.650, p=0.006) than for asymptomatic patients (r=-0.115, p=0.803). On the other hand, correlation of maximum axial strain averaged over the entire plaque region was significantly higher for asymptomatic patients (r=-0.817, p=0.016) than for symptomatic patients (r=-0.224, p=0.402). The results reveal a direct relationship between the maximum axial and lateral strain indices in carotid plaque and cognitive impairment. © 2014 World Federation for Ultrasound in Medicine & Biology.

PMID

24120415 [http://www.ncbi.nlm.nih.gov/pubmed/?term=24120415]

Institution

(Wang, Varghese) Department of Medical Physics, University of Wisconsin-Madison, Madison, WI, United States (Jackson, Hermann) Department of Neurology, University of Wisconsin-Madison, Madison, WI, United States

(Mitchell) Department of Biomedical Sciences, College of Health Sciences, University of Wisconsin-Milwaukee, Milwaukee, WI, United States

(Kliewer) Department of Radiology, University of Wisconsin-Madison, Madison, WI, United States

(Dempsey) Department of Neurologic Surgery, University of Wisconsin-Madison, Madison, WI, United States

Publisher

Elsevier USA

Emtree Heading

adult; aged; algorithm; article; carotid artery; carotid atherosclerosis/di [Diagnosis]; clinical article; *cognition; cognitive defect; controlled study; diagnostic imaging; *echography; female; heart cycle; human; male; microembolism/di [Diagnosis]; middle aged; priority journal; pulse wave; radiofrequency radiation; transducer; transient ischemic attack/di [Diagnosis]; young adult.

Other Index Terms

adult; aged; algorithm; article; carotid artery; carotid atherosclerosis / diagnosis; clinical article; *cognition; cognitive defect; controlled study; diagnostic imaging; *echography; female; heart cycle; human; male; microembolism / diagnosis; middle aged; priority journal; pulse wave; radiofrequency radiation; transducer; transient ischemic attack / diagnosis; young adult.

Link to the Ovid Full Text or citation:

Click here for full text options

For peer review only

Link to the External Link Resolver:

SFX

157.

The role of carotid intima-media thickness in predicting longitudinal cognitive function in an older adult cohort.

Frazier D.T., Seider T., Bettcher B.M., Mack W.J., Jastrzab L., Chao L., Weiner M.W., Decarli C., Reed B.R., Mungas D., Chui H.C., Kramer J.H.

Cerebrovascular Diseases. 38 (6) (pp 441-447), 2014. Date of Publication: 21 Jan 2014.

AN: 600782243

Background and Purpose: Carotid atherosclerosis is a risk factor for cerebrovascular disease in older adults. Although age-related cognitive decline has been associated with cerebrovascular disease, not much is known about the consequences of carotid atherosclerosis on longitudinal cognitive function. This study examines the longitudinal relationship between atherosclerosis and cognition in a sample of non-demented older subjects using baseline measurements of carotid intima media thickness (CIMT) and annual cognitive measures of executive function (EXEC) and verbal memory (MEM).

Method(s): Baseline measurements included CIMT derived from B-mode carotid artery ultrasound, structural T1-weighted images of white matter hypointensities (WMH), white matter lesions (WML), and cerebral infarct. Hypertension, low-density lipoprotein (LDL), diabetes, and waist to hip ratios (WHR) were included as covariates in our models to control for cerebrovascular risks and central adiposity. Annual composite scores of EXEC and MEM functions were derived from item response theory. Linear mixed models were used to model longitudinal cognitive change.

Result(s): A significant inverse relationship was found between baseline CIMT and annual EXEC score, but not annual MEM score. Subjects included in the highest 4th quartile of CIMT showed a rate of annual decline in EXEC score that was significant relative to subjects in lower quartile groups ($p < 0.01$). The relationship between the 4th quartile of CIMT and annual EXEC score remained significant after independently adjusting for imaging measures of white matter injury and cerebral infarct.

Conclusion(s): Older adult subjects with the highest index of CIMT showed an annual decline in EXEC scores that was significant relative to subjects with lower quartile measurements of CIMT, independent of our measures of white matter injury and cerebral infarct. Our findings suggest that

For peer review only - http://bmjopen.bmj.com/site/about/guidelines.xhtml

elevated measures of CIMT may mark an atherosclerotic state, resulting in a decline in executive function and not memory in non-demented older adults.

Copyright © 2014 S. Karger AG, Basel.

PMID

25502351 [http://www.ncbi.nlm.nih.gov/pubmed/?term=25502351]

Institution

(Frazier, Bettcher, Jastrzab, Kramer) UCSF Memory and Aging Center, University of California, 675 Nelson Rising Lane, San Francisco, CA 94143-1207, United States (Frazier, Bettcher, Jastrzab, Kramer) Department of Neurology, University of California, San Francisco, CA, United States

(Seider) Department of Clinical and Health Psychology, University of Florida, Gainesville, FL, United States

(Mack) Department of Preventive Medicine, Keck School of Medicine, University of Southern California, Los Angeles, CA, United States

(Chao, Weiner) Center for Imaging of Neurodegenerative Diseases, University of California, San Francisco, CA, United States

(Decarli, Reed, Mungas) Department of Neurology, School of Medicine, University of California, Davis, CA, United States

(Chui) Department of Neurology, Keck School of Medicine, University of Southern California, Los Angeles, CA, United States

Publisher

S. Karger AG

Emtree Heading

adult; aged; *arterial wall thickness; article; atherosclerosis; B scan; brain infarction; carotid artery; *cognition; cohort analysis; deterioration; diabetes mellitus; disease association; *executive function; female; human; hypercholesterolemia; hypertension; major clinical study; male; obesity; priority journal; risk factor; theory; *verbal memory; waist hip ratio; white matter injury; white matter lesion; low density lipoprotein/ec [Endogenous Compound].

Drug Index Terms

low density lipoprotein / endogenous compound.

Other Index Terms

adult; aged; *arterial wall thickness; Article; atherosclerosis; B scan; brain infarction; carotid artery; *cognition; cohort analysis; deterioration; diabetes mellitus; disease association; *executive function; female; human; hypercholesterolemia; hypertension; major clinical study; male; obesity; priority journal; risk factor; theory; *verbal memory; waist hip ratio; white matter injury; white matter lesion.

For peer review only

Link to the Ovid Full Text or citation:

Click here for full text options

Link to the External Link Resolver:

SFX

161.

An inflammatory pathway links atherosclerotic cardiovascular disease risk to neural activity evoked by the cognitive regulation of emotion.

Gianaros P.J., Marsland A.L., Kuan D.C.-H., Schirda B.L., Jennings J.R., Sheu L.K., Hariri A.R., Gross J.J., Manuck S.B.

Biological Psychiatry. 75 (9) (pp 738-745), 2014. Date of Publication: 01 May 2014.

AN: 52875335

Background Cognitive reappraisal is a form of emotion regulation that alters emotional responding by changing the meaning of emotional stimuli. Reappraisal engages regions of the prefrontal cortex that support multiple functions, including visceral control functions implicated in regulating the immune system. Immune activity plays a role in the preclinical pathophysiology of atherosclerotic cardiovascular disease (CVD), an inflammatory condition that is highly comorbid with affective disorders characterized by problems with emotion regulation. Here, we tested whether prefrontal engagement by reappraisal would be associated with atherosclerotic CVD risk and whether this association would be mediated by inflammatory activity. Methods Community volunteers (n = 157; 30-54 years of age; 80 women) without DSM-IV Axis-1 psychiatric diagnoses or cardiovascular or immune disorders performed a functional neuroimaging task involving the reappraisal of negative emotional stimuli. Carotid artery intima-media thickness and inter-adventitial diameter were measured by ultrasonography and used as markers of preclinical atherosclerosis. Also measured were circulating levels of interleukin-6 (IL-6), an inflammatory cytokine linked to CVD risk and prefrontal neural activity. Results Greater reappraisal-related engagement of the dorsal anterior cingulate cortex was associated with greater preclinical atherosclerosis and IL-6. Moreover, IL-6 mediated the association of dorsal anterior cingulate cortex engagement with preclinical atherosclerosis. These results were independent of age, sex, race, smoking status, and other known CVD risk factors. Conclusions The cognitive regulation of emotion might relate to CVD risk through a pathway involving the functional interplay between the

For peer review only - http://bmjopen.bmj.com/site/about/guidelines.xhtml

anterior cingulate region of the prefrontal cortex and inflammatory activity. © 2014 Society of Biological Psychiatry.

PMID

24267410 [http://www.ncbi.nlm.nih.gov/pubmed/?term=24267410]

Institution

(Gianaros, Marsland, Kuan, Sheu, Manuck) Department of Psychology, University of Pittsburgh, 506 Old Engineering Hall, 3943 O'Hara Street, Pittsburgh, PA 15260-9150, United States (Jennings) Department of Psychiatry, University of Pittsburgh, Pittsburgh, PA, United States

(Schirda) Department of Psychology, Ohio State University, Columbus, OH, United States

(Hariri) Department of Psychology and Neuroscience, Institute for Genome Sciences and Policy, Duke University, Durham, NC, United States

(Gross) Department of Psychology, Stanford University, Stanford, CA, United States

Publisher

Elsevier USA

Emtree Heading

adult; anterior cingulate; arterial wall thickness; article; *atherosclerotic cardiovascular disease/di [Diagnosis]; BOLD signal; *cardiovascular risk; *cognition; controlled study; DSM-IV; echography; *electroencephalogram; *emotionality; facial expression; female; functional neuroimaging; human; *inflammatory disease; major clinical study; male; prefrontal cortex; priority journal; smoking; interleukin 6/ec [Endogenous Compound].

Drug Index Terms

interleukin 6 / endogenous compound.

Other Index Terms

adult; anterior cingulate; arterial wall thickness; article; *atherosclerotic cardiovascular disease / *diagnosis; BOLD signal; *cardiovascular risk; *cognition; controlled study; DSM-IV; echography; *electroencephalogram; *emotionality; facial expression; female; functional neuroimaging; human; *inflammatory disease; major clinical study; male; prefrontal cortex; priority journal; smoking.

Link to the Ovid Full Text or citation:

Click here for full text options

Link to the External Link Resolver:

SFX

For peer review only

162.

Cognitive deterioration in bilateral asymptomatic severe carotid stenosis.

Buratti L., Balucani C., Viticchi G., Falsetti L., Altamura C., Avitabile E., Provinciali L., Vernieri F., Silvestrini M.

Stroke. 45 (7) (pp 2072-2077), 2014. Date of Publication: July 2014.
AN: 53180538

BACKGROUND AND PURPOSE - : This study aimed to monitor cognitive performance during a 3-year period in subjects with bilateral asymptomatic severe internal carotid artery stenosis and to explore the role of cerebral hemodynamics and atherosclerotic disease in the development of cognitive dysfunction. METHODS - : One hundred fifty-nine subjects with bilateral asymptomatic severe internal carotid artery stenosis were included and prospectively evaluated for a 3-year period. At entry, demographics, vascular risk profile, and pharmacological treatments were defined. Cognitive status was evaluated using the Mini-Mental State Examination at baseline and at follow-up. Cerebral hemodynamics was assessed by transcranial Doppler-based breath-holding index test. As a measure of the extent of systemic atherosclerotic disease, common carotid artery intima-media thickness was measured. A cutoff for pathological values was set at 0.69 for breath-holding index and 1.0 mm for intima-media thickness. RESULTS - : The risk of decreasing in Mini-Mental State Examination score increased progressively from patients with bilaterally normal to those with unilaterally abnormal breath-holding index, reaching the highest probability in patients with bilaterally abnormal breath-holding index (P<0.0001). Pathological values of intima-media thickness did not influence the risk of Mini-Mental State Examination score change. CONCLUSIONS - : Our findings suggest that patients with asymptomatic bilateral severe internal carotid artery stenosis may be at risk of developing cognitive impairment. The evaluation of the hemodynamic status, besides providing insights about the possible mechanism behind the cognitive dysfunction present in carotid atherosclerotic disease, may be of help for the individuation of subjects deserving earlier and more aggressive treatments. © 2014 American Heart Association, Inc.

PMID
24903984 [http://www.ncbi.nlm.nih.gov/pubmed/?term=24903984]

Institution
(Buratti, Viticchi, Avitabile, Provinciali, Silvestrini) Neurological Clinic, Marche Polytechnic University, Via Conca 1, 60020 Ancona, Italy (Balucani) Department of Neurology, SUNY Downstate Medical Center, Brooklyn, NY, United States
(Falsetti) Internal and Subintensive Medicine, Ospedali Riuniti Ancona, Ancona, Italy
(Altamura, Vernieri) Neurology Unit, Campus Bio-Medico University, Rome, Italy

For peer review only - http://bmjopen.bmj.com/site/about/guidelines.xhtml

Publisher
Lippincott Williams and Wilkins (E-mail: LRorders@phl.lrpub.com)
Emtree Heading
aged; arterial wall thickness; article; breath holding; cognition; common carotid artery; demography; diabetes mellitus; dyslipidemia; female; follow up; heart atrium fibrillation; hemodynamics; human; hypertension; *internal carotid artery occlusion; major clinical study; male; *mental deterioration; middle cerebral artery; Mini Mental State Examination; peripheral occlusive artery disease; priority journal; prospective study; Rankin scale; transcranial doppler.
Other Index Terms
aged; arterial wall thickness; article; breath holding; cognition; common carotid artery; demography; diabetes mellitus; dyslipidemia; female; follow up; heart atrium fibrillation; hemodynamics; human; hypertension; *internal carotid artery occlusion; major clinical study; male; *mental deterioration; middle cerebral artery; Mini Mental State Examination; peripheral occlusive artery disease; priority journal; prospective study; Rankin scale; transcranial doppler.

Link to the Ovid Full Text or citation:
Click here for full text options

Link to the External Link Resolver:
SFX

164.

Association of cognitive dysfunction with cardiovascular disease events in elderly hypertensive patients.
Yano Y., Bakris G.L., Inokuchi T., Ohba Y., Tamaki N., Nagata M., Kuwabara M., Yokota N., Eto T., Kuroki M., Shimada K., Kario K.
Journal of Hypertension. 32 (2) (pp 423-431), 2014. Date of Publication: February 2014.
AN: 52921730
Objectives: This study assesses whether presence of cognitive dysfunction can be a marker associated with the development of cardiovascular disease (CVD) events independent of ambulatory blood pressure (BP) or other indices of target organ damage (TOD) in elderly hypertensive patients.

For peer review only

Method(s): We recruited 585 hypertensive patients (mean age, 73 years; 41% men) who were ambulatory, lived independently, and were without clinically overt dementia. Cognitive function was assessed by Mini-Mental State Examination (MMSE) at baseline, and CVD events (coronary artery disease, stroke, congestive heart failure, and sudden death) were prospectively ascertained. Cognitive dysfunction was defined as the lowest quartile of MMSE scores (n=183, median 24 points).

Result(s): CVD events occurred in 42 people over an average of 2.8 years (1644 person-years). The prevalence of cognitive dysfunction was higher in patients with CVD events than those without (57 vs. 29%; both P<0.001) at baseline. Cognitive dysfunction was associated with CVD events, after adjustment for nocturnal SBP and evidence of TOD [i.e. albuminuria, cardiac hypertrophy, and carotid-artery intima-media thickness (IMT)], hazard ratio 2.5-2.9 (all P<0.01). Incorporation of MMSE in the risk model (including age, estimated glomerular filtration rate, and preexisting CVD) improved the C-statistics (from 0.691 to 0.741) and resulted in a net reclassification improvement of 17.6% (P=0.02). In contrast, incorporation of albuminuria, cardiac hypertrophy, and high carotid-artery IMT added little further improvement in the risk prediction.

Conclusion(s): Cognitive dysfunction is an independent marker associated with increased risk of CVD events in elderly hypertensive patients. © 2014 Wolters Kluwer Health Lippincott Williams & Wilkins.

PMID

24351802 [http://www.ncbi.nlm.nih.gov/pubmed/?term=24351802]

Institution

(Yano, Bakris) Department of Medicine, American Society of Hypertension Comprehensive Hypertension Center, University of Chicago Medicine, 5841 S. Maryland Avenue, MC 1027, Chicago, IL 60637, United States (Yano, Ohba, Shimada, Kario) Division of Cardiovascular Medicine, Department of Medicine, Jichi Medical University School of Medicine, Tochigi, Japan

(Inokuchi) Department of Orthopedics, Chikamorikai Medical Group, Kochi, Japan

(Tamaki) Tamaki Clinic, Miyazaki, Japan

(Nagata) Kijo Clinic, Miyazaki, Japan

(Kuwabara) Kuwabara Clinic, Miyazaki, Japan

(Yokota) Yokota Naika, Miyazaki, Japan

(Eto) Eto Cardiology Clinic, Miyazaki, Japan

(Kuroki) Kuroki Naika, Miyazaki, Japan

Publisher

Lippincott Williams and Wilkins (250 Waterloo Road, London SE1 8RD, United Kingdom)

Emtree Heading

adult; age; aged; aged hospital patient; albuminuria; arterial wall thickness; article; *cardiovascular disease; cardiovascular risk; cerebrovascular accident; *cognitive defect/ep [Epidemiology];

For peer review only - http://bmjopen.bmj.com/site/about/guidelines.xhtml

congestive heart failure; coronary artery disease; disease association; female; *geriatric disorder; glomerulus filtration rate; heart ventricle hypertrophy; high risk patient; human; *hypertension; major clinical study; male; Mini Mental State Examination; prevalence; priority journal; prospective study; sudden cardiac death; systolic blood pressure; target organ.

Other Index Terms

adult; age; aged; aged hospital patient; albuminuria; arterial wall thickness; article; *cardiovascular disease; cardiovascular risk; cerebrovascular accident; *cognitive defect / *epidemiology; congestive heart failure; coronary artery disease; disease association; female; *geriatric disorder; glomerulus filtration rate; heart ventricle hypertrophy; high risk patient; human; *hypertension; major clinical study; male; Mini Mental State Examination; prevalence; priority journal; prospective study; sudden cardiac death; systolic blood pressure; target organ.

Link to the Ovid Full Text or citation:

Click here for full text options

Link to the External Link Resolver:

SFX

166.

Cardiovascular Biomarkers and Carotid IMT Scores as Predictors of Cognitive Function.

Masley S.C., Masley L.V., Gualtieri C.T.

Journal of the American College of Nutrition. 33 (1) (pp 63-69), 2014. Date of Publication: February 2014.

AN: 372411102

Objective: Multiple cardiovascular risk factors are associated with early cognitive decline. Measures of complex information processing provide one of the earliest signs of cognitive decline and appear related to arterial plaque growth. The purpose of this study was to determine how cardiovascular risk factors and carotid intima-media thickness (IMT) scores are associated with cognitive function and complex information processing scores.

Method(s): This study used a retrospective, cross-sectional analysis of 536 men and women attending an executive evaluation program. Measurements were made of body composition,

For peer review only

cardiovascular status, fitness and diet, and laboratory measures, including carotid IMT. Each subject was tested with a computerized neurocognitive test battery.
Result(s): Complex information processing (CIP), also called executive function, is independently related to carotid IMT scores ($p < 0.01$), as are other cardiovascular biomarkers, including aerobic capacity fiber, B12, and long-chain n-3 fatty acid intake ($p < 0.01$ for each). However, after controlling for carotid IMT, only IMT showed a significant relationship with CIP scores.
Conclusion(s): Carotid IMT scores are the strongest independent cardiovascular biomarker for cognitive function, especially complex information processing. Greater intake of fiber, long-chain n-3 fatty acids (N3FAs), and vitamin B12, as well as measures of aerobic fitness, is associated with enhanced cognitive function, yet controlling for IMT scores diminished their association. Because decreasing CIP scores are linearly associated with cognitive decline, future randomized clinical trials that yield improvements in carotid IMT scores should also assess for changes in cognitive function. © 2014 Copyright Taylor and Francis Group, LLC.
PMID
24533609 [http://www.ncbi.nlm.nih.gov/pubmed/?term=24533609]
Institution
(Masley, Masley) Masley Optimal Health Center, University of South Florida, St. Petersburg, FL, United States (Gualtieri) North Carolina Neuropsychiatry Clinics, Chapel Hill, Charlotte and Raleigh, North Carolina, United States
Publisher
Routledge (325 Chestnut Street, Philadelphia PA 19106, United States)
Emtree Heading
adult; aerobic capacity; aged; *arterial wall thickness; article; biometry; body mass; *cardiovascular risk; carotid artery; *cognition; cross-sectional study; dietary intake; electrocardiography; executive function; female; human; lung gas exchange; male; memory; nutrition; retrospective study; risk factor; caffeine; cholesterol; cyanocobalamin; folic acid; glucose; homocysteine; long chain fatty acid.
Drug Index Terms
caffeine; cholesterol; cyanocobalamin; folic acid; glucose; homocysteine; long chain fatty acid.
Other Index Terms
adult; aerobic capacity; aged; *arterial wall thickness; article; biometry; body mass; *cardiovascular risk; carotid artery; *cognition; cross-sectional study; dietary intake; electrocardiography; executive function; female; human; lung gas exchange; male; memory; nutrition; retrospective study; risk factor.

Link to the Ovid Full Text or citation:

For peer review only - http://bmjopen.bmj.com/site/about/guidelines.xhtml

Click here for full text options

Link to the External Link Resolver:

SFX

168.

Chronic cerebral hypoperfusion and dementia.

Yata K., Tomimoto H.

Neurology and Clinical Neuroscience. 2 (5) (pp 129-134), 2014. Date of Publication: 01 Sep 2014.

AN: 600173277

"Cerebral small vessel disease" is a general term featuring a group of disease conditions with characteristic lesions affecting mainly small vessels in the brain, such as Binswanger's disease, leukoaraiosis and lacunar infarctions. Cerebral small vessels consist of a series of blood vessels, which originate from the pial arteries on the surface of the brain, and branch into arterioles, capillaries and postcapillary venules. Each of the blood vessels has a distinct structure and function. The blood-brain barrier, which does not exist in the other organs, functions in the brain. Dysfunction of the blood-brain barrier is thought to be a major cause of cerebral small vessel diseases. Recent findings have shown that maintenance of the blood-brain barrier is kept by various types of cells, such as vascular endothelial cells, astrocytes and pericytes, which work collaboratively as a neurovascular unit. Currently, larger vessels at the arteriolar level have been studied intensively; however, the pathological condition of the neurovascular unit at the capillary level still needs to be elucidated. The bilateral carotid artery stenosis model simulates chronic cerebral hypoperfusion, formation of white matter lesions and cognitive impairments seen in humans. Using this model, we found microcirculation disturbance especially in the postcapillary venule, and postulated it as a final step leading to white matter lesions and cognitive impairment. Taken together, we suggest that chronic cerebral hypoperfusion plays a pivotal role in the pathogenesis of cerebral small vessel diseases.

Copyright © 2014 Japanese Society of Neurology and Wiley Publishing Asia Pty Ltd.

Institution

(Yata, Tomimoto) Department of Neurology, Mie University Graduate School of Medicine, Tsu, Japan

Publisher

Blackwell Publishing Ltd (E-mail: customerservices@oxonblackwellpublishing.com)

For peer review only - http://bmjopen.bmj.com/site/about/guidelines.xhtml

Emtree Heading

Alzheimer disease; arteriolosclerosis; arteriosclerosis; atherosclerosis; blood brain barrier; brain capillary; *brain perfusion; carotid artery obstruction; cerebrovascular disease; cognitive defect; *dementia; human; meningeal artery; microcirculation; multiinfarct dementia; nonhuman; pia artery; priority journal; review; venule; white matter lesion; lipohyalinosis.

Candidate Terms

lipohyalinosis [other term].

Other Index Terms

Alzheimer disease; arteriolosclerosis; arteriosclerosis; atherosclerosis; blood brain barrier; brain capillary; *brain perfusion; carotid artery obstruction; cerebrovascular disease; cognitive defect; *dementia; human; meningeal artery; microcirculation; multiinfarct dementia; nonhuman; pia artery; priority journal; Review; venule; white matter lesion.

Link to the Ovid Full Text or citation:

Click here for full text options

Link to the External Link Resolver:

SFX

175.

Visit-to-visit blood pressure variability in the elderly: Associations with cognitive impairment and carotid artery remodeling.

Nagai M., Hoshide S., Nishikawa M., Masahisa S., Kario K.

Atherosclerosis. 233 (1) (pp 19-26), 2014. Date of Publication: March 2014.

AN: 372362301

Objective: Recently, visit-to-visit blood pressure (BP) variability has been shown to be associated with vascular remodeling and cognitive dysfunction. However, there have been no studies that focused on the relationship between visit-to-visit BP variability and cognitive dysfunction in relation to vascular remodeling. In this study, we investigated the relationships among visit-to-visit BP measures, carotid artery remodeling and cognitive function in the elderly at high risk of cardiovascular disease.

For peer review only

Method(s): The cognitive function was evaluated using a Mini-Mental State Examination (MMSE) and global deterioration scale (GDS) in 201 elderly subjects at high risk of cardiovascular disease (79.9+/-6.4 years old; female 75%). Based on 12 visits (once a month), visit-to-visit BP variability (expressed as the coefficient of variation [CV] and as delta [maximum-minimum] BP) were measured. Carotid ultrasound was performed to measure intima-media thickness (IMT) and the stiffness parameter beta.

Result(s): The patients having both high delta systolic BP (SBP) and high IMT had significantly higher prevalence of low MMSE score than those with both low delta SBP and low IMT ($p<0.05$), and the patients having both high delta SBP and high stiffness parameter beta also had significantly higher prevalence of low MMSE score than those with both low delta SBP and low stiffness parameter beta ($p<0.01$). In the logistic regression analysis adjusted for age, calcium channel blocker use, low density lipoprotein, average heart rate, and average SBP level, a significant interaction was found between delta SBP and stiffness parameter beta for the low MMSE score ($p<0.05$).

Conclusion(s): In the high risk elderly, exaggerated visit-to-visit BP variability and advanced carotid artery remodeling have a synergetic association with cognitive dysfunction. © 2013 Elsevier Ireland Ltd.

PMID

24529116 [http://www.ncbi.nlm.nih.gov/pubmed/?term=24529116]

Institution

(Nagai, Hoshide, Nishikawa, Masahisa, Kario) Division of Cardiovascular Medicine, Department of Medicine, Jichi Medical University School of Medicine, Yakushiji 3311-1, Shimotsuke, Tochigi 329-0498, Japan

Publisher

Elsevier Ireland Ltd (P.O. Box 85, Limerick, Ireland)

Emtree Heading

aged; arterial wall thickness; article; *blood pressure variability; blood sampling; cardiovascular disease; cardiovascular risk; carotid atherosclerosis; cholesterol blood level; clinical assessment tool; clinical feature; cognition; *cognitive defect; common carotid artery; cross-sectional study; disease association; echography; female; heart rate; human; hypertension; major clinical study; male; Mini Mental State Examination; prevalence; priority journal; rigidity; systolic blood pressure; calcium channel blocking agent; cholesterol/ec [Endogenous Compound]; glucose/ec [Endogenous Compound]; hemoglobin A1c/ec [Endogenous Compound]; high density lipoprotein/ec [Endogenous Compound]; low density lipoprotein/ec [Endogenous Compound]; triacylglycerol/ec [Endogenous Compound]; Global deterioration scale; *visit to visit blood pressure variability.

Candidate Terms

global deterioration scale [other term]; *visit to visit blood pressure variability [other term].

For peer review only

Drug Index Terms

calcium channel blocking agent; cholesterol / endogenous compound; glucose / endogenous compound; hemoglobin A1c / endogenous compound; high density lipoprotein / endogenous compound; low density lipoprotein / endogenous compound; triacylglycerol / endogenous compound.

Other Index Terms

aged; arterial wall thickness; article; *blood pressure variability; blood sampling; cardiovascular disease; cardiovascular risk; carotid atherosclerosis; cholesterol blood level; clinical assessment tool; clinical feature; cognition; *cognitive defect; common carotid artery; cross-sectional study; disease association; echography; female; heart rate; human; hypertension; major clinical study; male; Mini Mental State Examination; prevalence; priority journal; rigidity; systolic blood pressure.

Link to the Ovid Full Text or citation:

Click here for full text options

Link to the External Link Resolver:

SFX

178.

Carotid plaque echolucency is associated with poor cognitive performance and future cognitive decline in patients with atherosclerotic disease.

Mastroiacovo D., Camerota A., Pinelli M., De Blasis G., Turco G.L., Andriulli M., Cipollone F., Raffaele A., Lechiara M.C., Grassi D., Necozione S., Marini C., Ferri C., Desideri G.

High Blood Pressure and Cardiovascular Prevention. Conference: 31st National Congress of the Italian Society of Hypertension, SIIA 2014. Bologna Italy. Conference Publication: (var.pagings). 21 (4) (pp 328-329), 2014. Date of Publication: December 2014.

AN: 71990762

Introduction. Carotid plaque echolucency predicts both overt and silent cerebrovascular disease which in turn can affect cognition. Aim. This study evaluated, in a population-based, prospective design, whether carotid plaque morphology is associated with cognitive dysfunction and future cognitive decline. Methods. Grey-scale median (GSM), a computer-assisted index of echogenicity, was evaluated in carotid plaques in 113 subjects (72.6 +/- 5.6 years) with carotid atherosclerosis but

For peer review only - http://bmjopen.bmj.com/site/about/guidelines.xhtml

free of overt cerebrovascular disease or dementia. Cognitive functions were assessed by mini mental state examination, trail making test (TMT) A and B, and verbal fluency test (VFT). Fifty-five subjects were re-evaluated after a mean follow-up period of 4.27 years. Results. At baseline median, GSM was significantly associated with TMTA (r:-0.454, p<0.0001), TMTB (r:-0.429, p<0.0001), TMTB-A (r:-0.239, p = 0.011) and VFT (r:0.299, p = 0.001) scores, while no correlations were found between metabolic parameters, blood pressure and cognitive performance. During the follow-up period the median GSM did not significantly change (-0.145, p = 0.758). With regard to cognitive performances, a slight but significant worsening of TMTB (+26.09 s, p = 0.05) and TMTB-A (+26.25 s, p = 0.05) score was observed while neither MMSE (-0.2, p = 0.808) nor VFT (+1.0 words, p = 0.239) changed. Baseline median GSM was significantly associated with changes of TMTB (r:- 0.341, p = 0.011) and TMTB-A (r:-0.379, p = 0.004) during follow-up. No correlations were found between changes of metabolic parameters and blood pressure and variations of neuropsychological test scores. Conclusions. Carotid plaque echolucency is associated with poor cognitive performance and future cognitive decline in elderly subjects. Assessment of carotid plaque morphology may help identify subjects at increased risk for cognitive dysfunction.

Institution

(Mastroiacovo, Pinelli, Grassi, Desideri) Angiology Unit, SS. Filippo E Nicola Hospital, L'Aquila, Italy

(Camerota, Andriulli, Necozione, Marini, Ferri) Department of Life, Health and Environmental Sciences, University of L'Aquila, L'Aquila, Italy

(De Blasis, Turco) Vascular Surgery Unit, SS. Filippo E Nicola Hospital, L'Aquila, Italy

(Cipollone) European Center of Excellence on Atherosclerosis, Hypertension and Dyslipidemia, Clinical Research Center, G. D'Annunzio University, Chieti-Pescara, Italy

(Raffaele, Lechiara, Desideri) Geriatric Unit, SS. Filippo E Nicola Hospital, L'Aquila, Italy

Publisher

Springer International Publishing

Emtree Heading

*carotid artery; *patient; *human; *society; *hypertension; follow up; cerebrovascular disease; cognitive defect; morphology; blood pressure; cognition; metabolic parameters; Mini Mental State Examination; computer; trail making test; dementia; population; carotid atherosclerosis; risk; neuropsychological test; aged.

Other Index Terms

*carotid artery; *patient; *human; *society; *hypertension; follow up; cerebrovascular disease; cognitive defect; morphology; blood pressure; cognition; metabolic parameters; Mini Mental State Examination; computer; trail making test; dementia; population; carotid atherosclerosis; risk; neuropsychological test; aged.

For peer review only - http://bmjopen.bmj.com/site/about/guidelines.xhtml

Link to the Ovid Full Text or citation:

Click here for full text options

Link to the External Link Resolver:

SFX

180.

Arterial thickening and stiffness and cognitive function: Singapore longitudinal ageing studies.
Lim S.L., Gao Q., Ling L.H., Ng T.P.
Annals of the Academy of Medicine Singapore. Conference: NUHS Academic Psychiatry Conference 2014. Singapore Singapore. Conference Publication: (var.pagings). 43 (10 SUPPL. 1) (pp S55), 2014. Date of Publication: October 2014.
AN: 71798731

Introduction Cardiovascular ageing exacerbated by cardiovascular risk factors is marked by increased carotid intima-media thickness (IMT) and arterial stiffness. Previous studies suggested these vascular markers could be associated with cognitive decline and cognitive impairment. Objective In this population-based study, we determined the association between markers of vascular health and cognitive performance, and explored the differential associations of specific vascular indices on individual cognitive domains. Methods Cross-sectional analysis of 354 participants from the community-based sample of older persons without heart failure who were controls in the Singapore Heart Failure Outcomes and Phenotypes (SHOP) study. Cognitive function was measured by the Mini-Mental State Examination (MMSE) and a comprehensive neuropsychological test battery that assessed a wide range of cognitive domains. Arterial stiffness was measured by pulse wave velocity (PWV), augmentation index (AI), elasticity modulus (Ep), beta stiffness (beta) and arterial compliance (AC). Arterial thickening was measured by carotid IMT. Multiple linear regressions were performed with controlling of potential confounders (age, gender, education, and cardiovascular risk factors: hypertension, diabetes, dyslipidemia, smoking and BMI). Results The mean age of the study participants was 63.3 +/- 6.1 years, 45.2% were females and 39.3% had 6 or less years of education. In the total sample of 354 participants, there was a significant association between Ep and MMSE (beta = -0.127, P = 0.017) in the multivariate analysis. In the subgroup of 170 subjects with detailed neuropsychological data, central AI was associated with verbal memory domain after adjustment for cardiovascular risk factors (beta = -0.231, P = 0.002). All indices of arterial stiffness were associated with executive function in the unadjusted

For peer review only - http://bmjopen.bmj.com/site/about/guidelines.xhtml

model, with higher stiffness associated with longer time taken to complete colour trails test [Ep (beta = 0.244, P = 0.002), beta (beta = 0.24, P = 0.002), peripheral AI (beta = -0.169, P = 0.031), femoralcarotid PWV (beta = 0.311, P <0.001) and Aix (beta = 0.183, P = 0.019)]. Following adjustment for demographics and education, only femoral-carotid PWV remained significantly associated with executive function, albeit attenuated (beta = 0.151, P = 0.044). This association was not significant following adjustment for cardiovascular risk factors. Conclusion Different indices of vascular health were associated with different aspects of cognitive performance. High carotid stiffness was associated with impaired global cognition, aortic stiffness was inversely associated with verbal memory, and endothelial dysfunction was associated with reduced visuospatial ability. A larger study is necessary to confirm and refine our findings. In addition, extending this into a longitudinal study would allow us to correlate these indices of vascular health with the trajectories of cognitive decline, if any.

Institution

(Lim, Ling) Department of Cardiology, National University Hospital, National University Health System, Singapore, Singapore (Gao, Ng) Department of Psychological Medicine, National University Hospital, National University Health System, Singapore, Singapore

Publisher

Academy of Medicine Singapore

Emtree Heading

*rigidity; *cognition; *Singapore; *aging; *psychiatry; *arterial wall thickening; cardiovascular risk; human; arterial stiffness; carotid artery; health; education; verbal memory; heart failure; executive function; Mini Mental State Examination; pulse wave; smoking; neuropsychological test; multivariate analysis; augmentation index; cognitive defect; dyslipidemia; diabetes mellitus; hypertension; gender; phenotype; multiple linear regression analysis; community; artery compliance; female; longitudinal study; color; model; elasticity; population; endothelial dysfunction; arterial wall thickness; marker.

Drug Index Terms

marker.

Other Index Terms

*rigidity; *cognition; *Singapore; *aging; *psychiatry; *arterial wall thickening; cardiovascular risk; human; arterial stiffness; carotid artery; health; education; verbal memory; heart failure; executive function; Mini Mental State Examination; pulse wave; smoking; neuropsychological test; multivariate analysis; augmentation index; cognitive defect; dyslipidemia; diabetes mellitus; hypertension; gender; phenotype; multiple linear regression analysis; community; artery compliance; female; longitudinal study; color; model; elasticity; population; endothelial dysfunction; arterial wall thickness.

For peer review only

Link to the Ovid Full Text or citation:
Click here for full text options

Link to the External Link Resolver:
SFX

181.

The influence of dyslipidemia on cognitive function in patients with occlusive carotid disease.
Ogata T., Watanabe J., Kimura S., Oma S., Inoue T., Tsuboi Y.
Cerebrovascular Diseases. Conference: Annual Conference of the Asia Pacific Stroke Organization, APSO 2014. Taipei Taiwan (Republic of China). Conference Publication: (var.pagings). 38 (SUPPL. 1) (pp 71), 2014. Date of Publication: September 2014.
AN: 71662042
Background and Objectives: The objective of the current study was to elucidate the influence of dyslipidemia on cognitive function in patients with carotid stenosis or occlusion.
Method(s): We prospectively registered the patients with carotid occlusion or stenosis of over 40 percent in the Fukuoka University Hospital between 2011 and 2013. The patients with Alzheimer disease or distinctive dysfunction of higher brain were excluded from this study. The Mini-mental state examination (MMSE) was used to estimate the cognitive function and the score of MMSE of less than 27 was defined as subnormal. Also, the risk factors of atherosclerosis, educational age, the degree of carotid stenosis, cerebral blood flow, the presence or absence of white matter lesion and microbleeds were evaluated. The LDL- and HDL-cholesterol level and the treatment of statin were reviewed and the associations of MMSE with LDL- and HDL-cholesterol level and statin use were assessed. Using the multivariate analysis, the factors associated with the subnormal cognitive function was tested.
Result(s): A total of 115 patients were registered (Age: 71.5+/-6.8 years, male: 95 cases, female: 20 cases). The patients with statin (administration or use) scored higher MMSE (median: 27) compared with those without (median: 26, P = 0.025). HDL-cholesterol level was positively correlated with MMSE score. Multivariate analysis indicated that the nonuse of statin was significantly associated with the subnormal score of MMSE (Odds ratio: 0.33, P = 0.031), as well as lower educational age, male sex and the presence of diabetes.

For peer review only - http://bmjopen.bmj.com/site/about/guidelines.xhtml

Conclusion(s): In the patients with carotid occlusive disease, the treatment of statin was associated with higher score of MMSE than without. The nonuse of statin led to subnormal cognitive function. Statin might benefit for maintaining cognitive function in patients with carotid disease probably due to its protective effect of endothelium of artery or preventative effect of Alzheimer pathology.

Institution

(Ogata, Kimura, Oma, Tsuboi) Department of Neurology, Fukuoka University, Japan (Watanabe) Department of Rehabilitation Medicine, Fukuoka University Hospital, Japan

(Inoue) Department of Neurosurgery, Fukuoka University, Japan

Publisher

S. Karger AG

Emtree Heading

*dyslipidemia; *cognition; *patient; *human; *carotid artery; *Asia; *cerebrovascular accident; Mini Mental State Examination; carotid artery obstruction; multivariate analysis; male; implantable cardioverter defibrillator; Alzheimer disease; brain; university hospital; endothelium; stenosis; diabetes mellitus; risk; occlusion; white matter lesion; brain blood flow; atherosclerosis; artery; pathology; risk factor; female; statin (protein); high density lipoprotein cholesterol; high density lipoprotein; low density lipoprotein.

Drug Index Terms

statin (protein); high density lipoprotein cholesterol; high density lipoprotein; low density lipoprotein.

Other Index Terms

*dyslipidemia; *cognition; *patient; *human; *carotid artery; *Asia; *cerebrovascular accident; Mini Mental State Examination; carotid artery obstruction; multivariate analysis; male; implantable cardioverter defibrillator; Alzheimer disease; brain; university hospital; endothelium; stenosis; diabetes mellitus; risk; occlusion; white matter lesion; brain blood flow; atherosclerosis; artery; pathology; risk factor; female.

Link to the Ovid Full Text or citation:

Click here for full text options

Link to the External Link Resolver:

SFX

For peer review only

184.

Carotid inflammation is associated with white matter disease and cognitive impairment post stroke.
Goh O., Yeong Poh Y., Singh R., Kandiah N.
Alzheimer's and Dementia. Conference: Alzheimer's Association International Conference 2014. Copenhagen Denmark. Conference Publication: (var.pagings). 10 (SUPPL. 4) (pp P843), 2014. Date of Publication: July 2014.
AN: 71628927
Background: Cognitive impairment in post small-vessel stroke patients has been demonstrated to be associated with the burden of white matter disease (WMD), location and distribution of acute infarcts, inflammation and demyelination. The role of inflammation in the pathogenesis of cognitive impairment and WMD in post-stroke patients has not been adequately studied. There is evidence to demonstrate that carotid intima media thickness (IMT) is associated with markers of inflammation. This study examines the relationship between carotid IMT, cognitive function and WMD in patients following acute lacunar stroke.
Method(s):We studied consecutive patients with MRI-confirmed acute lacunar infarcts at a tertiary neurology center between September 2009 and March 2010. Patients with acute cerebral hemorrhage, neuroimaging evidence of large-vessel strokes, and potential embolic etiology were excluded. All patients had carotid IMT determined by ultrasonography.WMH was scored using the modified Fazekas scale on axial T2-weighted images. The degree of carotid IMT was divided into tertiles based on side with the higher IMT. The association between carotid IMT, cognitive impairment and WMD was modeled using logistic regression analysis.
Result(s): 100 patients with a mean age of 56.2 +/- 11.7 years were studied. The tertile with the greatest carotid IMT had a mean IMT of 0.86 whereas the tertile with the smallest carotid IMT had a mean of 0.49. MMSEwas lowest (27.58) in the highest carotid IMT tertile, and this association was approaching significance (p=0.0939). Total periventricular WMD was also highest (2.42) in the tertile with the highest IMT (p=0.0787). Logistic regression analyses showed an odds ratio (OR) 36.12, (confidence interval 1.91-682.51;p=0.017) for the association between periventricular WMD and IMT.
Conclusion(s): Carotid inflammation evidenced by IMT is an important risk factor for WMD and cognitive impairment post stroke. The role of anti-inflammatory markers among post-stroke patients warrants further studies.
Institution
(Goh, Singh, Kandiah) National Neuroscience Institute, Singapore, Singapore (Yeong Poh) Duke - National University of Singapore, Graduate Medical School, National Neuroscience Institute, Singapore, Singapore
Publisher

For peer review only - http://bmjopen.bmj.com/site/about/guidelines.xhtml

Elsevier Inc.
Emtree Heading
*carotid artery; *inflammation; *white matter; *cognitive defect; *cerebrovascular accident; human; patient; stroke patient; infarction; logistic regression analysis; etiology; neuroimaging; brain hemorrhage; neurology; lacunar stroke; cognition; demyelination; confidence interval; arterial wall thickness; risk; pathogenesis; risk factor; nuclear magnetic resonance imaging; marker.
Drug Index Terms
marker.
Other Index Terms
*carotid artery; *inflammation; *white matter; *cognitive defect; *cerebrovascular accident; human; patient; stroke patient; infarction; logistic regression analysis; etiology; neuroimaging; brain hemorrhage; neurology; lacunar stroke; cognition; demyelination; confidence interval; arterial wall thickness; risk; pathogenesis; risk factor; nuclear magnetic resonance imaging.

Link to the Ovid Full Text or citation:
Click here for full text options

Link to the External Link Resolver:
SFX

185.

Cerebrovascular atherosclerosis and cognition in patients with Alzheimer's disease.
Jang E.H., Lee E.H., Park E.K., Roh J.H., Lee J.-H.
Alzheimer's and Dementia. Conference: Alzheimer's Association International Conference 2014. Copenhagen Denmark. Conference Publication: (var.pagings). 10 (SUPPL. 4) (pp P377), 2014. Date of Publication: July 2014.
AN: 71627735
Background: Vascular risk factors are known to be associated with increased prevalence of Alzheimer's disease (AD) and poorer outcome of the disease. Most studies investigating, the relationship between the cerebrovascular diseases and AD focused on small vessel disease, however, the relationship between the large vessel disease and AD are relatively sparse. The objectives of this study were to compare the degree of atherosclerosis markers of large vessels

For peer review only

between AD patients and healthy controls and to investigate the relationship between these markers and neuropsychological test performances in patients with AD.
Method(s): A total of sixtythree AD patients, aged 55 to 90 (mean age 73.6 years, 41 women and 22 men), underwent carotid duplex sonography and magnetic resonance angiography (MRA).All the participants completed detailed neuropsychological evaluation with Seoul neuropsychological screening battery (SNSB) which examined attention, language and related functions, visuospatial functions, verbal and visual memory, frontal and executive functions, Korean version of mini-mental status examination (K-MMSE) and clinical dementia rating scale.A total of eighty-six healthy controls (mean age 60.7 years, 19 women and 67 men) who completed carotid duplex sonography were recruited for comparison. The severity of cerebrovascular atherosclerosis markers were measured by carotid plaque-score, intima-media thickness (IMT), and intracranial atherosclerotic stenosis. Statistical analyses were performed using SPSS version 21.0.
Result(s): The plaque-score of carotid artery (1.35 +/- 1.20 vs. 0.66 +/- 0.91) was significantly higher in patients with AD than healthy control (p=0.023) after adjustment of age, sex, hypertension, diabetes mellitus, and IMT. In AD patients, the presence of carotid plaque was associated with a lower Seoul Verbal Learning Test (SVLT)immediate recall score (p=0.026) and a lower Controlled Oral Word Association Test (COWAT) phonemic test score (p=0.044). Moreover, carotid IMTwas associated with higher error rates (p=0.027) and lower correction rates (p=0.018) of the Stroop-word test in AD patients. A severity of intracranial arterial stenosis was associated with poorer performances on SVLT immediate recall p=0.002), SVLT delayed recall (p=0.013), Ray Complex Figure Test (RCFT) immediate recall (p=0.000), RCFT delayed recall (p=0.000) and Stroop-color test correction rate (p=0.007).
Conclusion(s): The presence of carotid plaque is more common in AD patients than healthy controls. Carotid atherosclerosis influenced frontal executive functions, and intracranial atherosclerotic stenosis was associated with memory functions in AD patients. These findings suggest that large artery disease may impact on cognitive decline in patients with AD. Future studies are needed to clarify the mechanisms of how large artery disease interacts with AD and affects cognitive decline.
Institution
(Jang, Lee, Park, Roh, Lee) Asan Medical Center, Seoul, South Korea
Publisher
Elsevier Inc.
Emtree Heading
*human; *atherosclerosis; *cognition; *patient; *Alzheimer disease; carotid artery; recall; echography; stenosis; female; artery disease; executive function; male; language; screening; cerebrovascular disease; magnetic resonance angiography; prevalence; hypertension; learning test; artery occlusion; carotid atherosclerosis; statistical analysis; arterial wall thickness; Clinical Dementia

For peer review only - http://bmjopen.bmj.com/site/about/guidelines.xhtml

Rating; examination; mental health; task performance; risk factor; diabetes mellitus; learning; neuropsychological test; visual memory; personality test; color; memory; data analysis software; Mini Mental State Examination; marker.

Drug Index Terms

marker.

Other Index Terms

*human; *atherosclerosis; *cognition; *patient; *Alzheimer disease; carotid artery; recall; echography; stenosis; female; artery disease; executive function; male; language; screening; cerebrovascular disease; magnetic resonance angiography; prevalence; hypertension; learning test; artery occlusion; carotid atherosclerosis; statistical analysis; arterial wall thickness; Clinical Dementia Rating; examination; mental health; task performance; risk factor; diabetes mellitus; learning; neuropsychological test; visual memory; personality test; color; memory; data analysis software; Mini Mental State Examination.

Link to the Ovid Full Text or citation:

Click here for full text options

Link to the External Link Resolver:

SFX

186.

Carotid intima thickness and cognitive function in middle-age adults.

Al Hazzouri A.Z., Jacobs D., Vittinghoff E., Reis J., Yaffe K., Sidney S.

Alzheimer's and Dementia. Conference: Alzheimer's Association International Conference 2014. Copenhagen Denmark. Conference Publication: (var.pagings). 10 (SUPPL. 4) (pp P202), 2014. Date of Publication: July 2014.

AN: 71627269

Background: Carotid Intima Media thickness (IMT) is a marker of atherosclerosis and strongly related to vascular risk factors associated with cognitive impairment.We propose to determine if site-specific IMT is associated with cognitive performance invariousdomains measured in midlife. Method(s): We determined the association between IMTand cognitive function 5 years later using data from the Coronary Artery Risk Development in Young Adults (CARDIA) study, a young to

For peer review only

middle-aged cohort of white and black adults. At the year 20 follow-up exam(study baseline), participants underwent ultrasound studies to image the carotid artery resulting in site-specific IMT measures: common carotid artery (CCA) IMT, internal carotid artery (ICA) IMT and ICA/bulb IMT. Five years later, participants underwent a cognitive battery consisting of 3 tests: the Rey Auditory-Verbal Learning Test (RAVLT, range 0 to15), a test of verbal memory; the Digit Symbol Substitution Test (DSST, range 8 to 119), a test of speed and working memory; and the Stroop (range -46 to 127), a test of executive skills. We excluded subjects with reported baseline coronary heart disease (stroke, angina, heart attack) for a total of 2,576 participants included in this analysis (mean age 45.3 years, 43% blacks and 42.7%males).

Result(s): Being male, black, less educated, a smoker and having more co-morbidities such as higher BMI, hypertension, and type-2 diabetes was associated with having higher site-specific IMT from bivariate regressions. In linear regression models adjusted for socio-demographics (age, sex, race and education) and behavioral risk factors and comorbidities (alcohol, smoking, BMI, hypertension, diabetes, depression), 1 standard deviation difference in CCA IMT (0.123mm) was associated with 0.81 lower points (worse performance) on the DSST (p=0.006) and 0.41 higher points (worse performance) on the Stroop (p=0.05) but not on RAVLT. After adjustment for covariates, IMTof other sites of the carotid artery were not associated with cognitive performance.

Conclusion(s): Having higher common carotid IMT, reflective of early adulthood burden of atherosclerosis, was associated with worse cognitive performance in midlife. Atherosclerosis may begin as early as in childhood; thus, interventions in earlier years may delay or prevent cognitive impairment.

Institution

(Al Hazzouri, Vittinghoff, Yaffe) University of California San Francisco, San Francisco, CA, United States (Jacobs) University of Minnesota, Minneapolis, MN, United States

(Reis) NIH, Bethesda, MD, United States

(Sidney) Kaiser Permanente, Oakland, CA, United States

Publisher

Elsevier Inc.

Emtree Heading

*carotid artery; *intima; *thickness; *cognition; *middle aged; *adult; human; atherosclerosis; male; risk factor; hypertension; smoking; coronary artery; internal carotid artery; common carotid artery; heart infarction; ultrasound; angina pectoris; morbidity; non insulin dependent diabetes mellitus; learning; learning test; cerebrovascular accident; follow up; manager; working memory; skill; ischemic heart disease; velocity; childhood; arterial wall thickness; digit symbol substitution test; young adult; verbal memory; linear regression analysis; model; education; risk; diabetes mellitus; adulthood; cognitive defect; marker; alcohol.

Drug Index Terms

For peer review only - http://bmjopen.bmj.com/site/about/guidelines.xhtml

marker; alcohol.

Other Index Terms

*carotid artery; *intima; *thickness; *cognition; *middle aged; *adult; human; atherosclerosis; male; risk factor; hypertension; smoking; coronary artery; internal carotid artery; common carotid artery; heart infarction; ultrasound; angina pectoris; morbidity; non insulin dependent diabetes mellitus; learning; learning test; cerebrovascular accident; follow up; manager; working memory; skill; ischemic heart disease; velocity; childhood; arterial wall thickness; digit symbol substitution test; young adult; verbal memory; linear regression analysis; model; education; risk; diabetes mellitus; adulthood; cognitive defect.

Link to the Ovid Full Text or citation:

Click here for full text options

Link to the External Link Resolver:

SFX

187.

Carotid intima thickness and cognitive function in middle-age adults.

Al Hazzouri A.Z., Vittinghoff E., Sidney S., Reis J., Jacobs D., Yaffe K.

Alzheimer's and Dementia. Conference: Alzheimer's Association International Conference 2014. Copenhagen Denmark. Conference Publication: (var.pagings). 10 (SUPPL. 4) (pp P182), 2014. Date of Publication: July 2014.

AN: 71627225

Project Description: Carotid Intima Media thickness (IMT) is a marker of atherosclerosis and strongly related to vascular risk factors associated with cognitive impairment. We propose to determine if site-specific IMT is associated with cognitive performance in various domains measured in midlife. We determined the association between IMT and cognitive function 5 years later using data from the Coronary Artery Risk Development in Young Adults (CARDIA) study, a young to middle- aged cohort of white and black adults. At the year 20 follow-up exam (study baseline), participants underwent ultrasound studies to image the carotid artery resulting in site-specific IMT measures: common carotid artery (CCA) IMT, internal carotid artery (ICA) IMT and ICA/bulb IMT. Five years later, participants underwent a cognitive battery consisting of 3 tests: the

For peer review only - http://bmjopen.bmj.com/site/about/guidelines.xhtml

Rey Auditory-Verbal Learning Test (RAVLT, range 0 to15), a test of verbal memory; the Digit Symbol Substitution Test (DSST, range 8 to 119), a test of speed and working memory; and the Stroop (range -46 to 127), a test of executive skills. We excluded subjects with reported baseline coronary heart disease (stroke, angina, heart attack) for a total of 2,576 participants included in this analysis (mean age 45.3 years, 43% blacks and 42.7%males). Being male, black, less educated, a smoker and having more co-morbidities such as higher BMI, hypertension, and type-2 diabetes was associated with having higher site-specific IMT from bivariate regressions. In linear regression models adjusted for socio-demographics (age, sex, race and education) and behavioral risk factors and co-morbidities (alcohol, smoking, BMI, hypertension, diabetes, depression), 1 standard deviation difference in CCA IMT (0.123mm) was associated with 0.81 lower points (worse performance) on the DSST (p=0.006) and 0.41 higher points (worse performance) on the Stroop (p=0.05) but not on RAVLT. After adjustment for covariates, IMT of other sites of the carotid artery were not associated with cognitive performance. Higher common carotid IMT was associated with worse cognitive performance in midlife, among a cohort free of coronary heart disease. IMT, reflective of early adulthood burden of atherosclerosis, may begin as early as in childhood; thus, interventions in earlier years may delay or prevent cognitive impairment.

Institution
(Al Hazzouri, Yaffe) University of California San Francisco, San Francisco, CA, United States
(Vittinghoff) UCSF, San Francisco, CA, United States
(Sidney) Kaiser Permanente Division of Research, Oakland, CA, United States
(Reis) National Heart,Lung,and Blood Institute, Bethesda, MD, United States
(Jacobs) University of Minnesota, Minneapolis, MN, United States

Publisher
Elsevier Inc.

Emtree Heading

*carotid artery; *intima; *thickness; *cognition; *middle aged; *adult; human; smoking; cognitive defect; atherosclerosis; risk factor; morbidity; hypertension; ischemic heart disease; male; internal carotid artery; common carotid artery; heart infarction; ultrasound; coronary artery; non insulin dependent diabetes mellitus; linear regression analysis; follow up; cerebrovascular accident; working memory; manager; skill; velocity; young adult; angina pectoris; digit symbol substitution test; adulthood; risk; arterial wall thickness; verbal memory; learning test; education; model; learning; diabetes mellitus; childhood; marker; alcohol.

Drug Index Terms

marker; alcohol.

Other Index Terms

*carotid artery; *intima; *thickness; *cognition; *middle aged; *adult; human; smoking; cognitive defect; atherosclerosis; risk factor; morbidity; hypertension; ischemic heart disease; male; internal

For peer review only

For peer review only - http://bmjopen.bmj.com/site/about/guidelines.xhtml

carotid artery; common carotid artery; heart infarction; ultrasound; coronary artery; non insulin dependent diabetes mellitus; linear regression analysis; follow up; cerebrovascular accident; working memory; manager; skill; velocity; young adult; angina pectoris; digit symbol substitution test; adulthood; risk; arterial wall thickness; verbal memory; learning test; education; model; learning; diabetes mellitus; childhood.

Link to the Ovid Full Text or citation:
Click here for full text options

Link to the External Link Resolver:
SFX

192.

Association of inflammatory markers and intima - Media thickness in Mild Cognitive Impairment.
Coteanu C., Gusti S., Coteanu A.
Archives of the Balkan Medical Union. 48 (2) (pp 192-195), 2013. Date of Publication: June 2013.
AN: 369606574
Background: The current definition of Mild Cognitive Impairment (MCI) is a term which characterizes either mild impairment or very early dementia, a intermediate state between the cognitive changes of normal cognitive aging and dementia. The plasma levels of inflammatory factors have an important role in the pathogenesis of cognitive impairments. The aim of this study was to assess the asodation between plasma fibrinogen levels and plasma C-reactive protein levels with intima-media thickness and cognitive decline to people with mild cognitive impairment
Methods: We admitted in the study twenty-five patients with mild cognitive impairment, aged over 60. The inflammatory markers (plasma CRP and fibrinogen levels) and carotid IMT were measured at baseline and during follow-up at an interval of 6 to 12 months. The global cognitive functions were assessed at baseline and during follow-up periodically with Mini Mental State Examination (MMSE).
Result(s): In our study high CRP levels were significantly correlated with MMSE score and carotid IMT significantly increased according to the quartiles of CRP in both genders. We observed a significant association between hyperfibrinogenaemia and MMSE score as proven by chi-square

For peer review only

test and Spearman's coefficient. We say that hyperfibrinogenaemia and high CRP levels correlated with a raised IMT values are statistically significant in patients with MCI.
Conclusion(s): It can be concluded that plasma fibrinogen level and high plasma CRP level may be associated with IMT and cognitive decline. Our findings are consistent with the hypothesis that plasma levels of inflammatory factors have an important role in the pathogenesis of cognitive impairments. Abbreviations: CDR - Clinical Dementia Rating; CRP - C-reactive protein; GDS - Global Deterioration Scale; IMT - Intima-media thickness; MCI - Mild Cognitive Impairment; MMSE - Mini Mental State Examination.

Institution

(Coteanu) Department of Clinical Laboratory, Filantropia Hospital, Municipal Clinic, Constantin Brancusi street, no. 3, Craiova, Romania (Gusti) Department of Physiology, University of Medicine and Pharmacy of Craiova, Romania
(Coteanu) Department of Neurology, Unimed Clinic, Slatina, Romania

Publisher

Balkan medical union

Emtree Heading

adult; *arterial wall thickness; article; carotid artery; clinical article; disease association; female; fibrinogen blood level; follow up; human; human tissue; male; *mild cognitive impairment; Mini Mental State Examination; protein blood level; *C reactive protein/ec [Endogenous Compound]; *fibrinogen/ec [Endogenous Compound].

Drug Index Terms

*C reactive protein / *endogenous compound; *fibrinogen / *endogenous compound.

Other Index Terms

adult; *arterial wall thickness; article; carotid artery; clinical article; disease association; female; fibrinogen blood level; follow up; human; human tissue; male; *mild cognitive impairment; mini mental state examination; protein blood level.

Link to the Ovid Full Text or citation:

Click here for full text options

Link to the External Link Resolver:

SFX

For peer review only

For peer review only - http://bmjopen.bmj.com/site/about/guidelines.xhtml

193.

Carotid artery atherosclerosis is correlated with cognitive impairment in an elderly urban Chinese non-stroke population.

Xiang J., Zhang T., Yang Q.-W., Liu J., Chen Y., Cui M., Yin Z.-G., Li L., Wang Y.-J., Li J., Zhou H.-D.

Journal of Clinical Neuroscience. 20 (11) (pp 1571-1575), 2013. Date of Publication: November 2013.

AN: 52743846

Carotid artery atherosclerosis may cause increased intima-media thickness (IMT), plaque formation, and vessel stenosis or occlusion. However, the association between carotid artery atherosclerosis and cognitive impairment remains uncertain. This study explored the effects of IMT and carotid artery stenosis on cognitive function in an elderly Chinese non-stroke population. A total of 2015 patients were recruited. The IMT of carotid arteries and the presence of plaques and stenosis in carotid arteries were assessed with B-mode ultrasound examination. Cognitive performance was evaluated with neuropsychological tests. The cross-sectional relationships between cognitive performance and carotid wall characteristics were analyzed. Carotid artery atherosclerosis (IMT > 1.0) and stenosis were found in 86% and 51% of patients, respectively. Cognitive impairment was found in 356 (17.7%) patients. After adjustment for possible confounders, IMT (odds ratio [OR] = 1.96; 95% confidence interval [CI] 1.23-3.16) and hyperdense plaque (OR = 4.72; 95% CI 2.56-11.2) were associated with poor cognitive performance. Patients with severe (>=70%) carotid artery stenosis had a lower Mini-Mental State Examination score compared with the mild to modest (40-70%) carotid artery stenosis group. Cognitive performance differed between patients with left and right carotid artery stenosis, but no differences were observed between patients with severe left and right carotid artery stenosis. This study indicates that carotid artery atherosclerosis is correlated with cognitive impairment in the elderly Chinese population. A larger sample size across multiple centers and a longitudinal study are required to further explore the impact of carotid artery atherosclerosis on cognition in the elderly population.

© 2013 Elsevier Ltd. All rights reserved.

PMID

23978769 [http://www.ncbi.nlm.nih.gov/pubmed/?term=23978769]

Institution

(Xiang, Zhang, Yang, Liu, Chen, Cui, Yin, Li, Wang, Li, Zhou) Department of Neurology, Daping Hospital, Third Military Medical University, Yangzi River Street, Yuzhong District, Chongqing 400042, China

Publisher

Churchill Livingstone (1-3 Baxter's Place, Leith Walk, Edinburgh EH1 3AF, United Kingdom)

Emtree Heading

For peer review only

aged; arterial wall thickness; article; B scan; carotid artery obstruction; *carotid atherosclerosis; China; cognition; *cognitive defect/di [Diagnosis]; correlational study; cross-sectional study; disease severity; female; *geriatric patient; human; major clinical study; male; Mini Mental State Examination; neuropsychological test; population research; priority journal.

Other Index Terms

aged; arterial wall thickness; article; B scan; carotid artery obstruction; *carotid atherosclerosis; China; cognition; *cognitive defect / *diagnosis; correlational study; cross-sectional study; disease severity; female; *geriatric patient; human; major clinical study; male; Mini Mental State Examination; neuropsychological test; population research; priority journal.

Link to the Ovid Full Text or citation:

Click here for full text options

Link to the External Link Resolver:

SFX

195.

Relationship between common carotid artery intima media thickness and post-stroke cognitive impairment.

El-Shazli S., Selim K., Goda T.

Egyptian Journal of Neurology, Psychiatry and Neurosurgery. 50 (4) (pp 431-435), 2013. Date of Publication: October 2013.

AN: 372108358

Background: Common carotid artery intima-media thickness (CCA-IMT) has been associated with different cerebrovascular diseases, but its association with vascular cognitive impairment has not been clarified.

Objective(s): The purpose of this study was to investigate whether CCA-IMT is associated with cognitive impairment 6 months after an acute ischemic stroke.

Method(s): A total of 50 patients (32 males, 18 females) with a mean age 46.48+/-12.46, prospectively collected from neurology critical care unit in Zagazig university hospitals, with the first ever ischemi c stroke, underwent brain imaging and carotid ultrasonography during

For peer review only - http://bmjopen.bmj.com/site/about/guidelines.xhtml

hospitalization. Patients' cognitive performance was assessed using the Mini-Mental State Examination (MMSE) during hospitalization and six months later.
Result(s): CCA-IMT was significantly associated with cognitive impairment. Older age, hypertension, were also independently associated with post-stroke cognitive impairment.
Conclusion(s): CCA-IMT was independently associated with cognitive impairment 6 months after an acute ischemic stroke. It might help in screening of stroke patients at risk of cognitive impairment.
Institution
(El-Shazli, Selim, Goda) Department of Neurology, Zagazig University, Egypt
Publisher
Egyptian Society of Neurology, Psychiatry, and Neurosurgery (Egyptian Medical, Kasralainy, Cairo 11562, Egypt)
Emtree Heading
adult; age; aged; *arterial wall thickness; article; brain ischemia; clinical article; cognition; *cognitive defect/co [Complication]; *cognitive defect/di [Diagnosis]; *common carotid artery; controlled study; echography; Egypt; female; hospitalization; human; hypertension; intensive care unit; male; Mini Mental State Examination; neuroimaging; prospective study; screening; stroke patient; university hospital.
Other Index Terms
adult; age; aged; *arterial wall thickness; article; brain ischemia; clinical article; cognition; *cognitive defect / *complication / *diagnosis; *common carotid artery; controlled study; echography; Egypt; female; hospitalization; human; hypertension; intensive care unit; male; Mini Mental State Examination; neuroimaging; prospective study; screening; stroke patient; university hospital.

Link to the Ovid Full Text or citation:
Click here for full text options

Link to the External Link Resolver:
SFX

196.

For peer review only

Asymptomatic cervicocerebral atherosclerosis, intracranial vascular resistance and cognition: The AsIA-Neuropsychology Study.

Lopez-Oloriz J., Lopez-Cancio E., Arenillas J.F., Hernandez M., Jimenez M., Dorado L., Barrios M., Soriano-Raya J.J., Miralbell J., Caceres C., Fores R., Pera G., Davalos A., Mataro M.

Atherosclerosis. 230 (2) (pp 330-335), 2013. Date of Publication: October 2013.
AN: 369874323

Background and purpose: Carotid atherosclerosis has emerged as a relevant contributor to cognitive impairment and dementia whereas the role of intracranial stenosis and vascular resistance in cognition remains unknown. This study aims to assess the association of asymptomatic cervicocerebral atherosclerosis and intracranial vascular resistance with cognitive performance in a large dementia-free population.

Method(s): The Barcelona-AsIA (Asymptomatic Intracranial Atherosclerosis) Neuropsychology Study included 747 Caucasian subjects older than 50 with a moderate-high vascular risk (assessed by REGICOR score) and without history of neither symptomatic vascular disease nor dementia. Extracranial and transcranial color-coded duplex ultrasound examination was performed to assess carotid intima-media thickness (IMT), presence of carotid plaques (ECAD group), intracranial stenosis (ICAD group), and middle cerebral artery pulsatility index (MCA-PI) as a measure of intracranial vascular resistance. Neuropsychological assessment included tests in three cognitive domains: visuospatial skills and speed, verbal memory and verbal fluency.

Result(s): In univariate analyses, carotid IMT, ECAD and MCA-PI were associated with lower performance in almost all cognitive domains, and ICAD was associated with poor performance in some visuospatial and verbal cognitive tests. After adjustment for age, sex, vascular risk score, years of education and depressive symptoms, ECAD remained associated with poor performance in the three cognitive domains and elevated MCA-PI with worse performance in visuospatial skills and speed.

Conclusion(s): Carotid plaques and increased intracranial vascular resistance are independently associated with low cognitive functioning in Caucasian stroke and dementia-free subjects. We failed to find an independent association of intracranial large vessel stenosis with cognitive performance. © 2013 Elsevier Ireland Ltd.

PMID
24075765 [http://www.ncbi.nlm.nih.gov/pubmed/?term=24075765]

Institution
(Lopez-Oloriz, Soriano-Raya, Miralbell, Mataro) Department of Psychiatry and Clinical Psychobiology, Universitat de Barcelona, 08035 Barcelona, Spain (Lopez-Oloriz, Miralbell, Mataro) Institute for Brain, Cognition and Behavior (IR3C), Universitat de Barcelona, 08035 Barcelona, Spain

For peer review only - http://bmjopen.bmj.com/site/about/guidelines.xhtml

(Lopez-Cancio, Hernandez, Jimenez, Dorado, Caceres, Davalos) Department of Neurosciences, Hospital Universitari Germans Trias i Pujol, Universitat Autonoma de Barcelona, 08916 Badalona, Barcelona, Spain

(Arenillas) Stroke Unit, Department of Neurology, Hospital Clinico Universitario, 47003 Valladolid, Spain

(Barrios) Dept. of Methodology of Behavioural Sciences, Universitat de Barcelona, 08035 Barcelona, Spain

(Fores, Pera) Primary Healthcare Research Support Unit Metropolitana Nord, IDIAP Jordi Gol, Santa Coloma de Gramenet, Barcelona, Spain

Publisher

Elsevier Ireland Ltd (P.O. Box 85, Limerick, Ireland)

Emtree Heading

adult; aged; article; *brain atherosclerosis; carotid artery; Caucasian; cerebrovascular accident; *cognition; dementia; depression; female; human; intima; major clinical study; male; middle cerebral artery; *neuropsychology; priority journal; risk; scoring system; skill; stenosis; ultrasound; *vascular resistance; velocity; verbal memory; *asymptomatic cervicocerebral atherosclerosis; *intracranial vascular resistance.

Candidate Terms

*asymptomatic cervicocerebral atherosclerosis [other term]; *intracranial vascular resistance [other term].

Other Index Terms

adult; aged; article; *brain atherosclerosis; carotid artery; Caucasian; cerebrovascular accident; *cognition; dementia; depression; female; human; intima; major clinical study; male; middle cerebral artery; *neuropsychology; priority journal; risk; scoring system; skill; stenosis; ultrasound; *vascular resistance; velocity; verbal memory.

Link to the Ovid Full Text or citation:

Click here for full text options

Link to the External Link Resolver:

SFX

197.

For peer review only

Atherosclerosis and physical functioning in older men, a longitudinal study.

Den Ouden M.E.M., Schuurmans M.J., Arts E.M.A., Grobbee D.E., Bots M.L., Van Den Beld A.W., Lamberts S.W.J., Van Der Schouw Y.T.

Journal of Nutrition, Health and Aging. 17 (1) (pp 97-104), 2013. Date of Publication: January 2013.

AN: 52341644

Objective: Functional decline is a major threat to independency, progressing into functional limitations and eventually leading to disability. Chronic diseases, especially cardiovascular diseases, are important determinants of functional limitations and disability. Vascular damage exits long before it is clinically manifest and can have adverse effects on health, physical and cognitive functioning. The objective was to investigate the association between non-invasive atherosclerosis measures and physical functioning in older men.

Design(s): Prospective cohort study.

Setting(s): The study was conducted in the general community.

Participant(s): 195 independently living older men. Measurements: Atherosclerosis was measured by intima media thickness (CIMT) of the common carotid artery using ultrasonography and assessment for presence of atherosclerotic plaques. Physical functioning was measured by isometric handgrip strength and leg extensor strength using a hand held dynamometer, lower extremity function using the physical performance score and ability to perform activities of daily life using the modified Stanford Health Assessment Questionnaire. Linear regression analysis was performed to estimate the associations between CIMT or plaques and physical functioning.

Result(s): After adjustment for confounders, higher baseline CIMT was associated with lower isometric handgrip strength at follow up (betaCIMT =-7.21, 95% CI[-13.64;-0.77]). No other associations were found between CIMT and physical functioning. In addition, no associations were found for the presence of plaques and physical functioning either at baseline, or at follow-up.

Conclusion(s): Atherosclerosis, as measured by higher CIMT, is related to a lower isometric handgrip strength at follow-up, but no further associations with physical functioning were found in this longitudinal study among independently living older men. © 2013 Serdi and Springer-Verlag France.

PMID

23299387 [http://www.ncbi.nlm.nih.gov/pubmed/?term=23299387]

Institution

(Den Ouden, Arts, Grobbee, Bots, Van Der Schouw) Julius Center for Health Sciences and Primary Care, STR 6.131, University Medical Center Utrecht, PO Box 85500, 3508 GA Utrecht, Netherlands

(Schuurmans, Arts) Care for the Chronically Ill and Elderly, University of Applied Sciences Utrecht, Utrecht, Netherlands

For peer review only

(Schuurmans) Department of Rehabilitation, Nursing Science and Sports, UMC Utrecht, Utrecht, Netherlands
(Lamberts) Department of Internal Medicine, Erasmus University Medical Center, Rotterdam, Netherlands
(Van Den Beld) Department of Internal Medicine, Groene Hart Ziekenhuis, Gouda, Netherlands
Publisher
Springer Paris (1 rue Paul Cezanne, Paris 75008, France)
Emtree Heading
aged; arterial wall thickness; article; *atherosclerosis; atherosclerotic plaque; clinical assessment; cohort analysis; common carotid artery; daily life activity; dynamometer; follow up; grip strength; human; leg; longitudinal study; major clinical study; male; priority journal; prospective study; questionnaire; Stanford Health Assessment Questionnaire.
Candidate Terms
Stanford Health Assessment Questionnaire [other term].
Other Index Terms
aged; arterial wall thickness; article; *atherosclerosis; atherosclerotic plaque; clinical assessment; cohort analysis; common carotid artery; daily life activity; dynamometer; follow up; grip strength; human; leg; longitudinal study; major clinical study; male; priority journal; prospective study; questionnaire.

Link to the Ovid Full Text or citation:
Click here for full text options

Link to the External Link Resolver:
SFX

207.

Atherosclerotic calcification is related to cognitive decline.
Vernooij M., Bos D., Hofman A., Witteman J., Van Der Lugt A., Ikram M.
Alzheimer's and Dementia. Conference: Alzheimer's Association International Conference 2013. Boston, MA United States. Conference Publication: (var.pagings). 9 (4 SUPPL. 1) (pp P545), 2013.
Date of Publication: July 2013.

For peer review only - http://bmjopen.bmj.com/site/about/guidelines.xhtml

AN: 71417100

Background: Increasing evidence implicates atherosclerosis in the etiology of cognitive impairment and ultimately dementia, but longitudinal data are scarce. Also, it remains unclear whether atherosclerosis in different vessel beds affects cognitive decline differentially. In this study, we investigate the relationship between arterial calcification, as marker of atherosclerosis, in four vessel beds and cognitive decline over a 6 year interval.

Method(s): From the population-based Rotterdam Study, 1858 participants (mean age 68.4+/-5.9 years) underwent computed tomography of the coronaries, aortic arch, extracranial and intracranial carotid arteries to quantify atherosclerotic calcification. At baseline and after six years of follow-up, cognition was assessed using the MMSE and a neuropsychological test battery that comprised the following domains: global cognition, memory, executive function and information processing speed. Per participant, we calculated the difference between both time points in standardized scores for each cognitive domain. Relationships between atherosclerotic calcification and cognitive decline were assessed using linear regression models and adjusted for age, sex, education and additionally for APOE-epsilon4-status and cardiovascular risk factors.

Result(s): Larger load of calcification in the coronary arteries, aortic arch and intracranial carotid arteries, but not in the extracranial carotid arteries, was associated with a decline in MMSE scores and decline in global cognitive function (see Figure). These associations seemed primarily driven by decline in executive function.

Conclusion(s): Atherosclerosis is an important cause of cognitive decline and thus of interest as potential modifiable target for prevention of dementia. (Figure Presented).

Institution

(Vernooij, Bos, Hofman, Witteman, Van Der Lugt, Ikram) Erasmus Medical Center, Rotterdam, Netherlands

Publisher

Elsevier Inc.

Emtree Heading

*calcification; atherosclerosis; cognition; carotid artery; aorta arch; human; executive function; dementia; memory; neuropsychological test; follow up; population; cardiovascular risk; prevention; model; artery calcification; cognitive defect; linear regression analysis; computer assisted tomography; velocity; information processing; sexual education; coronary artery; Mini Mental State Examination; etiology; marker.

Drug Index Terms

marker.

Other Index Terms

*calcification; atherosclerosis; cognition; carotid artery; aorta arch; human; executive function; dementia; memory; neuropsychological test; follow up; population; cardiovascular risk; prevention;

For peer review only - http://bmjopen.bmj.com/site/about/guidelines.xhtml

model; artery calcification; cognitive defect; linear regression analysis; computer assisted tomography; velocity; information processing; sexual education; coronary artery; Mini Mental State Examination; etiology.

Link to the Ovid Full Text or citation:
Click here for full text options

Link to the External Link Resolver:
SFX

208.

Assessment of cerebral vasoreactivity using ultrasound techniques in Alzheimer's disease.
Giubilei F., Cipollini V., Sette G., De Carolis A., Capone F.T., Bianchi V., Monti M.S.
Alzheimer's and Dementia. Conference: Alzheimer's Association International Conference 2013. Boston, MA United States. Conference Publication: (var.pagings). 9 (4 SUPPL. 1) (pp P442), 2013.
Date of Publication: July 2013.
AN: 71416836
Background: Alzheimer's Disease (AD) is the most important cause of neurodegenerative dementia and its main pathological hallmarks include neuritic plaques and neurofibrillary tangles, involved in the beta amyloid cascade. Nevertheless, changes in cerebral hemodynamic might play a role in the cognitive decline. The aims of this study were to assess in AD subjects the cerebral vasomotor reactivity and to evaluate the possible correlation between this reactivity and the cognitive deficit.
Method(s): Thirty-six subjects (mean age +/- SD, 68,58 +/- 6,16 years) were consecutively enrolled. We recruited twenty-five subjects affected by AD, matched for age and education to eleven healthy control. Subjects with a Mini Mental State Evaluation (MMSE) score less than 15, cerebrovascular disease history, severe leucoencephalopathy and carotid stenoses major than 40% were excluded. All the subjects underwent MRI imaging, Neuropsychological evaluation and Carotid Duplex Ultrasonography. Cerebral vasomotor reactivity was assessed using the transcranial Doppler-based breath-holding index test (BHI).
Result(s): Both Cerebral blood flow velocity at the steady-state (CBFV) and BHI values were significantly lower in AD subject than in healthy control (46,34 +/- 7,61 cm/s vs 55,1 +/- 8,09 cm/s, p=0,007; 0,99 +/- 0,26 vs 1,21 +/- 0,24, p=0,031). Furthermore, we found a correlation between

For peer review only - http://bmjopen.bmj.com/site/about/guidelines.xhtml

CBFV value and MMSE score (p=0,003, r=0,654; Spearman's correlation), but no correlation between BHI value and MMSE score. No significant relationship was found between White Matter Lesions on MRI and both CBFVand BHI values. However, the AD subjects with carotid stenoses had a lower CBFVand BHI values than those without carotid stenoses (43,12 +/- 7,56 cm/s vs 48,68 +/- 7,07 cm/s, p=0,008; 0,9 +/- 0,23 vs 1,06 +/- 0,27, p=0,04; respectively). Considering the correlation between each neuropsychological test and the cerebrovascular reactivity indices, we only found a significant correlation with the Digit Span test score (CBFV: r= 0,542, p=0,046; BHI: r= 0,525, p =0,05; Spearman's correlation).

Conclusion(s): Our study suggests that AD subjects have changes in both dynamics and structure of their cerebral blood flow circulation. In particular, the CBFV reduction is correlated with the severity of the cognitive decline, suggesting that it can influence the cognitive decline in AD subjects.

Institution

(Giubilei) Faculty of Medicine, Psychology, Sapienza University of Rome, Rome, Italy (Cipollini, Sette, De Carolis, Capone, Bianchi, Monti) Sapienza University of Rome, Rome, Italy

Publisher

Elsevier Inc.

Emtree Heading

*ultrasound; *Alzheimer disease; carotid artery; brain blood flow; neurofibrillary tangle; senile plaque; dementia; human; cognitive defect; education; cerebrovascular disease; leukoencephalopathy; Mini Mental State Examination; mental health; imaging; transcranial doppler; breath holding; neuropsychological test; blood flow velocity; steady state; white matter lesion; dynamics; nuclear magnetic resonance imaging; amyloid.

Drug Index Terms

amyloid.

Other Index Terms

*ultrasound; *Alzheimer disease; carotid artery; brain blood flow; neurofibrillary tangle; senile plaque; dementia; human; cognitive defect; education; cerebrovascular disease; leukoencephalopathy; Mini Mental State Examination; mental health; imaging; transcranial doppler; breath holding; neuropsychological test; blood flow velocity; steady state; white matter lesion; dynamics; nuclear magnetic resonance imaging.

Link to the Ovid Full Text or citation:

Click here for full text options

Link to the External Link Resolver:

For peer review only - http://bmjopen.bmj.com/site/about/guidelines.xhtml

SFX

209.

Atherosclerotic calcification is related to cognitive decline.

Vernooij M., Bos D., Hofman A., Witteman J., Van Der Lugt A., Ikram M.

Alzheimer's and Dementia. Conference: Alzheimer's Association International Conference 2013. Boston, MA United States. Conference Publication: (var.pagings). 9 (4 SUPPL. 1) (pp P106), 2013.

Date of Publication: July 2013.

AN: 71416003

Background: Increasing evidence implicates atherosclerosis in the etiology of cognitive impairment and ultimately dementia, but longitudinal data are scarce. Also, it remains unclear whether atherosclerosis in different vessel beds affects cognitive decline differentially. In this study, we investigate the relationship between arterial calcification, as marker of atherosclerosis, in four vessel beds and cognitive decline over a 6 year interval.

Method(s): From the population-based Rotterdam Study, 1858 participants (mean age 68.4+/-5.9 years) underwent computed tomography of the coronaries, aortic arch, extracranial and intracranial carotid arteries to quantify atherosclerotic calcification. At baseline and after six years of follow-up, cognition was assessed using the MMSE and a neuropsychological test battery that comprised the following domains: global cognition, memory, executive function and information processing speed. Per participant, we calculated the difference between both time points in standardized scores for each cognitive domain. Relationships between atherosclerotic calcification and cognitive decline were assessed using linear regression models and adjusted for age, sex, education and additionally for APOE-e4-status and cardiovascular risk factors.

Result(s): Larger load of calcification in the coronary arteries, aortic arch and intracranial carotid arteries, but not in the extracranial carotid arteries, was associated with a decline in MMSE scores and decline in global cognitive function (see Figure). These associations seemed primarily driven by decline in executive function.

Conclusion(s): Atherosclerosis is an important cause of cognitive decline and thus of interest as potential modifiable target for prevention of dementia.

Institution

(Vernooij, Bos, Van Der Lugt, Ikram) Erasmus MC, Rotterdam, Netherlands (Hofman, Witteman) Erasmus Medical Center, Rotterdam, Netherlands

Publisher

For peer review only - http://bmjopen.bmj.com/site/about/guidelines.xhtml

Elsevier Inc.
Emtree Heading
*calcification; atherosclerosis; cognition; carotid artery; aorta arch; human; executive function; dementia; memory; neuropsychological test; follow up; population; cardiovascular risk; prevention; model; artery calcification; cognitive defect; linear regression analysis; computer assisted tomography; velocity; information processing; sexual education; coronary artery; Mini Mental State Examination; etiology; marker.
Drug Index Terms
marker.
Other Index Terms
*calcification; atherosclerosis; cognition; carotid artery; aorta arch; human; executive function; dementia; memory; neuropsychological test; follow up; population; cardiovascular risk; prevention; model; artery calcification; cognitive defect; linear regression analysis; computer assisted tomography; velocity; information processing; sexual education; coronary artery; Mini Mental State Examination; etiology.

Link to the Ovid Full Text or citation:
Click here for full text options

Link to the External Link Resolver:
SFX

211.

Carotid atherosclerosis and dementia risk in older adults. The three-city study.
Carcaillon L., Plichart M., Zureik M., Ritchie K., Rouaud O., Tzourio C., Dartigues J.-F., Empana J.-P.
Circulation. Conference: American Heart Association 2013 Scientific Sessions and Resuscitation Science Symposium. Dallas, TX United States. Conference Publication: (var.pagings). 128 (22 SUPPL. 1) (no pagination), 2013. Date of Publication: 26 Nov 2013.
AN: 71337927
Background: While carotid atherosclerosis has been associated with cognitive decline or prevalent dementia, there is limited evidence relating carotid atherosclerosis with incident dementia on a prospective basis.

For peer review only

Objective(s): To study the respective association of baseline carotid plaques and carotid intima media thickness (IMT) with incident all-cause dementia and dementia subtypes. Furthermore, the added value of carotid atherosclerosis for individual dementia risk prediction is quantified.
Method(s): Within the population-based Three-City study, 6,025 dementia-free men and women aged 65-85 underwent a standardized high-resolution -B-mode carotid ultrasound examination at baseline allowing to detect carotid plaques in the common carotid arteries (CCAs), the bifurcations and the origin of the internal carotid arteries and to quantify IMT per se in the CCAs. Incident all causes and dementia subtypes (Alzheimer disease (AD) and vascular/mixed dementia (VaD)) were validated by an independent expert committee. Hazards Ratio (HR) of the number of sites with carotid plaques (0, 1 and 2) and of 1 SD increase in CCA-IMT were estimated using Cox regression models. The added value of carotid atherosclerosis for dementia risk prediction was measured by the Harell's C index and the continuous net reclassification improvement index (NRI).
Result(s): After a mean follow-up of 5.4 years, 421 subjects had incident dementia, including 272 AD and 83 VaD. In multivariate analyses, carotid plaques were independently related to VaD only (HR for2 sites with plaques=1.93, 95% confidence interval (CI) 1.13-3.28; p for trend=0.013). CCA-IMT was not associated with dementia of any type (standardized HR for VaD=1.07, 95% CI: 0.85-1.33). Further adjustment for intercurrent stroke as time dependent variables or controlling for competing risk by death marginally modified the results. Adding carotid plaques to established dementia risk factors improved the Harell's c index from 0.716 to 0.739 (p=0.07) and yielded a significant continuous NRI of 43% (95% CI= 20.2-66.2; p < 0.001) regarding VaD risk.
Conclusion(s): In elderly men and women, carotid plaques on 2 sites or more are independent predictors of incident VaD and may improve the individual risk prediction of VaD.
Institution
(Carcaillon) Cardiovascular Epidemiology Section, Cntr for Rsch in Epidemiology and Population Health, Inserm UMR-S1018, Villejuif, France (Plichart) Paris Cardiovascular Rsch Cntr, Inserm UMR-S970, AP-HP Hopital Broca., Paris, France
(Zureik) Physiopathologie et epidemiologie de l'insuffisance respiratoire, Inserm U700, Paris, France
(Ritchie) Epidemiology, Univ of Montpellier, Imperial College, London, United Kingdom
(Rouaud) Cntr memoire de Ressource et de Recherche, CMRR CHU Dijon, Dijon, France
(Tzourio) Neuroepidemiology, Inserm U708 and Univ, Victor Segalen Bordeaux2, Bordeaux, France
(Dartigues) Epidemiology of Brain Aging, Inserm U897, Univ Victor Segalen Bordeaux2, Bordeaux, France
(Empana) Cardiovascular Epidemiology Section, Paris Cardiovascular Rsch Cntr, Inserm UMR-S970, Paris, France
Publisher
Lippincott Williams and Wilkins
Emtree Heading

*carotid artery; *cerebrovascular disease; *epidemiology; *aging; *carotid atherosclerosis; *dementia; *risk; *adult; *city; *medical society; *resuscitation; human; prediction; female; male; Alzheimer disease; internal carotid artery; common carotid artery; examination; ultrasound; multivariate analysis; confidence interval; dependent variable; follow up; proportional hazards model; population; model; hazard; cerebrovascular accident; arterial wall thickness; death; risk factor; aged.

Other Index Terms

*carotid artery; *cerebrovascular disease; *epidemiology; *aging; *carotid atherosclerosis; *dementia; *risk; *adult; *city; *medical society; *resuscitation; human; prediction; female; male; Alzheimer disease; internal carotid artery; common carotid artery; examination; ultrasound; multivariate analysis; confidence interval; dependent variable; follow up; proportional hazards model; population; model; hazard; cerebrovascular accident; arterial wall thickness; death; risk factor; aged.

Link to the Ovid Full Text or citation:

Click here for full text options

Link to the External Link Resolver:

SFX

217.

Carotid intima-media thickness is associated with cognitive deficiency in hypertensive patients with elevated central systolic blood pressure.

Dias E.D.M., Giollo L.T., Martinelli D.D., Mazeti C., Junior H.M., Vilela-Martin J.F., Yugar-Toledo J.C.

Cardiovascular Ultrasound. 10 (1) (no pagination), 2012. Article Number: 41. Date of Publication: 2012.

AN: 52268008

Background: The role of hypertension in the loss of cognitive function is controversial. Relationships between hypertension and increases in cerebral vascular resistance, diffused lesions and multiple lacunar infarcts of the white matter are well known. Thus, the objectives of this study were: to evaluate the relationship between hypertension and cognitive dysfunction (CD), identify risk factors and determine the association between early markers of vascular disease and CD in

For peer review only - http://bmjopen.bmj.com/site/about/guidelines.xhtml

hypertensive individuals. Methods. Two hundred individuals aged between 40 and 80years old were evaluated in this cross-sectional prospective study. Fifty participants were controls (CT). The remaining 150 hypertensive patients were subdivided into two groups, those with CD (HCD) and those without CD (HNCD). All participants underwent clinical evaluations and biochemical blood tests were performed. CD was investigated using the Mini Mental State Examination (MMSE) following the guidelines for its use in Brazil. The impact of hypertension on the arterial bed was assessed by identifying and measuring changes in the intima-media thickness (IMT) by vascular ultrasonography of the carotid arteries and analyses of the central blood pressure and Augmentation Index by applanation tonometry of the radial artery.

Result(s): There were no significant differences in the total cholesterol, high-density lipoprotein cholesterol and triglycerides plasma concentrations between the three groups. The serum creatinine and estimated glomerular filtration rate were within normal ranges for all three groups. A significantly lower MMSE score was recorded for the HCD Group compared to the HNCD and CT Groups (p-value<0.05).The IMT was significantly different between the HNCD and HCD Groups (p-value=0.0124). A significant difference in the IMT was also observed between hypertensive patients and the CT Group (p-value<0.0001). Age, low-density cholesterol, high-density cholesterol, triglycerides and IMT increased the Odds Ratio for cognitive dysfunction.The central systolic pressure was significantly higher in the HCD and HNCD Groups compared to CT Group (p-value<0.0001).

Conclusion(s): Hypertensive patients with CD have changes in the vascular morphology characterized by an increased carotid IMT, enhanced atherosclerotic lipid profile and impaired hemodynamic functional manifested by elevated central systolic blood pressure. © 2012 Dias et al.; licensee BioMed Central Ltd.

PMID

23078629 [http://www.ncbi.nlm.nih.gov/pubmed/?term=23078629]

Institution

(Dias, Giollo, Martinelli, Mazeti, Vilela-Martin, Yugar-Toledo) Hypertension Clinic, Department of Internal Medicine, State Medical School of Sao Jose Do Rio Preto (FAMERP), Rua: Las Vegas 200, Sao Jose do Rio Preto, SP CEP 15093-010, Brazil (Junior) Cardiovascular Pharmacology Laboratory, Faculty of Medical Sciences, State University of Campinas (UNICAMP), Campinas, SP CEP 13414-093, Brazil

Publisher

BioMed Central Ltd. (Floor 6, 236 Gray's Inn Road, London WC1X 8HB, United Kingdom)

Emtree Heading

adult; age; aged; *arterial wall thickness; article; blood analysis; blood chemistry; cardiovascular risk; *carotid artery; cholesterol blood level; clinical evaluation; *cognitive defect; controlled clinical trial; controlled study; creatinine blood level; disease association; disease marker; echography;

For peer review only

female; human; *hypertension; major clinical study; male; Mini Mental State Examination; radial artery; scoring system; systolic blood pressure; tonometry; triacylglycerol blood level; cholesterol/ec [Endogenous Compound]; creatinine/ec [Endogenous Compound]; high density lipoprotein cholesterol/ec [Endogenous Compound]; triacylglycerol/ec [Endogenous Compound].
Drug Index Terms
cholesterol / endogenous compound; creatinine / endogenous compound; high density lipoprotein cholesterol / endogenous compound; triacylglycerol / endogenous compound.
Other Index Terms
adult; age; aged; *arterial wall thickness; article; blood analysis; blood chemistry; cardiovascular risk; *carotid artery; cholesterol blood level; clinical evaluation; *cognitive defect; controlled clinical trial; controlled study; creatinine blood level; disease association; disease marker; echography; female; human; *hypertension; major clinical study; male; mini mental state examination; radial artery; scoring system; systolic blood pressure; tonometry; triacylglycerol blood level.

Link to the Ovid Full Text or citation:
Click here for full text options

Link to the External Link Resolver:
SFX

218.

Carotid artery plaque progression and cognitive decline: The Tromso Study 1994-2008.
Arntzen K.A., Schirmer H., Johnsen S.H., Wilsgaard T., Mathiesen E.B.
European Journal of Neurology. 19 (10) (pp 1318-1324), 2012. Date of Publication: October 2012.
AN: 51986146
Background: Carotid atherosclerosis is a risk factor for stroke and cognitive decline, but knowledge on how progression of carotid atherosclerosis affects cognitive function in stroke-free individuals is scarce.
Method(s): In the population-based Tromso study, we calculated the change in ultrasound-assessed carotid plaque number and total plaque area from baseline (survey 4) to follow-up 7 years later (survey 5) in 4274 middle-aged stroke-free subjects. Cognitive function was assessed at follow-up by the verbal memory test, the digit-symbol coding test, and the tapping test and

For peer review only

repeated after an additional 6 years in a subgroup of 2042 subjects (survey 6). Associations between the average of survey 4 and survey 5 plaque scores and the progression of plaque scores and cognitive test scores were assessed in regression analyses adjusted for baseline age, sex, education, depression, and cardiovascular risk factors.
Result(s): Progression of total plaque area was associated with lower scores in the digit-symbol coding test (multivariable adjusted standardized beta, -0.03; 95% CI, -0.05 to -0.00; P = 0.04) and the tapping test (beta, -0.03; 95% CI, -0.06 to -0.00; P = 0.03). Similar results were seen for progression of plaque number. The average plaque scores were associated with lower scores in all cognitive tests (P-values <= 0.01). No association was found between plaque scores and cognitive decline.
Conclusion(s): The average plaque scores were associated with lower scores in all cognitive tests. Progression of plaque scores was associated with lower scores in the digit-symbol coding test and the tapping test, but not with the verbal memory test or with cognitive decline. © 2012 EFNS.
PMID
22537454 [http://www.ncbi.nlm.nih.gov/pubmed/?term=22537454]
Institution
(Arntzen, Wilsgaard) Department of Community Medicine, University of TromsO, TromsO, Norway
(Arntzen, Johnsen, Mathiesen) Department of Neurology and Neurophysiology, University Hospital of North Norway, Tromso;, Norway
(Schirmer, Johnsen, Mathiesen) Department of Clinical Medicine, University of Tromso, Tromso;, Norway
(Schirmer) Department of Cardiology, University Hospital of North Norway, Tromso, Norway
Publisher
Blackwell Publishing Ltd (9600 Garsington Road, Oxford OX4 2XG, United Kingdom)
Emtree Heading
adult; aged; article; atherosclerotic plaque; cardiovascular risk; *carotid atherosclerosis; clinical assessment; cognition; depression; disease association; echography; education; female; follow up; human; major clinical study; male; *mental deterioration; population research; priority journal; regression analysis; verbal memory.
Other Index Terms
adult; aged; article; atherosclerotic plaque; cardiovascular risk; *carotid atherosclerosis; clinical assessment; cognition; depression; disease association; echography; education; female; follow up; human; major clinical study; male; *mental deterioration; population research; priority journal; regression analysis; verbal memory.

Link to the Ovid Full Text or citation:

For peer review only - http://bmjopen.bmj.com/site/about/guidelines.xhtml

Click here for full text options

Link to the External Link Resolver:
SFX

223.

Relationship between chronic stress and carotid intima-media thickness (IMT) in elderly Alzheimer's disease caregivers.
Roepke S.K., Allison M., Von Knel R., Mausbach B.T., Chattillion E.A., Harmell A.L., Patterson T.L., Dimsdale J.E., Mills P.J., Ziegler M.G., Ancoli-Israel S., Grant I.
Stress. 15 (2) (pp 121-129), 2012. Date of Publication: March 2012.
AN: 364138799
The stress associated with providing care for a spouse diagnosed with Alzheimer's disease can have adverse effects on cardiovascular health. One potential explanation is that chronic caregiving stress may contribute to the development of atherosclerosis. The purpose of this study was to determine whether the duration that one has provided care is associated with the degree of atherosclerotic burden, as measured by carotid artery intima-media thickness (IMT). One hundred and ten Alzheimer caregivers [mean age 74 +/- 8 (SD) years, 69% female] underwent in-home assessment of carotid artery IMT via B-mode ultrasonography. Data regarding medical history, blood pressure, and multiple indicators of caregiving stress were also collected. Multiple regression indicated that duration of care was positively associated with IMT measured in the internal/bifurcation segments of the carotid artery (beta = 0.202, p = 0.044) independent of risk factors such as age, gender, body mass index, smoking history, sleep quality, hypertension status, and caregiving stressors. Duration of care was positively associated with IMT in the common carotid artery, but the relationship was not significant. These findings provide more evidence of the link between chronic caregiving stress and cardiovascular disease and indicate that enduring the experience of caregiving over a period of years might be associated with atherosclerotic burden.
© Informa Healthcare USA, Inc.
PMID
21790484 [http://www.ncbi.nlm.nih.gov/pubmed/?term=21790484]
Institution
(Roepke, Chattillion, Ancoli-Israel) Joint Doctoral Program in Clinical Psychology, San Diego State University, San Diego, CA, United States (Roepke, Von Knel, Mausbach, Chattillion, Harmell,

For peer review only - http://bmjopen.bmj.com/site/about/guidelines.xhtml

Patterson, Dimsdale, Mills, Ancoli-Israel, Grant) Department of Psychiatry, School of Medicine, University of California San Diego, 9500 Gilman Drive, San Diego, CA 92093-0680, United States
(Allison) Department of Family and Preventative Medicine, University of California San Diego, San Diego, CA, United States
(Von Knel) Department of General Internal Medicine, Inselspital, Bern University Hospital, Bern, Switzerland
(Ziegler) Department of Medicine, University of California San Diego, San Diego, CA, United States
Publisher
Informa Healthcare (69-77 Paul Street, London EC2A 4LQ, United Kingdom)
Emtree Heading
adult; aged; *Alzheimer disease; *arterial wall thickness; article; B scan; blood pressure; body mass; cardiovascular disease; *caregiver; carotid artery bifurcation; cholesterol blood level; *chronic stress; daily life activity; educational status; female; gender; health status; human; hypertension; income; major clinical study; male; medical history; priority journal; psychosocial care; risk factor; sleep quality; smoking; low density lipoprotein cholesterol/ec [Endogenous Compound].
Drug Index Terms
low density lipoprotein cholesterol / endogenous compound.
Other Index Terms
adult; aged; *Alzheimer disease; *arterial wall thickness; article; B scan; blood pressure; body mass; cardiovascular disease; *caregiver; carotid artery bifurcation; cholesterol blood level; *chronic stress; daily life activity; educational status; female; gender; health status; human; hypertension; income; major clinical study; male; medical history; priority journal; psychosocial care; risk factor; sleep quality; smoking.

Link to the Ovid Full Text or citation:
Click here for full text options

Link to the External Link Resolver:
SFX

225.

Carotid atherosclerosis and 10-year changes in cognitive function.

For peer review only

Zhong W., Cruickshanks K.J., Schubert C.R., Acher C.W., Carlsson C.M., Klein B.E.K., Klein R., Chappell R.J.

Atherosclerosis. 224 (2) (pp 506-510), 2012. Date of Publication: October 2012.

AN: 52137109

Background: Carotid atherosclerosis has been suggested to be involved in cognitive decline. Method(s): The Epidemiology of Hearing Loss Study is a longitudinal study of aging among Beaver Dam residents, WI. In 1998-2000, carotid intima-media thickness (IMT) and plaque were measured by ultrasound; cognitive function was measured by the Mini-Mental State Examination (MMSE). Follow-up examinations were conducted in 2003-2005 and 2009-2010. Incidence of cognitive impairment was defined as an MMSE score <24 or reported physician-diagnosed dementia during the follow-up. In the last examination, five additional cognitive tests were added. The associations of carotid atherosclerosis with incident cognitive impairment and cognitive test performance ten years later were evaluated.

Result(s): A total of 1651 participants (mean age 66.8 years, 41% men) without cognitive impairment at baseline were included in the incidence analysis. IMT was associated with incidence of cognitive impairment after multiple adjustments (hazard ratio: 1.09, p = 0.02 for each 0.1 mm increase in IMT). A total of 1311 participants with atherosclerosis data at baseline had the additional cognitive tests 10 years later. Larger IMT was associated with longer time to complete the Trail-Making Test-part B after multiple adjustments (0.1 mm IMT: 2.3 s longer, p = 0.02). Plaque was not associated with incident cognitive impairment or cognitive test performance 10 years later.

Conclusion(s): In this population-based longitudinal study, carotid IMT was associated with a higher risk of developing cognitive impairment during the 10-year follow-up, and was associated with poorer performance in a test of executive function 10 years later. © 2012.

PMID

22854188 [http://www.ncbi.nlm.nih.gov/pubmed/?term=22854188]

Institution

(Zhong, Cruickshanks, Schubert, Acher, Carlsson, Klein, Klein, Chappell) University of Wisconsin-Madison, 1036 WARF 610 Walnut Street, WI 53726, United States

Publisher

Elsevier Ireland Ltd (P.O. Box 85, Limerick, Ireland)

Emtree Heading

aged; aging; article; brain function; *carotid atherosclerosis/di [Diagnosis]; cognition; *cognitive defect; common carotid artery; dementia/di [Diagnosis]; female; follow up; human; internal carotid artery; intima; longitudinal study; major clinical study; male; Mini Mental State Examination; priority journal; thickness; tunica media; ultrasound; apolipoprotein A4/ec [Endogenous Compound]; intima media thickness.

Candidate Terms

For peer review only

For peer review only - http://bmjopen.bmj.com/site/about/guidelines.xhtml

intima media thickness [other term].

Drug Index Terms

apolipoprotein A4 / endogenous compound.

Other Index Terms

aged; aging; article; brain function; *carotid atherosclerosis / *diagnosis; cognition; *cognitive defect; common carotid artery; dementia / diagnosis; female; follow up; human; internal carotid artery; intima; longitudinal study; major clinical study; male; mini mental state examination; priority journal; thickness; tunica media; ultrasound.

Link to the Ovid Full Text or citation:

Click here for full text options

Link to the External Link Resolver:

SFX

226.

Vascular predictors of cognitive decline in patients with mild cognitive impairment.

Viticchi G., Falsetti L., Vernieri F., Altamura C., Bartolini M., Luzzi S., Provinciali L., Silvestrini M.

Neurobiology of Aging. 33 (6) (pp 1127e1-1127e9), 2012. Date of Publication: June 2012.

AN: 51792706

Our aim in this study was to assess the relationship between the state of cerebral vessels and the risk of conversion from mild cognitive impairment (MCI) to Alzheimer's disease (AD). We included 117 MCI patients. They underwent an ultrasonographic assessment of common carotid arteries intima-media thickness (IMT) and carotid plaque index. Cerebrovascular reactivity to hypercapnia in the middle cerebral arteries was calculated with the Breath-Holding Index (BHI). After a 12-month follow-up period, neuropsychological examinations demonstrated a progression to dementia in 21 patients. Pathological values of BHI and IMT significantly increased the risk of conversion (BHI: odds ratio, 5.80; 95% confidence interval, 1.83-18.37, $p < 0.05$; IMT: odds ratio, 3.08; 95% confidence interval, 1.02-9.33; $p < 0.05$, multinomial logistic regression analysis). Comparison between patients with all normal values and those with the simultaneous alteration of the 2 vascular indexes showed an increase in the risk of conversion from 9% to 33% (ordinal

For peer review only - http://bmjopen.bmj.com/site/about/guidelines.xhtml

regression analysis). Our findings show that alterations of cerebral vessel functional and anatomic status increase the risk of conversion from MCI to dementia. © 2012 IBRO.

PMID

22217417 [http://www.ncbi.nlm.nih.gov/pubmed/?term=22217417]

Institution

(Viticchi, Bartolini, Luzzi, Provinciali, Silvestrini) Department of Experimental and Clinical Medicine, Marche Polytechnic University, Ancona, Italy (Falsetti) Internal and Subintensive Medicine, Ospedali Riuniti, Ancona, Italy

(Vernieri, Altamura) Neurological Clinic, Campus Biomedico University, Rome, Italy

Publisher

Elsevier Inc. (360 Park Avenue South, New York NY 10010, United States)

Emtree Heading

aged; *Alzheimer disease/di [Diagnosis]; arterial wall thickness; article; brain blood flow; brain blood vessel; carotid atherosclerosis; *cognitive defect; controlled study; echography; female; follow up; functional assessment; hemodynamics; human; logistic regression analysis; major clinical study; male; *mild cognitive impairment/di [Diagnosis]; morphology; *neuroimaging; neuropsychological test; priority journal.

Other Index Terms

aged; *Alzheimer disease / *diagnosis; arterial wall thickness; article; brain blood flow; brain blood vessel; carotid atherosclerosis; *cognitive defect; controlled study; echography; female; follow up; functional assessment; hemodynamics; human; logistic regression analysis; major clinical study; male; *mild cognitive impairment / *diagnosis; morphology; *neuroimaging; neuropsychological test; priority journal.

Link to the Ovid Full Text or citation:

Click here for full text options

Link to the External Link Resolver:

SFX

228.

For peer review only

For peer review only - http://bmjopen.bmj.com/site/about/guidelines.xhtml

Impaired cognitive function in patients with atherosclerotic carotid stenosis and correlation with ultrasound strain measurements.

Rocque B.G., Jackson D., Varghese T., Hermann B., McCormick M., Kliewer M., Mitchell C., Dempsey R.J.

Journal of the Neurological Sciences. 322 (1-2) (pp 20-24), 2012. Date of Publication: 15 Nov 2012.

AN: 52041434

Introduction: It has been postulated that up to 11 million silent strokes occur annually. While these patients are without classic neurologic deficits, they may exhibit cognitive decline. In this study, we examine the cognitive function of patients with carotid stenosis. Additionally, we evaluate a noninvasive measure of strain in pulsating carotid artery plaques to determine its ability to predict cognitive decline.

Method(s): We administered the Repeatable Battery for the Assessment of Neuropsychological Status (RBANS) to 44 patients with carotid stenosis. All patients had stenosis meeting NASCET or ACAS criteria for endarterectomy, and were classified as symptomatic or asymptomatic as defined by these publications. Age-adjusted scores for each of the 5 RBANS domains (immediate memory, visuospatial ability, language, attention, and delayed memory) were compared between symptomatic and asymptomatic patients. Mean score for each of the 5 domains was then compared to all other domains, regardless of symptom status. From this cohort, 23 patients underwent assessment of carotid plaque strain by tracking displacements in ultrasound radiofrequency data to estimate axial and principal strains over the cardiac cycle.

Result(s): Thirty symptomatic and 14 asymptomatic patients were studied. Visuospatial scores were significantly lower than any other domain regardless of symptoms ($p < 0.05$ for all pairwise comparisons). No other domain score was significantly different from any other. In the language domain, asymptomatic patients scored significantly higher than symptomatic patients ($p < 0.05$. For all other domains, no difference was found. Asymptomatic patients showed a relationship between plaque strain and immediate memory ($r = -.61$, p = ns). Left carotid disease was associated with poorer performance across multiple cognitive domains with increasing accumulated strain. This was not seen in right carotid disease.

Conclusion(s): Patients with large carotid plaques (> 70% stenosis) exhibit significant difficulties in mental status whether classically symptomatic or asymptomatic. While language deficits may be a non-specific marker for stroke symptoms, visuospatial deficits are seen before classic symptoms, suggesting that carotid disease may become symptomatic earlier and more subtly than previously suspected. Abnormal strain distribution with pulsation may be related to cognition. © 2012 Elsevier B.V.

PMID

22658531 [http://www.ncbi.nlm.nih.gov/pubmed/?term=22658531]

Institution

For peer review only - http://bmjopen.bmj.com/site/about/guidelines.xhtml

(Rocque, McCormick, Dempsey) UW-Madison School of Medicine and Public Health, Department of Neurological Surgery, 600 Highland Avenue, K4/866, Madison, WI 53792, United States
(Jackson, Hermann) UW-Madison School of Medicine and Public Health, Department of Neurology, United States
(Mitchell) University of Wisconsin Milwaukee, College of Health Sciences, Department of Health Sciences, 2400 Hartland Ave., Milwaukee, WI 53211, United States
(Varghese, McCormick) UW-Madison School of Medicine and Public Health, Department of Medical Physics, United States
(Kliewer) UW-Madison School of Medicine and Public Health, Department of Radiology, United States

Publisher
Elsevier (P.O. Box 211, Amsterdam 1000 AE, Netherlands)
Emtree Heading
aptitude test; article; attention; *carotid artery obstruction/su [Surgery]; *carotid atherosclerosis/su [Surgery]; carotid endarterectomy; clinical article; *cognitive defect; depth perception; *echography; human; language; neuroimaging; priority journal; radiofrequency; short term memory.
Other Index Terms
aptitude test; article; attention; *carotid artery obstruction / *surgery; *carotid atherosclerosis / *surgery; carotid endarterectomy; clinical article; *cognitive defect; depth perception; *echography; human; language; neuroimaging; priority journal; radiofrequency; short term memory.

Link to the Ovid Full Text or citation:
Click here for full text options

Link to the External Link Resolver:
SFX

230.

Effects of carotid endarterectomy on the dynamics of cognitive impairments in patients with atherosclerotic stenosis of the carotid arteries.

Yakhno N.N., Fedorova T.S., Damulin I.V., Shcherbyuk A.N., Vinogradov O.A., Lavrentiev A.V.

For peer review only - http://bmjopen.bmj.com/site/about/guidelines.xhtml

Neuroscience and Behavioral Physiology. 42 (6) (pp 543-549), 2012. Date of Publication: July 2012.
AN: 365619777

The clinical and neurochemical characteristics of non-dementia cognitive disorders were studied in 102 patients with atherosclerotic carotid sclerosis, with assessment of their dynamics after carotid endarterectomy (CEAE). Mild cognitive disorders were seen in 37 patients (36.3%) and moderate cognitive disorders in 36 patients (35.3%). Moderate cognitive impairments were significantly more common in patients with symptoms of carotid stenosis, dominated by structural changes in the brain on neuroimaging (leukoaraiosis and infarcts); unstable atherosclerotic plaques, with a predominance of the hypodense component, were also more frequent. This suggests that cognitive dysfunction in patients with atherosclerotic carotid stenosis results not only from decreased perfusion, but also from arterio-arterial microembolism. CEAE was found to have favorable effects on cognitive functions. Positive changes were marked in patients with asymptomatic carotid stenosis. However, CEAE could also have adverse influences on cognitive functions in patients with moderate cognitive disorders of dysmnestic type and symptoms of carotid stenosis. © 2012 Springer Science+Business Media, Inc.

Institution
(Yakhno, Fedorova, Damulin) 1 Department of Nervous Diseases, I. M. Sechenov First Moscow State Medical University, Russian Federation (Shcherbyuk, Vinogradov, Lavrentiev) 2 Department of Vascular Surgery, University Clinical Hospital No. 1, I. M. Sechenov First Moscow State Medical University, Russian Federation

Publisher
Springer New York (233 Spring Street, New York NY 10013-1578, United States)

Emtree Heading
adult; article; atherosclerotic plaque; brain infarction; brain perfusion; *carotid artery obstruction; *carotid atherosclerosis/su [Surgery]; *carotid endarterectomy; *cognitive defect; deterioration; disease severity; female; human; internal carotid artery occlusion; leukoaraiosis; major clinical study; male; memory disorder; microembolism; neuroimaging; neuropsychological test; nuclear magnetic resonance imaging.

Other Index Terms
adult; article; atherosclerotic plaque; brain infarction; brain perfusion; *carotid artery obstruction; *carotid atherosclerosis / *surgery; *carotid endarterectomy; *cognitive defect; deterioration; disease severity; female; human; internal carotid artery occlusion; leukoaraiosis; major clinical study; male; memory disorder; microembolism; neuroimaging; neuropsychological test; nuclear magnetic resonance imaging.

Link to the Ovid Full Text or citation:

For peer review only - http://bmjopen.bmj.com/site/about/guidelines.xhtml

Click here for full text options

Link to the External Link Resolver:
SFX

231.

A vascular approach to mild cognitive impairment in 12 adults. A pilot study.
Fromm A., Haasz J., Lundervold A.J., Moen G., Skulstad S., Thomassen L.
Cerebrovascular Diseases. Conference: 21st European Stroke Conference. Lisbon Portugal. Conference Publication: (var.pagings). 33 (SUPPL. 2) (pp 394-395), 2012. Date of Publication: May 2012.
AN: 71637254
Background: Mild cognitive impairment (MCI) is a subtle memory disorder, not matching criteria for dementia. There is evidence for vascular comorbidity, obviously in vascular dementia, but also in other types like Alzheimer's Disease. Increased carotid Intima-Media- Thickness (IMT) and spontaneous cerebral microemboli have been found more frequently in different types. We hypothesised that ultrasound examination and MRI would detect a high degree of vascular disease, brain injury and degenerative changes in patients with MCI.
Method(s): In cooperation with our memory clinic, 12 patients aged 61 to 77 (median 69 years) with amnestic MCI were referred to our department for neurovascular investigation. All patients underwent ultrasound examination with carotid duplex including IMT measurement, and Transcranial Doppler (TCD) including one-hour emboli monitoring, vasoreactivity measurement and Bubble test. Cerebral MRI for evaluation of vascular and white matter lesions, brain at rophy, hippocampal volumes and Amyloid angiopathy was performed in 11 patients.
Result(s): Vascular risk factors were found in 8 patients (67%). 5 patients had atherosclerotic lesions, of which 4 had mild (33%) and one had moderate (8%) carotid stenosis. Distal CCA IMT >1 mm was found in 2 patients (17%), no patient had IMT > 1.11 mm. None of the 10 patients with acceptable bone window (83%) had intracranial stenosis in TCD. Vasoreactivity was low (18 %) in one patient (8%). Permanent rightleft shunt was found in 4 patients (33%), of which one (8%) showed spontaneous cerebral microemboli. Hippocampal volume reduction was present in 2 patients (17%), and 4 patients (33%) had cortical atrophy. Chronic ischemic changes were found in 4 patients (33%), of which one (8%) also had a cortical infarction and microbleedings. Amyloid Angiopathy was not found.

For peer review only - http://bmjopen.bmj.com/site/about/guidelines.xhtml

Conclusion(s): We suggest that pure amnestic MCI is less associated with cerebrovascular disease, and may be more consistent with evolving Alzheimer's Disease.

Institution
(Fromm, Thomassen) Department of Clinical Medicine, University of Bergen, Bergen, Norway
(Haasz) Department of Biomedicine, University of Bergen, Bergen, Norway
(Lundervold) Department of Biological and Medical Psychology, University of Bergen, Bergen, Norway
(Moen) Department of Radiology, Haukeland University Hospital, Bergen, Norway
(Skulstad) NKS Olaviken, Hospital for Old Age Psychiatry, Erdal, Norway

Publisher
S. Karger AG

Emtree Heading
*mild cognitive impairment; *adult; *pilot study; *cerebrovascular accident; patient; human; examination; ultrasound; vascular amyloidosis; multiinfarct dementia; dementia; brain injury; memory disorder; vascular disease; carotid artery obstruction; stenosis; brain infarction; memory; embolism; comorbidity; transcranial doppler; arterial wall thickness; monitoring; white matter lesion; brain; carotid artery; hospital; risk factor; bone; atrophy; cerebrovascular disease; Alzheimer disease; nuclear magnetic resonance imaging.

Other Index Terms
*mild cognitive impairment; *adult; *pilot study; *cerebrovascular accident; patient; human; examination; ultrasound; vascular amyloidosis; multiinfarct dementia; dementia; brain injury; memory disorder; vascular disease; carotid artery obstruction; stenosis; brain infarction; memory; embolism; comorbidity; transcranial doppler; arterial wall thickness; monitoring; white matter lesion; brain; carotid artery; hospital; risk factor; bone; atrophy; cerebrovascular disease; Alzheimer disease; nuclear magnetic resonance imaging.

Link to the Ovid Full Text or citation:
Click here for full text options

Link to the External Link Resolver:
SFX

233.

For peer review only - http://bmjopen.bmj.com/site/about/guidelines.xhtml

Ultrasound characteristics of dementia in renal patients.

Tegos T.J., Dimas G., Pitsalidis C., Valavanis A., Chatziapostolou A., Papadimitriou A., Chrysogonidis I., Grekas D., Orologas A.

Cerebrovascular Diseases. Conference: 21st European Stroke Conference. Lisbon Portugal. Conference Publication: (var.pagings). 33 (SUPPL. 2) (pp 225-226), 2012. Date of Publication: May 2012.

AN: 71637053

Background: It has been suggested that vascular ultrasound findings are associated with dementia. The aim of this study was to determine the association of carotid, femoral, middle cerebral artery (MCA) ultrasound findings with dementia.

Method(s): Analysis involved imaging by duplex of carotid and femoral arteries of 61 patients(43 male, 18 female, mean age: 63.14 years) in a longitudinal fashion, to detect the presence of plaque and to assess the intima media thickness(IMT). Each artery was assigned a score(presence of plaque=1, absence of plaque=0, IMT>=0.8 mm=1, IMT<0.8 mm=0) and the total score of the four vessels(two carotid and two femoral) was calculated per patient(atherosclerotic ultrasonic score-ATHUS). Subsequently the mean pulsatility index(PI) of both MCAs and the minimental state examination(MMSE) of every patient was evaluated. Brain CT scans were performed to ensure the small vessel disease as the cause for the intellectual decline of patients.

Result(s): Group A(ATHUS=0-2, 26 patients) was associated with median MMSE of 29 and interquartile range of 2. The corresponding values for Group B (ATHUS=3-5, 16 patients) and Group C (ATHUS=6-8, 19 patients) were: 28(3.75) and 25(5) respectively (p<0.01). PI of 0.97 separated the patients in terms of MMSE into: Group D (PI<=0.97, median MMSE=29, interquartile range=2) and Group E (PI>0.97) with corresponding values of 28(4) (p<0.01).

Conclusion(s): Our results suggested that the degree of atherosclerosis was inversely related to MMSE. This position might be clarified in larger studies of intellectually declined patients, aiming to establish the role of atherosclerosis detected on ultrasound in dementia.

Institution

(Tegos, Dimas, Pitsalidis, Valavanis, Papadimitriou, Chrysogonidis, Grekas, Orologas) A Department of Neurology, Ahepa Hospital, Aristotelian University of Thessaloniki, Thessaloniki, Greece
(Chatziapostolou) First Medical Propaedeytic Department, Ahepa Hospital, Aristotelian University of Thessaloniki, Thessaloniki, Greece

Publisher

S. Karger AG

Emtree Heading

For peer review only

*dementia; *patient; *human; *cerebrovascular accident; *ultrasound; carotid artery; Mini Mental State Examination; atherosclerosis; middle cerebral artery; imaging; computer assisted tomography; brain; examination; artery; arterial wall thickness; femoral artery; male; female.

Other Index Terms

*dementia; *patient; *human; *cerebrovascular accident; *ultrasound; carotid artery; Mini Mental State Examination; atherosclerosis; middle cerebral artery; imaging; computer assisted tomography; brain; examination; artery; arterial wall thickness; femoral artery; male; female.

Link to the Ovid Full Text or citation:

Click here for full text options

Link to the External Link Resolver:

SFX

234.

Carotid atherosclerosis predicts incidence of dementia in the baltimore longitudinal study of aging.

Wendell C.R., Waldstein S.R., Zonderman A.B.

Psychosomatic Medicine. Conference: 70th Annual Scientific Meeting of the American Psychosomatic Society. Athens Greece. Conference Publication: (var.pagings). 74 (3) (pp A95), 2012. Date of Publication: 01 Apr 2012.

AN: 71123883

A rapidly emerging literature suggests that cardiovascular health may be as important to the pathogenesis of Alzheimer's disease and all-cause dementia as it is to the development of vascular dementia. Though several studies have identified higher prevalence of carotid atherosclerosis among dementia patients vs. controls, very few have focused on carotid atherosclerosis as a predictor of dementia in a cognitively normal baseline sample. Here we examined prospective relations of common carotid artery plaque and intimal medial thickness (IMT) to incident dementia among 293 participants aged 60 and older (mean baseline age=73 years, 60% male, 81% white) from the Baltimore Longitudinal Study of Aging. Carotid studies were performed with high resolution B-mode ultrasonography, and cognitive status was determined by annual neuropsychological assessment and consensus conference. Cox proportional hazards models were adjusted for baseline age, sex, race, education, blood pressure, cholesterol, cardiovascular disease,

For peer review only

and smoking. The dependent measure was age at onset of dementia or the last observed (censored) age of cognitively normal participants. After up to 14 years of follow-up (median=7), 52 participants developed dementia. Presence of carotid plaque predicted dementia significantly (hazard ratio [HR] = 1.42; 95% confidence interval [CI], 1.02-1.98). Thirty-three percent of participants with bilateral carotid plaque developed dementia during the study period, versus 17% and 13% with unilateral and no plaque, respectively. Maximal carotid IMT also predicted dementia significantly, but in men only (HR=1.42; 95% CI, 1.02-1.97). Unadjusted descriptive analyses showed escalating dementia incidence with increasing baseline carotid IMT quintile (i.e., 17%, 19%, 26%, 21%, 31%, respectively). Results suggest possible dose-response relations between atherosclerosis severity and prospective dementia risk. Our findings provide indirect evidence that early intervention to reduce atherosclerosis may limit or delay the onset of dementia with aging.

Institution

(Wendell) Radiology, Johns Hopkins University, Baltimore, MD, United States (Waldstein) Psychology, University of Maryland, Baltimore County, Baltimore, MD, United States

(Zonderman) Laboratory of Behavioral Neuroscience, National Institute on Aging, Baltimore, MD, United States

Publisher

Lippincott Williams and Wilkins

Emtree Heading

*carotid atherosclerosis; *dementia; *United States; *longitudinal study; *aging; *psychosomatics; *society; human; carotid artery; male; atherosclerosis; multiinfarct dementia; common carotid artery; patient; cardiovascular disease; onset age; follow up; hazard ratio; arterial wall thickness; dose response; blood pressure; prevalence; proportional hazards model; education; smoking; pathogenesis; health; consensus; confidence interval; early intervention; risk; echography; Alzheimer disease; cholesterol.

Drug Index Terms

cholesterol.

Other Index Terms

*carotid atherosclerosis; *dementia; *United States; *longitudinal study; *aging; *psychosomatics; *society; human; carotid artery; male; atherosclerosis; multiinfarct dementia; common carotid artery; patient; cardiovascular disease; onset age; follow up; hazard ratio; arterial wall thickness; dose response; blood pressure; prevalence; proportional hazards model; education; smoking; pathogenesis; health; consensus; confidence interval; early intervention; risk; echography; Alzheimer disease.

Link to the Ovid Full Text or citation:

For peer review only - http://bmjopen.bmj.com/site/about/guidelines.xhtml

Click here for full text options

Link to the External Link Resolver:
SFX

243.

Worsening of cognitive abilities after carotid endarterectomy and carotid artery stenting.
Stanisic M.G., Stepak H., Stefaniak K., Majewska N., Cicha E., Oszkinis G.
CardioVascular and Interventional Radiology. Conference: Cardiovascular and Interventional Radiological Society of Europe, CIRSE 2012. Lisbon Portugal. Conference Publication: (var.pagings). 35 (SUPPL. 1) (pp S244-S245), 2012. Date of Publication: September 2012.
AN: 70901683
Learning objectives: Atherosclerosis of carotid arteries, in many cases asymptomatic, not only increases the risk of severe central nervous system complication like transient ischaemic episodes (TIAs) and strokes but also decreases the quality of cognition. The aim of this study was to compare the effect on cognitive functions of carotid endarterectomy (CEA) and carotid artery stenting (CAS) for asymptomatic carotid artery stenosis.
Background(s): A prospective study was conducted among 32 asymptomatic patients with no neurological deficits, who were admitted for the treatment of carotid artery stenosis. 20 patients consented to participate in the study. 12 were classified for CEA and 8 for CAS. CAS and CEA were performed by vascular surgeons and endovascular specialist with high level of expertise. Before analyzing cognitive functions, quick test of intelligence (Wechsler Adult Intelligence Scale Test) was performed. 8 patients were excluded due to low intelligence quotient. Among the rest of the study group (12 patients: 6 women, 6 men) cognitive functions were evaluated by the Benton visual retention test (BVRT). The measurement was performed 12-24 hours before and 12-14 weeks after revascularization. Statistical analysis of numeric results of BVRT was performed with the use of chi2 test. Clinical Findings/Procedure: Among analyzed patients, 9 (4 women, 5 men) presented worse results in BVRT after revascularization ($p<0,05$). 1 patient presented better and 2 patients had the same re-sults after treatment. There was no statistically significant difference between those who underwent CAS and CEA.
Conclusion(s): CAS and CEA equally reduce cognitive functions. It can be explained by asymptomatic psycho-organic brain damage due to atherosclerosis itself and embolisation during interventions.

For peer review only - http://bmjopen.bmj.com/site/about/guidelines.xhtml

Institution

(Stanisic, Stepak, Stefaniak, Cicha, Oszkinis) Department of General and Vascular Surgery, Poznan University of Medical Sciences, Poznan, Poland (Majewska) Department of Clinical Radiology, Poznan University of Medical Sciences, Poznan, Poland

Publisher

Springer New York

Emtree Heading

*carotid endarterectomy; *carotid artery stenting; *society; *Europe; human; patient; cognition; atherosclerosis; revascularization; carotid artery obstruction; female; male; Wechsler intelligence scale; intelligence; medical specialist; brain damage; surgeon; chi square test; cerebrovascular accident; carotid artery; statistical analysis; prospective study; artificial embolism; central nervous system; intelligence quotient; risk; learning; carcinoembryonic antigen.

Drug Index Terms

carcinoembryonic antigen.

Other Index Terms

*carotid endarterectomy; *carotid artery stenting; *society; *Europe; human; patient; cognition; atherosclerosis; revascularization; carotid artery obstruction; female; male; Wechsler Intelligence Scale; intelligence; medical specialist; brain damage; surgeon; chi square test; cerebrovascular accident; carotid artery; statistical analysis; prospective study; artificial embolism; central nervous system; intelligence quotient; risk; learning.

Link to the Ovid Full Text or citation:

Click here for full text options

Link to the External Link Resolver:

SFX

246.

Elevated arterial stiffness and deepbrain white matter hyperintensity in patients with mild cognitive impairment.

Tseng B., Ayaz M., Brunk E., Armstrong K., Martin-Cook K., Diaz-Arrastia R., Weiner M., Cullum M., Levine B., Lu H., Zhang R.

For peer review only

Alzheimer's and Dementia. Conference: Alzheimer's Association International Conference 2012. Vancouver, BC Canada. Conference Publication: (var.pagings). 8 (4 SUPPL. 1) (pp P700-P701), 2012. Date of Publication: 2012.

AN: 70860991

Background: Brain white matter hyperintensity (WMH) is a measure of white matter damage which may be related to small cerebral vessel disease. Increases in arterial stiffness and carotid artery intima-media thickness (IMT) are the hallmark of arterial aging indicating the presence of subclinical atherosclerosis. The purpose of this study was to assess arterial stiffness, carotid artery IMT and WMH in patients with mild cognitive impairment (MCI).

Method(s): Twenty-eight MCI patients (14 males, age=6767yrs, clinical dementia rating score=0.5) and 30 age- and education-matched cognitively normal adults (14 males, age=6767yrs) participated. The carotid arterial (b) stiffness index and the intima-media thickness (IMT) were measured using high resolution 2D Doppler ultrasonography and applanation tonometry in the left and right common carotid arteries (CCA). Peripheral and central pulse-wave-velocity (PWV) were measured between the right common carotid artery and the right radial and left femoral artery, respectively. WMH was assessed in 22 patients with MCI (12 males, age=6767yrs) and 19 controls (5 males, age=6767yrs) using MRI Fluid-Attenuated-Inversion-Recovery (FLAIR) images on a 3T Philips Achieva MR system. Periventricular and deep-brain WMH volumes were quantified and differentiated using semi-automatic programs (MRICro and MatLab).

Result(s): MCI patients showed higher b-stiffness index (7.361.8 vs. 6.361.5 p<0.05) and decreased distensibility (0.3160.07 vs. 0.3760.09%mmHg, P = 0.01) in the CCA when compared to the cognitively normal subjects. No significant differences were found in the carotid artery IMT (0.7060.11 vs. 0.6660.10cm/s), peripheral (8.361.2 vs. 8.361.4cm/s) and central PWV (9.762.1 vs. 9.962.2cm/s) between the groups. Deep-brain WMH volume, normalized to the whole intracranium volume, was twice as high in the MCI group relative to the controls (dWMHx, 0.1860.10 vs. 0.0960.07%, P<0.01) although global WMH volume did not differ between the two groups.

Conclusion(s): Deep-brain WMH volume was increased in patients with MCI and was associated with increases in carotid arterial stiffness. The relationship between arterial stiffness and deep-brain WMH volume in MCI merits further investigation.

Institution

(Tseng, Ayaz, Brunk, Armstrong, Levine, Zhang) Institute for Exercise and Environmental Medicine, Dallas, TX, United States (Martin-Cook, Weiner, Cullum, Lu) University of Texas Southwestern Medical Center, Dallas, TX, United States

(Diaz-Arrastia) Uniformed Services University of the Health Sciences, Rockville, MD, United States

Publisher

Elsevier Inc.

For peer review only - http://bmjopen.bmj.com/site/about/guidelines.xhtml

Emtree Heading

*human; *white matter; *patient; *arterial stiffness; *mild cognitive impairment; carotid artery; brain; male; common carotid artery; arterial wall thickness; rigidity; femoral artery; pulse wave; artery intima; tonometry; Doppler flowmetry; brain blood vessel; adult; education; dementia; atherosclerosis; normal human; aging; nuclear magnetic resonance; liquid; nuclear magnetic resonance imaging.

Other Index Terms

*human; *white matter; *patient; *arterial stiffness; *mild cognitive impairment; carotid artery; brain; male; common carotid artery; arterial wall thickness; rigidity; femoral artery; pulse wave; artery intima; tonometry; Doppler flowmetry; brain blood vessel; adult; education; dementia; atherosclerosis; normal human; aging; nuclear magnetic resonance; liquid; nuclear magnetic resonance imaging.

For peer review only

Link to the Ovid Full Text or citation:

Click here for full text options

Link to the External Link Resolver:

SFX

249.

Increased intima-media thickness and central blood pressure in hypertensive individuals with cognitive impairment.

Vilela-Martin J.F., Cosenso-Martin L.N., Mota-Dias E., Giollo-Junior L.T., Cosenso-Sacomani C.N., Yugar-Toledo J.C., Moreno-Junior H.

Endocrine Reviews. Conference: 94th Annual Meeting and Expo of the Endocrine Society, ENDO 2012. Houston, TX United States. Conference Publication: (var.pagings). 33 (3 MeetingAbstracts) (no pagination), 2012. Date of Publication: June 2012.

AN: 70832504

Introduction: The role of hypertension in the loss of cognitive function is controversial. Relationships of hypertension with increases in cerebral vascular resistance, diffused lesions and multiple lacunar infarcts of the white matter are well known. Thus, the objectives of this study were: To evaluate the relationship between hypertension and cognitive dysfunction (CD), identify risk

factors and determine the association between markers of early vascular disease and CD in hypertensive individuals.

Method(s): Two hundred individuals aged between 40 and 80 years old were evaluated in this cross sectional study. Fifty participants were normotensive (NT). The remaining 150 hypertensive patients were subdivided into two groups, those with CD (HCD) and those without CD (HNCD). All participants underwent clinical evaluations and biochemical blood tests were performed. CD was investigated using the Mini Mental State Examination (MMSE) following the guidelines for its use in Brazil. The impact of hypertension on the arterial bed was assessed by identifying and measuring changes in the intima-media thickness (IMT) by vascular ultrasonography of the carotid arteries and analyses of the central blood pressure and Augmentation Index by applanation tonometry of the radial artery.

Result(s): There were no significant differences in the plasma concentrations of total cholesterol, high-density lipoprotein cholesterol and triglycerides of the three groups. The serum creatinine and estimated glomerular filtration rate were within normal ranges for all three groups. A significantly lower MMSE score was recorded for the HCD Group compared to the HNCD and NT Groups ($p<0.05$).The IMT was significantly different between the HNCD and HCD Groups ($p=0.0124$). A significant difference in the IMT was also observed between hypertensive patients and the NT Group ($p<0.0001$).The central systolic pressure was significantly higher in the HCD and HNCD Groups compared to NT Group ($p<0.0001$). There were no significant differences in the Augmentation Index (corrected for heart rate) between the three groups (HCD, HNCD and NT).

Conclusion s: Hypertensive patients with CD have changes in the vascular morphology characterized by an increased carotid IMT and hemodynamic functional impairment manifested by elevated central systolic blood pressure.

Institution

(Vilela-Martin, Cosenso-Martin, Mota-Dias, Giollo-Junior, Cosenso-Sacomani, Yugar-Toledo) Internal Medicine, State Medical School of Sao Jose do Rio Preto (FAMERP), Sao Jose do Rio Preto, Brazil (Moreno-Junior) Internal Medicine, Faculty of Medical Sciences, University of Campinas, Campinas, Brazil

Publisher

Endocrine Society

Emtree Heading

*blood pressure; *cognitive defect; *society; *arterial wall thickness; human; hypertension; patient; systolic blood pressure; augmentation index; carotid artery; Mini Mental State Examination; white matter; infarction; blood; morphology; heart rate; clinical evaluation; Brazil; echography; glomerulus filtration rate; cross-sectional study; brain vascular resistance; cholesterol blood level; blood level; vascular disease; creatinine blood level; radial artery; tonometry; risk factor; functional disease; cognition; high density lipoprotein cholesterol; triacylglycerol; marker.

For peer review only - http://bmjopen.bmj.com/site/about/guidelines.xhtml

Drug Index Terms

high density lipoprotein cholesterol; triacylglycerol; marker.

Other Index Terms

*blood pressure; *cognitive defect; *society; *arterial wall thickness; human; hypertension; patient; systolic blood pressure; augmentation index; carotid artery; mini mental state examination; white matter; infarction; blood; morphology; heart rate; clinical evaluation; Brazil; echography; glomerulus filtration rate; cross-sectional study; brain vascular resistance; cholesterol blood level; blood level; vascular disease; creatinine blood level; radial artery; tonometry; risk factor; functional disease; cognition.

Link to the Ovid Full Text or citation:

Click here for full text options

Link to the External Link Resolver:

SFX

252.

The effect of carotid endarterectomy on cognitive disturbances in patients with atherosclerotic stenosis of carotid arteries.

Yakhno N.N., Fedorova T.S., Damulin I.V., Shcherbiuk A.N., Vinogradov O.A., Lavrent'ev A.V.

Zhurnal Nevrologii i Psihiatrii imeni S.S. Korsakova. 111 (3) (pp 31-37), 2011. Date of Publication: 2011.

AN: 362503147

Clinical and neuropsychological features of non-dementia cognitive disturbances were studied in 102 patients with atherosclerotic carotid stenosis. Cognitive disturbances were assessed after the carotid endarterectomy (CEAE). Mild cognitive impairment was found in 37 (36,3%) of patients, moderate cognitive impairment was diagnosed in 36 (35,3%)patients. Moderate cognitive impairment was found more often in patients with symptomatic carotid stenosis with structural brain changes confirmed by neuroimaging data and with instable atherosclerotic plaques with the predomination of hypodensity component. It allows to suggest that both the reduction of perfusion and arterio-arterial microemboli may cause cognitive dysfunction in patients with atherosclerotic carotid stenosis. The data on the positive effect of CEAE on cognitive functions

For peer review only

For peer review only - http://bmjopen.bmj.com/site/about/guidelines.xhtml

have been obtained. The positive changes were more distinct in patients with asymptomatic course of carotid stenosis. However CEAE may have a negative effect on cognitive functions in patients with moderate cognitive impairment of dysmnestic character and symptomatic carotid stenosis.

PMID

21423113 [http://www.ncbi.nlm.nih.gov/pubmed/?term=21423113]

Publisher

Media Sfera (Dmitrovskoe shosse 46, korp 2, etazh 4, P.O. Box 54, Moscow 127238, Russian Federation)

Emtree Heading

amnesia/co [Complication]; article; atherosclerotic plaque/su [Surgery]; brain perfusion; brain region; *carotid artery obstruction/su [Surgery]; *carotid atherosclerosis/su [Surgery]; *carotid endarterectomy; clinical assessment; clinical feature; cognition; *cognitive defect/co [Complication]; controlled study; disease severity; human; major clinical study; microembolism; mild cognitive impairment/co [Complication]; neuroimaging; arterioarterial microembolus.

Candidate Terms

arterioarterial microembolus [other term].

Other Index Terms

amnesia / complication; article; atherosclerotic plaque / surgery; brain perfusion; brain region; *carotid artery obstruction / *surgery; *carotid atherosclerosis / *surgery; *carotid endarterectomy; clinical assessment; clinical feature; cognition; *cognitive defect / *complication; controlled study; disease severity; human; major clinical study; microembolism; mild cognitive impairment / complication; neuroimaging.

Link to the Ovid Full Text or citation:

Click here for full text options

Link to the External Link Resolver:

SFX

253.

Subclinical carotid atherosclerosis and cognitive function.

For peer review only - http://bmjopen.bmj.com/site/about/guidelines.xhtml

Arntzen K.A., Mathiesen E.B.
Acta Neurologica Scandinavica. 124 (SUPPL. 191) (pp 18-22), 2011. Date of Publication: August 2011.
AN: 362095535
Carotid artery atherosclerosis is a major risk factor for stroke and subsequent cognitive impairment. Recent studies indicate that carotid atherosclerosis without clinical stroke may also be an independent risk factor for cognitive decline and dementia. Ultrasonography is an easily assessable and non-invasive method to measure different stages of the carotid artery atherosclerotic process and is widely used in clinical assessment as well as in epidemiological and clinical research. We give a brief review of studies that have investigated degrees of the subclinical atherosclerosis in the carotid arteries in relation to cognitive function and dementia, and we discuss several possible mechanisms that could explain the association between atherosclerosis and cognitive impairment. © 2011 John Wiley & Sons A/S.
PMID
21711252 [http://www.ncbi.nlm.nih.gov/pubmed/?term=21711252]
Institution
(Arntzen) Department of Community Medicine, University of Tromso, Tromso, Norway (Arntzen, Mathiesen) Department of Neurology and Clinical Neurophysiology, University Hospital of North Norway, Tromso, Norway
(Mathiesen) Department of Clinical Medicine, University of Tromso, Tromso, Norway
Publisher
Blackwell Publishing Ltd (9600 Garsington Road, Oxford OX4 2XG, United Kingdom)
Emtree Heading
Alzheimer disease; arterial wall thickness; article; cardiovascular risk; carotid artery bifurcation; *carotid atherosclerosis; *cognition; dementia; disease association; human; mortality; nuclear magnetic resonance imaging.
Other Index Terms
Alzheimer disease; arterial wall thickness; article; cardiovascular risk; carotid artery bifurcation; *carotid atherosclerosis; *cognition; dementia; disease association; human; mortality; nuclear magnetic resonance imaging.

Link to the Ovid Full Text or citation:
Click here for full text options

Link to the External Link Resolver:
SFX

For peer review only

For peer review only - http://bmjopen.bmj.com/site/about/guidelines.xhtml

258.

Carotid atherosclerosis and cognitive function in midlife: The beaver dam offspring study.

Zhong W., Cruickshanks K.J., Huang G.-H., Klein B.E.K., Klein R., Nieto F.J., Pankow J.S., Schubert C.R.

Atherosclerosis. 219 (1) (pp 330-333), 2011. Date of Publication: November 2011.

AN: 51563811

Background: Atherosclerosis may be associated with cognitive function; however the studies are few, especially among midlife adults.

Method(s): Participants in the beaver dam offspring study who had cognitive test data and gradable carotid artery ultrasound scans were included (n= 2794, mean age: 49 years). Atherosclerosis was measured by carotid intima-media thickness (IMT) and the presence of plaque. Cognitive function was measured by the trail making test (TMT), grooved pegboard test (GPT) and mini-mental state examination (MMSE). Generalized cognitive function was defined by a summary score calculated from the TMT and GPT. Linear regression was used to evaluate the associations between carotid atherosclerosis and cognitive function tests.

Result(s): Larger IMT was associated with lower GPT, MMSE and the summary score adjusting for multiple factors, the coefficients were: 13.8s (p< 0.0001), -0.6 (p= 0.007), and 0.47 (p= 0.01), respectively for 1. mm increase in IMT. Plaque scores were significantly associated with TMT-B, GPT, MMSE, and the summary score adjusting for age, sex and education. The associations remained statistically significant after further adjustments except for the association with TMT-B, which was attenuated and no longer significant.

Conclusion(s): Our results show the significant associations between markers of carotid atherosclerosis and cognitive function in a cohort of persons aged 21-84 years. Longitudinal studies are needed to further examine these associations. © 2011 Elsevier Ireland Ltd.

PMID

21831374 [http://www.ncbi.nlm.nih.gov/pubmed/?term=21831374]

Institution

(Zhong, Cruickshanks, Klein, Klein, Nieto, Schubert) University of Wisconsin-Madison, 1036 WARF, 610 Walnut Street, Madison, WI 53726, United States (Huang) National Chiao Tung University, 1001 Ta Hsueh Road, Hsinchu 300, Taiwan (Republic of China)

(Pankow) University of Minnesota, MN 55454, United States

Publisher

Elsevier Ireland Ltd (P.O. Box 85, Limerick, Ireland)

For peer review only - http://bmjopen.bmj.com/site/about/guidelines.xhtml

Emtree Heading
adult; article; *carotid atherosclerosis; *cognition; disease association; female; human; major clinical study; male; middle aged; Mini Mental State Examination; priority journal; progeny; psychologic test; senile plaque.
Other Index Terms
adult; article; *carotid atherosclerosis; *cognition; disease association; female; human; major clinical study; male; middle aged; mini mental state examination; priority journal; progeny; psychologic test; senile plaque.

Link to the Ovid Full Text or citation:
Click here for full text options

Link to the External Link Resolver:
SFX

265.

Carotid atherosclerosis and incident cognitive impairment.
Zhong W., Schubert C.R., Cruickshanks K.J., Klein B.E.K., Klein R., Chappell R.J.
American Journal of Epidemiology. Conference: 3rd North American Congress of Epidemiology. Montreal, QC Canada. Conference Publication: (var.pagings). 173 (SUPPL. 11) (pp S246), 2011. Date of Publication: 01 Jun 2011.
AN: 70699780
Aim: To determine the relationship between atherosclerosis and incident cognitive impairment. Method(s): The Epidemiology of Hearing Loss Study (EHLS) is a population-based study among residents in Beaver Dam, WI. Participants had a carotid ultrasound scan (Biosound AU4) at the 1998-00 EHLS exam. The intima-media thickness (IMT) was evaluated at 12 sites in the carotid artery (the near and far walls of common carotid artery, the bifurcation and the internal carotid artery on the right and left sides); and the mean IMT of the 12 sites was used as an indicator of atherosclerosis. Cognitive function was measured at three EHLS exams (1998-00, 2003-05, 2009-10); and cognitive impairment was defined as the Mini-Mental State Examination (MMSE) score < 24 out of 30, or a proxy report of dementia. Participants without cognitive impairment at baseline and with a least one follow-up visit were included (n = 1651, mean age at baseline: 67 years) in the

For peer review only - http://bmjopen.bmj.com/site/about/guidelines.xhtml

analyses. The Cox proportional hazard model was used and the event's time was defined as the first examination at which a participant was cognitively impaired.

Result(s): There were 14,470 person-years of follow-up, with 143 cases of incident cognitive impairment. After adjusting for age, sex and education, IMT at baseline was associated with the 10-year cumulative incidence of cognitive impairment (hazard ratio: 1.08, 95% confidence interval: 1.01-1.16, per 100lm). The association was similar when further adjusting for smoking, drinking, serum HDL-cholesterol, A1C, and SF-36 mental score at baseline.

Conclusion(s): Carotid IMT was associated with the incidence of cognitive impairment, which suggests that interventions to prevent atherosclerosis may also have cognitive benefits.

Institution

(Zhong, Schubert, Cruickshanks, Klein, Klein, Chappell) University of WI-Madison, Madison, WI 53705, United States

Publisher

Oxford University Press

Emtree Heading

*cognitive defect; *epidemiology; *carotid atherosclerosis; human; carotid artery; atherosclerosis; follow up; education; hazard ratio; confidence interval; smoking; drinking; serum; hearing loss; population; mare; ultrasound; arterial wall thickness; internal carotid artery; cognition; Mini Mental State Examination; custody; dementia; proportional hazards model; examination; common carotid artery; high density lipoprotein cholesterol; high density lipoprotein.

Drug Index Terms

high density lipoprotein cholesterol; high density lipoprotein.

Other Index Terms

*cognitive defect; *epidemiology; *carotid atherosclerosis; human; carotid artery; atherosclerosis; follow up; education; hazard ratio; confidence interval; smoking; drinking; serum; hearing loss; population; mare; ultrasound; arterial wall thickness; internal carotid artery; cognition; mini mental state examination; custody; dementia; proportional hazards model; examination; common carotid artery.

Link to the Ovid Full Text or citation:

Click here for full text options

Link to the External Link Resolver:

SFX

For peer review only

269.

Carotid plaque progression predicts lower cognitive test results in a stroke-free population. the tromso study.

Arntzen K.A., Schirmer H., Johnsen S.H., Wilsgaard T., Mathiesen E.

European Journal of Neurology. Conference: 15th Congress of the EFNS. Budapest Hungary. Conference Publication: (var.pagings). 18 (SUPPL. 2) (pp 64), 2011. Date of Publication: September 2011.

AN: 70602489

Background: Atherosclerosis of the carotid arteries is a major risk factor for stroke and subsequent cognitive impairment. Whether subclinical carotid atherosclerosis predicts cognitive function is less clear.

Method(s): We followed a stroke-free middle-aged population of 4198 subjects who underwent carotid ultrasound examination at baseline (mean age 59y) and was re-examined with carotid ultrasound and tested for cognitive function 7 years later. Presence of plaques, the number of plaques and the total plaque area (TPA) at baseline, the average plaque scores between the two examinations and the progression of plaque formation were tested in regression models in order to predict cognitive test scores on verbal memory test, digit-symbol coding test and tapping test. Standardized regression coefficients (z-scores) were made for the dependent and independent variables to compare results between models and each cognitive test.

Result(s): In multivariable analysis adjusted for sex, age, education, depression and cardiovascular risk factors, baseline number of plaques and TPA were associated with lower test scores on the verbal memory test. The average number of plaques and TPA between baseline and follow-up were associated with lower cognitive test scores on all tests, and progression of the number of plaques predicted lower scores on the coding test and the tapping test.

Conclusion(s): In this stroke-free middle-aged general population, carotid plaques at baseline and progression of plaques were predictors of lower cognitive test scores.

Institution

(Arntzen, Schirmer, Wilsgaard) Department of Community Medicine, University of Tromso, Tromso, Norway (Arntzen, Johnsen, Mathiesen) Department of Neurology, University Hospital of North Norway, Tromso, Norway

(Schirmer) Department of Cardiology, University Hospital of North Norway, Tromso, Norway

(Johnsen, Mathiesen) Department of Clinical Medicine, University of Tromso, Tromso, Norway

Publisher

Blackwell Publishing Ltd

For peer review only

Emtree Heading
*carotid artery; *population; *stroke; examination; model; cognition; middle aged; ultrasound; verbal memory; carotid atherosclerosis; atherosclerosis; cardiovascular risk; follow up; independent variable; education; risk factor; cognitive defect.
Other Index Terms
*carotid artery; *population; *stroke; examination; model; cognition; middle aged; ultrasound; verbal memory; carotid atherosclerosis; atherosclerosis; cardiovascular risk; follow up; independent variable; education; risk factor; cognitive defect.

Link to the Ovid Full Text or citation:
Click here for full text options

Link to the External Link Resolver:
SFX

272.

Atherosclerosis and progression of brain atrophy: The SMART-MR study.
Muller M., Van Der Graaf Y., Algra A., Hendrikse J., Mali W., Geerlings M.
Alzheimer's and Dementia. Conference: Alzheimer's Association International Conference, AAIC 11. Paris France. Conference Publication: (var.pagings). 7 (4 SUPPL. 1) (pp S296), 2011. Date of Publication: July 2011.
AN: 70501570
Background: Atherosclerosis is believed to be involved in the etiology of ementia, including Alzheimer's disease. It is unknown to what extent neurodegenerative pathology underlies this association. Evidence from crosssectional tudies associating atherosclerosis with brain atrophy is limited and no prospective studies examined this relation. We investigated whether resence and severity of carotid atherosclerosis was related to progression of global, cortical and subcortical brain atrophy.
Method(s):Within the Second anifestations of Arterial disease-Magnetic Resonance (SMART-MR) study, a prospective cohort study among patients (mean age 58 (10) year, 0% men) with vascular disease, MRI of the brain was performed in 1232 patients at baseline (2001-2005) and in 663 patients at follow-up (2006- 009) after on average 3.9 years (range 3.0-5.8 years). At baseline,

For peer review only

measurements of carotid intima media thickness (CIMT) and carotid stenosis were erformed. Carotid stenosis was classified into 0-50%, 50-70% (moderate), and >70% (severe) and into unilateral or bilateral stenosis. Brain segmentation as used to quantify total brain volume, cortical gray matter volume, and ventricular volume as indicators of global, cortical, and subcortical atrophy. ll brain volumes were normalized for intracranial volume (ICV). Linear regression analysis was used to estimate cross-sectional and prospective ssociations of measures of carotid atherosclerosis with brain volumes, adjusted for age, sex, and vascular risk factors, and follow-up time in the rospective analysis.

Result(s): Increased CIMTand carotid stenosis were associated with decreased total brain and cortical gray matter volume in crosssectional nalyses. Prospective analyses showed that CIMT and moderate stenosis were not related to progression of brain atrophy. Compared with patients ith no or limited carotid stenosis, only severe or bilateral carotid stenosis was related to progression of global atrophy, cortical atrophy and ubcortical atrophy.

Conclusion(s): We confirmed previous cross-sectional findings of more brain atrophy with CIMT and stenosis. However, our prospective indings in a study of patients with vascular disease with 4 years of follow-up showed that only severe or bilateral carotid stenosis, and not moderate arotid stenosis and increased CIMT, were associated with progression of brain atrophy. These findings suggest that high grade carotid artery narrowing ontributes to the progression of neurodegeneration.

Institution

(Muller) VU University Medical Center, Amsterdam, Netherlands (Van Der Graaf, Algra, Hendrikse, Mali, Geerlings) University Medical Center Utrecht, Utrecht, Netherlands

Publisher

Elsevier Inc.

Emtree Heading

*brain atrophy; *atherosclerosis; *nuclear magnetic resonance; carotid artery obstruction; human; patient; stenosis; atrophy; brain size; brain; follow up; carotid atherosclerosis; carotid artery; gray matter; vascular disease; etiology; pathology; prospective study; artery disease; cohort analysis; male; arterial wall thickness; linear regression analysis; artery occlusion; nerve degeneration; risk factor; Alzheimer disease; nuclear magnetic resonance imaging.

Other Index Terms

*brain atrophy; *atherosclerosis; *nuclear magnetic resonance; carotid artery obstruction; human; patient; stenosis; atrophy; brain size; brain; follow up; carotid atherosclerosis; carotid artery; gray matter; vascular disease; etiology; pathology; prospective study; artery disease; cohort analysis; male; arterial wall thickness; linear regression analysis; artery occlusion; nerve degeneration; risk factor; Alzheimer disease; nuclear magnetic resonance imaging.

For peer review only - http://bmjopen.bmj.com/site/about/guidelines.xhtml

Link to the Ovid Full Text or citation:

Click here for full text options

Link to the External Link Resolver:

SFX

273.

Arterial calcifications in relation to cognitive function and structural brain changes.

Bos D., Van Der Lugt A., Witteman J., Krestin G., Hofman A., Vernooij M., Ikram M., Medical E.

Alzheimer's and Dementia. Conference: Alzheimer's Association International Conference, AAIC 11. Paris France. Conference Publication: (var.pagings). 7 (4 SUPPL. 1) (pp S295), 2011. Date of Publication: July 2011.

AN: 70501568

Background: Atherosclerosis may play an important role in the etiology of ognitive decline and dementia. This study investigates associations between atherosclerosis in four vessel beds outside the brain, with cognition nd preclinical MRI-markers of dementia.

Method(s): From the general population, 863 participants (mean age 66.6 years) underwent non-enhanced T of the coronaries, aortic arch, extracranial and intracranial carotid arteries to quantify calcification volume as a measure of atherosclerosis. ognitive function was assessed with a neuropschycological test battery comprising the following domains: memory, executive function, information rocessing speed, global cognition and motor speed. On brain MRI, total brain volume (TBV), grey matter volume (GM), white matter volume WM) and hippocampal volume were assessed. Associations between arterial calcifications (stratified by gender), cognition and brain issue volumes were assessed with linear regression, adjusted for relevant confounders.

Result(s): Higher CT-assessed calcification load in all vessel eds was associated with worse cognitive scores in all domains. Adjustment for total brain volume attenuated these associations, except or the associations between extracranial and intracranial carotid artery calcifications and motor speed in men. A higher load of extracranial carotid rtery calcifications in men and intracranial carotid artery calcifications in women was significantly associated with smaller total brain volume nd smaller white matter volume. In women, aortic calcifications were strongly associated with smaller grey matter volume. Calcifications in ny vessel bed were not associated with hippocampal volume. Adjustment for cardiovascular risk factors or carotid plaque did not change these associations.

For peer review only - http://bmjopen.bmj.com/site/about/guidelines.xhtml

onclusions: A higher arterial calcification load is associated with worse cognitive function. Furthermore, calcification load in specific essel beds is associated with smaller total brain volume, white matter volume and grey matter volume. The association between arterial calcification oad and cognitive function is partly mediated by its effect on brain tissue volumes.

Institution

(Bos, Van Der Lugt, Witteman, Krestin, Hofman, Vernooij, Ikram, Medical) Centre, Rotterdam, Netherlands

Publisher

Elsevier Inc.

Emtree Heading

*artery calcification; *cognition; *brain; calcification; human; carotid artery; brain size; velocity; gray matter; white matter; atherosclerosis; dementia; female; male; nuclear magnetic resonance imaging; population; aorta arch; memory; executive function; gender; linear regression analysis; cardiovascular risk; brain tissue; etiology; marker.

Drug Index Terms

marker.

Other Index Terms

*artery calcification; *cognition; *brain; calcification; human; carotid artery; brain size; velocity; gray matter; white matter; atherosclerosis; dementia; female; male; nuclear magnetic resonance imaging; population; aorta arch; memory; executive function; gender; linear regression analysis; cardiovascular risk; brain tissue; etiology.

Link to the Ovid Full Text or citation:

Click here for full text options

Link to the External Link Resolver:

SFX

274.

Carotid arterial plaque and clinical factors in stroke patients with dementia.

Lee J.H.

For peer review only - http://bmjopen.bmj.com/site/about/guidelines.xhtml

Alzheimer's and Dementia. Conference: Alzheimer's Association International Conference, AAIC 11. Paris France. Conference Publication: (var.pagings). 7 (4 SUPPL. 1) (pp S171), 2011. Date of Publication: July 2011.

AN: 70501186

Background: Carotid arterial stenosis becomes more common and important risk factor for stroke patients with dementia in Asian area.We reviewed stroke database to investigate clinical factors related to carotid arterial stenosis, including intracranial arterial stenosis and peripheral arterial disease which reflects advanced atherosclerosis in patients with dementia.

Method(s): Acute stroke patients with dementia whose stroke onset were within 1 week when admitted at the National Health Insurance Corporation Ilsan Hospital from January 2008 to December 2010 with available carotid ultrasound study, transcranial Doppler (TCD) examination and ankle-brachial indexes (ABI) formed the analysis cohorts. Retrospective review was performed.

Result(s): A total of 304 patients were included during that period. By duplex ultrasound, common/internal carotid arteries are examined and the greatest diameter of plaques are recorded. 3 groups of carotid arterial plaques are defined: diameter is less than 2mm (112 patients, 37%), 2-4mm (174 patients, 57%) and greater than 4mm (18 patients, 6%). As the size of carotid arterial plaques increased, ABI is decreased (P = 0.000) and the number of intracranial arterial stenosis is increased (P = 0.008). Among the risk factors, Age, diabetes, male patients are increased (P = 0.000, P = 0.047, P = 0.004) and smoking history showed tendency of increase (P = 0.057) as diameter of carotid arterial plaque increase. However hypertension, total cholesterol, LDL cholesterol, HDL cholesterol, triglyceride and past stroke history are not correlated with carotid arterial stenosis.

Conclusion(s): Among the acute stroke patients with dementia, more than a half of them have carotid arterial plaque which diameters are greater than 2mm and these patients tend to have higher burden of advanced atherosclerosis as evidenced by a higher prevalence of diabetes, intracranial arterial stenosis and peripheral arterial occlusive disease.

Institution

(Lee) Ilsan Hospital, Koyang-Shi, Kyonggi-do, South Korea

Publisher

Elsevier Inc.

Emtree Heading

*carotid artery; *dementia; *stroke patient; human; patient; artery occlusion; stroke; risk factor; atherosclerosis; ultrasound; diabetes mellitus; peripheral occlusive artery disease; national health insurance; hospital; data base; Doppler echography; examination; ankle brachial index; cohort analysis; Asian; male; smoking; hypertension; cholesterol blood level; prevalence; low density lipoprotein cholesterol; high density lipoprotein cholesterol; high density lipoprotein; triacylglycerol; low density lipoprotein.

For peer review only

Drug Index Terms
low density lipoprotein cholesterol; high density lipoprotein cholesterol; high density lipoprotein; triacylglycerol; low density lipoprotein.
Other Index Terms
*carotid artery; *dementia; *stroke patient; human; patient; artery occlusion; stroke; risk factor; atherosclerosis; ultrasound; diabetes mellitus; peripheral occlusive artery disease; national health insurance; hospital; data base; Doppler echography; examination; ankle brachial index; cohort analysis; Asian; male; smoking; hypertension; cholesterol blood level; prevalence.

Link to the Ovid Full Text or citation:
Click here for full text options

Link to the External Link Resolver:
SFX

276.

Arterial calcifications in relation to cognitive function and structural brain changes.
Bos D., Van der Lugt A., Witteman J., Krestin G., Hofman A., Vernooij M., Ikram M.
Alzheimer's and Dementia. Conference: Alzheimer's Association International Conference, AAIC 11. Paris France. Conference Publication: (var.pagings). 7 (4 SUPPL. 1) (pp S71-S72), 2011. Date of Publication: July 2011.
AN: 70500894
Background: Atherosclerosis may play an important role in the etiology of cognitive decline and dementia. This study investigates associations between atherosclerosis in four vessel beds outside the brain, with cognition and preclinical MRI-markers of dementia.
Method(s): From the general population, 863 participants (mean age 66.6 years) underwent nonenhanced CT of the coronaries, aortic arch, extracranial and intracranial carotid arteries to quantify calcification volume as a measure of atherosclerosis. Cognitive function was assessed with a neuropschycological test battery comprising the following domains: memory, executive function, information processing speed, global cognition and motor speed. On brain MRI, total brain volume (TBV), grey matter volume (GM), white matter volume (WM) and hippocampal volume

For peer review only - http://bmjopen.bmj.com/site/about/guidelines.xhtml

were assessed. Associations between arterial calcifications (stratified by gender), cognition and brain tissue volumes were assessed with linear regression, adjusted for relevant confounders.
Result(s): Higher CT-assessed calcification load in all vessel beds was associated with worse cognitive scores in all domains. Adjustment for total brain volume attenuated these associations, except for the associations between extracranial and intracranial carotid artery calcifications and motor speed in men. A higher load of extracranial carotid artery calcifications in men and intracranial carotid artery calcifications in women was significantly associated with smaller total brain volume and smaller white matter volume. In women, aortic calcifications were strongly associated with smaller grey matter volume. Calcifications in any vessel bed were not associated with hippocampal volume. Adjustment for cardiovascular risk factors or carotid plaque did not change these associations.
Conclusion(s): A higher arterial calcification load is associated with worse cognitive function. Furthermore, calcification load in specific vessel beds is associated with smaller total brain volume, white matter volume and grey matter volume. The association between arterial calcification load and cognitive function is partly mediated by its effect on brain tissue volumes.
Institution
(Bos, Van der Lugt, Witteman, Krestin, Hofman, Vernooij, Ikram) Erasmus Medical Centre, Rotterdam, Netherlands
Publisher
Elsevier Inc.
Emtree Heading
*artery calcification; *cognition; *brain; human; calcification; carotid artery; brain size; atherosclerosis; white matter; gray matter; velocity; female; brain tissue; male; dementia; nuclear magnetic resonance imaging; population; aorta arch; memory; executive function; information processing; gender; linear regression analysis; cardiovascular risk; etiology; marker.
Drug Index Terms
marker.
Other Index Terms
*artery calcification; *cognition; *brain; human; calcification; carotid artery; brain size; atherosclerosis; white matter; gray matter; velocity; female; brain tissue; male; dementia; nuclear magnetic resonance imaging; population; aorta arch; memory; executive function; information processing; gender; linear regression analysis; cardiovascular risk; etiology.

Link to the Ovid Full Text or citation:
Click here for full text options

For peer review only - http://bmjopen.bmj.com/site/about/guidelines.xhtml

Link to the External Link Resolver:

SFX

277.

Enhanced carotid plaque echolucency is associated with worse cognitive performance in elderly patients with atherosclerotic disease.

Desideri G., Mastroiacovo D., Pinelli M., Grassi D., Turco G.L., Camerota A., D'Andrea M., Andriulli M., Petrella I., Marini C., Lechiara M.C., Ghiadoni L., Bocale R., De Blasis G., Ferri C.

High Blood Pressure and Cardiovascular Prevention. Conference: National Congress of the Italian Society for Cardiovascular Prevention, SIPREC 2011. Genova Italy. Conference Publication: (var.pagings). 18 (2) (pp 77), 2011. Date of Publication: 01 Jun 2011.

AN: 70497061

Introduction: Numerous studies have demonstrated the existence of a relationship between echogenicity of carotid plaque and cerebral ischaemic events, both silent and clinically manifest. These, in turn, expose the patient to increased risk of developing cognitive impairment and dementia in geriatric age.

Aim(s): Based on this scientific evidence we decided to investigate the relationship between echogenicity of carotid plaques and cognitive performance in patients with carotid atheromatous plaques but with no history of cerebrovascular events and/or clinical evidence for dementia.

Method(s): We studied 93 individuals aged 65 years or more (72.6+/-; 5.2 years) referred to our angiology unit for atheroma of neck vessels. At enrolment, patients underwent ultrasonography of the neck vessels using a 7-8 MHz linear probe; images were stored on magneto-optical disk and subsequently used to calculate the grey scale median (GSM) using Adobe Photoshop 5.0. The calculation of the GSM was performed by researchers blinded to the clinical characteristics of patients. All participants were also subjected to the study of cognitive function by Mini Mental State Examination (MMSE), Trail Making Test (TMT) A and B and verbal fluency test (VFT). The results of psychometric tests were logarithmically transformed and used to calculate a composite cognitive score.

Result(s): Patients were divided into two groups according to echolucency of carotid plaques used as the discriminating median GSM obtained in the study population (33.5+/-;6.6 vs 60.4+/-; 13.1, respectively, p<0.0001). Cognitive performance was on average worse in patients with more plaque echolucency (MMSE: 28.5+/-;1.4 vs 28.6+/-;1.4, p = ns; TMTA 92.5+/-;27.2 vs 64.3+/-;31.1, p<0.001; TMTB 214.2+/-;76.6 vs 147.1 +/-;55.9, p<0.005; VFT 33.5+/-;8.3 vs 41.3+/-;9.5, p<0.004; z score: -

For peer review only - http://bmjopen.bmj.com/site/about/guidelines.xhtml

0276 +/-;0540 vs 0398 +/-;0598, p< 0.0001). Considering the study population as a whole, we observed a direct correlation between GSM and cognitive performance (r: 0.526, p<0.001).
Conclusion(s): The results of our study demonstrate the existence of an inverse relationship between echolucency of carotid plaques and cognitive function in the elderly and suggest the possible use of this method to identify subjects at increased risk of developing dementia.

Institution

(Desideri, Mastroiacovo, Grassi, Camerota, D'Andrea, Andriulli, Petrella, Marini, Bocale, Ferri) Universita degli Studi dell'Aquila, Dipartimento di Medicina Interna e Sanita Pubblica, L'Aquila, Italy
(Pinelli, Turco, De Blasis) Ospedale Civile di Avezzano, Avezzano, Italy
(Lechiara) Ospedale S. Rinaldi, Pescina, Italy
(Ghiadoni) Universita degli Studi di Pisa, Dipartimento di Medicina Interna, Pisa, Italy

Publisher

Adis International Ltd

Emtree Heading

*carotid artery; *prevention; *society; *aged; human; patient; dementia; population; cognition; neck; risk; cognitive defect; atherosclerotic plaque; angiology; atheroma; echography; optical disk; Mini Mental State Examination; psychologic test; psychometry.

Other Index Terms

*carotid artery; *prevention; *society; *aged; human; patient; dementia; population; cognition; neck; risk; cognitive defect; atherosclerotic plaque; angiology; atheroma; echography; optical disk; mini mental state examination; psychologic test; psychometry.

Link to the Ovid Full Text or citation:

Click here for full text options

Link to the External Link Resolver:

SFX

278.

Carotid atherosclerosis is associated with lower cognitive test results in a stroke-free middle-aged population. the tromso study.

Arntzen K.A., Schirmer H., Johnsen S.H., Wilsgaard T., Mathiesen E.B.

For peer review only - http://bmjopen.bmj.com/site/about/guidelines.xhtml

Cerebrovascular Diseases. Conference: 20th European Stroke Conference, ESC 2011. Hamburg Germany. Sponsor: Bayer, Boehringer, ev3, Allergan, Pfizer, et al. . Conference Publication: (var.pagings). 31 (SUPPL. 2) (pp 159-160), 2011. Date of Publication: May 2011.

AN: 70432648

Background: Carotid artery atherosclerosis is a major risk factor for stroke and subsequent cognitive impairment. Prospective studies indicate that also subclinical carotid atherosclerosis is associated with a higher risk of cognitive decline and dementia in elderly persons. The relationship between degrees of carotid atherosclerosis and cognitive function in a middle-aged general population is less known.

Method(s): In a prospective study we followed a stroke-free middle-aged population of 4371 participants (mean age 59 yrs) who at baseline underwent carotid ultrasound examination and assessment of cardiovascular risk factors and 7 years later tests of cognitive function. Associations between intima-media thickness (IMT), number of plaques and total plaque area and cognitive test scores on verbal memory, digit-symbol coding and tapping tests were assessed in linear regression models.

Result(s): Presence of plaque and number of plaques were associated with lower test scores on the verbal memory test and on the digit-symbol coding test in the multivariable regression model adjusted for sex, age, education, depression and cardiovascular risk factors. Total plaque area was associated with lower cognitive scores on the verbal memory test, whereas IMT was associated with lower scores on the digit-symbol coding test. No significant association was seen between carotid atherosclerosis and the tapping test scores.

Conclusion(s): In this middle-aged general population we found that subclinical carotid atherosclerosis measured as IMT, number of plaques and total plaque area were independent risk factors for lower cognitive test scores after 7 years of follow-up.

Institution

(Arntzen, Schirmer) Department of Community Medicine, University of Tromso, University Hospital of North Norway, Tromso, Norway (Johnsen) Department of Neurology and Neurophysiology, University Hospital of North Norway, University of Tromso, Tromso, Norway

(Wilsgaard) Department of Community Medicine, University of Tromso, Tromso, Norway

(Mathiesen) Department of Clinical Medicine, University of Tromso, University Hospital of North Norway, Tromso, Norway

Publisher

S. Karger AG

Emtree Heading

*middle aged; *stroke; *carotid atherosclerosis; *population; verbal memory; prospective study; model; risk factor; cognition; cardiovascular risk; follow up; cognitive defect; risk; dementia; aged; carotid artery; ultrasound; examination; arterial wall thickness; linear regression analysis; education.

For peer review only - http://bmjopen.bmj.com/site/about/guidelines.xhtml

Other Index Terms

*middle aged; *stroke; *carotid atherosclerosis; *population; verbal memory; prospective study; model; risk factor; cognition; cardiovascular risk; follow up; cognitive defect; risk; dementia; aged; carotid artery; ultrasound; examination; arterial wall thickness; linear regression analysis; education.

Link to the Ovid Full Text or citation:

Click here for full text options

Link to the External Link Resolver:

SFX

For peer review only

281.

Infectious burden and cognition: The northern manhattan study.

Katan M., Moon Y.P., Paik M.C., Gervasi-Franklin P., Sacco R.L., Wright C.B., Elkind M.S.

Stroke. Conference: 2011 International Stroke Conference. Los Angeles, CA United States. Conference Publication: (var.pagings). 42 (3) (pp e163), 2011. Date of Publication: 01 Mar 2011.

AN: 70362597

Background: A composite measure of several chronic infections (infectious burden, or IB) is associated with risk of stroke and carotid atherosclerosis in our cohort. The association of IB with cognitive impairment and dementia remains mostly unexplored, however. We hypothesized that a measure of IB associated with vascular risk would also be associated with cognition and cognitive decline in a prospective cohort study.

Method(s): Crosssectional and prospective analyses among stroke-free community participants in the multi-ethnic Northern Manhattan Study were performed. Cognition was assessed using both the Mini Mental State Exam (MMSE) at enrolment and the modified Telephone Interview for Cognitive Status (TICS-m) during annual telephone follow up . IB was calculated based on a composite measure of serologies against microbial agents previously shown to be associated with risk of stroke and carotid plaque (i.e. Chlamydia pneumoniae, Helicobacter pylori, CMV, HSV1 and 2). Linear and logistic regression were used to measure the magnitude of association between IB and cognition after adjusting for other risk factors, and generalized estimating equation models were used to evaluate associations with TICS-m and its change over time.

Result(s): Both serologies and cognitive assessments were available in 1623 participants (mean age 68.5+/-10.1 yrs, 64.9% women). Median MMSE was 27 (interquartile range (IQR) 24-29) and median TICS-m 32 (IQR 27-36). In the unadjusted model IB index was associated with MMSE and TICS-m (both P<0.0001; see table). These effects were attenuated after adjusting for demographics, and there was little change after further adjusting for vascular risk factors. The effect of IB remained significant for TICS-m (p<0.0001; see table). IB was associated with MMSE =<24 (adjusted OR 1.22, 95% confidence interval 1.03-1.45). However, IB was not associated with cognitive decline over time (p=0.07).

Conclusion(s): A measure of infectious burden that is associated with vascular disease risk was independently and inversely associated with cognitive performance in this multi-ethnic cohort, though it was not associated with further cognitive decline. Past infection and associated inflammationrelated vascular damage may contribute to cognitive impairment in the elderly. (Table presented).

Institution

(Katan, Moon, Gervasi-Franklin, Elkind) Columbia Univ., Dept. of Neurology, New York, NY, United States (Paik) Columbia Univ., Dept. of Biostatistics, Mailman Sch. of Public Health, New York, NY, United States

(Sacco) Univesity of Miami, Dept. of Neurology and Epidemiology an Genetics, Miller Sch. of Medicine, Miami, FL, United States

(Wright) Univ. of Miami, Dept. of Neurology, Miller Sch. of Medicine, Miami, FL, United States

Publisher

Lippincott Williams and Wilkins

Emtree Heading

*cognition; *stroke; risk; Mini Mental State Examination; telephone; infection; serology; model; cognitive defect; risk factor; Chlamydophila pneumoniae; blood vessel injury; aged; carotid atherosclerosis; dementia; cohort analysis; interview; tic; follow up; carotid artery; mental health; Helicobacter pylori; Herpes simplex virus 1; logistic regression analysis; female; confidence interval; vascular disease; community.

Other Index Terms

*cognition; *stroke; risk; mini mental state examination; telephone; infection; serology; model; cognitive defect; risk factor; Chlamydophila pneumoniae; blood vessel injury; aged; carotid atherosclerosis; dementia; cohort analysis; interview; tic; follow up; carotid artery; mental health; Helicobacter pylori; Herpes simplex virus 1; logistic regression analysis; female; confidence interval; vascular disease; community.

Link to the Ovid Full Text or citation:

For peer review only - http://bmjopen.bmj.com/site/about/guidelines.xhtml

Click here for full text options

Link to the External Link Resolver:
SFX

284.

Carotid artery atherosclerosis, MRI indices of brain ischemia, aging, and cognitive impairment: The framingham study.
Romero J.R., Beiser A., Seshadri S., Benjamin E.J., Polak J.F., Vasan R.S., Au R., Decarli C., Wolf P.A.
Stroke. 40 (5) (pp 1590-1596), 2009. Date of Publication: 01 May 2009.
AN: 354869796
Background and Purpose-: Carotid atherosclerosis has been associated with increased risk of stroke and poorer cognitive performance in older adults. The relation of carotid atherosclerosis to cognitive impairment and MRI indices of ischemia and aging in midlife is less clear. Methods-: We studied 1975 Framingham Offspring Study participants free of stroke and dementia with available carotid ultrasound, brain MRI, and neuropsychological testing. We related common and internal carotid artery intima-media thickness and internal carotid stenosis to large white matter hyperintensity (>1 SD above age-specific mean), total brain volume, hippocampal volume, silent cerebral infarcts, and neuropsychological measures of verbal memory, executive function, and nonverbal memory measures. Results-: We observed that internal carotid artery intima-media thickness, but not common carotid artery intima-media thickness, was associated with higher prevalence of silent cerebral infarcts (OR, 1.21; 95% CI, 1.03-1.43; P<0.05), large white matter hyperintensity (OR, 1.19; 95% CI, 1.03-1.38; P<0.05), lower total brain volume (-0.05 per SD; P<0.05), and poorer performance in verbal memory (-0.06 per SD; P<0.05) and nonverbal memory measures (-0.08 per SD; P<0.01), but not with hippocampal volume. Internal carotid stenosis =25% was associated with a higher prevalence of large white matter hyperintensity (adjusted OR, 1.77; 95% CI, 1.25-2.53) and lower total brain volume (-0.11 per SD; P=0.042) but not with silent cerebral infarcts or hippocampal volume. Internal carotid stenosis =50% was associated with higher prevalence of silent cerebral infarcts (OR, 2.53; 95% CI, 1.17-5.44), large white matter hyperintensity (OR, 2.35; 95% CI, 1.08-5.13), and poorer performance on executive function (-0.39 per SD; P<0.05), but not with total brain volume or hippocampal volume. Conclusions-: Carotid atherosclerosis markers were associated with MRI indices of brain ischemia and aging and with cognitive impairment in a community-based sample of middle-aged adults. Our data suggest that

For peer review only - http://bmjopen.bmj.com/site/about/guidelines.xhtml

internal carotid artery intima-media thickness may be a better marker for cognitive impairment than common carotid artery intima-media thickness. © 2009 American Heart Association, Inc.

PMID

19265054 [http://www.ncbi.nlm.nih.gov/pubmed/?term=19265054]

Institution

(Romero, Seshadri, Au, Wolf) Department of Neurology, Boston University, Boston, MA (Benjamin, Vasan) Section of Preventive Medicine, Boston University, Boston, MA
(Benjamin, Vasan) Section of Cardiology, Boston University, Boston, MA
(Beiser) School of Medicine, Department of Biostatistics, Boston University, Boston, MA
(Decarli) Department of Neurology, University of California-Davis, Davis, CA
(Polak) Department of Radiology, Tufts University, School of Medicine, Boston, MA
(Romero, Beiser, Benjamin, Vasan, Au, Wolf) NHLBI's Framingham Heart Study, Framingham, MA
(Romero) Department of Neurology, Boston University, School of Medicine, 72 East Concord St, D410-B, Boston, MA 02118

Publisher

Lippincott Williams and Wilkins (530 Walnut Street,P O Box 327, Philadelphia PA 19106-3621, United States)

Emtree Heading

adult; aging; artery intima proliferation; article; *atherosclerosis; brain infarction; *brain ischemia; brain size; carotid artery obstruction; *cognitive defect; controlled study; dementia; disease marker; female; hippocampus; human; internal carotid artery; major clinical study; male; neuropsychology; nuclear magnetic resonance imaging; prevalence; priority journal; ultrasound; verbal memory; white matter.

Other Index Terms

adult; aging; artery intima proliferation; article; *atherosclerosis; brain infarction; *brain ischemia; brain size; carotid artery obstruction; *cognitive defect; controlled study; dementia; disease marker; female; hippocampus; human; internal carotid artery; major clinical study; male; neuropsychology; nuclear magnetic resonance imaging; prevalence; priority journal; ultrasound; verbal memory; white matter.

Link to the Ovid Full Text or citation:

Click here for full text options

Link to the External Link Resolver:

SFX

For peer review only

For peer review only - http://bmjopen.bmj.com/site/about/guidelines.xhtml

291.

Experimental models of vascular dementia and vascular cognitive impairment: A systematic review.
Jiwa N.S., Garrard P., Hainsworth A.H.
Journal of Neurochemistry. 115 (4) (pp 814-828), 2010. Date of Publication: November 2010.
AN: 359840579
Vascular cognitive impairment (VCI) encompasses vascular dementia and is the second most common cause of dementing illness after Alzheimer's disease. The main causes of VCI are: cerebral small vessel disease; multi-infarct dementia; strategic infarct (i.e. located in a functionally-critical brain area); haemorrhage/microbleed; angiopathy (including cerebral amyloid angiopathy); severe hypoperfusion (e.g. cardiac arrhythmia); and hereditary vasculopathy (e.g. cerebral autosomal dominant arteriopathy with subcortical infarcts and leukoencephalopathy, CADASIL). In this systematic analysis, we aimed to relate cognitive and neuropathological features of experimental models to clinical VCI. We extracted data from 107 studies covering 16 models. These included: brief global ischaemic insults (in rats, mice or gerbils); chronic global hypoperfusion (rats, mice, gerbils); chronic hypertension (in primates or stroke-prone, spontaneously-hypertensive rats); multiple ischaemic lesions because of intra-vascular emboli (in rodents, rabbits or primates); strategic ischaemic lesions (in rats or mini-pigs); generalised vasculopathies, because of mutant Notch3, hyperhomocysteinaemia, experimental diabetes mellitus or lack of cerebral vasodilator M5 receptors (rats or mice). Most cognitive testing showed deficits in working and reference memory. The lesions observed were microinfarcts, diffuse white matter lesions, hippocampal neuronal death, focal ischaemic lesions and micro-haemorrhages. The most-used model was bilateral carotid artery occlusion in rats, leading to chronic hypoperfusion and white matter injury. © 2010 The Authors. Journal of Neurochemistry © 2010 International Society for Neurochemistry.
PMID
20731763 [http://www.ncbi.nlm.nih.gov/pubmed/?term=20731763]
Institution
(Jiwa, Garrard, Hainsworth) Clinical Neuroscience, Division of Clinical Sciences, St George's University of London, London, United Kingdom
Publisher
Blackwell Publishing Ltd (9600 Garsington Road, Oxford OX4 2XG, United Kingdom)
Emtree Heading
atherosclerosis; brain embolism; CADASIL; carotid artery obstruction; cognitive defect/dt [Drug Therapy]; *dementia; gerbil; hippocampus; hyperhomocysteinemia; hypertension; memory

disorder/dt [Drug Therapy]; middle cerebral artery occlusion; *multiinfarct dementia; neuropsychology; priority journal; review; streptozocin diabetes; stroke; systematic review; transgenic mouse; vascular amyloidosis; white matter; candesartan/dt [Drug Therapy]; cilostazol/dt [Drug Therapy]; endothelin 1; muscarinic M5 receptor/ec [Endogenous Compound]; Notch3 receptor/ec [Endogenous Compound]; *vascular cognitive impairment.

Candidate Terms

*vascular cognitive impairment [other term].

Drug Index Terms

candesartan / drug therapy; cilostazol / drug therapy; endothelin 1; muscarinic M5 receptor / endogenous compound; Notch3 receptor / endogenous compound.

Other Index Terms

atherosclerosis; brain embolism; CADASIL; carotid artery obstruction; cognitive defect / drug therapy; *dementia; gerbil; hippocampus; hyperhomocysteinemia; hypertension; memory disorder / drug therapy; middle cerebral artery occlusion; *multiinfarct dementia; neuropsychology; priority journal; review; streptozocin diabetes; stroke; systematic review; transgenic mouse; vascular amyloidosis; white matter.

Link to the Ovid Full Text or citation:

Click here for full text options

Link to the External Link Resolver:

SFX

295.

Carotid- intima media thickness is independently associated with cognitive decline. The INVADE study.

Sander K., Bickel H., Forstl H., Etgen T., Briesenick C., Poppert H., Sander D.

International Journal of Geriatric Psychiatry. 25 (4) (pp 389-394), 2010. Date of Publication: April 2010.

AN: 358470612

Objectives: Increased carotid intima-media thickness (C-IMT) is a non-invasive marker of atherosclerosis and predicts vascular events. Moreover, increasing evidence suggests an

For peer review only - http://bmjopen.bmj.com/site/about/guidelines.xhtml
For peer review only

association between carotid atherosclerosis and cognitive decline. The purpose of this study is to investigate the relationship between C-IMT and the development of cognitive impairment in a large population-based sample.
Method(s): This study was based on the data of the participants of the INVADE (Intervention project on cerebrovascular diseases and dementia in the district of Ebersberg, Bavaria) project. Vascular risk factors, Geriatric depression scale (GDS) and "6 Item Cognitive Impairment Test" (6CIT) were evaluated at baseline and after 2 years. The relationship between C-IMT and cognitive impairment was analysed using multivariate logistic regression.
Result(s): Complete baseline data were available in 3386 subjects (mean age 67.7 [95% confidence interval (CI): 67.5, 68.0] years, 41% male). During follow-up, 174 subjects developed a new cognitive impairment. In the subgroup without cognitive impairment at baseline a significant association between cognitive decline after 2 years and elevated C-IMT at baseline could be detected with a significantly higher baseline C-IMT in those with cognitive decline (0.87mm vs. 0.78 mm; $p < 0.0001$). After adjustment for various risk factors only age, GDS baseline 6CIT and C-IMT were independently associated with the development of a new cognitive impairment.
Conclusion(s): Our data indicate that an increased carotid intima-media thickness predicts a cognitive decline in an elderly population without prevalent cognitive impairment. Copyright © 2009 John Wiley & Sons, Ltd.

PMID

19750556 [http://www.ncbi.nlm.nih.gov/pubmed/?term=19750556]

Institution

(Sander, Poppert, Sander) Department of Neurology, Technical University, Munich, Germany

(Bickel, Forstl, Etgen) Department of Psychiatry and Psychotherapy, Technical University, Munich, Germany

(Briesenick) INVADE Study Group, Baldham, Germany

(Sander, Sander) Department of Neurology, Medical Park Hospital, Bischofswiesen, Germany

(Etgen) Department of Neurology, Klinikum Traunstein, Germany

Publisher

John Wiley and Sons Ltd (Southern Gate, Chichester, West Sussex PO19 8SQ, United Kingdom)

Emtree Heading

aged; *arterial wall thickness; *artery media; article; cardiovascular risk; *carotid artery; *cognitive defect; dementia; depression; diabetes mellitus; disease association; disease course; female; Geriatric Depression Scale; human; *intima; ischemic heart disease; major clinical study; male; prediction; prospective study; rating scale.

Other Index Terms

aged; *arterial wall thickness; *artery media; article; cardiovascular risk; *carotid artery; *cognitive defect; dementia; depression; diabetes mellitus; disease association; disease course; female;

For peer review only - http://bmjopen.bmj.com/site/about/guidelines.xhtml

Geriatric Depression Scale; human; *intima; ischemic heart disease; major clinical study; male; prediction; prospective study; rating scale.

Link to the Ovid Full Text or citation:
Click here for full text options

Link to the External Link Resolver:
SFX

297.

Subclinical vascular atherosclerosis related to cognitive impairment and its biomarkers in community elderly subjects.
Yang C.-Y., Lee I., Park H.-I., Park H.-Y., Song J.-E.
PM and R. Conference: 71st Annual Assembly of the American Academy of Physical Medicine and Rehabilitation. Seattle, WA United States. Conference Publication: (var.pagings). 2 (9 SUPPL. 1) (pp S192), 2010. Date of Publication: September 2010.
AN: 70477779
Objective: To evaluate the effect of common carotid artery atherosclerosis to the cognitive decline and cognition related biomarkers in older adult.
Design(s): Cross-sectional survey, controlled study.
Setting(s): Community health care course.
Participant(s): Seventy adult lived in community, aged 64-82 years, were included. They did not have diagnosed previously as cognitive impairment or dementia.
Intervention(s): We performed survey, cognitive function test, blood sample test, and carotid sonography. All subjects were divided into IMT-normal (CCA-IMT <1 mm, n=46), IMT-abnormal group (CCA-IMT >1mm, n=24).
Main Outcome Measure(s): We conducted life style survey (BMI, the history of smoking, alcohol, diabetes, hypertension, previous stroke, hyperlipidemia, vascular and cardiovascular disease, and regular exercise), the cognitive function by mini-mental state examination (K-MMSE), clinical dementia rating (CDR-K), Montreal cognitive assessment (MOCA-K), the cognition biomarkers by insulin-like growth factor-1, HDL, LDL, total cholesterol, triglyceride, high sensitivity C-reactive protein (hs-CRP), total homocysteine (t-HCY), glucose, insulin, HOMA insulin resistance index,

For peer review only - http://bmjopen.bmj.com/site/about/guidelines.xhtml

vitamin B12 and folate level, and the common carotid arteryintima media thickness (CCA-IMT) and plaques by carotid ultrasonography.

Result(s): There were significant difference between 2 groups in LDL, triglyceride, hs-CRP, abnormal t-HCY, age, sex, body weight and height, average IMT of both CCA, abnormal in IMT or plaque, IMT quartile, MOCA-K, K-MMSE, MMSE quartile ($P<.05$). The risk factors for abnormal IMT were LDL, average IMT of both CCA, IMT quartile, K-MMSE, MMSE quartile, age, and height as odds ratio more than 1 ($P<.05$). After the adjustment of age, the risk factors for abnormal IMT were LDL, average IMT of both CCA, IMT quartile, MOCA-K, K-MMSE, MMSE quartile, height, and weight as odds ratio more than 1 ($P<.05$).

Conclusion(s): The subclinical vascular atherosclerosis (IMT) and cognitive biomarkers maybe one of the important factor of cognitive decline.

Institution

(Yang, Lee, Park, Park, Song) Wonkwang Univ., School of Medicine, Iksan, South Korea

Publisher

Elsevier Inc.

Emtree Heading

*aged; *human; *atherosclerosis; *cognitive defect; *community; *rehabilitation; *physical medicine; Mini Mental State Examination; cognition; height; carotid artery; echography; adult; risk factor; risk; dementia; carotid atherosclerosis; controlled study; community care; blood sampling; lifestyle; smoking; diabetes mellitus; function test; stroke; hyperlipidemia; cardiovascular disease; exercise; cholesterol blood level; hypertension; insulin resistance; thickness; body weight; weight; common carotid artery; low density lipoprotein; triacylglycerol; alcohol; C reactive protein; somatomedin C; homocysteine; glucose; insulin; vitamin; cyanocobalamin; folic acid; high density lipoprotein.

Drug Index Terms

low density lipoprotein; triacylglycerol; alcohol; C reactive protein; somatomedin C; homocysteine; glucose; insulin; vitamin; cyanocobalamin; folic acid; high density lipoprotein.

Other Index Terms

*aged; *human; *atherosclerosis; *cognitive defect; *community; *rehabilitation; *physical medicine; mini mental state examination; cognition; height; carotid artery; echography; adult; risk factor; risk; dementia; carotid atherosclerosis; controlled study; community care; blood sampling; lifestyle; smoking; diabetes mellitus; function test; stroke; hyperlipidemia; cardiovascular disease; exercise; cholesterol blood level; hypertension; insulin resistance; thickness; body weight; weight; common carotid artery.

Link to the Ovid Full Text or citation:

For peer review only - http://bmjopen.bmj.com/site/about/guidelines.xhtml

Click here for full text options

Link to the External Link Resolver:
SFX

302.

Intima media thickness abnormality related to cognitive impairment and vascular biomarkers in the community-based elderly.
Yang C.-Y., Park H.-Y., Park H.-I., Song J.-E., Park S.-A.
International Journal of Stroke. Conference: World Stroke Congress 2010. Seoul South Korea. Conference Publication: (var.pagings). 5 (SUPPL. 2) (pp 374), 2010. Date of Publication: October 2010.
AN: 70335957
Objective: This study aimed to evaluate the effect of common carotid artery atherosclerosis to the cognitive impairment and vascularrelated biomarkers in elder people.
Method(s): Seventy physically healthy adult lived in community, aged 64-82 years, were included. All subjects were divided into IMT-normal (CCA-IMT< 1 mm, n = 46), IMT-abnormal group (CCAIMT>= 1 mm, n = 24). We conducted life style survey (body weight and height, the history of smoking, alcohol, diabetes, hypertension, previous stroke, hyperlipidemia, vascular and cardiovascular disease, and regular exercise), the cognitive function by mini-mental state examination (K-MMSE), clinical dementia rating (CDR-K), Montreal cognitive assessment (MOCA-K), biomarkers by insulin-like growth factor-1, HDL, LDL, total cholesterol, triglyceride, high sensitivity C-reactive protein (hs-CRP), total homocystein (t-HCY), glucose, insulin, HOMA insulin resistance index, vitamin B12 and folate level, and the common carotid artery-intima media thickness (CCA-IMT) and plaques by carotid ultrasonography.
Result(s): There were significant difference between two groups in LDL, triglyceride, hs-CRP, abnormal t-HCY, age, sex, body weight and height, average IMT of both CCA, abnormal in IMT or plaque, IMT quartile, MOCA-K, K-MMSE, MMSE quartile ($P < 0.05$). The risk factors for abnormal IMT were LDL, average IMT of both CCA, IMT quartile, K-MMSE, MMSE quartile, age, and height as odds ratio more than 1 ($P < 0.05$). After the adjustment of age, the risk factors for abnormal IMT were significant in LDL, average IMT of both CCA, IMT quartile, MOCA-K, K-MMSE, MMSE quartile, height and weight ($P < 0.05$).

For peer review only - http://bmjopen.bmj.com/site/about/guidelines.xhtml

Conclusion(s): The subclinical vascular atherosclerosis of IMT and vascular biomarkers maybe one of the important factors of cognitive impairment.

Institution
(Yang) Department of Physcial Medicine, Rehabilitation, Iksan, South Korea (Park) Department of Neurology, Wonkwang University, College of Medicine, Iksan, South Korea
(Park, Song) Department of Physical Medicine, Rehabilitation, Wonkwang University Hospital, Iksan, South Korea
(Park) Department of Nuclear Medicine, Wonkwang University, College of Medicine, Iksan, South Korea

Publisher
Blackwell Publishing Ltd

Emtree Heading
*cognitive defect; *arterial wall thickness; *community; *stroke; *aged; Mini Mental State Examination; height; risk factor; common carotid artery; body weight; carotid artery; echography; risk; weight; carotid atherosclerosis; normal human; lifestyle; smoking; diabetes mellitus; hypertension; hyperlipidemia; cardiovascular disease; exercise; cognition; dementia; cholesterol blood level; insulin resistance; artery intima; atherosclerosis; low density lipoprotein; triacylglycerol; alcohol; high density lipoprotein; C reactive protein; somatomedin C; glucose; insulin; vitamin; cyanocobalamin; folic acid.

Drug Index Terms
low density lipoprotein; triacylglycerol; alcohol; high density lipoprotein; C reactive protein; somatomedin C; glucose; insulin; vitamin; cyanocobalamin; folic acid.

Other Index Terms
*cognitive defect; *arterial wall thickness; *community; *stroke; *aged; mini mental state examination; height; risk factor; common carotid artery; body weight; carotid artery; echography; risk; weight; carotid atherosclerosis; normal human; lifestyle; smoking; diabetes mellitus; hypertension; hyperlipidemia; cardiovascular disease; exercise; cognition; dementia; cholesterol blood level; insulin resistance; artery intima; atherosclerosis.

Link to the Ovid Full Text or citation:
Click here for full text options

Link to the External Link Resolver:
SFX

For peer review only

303.

Correlation of cognitive functions and CIMT in young diabetics.

Srivastava P., Kumar M., Bhatia R., Singh M.B., Tripathi M., Prasad K., Tandon N.

International Journal of Stroke. Conference: World Stroke Congress 2010. Seoul South Korea. Conference Publication: (var.pagings). 5 (SUPPL. 2) (pp 373), 2010. Date of Publication: October 2010.

AN: 70335953

Introduction: Cognitive dysfunction in diabetes mellitus (DM), is correlated both with hyper and hypoglycemia, insulin resistance and concomitant vascular risk factors. The relation of atherogenesis with subclinical cognitive impairment has been evaluated in older adults but not in young asymptomatic patients.

Objective(s): Aim is to identify subclinical impairment in individual cognitive domains in young diabetics and correlate with carotid intimal media thickness (CIMT) identified as a surrogate marker for atherosclerosis.

Method(s): Young literate diabetics (18-45 years), from the diabetes clinic were administered Addenbrooke's cognitive examination (ACE) in English and Hindi followed by Carotid Doppler study on the same day. Past history of stroke or transient ischemic attacks (TIAs) were exclusion factors.

Result(s): Of 53 patients included, mean age was 35.7 years (M: 38; F: 15). Abnormal CIMT was taken as > 0.06 cm on either side. Abnormal ACE , Mini Mental Scale Examination (MMSE) scores were taken as < 82 and < 24 respectively. Two points less than normal in each cognitive domain was defined as abnormal. Increased right CIMT was found associated with visuo spatial, memory, MMSE and total ACE scores. Increased left CIMT correlated with attention, orientation, language, visuo spatial, memory , MMSE scores and total AC score in Hindi (P < 0.01).

Conclusion(s): The study demonstrated derangements in individual domains of cognition and high CIMT in young diabetics, suggesting accelerated atherosclerosis and chronic subclinical cerebral ischemia which may account for subclinical cognitive dysfunction.

Institution

(Srivastava, Kumar, Bhatia, Singh, Tripathi, Prasad) Neurology, All India Institute of Medical Sciences, New Delhi, India (Tandon) Endocrinology, All India Institute of Medical Sciences, New Delhi, India

Publisher

Blackwell Publishing Ltd

Emtree Heading

For peer review only

For peer review only - http://bmjopen.bmj.com/site/about/guidelines.xhtml

*diabetes mellitus; *cognition; *stroke; cognitive defect; spatial memory; patient; carotid artery; atherosclerosis; examination; Mini Mental State Examination; language; thickness; disease marker; brain ischemia; hospital; transient ischemic attack; hypoglycemia; insulin resistance; risk factor; atherogenesis; adult.

Other Index Terms

*diabetes mellitus; *cognition; *stroke; cognitive defect; spatial memory; patient; carotid artery; atherosclerosis; examination; mini mental state examination; language; thickness; disease marker; brain ischemia; hospital; transient ischemic attack; hypoglycemia; insulin resistance; risk factor; atherogenesis; adult.

Link to the Ovid Full Text or citation:

Click here for full text options

Link to the External Link Resolver:

SFX

306.

Relationship between plaque echolucency and cognitive function in patients with carotid atherosclerosis.

Desideri G., Mastroiacovo D., Pinelli M., Grassi D., Turco G.L., Camerota A., D'Andrea M., Andriulli M., Petrella I., Marini C., Lechiara M.C., Ghiadoni L., Bocale R., De Blasis G., Ferri C.

High Blood Pressure and Cardiovascular Prevention. Conference: National Congress of the Italian Society of Hypertension, SIIA 2010. Rome Italy. Conference Publication: (var.pagings). 17 (3) (pp 133), 2010. Date of Publication: 2010.

AN: 70271179

Dipartimento di Medicina Interna, Pisa, Italy Introduction:Numerous studies have demonstrated the existence of a relationship between echogenicity of carotid plaques and cerebral ischaemic events, both silent and clinically manifest. These, in turn, expose the patient to increased risk of developing cognitive impairment and dementia in geriatric age.

Aim(s): Based on this scientific evidence we decided to investigate the relationship between echogenicity of carotid plaques and cognitive performance in patients with carotid atheromatous plaques but no history of cerebrovascular events and/or clinical evidence for dementia.

For peer review only

Method(s):We studied 55 individuals aged 65 years ormore (73.3+/-5.1 years) relating to our angiology unit for atheroma of neck vessels. At enrolment, patients underwent ultrasonography of the neckvessels using a 7-8MHz linear probe; images were stories on magneto-optical disk and subsequently used to calculate the grey scale median (GSM) using Adobe Photoshop 5.0. The calculation of the GSMwas performed by researchers blinded to the clinical characteristics of patients.All participants were also subjected to the study of cognitive functions byMiniMental State Examination (MMSE), TrailMaking Test (TMT) A and B and verbal fluency test (VFT). The results of psychometric tests were logarithmically transformed and used to calculate a composite cognitive score.
Result(s): Patients were divided into two groups according echolucency of carotid plaques using as discriminating median GSM obtained in the study population (36.5+/-14.1 vs 52.3+/-16.2 respectively, p <0.01). Cognitive performance was on average worse in patients with more plaque echolucency (MMSE: 28.1+/-1.4 vs 28.3+/-1.3, p = ns; TMTA: 86.1+/-24.9 vs 69.7+/-36.6, p <0.04; TMTB: 220.1+/-81.9 vs 160.4+/-74.0, p <0004; VFT: 33.5+/-9.1 vs 40.0+/-10.2, p <0.01; z score: -0309+/-0496 vs 0236+/-0712, p <0.001). Considering the study population as a whole, we observed an inverse correlation between GSM and cognitive performance (r: -0.345, p <0.002).
Conclusion(s): The results of our study demonstrate the existence of an inverse relationship between echolucency of carotid plaques and cognitive function in the elderly and suggest the possible use of this method to identify subjects at increased risk of developing dementia.
Institution
(Desideri, Mastroiacovo, Grassi, Camerota, D'Andrea, Andriulli, Petrella, Marini, Bocale, Ferri) Universita degli Studi dell'Aquila, Dipartimento di Medicina Interna e Sanita Pubblica, L'Aquila, Italy
(Pinelli, Turco, De Blasis) Ospedale Civile di Avezzano, Avezzano, Italy
(Lechiara) Ospedale S. Rinaldi, Pescina, Italy
(Ghiadoni) Universita degli Studi di Pisa, Italy
Publisher
Adis International Ltd
Emtree Heading
*patient; *cognition; *hypertension; *society; *carotid atherosclerosis; carotid artery; dementia; population; risk; angiology; atheroma; neck; echography; optical disk; examination; psychometry; aged; Italy; cognitive defect; atherosclerotic plaque.
Other Index Terms
*patient; *cognition; *hypertension; *society; *carotid atherosclerosis; carotid artery; dementia; population; risk; angiology; atheroma; neck; echography; optical disk; examination; psychometry; aged; Italy; cognitive defect; atherosclerotic plaque.

For peer review only

Link to the Ovid Full Text or citation:

Click here for full text options

Link to the External Link Resolver:

SFX

309.

Peripheral arterial disease and cognition.

El-Kattan M.M., Zakaria Y.A., El-Fayomy N., Helmy S.M., Khairy H.M., Ramzy G.M., El-Jaafary S.I.

Egyptian Journal of Neurology, Psychiatry and Neurosurgery. 46 (2) (pp 311-322), 2009. Date of Publication: July 2009.

AN: 355219079

Background: Some investigators studied the relation between PVD and cognition and they found that, PVD patients performed more poorly than healthy subjects in some cognitive tests.

Objective(s): The aim of this study is to assess the relationship between PAD and cognitive dysfunction in patients without a known history of previous ischemic insult to the brain and to stress on the role of (ABI) as an accurate, simple, and non invasive measure of PAD and generalized atherosclerosis.

Method(s): Twenty patients with PAD and10 healthy normal control were included in the present study. All patients and control were subjected to: complete neurovascular examination, laboratory investigations, duplex arterial scan of the arteries of the lower extremities and of the carotid arteries, measurement of the ankle/brachial index (ABI), neuroimaging: M.R.I brain and cognitive assessment using tests for global and specific cognitive functions.

Result(s): In comparison to normal healthy subjects, patients with PAD and low ABI performed significantly worse in MMSE, and tests for attention, perceptuomotor speed, executive functions, short term memory, visual memory and visuoconstructive abilities. Moreover, subjects with PAD and low ABI had significant abnormal changes in neuroimaging and had significant increase in IMT and stenosis of the carotid artery.

Conclusion(s): (ABI) could be considered as an accurate, simple, and non invasive measure for diagnosis of PAD that associated with some cognitive impairment and generalized atherosclerosis.

Institution

(El-Kattan, Zakaria, El-Fayomy, Helmy, Ramzy, El-Jaafary) Department of Neurology, Cairo University, Cairo, Egypt (Khairy) Vascular Surgery, Cairo University, Cairo, Egypt

For peer review only

Publisher

Egyptian Society of Neurology, Psychiatry, and Neurosurgery (176 Tahrir St., Bab El-Louk, Cairo 11111, Egypt)

Emtree Heading

adult; aged; *ankle brachial index; *artery disease/di [Diagnosis]; artery occlusion; article; atherosclerosis/di [Diagnosis]; attention; carotid artery; clinical article; cognition; *cognitive defect; controlled study; depth perception; diagnostic accuracy; diagnostic value; disease association; echography; female; human; laboratory test; leg artery; male; neuroimaging; neurologic examination; non invasive measurement; nuclear magnetic resonance imaging; perception; short term memory; visual memory.

Other Index Terms

adult; aged; *ankle brachial index; *artery disease / *diagnosis; artery occlusion; article; atherosclerosis / diagnosis; attention; carotid artery; clinical article; cognition; *cognitive defect; controlled study; depth perception; diagnostic accuracy; diagnostic value; disease association; echography; female; human; laboratory test; leg artery; male; neuroimaging; neurologic examination; non invasive measurement; nuclear magnetic resonance imaging; perception; short term memory; visual memory.

Link to the Ovid Full Text or citation:

Click here for full text options

Link to the External Link Resolver:

SFX

315.

Subclinical atherosclerosis is weakly associated with lower cognitive function in healthy hyperhomocysteinemic adults without clinical cardiovascular disease.

Gatto N.M., Henderson V.W., St. John J.A., McCleary C., Detrano R., Hodis H.N., Mack W.J.

International Journal of Geriatric Psychiatry. 24 (4) (pp 390-399), 2009. Date of Publication: 2009.

AN: 354795210

For peer review only

Objective: Atherosclerosis is the most common pathologic process underlying cardiovascular disease (CVD). It is not well known whether subclinical atherosclerosis is an independent risk factor for lower cognitive function among individuals without clinically evident CVD.
Method(s): We examined cross-sectional associations between subclinical atherosclerosis and cognitive function in a community-based sample of otherwise healthy adults with plasma homocysteine >=8.5 mumol/L enrolled in the BVAIT study (n=504, mean age 61 years). Carotid artery intima-media thickness (CIMT), coronary artery calcium (CAC) and abdominal aortic calcium (AAC) were used to measure subclinical atherosclerosis. Cognitive function was assessed with a battery of neuropsychological tests. A principal components analysis was used to extract five uncorrelated cognitive factors from scores on individual tests, and a measure of global cognition was derived. Multivariable linear regression was used to examine the association between subclinical atherosclerosis and cognitive function, adjusting for other correlates of cognition.
Result(s): Increasing thickness of CIMT was associated with significantly lower scores on the verbal learning factor (beta=-0.07 per 0.1mm increase CIMT [SE(beta)=0.03], p=0.01). CAC and AAC were not individually associated with any of the cognitive factors.
Conclusion(s): This study provides evidence that increasing CIMT is weakly associated with lower verbal learning abilities but not global cognition in a population of otherwise healthy middle-to-older aged adults with elevated plasma homocysteine levels but without clinically evident CVD. The association between CIMT and poor verbal learning may pertain particularly to men. Copyright © 2008 John Wiley & Sons, Ltd.
PMID
18836986 [http://www.ncbi.nlm.nih.gov/pubmed/?term=18836986]
Institution
(Gatto, St. John, Hodis, Mack) Department of Preventive Medicine, USC Keck School of Medicine, 1540 Alcazar Street, Los Angeles, CA 90089-9010, United States (Henderson) Stanford University, Department of Health Research and Policy, Department of Neurology and Neurological Sciences, Stanford, CA, United States
(St. John, Hodis, Mack) Atherosclerosis Research Unit, Department of Medicine, USC Keck School of Medicine, Los Angeles, CA, United States
(McCleary) Department of Neurology, USC Keck School of Medicine, Los Angeles, CA, United States
(Detrano) UC Irvine, Department of Radiological Sciences, Irvine, CA, United States
Publisher
John Wiley and Sons Ltd (Southern Gate, Chichester, West Sussex PO19 8SQ, United Kingdom)
Emtree Heading
adult; aged; arterial wall thickness; article; *atherosclerosis; brain function; cholesterol blood level; *cognitive defect; community sample; diastolic blood pressure; disease association; female;

functional assessment; human; *hyperhomocysteinemia; learning disorder; major clinical study; male; neuropsychological test; protein blood level; sex difference; systolic blood pressure; triacylglycerol blood level; calcium/ec [Endogenous Compound]; high density lipoprotein cholesterol/ec [Endogenous Compound]; homocysteine/ec [Endogenous Compound]; low density lipoprotein cholesterol/ec [Endogenous Compound]; triacylglycerol/ec [Endogenous Compound].

Drug Index Terms

calcium / endogenous compound; high density lipoprotein cholesterol / endogenous compound; homocysteine / endogenous compound; low density lipoprotein cholesterol / endogenous compound; triacylglycerol / endogenous compound.

Other Index Terms

adult; aged; arterial wall thickness; article; *atherosclerosis; brain function; cholesterol blood level; *cognitive defect; community sample; diastolic blood pressure; disease association; female; functional assessment; human; *hyperhomocysteinemia; learning disorder; major clinical study; male; neuropsychological test; protein blood level; sex difference; systolic blood pressure; triacylglycerol blood level.

Link to the Ovid Full Text or citation:

Click here for full text options

Link to the External Link Resolver:

SFX

For peer review only

317.

Carotid atherosclerosis and cognitive decline in patients with Alzheimer's disease.

Silvestrini M., Gobbi B., Pasqualetti P., Bartolini M., Baruffaldi R., Lanciotti C., Cerqua R., Altamura C., Provinciali L., Vernieri F.

Neurobiology of Aging. 30 (8) (pp 1177-1183), 2009. Date of Publication: August 2009.

AN: 50013216

Aim of the study was to explore the correlation between the progression of carotid atherosclerosis and the evolution of cognitive impairment in 66 patients with Alzheimer's disease (AD). They underwent cognitive status evaluation and ultrasonography (US) to investigate carotid arteries intima-media thickness (IMT) and plaque index (PI). After a 12-month follow-up period,

For peer review only - http://bmjopen.bmj.com/site/about/guidelines.xhtml

neuropsychological and US examinations were repeated to assess the progression of carotid atherosclerosis and of cognitive decline [in terms of changes in Mini Mental State Examination (MMSE) scores]. MMSE score changes were related to baseline IMT ($p = 0.018$), changes in IMT ($p < 0.001$) and PI ($p = 0.006$), and "antihypertensive drug intake" ($p < 0.001$). While the first three variables correlated with increased cognitive impairment, the last one was associated with a reduced extent of MMSE score decline. Results show a link between progression of carotid wall changes and of cognitive decline, and suggest a possible protective role of antihypertensive therapy. Given the potential clinical implications, our preliminary findings could stimulate further investigations into the role of vascular impairment in patients with AD. © 2007 Elsevier Inc. All rights reserved.

PMID

18077061 [http://www.ncbi.nlm.nih.gov/pubmed/?term=18077061]

Institution

(Silvestrini, Gobbi, Bartolini, Baruffaldi, Lanciotti, Cerqua, Provinciali) Neurological Clinic, Polytechnic University of Marche, Via Conca 1, 60020 Ancona, Italy (Pasqualetti) Center of Medical Statistics and Information Technology, AFaR-Fatebenefratelli Hospital-Isola Tiberina, Lungotevere degli Anguillara 12, 00153 Rome, Italy

(Altamura, Vernieri) Neurological Clinic, Campus Biomedico University, Via Dei Compositori 130, 00128 Rome, Italy

Publisher

Elsevier Inc. (360 Park Avenue South, New York NY 10010, United States)

Emtree Heading

aged; Alzheimer disease/dt [Drug Therapy]; *Alzheimer disease/dt [Drug Therapy]; arterial wall thickness; article; atherosclerotic plaque; *cerebrovascular disease/di [Diagnosis]; *cognitive defect/di [Diagnosis]; disease association; disease course; echography; female; follow up; human; hypertension/dt [Drug Therapy]; major clinical study; male; Mini Mental State Examination; neuroprotection; neuropsychological test; priority journal; antihypertensive agent/dt [Drug Therapy]; donepezil/dt [Drug Therapy]; *carotid atherosclerosis/di [Diagnosis].

Candidate Terms

*carotid atherosclerosis / *diagnosis [other term].

Drug Index Terms

antihypertensive agent / drug therapy; donepezil / drug therapy.

Other Index Terms

aged; Alzheimer disease / drug therapy; *Alzheimer disease / *drug therapy; arterial wall thickness; article; atherosclerotic plaque; *cerebrovascular disease / *diagnosis; *cognitive defect / *diagnosis; disease association; disease course; echography; female; follow up; human; hypertension / drug

For peer review only

therapy; major clinical study; male; mini mental state examination; neuroprotection; neuropsychological test; priority journal.

Link to the Ovid Full Text or citation:
Click here for full text options

Link to the External Link Resolver:
SFX

322.

Intracranial atherosclerosis as a contributing factor to Alzheimer's disease dementia.
Kokjohn T.A., Maarouf C.L., Sabbagh M.N., Belohlavek M., Garami Z., Beach T.G., Roher A.E.
Alzheimer's and Dementia. Conference: Alzheimer's Association International Conference on Alzheimer's Disease. Vienna Austria. Conference Publication: (var.pagings). 5 (4 SUPPL. 1) (pp 293), 2009. Date of Publication: July 2009.
AN: 70109345
Background: Cardiovascular diseases are significant Alzheimer's disease (AD) risk factors. Atherosclerosis of carotid and intracranial arteries results in decreased perfusion pressure and consequent hypoxiA/ischemia, metabolic distress, neuronal dysfunction and dementia. Phase contrast magnetic resonance angiography and Duplex ultrasound of cervical arteries have demonstrated significant differences in cerebral blood flow between AD and non-demented (ND) individuals.
Method(s): We quantified the degree of occlusion in the circle of Willis arteries collected from 102 subjects: 59 AD, 36 ND and 7 non-AD dementia (NADD) individuals. The external and internal (lumenal) surfaces were electronically measured on arterial cross-sections (~ 0.5 cm) to obtain an index of stenosis and evaluate the extent of atherosclerotic occlusion.
Result(s): Arteries of the circle of Willis were more severely occluded by atherosclerotic lesions in AD than in the ND and NADD groups. In the AD cohort, 21% of arterial sections were 60-69% occluded, 15% were 70-79%, 9.3% were 80-89% 3.9% were 90-99% with 1.3% of arteries exhibiting complete (100%) occlusion. By contrast, for the identical decile degree of stenosis, the corresponding percentages were: 15%, 7.9%, 2.2, 1.2% and 0.5% in the ND group. The NADD arterial stenoses were uniformly lower than those of the ND cohort. A comparison between

For peer review only

individual major intracranial arteries demonstrated significant differences between the AD and ND cohorts being the former group more affected than the latter: vertebral arteries, p=0.028; basilar artery, p= 0.004; posterior cerebral artery, p=0.002; middle cerebral artery, p=0.0005; anterior cerebral artery, p=0.037 and internal carotid artery, p=0.003.

Conclusion(s): Atherosclerosis of intracranial arteries is more severe and involves a larger number of arterial sites in AD patients than in ND and NADD. The data is consistent with the hypothesis that combined functional and anatomical alterations of the cardiovascular system attenuate cerebral blood flow and contribute to neuronal damage and dementia.

Institution

(Kokjohn) Midwestern University, Sun Health Research Institute, Glendale and Sun City, AZ, United States (Maarouf, Sabbagh, Beach, Roher) Sun Health Research Institute, Sun City, AZ, United States

(Belohlavek) Mayo Clinic, Scottsdale, AZ, United States

(Garami) Methodist Hospital, Houston, TX, United States

Publisher

Elsevier Inc.

Emtree Heading

*Alzheimer disease; *atherosclerosis; *dementia; artery; occlusion; brain circulus arteriosus; stenosis; brain blood flow; cardiovascular disease; risk factor; carotid artery; perfusion pressure; magnetic resonance angiography; ultrasound; basilar artery; posterior cerebral artery; middle cerebral artery; anterior cerebral artery; vertebral artery; internal carotid artery; patient; hypothesis; cardiovascular system; peripheral occlusive artery disease.

Other Index Terms

*Alzheimer disease; *atherosclerosis; *dementia; artery; occlusion; brain circulus arteriosus; stenosis; brain blood flow; cardiovascular disease; risk factor; carotid artery; perfusion pressure; magnetic resonance angiography; ultrasound; basilar artery; posterior cerebral artery; middle cerebral artery; anterior cerebral artery; vertebral artery; internal carotid artery; patient; hypothesis; cardiovascular system; peripheral occlusive artery disease.

Link to the Ovid Full Text or citation:

Click here for full text options

Link to the External Link Resolver:

SFX

For peer review only - http://bmjopen.bmj.com/site/about/guidelines.xhtml

325.

Vascular ultrasound parameters and cognitive performance in patients with dementia.

Martinic Popovic I., Jurasic M.J., Seric V., Trkanjec Z., Morovic S., Demarin V.

Journal of the Neurological Sciences. Conference: 5th International Congress on Vascular Dementia. Budapest Hungary. Conference Publication: (var.pagings). 283 (1-2) (pp 314), 2009. Date of Publication: 15 Aug 2009.

AN: 70102355

Background and aim: Non-invasive neurosonology methods are known to be a convenient tool in the assessment of vascular changes of both extracranial and basal cerebral arteries. Vascular pathology is common characteristics of both vascular dementia (VAD) and Alzheimer's disease (AD), so these methods are increasing in importance in order to early recognize the patients at possible risk of clinically evident dementia. The aim of this study was to compare vascular ultrasound parameters and cognitive decline in patients with VAD and AD.

Patients and Methods: The study included 16 patients diagnosed with AD (8 men and 8 women, mean age72.19+/-6.86 years) and 22 patients with VAD (14 women and 8 men, mean age 70.20+/-15.50 years) with similar scores on MMSE (mean 19.95+/-5.01 points).Ultrasound measurements on common carotid artery (CCA)were performed usingAloka ProSoundALPHA 10with 13MHz linear probe.

Result(s): Both groups did not differ significantly in mean MMSE scores (20.38+/-4.73 points for patients with AD; 19.80+/-5.19 points for patients with VAD). We found no statistically significant differences among both groups regarding body mass index, blood and pulse pressure, intima media thickness, CCA diameter and beta stiffness index. However, regression analysis for both groups proved that MMSE corresponds to vascular diameter change and beta stiffness index increase ($p<0.01$).

Conclusion(s): Positive correlation between MMSE results and vascular parameters, such as vascular diameter increase and beta stiffness increase indicate that non-invasivemonitoring vascular changeswith ultrasoundmay be useful in follow up of demented patients. The same method would probably be useful in early identification of subjects at the presymptomatic stage of cognitive decline.

Institution

(Martinic Popovic, Jurasic, Seric, Trkanjec, Morovic, Demarin) Clinical Department of Neurology, Zagreb, Croatia

Publisher

Elsevier

For peer review only - http://bmjopen.bmj.com/site/about/guidelines.xhtml

Emtree Heading
*patient; *multiinfarct dementia; *ultrasound; *dementia; Mini Mental State Examination; rigidity; female; blood vessel diameter; vascular disease; body mass; common carotid artery; blood; pulse pressure; arterial wall thickness; brain artery; regression analysis; follow up; risk; Alzheimer disease.
Other Index Terms
*patient; *multiinfarct dementia; *ultrasound; *dementia; mini mental state examination; rigidity; female; blood vessel diameter; vascular disease; body mass; common carotid artery; blood; pulse pressure; arterial wall thickness; brain artery; regression analysis; follow up; risk; Alzheimer disease.

Link to the Ovid Full Text or citation:
Click here for full text options

Link to the External Link Resolver:
SFX

328.

Signs of subclinical vascular disease and amnestic form of mild cognitive impairment.
Adam I., Csanyi A.F.
Journal of the Neurological Sciences. Conference: 5th International Congress on Vascular Dementia. Budapest Hungary. Conference Publication: (var.pagings). 283 (1-2) (pp 293), 2009. Date of Publication: 15 Aug 2009.
AN: 70102278
Background and aims: Mild cognitive impairment (MCI) is regarded as a precursor to dementia, but not all patients with MCI develop dementia. Current diagnostic criteria for MCI define heterogeneous populations, but signs of subclinical vascular disease may be of help in predicting a conversion to dementia. In a preliminary study we investigated the association of early signs of atherosclerosis with amnestic MCI by various noninvasive measures.
Method(s): Performances onADAS-Cog test andMini-Mental State Examination (MMSE) were compared in 26 subjects (70.6+/-6.5 years) with ultrasoundassessed common carotid intima-media thickness (IMT), index of augmentation (AIX) and aortic pulse wave velocity (PWV). Mayo Clinic criteria were used for evaluation of amnestic form of MCI. Subjects with a previous history of strokewere excluded. The values of Hamilton Rating Scale for depression were less than 12.

For peer review only

Result(s): No associations were found for cognitive values (ADAS-cog and MMSE) and IMT, AIX, PWV.

Conclusion(s): The amnestic subtype of MCI has putatively a degenerative etiology. Studies have shown that these people overwhelmingly progressed to AD. Further studies are needed to assess the association between the amnestic and other types (multiple domain and single non-memory domain) of MCI and early vascular risk factors.

Institution

(Adam, Csanyi) Department of Neurology, Petz Hospital, Gyor, Hungary (Csanyi) Lajos Petz Department, Istvan Szechenyi University, Gyor, Hungary

Publisher

Elsevier

Emtree Heading

*vascular disease; *mild cognitive impairment; *multiinfarct dementia; dementia; arterial wall thickness; pulse wave; hospital; Hamilton scale; etiology; memory; risk factor; precursor; patient; diagnosis; Mini Mental State Examination; atherosclerosis; mental health; examination; carotid artery; population.

Other Index Terms

*vascular disease; *mild cognitive impairment; *multiinfarct dementia; dementia; arterial wall thickness; pulse wave; hospital; Hamilton scale; etiology; memory; risk factor; precursor; patient; diagnosis; mini mental state examination; atherosclerosis; mental health; examination; carotid artery; population.

Link to the Ovid Full Text or citation:

Click here for full text options

Link to the External Link Resolver:

SFX

329.

Carotid stenosis and cognitive function.

Sztriha L.K.

For peer review only - http://bmjopen.bmj.com/site/about/guidelines.xhtml

Journal of the Neurological Sciences. Conference: 5th International Congress on Vascular Dementia. Budapest Hungary. Conference Publication: (var.pagings). 283 (1-2) (pp 278), 2009. Date of Publication: 15 Aug 2009.

AN: 70102224

While stroke is a known cause of cognitive impairment, the relationship between carotid artery stenosis and cognitive function in people without a history of stroke is less clear. A number of risk factors for vascular disease are related to cognitive impairment. Hypertension, diabetes mellitus, cigarette smoking, and dyslipidemia are associated with an increased risk of carotid artery disease too. Some studies have suggested that stenosis of the internal carotid artery may be an independent risk factor for cognitive impairment. Cognitive impairment and decline tend to be more frequent with left-sided stenosis, if tests for dominant hemispheric function in right-handed individuals are utilized during the assessment. The possible pathomechanisms of cognitive impairment include silent embolization and hypoperfusion. High-grade stenosis of the internal carotid artery may be associated with cognitive impairment even without evidence of infarction on magnetic resonance imaging. On the other hand, it is fairly common that patients have normal cognition despite severe carotid artery disease, highlighting the important role of an efficient collateral blood supply. The carotid intimamedia thickness appears to be a marker of underlying risk factors and generalized atherosclerosis, rather than a direct cause of cognitive impairment. Carotid endarterectomy or stenting may lead to a decline in cognitive function due to microembolic ischemia or intraprocedural hypoperfusion. Conversely, restoring perfusion could improve cognitive dysfunction that might have occurred from a state of chronic hypoperfusion. It is unclear whether these complex interactions ultimately result in a net improvement or a deterioration of cognitive function. Evidence at present does not seem strong enough to include loss of cognition as a factor in determining the balance of risk and benefit from therapy for asymptomatic carotid stenosis.

Institution

(Sztriha) Department of Neurology, University of Szeged, Szeged, Hungary

Publisher

Elsevier

Emtree Heading

*cognition; *carotid artery obstruction; *multiinfarct dementia; cognitive defect; perfusion; risk factor; stenosis; risk; stroke; carotid artery disease; internal carotid artery; carotid endarterectomy; stent; ischemia; deterioration; therapy; diabetes mellitus; dyslipidemia; hypertension; artificial embolism; vascular disease; infarction; nuclear magnetic resonance imaging; patient; collateral circulation; carotid artery; arterial wall thickness; atherosclerosis; cigarette smoking; marker.

Drug Index Terms

marker.

For peer review only

Other Index Terms

*cognition; *carotid artery obstruction; *multiinfarct dementia; cognitive defect; perfusion; risk factor; stenosis; risk; stroke; carotid artery disease; internal carotid artery; carotid endarterectomy; stent; ischemia; deterioration; therapy; diabetes mellitus; dyslipidemia; hypertension; artificial embolism; vascular disease; infarction; nuclear magnetic resonance imaging; patient; collateral circulation; carotid artery; arterial wall thickness; atherosclerosis; cigarette smoking.

Link to the Ovid Full Text or citation:

[Click here for full text options]

Link to the External Link Resolver:

[SFX]

341.

Socioeconomic status moderates the association between carotid intima-media thickness and cognition in midlife: Evidence from the Whitehall II study.

Singh-Manoux A., Britton A., Kivimaki M., Gueguen A., Halcox J., Marmot M.

Atherosclerosis. 197 (2) (pp 541-548), 2008. Date of Publication: April 2008.

AN: 351444793

Background: Common carotid artery intima-media thickness (IMT) is a measure of generalized atherosclerosis and has been shown to be associated with cognitive function. We examine two questions: does socioeconomic status (SES) moderate this association and is IMT more strongly associated with specific aspects of cognitive function? Methods: Data are drawn from the Phase 7 (2003-2004) of the Whitehall II study (N = 3896). In cross-sectional analyses the association between IMT and six measures of cognition (short-term verbal memory, inductive reasoning, vocabulary, semantic and phonemic fluency and a measure of global cognitive status) was examined in analyses adjusted for previous history of coronary heart disease, health behaviours and other vascular risk measures such as blood pressure, cholesterol and body mass index. Result(s): The overall association between IMT and the six measures of cognition was restricted to the low SES group (p = 0.02). Within this group, IMT was significantly associated with inductive reasoning (p = 0.001), vocabulary (p = 0.002), phonemic (p = 0.006) and semantic fluency (p = 0.02). The covariates examined explained about a quarter of the association between IMT and

For peer review only - http://bmjopen.bmj.com/site/about/guidelines.xhtml

cognition in the low SES group. The associations with the measure of inductive reasoning (p = 0.02), vocabulary (p = 0.02) and phonemic fluency (p = 0.04) remained after adjustment for all covariates.

Conclusion(s): SES is an important modifier of the association between IMT and cognition, an inverse association between the two was observed only in the low SES group. It is possible that high cognitive reserve among the high SES individuals prevents the functional manifestations of atherosclerosis. Verbal memory was not one of the cognitive domains associated with IMT. © 2007 Elsevier Ireland Ltd. All rights reserved.

PMID

17854813 [http://www.ncbi.nlm.nih.gov/pubmed/?term=17854813]

Institution

(Singh-Manoux, Gueguen) INSERM, U687, IFR69, HNSM, 14 rue du Val d'Osne, 94415 Saint-Maurice Cedex, France (Singh-Manoux, Britton, Kivimaki, Marmot) Department of Epidemiology and Public Health, University College London, United Kingdom

(Singh-Manoux) Centre de Gerontologie, Hopital Ste Perine, AP-HP, France

(Halcox) Vascular Physiology Unit, Department of Cardiology, Institute of Child Health, United Kingdom

Publisher

Elsevier Ireland Ltd (P.O. Box 85, Limerick, Ireland)

Emtree Heading

adult; anamnesis; artery intima; artery media; article; *atherosclerosis; blood pressure; body mass; cardiovascular risk; *carotid artery; cholesterol blood level; *cognition; disease association; female; health behavior; human; ischemic heart disease; major clinical study; male; priority journal; semantics; socioeconomics; verbal memory; cholesterol/ec [Endogenous Compound].

Drug Index Terms

cholesterol / endogenous compound.

Other Index Terms

adult; anamnesis; artery intima; artery media; article; *atherosclerosis; blood pressure; body mass; cardiovascular risk; *carotid artery; cholesterol blood level; *cognition; disease association; female; health behavior; human; ischemic heart disease; major clinical study; male; priority journal; semantics; socioeconomics; verbal memory.

Link to the Ovid Full Text or citation:

Click here for full text options

Link to the External Link Resolver:

For peer review only - http://bmjopen.bmj.com/site/about/guidelines.xhtml

SFX

345.

Cardiovascular disease and cognitive performance in middle-aged and elderly men.

Muller M., Grobbee D.E., Aleman A., Bots M., van der Schouw Y.T.

Atherosclerosis. 190 (1) (pp 143-149), 2007. Date of Publication: January 2007.

AN: 44908308

Background: Decline of cognitive function with age may be due, in part, to atherosclerotic changes. The aim of the present study was to determine the relative contribution of cardiovascular disease (CVD) to cognitive functioning in middle-aged and elderly men.

Method(s): In a cross-sectional study, cognitive tests were administered to 400 independently living men aged 40-80 years. Compound scores were calculated for memory function, processing capacity/speed, and executive function. The MMSE was used as a measure of global cognitive function. Carotid intima-media thickness, pulse wave velocity and ankle brachial blood pressure index were assessed as measures of sub-clinical CVD. The adjusted association of sub-clinical and prevalent CVD with neuropsychological test scores in the total group and in subgroups was assessed by linear regression analysis.

Result(s): Increased IMT was associated with lower scores on memory performance, and increased PWV was associated with lower scores on processing capacity and executive functioning. Compared with subjects with no CVD, both sub-clinical and prevalent cardiovascular diseases were related to a lower memory performance, beta's (95% CI) were -0.45 (-0.83, -0.07) and -0.45 (-0.84, 0.01), respectively. These associations were present in both middle-aged and elderly men. Furthermore, we observed that for subjects who had sub-clinical or prevalent cardiovascular disease the distribution of MMSE-scores was shifted toward lower values; the distributions were statistically different (p = 0.003).

Conclusion(s): The results of this study support a relation of sub-clinical CVD with cognitive functioning in middle-aged and elderly men. These results suggest that actions to prevent cognitive decline by preventing atherosclerosis should be taken before middle age. © 2006 Elsevier Ireland Ltd. All rights reserved.

PMID

16488420 [http://www.ncbi.nlm.nih.gov/pubmed/?term=16488420]

Institution

(Muller, Grobbee, Bots, van der Schouw) Julius Center for Health Sciences and Primary Care, University Medical Center Utrecht, Netherlands (Muller) Department of Geriatrics, University Medical Center Utrecht, Netherlands
(Aleman) BCN NeuroImaging Center, University of Groningen, Groningen, Netherlands
Publisher
Elsevier Ireland Ltd (P.O. Box 85, Limerick, Ireland)
Emtree Heading
adult; aged; *aging; ankle brachial index; article; *cardiovascular disease; carotid artery obstruction; *cognition; controlled study; correlation analysis; human; major clinical study; male; memory; Mini Mental State Examination; neuropsychological test; priority journal; pulse wave.
Other Index Terms
adult; aged; *aging; ankle brachial index; article; *cardiovascular disease; carotid artery obstruction; *cognition; controlled study; correlation analysis; human; major clinical study; male; memory; mini mental state examination; neuropsychological test; priority journal; pulse wave.

Link to the Ovid Full Text or citation:
Click here for full text options

Link to the External Link Resolver:
SFX

347.

Carotid intima-media thickness and cognitive function in elderly women: A population-based study.
Komulainen P., Kivipelto M., Lakka T.A., Hassinen M., Helkala E.-L., Patja K., Nissinen A., Rauramaa R.
Neuroepidemiology. 28 (4) (pp 207-213), 2007. Date of Publication: October 2007.
AN: 47573715
Objective: Several vascular risk factors have been linked to cognitive decline. However, little is known about the association between the atherosclerotic process and cognitive impairment. We investigated whether carotid intima-media thickness (IMT) predicts the risk of cognitive impairment and whether the putative impairment is specific for some cognitive domains.

Method(s): A 12-year population-based follow-up study was performed for a total of 91 women, aged 60-70 years at baseline. Ultrasonographically assessed carotid artery IMT and the Mini-Mental State Examination test were performed at baseline and 12-year follow-up. A detailed cognitive evaluation for memory and cognitive speed was performed in 2003. The mean of left and right carotid bifurcation IMT was used in the analyses for association with the risk for poor cognitive speed and memory.

Result(s): Increased IMT at baseline was an independent predictor for poor memory (beta = -5.004, 95% confidence interval = -7.74 to -2.27; p = 0.001) and cognitive speed (beta = 2.562, 95% confidence interval = 1.19-4.94; p = 0.035) at 12-year follow-up after adjustment for age, education, depression, plasma LDL cholesterol, systolic blood pressure, cardiovascular disease, hormone replacement therapy, smoking, alcohol consumption and physical activity. The risk for poor memory (p = 0.023 for linear trend) and cognitive speed (p = 0.070 for linear trend) increased with increasing IMT tertiles.

Conclusion(s): Carotid IMT predicts an increased risk for cognitive impairment, particularly poor memory and cognitive speed, in elderly women. Copyright © 2007 S. Karger AG.

PMID

17851259 [http://www.ncbi.nlm.nih.gov/pubmed/?term=17851259]

Institution

(Komulainen, Lakka, Hassinen, Rauramaa) Kuopio Research Institute of Exercise Medicine, University of Kuopio, Kuopio, Finland (Kivipelto) Department of Neuroscience and Neurology, University of Kuopio, Kuopio, Finland

(Helkala) Department of Public Health and General Practice, University of Kuopio, Kuopio, Finland

(Lakka) Department of Physiology, Institute of Biomedicine, University of Kuopio, Kuopio, Finland

(Rauramaa) Department of Clinical Physiology and Nuclear Medicine, Kuopio University Hospital, Kuopio, Finland

(Patja, Nissinen) Department of Epidemiology and Health Promotion, National Public Health Institute, Helsinki, Finland

(Kivipelto) Aging Research Center, Karolinska Institutet, Stockholm, Sweden

(Komulainen) Kuopio Research Institute of Exercise Medicine, Haapaniementie 16, FIN-70100 Kuopio, Finland

Publisher

S. Karger AG (Allschwilerstrasse 10, P.O. Box, Basel CH-4009, Switzerland)

Emtree Heading

adult; aged; alcohol consumption; *artery intima proliferation; *artery media; article; *atherosclerosis; cardiovascular disease; *carotid artery; carotid artery bifurcation; cholesterol blood level; *cognition; cognitive defect; depression; female; hormone substitution; human; major

For peer review only - http://bmjopen.bmj.com/site/about/guidelines.xhtml

clinical study; memory; Mini Mental State Examination; physical activity; smoking; systolic blood pressure.

Other Index Terms

adult; aged; alcohol consumption; *artery intima proliferation; *artery media; article; *atherosclerosis; cardiovascular disease; *carotid artery; carotid artery bifurcation; cholesterol blood level; *cognition; cognitive defect; depression; female; hormone substitution; human; major clinical study; memory; mini mental state examination; physical activity; smoking; systolic blood pressure.

Link to the Ovid Full Text or citation:

Click here for full text options

Link to the External Link Resolver:

SFX

349.

Carotid artery intima-media thickness and cognition in cardiovascular disease.

Haley A.P., Forman D.E., Poppas A., Hoth K.F., Gunstad J., Jefferson A.L., Paul R.H., Ler A.S.H., Sweet L.H., Cohen R.A.

International Journal of Cardiology. 121 (2) (pp 148-154), 2007. Date of Publication: 01 Oct 2007.

AN: 47385831

Background: Increased carotid artery intima-media thickness (IMT) is a non-invasive marker of systemic arterial disease. Increased IMT has been associated with atherosclerosis, abnormal arterial mechanics, myocardial infarction, and stroke. Given evidence of a relationship between cardiovascular health and attention-executive-psychomotor functioning, the purpose of this study was to examine IMT in relation to neuropsychological test performance in patients with a variety of cardiovascular diagnoses.

Method(s): One hundred and nine participants, ages 55 to 85, underwent neuropsychological assessment and B-mode ultrasound of the left common carotid artery. IMT was calculated using an automated algorithm based on a validated edge-detection technique. The relationship between IMT and measures of language, memory, visual-spatial abilities and attention-executive-psychomotor functioning was modeled using hierarchical linear regression analyses adjusted for

age, education, sex, cardiovascular risk, current systolic blood pressure, and history of coronary artery disease (CAD).

Result(s): Increased IMT was associated with significantly lower performance in the attention-executive-psychomotor domain (IMT beta = - 0.26, $p < .01$), independent of age, education, sex, cardiovascular risk, current systolic blood pressure, and CAD ($F(10,100) = 3.61$, $p < .001$). IMT was not significantly related to language, memory, or visual-spatial abilities.

Conclusion(s): Our findings suggest that, in patients with cardiovascular disease, IMT may be associated with the integrity of frontal subcortical networks responsible for attention-executive-psychomotor performance. Future studies are needed to clarify the mechanisms by which IMT affects cognition and examine potential interactions between increased IMT and other measures of cardiovascular health such as blood pressure variability, cardiac systolic performance, and systemic perfusion. © 2006 Elsevier Ireland Ltd. All rights reserved.

PMID

17196687 [http://www.ncbi.nlm.nih.gov/pubmed/?term=17196687]

Institution

(Haley, Hoth, Sweet, Cohen) Department of Psychiatry and Human Behavior, Brown Medical School, Providence, RI, United States (Forman) Division of Cardiology, Brigham and Women's Hospital, Geriatric Research, Education, and Clinical Care, Boston, MA, United States

(Poppas) Department of Cardiology, Rhode Island Medical Center, Providence, RI, United States

(Gunstad) Department of Psychology, Kent State University, Kent, OH, United States

(Jefferson) Alzheimer's Disease Center, Department of Neurology, Boston University School of Medicine, Boston, MA, United States

(Paul) Department of Behavioral Neuroscience, University of Missouri, St. Louis, MO, United States

(Ler) Department of Cognitive and Neural Systems, Boston University, Boston, MA, United States

Publisher

Elsevier Ireland Ltd (P.O. Box 85, Limerick, Ireland)

Emtree Heading

adult; aged; algorithm; article; attention; B scan; blood pressure variability; *blood vessel parameters; *cardiovascular disease; cardiovascular risk; *carotid artery; *cognition; common carotid artery; coronary artery disease; education; female; frontal cortex; human; language; linear regression analysis; major clinical study; male; memory; neuropsychological test; priority journal; psychomotor activity; sex difference; systolic blood pressure; task performance; *intima media thickness.

Candidate Terms

*intima media thickness [other term].

Other Index Terms

For peer review only

adult; aged; algorithm; article; attention; B scan; blood pressure variability; *blood vessel parameters; *cardiovascular disease; cardiovascular risk; *carotid artery; *cognition; common carotid artery; coronary artery disease; education; female; frontal cortex; human; language; linear regression analysis; major clinical study; male; memory; neuropsychological test; priority journal; psychomotor activity; sex difference; systolic blood pressure; task performance.

Link to the Ovid Full Text or citation:
Click here for full text options

Link to the External Link Resolver:
SFX

354.

Atherosclerosis and risk for dementia.
Van Oijen M., De Jong F.J., Witteman J.C.M., Hofman A., Koudstaal P.J., Breteler M.M.B.
Annals of Neurology. 61 (5) (pp 403-410), 2007. Date of Publication: May 2007.
AN: 46878768
Objective: Atherosclerosis has been implicated in the development of dementia and its major subtypes, Alzheimer's disease and vascular dementia. However, support for this association mainly comes from cross-sectional studies. We investigated the association of atherosclerosis with dementia and subtypes of dementia during long follow-up, with various noninvasive measures of atherosclerosis.
Method(s): This study was based on 6,647 participants in the Rotterdam Study, a population-based prospective cohort study among 7,983 elderly subjects. At baseline (1990-1993) and at the third survey (1997-1999), common carotid intima media thickness, carotid plaques, and peripheral arterial disease (measured as ankle-brachial index) were measured. During follow-up (mean, 9.0 years), 678 subjects developed dementia. We estimated the associations of different measures of atherosclerosis with risk for dementia and subtypes of dementia by means of Cox proportional hazard models. Analyses were repeated and stratified on duration of follow-up. To evaluate competing risk for mortality, we examined the association between measures of atherosclerosis and risk for dementia or mortality by combining the two in a single outcome measure.

For peer review only - http://bmjopen.bmj.com/site/about/guidelines.xhtml

Result(s): We found that atherosclerosis, predominantly carotid atherosclerosis, was associated with an increased risk for dementia during short follow-up. This association attenuated with longer follow-up, likely because of the strong association between atherosclerosis and mortality. The associations did not differ across apolipoprotein E genotypes.
Interpretation(s): Our findings suggest that atherosclerosis is associated with an increased risk for dementia. Stronger associations between atherosclerosis and mortality may attenuate the association between atherosclerosis and dementia in prospective cohort studies with long follow-up periods.

PMID

17328068 [http://www.ncbi.nlm.nih.gov/pubmed/?term=17328068]

Institution

(Van Oijen, De Jong, Witteman, Hofman, Breteler) Department of Epidemiology and Biostatistics, Erasmus Medical Center, Rotterdam, Netherlands (Van Oijen, De Jong, Koudstaal) Department of Neurology, Erasmus Medical Center, Rotterdam, Netherlands
(Breteler) Department of Epidemiology and Biostatistics, Erasmus Medical Center, PO Box 1738, 3000 DR Rotterdam, Netherlands

Publisher

John Wiley and Sons Inc. (P.O.Box 18667, Newark NJ 07191-8667, United States)

Emtree Heading

adult; aged; Alzheimer disease; ankle brachial index; artery disease; artery intima proliferation; article; *atherosclerosis; atherosclerotic plaque; carotid artery; cerebrovascular disease; *dementia; disease association; female; follow up; genotype; human; major clinical study; male; mortality; multiinfarct dementia; non invasive measurement; priority journal; apolipoprotein E/ec [Endogenous Compound].

Drug Index Terms

apolipoprotein E / endogenous compound.

Other Index Terms

adult; aged; Alzheimer disease; ankle brachial index; artery disease; artery intima proliferation; article; *atherosclerosis; atherosclerotic plaque; carotid artery; cerebrovascular disease; *dementia; disease association; female; follow up; genotype; human; major clinical study; male; mortality; multiinfarct dementia; non invasive measurement; priority journal.

Link to the Ovid Full Text or citation:

Click here for full text options

Link to the External Link Resolver:

For peer review only - http://bmjopen.bmj.com/site/about/guidelines.xhtml

SFX

360.

Cognitive impairment and carotid atherosclerosis in a general Italian midlife and old population.
Prati P., Casaroli M., Bignamini A., Scotti S., Canciani L., Ruscio M., Balestrieri M., Bornstein N., Zanetti O., Tosetto A., Castellani S., Pantoni L., Touboul P.J., Inzitari D.
Neuroepidemiology. 27 (1) (pp 33-38), 2006. Date of Publication: July 2006.
AN: 44114409
The authors describe the design and the general, ultrasonographic, neuropsychological methodology of an observational epidemiological population survey, named REMEMBER (Registry Evaluation Memory in Buttrio e Remanzacco) conducted in the northeast of Italy in a randomized stratified sample of 1,026 subjects (554 F and 472 M) aged 55-98 years. The study was planned as cross-sectional and longitudinal survey of cognitive impairment, cardiovascular risk factors, carotid atherosclerosis in a midlife and older Italian population sample. The objectives of the first phase are to assess the prevalence of the different types of dementia, the cognitive impairment non-dementia, the cardiovascular risk factors, the carotid intima-media thickness and arterial distensibility, and of depression. The conclusions of this study will make it possible to organize preventive and interventional strategies for these epidemic conditions. Copyright © 2006 S. Karger AG.
PMID
16804332 [http://www.ncbi.nlm.nih.gov/pubmed/?term=16804332]
Institution
(Prati, Casaroli, Scotti) Department of Neurology, Gervasutta Hospital, Udine, Italy (Bignamini) School of Pharmacy, University of Milan, Milan, Italy
(Canciani) GP, Udine, Italy
(Ruscio) Analysis Laboratory, S. Daniele Hospital, San Daniele, Italy
(Balestrieri) Department of Psychiatry, University of Udine, Udine, Italy
(Bornstein) Department of Neurology, Sourasky Medical Center, Tel Aviv, Israel
(Zanetti) Alzheimer Unit, IRCCS, Fatebenefratelli Institute, Brescia, Italy
(Tosetto) Department of Haematology, S. Bortolo Hospital, Vicenza, Italy
(Castellani) Department of Angiology, University of Florence, Florence, Italy
(Pantoni, Inzitari) Department of Neurology, University of Florence, Florence, Italy
(Touboul) Department of Neurology, Bichat University Hospital, Paris, France

For peer review only

(Prati) via Grazzano 3, IT-33100 Udine, Italy
Publisher
S. Karger AG (Allschwilerstrasse 10, P.O. Box, Basel CH-4009, Switzerland)
Emtree Heading
adult; aged; Alzheimer disease; artery compliance; article; *atherosclerosis; cardiovascular risk; carotid artery; cerebrovascular disease; *cognitive defect; correlation analysis; dementia/ep [Epidemiology]; depression; disease severity; echography; ethnic group; female; human; Italy; major clinical study; male; morbidity; multiinfarct dementia; neuropsychology; population; prevalence; risk factor; sample.
Other Index Terms
adult; aged; Alzheimer disease; artery compliance; article; *atherosclerosis; cardiovascular risk; carotid artery; cerebrovascular disease; *cognitive defect; correlation analysis; dementia / epidemiology; depression; disease severity; echography; ethnic group; female; human; Italy; major clinical study; male; morbidity; multiinfarct dementia; neuropsychology; population; prevalence; risk factor; sample.

Link to the Ovid Full Text or citation:
Click here for full text options

Link to the External Link Resolver:
SFX

366.

Cognitive functions in carotid artery disease before endarterectomy.
Bossema E.R., Brand N., Moll F.L., Ackerstaff R.G.A., De Haan E.H.F., Van Doornen L.J.P.
Journal of Clinical and Experimental Neuropsychology. 28 (3) (pp 357-369), 2006. Date of Publication: April 2006.
AN: 43477936
Restorative effects of carotid endarterectomy (CEA) on cognitive functioning in patients with severe atherosclerotic disease presuppose the existence of cognitive deficits prior to the intervention. Thorough examination of this premise received only minor attention. The present study assessed symptomatic and asymptomatic patients with severe unilateral or bilateral stenosis

For peer review only - http://bmjopen.bmj.com/site/about/guidelines.xhtml

of the carotid arteries one day before CEA. Healthy volunteers with similar demographic characteristics served as control subjects. Patients overall showed decreased functioning on tests of attention, verbal and visual memory, verbal fluency, and psychomotor speed and executive functioning, even after correction for the effects of mood. Simple motor skills and visuospatial functioning were not affected. Patients grouped according to presence and type of previous clinical symptoms and severity of contralateral stenosis only slightly differed from each other. The findings leave open the potential of improving cognitive function after CEA. Copyright © Taylor & Francis Group, LLC.

PMID
16618625 [http://www.ncbi.nlm.nih.gov/pubmed/?term=16618625]

Institution
(Bossema, Brand, Van Doornen) Department of Health Psychology, Utrecht University, Netherlands
(Moll) Department of Vascular Surgery, University Medical Centre Utrecht, Netherlands
(Ackerstaff) Department of Clinical Neurophysiology, St. Antonius Hospital, Nieuwegein, Netherlands
(De Haan) Department of Psychonomics, Utrecht University, Netherlands
(Bossema) Department of Medical Decision Making, Leiden University Medical Centre, P.O. Box 9600, 2300 RC Leiden, Netherlands

Publisher
Psychology Press Ltd (4 Park Square, Milton Park, Abingdon, Oxfordshire OX14 4RN, United Kingdom)

Emtree Heading
aged; article; atherosclerosis; *carotid artery disease; *carotid endarterectomy; *cognition; controlled study; demography; depth perception; female; human; major clinical study; male; motor performance; priority journal; psychomotor activity; stenosis; verbal memory; visual memory.

Other Index Terms
aged; article; atherosclerosis; *carotid artery disease; *carotid endarterectomy; *cognition; controlled study; demography; depth perception; female; human; major clinical study; male; motor performance; priority journal; psychomotor activity; stenosis; verbal memory; visual memory.

Link to the Ovid Full Text or citation:
Click here for full text options

Link to the External Link Resolver:
SFX

For peer review only

373.

Effect of carotid endarterectomy on patient evaluations of cognitive functioning and mental and physical health.

Bossema E.R., Brand A.N., Geenen R., Moll F.L., Ackerstaff R.G.A., Van Doornen L.J.P.

Annals of Vascular Surgery. 19 (5) (pp 673-677), 2005. Date of Publication: September 2005.

AN: 41218947

The prophylactic effect of carotid endarterectomy (CEA) against stroke has been well established. As a consequence of the restoration of cerebral blood supply and reduced risk of stroke, cognitive functioning and perceived health may improve. Fifty-one patients with severe atherosclerotic disease of the carotid artery but without history of major stroke completed the Cognitive Failures Questionnaire and the Short Form 36 Health Survey before CEA and 3 and 12 months thereafter. Before CEA, patients reported significant but small deviations from the norm in physical function, general health, and vitality. Small improvements after CEA were observed in the perception of physical role function, general health, vitality, and mental health. Patients also retrospectively noted a slight worsening of health in the year before surgery and some improvement after surgery. Evaluation of cognitive failures in daily life did not change. Demographic or medical characteristics, such as a history of temporary ischemic symptoms, occlusion of the contralateral artery, and shunt use during surgery, did not affect outcome. In conclusion, no negative outcomes and even some limited positive effects in the perception of mental and physical health are to be expected after CEA. © Annals of Vascular Surgery Inc.

PMID

16078006 [http://www.ncbi.nlm.nih.gov/pubmed/?term=16078006]

Institution

(Bossema, Brand, Geenen, Van Doornen) Department of Health Psychology, Utrecht University, Utrecht, Netherlands (Moll) Department of Vascular Surgery, University Medical Center Utrecht, Netherlands

(Ackerstaff) Department of Clinical Neurophysiology, St. Antonius Hospital, Nieuwegein, Netherlands

(Bossema) Department of Health Psychology, Utrecht University, PO Box 80140, 3508, TC, Utrecht, Netherlands

Publisher

Elsevier Inc. (360 Park Avenue South, New York NY 10010, United States)

Emtree Heading

For peer review only

adult; artery occlusion; article; atherosclerosis; carotid artery; *carotid endarterectomy; *cognition; daily life activity; demography; disease severity; female; health; health survey; human; ischemia; major clinical study; male; *mental health; patient; perception; priority journal; questionnaire; retrospective study; Short Form 36; shunt infection; stroke.

Other Index Terms

adult; artery occlusion; article; atherosclerosis; carotid artery; *carotid endarterectomy; *cognition; daily life activity; demography; disease severity; female; health; health survey; human; ischemia; major clinical study; male; *mental health; patient; perception; priority journal; questionnaire; retrospective study; short form 36; shunt infection; stroke.

Link to the Ovid Full Text or citation:

Click here for full text options

Link to the External Link Resolver:

SFX

375.

Arterial stiffness is an independent risk factor for cognitive impairment in the elderly: A pilot study.

Scuteri A., Brancati A.M., Gianni W., Assisi A., Volpe M.

Journal of Hypertension. 23 (6) (pp 1211-1216), 2005. Date of Publication: June 2005.

AN: 40862725

Background: Loss of cognitive function is a common condition in the elderly population. Cognitive impairment is defined as the transitional stage of cognitive decline, between normal aging and early dementia. We tested whether arterial stiffness, evaluated as pulse wave velocity (PWV), is associated with cognitive impairment in older subjects, and whether PWV is increased at a comparable extent in older subjects with cortical or subcortical cerebral lesions when compared with age-matched controls referred for memory deficits. Subjects and methods: Eighty-four subjects (78 +/- 5 years, 30 men and 54 women) referred for memory deficit with no history of stroke or atrial fibrillation were studied. Carotid-femoral PWV was determined non-invasively with Complior. The Mini Mental State Examination was assessed as a measure of global cognitive function. The sum of the score on the Activities of Daily Living and Instrumental Activities of Daily Living scales was used as a measure of personal independency. Based upon brain imaging,

For peer review only

subjects were classified as referred for memory deficits with normal brain imaging, or control, with subcortical microvascular lesions or with cortical atrophy.

Result(s): PWV, normalized for mean blood pressure, was inversely correlated with the Mini Mental State Examination (r = -0.26, P < 0.05), even after controlling for education, prevalent cardiovascular (CV) disease, CV risk factors, and medication use (beta coefficient = -0.28, P < 0.01). PWV was also inversely correlated with personal independency (r = -0.36, P < 0.01; beta coefficient = -0.38, P < 0.01, after multiple adjustment). In the presence of no significant differences in age, education, traditional CV risk factor levels, carotid plaques, or prevalence of CV disease, higher PWV values were more frequent in subjects with cortical atrophy than in patients with subcortical microvascular lesions or controls (P < 0.05).

Conclusion(s): PWV was associated with cognitive impairment and with a greater personal dependency, independently of major modifiable CV risk factors. © 2005 Lippincott Williams & Wilkins.

PMID

15894897 [http://www.ncbi.nlm.nih.gov/pubmed/?term=15894897]

Institution

(Scuteri, Brancati, Gianni, Assisi) UO Geriatria, INRCA/IRCCS, Roma, Italy (Volpe) Cattedra Cardiologia, Universita La Sapienza, Roma, Italy

(Volpe) IRCCS Neuromed, Pozzilli, Italy

(Scuteri) UO Geriatria, INRCA/IRCCS, Via Cassia 1167, 00189 Roma, Italy

Publisher

Lippincott Williams and Wilkins (250 Waterloo Road, London SE1 8RD, United Kingdom)

Emtree Heading

aged; aging; *artery compliance; atherosclerotic plaque; brain atrophy; cardiovascular disease; cardiovascular risk; carotid artery; cognition; *cognitive defect; conference paper; daily life activity; dementia; drug use; female; femoral artery; heart atrium fibrillation; human; major clinical study; male; memory disorder; Mini Mental State Examination; neuroimaging; priority journal; pulse wave; stroke.

Other Index Terms

aged; aging; *artery compliance; atherosclerotic plaque; brain atrophy; cardiovascular disease; cardiovascular risk; carotid artery; cognition; *cognitive defect; conference paper; daily life activity; dementia; drug use; female; femoral artery; heart atrium fibrillation; human; major clinical study; male; memory disorder; mini mental state examination; neuroimaging; priority journal; pulse wave; stroke.

Link to the Ovid Full Text or citation:

For peer review only - http://bmjopen.bmj.com/site/about/guidelines.xhtml

Click here for full text options

Link to the External Link Resolver:
SFX

379.

Left or right carotid endarterectomy in patients with atherosclerotic disease: Ipsilateral effects on cognition?.

Brand N., Bossema E.R., Van Ommen M., Moll F.L., Ackerstaff R.G.A.

Brain and Cognition. 54 (2) (pp 117-123), 2004. Date of Publication: March 2004.

AN: 38229765

We evaluated hemispheric functions ipsilateral to the side of carotid endarterectomy (CEA) in patients with a severe stenosis in the left or right carotid artery. Assessments took place 1 day before and 3 months after CEA. Only right-handed males were included. Nineteen patients underwent surgery of the left carotid artery and 17 of the right. Valid instruments for hemispheric function were included, such as verbal dichotic listening, finger tapping, and a lateralised test for motor planning. Results showed that, preoperatively, patients had lower scores compared to norms on the laterality tests, and on a visuoconstructive test. There was no evidence of ipsilateral improvement related to side of surgery. Left ear dichotic listening improved, which was seen in both left and right surgery groups. Also in both groups, left- and right-hand movement speed in the motor planning test decreased. Conclusion is that beneficial ipsilateral cognitive change after CEA in patients with severe stenosis in one of the carotid arteries may not be demonstrated, even if valid instruments for hemispheric function are included. © 2004 Elsevier Inc. All rights reserved.

PMID

14980452 [http://www.ncbi.nlm.nih.gov/pubmed/?term=14980452]

Institution

(Brand, Bossema) Department of Health Psychology, Res. Inst. for Psychology and Health, Utrecht University, Utrecht TC, Netherlands (Van Ommen) Heliomare Rehabilitation Centre, Wijk aan Zee, Netherlands

(Moll) Department of Vascular Surgery, University Medical Center Utrecht, Netherlands

(Ackerstaff) Department Clinical Neurophysiology, St. Antonius Hospital, Nieuwegein, Netherlands

Publisher

Academic Press Inc. (6277 Sea Harbor Drive, Orlando FL 32887-4900, United States)

For peer review only - http://bmjopen.bmj.com/site/about/guidelines.xhtml

Emtree Heading
adult; aged; article; *atherosclerosis/su [Surgery]; *carotid artery; clinical article; *cognition; controlled study; *endarterectomy; evaluation; hemisphere; human; male; priority journal; right handedness; validation process.
Other Index Terms
adult; aged; article; *atherosclerosis / *surgery; *carotid artery; clinical article; *cognition; controlled study; *endarterectomy; evaluation; hemisphere; human; male; priority journal; right handedness; validation process.

Link to the Ovid Full Text or citation:
Click here for full text options

Link to the External Link Resolver:
SFX

381.

Carotid intima-media thickness and cognitive decline: What does it mean for prevention of dementia?.
Spence J.D.
Journal of the Neurological Sciences. 223 (2) (pp 103-105), 2004. Date of Publication: 30 Aug 2004.
AN: 39164350
PMID
15337609 [http://www.ncbi.nlm.nih.gov/pubmed/?term=15337609]
Institution
(Spence) Stroke Prev. Atherosclerosis Res. C., Siebens-Drake Centre, Robarts Research Institute, 1400 Western Road, London, Ont. N6G 2V2, Canada
Publisher
Elsevier (P.O. Box 211, Amsterdam 1000 AE, Netherlands)
Emtree Heading
atherosclerosis; *carotid artery; carotid artery obstruction; *cognitive defect/dt [Drug Therapy]; *cognitive defect/pc [Prevention]; *dementia/dt [Drug Therapy]; *dementia/pc [Prevention]; diabetes mellitus; disease predisposition; editorial; heart infarction; human; hypertension; *intima;

For peer review only

mixed depression and dementia; multiinfarct dementia; priority journal; prophylaxis; risk assessment; stroke; *thickness; treatment indication; *tunica media; atenolol/dt [Drug Therapy]; atenolol/pd [Pharmacology]; candesartan/dt [Drug Therapy]; candesartan/pd [Pharmacology]; dipeptidyl carboxypeptidase inhibitor/dt [Drug Therapy]; dipeptidyl carboxypeptidase inhibitor/pd [Pharmacology]; enalapril/dt [Drug Therapy]; enalapril/pd [Pharmacology]; losartan/dt [Drug Therapy]; losartan/pd [Pharmacology]; valsartan/dt [Drug Therapy]; valsartan/pd [Pharmacology].

Drug Index Terms

atenolol / drug therapy / pharmacology; candesartan / drug therapy / pharmacology; dipeptidyl carboxypeptidase inhibitor / drug therapy / pharmacology; enalapril / drug therapy / pharmacology; losartan / drug therapy / pharmacology; valsartan / drug therapy / pharmacology.

Other Index Terms

atherosclerosis; *carotid artery; carotid artery obstruction; *cognitive defect / *drug therapy / *prevention; *dementia / *drug therapy / *prevention; diabetes mellitus; disease predisposition; editorial; heart infarction; human; hypertension; *intima; mixed depression and dementia; multiinfarct dementia; priority journal; prophylaxis; risk assessment; stroke; *thickness; treatment indication; *tunica media.

Link to the Ovid Full Text or citation:

[Click here for full text options]

Link to the External Link Resolver:

[SFX]

384.

Small Dense Low-Density Lipoprotein and Carotid Atherosclerosis in Relation to Vascular Dementia.

Watanabe T., Koba S., Kawamura M., Itokawa M., Idei T., Nakagawa Y., Iguchi T., Katagiri T.

Metabolism: Clinical and Experimental. 53 (4) (pp 476-482), 2004. Date of Publication: April 2004.

AN: 38419191

Vascular dementia (VaD) and Alzheimer's disease (AD) are the most common causes of dementia in the elderly. The aim of this study was to investigate carotid atherosclerosis, serum lipid profiles, and atherogenic hormone levels in nondiabetic Japanese men with VaD or AD. Carotid artery

For peer review only

intima-media thickness (IMT) and plaque, serum lipid and lipoprotein profiles, including low-density lipoprotein (LDL) particle size, as well as insulin-like growth factor-I (IGF-I, somatomedin C) and testosterone levels, were determined in 34 patients with AD, 37 patients with VaD, and 63 healthy male controls. Age, body mass index, systolic and diastolic blood pressure, and fasting plasma glucose, hemoglobin A1c (HbA1c), triglyceride, high-density lipoprotein (HDL)-cholesterol, and apolipoproteins (apo) A-I, B, and E levels did not differ significantly among the 3 groups. However, the mean value of carotid IMT, the frequency of atherosclerotic plaque deposition, the serum levels of LDL-cholesterol, lipoprotein(a), and lipid peroxides, and the incidence of small dense LDL (particle diameter <= 25.5 nm) were increased significantly in VaD patients compared with AD patients or controls. VaD patients had a close reverse correlation between carotid IMT and LDL particle diameter, which were statistically proven independent risk factors for VaD. In contrast, AD patients had significantly lower serum levels of IGF-I and testosterone than either VaD patients or contols. Our results indicate that VaD is associated with atherogenic dyslipidemia, in particular, small dense LDL and carotid atherosclerosis, whereas AD is associated with hyposomatomedinemia and hypogonadism rather than atherosclerosis. © 2004 Elsevier Inc. All rights reserved.

PMID

15045695 [http://www.ncbi.nlm.nih.gov/pubmed/?term=15045695]

Institution

(Watanabe) Department of Biochemistry, Showa University School of Medicine, 1-5-8 Hatanodai, Shinagawa-ku, Tokyo 142-8555, Japan

Publisher

W.B. Saunders (Independence Square West, Philadelphia PA 19106-3399, United States)

Emtree Heading

adult; aged; alcohol consumption; Alzheimer disease; artery intima proliferation; article; atherogenesis; *atherosclerosis; atherosclerotic plaque; controlled study; dementia/ep [Epidemiology]; disease association; dyslipidemia/co [Complication]; dyslipidemia/di [Diagnosis]; female; human; hypogonadism; intravascular ultrasound; lipid blood level; lipoprotein blood level; major clinical study; male; *multiinfarct dementia; particle size; priority journal; risk factor; smoking; testosterone blood level; alcohol; lipid/ec [Endogenous Compound]; lipid peroxide/ec [Endogenous Compound]; lipoprotein/ec [Endogenous Compound]; lipoprotein A/ec [Endogenous Compound]; *low density lipoprotein/ec [Endogenous Compound]; low density lipoprotein cholesterol/ec [Endogenous Compound]; somatomedin C/ec [Endogenous Compound]; testosterone/ec [Endogenous Compound]; unclassified drug; *carotid atherosclerosis; *small dense low density lipoprotein/ec [Endogenous Compound].

Candidate Terms

For peer review only - http://bmjopen.bmj.com/site/about/guidelines.xhtml

*carotid atherosclerosis [other term]; *small dense low density lipoprotein / *endogenous compound [drug term].

Drug Index Terms

alcohol; lipid / endogenous compound; lipid peroxide / endogenous compound; lipoprotein / endogenous compound; lipoprotein A / endogenous compound; *low density lipoprotein / *endogenous compound; low density lipoprotein cholesterol / endogenous compound; somatomedin C / endogenous compound; testosterone / endogenous compound; unclassified drug.

Other Index Terms

adult; aged; alcohol consumption; Alzheimer disease; artery intima proliferation; article; atherogenesis; *atherosclerosis; atherosclerotic plaque; controlled study; dementia / epidemiology; disease association; dyslipidemia / complication / diagnosis; female; human; hypogonadism; intravascular ultrasound; lipid blood level; lipoprotein blood level; major clinical study; male; *multiinfarct dementia; particle size; priority journal; risk factor; smoking; testosterone blood level.

Link to the Ovid Full Text or citation:

Click here for full text options

Link to the External Link Resolver:

SFX

388.

Peripheral arterial disease and cognitive function.

Waldstein S.R., Tankard C.F., Maier K.J., Pelletier J.R., Snow J., Gardner A.W., Macko R., Katzel L.I.

Psychosomatic Medicine. 65 (5) (pp 757-763), 2003. Date of Publication: September/October 2003.

AN: 37163380

Objective: Peripheral arterial disease (PAD) is associated with comorbid atherosclerosis of the coronary and carotid arteries and is a significant risk factor for stroke. However, cognitive function in PAD patients before clinically evident stroke remains poorly characterized. Here we hypothesized that, on neuropsychological testing, PAD patients would perform more poorly than healthy control subjects, and persons with mild cardiovascular disease (essential hypertension), but

For peer review only - http://bmjopen.bmj.com/site/about/guidelines.xhtml

better than stroke patients, thus reflecting a continuum of cognitive impairment associated with increased severity of vascular disease.

Method(s): The cognitive performance of 38 PAD patients (mean ankle-brachial index=0.67, Fontaine Class II) was contrasted with that of 23 healthy normotensive controls, 20 essential hypertensives, and 26 anterior ischemic stroke patients on twelve neuropsychological tests.

Result(s): PAD patients performed significantly more poorly than hypertensives and normotensives, but better than stroke patients, on seven tests of nonverbal memory, concentration, executive function, perceptuo-motor speed, and manual dexterity. Hypertensives displayed poorer performance than normotensives on tests of nonverbal memory and manual dexterity. These findings were independent of age, education, and depression scores. Higher diastolic blood pressure and plasma glucose levels predicted poorer performance of select cognitive tests by PAD patients. Eight to 67% of PAD patients displayed impaired performance (< 5th percentile of normotensive controls) on the seven aforementioned cognitive tests.

Conclusion(s): PAD patients exhibit diminished performance across a variety of domains of cognitive function. Findings also suggest a continuum of cognitive impairment associated with increasingly severe manifestations of cardiovascular disease, thus emphasizing the need for enhanced preventative measures to avert functional declines.

PMID

14508017 [http://www.ncbi.nlm.nih.gov/pubmed/?term=14508017]

Institution

(Waldstein, Tankard, Maier, Pelletier, Snow) Department of Psychology, University of Maryland, Baltimore County, Baltimore, MD, United States (Waldstein, Gardner, Macko, Katzel) Department of Medicine, Univ. of Maryland School of Medicine, Baltimore Vet. Aff. Medical Center, Baltimore, MD, United States

(Waldstein) Department of Psychology, University of Maryland, Baltimore County, 1000 Hilltop Circle, Baltimore, MD 21250, United States

Publisher

Lippincott Williams and Wilkins (351 West Camden Street, Baltimore MD 21201-2436, United States)

Emtree Heading

adult; *artery disease; article; atherosclerosis; cerebrovascular accident; clinical article; *cognition; cognitive defect; comorbidity; controlled study; diastolic blood pressure; disease severity; female; glucose blood level; human; hypertension; male; memory; neuropsychological test; neuropsychology; priority journal; risk factor; stroke/et [Etiology]; glucose/ec [Endogenous Compound].

Drug Index Terms

glucose / endogenous compound.

Other Index Terms

For peer review only - http://bmjopen.bmj.com/site/about/guidelines.xhtml

adult; *artery disease; article; atherosclerosis; cerebrovascular accident; clinical article; *cognition; cognitive defect; comorbidity; controlled study; diastolic blood pressure; disease severity; female; glucose blood level; human; hypertension; male; memory; neuropsychological test; neuropsychology; priority journal; risk factor; stroke / etiology.

Link to the Ovid Full Text or citation:
Click here for full text options

Link to the External Link Resolver:
SFX

404.

Ultrasonographic assessment of carotid wall characteristics and cognitive functions in a community sample of 59- to 71-year-olds.
Auperin A., Berr C., Bonithon-Kopp C., Touboul P.-J., Ruelland I., Ducimetiere P., Alperovitch A.
Stroke. 27 (8) (pp 1290-1295), 1996. Date of Publication: August 1996.
AN: 26286154
Background and Purpose: This study was aimed at analyzing cross-section relationships between cognitive performance and ultrasonographic assessment of carotid wall characteristics.
Method(s): A cohort of 1279 subjects (men, 41%) aged 59 to 71 years was recruited from the electoral rolls of the city of Nantes (western France). Cognitive performances were evaluated with the Mini-Mental State Examination MMSE) and seven neuropsychological tests assessing attention, psychomotor rapidity, verbal abilities, memory, and visuospatial perception. For each tests, subject were classified into three performance levels with a quartile distribution: 25% lowest, and 50% middle. The intima-media thickness of common arteries and the presence of plaques in the carotid arteries were assessed with B-mode ultrasound examination.
Result(s): Only 28% of men and 17% of women had carotid plaques inducing moderate stenosis of the lumen (<40%). After adjustment for possible confounders, odds ratios for poor cognitive performance associated with plaques were above 1 for all cognitive tests in men. This association was statistically significant for the MMSE and another test assessing attention skills. There was a slight association between increase of the common carotid intima-media thickness and poor

For peer review only

cognitive scores in men with plaques. In women, no association was found between cognitive functions and presence of plaques or intima-media thickness.

Conclusion(s): This study indicated a moderate association between atherosclerosis of the carotid arteries and poor cognitive functioning in men aged 59 to 79 years. In view of these moderate cross-sectional results, further studies are required to better assess the relationship between carotid atherosclerosis and cognitive impairment.

PMID

8711788 [http://www.ncbi.nlm.nih.gov/pubmed/?term=8711788]

Institution

(Auperin, Berr, Alperovitch) INSERM U360, Hop. de la Salpetriere, Paris, France (Bonithon-Kopp, Ducimetiere) INSERM U258, Hopital Broussais, Paris, France

(Touboul) Service de Neurologie, Hopital Saint Antoine, Paris, France

(Ruelland) Centre EVA, Nantes, France

(Berr) INSERM U360, Hop. de la Salpetriere, 47 Blvd de l'Hopital, 75651 Paris Cedex 13, France

Publisher

Lippincott Williams and Wilkins (530 Walnut Street,P O Box 327, Philadelphia PA 19106-3621, United States)

Emtree Heading

adult; aged; aging; artery wall; article; atherosclerosis; atherosclerotic plaque; *carotid artery anomaly/di [Diagnosis]; carotid artery bifurcation; *cognition; cognitive defect; echography; female; human; major clinical study; male; neuropsychological test; priority journal; risk assessment.

Other Index Terms

adult; aged; aging; artery wall; article; atherosclerosis; atherosclerotic plaque; *carotid artery anomaly / *diagnosis; carotid artery bifurcation; *cognition; cognitive defect; echography; female; human; major clinical study; male; neuropsychological test; priority journal; risk assessment.

Link to the Ovid Full Text or citation:

Click here for full text options

Link to the External Link Resolver:

SFX

406.

For peer review only - http://bmjopen.bmj.com/site/about/guidelines.xhtml

Cardiovascular disease and distribution of cognitive function in elderly people: The Rotterdam study.

Breteler M.M.B., Claus J.J., Grobbee D.E., Hofman A.

British Medical Journal. 308 (6944) (pp 1604-1608), 1994. Date of Publication: 18 Jun 1994.

AN: 24181869

Objective - To investigate the distribution of cognitive function in elderly people and to assess the impact of clinical manifestations of atherosclerotic disease on this distribution. Design - Single centre population based cross sectional door to door study. Setting - Ommoord, a suburb of Rotterdam, the Netherlands. Subjects - 4971 subjects aged 55 to 94 years. Main outcome measure -Cognitive function as measured by the mini mental state examination. Results - The overall participation rate in the study was 80%. Cognitive test data were available for 90% of the participants. Increasing age and lower educational level were associated with poorer cognitive function. Previous vascular events, presence of plaques in the carotid arteries, and presence of peripheral arterial atherosclerotic disease were associated with worse cognitive performance independent of the effects of age and education. On average the differences were moderate; however, they reflected the net result of a shift of the total population distribution of cognitive function towards lower values. Thereby, they resulted in a considerable increase in the proportion of subjects with scores indicative of dementia. Conclusions - These findings are compatible with the view that atherosclerotic disease accounts for considerable cognitive impairment in the general population.

PMID

8025427 [http://www.ncbi.nlm.nih.gov/pubmed/?term=8025427]

Institution

(Breteler, Claus, Grobbee, Hofman) Depts. Epidemiol. Biostatist. N., Erasmus University Medical School, Rotterdam, Netherlands

Publisher

BMJ Publishing Group (Tavistock Square, London WC1H 9JR, United Kingdom)

Emtree Heading

adult; aged; *aging; article; *atherosclerosis/ep [Epidemiology]; cardiovascular disease/ep [Epidemiology]; clinical trial; *cognitive defect/ep [Epidemiology]; *cognitive defect/et [Etiology]; controlled clinical trial; controlled study; *dementia/ep [Epidemiology]; *dementia/et [Etiology]; female; human; major clinical study; male; Netherlands; priority journal.

Other Index Terms

adult; aged; *aging; article; *atherosclerosis / *epidemiology; cardiovascular disease / epidemiology; clinical trial; *cognitive defect / *epidemiology / *etiology; controlled clinical trial;

For peer review only - http://bmjopen.bmj.com/site/about/guidelines.xhtml

controlled study; *dementia / *epidemiology / *etiology; female; human; major clinical study; male; netherlands; priority journal.

Link to the Ovid Full Text or citation:
Click here for full text options

Link to the External Link Resolver:
SFX

407.

Carotid stenosis and atherosclerotic heart disease: Interactive effects on cognitive status.
Seidenberg M., Parker J.C., Nichols W.K.
International Journal of Clinical Neuropsychology. 7 (1) (pp 45-48), 1985. Date of Publication: 1985.
AN: 15094490
It is quite common for patients with carotid artery disease to also suffer from other chronic medical disease. To date, there have been few systematic investigations of the influence and interaction of such associated medical problems. In this paper, we present the results of a pilot study using a retrospective approach to investigate the interaction of carotid artery disease and atherosclerotic heart disease for neuropsychological performance. Forty-six patients with carotid artery disease were divided into four groups: 1) discrete carotid lesions and heart disease, b) discrete carotid lesions alone, c) generalized cerebrovascular disease and heart disease, and d) generalized cerebrovascular disease alone. Results indicated a consistent pattern of greater cognitive impairment in those carotid artery patients who have both generalized cerebrovascular disease and atherosclerotic heart disease which was most evident on tests of nonverbal memory abilities. The potential significance of these findings for both clinical assessment and methodological considerations in studying carotid artery disease is discussed.
Institution
(Seidenberg, Parker, Nichols) Department of Psychology, University of Health Sciences/The Chicago Medical School, North Chicago, IL 60064 United States
Emtree Heading
*atherosclerosis; cardiovascular system; *carotid artery obstruction; central nervous system; clinical article; *cognition; heart; *heart disease; human; peripheral vascular system; psychological aspect.

For peer review only - http://bmjopen.bmj.com/site/about/guidelines.xhtml

Other Index Terms

*atherosclerosis; cardiovascular system; *carotid artery obstruction; central nervous system; clinical article; *cognition; heart; *heart disease; human; peripheral vascular system; psychological aspect.

Link to the Ovid Full Text or citation:

Click here for full text options

Link to the External Link Resolver:

SFX

For peer review only

Database: Ovid MEDLINE(R) and Epub Ahead of Print, In-Process & Other Non-Indexed Citations and Daily <1946 to February 26, 2020>
Search Strategy:
----
1 cognition/ (94748)
2 cognitive function*.tw. (58742)
3 dementia/ (49736)
4 Alzheimer disease/ (91342)
5 1 or 2 or 3 or 4 (257625)
6 (atheroscolerosis or intima media thickness or plaque).mp. (119020)
7 atherosclerosis/ (35080)
8 6 or 7 (145302)
9 5 and 8 (6774)
10 Carotid Arteries/ (32174)
11 carotid arter*.mp. (102137)
12 10 or 11 (102137)
13 9 and 12 (124)

****

2.

A Cross-Sectional Investigation of Cognition and Ultrasound-Based Vascular Strain Indices.
Meshram NH; Jackson D; Varghese T; Mitchell CC; Wilbrand SM; Dempsey RJ; Hermann BP.
Archives of Clinical Neuropsychology. 35(1):46-55, 2019 Jan 24.
[Journal Article]
UI: 30805597
OBJECTIVE: We examine the relationship between variability in the plaque strain distribution estimated using ultrasound with multiple cognitive domains including executive, language, visuospatial reasoning, and memory function.
METHOD: Asymptomatic (n = 42) and symptomatic (n = 34) patients with significant (>60%) carotid artery stenosis were studied for plaque instability using ultrasound strain imaging and

For peer review only

multiple cognitive domains including executive, language, visuospatial reasoning, and memory function. Correlation and ROC analyses were performed between ultrasound strain indices and cognitive function. Strain indices and cognition scores were also compared between symptomatic and asymptomatic patients to determine whether there are significant group differences.

RESULTS: Association of high-strain distributions with dysexecutive function was observed in both asymptomatic and symptomatic patients. For memory, visuospatial, and language functions, the correlations between strain and cognition were weaker for the asymptomatic compared to symptomatic group.

CONCLUSIONS: Both asymptomatic and symptomatic patients demonstrate a relationship between vessel strain indices and executive function indicating that silent strokes and micro-emboli could initially contribute to a decline in executive function, whereas strokes and transient ischemic attacks may cause the further decline in other cognitive functions.

Copyright © The Author(s) 2019. Published by Oxford University Press. All rights reserved. For permissions, please e-mail: journals.permissions@oup.com.

Version ID

1

Record Owner

From MEDLINE, a database of the U.S. National Library of Medicine.

Status

In-Process

Authors Full Name

Meshram, N H; Jackson, D; Varghese, T; Mitchell, C C; Wilbrand, S M; Dempsey, R J; Hermann, B P.

Institution

Meshram, N H. Department of Medical Physics, University of Wisconsin School of Medicine and Public Health, Madison, WI, USA. Meshram, N H. Department of Electrical and Computer Engineering, University of Wisconsin-Madison, Madison, WI, USA.

Jackson, D. Department of Neurology, University of Wisconsin School of Medicine and Public Health, Madison, WI, USA.

Varghese, T. Department of Medical Physics, University of Wisconsin School of Medicine and Public Health, Madison, WI, USA.

Varghese, T. Department of Electrical and Computer Engineering, University of Wisconsin-Madison, Madison, WI, USA.

Mitchell, C C. Department of Medicine, University of Wisconsin School of Medicine and Public Health, Madison, WI, USA.

Wilbrand, S M. Department of Neurological Surgery, University of Wisconsin School of Medicine and Public Health, Madison, WI, USA.

For peer review only - http://bmjopen.bmj.com/site/about/guidelines.xhtml

Dempsey, R J. Department of Neurological Surgery, University of Wisconsin School of Medicine and Public Health, Madison, WI, USA.
Hermann, B P. Department of Neurology, University of Wisconsin School of Medicine and Public Health, Madison, WI, USA.
Keyword Heading
Carotid stenosis Cerebrovascular disease
Cognitive impairment
Early diagnosis
Executive functioning
Neuropsychological tests.
Year of Publication
2019

Link to the Ovid Full Text or citation:
Click here for full text options

Link to the External Link Resolver:
SFX

4.

Carotid Intima-media Thickness, Cognitive Performance and Cognitive Decline in Stroke-free Middle-aged and Older Adults. The Atahualpa Project.
Del Brutto OH; Mera RM; Recalde BY; Del Brutto VJ.
Journal of Stroke & Cerebrovascular Diseases. 29(2):104576, 2020 Feb.
[Journal Article]
UI: 31839546
BACKGROUND: Little is known on factors influencing cognitive function in rural communities. Using the Atahualpa Project cohort, we aimed to assess whether the carotid intima-media thickness (cIMT) - used as a surrogate of extracranial carotid atherosclerosis - is associated with cognitive performance and further decline in community-dwelling adults living in a rural setting. METHODS: The study included Atahualpa residents aged greater than or equal to 40 years who had ultrasound examination of the extracranial carotid arteries and a baseline Montreal Cognitive

For peer review only - http://bmjopen.bmj.com/site/about/guidelines.xhtml

Assessment (MoCA), as well as the subset of individuals who also had a follow-up MoCA at least 1 year after baseline. Relationship between cIMT and cognitive function was assessed by means of generalized linear and longitudinal models, adjusted for relevant covariates. Mediation analysis was utilized to establish the proportion of the effect between increased cIMT and cognitive performance, which is mediated by age.

RESULTS: A total of 561 individuals were included for the cross-sectional study, and 510 of them were assessed for the prospective cohort. Univariate analysis showed a significant association between increased cIMT and worse cognitive performance (P < .001), which vanishes after considering the effect of age and low scholarity. Causal mediation analysis confirms that age captures 82.6% (95% C.I.: 63.9% to 100%) of the effect of this association. There was no relationship between increased cIMT and cognitive decline in the follow-up.

CONCLUSIONS: In this rural population, the association between increased cIMT and cognitive dysfunction is mostly mediated by increasing age.

Copyright © 2019 Elsevier Inc. All rights reserved.

Version ID

1

Record Owner

From MEDLINE, a database of the U.S. National Library of Medicine.

Status

MEDLINE

Authors Full Name

Del Brutto, Oscar H; Mera, Robertino M; Recalde, Bettsy Y; Del Brutto, Victor J.

Institution

Del Brutto, Oscar H. School of Medicine, Universidad Espiritu Santo - Ecuador, Samborondon, Ecuador. Electronic address: oscardelbrutto@hotmail.com. Mera, Robertino M. Department of Epidemiology, Gilead Sciences, Inc., Foster City, California.

Recalde, Bettsy Y. Community Center, the Atahualpa Project, Atahualpa, Ecuador.

Del Brutto, Victor J. Department of Neurology, Miller School of Medicine, University of Miami, Miami, Florida.

MeSH Heading

Adult. Age Factors. Aged. Aged, 80 and over. *Carotid Artery Diseases/dg [Diagnostic Imaging]. Carotid Artery Diseases/ep [Epidemiology]. *Carotid Intima-Media Thickness. *Cognition. *Cognitive Aging/px [Psychology]. Cognitive Dysfunction/di [Diagnosis]. Cognitive Dysfunction/ep [Epidemiology]. *Cognitive Dysfunction/px [Psychology]. Cross-Sectional Studies. Ecuador/ep [Epidemiology]. Female. Humans. Longitudinal Studies. Male. Middle Aged. Predictive Value of Tests. Prognosis. Prospective Studies. Risk Assessment. Risk Factors. *Rural Health. Time Factors.

Keyword Heading

For peer review only - http://bmjopen.bmj.com/site/about/guidelines.xhtml

Carotid intima-media thickness aging
cognitive decline
cognitive performance
extracranial carotid atherosclerosis
population study.
Year of Publication
2020

Link to the Ovid Full Text or citation:
Click here for full text options

Link to the External Link Resolver:
SFX

5.

Association Between Carotid Artery Intima-Media Thickness and Combinations of Mild Cognitive Impairment and Pre-Frailty in Older Adults.
Park J; Park JH; Park H.
International Journal of Environmental Research & Public Health [Electronic Resource]. 16(16), 2019 08 19.
[Journal Article. Research Support, Non-U.S. Gov't]
UI: 31430926
Carotid intima-media thickness (CIMT) has been proposed as a surrogate marker of cardiovascular disease. Mild cognitive impairment (MCI) and pre-frailty are reportedly associated with increased CIMT. As the evidence on the association of CIMT with combinations of MCI and pre-frailty is limited, this association is examined. A total of 231 older adults participated. MCI was defined according to clinical consensus or psychometric criteria by a dementia specialist, and considering detailed neuropsychological assessments. Also, pre-frailty was defined as subjects with frail component of 1 or 2. Carotid variables were measured using a B-mode ultrasound. The analysis of covariance (ANCOVA) was performed to assess independent differences in CIMT among the four groups, according to the cognitive function and frailty status after a multivariate adjustment. Increased CIMT is associated with combinations of MCI and pre-frailty. ANCOVA showed that

For peer review only - http://bmjopen.bmj.com/site/about/guidelines.xhtml
For peer review only

CIMTs were significantly different among the four groups according to the cognitive function and frailty status. CIMTmax combined with MCI and pre-frailty was the thickest (1.04 +/- 0.3 mm), whereas the CIMT of no MCI and no pre-frailty was the thinnest (0.82 +/- 0.2 mm). The results suggest that combinations of MCI and pre-frailty are associated with increased CIMT in older adults.

Version ID

1

Record Owner

From MEDLINE, a database of the U.S. National Library of Medicine.

Status

MEDLINE

Author NameID

Park, Jong-Hwan; ORCID: https://orcid.org/0000-0003-2815-7248 Park, Hyuntae; ORCID: https://orcid.org/0000-0002-1976-0005

Authors Full Name

Park, Jinkee; Park, Jong-Hwan; Park, Hyuntae.

Institution

Park, Jinkee. Department of Sport Rehabilitation, Dong-Ju College, Busan 49318, Korea. Park, Jong-Hwan. Health Convergence Medicine Research Group, Biomedical Research Institute, Pusan National University Hospital, 179, Gudeok-Ro, Seo-Gu, Busan 49241, Korea.

Park, Hyuntae. Department of Health Care Science, Dong-A University, Busan 49315, Korea. htpark@dau.ac.kr.

Park, Hyuntae. Institute of Convergence Bio-Health, Dong-A University, Busan 49201, Korea. htpark@dau.ac.kr.

MeSH Heading

Aged. Aged, 80 and over. *Carotid Intima-Media Thickness. *Cognitive Dysfunction/co [Complications]. Cognitive Dysfunction/di [Diagnosis]. Cognitive Dysfunction/pp [Physiopathology]. Cross-Sectional Studies. Female. *Frailty/co [Complications]. Frailty/pp [Physiopathology]. Frailty/px [Psychology]. Humans. Male. Neuropsychological Tests.

Keyword Heading

*cardiovascular disease *carotid intima-media thickness

*mild cognitive impairment

*older adults

*pre-frailty.

Year of Publication

2019

For peer review only

Link to the Ovid Full Text or citation:

Click here for full text options

Link to the External Link Resolver:

SFX

6.

Subclinical carotid atherosclerosis and neurocognitive function in an urban population.

Wendell CR; Waldstein SR; Evans MK; Zonderman AB.

Atherosclerosis. 249:125-31, 2016 06.

[Journal Article. Research Support, N.I.H., Extramural. Research Support, N.I.H., Intramural]

UI: 27092741

BACKGROUND AND AIMS: Examine age, sex, race, and socioeconomic status as modifiers of the association between carotid intimal medial thickness (IMT) and neurocognitive performance in a socioeconomically diverse, biracial, urban, adult population.

METHODS: Participants were 1712 community-dwelling adults (45% men, 56% African-American, 38% below poverty threshold, aged 30-64 years) enrolled in the Healthy Aging in Neighborhoods of Diversity across the Life Span (HANDLS) study. Participants underwent initial carotid ultrasonography followed by cognitive testing on up to two occasions over 4 years. Mixed-effects regression analyses were adjusted for demographic, behavioral, and biomedical covariates.

RESULTS: Significant cross-sectional IMT x race x poverty interactions were identified for measures of delayed recall memory, auditory-verbal attention, and working memory. An IMT x race interaction also appeared for auditory-verbal learning. Higher IMT was generally associated with worse cognitive performance, but the disadvantage was most pronounced among those with higher socioeconomic status and white participants. No longitudinal associations were identified.

CONCLUSIONS: Carotid IMT-cognition associations differed as a function of race and socioeconomic status and were most compelling for measures of attention, executive function, and memory. These findings highlight the possibility that subclinical atherosclerosis may be differentially informative as a predictor of cognitive performance among varied demographic subgroups.

Copyright © 2016 Elsevier Ireland Ltd. All rights reserved.

Version ID

For peer review only - http://bmjopen.bmj.com/site/about/guidelines.xhtml

For peer review only

1

Record Owner

From MEDLINE, a database of the U.S. National Library of Medicine.

Status

MEDLINE

Authors Full Name

Wendell, Carrington R; Waldstein, Shari R; Evans, Michele K; Zonderman, Alan B.

Institution

Wendell, Carrington R. Department of Psychology, University of Maryland, Baltimore County, USA; Laboratory of Epidemiology and Population Sciences, Intramural Research Program, National Institute on Aging, NIH, USA. Electronic address: crwendell@umbc.edu. Waldstein, Shari R. Department of Psychology, University of Maryland, Baltimore County, USA; Division of Gerontology & Geriatric Medicine, Department of Medicine, University of Maryland School of Medicine, USA; Geriatric Research Education and Clinical Center, Baltimore VA Medical Center, USA.

Evans, Michele K. Laboratory of Epidemiology and Population Sciences, Intramural Research Program, National Institute on Aging, NIH, USA.

Zonderman, Alan B. Laboratory of Epidemiology and Population Sciences, Intramural Research Program, National Institute on Aging, NIH, USA.

MeSH Heading

Adult. African Americans. Atherosclerosis/ep [Epidemiology]. Attention. Baltimore. Carotid Artery Diseases/co [Complications]. *Carotid Artery Diseases/di [Diagnosis]. Carotid Artery Diseases/eh [Ethnology]. Carotid Intima-Media Thickness. Cognition. Cognition Disorders/co [Complications]. *Cognition Disorders/di [Diagnosis]. Cognition Disorders/eh [Ethnology]. European Continental Ancestry Group. Female. Humans. Longitudinal Studies. Male. Memory. Middle Aged. Poverty. Prospective Studies. Regression Analysis. Social Class. *Urban Population.

Keyword Heading

*Carotid intimal medial thickness *Cognitive function

*Subclinical cardiovascular disease.

Year of Publication

2016

Link to the Ovid Full Text or citation:

Click here for full text options

Link to the External Link Resolver:

For peer review only - http://bmjopen.bmj.com/site/about/guidelines.xhtml

SFX

8.

Carotid artery plaque detected on ultrasound is associated with impaired cognitive state in the elderly: A population-based study in Wakiso district, Uganda.

Mworozi K; Ameda F; Byanyima RK; Nakasujja N.

Journal of Clinical Neuroscience. 68:194-200, 2019 Oct.

[Journal Article]

UI: 31301929

Carotid artery disease which includes carotid artery stenosis, plaques, clots and increased intima media thickness, have been reported by many studies to be associated with dementia. Dementia is an end stage of usually asymptomatic cognitive impairment. Risk factors of carotid artery disease include; age, atherosclerosis, arteriosclerosis, shorter years in school, history of hypertension, diabetes mellitus, stroke and depression. This study set out to determine the prevalence of abnormal carotid ultrasound findings and their association with cognitive function among the adults >=60years in Wakiso district, Uganda in 2018. A total of 210 participants were included. Carotid artery stenosis, presence of plaque, stenosis and intima-media thickness were assessed by ultrasound. Cognitive status was assessed using a Mini Mental State Exam (MMSE) test. The prevalence of plaque was 21.4%. Variables which included; presence of plaque, age, education, gender, marital status, whether participant stayed alone or with someone else, care for self, occupation status, division of staying and history of smoking. The presence of plaque was associated with an abnormal cognitive function at both univariate and multivariate analysis with respective OR=3.8 (95% CI=1.90-7.54, p-value=0.0001) and OR=3.4 (95% CI=1.38-8.15, p-value=0.007). The cognitive function distribution was 43.8%, 19%, 34.3% and 2.9% within the normal, mild, moderate, and severe cognitive function status respectively. This study showed that prevalence of carotid artery plaque was high in this elderly population in Wakiso district Uganda. Also, carotid artery plaque was associated with abnormal cognitive function.

Copyright © 2019 Elsevier Ltd. All rights reserved.

Version ID

1

Record Owner

From MEDLINE, a database of the U.S. National Library of Medicine.

Status

For peer review only - http://bmjopen.bmj.com/site/about/guidelines.xhtml

MEDLINE

Authors Full Name

Mworozi, Kenneth; Ameda, Faith; Byanyima, Rosemary K; Nakasujja, Noeline.

Institution

Mworozi, Kenneth. Department of Radiology, School of Medicine, P.O. Box 7062, Kampala, Uganda. Electronic address: mworozikenneth@gmail.com. Ameda, Faith. Department of Radiology, Makerere University College of Health Sciences, P.O. Box 7062, Kampala, Uganda. Byanyima, Rosemary K. Department of Radiology, Mulago National Referral Hospital, P.O. Box 7072, Kampala, Uganda.

Nakasujja, Noeline. Department of Psychiatry, Makerere University College of Health Sciences, P.O. Box 7062, Kampala, Uganda.

MeSH Heading

Aged. Carotid Intima-Media Thickness/px [Psychology]. *Carotid Stenosis/co [Complications]. Carotid Stenosis/dg [Diagnostic Imaging]. *Carotid Stenosis/ep [Epidemiology]. *Cognitive Dysfunction/et [Etiology]. Female. Humans. Male. Middle Aged. Multivariate Analysis. *Plaque, Atherosclerotic/co [Complications]. Plaque, Atherosclerotic/dg [Diagnostic Imaging]. *Plaque, Atherosclerotic/ep [Epidemiology]. Prevalence. Risk Factors. Uganda/ep [Epidemiology]. Ultrasonography.

Keyword Heading

Abnormal cognitive function Carotid artery disease

Carotid artery plaque

Carotid ultrasound

Elderly

Intima-media thickness

Mini-mental state examination.

Year of Publication

2019

Link to the Ovid Full Text or citation:

Click here for full text options

Link to the External Link Resolver:

SFX

For peer review only

10.

Common Carotid Artery Calcification Impacts on Cognitive Function in Older Patients.

Di Daniele N; Celotto R; Alunni Fegatelli D; Gabriele M; Rovella V; Scuteri A.

High Blood Pressure & Cardiovascular Prevention. 26(2):127-134, 2019 Apr.

[Journal Article]

UI: 30779026

INTRODUCTION: Cognitive impairment and dementia represent an emerging health problem. Cardiovascular (CV) risk factors contribute to cognitive impairment.

AIM: To investigate the effect of vascular calcification on cognitive impairment and dementia, independently of plaque and traditional CV risk factors.

METHODS: Four hundred and sixty-nine patients (age of 78.6 +/- 6.1 years, 74.4% women) were studied. Traditional CV risk factors levels, cognitive function (MMSE), brain CT scan, and other vascular parameters were measured. Common Carotid Artery (CCA) plaque and calcification were evaluated by ultrasound.

RESULTS: CCA calcification was associated with a lower MMSE score than in subjects with no CCA calcification (23.7 +/- 0.3 versus 25.5 +/- 0.8; p = 0.015), after controlling for age, sex, education, blood pressure levels, diabetes, creatinine, lipid lowering therapy, neuroimaging alteration, and CCA plaque. Similarly, CCA calcification was associated with higher odds of dementia regardless of the presence of CCA plaque (OR 1.70, 95% CI 1.01-2.94, p < 0.05). This trend was not observed when stratifying patients according to the presence of CCA plaque.

CONCLUSION: CCA calcification is associated with cognitive impairment and dementia, independently of established CV risk factors and CCA plaque. The impact of arterial calcification on cognition seems largely independent of arterial stiffness.

Version ID

1

Record Owner

From MEDLINE, a database of the U.S. National Library of Medicine.

Status

MEDLINE

Authors Full Name

Di Daniele, Nicola; Celotto, Roberto; Alunni Fegatelli, Danilo; Gabriele, Marco; Rovella, Valentina; Scuteri, Angelo.

Institution

Di Daniele, Nicola. Hypertension and Nephrology Unit, Department of Medicine, Policinico Tor Vergata, Universita'di Roma Tor Vergata, Rome, Italy.   Celotto, Roberto. Hypertension and

For peer review only - http://bmjopen.bmj.com/site/about/guidelines.xhtml

Nephrology Unit, Department of Medicine, Policinico Tor Vergata, Universita'di Roma Tor Vergata, Rome, Italy.
Alunni Fegatelli, Danilo. Department of Public Health and Infectious Disease, University "La Sapienza", Rome, Italy.
Gabriele, Marco. Hypertension and Nephrology Unit, Department of Medicine, Policinico Tor Vergata, Universita'di Roma Tor Vergata, Rome, Italy.
Rovella, Valentina. Hypertension and Nephrology Unit, Department of Medicine, Policinico Tor Vergata, Universita'di Roma Tor Vergata, Rome, Italy.
Scuteri, Angelo. Department of Medical, Surgical, and Experimental Sciences, University of Sassari, Sassari, Italy. d341elefante@virgilio.it.

MeSH Heading

Age Factors. Aged. Aged, 80 and over. *Carotid Artery Diseases/co [Complications]. Carotid Artery Diseases/dg [Diagnostic Imaging]. Carotid Artery Diseases/pp [Physiopathology]. Carotid Artery, Common/dg [Diagnostic Imaging]. Carotid Artery, Common/pp [Physiopathology]. *Carotid Artery, Common. *Cognition. Cognition Disorders/di [Diagnosis]. *Cognition Disorders/et [Etiology]. Cognition Disorders/px [Psychology]. *Cognitive Aging. Dementia/di [Diagnosis]. *Dementia/et [Etiology]. Dementia/px [Psychology]. Female. Humans. Male. Mental Status and Dementia Tests. Plaque, Atherosclerotic. Pulse Wave Analysis. Risk Factors. Tomography, X-Ray Computed. Ultrasonography. *Vascular Calcification/co [Complications]. Vascular Calcification/dg [Diagnostic Imaging]. Vascular Calcification/pp [Physiopathology]. Vascular Stiffness.

Keyword Heading

Arterial stiffness Cognitive impairment

Dementia

Vascular aging

Vascular calcification.

Year of Publication

2019

Link to the Ovid Full Text or citation:

Click here for full text options

Link to the External Link Resolver:

SFX

For peer review only - http://bmjopen.bmj.com/site/about/guidelines.xhtml

11.

Cortical Cerebral Microinfarcts on 3T Magnetic Resonance Imaging in Patients With Carotid Artery Stenosis.

Takasugi J; Miwa K; Watanabe Y; Okazaki S; Todo K; Sasaki T; Sakaguchi M; Mochizuki H.

Stroke. 50(3):639-644, 2019 03.

[Journal Article. Research Support, Non-U.S. Gov't]

UI: 30744544

Background and Purpose - Carotid artery stenosis is common in the elderly and contributes to cognitive impairment and dementia. Cortical cerebral microinfarcts (CMIs) play an important role in vascular cognitive impairment and dementia. We aimed to investigate the association between CMIs on 3T magnetic resonance imaging and clinical and radiological features, including plaque morphology, and cognitive function in patients with carotid stenosis. Methods- Eighty-nine patients with >30% carotid stenosis on ultrasound were prospectively enrolled, and underwent brain and carotid artery magnetic resonance imaging. CMIs were rated according to predetermined criteria based on 3D-double inversion recovery and fluid-attenuated inversion recovery images. Results - CMIs were identified in 26 patients (29%; median number 0, range 0-9). Poisson regression models adjusted for age and sex revealed that CMIs were associated with intraplaque hemorrhage (rate ratio, 1.95; 95% CI, 1.26-3.18), lacunar infarcts (rate ratio, 1.54; 95% CI, 1.00-2.44), and cortical infarcts (rate ratio, 3.22; 95% CI, 2.20-5.00). These associations were also observed in asymptomatic patients (n=64). Of 81 patients with unilateral carotid stenosis, the prevalence and number of CMIs were significantly higher in the hemisphere ipsilateral to the carotid stenosis than in the contralateral hemisphere (P=0.005 and P<0.001, respectively). The presence of CMIs was associated with poor cognitive function. Conclusions - Our results indicate that vulnerable carotid plaque increases the risk of CMIs and subsequent cognitive impairment. Carotid atherosclerosis could be a potential therapeutic target for cognitive impairment.

Version ID

1

Record Owner

From MEDLINE, a database of the U.S. National Library of Medicine.

Status

MEDLINE

Authors Full Name

Takasugi, Junji; Miwa, Kaori; Watanabe, Yoshiyuki; Okazaki, Shuhei; Todo, Kenichi; Sasaki, Tsutomu; Sakaguchi, Manabu; Mochizuki, Hideki.

Institution

For peer review only - http://bmjopen.bmj.com/site/about/guidelines.xhtml

Takasugi, Junji. From the Department of Neurology (J.T., K.M., S.O., K.T., T.S., M.S., H.M.), Osaka University Graduate School of Medicine, Japan. Miwa, Kaori. From the Department of Neurology (J.T., K.M., S.O., K.T., T.S., M.S., H.M.), Osaka University Graduate School of Medicine, Japan.
Miwa, Kaori. Department of Cerebrovascular Medicine, National Cerebral and Cardiovascular Center, Osaka, Japan (K.W.).
Watanabe, Yoshiyuki. Department of Diagnostic and Interventional Radiology (Y.W.), Osaka University Graduate School of Medicine, Japan.
Okazaki, Shuhei. From the Department of Neurology (J.T., K.M., S.O., K.T., T.S., M.S., H.M.), Osaka University Graduate School of Medicine, Japan.
Todo, Kenichi. From the Department of Neurology (J.T., K.M., S.O., K.T., T.S., M.S., H.M.), Osaka University Graduate School of Medicine, Japan.
Sasaki, Tsutomu. From the Department of Neurology (J.T., K.M., S.O., K.T., T.S., M.S., H.M.), Osaka University Graduate School of Medicine, Japan.
Sakaguchi, Manabu. From the Department of Neurology (J.T., K.M., S.O., K.T., T.S., M.S., H.M.), Osaka University Graduate School of Medicine, Japan.
Mochizuki, Hideki. From the Department of Neurology (J.T., K.M., S.O., K.T., T.S., M.S., H.M.), Osaka University Graduate School of Medicine, Japan.

MeSH Heading

Aged. Aged, 80 and over. *Carotid Stenosis/dg [Diagnostic Imaging]. Carotid Stenosis/ep [Epidemiology]. Carotid Stenosis/px [Psychology]. *Cerebral Cortex/dg [Diagnostic Imaging]. *Cerebral Infarction/dg [Diagnostic Imaging]. Cerebral Infarction/ep [Epidemiology]. Cerebral Infarction/px [Psychology]. Cognition. Female. Humans. Image Processing, Computer-Assisted. Intracranial Arteriosclerosis/dg [Diagnostic Imaging]. Intracranial Arteriosclerosis/ep [Epidemiology]. Magnetic Resonance Imaging. Male. Mental Status and Dementia Tests. Neuropsychological Tests. Prevalence. Prospective Studies. Ultrasonography.

Keyword Heading

*carotid stenosis *cognition
*dementia
*embolism
*magnetic resonance imaging.

Year of Publication

2019

Link to the Ovid Full Text or citation:

[Click here for full text options]

For peer review only

Link to the External Link Resolver:

SFX

12.

Healthy Lifestyle During the Midlife Is Prospectively Associated With Less Subclinical Carotid Atherosclerosis: The Study of Women's Health Across the Nation.

Wang D; Jackson EA; Karvonen-Gutierrez CA; Elliott MR; Harlow SD; Hood MM; Derby CA; Sternfeld B; Janssen I; Crawford SL; Huang MH; El Khoudary SR; Chae CU; Baylin A.

Journal of the American Heart Association. 7(23):e010405, 2018 12 04.

[Journal Article. Research Support, N.I.H., Extramural]

UI: 30482079

Background Measures of subclinical atherosclerosis are predictors of future cardiovascular outcomes as well as of physical and cognitive functioning. The menopausal transition is associated with accelerated progression of atherosclerosis in women. The prospective association between a healthy lifestyle during the midlife and subclinical atherosclerosis is unclear. Methods and Results Self-reported data on smoking, diet, and physical activity from 1143 women in the Study of Women's Health Across the Nation were used to construct a 10-year average Healthy Lifestyle Score (HLS) during the midlife. Markers of subclinical atherosclerosis were measured 14 years after baseline and included common carotid artery intima-media thickness (CCA - IMT), adventitial diameter (CCA - AD), and carotid plaque. The associations of average HLS with CCA - IMT and CCA - AD were estimated using linear models; the association of average HLS with carotid plaque was estimated using cumulative logit models. Average HLS was associated with smaller CCA - IMT and CCA - AD in the fully adjusted models (P=0.0031 and <0.001, respectively). Compared with participants in the lowest HLS level, those in the highest level had 0.024 mm smaller CCA - IMT (95% confidence interval: -0.048, 0.000), which equals 17% of the SD of CCA - IMT , and 0.16 mm smaller CCA - AD (95% confidence interval: -0.27, -0.04), which equals 24% of the SD of CCA - AD . Among the 3 components of the HLS , abstinence from smoking had the strongest association with subclinical atherosclerosis. Conclusions Healthy lifestyle during the menopausal transition is associated with less subclinical atherosclerosis, highlighting the growing recognition that the midlife is a critical window for cardiovascular prevention in women.

Version ID

1

Record Owner

For peer review only - http://bmjopen.bmj.com/site/about/guidelines.xhtml

From MEDLINE, a database of the U.S. National Library of Medicine.

Status

MEDLINE

Authors Full Name

Wang, Dongqing; Jackson, Elizabeth A; Karvonen-Gutierrez, Carrie A; Elliott, Michael R; Harlow, Sioban D; Hood, Michelle M; Derby, Carol A; Sternfeld, Barbara; Janssen, Imke; Crawford, Sybil L; Huang, Mei-Hua; El Khoudary, Samar R; Chae, Claudia U; Baylin, Ana.

Institution

Wang, Dongqing. 1 Department of Epidemiology University of Michigan Ann Arbor MI. Jackson, Elizabeth A. 2 Division of Cardiovascular Disease University of Alabama at Birmingham AL.
Karvonen-Gutierrez, Carrie A. 1 Department of Epidemiology University of Michigan Ann Arbor MI.
Elliott, Michael R. 3 Department of Biostatistics University of Michigan Ann Arbor MI.
Elliott, Michael R. 4 Survey Research Center University of Michigan Ann Arbor MI.
Harlow, Sioban D. 1 Department of Epidemiology University of Michigan Ann Arbor MI.
Hood, Michelle M. 1 Department of Epidemiology University of Michigan Ann Arbor MI.
Derby, Carol A. 5 Department of Neurology Albert Einstein College of Medicine Bronx NY.
Derby, Carol A. 6 Department of Epidemiology and Population Health Albert Einstein College of Medicine Bronx NY.
Sternfeld, Barbara. 7 Division of Research Kaiser Permanente Oakland CA.
Janssen, Imke. 8 Department of Preventive Medicine Rush University Medical Center Chicago IL.
Crawford, Sybil L. 9 Medical School University of Massachusetts Worcester MA.
Huang, Mei-Hua. 10 Division of Geriatrics University of California Los Angeles Los Angeles CA.
El Khoudary, Samar R. 11 Department of Epidemiology University of Pittsburgh PA.
Chae, Claudia U. 12 Cardiology Division Massachusetts General Hospital Boston MA.
Baylin, Ana. 1 Department of Epidemiology University of Michigan Ann Arbor MI.
Baylin, Ana. 13 Department of Nutritional Sciences University of Michigan Ann Arbor MI.

Comments

Erratum in (EIN)

MeSH Heading

Asymptomatic Diseases. Carotid Artery Diseases/ep [Epidemiology]. Carotid Artery Diseases/et [Etiology]. *Carotid Artery Diseases/pc [Prevention & Control]. Diet/ae [Adverse Effects]. Exercise. Female. Healthy Lifestyle/ph [Physiology]. *Healthy Lifestyle. Humans. Middle Aged. Prospective Studies. Risk Factors. Smoking/ae [Adverse Effects]. United States/ep [Epidemiology].

Keyword Heading

*atherosclerosis *cardiovascular disease

*lifestyle

*risk factors

For peer review only

*women.

Year of Publication

2018

Link to the Ovid Full Text or citation:

Click here for full text options

Link to the External Link Resolver:

SFX

13.

Pulse Wave Velocity Is Associated With Greater Risk of Dementia in Mild Cognitive Impairment Patients.

Rouch L; Cestac P; Sallerin B; Andrieu S; Bailly H; Beunardeau M; Cohen A; Dubail D; Hernandorena I; Seux ML; Vidal JS; Hanon O.

Hypertension. 72(5):1109-1116, 2018 11.

[Journal Article]

UI: 30354804

To investigate the association between pulse wave velocity, intima-media thickness, carotid artery diameter, carotid plaques, and conversion from mild cognitive impairment to dementia. Three hundred and seventy-five elderly ambulatory subjects with mild cognitive impairment were followed yearly to examine potential conversion to dementia. Vascular function was assessed by carotid-femoral pulse wave velocity. Vascular structure was evaluated by intima-media thickness, carotid artery diameter, and carotid plaques using an ultrasonographic assessment of carotid arteries. One hundred and five patients (28%) converted to dementia during a mean follow-up period of 4.5 years. Higher pulse wave velocity was associated with greater risk of conversion to dementia (1-SD increase of pulse wave velocity: hazard ratio, 1.33; 95% CI, 1.04-1.71; P=0.02) independently of age, sex, educational level, systolic blood pressure, cardiovascular diseases, body mass index, calcium channel blockers intake, Mini-Mental State Examination at baseline, and apoE epsilon4 status. Intima-media thickness, carotid plaques, and carotid artery diameter did not predict conversion to dementia (1-SD increase of intima-media thickness: hazard ratio, 0.93; 95% CI, 0.73-1.18; P=0.55; presence of carotid plaques: hazard ratio, 1.08; 95% CI, 0.62-1.87; P=0.79; 1-

For peer review only - http://bmjopen.bmj.com/site/about/guidelines.xhtml

SD increase of carotid artery diameter: hazard ratio, 1.08; 95% CI, 0.89-1.31; P=0.44). Pulse wave velocity was associated with conversion to dementia, whereas intima-media thickness, carotid plaques, or carotid artery diameter were not after controlling for age and other confounding factors. Arterial stiffness could identify mild cognitive impairment patients at higher risk of dementia and may be a therapeutic target to delay or prevent the onset of dementia.

Version ID

1

Record Owner

From MEDLINE, a database of the U.S. National Library of Medicine.

Status

MEDLINE

Authors Full Name

Rouch, Laure; Cestac, Philippe; Sallerin, Brigitte; Andrieu, Sandrine; Bailly, Henri; Beunardeau, Maelle; Cohen, Adrien; Dubail, Delphine; Hernandorena, Intza; Seux, Marie-Laure; Vidal, Jean-Sebastien; Hanon, Olivier.

Institution

Rouch, Laure. From the EA 4468, Universite Paris Descartes, Sorbonne Paris Cite, France (L.R., H.B., M.B., A.C., D.D., I.H., M.-L.S., J.-S.V., O.H.). Cestac, Philippe. Unite INSERM 1027, Toulouse, France (P.C., S.A.).

Cestac, Philippe. University Paul Sabatier Toulouse III, France (P.C., B.S., S.A.).

Cestac, Philippe. Pole Pharmacie, Centre Hospitalier Universitaire de Toulouse, France (P.C., B.S.).

Sallerin, Brigitte. University Paul Sabatier Toulouse III, France (P.C., B.S., S.A.).

Sallerin, Brigitte. Pole Pharmacie, Centre Hospitalier Universitaire de Toulouse, France (P.C., B.S.).

Sallerin, Brigitte. Unite INSERM 1048, Toulouse, France (B.S.).

Andrieu, Sandrine. Unite INSERM 1027, Toulouse, France (P.C., S.A.).

Andrieu, Sandrine. University Paul Sabatier Toulouse III, France (P.C., B.S., S.A.).

Andrieu, Sandrine. Unite INSERM 1048, Toulouse, France (B.S.).

Andrieu, Sandrine. Pole Epidemiologie et Sante Publique, Centre Hospitalier Universitaire de Toulouse, France (S.A.).

Bailly, Henri. From the EA 4468, Universite Paris Descartes, Sorbonne Paris Cite, France (L.R., H.B., M.B., A.C., D.D., I.H., M.-L.S., J.-S.V., O.H.).

Bailly, Henri. Service de geriatrie, Hopital Broca, AP-HP, Hopitaux Universitaires Paris Centre, France (H.B., M.B., A.C., D.D., I.H., M.-L.S., J.-S.V., O.H.).

Beunardeau, Maelle. From the EA 4468, Universite Paris Descartes, Sorbonne Paris Cite, France (L.R., H.B., M.B., A.C., D.D., I.H., M.-L.S., J.-S.V., O.H.).

Beunardeau, Maelle. Service de geriatrie, Hopital Broca, AP-HP, Hopitaux Universitaires Paris Centre, France (H.B., M.B., A.C., D.D., I.H., M.-L.S., J.-S.V., O.H.).

For peer review only

Cohen, Adrien. From the EA 4468, Universite Paris Descartes, Sorbonne Paris Cite, France (L.R., H.B., M.B., A.C., D.D., I.H., M.-L.S., J.-S.V., O.H.).

Cohen, Adrien. Service de geriatrie, Hopital Broca, AP-HP, Hopitaux Universitaires Paris Centre, France (H.B., M.B., A.C., D.D., I.H., M.-L.S., J.-S.V., O.H.).

Dubail, Delphine. From the EA 4468, Universite Paris Descartes, Sorbonne Paris Cite, France (L.R., H.B., M.B., A.C., D.D., I.H., M.-L.S., J.-S.V., O.H.).

Dubail, Delphine. Service de geriatrie, Hopital Broca, AP-HP, Hopitaux Universitaires Paris Centre, France (H.B., M.B., A.C., D.D., I.H., M.-L.S., J.-S.V., O.H.).

Hernandorena, Intza. From the EA 4468, Universite Paris Descartes, Sorbonne Paris Cite, France (L.R., H.B., M.B., A.C., D.D., I.H., M.-L.S., J.-S.V., O.H.).

Hernandorena, Intza. Service de geriatrie, Hopital Broca, AP-HP, Hopitaux Universitaires Paris Centre, France (H.B., M.B., A.C., D.D., I.H., M.-L.S., J.-S.V., O.H.).

Seux, Marie-Laure. From the EA 4468, Universite Paris Descartes, Sorbonne Paris Cite, France (L.R., H.B., M.B., A.C., D.D., I.H., M.-L.S., J.-S.V., O.H.).

Seux, Marie-Laure. Service de geriatrie, Hopital Broca, AP-HP, Hopitaux Universitaires Paris Centre, France (H.B., M.B., A.C., D.D., I.H., M.-L.S., J.-S.V., O.H.).

Vidal, Jean-Sebastien. From the EA 4468, Universite Paris Descartes, Sorbonne Paris Cite, France (L.R., H.B., M.B., A.C., D.D., I.H., M.-L.S., J.-S.V., O.H.).

Vidal, Jean-Sebastien. Service de geriatrie, Hopital Broca, AP-HP, Hopitaux Universitaires Paris Centre, France (H.B., M.B., A.C., D.D., I.H., M.-L.S., J.-S.V., O.H.).

Hanon, Olivier. From the EA 4468, Universite Paris Descartes, Sorbonne Paris Cite, France (L.R., H.B., M.B., A.C., D.D., I.H., M.-L.S., J.-S.V., O.H.).

Hanon, Olivier. Service de geriatrie, Hopital Broca, AP-HP, Hopitaux Universitaires Paris Centre, France (H.B., M.B., A.C., D.D., I.H., M.-L.S., J.-S.V., O.H.).

Comments

Comment in (CIN)

MeSH Heading

Aged. Aged, 80 and over. *Blood Flow Velocity/ph [Physiology]. *Carotid Arteries/pp [Physiopathology]. Carotid Intima-Media Thickness. Cognitive Dysfunction/co [Complications]. *Cognitive Dysfunction/pp [Physiopathology]. Dementia/co [Complications]. *Dementia/pp [Physiopathology]. Female. Humans. Male. Middle Aged. Pulse Wave Analysis. Ultrasonography.

Keyword Heading

*blood pressure *dementia

*patients

*pulse wave analysis

*risk

*vascular stiffness.

For peer review only

Year of Publication
2018

Link to the Ovid Full Text or citation:
Click here for full text options

Link to the External Link Resolver:
SFX

15.

Association between carotid atheroma and cerebral cortex structure at age 73 years.
Alhusaini S; Karama S; Nguyen TV; Thiel A; Bernhardt BC; Cox SR; Corley J; Taylor A; Evans AC; Star JM; Bastin ME; Wardlaw JM; Deary IJ; Ducharme S.
Annals of Neurology. 84(4):576-587, 2018 10.
[Journal Article. Research Support, Non-U.S. Gov't]
UI: 30179274
OBJECTIVE: To examine the relationship between carotid atherosclerosis and cerebral cortical thickness and investigate whether cortical thickness mediates the association between carotid atheroma and relative cognitive decline.
METHODS: We assessed 554 community-dwelling subjects (male/female: 296/258) from the Lothian Birth Cohort 1936 who underwent brain magnetic resonance imaging and carotid Doppler ultrasound studies at age 73 years. The relationship between carotid atherosclerosis markers (internal carotid artery stenosis, intima-media thickness, velocity, pulsatility, and resistivity indexes) and vertex-wide cerebral cortical thickness was examined cross-sectionally, controlling for gender, extensive vascular risk factors (VRFs), and intelligence quotient at age 11 (IQ-11). We also determined the association between carotid stenosis and a composite measure of fluid intelligence at age 73 years. A mediation model was applied to examine whether cortical thickness mediated the relationship between carotid stenosis and cognitive function.
RESULTS: A widespread negative association was identified between carotid stenosis (median = 15%) and cerebral cortical thickness at age 73 years, independent of the side of carotid stenosis, other carotid measures, VRFs, and IQ-11. This association increased in an almost dose-response relationship from mild to severe degrees of carotid stenosis, across the anterior and posterior

For peer review only - http://bmjopen.bmj.com/site/about/guidelines.xhtml

circulation territories. A negative association was also noted between carotid stenosis and fluid intelligence (standardized beta coefficient = -0.151, p = 0.001), which appeared partly (approximately 22%) mediated by carotid stenosis-related thinning of the cerebral cortex. INTERPRETATION: The findings suggest that carotid stenosis represents a marker of processes that accelerate aging of the cerebral cortex and cognition that is in part independent of measurable VRFs. Cortical thinning within the anterior and posterior circulation territories partially mediated the relationship between carotid atheroma and fluid intelligence. Ann Neurol 2018;84:576-587.
Copyright © 2018 The Authors. Annals of Neurology published by Wiley Periodicals, Inc. on behalf of American Neurological Association.

Version ID

1

Record Owner

From MEDLINE, a database of the U.S. National Library of Medicine.

Status

MEDLINE

Author NameID

Wardlaw, Joanna M; ORCID: https://orcid.org/0000-0002-9812-6642

Authors Full Name

Alhusaini, Saud; Karama, Sherif; Nguyen, Tuong-Vi; Thiel, Alexander; Bernhardt, Boris C; Cox, Simon R; Corley, Janie; Taylor, Adele; Evans, Alan C; Star, John M; Bastin, Mark E; Wardlaw, Joanna M; Deary, Ian J; Ducharme, Simon.

Institution

Alhusaini, Saud. Department of Neurology and Neurosurgery, Montreal Neurological Institute and Hospital, McGill University, Montreal, Quebec, Canada. Karama, Sherif. McConnell Brain Imaging Centre, Montreal Neurological Institute, McGill University, Montreal, Quebec, Canada.

Karama, Sherif. Department of Psychiatry, Douglas Mental Health University Institute, McGill University, Montreal, Quebec, Canada.

Nguyen, Tuong-Vi. Department of Psychiatry, McGill University Health Centre, McGill University, Montreal, Quebec, Canada.

Nguyen, Tuong-Vi. Department of Obstetrics-Gynecology, McGill University Health Centre, McGill University, Montreal, Quebec, Canada.

Thiel, Alexander. Department of Neurology and Neurosurgery, Montreal Neurological Institute and Hospital, McGill University, Montreal, Quebec, Canada.

Thiel, Alexander. Department of Neurology, Jewish General Hospital, Lady Davis Institute for Medical Research, Montreal, Quebec, Canada.

Bernhardt, Boris C. McConnell Brain Imaging Centre, Montreal Neurological Institute, McGill University, Montreal, Quebec, Canada.

For peer review only - http://bmjopen.bmj.com/site/about/guidelines.xhtml

Cox, Simon R. Centre for Cognitive Ageing and Cognitive Epidemiology, Department of Psychology, University of Edinburgh, Edinburgh, United Kingdom.
Corley, Janie. Centre for Cognitive Ageing and Cognitive Epidemiology, Department of Psychology, University of Edinburgh, Edinburgh, United Kingdom.
Taylor, Adele. Centre for Cognitive Ageing and Cognitive Epidemiology, Department of Psychology, University of Edinburgh, Edinburgh, United Kingdom.
Evans, Alan C. McConnell Brain Imaging Centre, Montreal Neurological Institute, McGill University, Montreal, Quebec, Canada.
Star, John M. Centre for Cognitive Ageing and Cognitive Epidemiology, Department of Psychology, University of Edinburgh, Edinburgh, United Kingdom.
Star, John M. Alzheimer Scotland Dementia Research Centre, Department of Psychology, University of Edinburgh, Edinburgh, United Kingdom.
Bastin, Mark E. Centre for Cognitive Ageing and Cognitive Epidemiology, Department of Psychology, University of Edinburgh, Edinburgh, United Kingdom.
Bastin, Mark E. Brain Research Imaging Centre, Centre for Clinical Brain Sciences, University of Edinburgh, Edinburgh, United Kingdom.
Wardlaw, Joanna M. Centre for Cognitive Ageing and Cognitive Epidemiology, Department of Psychology, University of Edinburgh, Edinburgh, United Kingdom.
Wardlaw, Joanna M. Brain Research Imaging Centre, Centre for Clinical Brain Sciences, University of Edinburgh, Edinburgh, United Kingdom.
Wardlaw, Joanna M. UK Dementia Research Institute at the University of Edinburgh, Edinburgh, United Kingdom.
Deary, Ian J. Centre for Cognitive Ageing and Cognitive Epidemiology, Department of Psychology, University of Edinburgh, Edinburgh, United Kingdom.
Deary, Ian J. Alzheimer Scotland Dementia Research Centre, Department of Psychology, University of Edinburgh, Edinburgh, United Kingdom.
Ducharme, Simon. Department of Neurology and Neurosurgery, Montreal Neurological Institute and Hospital, McGill University, Montreal, Quebec, Canada.
Ducharme, Simon. McConnell Brain Imaging Centre, Montreal Neurological Institute, McGill University, Montreal, Quebec, Canada.
Ducharme, Simon. Department of Psychiatry, McGill University Health Centre, McGill University, Montreal, Quebec, Canada.

MeSH Heading

Aged. *Carotid Artery Diseases/dg [Diagnostic Imaging]. *Carotid Artery Diseases/px [Psychology]. *Carotid Artery, Internal/dg [Diagnostic Imaging]. *Cerebral Cortex/dg [Diagnostic Imaging]. Cohort Studies. Cross-Sectional Studies. Female. Humans. Magnetic Resonance Imaging/td

For peer review only

[Trends]. Male. *Mental Status and Dementia Tests. Organ Size. Scotland/ep [Epidemiology]. Ultrasonography, Doppler/td [Trends].
Year of Publication
2018

Link to the Ovid Full Text or citation:
Click here for full text options

Link to the External Link Resolver:
SFX

17.

Carotid circumferential wall stress is not associated with cognitive performance among individuals in late middle age: The Maastricht Study.
Geijselaers SL; Sep SJ; Schram MT; van Boxtel MP; van Sloten TT; Op Het Roodt J; Henry RM; Reesink KD; Schaper NC; Dagnelie PC; van der Kallen CJ; Biessels GJ; Stehouwer CD.
Atherosclerosis. 276:15-22, 2018 09.
[Journal Article. Research Support, Non-U.S. Gov't]
UI: 30006323
BACKGROUND AND AIMS: Arterial remodelling aims at normalising circumferential wall stress (CWS). Greater CWS in the carotid artery has previously been associated with the prevalence and severity of cerebral small vessel disease, a major cause of ageing-related cognitive decline. Here we test the hypothesis that greater carotid CWS is associated with poorer cognitive performance. METHODS: We studied 722 individuals (60+/-8 years, 55% men, 42.5% highly educated, blood pressure 137+/-19/77+/-11mmHg, n=197 with type 2 diabetes) who completed a neuropsychological assessment and underwent vascular ultrasound to measure the intima-media thickness (IMT) and interadventitial diameter (IAD) of the left common carotid artery at a plaque-free site. From IMT and IAD, lumen diameter (LD) was calculated. These structural measures were then combined with local carotid pulse pressure and brachial mean arterial pressure to obtain a measure of pulsatile (CWSpulsatile) and average (CWSmean) mechanical load on the vessel wall. Cognitive domains assessed were memory, executive function and attention, and processing speed.

For peer review only

RESULTS: After adjustment for age, sex, and education, regression analyses showed that neither CWSpulsatile nor CWSmean were associated with measures of cognitive performance (p-values >=0.31). This null association did not differ by age or educational level, and was observed in both individuals with and without carotid plaque, diabetes and/or hypertension. In addition, none of the individual measures of carotid structure (i.e. IMT, IAD, and LD) was related to cognitive performance.

CONCLUSIONS: The present cross-sectional study shows that carotid CWS is not associated with cognitive performance, at least not among relatively highly educated individuals in late middle age with adequately controlled cardiovascular risk factors.

Copyright © 2018 The Authors. Published by Elsevier B.V. All rights reserved.

Version ID

1

Record Owner

From MEDLINE, a database of the U.S. National Library of Medicine.

Status

MEDLINE

Authors Full Name

Geijselaers, Stefan Lc; Sep, Simone Js; Schram, Miranda T; van Boxtel, Martin Pj; van Sloten, Thomas T; Op Het Roodt, Jos; Henry, Ronald Ma; Reesink, Koen D; Schaper, Nicolaas C; Dagnelie, Pieter C; van der Kallen, Carla Jh; Biessels, Geert Jan; Stehouwer, Coen DA.

Institution

Geijselaers, Stefan Lc. Department of Internal Medicine, Maastricht University Medical Centre +, Maastricht, the Netherlands; CARIM School for Cardiovascular Diseases, Maastricht University, Maastricht, the Netherlands; Department of Neurology, Brain Centre Rudolf Magnus, University Medical Centre Utrecht, Utrecht, the Netherlands. Electronic address: stefan.geijselaers@gmail.com. Sep, Simone Js. Department of Internal Medicine, Maastricht University Medical Centre +, Maastricht, the Netherlands; CARIM School for Cardiovascular Diseases, Maastricht University, Maastricht, the Netherlands.

Schram, Miranda T. Department of Internal Medicine, Maastricht University Medical Centre +, Maastricht, the Netherlands; CARIM School for Cardiovascular Diseases, Maastricht University, Maastricht, the Netherlands.

van Boxtel, Martin Pj. Department of Psychiatry and Neuropsychology and MHeNS School for Mental Health and Neuroscience, Maastricht University Medical Centre +, Maastricht, the Netherlands.

van Sloten, Thomas T. Department of Internal Medicine, Maastricht University Medical Centre +, Maastricht, the Netherlands; CARIM School for Cardiovascular Diseases, Maastricht University, Maastricht, the Netherlands.

For peer review only - http://bmjopen.bmj.com/site/about/guidelines.xhtml

Op Het Roodt, Jos. Department of Internal Medicine, Maastricht University Medical Centre +, Maastricht, the Netherlands; CARIM School for Cardiovascular Diseases, Maastricht University, Maastricht, the Netherlands.
Henry, Ronald Ma. Department of Internal Medicine, Maastricht University Medical Centre +, Maastricht, the Netherlands; CARIM School for Cardiovascular Diseases, Maastricht University, Maastricht, the Netherlands.
Reesink, Koen D. Department of Biomedical Engineering, Maastricht University Medical Centre +, Maastricht, the Netherlands.
Schaper, Nicolaas C. Department of Internal Medicine, Maastricht University Medical Centre +, Maastricht, the Netherlands; CARIM School for Cardiovascular Diseases, Maastricht University, Maastricht, the Netherlands; CAPHRI School for Public Health and Primary Care, Maastricht University, Maastricht, the Netherlands.
Dagnelie, Pieter C. CARIM School for Cardiovascular Diseases, Maastricht University, Maastricht, the Netherlands; CAPHRI School for Public Health and Primary Care, Maastricht University, Maastricht, the Netherlands; Department of Epidemiology, Maastricht University, Maastricht, the Netherlands.
van der Kallen, Carla Jh. Department of Internal Medicine, Maastricht University Medical Centre +, Maastricht, the Netherlands; CARIM School for Cardiovascular Diseases, Maastricht University, Maastricht, the Netherlands.
Biessels, Geert Jan. Department of Neurology, Brain Centre Rudolf Magnus, University Medical Centre Utrecht, Utrecht, the Netherlands.
Stehouwer, Coen DA. Department of Internal Medicine, Maastricht University Medical Centre +, Maastricht, the Netherlands; CARIM School for Cardiovascular Diseases, Maastricht University, Maastricht, the Netherlands.

MeSH Heading

Age Factors. Aged. Attention. Blood Pressure. Carotid Arteries/dg [Diagnostic Imaging]. *Carotid Arteries/pp [Physiopathology]. Carotid Artery Diseases/dg [Diagnostic Imaging]. *Carotid Artery Diseases/pp [Physiopathology]. *Carotid Artery Diseases/px [Psychology]. Carotid Intima-Media Thickness. *Cognition. Cross-Sectional Studies. Educational Status. Executive Function. Female. Humans. Male. Memory. Middle Aged. Netherlands. Neuropsychological Tests. Risk Factors. Stress, Mechanical. *Vascular Remodeling.

Year of Publication

2018

Link to the Ovid Full Text or citation:

Click here for full text options

For peer review only

Link to the External Link Resolver:

SFX

18.

Cognitive function of patients with rheumatoid arthritis is associated with disease activity but not carotid atherosclerotic changes.

Lee JH; Kim GT; Kim YK; Lee SG.

Clinical & Experimental Rheumatology. 36(5):856-861, 2018 Sep-Oct.

[Journal Article]

UI: 29652660

OBJECTIVES: Although the relationship between atherosclerosis and cognitive impairment has been studied and replicated, whether cognitive deficits in RA can be attributed to atherosclerotic changes is not well understood. This study investigated cognitive function in patients with RA and evaluated whether cognitive function was affected by carotid arterial atherosclerosis.

METHODS: We examined 70 RA patients and 40 healthy controls. RA activity was assessed by disease activity score with 28 joint-erythrocyte sedimentation rate (DAS28-ESR). Cognitive function was assessed by the Korean version of the Consortium to Establish a Registry for Alzheimer's disease (CERAD-K) neuropsychological battery. Carotid arteries were scanned for the presence of plaques and to assess intima-media thickness (IMT). We assessed potential risk factors of cognitive impairment in RA patients using regression analyses.

RESULTS: There was a significant difference between RA patients and healthy controls in the verbal fluency (p=0.004) and Boston naming test (p=0.035). Carotid ultrasound revealed significantly more plaque in RA patients than in healthy controls (p=0.017). RA patients with memory impairment had significantly higher DAS28-ESR scores (p<0.001), age (p=0.009), and mean cIMT (p=0.027) than RA patients without memory impairment. In multivariable regression analysis, CERAD-K total score showed a significant negative correlation with age (beta=-0.415, p<0.001) or DAS28-ESR (beta=-4.685, p<0.001), but no correlation was found between CERAD-K total score and presence of plaque or cIMT.

CONCLUSIONS: Our results indicate that disease activity of RA and aging contribute to cognitive dysfunction, but there was no association between cognitive function and carotid atherosclerotic changes in RA patients.

Version ID

For peer review only - http://bmjopen.bmj.com/site/about/guidelines.xhtml

1

Record Owner

From MEDLINE, a database of the U.S. National Library of Medicine.

Status

MEDLINE

Authors Full Name

Lee, Ji Hyun; Kim, Geun-Tae; Kim, Yun-Kyung; Lee, Seung-Geun.

Institution

Lee, Ji Hyun. Division of Rheumatology, Department of Internal Medicine, Maryknoll Medical Center, Busan, Republic of Korea.   Kim, Geun-Tae. Division of Rheumatology, Department of Internal Medicine, Kosin University College of Medicine, Busan, Republic of Korea. gtah@hanmail.net.

Kim, Yun-Kyung. Division of Rheumatology, Department of Internal Medicine, Kosin University College of Medicine, Busan, Republic of Korea.

Lee, Seung-Geun. Division of Rheumatology, Department of Internal Medicine, Pusan National University Hospital, Busan, Republic of Korea.

MeSH Heading

Age Factors. Aged. Aging/px [Psychology]. *Arthritis, Rheumatoid/co [Complications]. Arthritis, Rheumatoid/di [Diagnosis]. Arthritis, Rheumatoid/px [Psychology]. *Carotid Artery Diseases/co [Complications]. Carotid Artery Diseases/dg [Diagnostic Imaging]. Carotid Artery Diseases/px [Psychology]. Carotid Intima-Media Thickness. Case-Control Studies. *Cognition. Cognition Disorders/di [Diagnosis]. *Cognition Disorders/et [Etiology]. Cognition Disorders/px [Psychology]. Female. Humans. Male. Memory. Middle Aged. Neuropsychological Tests. Plaque, Atherosclerotic. Risk Factors. Verbal Behavior.

Year of Publication

2018

Link to the Ovid Full Text or citation:

Click here for full text options

Link to the External Link Resolver:

SFX

For peer review only

For peer review only - http://bmjopen.bmj.com/site/about/guidelines.xhtml

22.

Association of subclinical carotid atherosclerosis with immediate memory and other cognitive functions.

Matsumoto L; Suzuki K; Mizuno Y; Ohike Y; Ozeki A; Ono S; Takanashi M; Sawaki D; Suzuki T; Yamazaki T; Tsuji S; Iwata A.

Geriatrics & gerontology international. 18(1):65-71, 2018 Jan.

[Journal Article]

UI: 28776906

AIM: To clarify whether carotid atherosclerosis and its risk factors are associated with cognitive decline.

METHODS: We evaluated 206 individuals who visited our center for health screening. We carried out physical examinations, blood tests, intima-media thickness (IMT) measurement by carotid ultrasonography, brain magnetic resonance imaging scanning and cognitive function assessments. A total of 30 individuals, who had significant cerebrovascular lesions detected in magnetic resonance imaging scans, were excluded. To detect early cognitive decline, we defined "cognitive impairment (CI)" when a patient satisfied at least one of three criteria. These were Mini-Mental State Examination score <24, clock-drawing test score <4 coexisting with forgetfulness and Wechsler Memory Scale-revised delayed recall score below the normal range for the duration of education (>16 years of education: >=9, 10-15 years: >=5, 0-9 years: >=3).

RESULTS: Among 176 individuals, 27 were placed in the CI group. IMT was significantly higher in the CI group as compared with the non-CI group (mean +/- SD: 2.0 +/- 1.0 vs 1.7 +/- 0.7, P = 0018 by Student's t-test). Other atherosclerotic risk factors, such as blood pressure, low-density lipoprotein cholesterol, and hemoglobin A1c, were not significantly different between the two groups. In multivariate analysis, maximum IMT was associated with impaired immediate recall score on Wechsler Memory Scale-revised, independent of the presence of deep white matter hyperintensities on the magnetic resonance imaging scan.

CONCLUSIONS: Subclinical carotid atherosclerosis, defined as thickened IMT, could be a marker for early stages of CI, especially for immediate memory recall. The impairment is presumably caused by inducing cerebral microvascular dysfunction in the frontal lobe. Geriatr Gerontol Int 2018; 18: 65-71.

Copyright © 2017 Japan Geriatrics Society.

Version ID

1

Record Owner

From MEDLINE, a database of the U.S. National Library of Medicine.

Status

For peer review only - http://bmjopen.bmj.com/site/about/guidelines.xhtml

MEDLINE

Author NameID

Iwata, Atsushi; ORCID: http://orcid.org/0000-0001-7308-5314

Authors Full Name

Matsumoto, Lumine; Suzuki, Kazushi; Mizuno, Yoshiko; Ohike, Yumiko; Ozeki, Atsuko; Ono, Satoshi; Takanashi, Mikio; Sawaki, Daigo; Suzuki, Toru; Yamazaki, Tsutomu; Tsuji, Shoji; Iwata, Atsushi.

Institution

Matsumoto, Lumine. Department of Neurology, The University of Tokyo Hospital, Tokyo, Japan.

Matsumoto, Lumine. Center for Epidemiology and Preventive Medicine, The University of Tokyo Hospital, Tokyo, Japan.

Suzuki, Kazushi. Department of Neurology, The University of Tokyo Hospital, Tokyo, Japan.

Suzuki, Kazushi. Center for Epidemiology and Preventive Medicine, The University of Tokyo Hospital, Tokyo, Japan.

Mizuno, Yoshiko. Center for Epidemiology and Preventive Medicine, The University of Tokyo Hospital, Tokyo, Japan.

Ohike, Yumiko. Center for Epidemiology and Preventive Medicine, The University of Tokyo Hospital, Tokyo, Japan.

Ozeki, Atsuko. Center for Epidemiology and Preventive Medicine, The University of Tokyo Hospital, Tokyo, Japan.

Ono, Satoshi. Center for Epidemiology and Preventive Medicine, The University of Tokyo Hospital, Tokyo, Japan.

Takanashi, Mikio. Center for Epidemiology and Preventive Medicine, The University of Tokyo Hospital, Tokyo, Japan.

Sawaki, Daigo. Center for Epidemiology and Preventive Medicine, The University of Tokyo Hospital, Tokyo, Japan.

Suzuki, Toru. Center for Epidemiology and Preventive Medicine, The University of Tokyo Hospital, Tokyo, Japan.

Yamazaki, Tsutomu. Center for Epidemiology and Preventive Medicine, The University of Tokyo Hospital, Tokyo, Japan.

Tsuji, Shoji. Department of Neurology, The University of Tokyo Hospital, Tokyo, Japan.

Iwata, Atsushi. Department of Neurology, The University of Tokyo Hospital, Tokyo, Japan.

MeSH Heading

Carotid Artery Diseases/di [Diagnosis]. *Carotid Artery Diseases/px [Psychology]. *Carotid Intima-Media Thickness. Cognition Disorders/ep [Epidemiology]. Humans. Memory Disorders/ep [Epidemiology]. Memory, Short-Term. Risk Factors.

Keyword Heading

For peer review only

atherosclerosis cognitive function
immediate memory
intima-media thickness.
Year of Publication
2018

Link to the Ovid Full Text or citation:
Click here for full text options

Link to the External Link Resolver:
SFX

23.

Carotid atherosclerotic plaque instability and cognition determined by ultrasound-measured plaque strain in asymptomatic patients with significant stenosis.
Dempsey RJ; Varghese T; Jackson DC; Wang X; Meshram NH; Mitchell CC; Hermann BP; Johnson SC; Berman SE; Wilbrand SM.
Journal of Neurosurgery. 128(1):111-119, 2018 01.
[Clinical Trial. Journal Article. Research Support, N.I.H., Extramural]
UI: 28298048
OBJECTIVE This article describes the use of ultrasound measurements of physical strain within carotid atherosclerotic plaques as a measure of instability and the potential for vascular cognitive decline, microemboli, and white matter changes. METHODS Asymptomatic patients with significant (> 60%) carotid artery stenosis were studied for dynamic measures of plaque instability, presence of microemboli, white matter changes, and vascular cognitive decline in comparison with normative controls and premorbid state. RESULTS Although classically asymptomatic, these patients showed vascular cognitive decline. The degree of strain instability measured within the atherosclerotic plaque directly predicted vascular cognitive decline in these patients thought previously to be asymptomatic according to classic criteria. Furthermore, 26% of patients showed microemboli, and patients had twice as much white matter hyperintensity as controls. CONCLUSIONS These data show that physical measures of plaque instability are possible through interpretation of ultrasound strain data during pulsation, which may be more clinically relevant

For peer review only - http://bmjopen.bmj.com/site/about/guidelines.xhtml
For peer review only

than solely measuring degree of stenosis. The data also highlight the importance of understanding that the definition of symptoms should not be limited to motor, speech, and vision function but underscore the role of vascular cognitive decline in the pathophysiology of carotid atherosclerotic disease. Clinical trial registration no.: NCT02476396 (clinicaltrials.gov).

Version ID

1

Record Owner

From MEDLINE, a database of the U.S. National Library of Medicine.

Status

MEDLINE

Authors Full Name

Dempsey, Robert J; Varghese, Tomy; Jackson, Daren C; Wang, Xiao; Meshram, Nirvedh H; Mitchell, Carol C; Hermann, Bruce P; Johnson, Sterling C; Berman, Sara E; Wilbrand, Stephanie M.

Institution

Dempsey, Robert J. Departments of1Neurological Surgery and.   Varghese, Tomy. 2Medical Physics, University of Wisconsin School of Medicine and Public Health.

Jackson, Daren C. 3Wisconsin Surgical Outcomes Research Program, Department of Surgery, University of Wisconsin School of Medicine and Public Health, Madison, Wisconsin.

Wang, Xiao. 4Rutgers Cancer Institute of New Jersey, New Brunswick, New Jersey.

Meshram, Nirvedh H. 2Medical Physics, University of Wisconsin School of Medicine and Public Health.

Mitchell, Carol C. 5Department of Medicine, Cardiovascular Medicine Division.

Hermann, Bruce P. 6Department of Neurology, University of Wisconsin School of Medicine and Public Health; and.

Johnson, Sterling C. 7Alzheimer's Disease Research Center, University of Wisconsin School of Medicine and Public Health, Waisman Laboratory for Brain Injury and Behavior, University of Wisconsin-Madison & Geriatric Research Education & Clinical Center, William S. Middleton Veterans Hospital, Madison, Wisconsin.

Berman, Sara E. 7Alzheimer's Disease Research Center, University of Wisconsin School of Medicine and Public Health, Waisman Laboratory for Brain Injury and Behavior, University of Wisconsin-Madison & Geriatric Research Education & Clinical Center, William S. Middleton Veterans Hospital, Madison, Wisconsin.

Wilbrand, Stephanie M. Departments of1Neurological Surgery and.

Comments

Comment in (CIN)

MeSH Heading

For peer review only

Aged. Aged, 80 and over. Biomechanical Phenomena. *Carotid Stenosis/dg [Diagnostic Imaging]. *Carotid Stenosis/px [Psychology]. *Cognition. Cognitive Dysfunction/dg [Diagnostic Imaging]. Cognitive Dysfunction/px [Psychology]. Constriction, Pathologic/dg [Diagnostic Imaging]. Constriction, Pathologic/px [Psychology]. Dementia, Vascular/dg [Diagnostic Imaging]. Dementia, Vascular/px [Psychology]. Female. Humans. Image Interpretation, Computer-Assisted. Magnetic Resonance Imaging. Male. Middle Aged. Neuropsychological Tests. *Plaque, Atherosclerotic/dg [Diagnostic Imaging]. *Plaque, Atherosclerotic/px [Psychology]. Prodromal Symptoms. Severity of Illness Index. *Ultrasonography. White Matter/dg [Diagnostic Imaging].

Keyword Heading

*CA = carotid artery *CAS = CA stenosis

*HITS = high-intensity transient signal

*MCA = middle cerebral artery

*TCD = transcranial Doppler

*TIA = transient ischemic attack

*WMH = white matter hyperintensity

*carotid atherosclerosis

*plaque instability

*stroke

*ultrasound

*ultrasound strain

*vascular cognitive decline

*vascular disorders.

Year of Publication

2018

Link to the Ovid Full Text or citation:

Click here for full text options

Link to the External Link Resolver:

SFX

24.

For peer review only - http://bmjopen.bmj.com/site/about/guidelines.xhtml

For peer review only

Carotid Atherosclerosis and Cognitive Impairment in Nonstroke Patients. [Review]

Chen WH; Jin W; Lyu PY; Liu Y; Li R; Hu M; Xiao XJ.

Chinese Medical Journal. 130(19):2375-2379, 2017 Oct 05.

[Journal Article. Review]

UI: 28937045

OBJECTIVE: As a vascular risk factor, carotid atherosclerosis is crucial to cognitive impairment. While carotid intima-media thickness, carotid artery plaque, and carotid stenosis can reflect carotid atherosclerosis in different stages, this review aimed to explore researches on the role of carotid intima-media thickness, carotid artery plaque, and carotid stenosis in the progress of cognitive impairment in nonstroke patients and tried to illustrate the possible mechanisms.

DATA SOURCES: We searched the PubMed database for recently published research articles up to July 2017, with the key words of "carotid atherosclerosis," "carotid intima-media thickness," "carotid plaque," "carotid stenosis," "nonstroke," and "cognitive impairment."

STUDY SELECTION: Articles were obtained and reviewed to analyze the role of carotid atherosclerosis such as carotid intima-thickness, carotid plaque, and carotid stenosis in the progress of cognitive impairment in nonstroke patients and the possible mechanisms.

RESULTS: In recent years, most studies proved that by evaluating carotid atherosclerosis with ultrasonography, carotid atherosclerosis accounts for the development of cognitive decline in nonstroke patients. Carotid atherosclerosis not only impairs the subtle general cognitive function but also decreases the specific domains of cognitive function, such as memory, motor function, visual perception, attention, and executive function. But, it is still controversial. The possible mechanisms of cognitive impairment in nonstroke patients with carotid atherosclerosis can be classified as systemic global cerebrovascular function, small-vessel diseases, and the mixed lesions.

CONCLUSIONS: Carotid atherosclerosis can be used to predict the risk of cognitive impairment. Furthermore, diagnosing and treating carotid atherosclerosis at early stage might help clinicians prevent and treat vascular cognitive impairment in nonstroke patients.

Version ID

1

Record Owner

From MEDLINE, a database of the U.S. National Library of Medicine.

Status

MEDLINE

Authors Full Name

Chen, Wei-Hong; Jin, Wei; Lyu, Pei-Yuan; Liu, Yang; Li, Rui; Hu, Ming; Xiao, Xiang-Jian.

Institution

For peer review only

Chen, Wei-Hong. Graduate School, Hebei Medical University; Department of Neurology, Hebei General Hospital, Shijiazhuang, Hebei 050051, China. Jin, Wei. Department of Neurology, Hebei General Hospital, Shijiazhuang, Hebei 050051, China.
Lyu, Pei-Yuan. Graduate School, Hebei Medical University; Department of Neurology, Hebei General Hospital, Shijiazhuang, Hebei 050051, China.
Liu, Yang. Graduate School, Hebei Medical University; Department of Neurology, Hebei General Hospital, Shijiazhuang, Hebei 050051, China.
Li, Rui. Graduate School, Hebei Medical University; Department of Neurology, Hebei General Hospital, Shijiazhuang, Hebei 050051, China.
Hu, Ming. Department of Neurology, Hebei General Hospital, Shijiazhuang, Hebei 050051, China.
Xiao, Xiang-Jian. Department of Neurology, Hebei General Hospital, Shijiazhuang, Hebei 050051, China.
MeSH Heading
*Carotid Artery Diseases/pp [Physiopathology]. Carotid Intima-Media Thickness. Carotid Stenosis/pp [Physiopathology]. Cognition/ph [Physiology]. Cognitive Dysfunction/pp [Physiopathology]. Female. Humans. Male.
Year of Publication
2017

Link to the Ovid Full Text or citation:
Click here for full text options

Link to the External Link Resolver:
SFX

25.

Asymptomatic carotid stenosis is associated with cognitive impairment.
Lal BK; Dux MC; Sikdar S; Goldstein C; Khan AA; Yokemick J; Zhao L.
Journal of Vascular Surgery. 66(4):1083-1092, 2017 10.
[Journal Article. Research Support, N.I.H., Extramural. Research Support, U.S. Gov't, Non-P.H.S.]
UI: 28712815

For peer review only

BACKGROUND: Cerebrovascular risk factors (eg, hypertension, coronary artery disease) and stroke can lead to vascular cognitive impairment. The Asymptomatic Carotid Stenosis and Cognitive Function study evaluated the isolated impact of asymptomatic carotid stenosis (no prior ipsilateral or contralateral stroke or transient ischemic attack) on cognitive function. Cerebrovascular hemodynamic and carotid plaque characteristics were analyzed to elucidate potential mechanisms affecting cognition.

METHODS: There were 82 patients with >=50% asymptomatic carotid stenosis and 62 controls without stenosis but matched for vascular comorbidities who underwent neurologic, National Institutes of Health Stroke Scale, and comprehensive neuropsychological examination. Overall cognitive function and five domain-specific scores were computed. Duplex ultrasound with Doppler waveform and B-mode imaging defined the degree of stenosis, least luminal diameter, plaque area, and plaque gray-scale median. Breath-holding index (BHI) and microembolization were measured using transcranial Doppler. We assessed cognitive differences between stenosis patients and control patients and of stenosis patients with low vs high BHI and correlated cognitive function with microembolic counts and plaque characteristics.

RESULTS: Stenosis and control patients did not differ in vascular risk factors, education, estimated intelligence, or depressive symptoms. Stenosis patients had worse composite cognitive scores (P = .02; Cohen's d = 0.43) and domain-specific scores for learning/memory (P = .02; d = 0.42) and motor/processing speed (P = .01; d = 0.65), whereas scores for executive function were numerically lower (P = .08). Approximately 49.4% of all stenosis patients were impaired in at least two cognitive domains. Precisely 50% of stenosis patients demonstrated a reduced BHI. Stenosis patients with reduced BHI performed worse on the overall composite cognitive score (t = -2.1; P = .02; d = 0.53) and tests for learning/memory (t = -2.7; P = .01; d = 0.66). Cognitive function did not correlate with measures of plaque burden (degree of stenosis, least luminal diameter, and plaque area) or with plaque gray-scale median.

CONCLUSIONS: Asymptomatic carotid stenosis is associated with cognitive impairment independent of known vascular risk factors for vascular cognitive impairment. Approximately 49.4% of these patients demonstrate impairment in at least two neuropsychological domains. The deficit is driven primarily by reduced motor/processing speed and learning/memory and is mild to moderate in severity. The mechanism for impairment is likely to be hemodynamic as evidenced by reduced cerebrovascular reserve and the likely result of hypoperfusion from a pressure drop across the stenosis in the presence of inadequate collateralization.

Copyright © 2017 Society for Vascular Surgery. Published by Elsevier Inc. All rights reserved.

Version ID

1

Record Owner

From MEDLINE, a database of the U.S. National Library of Medicine.

For peer review only - http://bmjopen.bmj.com/site/about/guidelines.xhtml

Status

MEDLINE

Authors Full Name

Lal, Brajesh K; Dux, Moira C; Sikdar, Siddhartha; Goldstein, Carly; Khan, Amir A; Yokemick, John; Zhao, Limin.

Institution

Lal, Brajesh K. Department of Vascular Surgery, University of Maryland School of Medicine, Baltimore, Md; Vascular Service, Veterans Affairs Medical Center, Baltimore, Md. Electronic address: blal@som.umaryland.edu. Dux, Moira C. Neuropsychology Section, Veterans Affairs Medical Center, Baltimore, Md.

Sikdar, Siddhartha. Department of Bioengineering, George Mason University, Fairfax, Va.

Goldstein, Carly. Department of Vascular Surgery, University of Maryland School of Medicine, Baltimore, Md; Vascular Service, Veterans Affairs Medical Center, Baltimore, Md.

Khan, Amir A. Department of Vascular Surgery, University of Maryland School of Medicine, Baltimore, Md; Department of Bioengineering, George Mason University, Fairfax, Va.

Yokemick, John. Department of Vascular Surgery, University of Maryland School of Medicine, Baltimore, Md.

Zhao, Limin. Department of Vascular Surgery, University of Maryland School of Medicine, Baltimore, Md; Vascular Service, Veterans Affairs Medical Center, Baltimore, Md.

MeSH Heading

Aged. Asymptomatic Diseases. Attention. Carotid Arteries/dg [Diagnostic Imaging]. Carotid Arteries/pp [Physiopathology]. *Carotid Arteries. *Carotid Stenosis/co [Complications]. Carotid Stenosis/dg [Diagnostic Imaging]. Carotid Stenosis/pp [Physiopathology]. Carotid Stenosis/px [Psychology]. Case-Control Studies. Cerebrovascular Circulation. *Cognition. Cognition Disorders/di [Diagnosis]. *Cognition Disorders/et [Etiology]. Cognition Disorders/pp [Physiopathology]. Cognition Disorders/px [Psychology]. Executive Function. Female. Humans. Intracranial Embolism/dg [Diagnostic Imaging]. *Intracranial Embolism/et [Etiology]. Intracranial Embolism/pp [Physiopathology]. Intracranial Embolism/px [Psychology]. Male. Memory. Middle Aged. Motor Activity. Neurologic Examination. Neuropsychological Tests. Plaque, Atherosclerotic. Prospective Studies. Risk Factors. Severity of Illness Index. Ultrasonography, Doppler, Duplex. Ultrasonography, Doppler, Transcranial.

Year of Publication

2017

Link to the Ovid Full Text or citation:

Click here for full text options

For peer review only - http://bmjopen.bmj.com/site/about/guidelines.xhtml

Link to the External Link Resolver:
SFX

29.

Carotid disease at age 73 and cognitive change from age 70 to 76 years: A longitudinal cohort study.
Wardlaw JM; Allerhand M; Eadie E; Thomas A; Corley J; Pattie A; Taylor A; Shenkin SD; Cox S; Gow A; Starr JM; Deary IJ.
Journal of Cerebral Blood Flow & Metabolism. 37(8):3042-3052, 2017 Aug.
[Journal Article]
UI: 28155579
Cognitive decline and carotid artery atheroma are common at older ages. In community-dwelling subjects, we assessed cognition at ages 70, 73 and 76 and carotid Doppler ultrasound at age 73, to determine whether carotid stenosis was related to cognitive decline. We used latent growth curve models to examine associations between four carotid measures (internal carotid artery stenosis, velocity, pulsatility and resistivity indices) and four cognitive ability domains (memory, visuospatial function, crystallised intelligence, processing speed) adjusted for cognitive ability at age 11, current age, gender and vascular risk factors. Amongst 866 participants, carotid stenosis (median 12.96%) was not associated with cognitive abilities at age 70 or cognitive decline from age 70 to 76. Increased ICA pulsatility and resistivity indices were associated with slower processing speed (both $P < 0.001$) and worse visuospatial function ($P = 0.036, 0.031$, respectively) at age 70, and declining crystallised intelligence from ages 70 to 76 ($P = 0.008, 0.006$, respectively). The findings suggest that vascular stiffening, rather than carotid luminal narrowing, adversely influences cognitive ageing and provides a potential target for ameliorating age-related cognitive decline.
Version ID
1
Record Owner
From MEDLINE, a database of the U.S. National Library of Medicine.
Status
MEDLINE
Authors Full Name

Wardlaw, Joanna M; Allerhand, Michael; Eadie, Elizabeth; Thomas, Avril; Corley, Janey; Pattie, Alison; Taylor, Adele; Shenkin, Susan D; Cox, Simon; Gow, Alan; Starr, John M; Deary, Ian J.

Institution

Wardlaw, Joanna M. 1 Brain Research Imaging Centre, Centre for Clinical Brain Sciences, University of Edinburgh, Edinburgh, UK. Wardlaw, Joanna M. 2 Department of Neuroradiology, NHS Lothian, Western General Hospital, Edinburgh, UK.

Wardlaw, Joanna M. 3 Centre for Cognitive Ageing and Cognitive Epidemiology, University of Edinburgh, Edinburgh, UK.

Allerhand, Michael. 3 Centre for Cognitive Ageing and Cognitive Epidemiology, University of Edinburgh, Edinburgh, UK.

Eadie, Elizabeth. 2 Department of Neuroradiology, NHS Lothian, Western General Hospital, Edinburgh, UK.

Thomas, Avril. 2 Department of Neuroradiology, NHS Lothian, Western General Hospital, Edinburgh, UK.

Corley, Janey. 3 Centre for Cognitive Ageing and Cognitive Epidemiology, University of Edinburgh, Edinburgh, UK.

Pattie, Alison. 3 Centre for Cognitive Ageing and Cognitive Epidemiology, University of Edinburgh, Edinburgh, UK.

Taylor, Adele. 3 Centre for Cognitive Ageing and Cognitive Epidemiology, University of Edinburgh, Edinburgh, UK.

Shenkin, Susan D. 3 Centre for Cognitive Ageing and Cognitive Epidemiology, University of Edinburgh, Edinburgh, UK.

Shenkin, Susan D. 4 Geriatric Medicine, University of Edinburgh, Royal Infirmary, Edinburgh, UK.

Cox, Simon. 3 Centre for Cognitive Ageing and Cognitive Epidemiology, University of Edinburgh, Edinburgh, UK.

Gow, Alan. 3 Centre for Cognitive Ageing and Cognitive Epidemiology, University of Edinburgh, Edinburgh, UK.

Gow, Alan. 5 Department of Psychology, Heriot-Watt University, Edinburgh, UK.

Starr, John M. 3 Centre for Cognitive Ageing and Cognitive Epidemiology, University of Edinburgh, Edinburgh, UK.

Deary, Ian J. 3 Centre for Cognitive Ageing and Cognitive Epidemiology, University of Edinburgh, Edinburgh, UK.

MeSH Heading

Aged. Carotid Stenosis/dg [Diagnostic Imaging]. Carotid Stenosis/en [Enzymology]. *Carotid Stenosis/px [Psychology]. *Cognition/ph [Physiology]. Cognition Disorders/ep [Epidemiology]. *Cognition Disorders/px [Psychology]. Cognitive Aging/ph [Physiology]. *Cognitive Aging/px [Psychology]. Female. Humans. Longitudinal Studies. Male. Models, Neurological. Plaque,

For peer review only

Atherosclerotic/dg [Diagnostic Imaging]. Plaque, Atherosclerotic/ep [Epidemiology]. *Plaque, Atherosclerotic/px [Psychology]. Ultrasonography, Doppler, Color.

Keyword Heading

Carotid stenosis ageing

cognition

vascular risk factors

white matter hyperintensities.

Year of Publication

2017

Link to the Ovid Full Text or citation:

Click here for full text options

Link to the External Link Resolver:

SFX

35.

Atherosclerotic carotid stenosis and cognitive function. [Review]

Wang T; Mei B; Zhang J.

Clinical Neurology & Neurosurgery. 146:64-70, 2016 Jul.

[Journal Article. Review]

UI: 27152468

Atherosclerosis carotid stenosis is associated with stroke and cognitive impairment. Progressive cognitive decline may be an even greater problem than stroke, but it has not been widely recognized and therefore must be adequately addressed. Although both Carotid Endarterectomy (CEA) and Carotid Artery Stenting (CAS) have been proven can prevent future stroke in patients with atherosclerotic carotid stenosis, the influence of CEA and CAS on cognitive function is not clear. In the first part of this review, we evaluated the literature concerning carotid stenosis and the risk of cognitive impairment. Studies have suggested that both symptomatic and asymptomatic carotid stenosis are associated with cognitive impairment. In the second part, we reviewed the impact of CEA and CAS on cognitive function, some studies have shown benefits, but others have not.

For peer review only

Copyright © 2016 Elsevier B.V. All rights reserved.

Version ID

1

Record Owner

From MEDLINE, a database of the U.S. National Library of Medicine.

Status

MEDLINE

Authors Full Name

Wang, Tao; Mei, Bin; Zhang, Junjian.

Institution

Wang, Tao. Department of Neurology and Neuropsychological Research Center, Zhongnan Hospital, Wuhan University, Wuhan, China. Mei, Bin. Department of Neurology and Neuropsychological Research Center, Zhongnan Hospital, Wuhan University, Wuhan, China. Zhang, Junjian. Department of Neurology and Neuropsychological Research Center, Zhongnan Hospital, Wuhan University, Wuhan, China. Electronic address: 851895243@qq.com.

MeSH Heading

*Atherosclerosis/co [Complications]. *Carotid Stenosis/co [Complications]. *Cognitive Dysfunction/et [Etiology]. Humans.

Keyword Heading

Carotid artery stenting Carotid endarterectomy

Carotid stenosis

Cognitive function

Revascularization.

Year of Publication

2016

Link to the Ovid Full Text or citation:

Click here for full text options

Link to the External Link Resolver:

SFX

37.

For peer review only

Carotid atherosclerosis, cytomegalovirus infection, and cognitive decline in the very old: a community-based prospective cohort study.

Kawasaki M; Arai Y; Takayama M; Hirata T; Takayama M; Abe Y; Niimura H; Mimura M; Takebayashi T; Hirose N.

Age. 38(2):29, 2016 Apr.

[Journal Article. Multicenter Study. Observational Study. Research Support, Non-U.S. Gov't]

UI: 26886582

To investigate various risk factors of cognitive decline in the very old, we studied 494 subjects over 85 years old without diagnosis of dementia at baseline from the Tokyo Oldest Old Survey on Total Health, an ongoing, community-based cohort in Japan. Cognitive function was assessed at baseline and at 3-year follow-up using Mini-Mental State Examination (MMSE). Plasma samples were assayed for levels of cytomegalovirus (CMV) immunoglobulin G (IgG) antibodies, tumor necrosis factor-alpha, interleukin-6, and blood chemistry. Carotid artery plaques were measured using an ultrasonography. In the cross-sectional analyses using Tobit regression, individuals with high carotid artery plaque score (>=5.0) had MMSE scores that were 1.08 points lower compared to those with no plaque (95 % confidence interval (CI) -1.95 to -0.20; p = 0.016), adjusted for age, sex, and education. Individuals with CMV IgG titers in the highest quartile had MMSE scores that were 1.47 points lower compared to individuals in the lowest quartile (95 % CI -2.44 to -0.50; p = 0.003). CMV and carotid atherosclerosis showed evidence of an interaction, where the association between CMV and MMSE was present only in subjects with carotid artery plaque. In the longitudinal analyses using linear regression, carotid atherosclerosis, smoking, low grip strength, and poor activities of daily living (ADL) status were associated with faster cognitive decline, adjusted for age, sex, education, and baseline cognitive function. Our findings suggest that carotid atherosclerosis is consistently associated with low cognitive function in the very old and modifies the association between latent CMV infection and cognition.

Version ID

1

Record Owner

From MEDLINE, a database of the U.S. National Library of Medicine.

Status

MEDLINE

Authors Full Name

Kawasaki, Midori; Arai, Yasumichi; Takayama, Michiyo; Hirata, Takumi; Takayama, Midori; Abe, Yukiko; Niimura, Hidehito; Mimura, Masaru; Takebayashi, Toru; Hirose, Nobuyoshi.

Institution

For peer review only

Kawasaki, Midori. Center for Supercentenarian Medical Research, Keio University School of Medicine, 35 Shinanomachi, Shinjuku-ku, Tokyo, 160-8582, Japan. Arai, Yasumichi. Center for Supercentenarian Medical Research, Keio University School of Medicine, 35 Shinanomachi, Shinjuku-ku, Tokyo, 160-8582, Japan. yasumich@keio.jp.
Takayama, Michiyo. Center for Preventive Medicine, Keio University School of Medicine, 35 Shinanomachi, Shinjuku-ku, Tokyo, 160-8582, Japan.
Hirata, Takumi. Center for Supercentenarian Medical Research, Keio University School of Medicine, 35 Shinanomachi, Shinjuku-ku, Tokyo, 160-8582, Japan.
Takayama, Midori. Faculty of Science and Technology, Keio University, 4-1-1 Hiyoshi, Kohoku-ku, Yokohama, Kanagawa, 252-8520, Japan.
Abe, Yukiko. Center for Supercentenarian Medical Research, Keio University School of Medicine, 35 Shinanomachi, Shinjuku-ku, Tokyo, 160-8582, Japan.
Niimura, Hidehito. Department of Neuropsychiatry, Keio University School of Medicine, 35 Shinanomachi, Shinjuku-ku, Tokyo, 160-8582, Japan.
Mimura, Masaru. Department of Neuropsychiatry, Keio University School of Medicine, 35 Shinanomachi, Shinjuku-ku, Tokyo, 160-8582, Japan.
Takebayashi, Toru. Department of Preventative Medicine and Public Health, Keio University School of Medicine, 35 Shinanomachi, Shinjuku-ku, Tokyo, 160-8582, Japan.
Hirose, Nobuyoshi. Center for Supercentenarian Medical Research, Keio University School of Medicine, 35 Shinanomachi, Shinjuku-ku, Tokyo, 160-8582, Japan.

MeSH Heading

Aged, 80 and over. *Aging. Carotid Artery Diseases/di [Diagnosis]. *Carotid Artery Diseases/ep [Epidemiology]. Cognition. Cognition Disorders/di [Diagnosis]. *Cognition Disorders/ep [Epidemiology]. Cross-Sectional Studies. Cytomegalovirus Infections/di [Diagnosis]. *Cytomegalovirus Infections/ep [Epidemiology]. Female. Follow-Up Studies. Humans. Incidence. Japan/ep [Epidemiology]. Male. Neuropsychological Tests. *Population Surveillance. Prospective Studies. Risk Factors. Time Factors.

Keyword Heading

Atherosclerosis Cognitive decline

Cytomegalovirus

Inflammation

Very old.

Year of Publication

2016

Link to the Ovid Full Text or citation:

For peer review only - http://bmjopen.bmj.com/site/about/guidelines.xhtml

Click here for full text options

Link to the External Link Resolver:
SFX

38.

Low carotid artery wall shear stress is independently associated with brain white-matter hyperintensities and cognitive impairment in older patients.

Liu Z; Zhao Y; Wang X; Zhang H; Cui Y; Diao Y; Xiu J; Sun X; Jiang G.

Atherosclerosis. 247:78-86, 2016 Apr.

[Journal Article. Research Support, Non-U.S. Gov't]

UI: 26868512

BACKGROUND & AIMS: Brain white-matter lesions and cognitive impairment are increasing because of the increasing number of patients aged >=80 y. Wall shear stress (WSS) plays a pivotal role as a fluid mechanical mediator in vascular reactivity and atherosclerosis. In this study, we investigated the associations among common carotid artery (CCA) WSS, white-matter lesions, and cognitive impairment in patients aged >=80 y

METHODS: We enrolled 384 patients aged >=80 y. All subjects had CCA-WSS, brain white-matter hyperintensities (WMH), and Mini-Mental State Examination (MMSE) assessments and were divided into three groups using tertiles of mean and peak CCA-WSS.

RESULTS: For groups classified by the tertile of mean CCA-WSS, WMH, and WMH fraction were decreased; the MMSE score increased from low to high in the respective groups. Differences in WMH, WMH fraction, and the MMSE score were significant between any two groups (all adjusted p < 0.001). Groups classified by the tertile of peak CCA-WSS had the same pattern. Mean and peak CCA-WSS were significantly and inversely correlated with WMH (r = -0.575 and -0.570, respectively; p < 0.001) and WMH fraction (r = -0.574 and -0.569, respectively; p < 0.001) but positively correlated with the MMSE score (r = 0.390 and 0.278, respectively; p < 0.001). Multiple linear backward stepwise regression indicated the mean and peak CCA-WSS were significantly and independently associated with WMH, WMH fraction, and the MMSE score (all adjusted p < 0.001).

CONCLUSION: Carotid artery WSS was independently associated with brain white-matter lesions and cognitive impairment in patients aged >=80 y.

Copyright © 2016 Elsevier Ireland Ltd. All rights reserved.

Version ID

For peer review only - http://bmjopen.bmj.com/site/about/guidelines.xhtml

1

Record Owner

From MEDLINE, a database of the U.S. National Library of Medicine.

Status

MEDLINE

Authors Full Name

Liu, Zhendong; Zhao, Yingxin; Wang, Xidi; Zhang, Hua; Cui, Yi; Diao, Yutao; Xiu, Jianchao; Sun, Xiaolin; Jiang, Guosheng.

Institution

Liu, Zhendong. Cardio-Cerebrovascular Control and Research Center, Institute of Basic Medicine, Shandong Academy of Medical Sciences, Jinan, Shandong, 250062, China. Electronic address: zhendongliu876@126.com. Zhao, Yingxin. Cardio-Cerebrovascular Control and Research Center, Institute of Basic Medicine, Shandong Academy of Medical Sciences, Jinan, Shandong, 250062, China.

Wang, Xidi. Department of Neurology, Zhangqiu People's Hospital, Zhangqiu, Shandong, 250200, China.

Zhang, Hua. Cardio-Cerebrovascular Control and Research Center, Institute of Basic Medicine, Shandong Academy of Medical Sciences, Jinan, Shandong, 250062, China.

Cui, Yi. Department of Radiology, Qilu Hospital of Shandong University, Jinan, Shandong, 250012, China.

Diao, Yutao. Cardio-Cerebrovascular Control and Research Center, Institute of Basic Medicine, Shandong Academy of Medical Sciences, Jinan, Shandong, 250062, China.

Xiu, Jianchao. Cardio-Cerebrovascular Control and Research Center, Institute of Basic Medicine, Shandong Academy of Medical Sciences, Jinan, Shandong, 250062, China.

Sun, Xiaolin. Cardio-Cerebrovascular Control and Research Center, Institute of Basic Medicine, Shandong Academy of Medical Sciences, Jinan, Shandong, 250062, China.

Jiang, Guosheng. Cardio-Cerebrovascular Control and Research Center, Institute of Basic Medicine, Shandong Academy of Medical Sciences, Jinan, Shandong, 250062, China. Electronic address: jiangguosh@163.com.

MeSH Heading

Age Factors. Aged, 80 and over. Blood Flow Velocity. *Carotid Artery Diseases/co [Complications]. Carotid Artery Diseases/dg [Diagnostic Imaging]. Carotid Artery Diseases/pp [Physiopathology]. Carotid Artery, Common/dg [Diagnostic Imaging]. *Carotid Artery, Common/pp [Physiopathology]. Carotid Intima-Media Thickness. *Cognition. Cognition Disorders/di [Diagnosis]. *Cognition Disorders/et [Etiology]. Cognition Disorders/px [Psychology]. Cross-Sectional Studies. Female. Humans. Leukoencephalopathies/dg [Diagnostic Imaging]. *Leukoencephalopathies/et [Etiology]. Linear Models. Magnetic Resonance Imaging. Male. Multivariate Analysis. Psychiatric

For peer review only

Status Rating Scales. Regional Blood Flow. Risk Factors. Stress, Mechanical. Ultrasonography, Doppler, Duplex.

Keyword Heading

Atherosclerosis Cognitive function

Older patients

Wall shear stress

White matter lesions.

Year of Publication

2016

Link to the Ovid Full Text or citation:

Click here for full text options

Link to the External Link Resolver:

SFX

39.

Classification of Symptomatic and Asymptomatic Patients with and without Cognitive Decline Using Non-invasive Carotid Plaque Strain Indices as Biomarkers.

Wang X; Jackson DC; Mitchell CC; Varghese T; Wilbrand SM; Rocque BG; Hermann BP; Dempsey RJ.

Ultrasound in Medicine & Biology. 42(4):909-18, 2016 Apr.

[Journal Article. Research Support, N.I.H., Extramural. Research Support, Non-U.S. Gov't]

UI: 26778288

Vascular cognitive decline may be caused by micro-emboli generated by carotid plaque instability. We previously found that maximum strain indices in carotid plaque were significantly correlated with cognitive function. In the work described here, we examined these associations with a larger sample size, as well as evaluated the performance of these maximum strain indices in predicting cognitive impairment. Ultrasound-based strain imaging and cognition assessment were conducted on 75 human patients. Patients underwent one of two standardized cognitive test batteries, either the Repeatable Battery for the Assessment of Neuropsychological Status (RBANS) or the National Institute of Neurologic Disorder and Stroke-Canadian Stroke Network (NINDS-CSN) Vascular

For peer review only

Cognitive Impairment Harmonization Standards (60 min). Scores were standardized within each battery to allow these data to be combined across all participants. Radiofrequency signals for ultrasound strain imaging were acquired on the carotid arteries using either a Siemens Antares with a VFX 13-5 linear array transducer or a Siemens S2000 with an 18 L6 linear array transducer. The same hierarchical block-matching motion tracking algorithm developed in our laboratory was used to estimate accumulated axial, lateral, and shear strain indices in carotid plaque, with inclusion of adventitia regardless of the ultrasound system and transducer used. Associations between cognitive z-scores and maximum strain indices were examined using Pearson's correlation coefficients. Maximum strain indices were also employed to predict cognitive impairment using receiver operating characteristic analysis. All correlations between maximum strain indices and total cognition were statistically significant ($p < 0.05$), indicating that these indices have good utility in predicting cognitive impairment. Maximum lateral strain indices provided an area under the curve of 0.85 for symptomatic patients and 0.68 for asymptomatic patients. Our results indicate the important relationship of maximum strain indices to cognitive function and the feasibility of using maximum strain indices to predict cognitive decline with inclusion of the adventitia layer into the segmentation of plaque.

Copyright © 2016 World Federation for Ultrasound in Medicine & Biology. Published by Elsevier Inc. All rights reserved.

Version ID

1

Record Owner

From MEDLINE, a database of the U.S. National Library of Medicine.

Status

MEDLINE

Authors Full Name

Wang, Xiao; Jackson, Daren C; Mitchell, Carol C; Varghese, Tomy; Wilbrand, Stephanie M; Rocque, Brandon G; Hermann, Bruce P; Dempsey, Robert J.

Institution

Wang, Xiao. Department of Medical Physics, University of Wisconsin School of Medicine and Public Health, University of Wisconsin-Madison, Madison, Wisconsin, USA. Electronic address: xwang235@wisc.edu.   Jackson, Daren C. Department of Neurology, University of Wisconsin School of Medicine and Public Health, University of Wisconsin-Madison, Madison, Wisconsin, USA.
Mitchell, Carol C. Department of Medicine, University of Wisconsin School of Medicine and Public Health, University of Wisconsin-Madison, Madison, Wisconsin, USA.
Varghese, Tomy. Department of Medical Physics, University of Wisconsin School of Medicine and Public Health, University of Wisconsin-Madison, Madison, Wisconsin, USA.

For peer review only

Wilbrand, Stephanie M. Department of Neurological Surgery, University of Wisconsin School of Medicine and Public Health, University of Wisconsin-Madison, Madison, Wisconsin, USA.
Rocque, Brandon G. Department of Neurological Surgery, University of Wisconsin School of Medicine and Public Health, University of Wisconsin-Madison, Madison, Wisconsin, USA.
Hermann, Bruce P. Department of Neurology, University of Wisconsin School of Medicine and Public Health, University of Wisconsin-Madison, Madison, Wisconsin, USA.
Dempsey, Robert J. Department of Neurological Surgery, University of Wisconsin School of Medicine and Public Health, University of Wisconsin-Madison, Madison, Wisconsin, USA.

MeSH Heading

Adult. Aged. Aged, 80 and over. Algorithms. Asymptomatic Diseases. *Carotid Stenosis/co [Complications]. *Carotid Stenosis/dg [Diagnostic Imaging]. *Cognitive Dysfunction/co [Complications]. *Cognitive Dysfunction/dg [Diagnostic Imaging]. Diagnosis, Differential. *Elasticity Imaging Techniques/mt [Methods]. Female. Humans. Male. Middle Aged. *Pattern Recognition, Automated/mt [Methods]. Reproducibility of Results. Sensitivity and Specificity.

Keyword Heading

Adventitia   Carotid Plaque

Elasticity imaging

Elastography

Motion tracking

Multi-level

Strain imaging

Vascular cognitive impairment.

Year of Publication

2016

Link to the Ovid Full Text or citation:

Click here for full text options

Link to the External Link Resolver:

SFX

41.

For peer review only - http://bmjopen.bmj.com/site/about/guidelines.xhtml

Improved Correlation of Strain Indices with Cognitive Dysfunction with Inclusion of Adventitial Layer with Carotid Plaque.

Wang X; Mitchell CC; Varghese T; Jackson DC; Rocque BG; Hermann BP; Dempsey RJ.

Ultrasonic Imaging. 38(3):194-208, 2016 May.

[Journal Article. Research Support, N.I.H., Extramural. Research Support, Non-U.S. Gov't. Research Support, U.S. Gov't, Non-P.H.S.]

UI: 26025578

Plaque instability may lead to chronic embolization, which in turn may contribute to progressive cognitive decline. Accumulated strain tensor indices over a cardiac cycle within a pulsating carotid plaque may be viable biomarkers for the diagnosis of plaque instability. Using plaque-only carotid artery segmentations, we recently demonstrated that impaired cognitive function correlated significantly with maximum axial and lateral strain indices within a localized region of interest in plaque. Inclusion of the adventitial layer focuses our strain or instability measures on the vessel wall-plaque interface hypothesized to be a region with increased shearing forces and measureable instability. A hierarchical block-matching motion tracking algorithm developed in our laboratory was used to estimate accumulated axial, lateral, and shear strain distribution in plaques identified with the plaque-with-adventitia segmentation. Correlations of strain indices to the Repeatable Battery for the Assessment of Neuropsychological Status Total score were performed and compared with previous results. Overall, correlation coefficients (r) and significance (p) values improved for axial, lateral, and shear strain indices. Shear strain indices, however, demonstrated the largest improvement. The Pearson correlation coefficients for maximum shear strain and cognition improved from the previous plaque-only analyses of -0.432 and -0.345 to -0.795 and -0.717 with the plaque-with-adventitia segmentation for the symptomatic group and for all patients combined, respectively. Our results demonstrate the advantage of including adventitia for ultrasound carotid strain imaging providing improved association to parameters assessing cognitive impairment in patients. This supports theories of the importance of the vessel wall plaque interface in the pathophysiology of embolic disease.

Copyright © The Author(s) 2015.

Version ID

1

Record Owner

From MEDLINE, a database of the U.S. National Library of Medicine.

Status

MEDLINE

Authors Full Name

Wang, X; Mitchell, C C; Varghese, T; Jackson, D C; Rocque, B G; Hermann, B P; Dempsey, R J.

Institution

For peer review only - http://bmjopen.bmj.com/site/about/guidelines.xhtml

Wang, X. Department of Medical Physics, University of Wisconsin-Madison School of Medicine and Public Health, Madison, WI, USA. Mitchell, C C. Department of Medicine, University of Wisconsin-Madison, Madison School of Medicine and Public Health, WI, USA.
Varghese, T. Department of Medical Physics, University of Wisconsin-Madison School of Medicine and Public Health, Madison, WI, USA tvarghese@wisc.edu.
Jackson, D C. Department of Neurology, University of Wisconsin-Madison, Madison School of Medicine and Public Health, WI, USA.
Rocque, B G. Department of Neurological Surgery, University of Wisconsin-Madison School of Medicine and Public Health, Madison, WI, USA.
Hermann, B P. Department of Neurology, University of Wisconsin-Madison, Madison School of Medicine and Public Health, WI, USA.
Dempsey, R J. Department of Neurological Surgery, University of Wisconsin-Madison School of Medicine and Public Health, Madison, WI, USA.
MeSH Heading
Adult. *Adventitia/dg [Diagnostic Imaging]. Aged. *Carotid Stenosis/dg [Diagnostic Imaging]. *Cognitive Dysfunction/et [Etiology]. Elasticity Imaging Techniques. Female. Humans. *Image Interpretation, Computer-Assisted/mt [Methods]. Male. Middle Aged. Neuropsychological Tests.
Keyword Heading
carotid plaque elasticity imaging
motion tracking
strain
vascular cognitive dementia.
Year of Publication
2016

Link to the Ovid Full Text or citation:
Click here for full text options

Link to the External Link Resolver:
SFX

44.

For peer review only

Subclinical carotid artery atherosclerosis and performance on cognitive tests in middle-aged adults: Baseline results from the ELSA-Brasil.

Suemoto CK; Santos IS; Bittencourt MS; Pereira AC; Goulart AC; Rundek T; Passos VM; Lotufo P; Bensenor IM.

Atherosclerosis. 243(2):510-5, 2015 Dec.

[Journal Article. Multicenter Study. Research Support, Non-U.S. Gov't]

UI: 26520907

BACKGROUND AND AIMS: Carotid artery intima-media thickness (CIMT) may be used as a biomarker for early cognitive impairment. However, the results of the association between CIMT and cognitive function in middle-aged subjects are mixed. We aimed to investigate this association in a large Brazilian sample with no history of stroke at baseline. Additionally, we tested the effect of interactions between CIMT and cardiovascular risk factors on cognitive performance.

METHODS: In this cross-sectional study, cognition was evaluated using the delayed word recall (DWRT), the category fluency, and the trail making tests (TMT). CIMT was measured at the common carotid artery. The association between CIMT and cognitive tests was investigated using linear regression models, adjusted for an extensive set of possible confounding variables. We also included interaction terms with selected risk factors.

RESULTS: The mean age of the 8208 participants was 49.6 +/- 7.3 years, 44% were male, and 56% White. Increase in CIMT was associated with worse performance on the DWRT (beta = -0.433, 95%CI = -0.724;-0.142, p = 0.004). We found effect modification of the association between cognitive function and CMIT by self-reported heart failure and alcohol intake. Participants had worse performance in the TMT if they had greater CIMT and current alcohol use (p < 0.0001). The interaction between CIMT and heart failure on TMT performance was not significant after adjustment for multiple comparisons (p = 0.07).

CONCLUSIONS: In this sample of middle-aged adults, CIMT was inversely associated with memory function. Additionally, the presence of alcohol use resulted in a stronger association of CIMT with worse performance on an executive function test.

Copyright © 2015 Elsevier Ireland Ltd. All rights reserved.

Version ID

1

Record Owner

From MEDLINE, a database of the U.S. National Library of Medicine.

Status

MEDLINE

Authors Full Name

Suemoto, Claudia K; Santos, Itamar S; Bittencourt, Marcio S; Pereira, Alexandre C; Goulart, Alessandra C; Rundek, Tatjana; Passos, Valeria M; Lotufo, Paulo; Bensenor, Isabela M.

For peer review only - http://bmjopen.bmj.com/site/about/guidelines.xhtml

Institution

Suemoto, Claudia K. Division of Geriatrics, University of Sao Paulo Medical School, Sao Paulo, Brazil; Department of Global Health and Population, Harvard School of Public Health, Boston, USA. Electronic address: cksuemoto@usp.br. Santos, Itamar S. Department of Internal Medicine, University of Sao Paulo Medical School, Sao Paulo, Brazil; Center for Clinical and Epidemiological Research, Hospital Universitario, University of Sao Paulo, Sao Paulo, Brazil.

Bittencourt, Marcio S. Center for Clinical and Epidemiological Research, Hospital Universitario, University of Sao Paulo, Sao Paulo, Brazil.

Pereira, Alexandre C. Laboratory of Genetics and Molecular Cardiology, Division of Cardiology, University of Sao Paulo Medical School, Sao Paulo, Brazil.

Goulart, Alessandra C. Center for Clinical and Epidemiological Research, Hospital Universitario, University of Sao Paulo, Sao Paulo, Brazil.

Rundek, Tatjana. Departments of Neurology and Public Health Sciences, University of Miami, Miller School of Medicine, Miami, FL, USA.

Passos, Valeria M. Department of Internal Medicine, Federal University of Minas Gerais Medical School, Minas Gerais, Brazil.

Lotufo, Paulo. Department of Internal Medicine, University of Sao Paulo Medical School, Sao Paulo, Brazil; Center for Clinical and Epidemiological Research, Hospital Universitario, University of Sao Paulo, Sao Paulo, Brazil.

Bensenor, Isabela M. Department of Internal Medicine, University of Sao Paulo Medical School, Sao Paulo, Brazil; Center for Clinical and Epidemiological Research, Hospital Universitario, University of Sao Paulo, Sao Paulo, Brazil.

MeSH Heading

Adult. Alcohol Drinking/ae [Adverse Effects]. Alcohol Drinking/px [Psychology]. Asymptomatic Diseases. Brazil. *Carotid Artery Diseases/co [Complications]. Carotid Artery Diseases/dg [Diagnostic Imaging]. Carotid Artery Diseases/px [Psychology]. Carotid Artery, Common/dg [Diagnostic Imaging]. *Carotid Artery, Common. Carotid Intima-Media Thickness. *Cognition. Cognition Disorders/di [Diagnosis]. *Cognition Disorders/et [Etiology]. Cognition Disorders/px [Psychology]. Cross-Sectional Studies. Executive Function. Female. Heart Failure/co [Complications]. Heart Failure/px [Psychology]. Humans. Linear Models. Male. Mental Recall. Middle Aged. Multivariate Analysis. *Plaque, Atherosclerotic. Predictive Value of Tests. Risk Factors. Trail Making Test.

Keyword Heading

Atherosclerosis Carotid intima media thickness

Cognition disorders.

Year of Publication

2015

For peer review only - http://bmjopen.bmj.com/site/about/guidelines.xhtml

Link to the Ovid Full Text or citation:

Click here for full text options

Link to the External Link Resolver:

SFX

46.

Subclinical Atherosclerosis Is Inversely Associated With Gray Matter Volume in African Americans With Type 2 Diabetes.

Freedman BI; Divers J; Whitlow CT; Bowden DW; Palmer ND; Smith SC; Xu J; Register TC; Carr JJ; Wagner BC; Williamson JD; Sink KM; Maldjian JA.

Diabetes Care. 38(11):2158-65, 2015 Nov.

[Journal Article. Research Support, N.I.H., Extramural]

UI: 26370382

OBJECTIVE: Relative to European Americans, African Americans manifest lower levels of computed tomography-based calcified atherosclerotic plaque (CP), a measure of subclinical cardiovascular disease (CVD). Potential relationships between CP and cerebral structure are poorly defined in the African American population. We assessed associations among glycemic control, inflammation, and CP with cerebral structure on MRI and with cognitive performance in 268 high-risk African Americans with type 2 diabetes.

RESEARCH DESIGN AND METHODS: Associations among hemoglobin A1c (HbA1c), C-reactive protein (CRP), and CP in coronary arteries, carotid arteries, and the aorta with MRI volumetric analysis (white matter volume, gray matter volume [GMV], cerebrospinal fluid volume, and white matter lesion volume) were assessed using generalized linear models adjusted for age, sex, African ancestry proportion, smoking, BMI, use of statins, HbA1c, hypertension, and prior CVD.

RESULTS: Participants were 63.4% female with mean (SD) age of 59.8 years (9.2), diabetes duration of 14.5 years (7.6), HbA1c of 7.95% (1.9), estimated glomerular filtration rate of 86.6 mL/min/1.73 m(2) (24.6), and coronary artery CP mass score of 215 mg (502). In fully adjusted models, GMV was inversely associated with coronary artery CP (parameter estimate [beta] -0.47 [SE 0.15], P = 0.002; carotid artery CP (beta -1.92 [SE 0.62], P = 0.002; and aorta CP [beta -0.10 [SE 0.03] P = 0.002),

For peer review only - http://bmjopen.bmj.com/site/about/guidelines.xhtml

whereas HbA1c and CRP did not associate with cerebral volumes. Coronary artery CP also associated with poorer global cognitive function on the Montreal Cognitive Assessment. CONCLUSIONS: Subclinical atherosclerosis was associated with smaller GMV and poorer cognitive performance in African Americans with diabetes. Cardioprotective strategies could preserve GMV and cognitive function in high-risk African Americans with diabetes.
Copyright © 2015 by the American Diabetes Association. Readers may use this article as long as the work is properly cited, the use is educational and not for profit, and the work is not altered.

Version ID

1

Record Owner

From MEDLINE, a database of the U.S. National Library of Medicine.

Status

MEDLINE

Authors Full Name

Freedman, Barry I; Divers, Jasmin; Whitlow, Christopher T; Bowden, Donald W; Palmer, Nicholette D; Smith, S Carrie; Xu, Jianzhao; Register, Thomas C; Carr, J Jeffrey; Wagner, Benjamin C; Williamson, Jeff D; Sink, Kaycee M; Maldjian, Joseph A.

Institution

Freedman, Barry I. Department of Internal Medicine, Section on Nephrology, Wake Forest School of Medicine, Winston-Salem, NC Center for Diabetes Research and Center for Genomics and Personalized Medicine Research, Wake Forest School of Medicine, Winston-Salem, NC bfreedma@wakehealth.edu. Divers, Jasmin. Division of Public Health Sciences, Department of Biostatistical Sciences, Wake Forest School of Medicine, Winston-Salem, NC.

Whitlow, Christopher T. Advanced Neuroscience Imaging Research Laboratory, Department of Radiologic Sciences, Wake Forest School of Medicine, Winston-Salem, NC.

Bowden, Donald W. Center for Diabetes Research and Center for Genomics and Personalized Medicine Research, Wake Forest School of Medicine, Winston-Salem, NC Department of Biochemistry, Wake Forest School of Medicine, Winston-Salem, NC.

Palmer, Nicholette D. Center for Diabetes Research and Center for Genomics and Personalized Medicine Research, Wake Forest School of Medicine, Winston-Salem, NC Department of Biochemistry, Wake Forest School of Medicine, Winston-Salem, NC.

Smith, S Carrie. Department of Biochemistry, Wake Forest School of Medicine, Winston-Salem, NC.

Xu, Jianzhao. Center for Diabetes Research and Center for Genomics and Personalized Medicine Research, Wake Forest School of Medicine, Winston-Salem, NC.

Register, Thomas C. Department of Pathology, Wake Forest School of Medicine, Winston-Salem, NC.

Carr, J Jeffrey. Department of Radiology, Vanderbilt University School of Medicine, Nashville, TN.

For peer review only

Wagner, Benjamin C. Advanced Neuroscience Imaging Research Laboratory, Department of Radiologic Sciences, Wake Forest School of Medicine, Winston-Salem, NC.
Williamson, Jeff D. Section on Gerontology and Geriatric Medicine, Wake Forest School of Medicine, Department of Internal Medicine, Winston-Salem, NC.
Sink, Kaycee M. Section on Gerontology and Geriatric Medicine, Wake Forest School of Medicine, Department of Internal Medicine, Winston-Salem, NC.
Maldjian, Joseph A. Advanced Neuroscience Imaging Research Laboratory, Department of Radiologic Sciences, Wake Forest School of Medicine, Winston-Salem, NC.
MeSH Heading
*African Americans. Aged. *Atherosclerosis/co [Complications]. Atherosclerosis/di [Diagnosis]. Atherosclerosis/eh [Ethnology]. Blood Glucose/an [Analysis]. Cognition. Cognition Disorders/et [Etiology]. *Cognition Disorders/pa [Pathology]. *Diabetes Mellitus, Type 2/co [Complications]. Diabetes Mellitus, Type 2/eh [Ethnology]. Female. *Gray Matter/pa [Pathology]. Humans. Magnetic Resonance Imaging. Male. Middle Aged. Organ Size. Plaque, Atherosclerotic/dg [Diagnostic Imaging]. Plaque, Atherosclerotic/pa [Pathology]. Radiography.
Registry Number/Name of Substance
0 (Blood Glucose).
Year of Publication
2015

Link to the Ovid Full Text or citation:
[Click here for full text options]

Link to the External Link Resolver:
[SFX]

47.

Intima-Media Thickness and Cognitive Function in Stroke-Free Middle-Aged Adults: Findings From the Coronary Artery Risk Development in Young Adults Study.
Zeki Al Hazzouri A; Vittinghoff E; Sidney S; Reis JP; Jacobs DR Jr; Yaffe K.
Stroke. 46(8):2190-6, 2015 Aug.
[Journal Article. Research Support, N.I.H., Extramural. Research Support, Non-U.S. Gov't]

For peer review only

UI: 26106116

BACKGROUND AND PURPOSE: The relationship between carotid artery intima-media thickness (IMT) and cognitive function in midlife remains relatively unexplored. We examined the association between IMT and cognitive function in a middle-aged epidemiological cohort of 2618 stroke-free participants.

METHODS: At the year 20 visit (our study baseline), participants from the Coronary Artery Risk Development in Young Adults study had IMT measured by ultrasound at the common carotid artery. Five years later, participants completed a cognitive battery consisting of the Rey Auditory-Verbal Learning Test of verbal memory, the Digit Symbol Substitution Test of processing speed, and the Stroop test of executive function. We transformed cognitive scores into standardized z scores, with negative values indicating worse performance.

RESULTS: Mean age at baseline was 45.3 years (SD, 3.6). Greater IMT (per 1 SD difference of 0.12 mm) was significantly associated with worse performance on all cognitive tests (z scores) in unadjusted linear regression models (verbal memory, -0.16; 95% confidence interval [CI], -0.20 to -0.13; processing speed, -0.23; 95% CI, -0.27 to -0.19; and executive function, -0.17; 95% CI, -0.20 to -0.13). In models adjusted for sociodemographics and vascular risk factors that lie earlier in the causal pathway, greater IMT remained negatively associated with processing speed (-0.06; 95% CI, -0.09 to -0.02; P, 0.003) and borderline associated with executive function (-0.03; 95% CI, -0.07 to 0.00; P, 0.07) but not with verbal memory.

CONCLUSIONS: We observed an association between greater IMT and worse processing speed-a key component of cognitive functioning-at middle age above and beyond traditional vascular risk factors. Efforts targeted at preventing early stages of atherosclerosis may modify the course of cognitive aging.

Copyright © 2015 American Heart Association, Inc.

Version ID

1

Record Owner

From MEDLINE, a database of the U.S. National Library of Medicine.

Status

MEDLINE

Authors Full Name

Zeki Al Hazzouri, Adina; Vittinghoff, Eric; Sidney, Stephen; Reis, Jared P; Jacobs, David R Jr; Yaffe, Kristine.

Institution

Zeki Al Hazzouri, Adina. From the Division of Epidemiology and Population Health Sciences, Department of Public Health Sciences, University of Miami, FL (A.Z.A.H.); Departments of Epidemiology and Biostatistics (E.V., K.Y.), Psychiatry (K.Y.), and Neurology (K.Y.), University of

For peer review only - http://bmjopen.bmj.com/site/about/guidelines.xhtml

California San Francisco; San Francisco Veterans Affairs Medical Center, San Francisco, CA (K.Y.); Kaiser Permanente Division of Research, Oakland, CA (S.S.); Division of Cardiovascular Sciences, National Heart, Lung, and Blood Institute, National Institutes of Health, Bethesda, MD (J.P.R.); and Division of Epidemiology and Community Health, School of Public Health, University of Minnesota, Minneapolis (D.R.J.). axz122@miami.edu. Vittinghoff, Eric. From the Division of Epidemiology and Population Health Sciences, Department of Public Health Sciences, University of Miami, FL (A.Z.A.H.); Departments of Epidemiology and Biostatistics (E.V., K.Y.), Psychiatry (K.Y.), and Neurology (K.Y.), University of California San Francisco; San Francisco Veterans Affairs Medical Center, San Francisco, CA (K.Y.); Kaiser Permanente Division of Research, Oakland, CA (S.S.); Division of Cardiovascular Sciences, National Heart, Lung, and Blood Institute, National Institutes of Health, Bethesda, MD (J.P.R.); and Division of Epidemiology and Community Health, School of Public Health, University of Minnesota, Minneapolis (D.R.J.).

Sidney, Stephen. From the Division of Epidemiology and Population Health Sciences, Department of Public Health Sciences, University of Miami, FL (A.Z.A.H.); Departments of Epidemiology and Biostatistics (E.V., K.Y.), Psychiatry (K.Y.), and Neurology (K.Y.), University of California San Francisco; San Francisco Veterans Affairs Medical Center, San Francisco, CA (K.Y.); Kaiser Permanente Division of Research, Oakland, CA (S.S.); Division of Cardiovascular Sciences, National Heart, Lung, and Blood Institute, National Institutes of Health, Bethesda, MD (J.P.R.); and Division of Epidemiology and Community Health, School of Public Health, University of Minnesota, Minneapolis (D.R.J.).

Reis, Jared P. From the Division of Epidemiology and Population Health Sciences, Department of Public Health Sciences, University of Miami, FL (A.Z.A.H.); Departments of Epidemiology and Biostatistics (E.V., K.Y.), Psychiatry (K.Y.), and Neurology (K.Y.), University of California San Francisco; San Francisco Veterans Affairs Medical Center, San Francisco, CA (K.Y.); Kaiser Permanente Division of Research, Oakland, CA (S.S.); Division of Cardiovascular Sciences, National Heart, Lung, and Blood Institute, National Institutes of Health, Bethesda, MD (J.P.R.); and Division of Epidemiology and Community Health, School of Public Health, University of Minnesota, Minneapolis (D.R.J.).

Jacobs, David R Jr. From the Division of Epidemiology and Population Health Sciences, Department of Public Health Sciences, University of Miami, FL (A.Z.A.H.); Departments of Epidemiology and Biostatistics (E.V., K.Y.), Psychiatry (K.Y.), and Neurology (K.Y.), University of California San Francisco; San Francisco Veterans Affairs Medical Center, San Francisco, CA (K.Y.); Kaiser Permanente Division of Research, Oakland, CA (S.S.); Division of Cardiovascular Sciences, National Heart, Lung, and Blood Institute, National Institutes of Health, Bethesda, MD (J.P.R.); and Division of Epidemiology and Community Health, School of Public Health, University of Minnesota, Minneapolis (D.R.J.).

Yaffe, Kristine. From the Division of Epidemiology and Population Health Sciences, Department of Public Health Sciences, University of Miami, FL (A.Z.A.H.); Departments of Epidemiology and Biostatistics (E.V., K.Y.), Psychiatry (K.Y.), and Neurology (K.Y.), University of California San Francisco; San Francisco Veterans Affairs Medical Center, San Francisco, CA (K.Y.); Kaiser Permanente Division

For peer review only

of Research, Oakland, CA (S.S.); Division of Cardiovascular Sciences, National Heart, Lung, and Blood Institute, National Institutes of Health, Bethesda, MD (J.P.R.); and Division of Epidemiology and Community Health, School of Public Health, University of Minnesota, Minneapolis (D.R.J.).

MeSH Heading

Adult. *Carotid Artery Diseases/dg [Diagnostic Imaging]. Carotid Artery Diseases/px [Psychology]. *Carotid Artery, Common/dg [Diagnostic Imaging]. Carotid Intima-Media Thickness/px [Psychology]. *Carotid Intima-Media Thickness. *Cognition Disorders/dg [Diagnostic Imaging]. Cognition Disorders/px [Psychology]. Cohort Studies. *Coronary Artery Disease/dg [Diagnostic Imaging]. Coronary Artery Disease/px [Psychology]. Female. Humans. Male. Middle Aged. Prospective Studies. Risk Factors. *Stroke/dg [Diagnostic Imaging]. Stroke/px [Psychology]. Young Adult.

Keyword Heading

carotid intima-media thickness   cognition

epidemiology

risk factors

stroke.

Year of Publication

2015

Link to the Ovid Full Text or citation:

Click here for full text options

Link to the External Link Resolver:

SFX

48.

Carotid and vertebral arterial variations in Alzheimer's disease.

Zhou R; Liu D; Yu K; Chen Y; Li L; Xu J; Zhou H.

Current Alzheimer Research. 12(4):368-76, 2015.

[Journal Article]

UI: 25817257

For peer review only

The effects of carotid and vertebral arterial morphological variations on cognitive function impairment remain unclear. We investigated the association between extracranial carotid and vertebral arterial variations and the risk of Alzheimer's disease (AD). A prospective study with a 5-year followup was conducted from July 2008 to June 2013. A total of 1741 subjects (50 years of age and older) were examined for carotid and vertebral arterial variations using computed tomography angiography (CTA) and completed the study follow-up. Variations of the carotid and vertebral arteries were classified as tortuosity, kinking and coiling, according to the Weibel and Fields criteria. Cognitive function was assessed using the Mini-Mental State Examination and the Activities of Daily Living scale. We analyzed the association between arterial variations and the risk of AD by using multivariate Cox proportional-hazards models. The prevalence of carotid arterial variations was 38.4%, and the prevalence of vertebral arterial variations was 86.6%. Among the 1741 subjects who completed the study follow-up, 134 AD cases were detected. The subjects diagnosed with AD displayed greater kinking and coiling in the carotid artery (P<0.01) and vertebral artery (P<0.05) than the subjects without AD. After adjusting for potential confounders, kinking and coiling (hazard ratio [HR]=1.93, 95% confidence interval [CI], 1.37 to 2.86, P<0.01) in the carotid artery were significantly associated with AD. Additionally, after adjusting for potential confounders, kinking and coiling (HR=1.73, 95% CI, 1.25 to 2.31, P<0.01) in the vertebral artery were significantly associated with the risk of AD. We determined that age, hypertension and smoking status were significant predictors of AD in the multivariable models with carotid and vertebral arterial variation. The results of the current study indicate that severe carotid and vertebral arterial variations are associated with a significantly increased risk of AD. Further investigation into the association between these variations and AD would be useful for preventing AD.

Version ID

1

Record Owner

From MEDLINE, a database of the U.S. National Library of Medicine.

Status

MEDLINE

Authors Full Name

Zhou, Rui; Liu, Dong; Yu, Ke; Chen, Yang; Li, Ling; Xu, Jianzhong; Zhou, Huadong.

Institution

Zhou, Huadong. Department of Neurology, Daping hospital, Third Military Medical University, Chongqing, 400038, P.R. China. zhouhuad@163.com.

MeSH Heading

Aged. Aged, 80 and over. Alzheimer Disease/ep [Epidemiology]. *Alzheimer Disease/pa [Pathology]. Angiography. *Carotid Arteries/pa [Pathology]. Carotid Intima-Media Thickness. Disease-Free Survival. Female. Follow-Up Studies. Humans. Incidence. Male. Middle Aged.

For peer review only

Multivariate Analysis. Neuropsychological Tests. Proportional Hazards Models. Sex Characteristics. Tomography, X-Ray Computed. *Vertebral Artery/pa [Pathology].
Year of Publication
2015

Link to the Ovid Full Text or citation:
[Click here for full text options]

Link to the External Link Resolver:
[SFX]

50.

Markers for the risk of progression from mild cognitive impairment to Alzheimer's disease.
Buratti L; Balestrini S; Altamura C; Viticchi G; Falsetti L; Luzzi S; Provinciali L; Vernieri F; Silvestrini M.
Journal of Alzheimer's Disease. 45(3):883-90, 2015.
[Journal Article]
UI: 25633680
BACKGROUND: Defining reliable markers of conversion to dementia could be the first step in order to identify appropriate treatment strategies for mild cognitive impairment (MCI) patients.
OBJECTIVE: To develop a tool able to predict the risk of progression from MCI to Alzheimer's disease (AD).
METHODS: 406 MCI patients were included and followed for a one-year period. Demographic characteristics, vascular risk factors, extent of cerebrovascular lesions, markers of carotid atherosclerosis investigated with an ultrasonographic assessment (plaque index and intima-media thickness) and cerebrovascular reactivity to apnea (breath-holding index) were considered as potential predictors of conversion.
RESULTS: 106 (26%) MCI patients showed a conversion to AD. Plaque index, intima-media thickness, and breath-holding index were relevant predictors of conversion (p = 0.042; p = 0.003; p < 0.001, multivariate logistic regression analysis). A simplified scoring system was devised based on the magnitude of the estimated multinomial logistic regression beta coefficient results. A total score was calculated as the sum of each predictive factor which resulted in a 0-5 range. The optimal cut-off score was >=3 (sensitivity, 23.6%, 95% CI 15.9%-32.8%; specificity, 97.7%, 95% CI

95.3%-99.1%; positive likelihood ratio, 10.1, 95% CI 4.5%-22.7%; negative likelihood ratio, 0.78, 95% CI 0.70%-0.87%). The AUC was 0.71 (95% CI, 0.65-0.77).

CONCLUSIONS: Our findings show the possibility to obtain a predictive indicator of the risk of conversion from MCI to dementia by considering the presence of both atherosclerotic changes in the carotid district and impairment of cerebral hemodynamics. Such an approach may allow us to formulate a correct prognosis in more than 70% of patients with amnesic MCI.

Version ID

1

Record Owner

From MEDLINE, a database of the U.S. National Library of Medicine.

Status

MEDLINE

Authors Full Name

Buratti, Laura; Balestrini, Simona; Altamura, Claudia; Viticchi, Giovanna; Falsetti, Lorenzo; Luzzi, Simona; Provinciali, Leandro; Vernieri, Fabrizio; Silvestrini, Mauro.

Institution

Buratti, Laura. Neurological Clinic, Marche Polytechnic University, Ancona, Italy. Balestrini, Simona. Neurological Clinic, Marche Polytechnic University, Ancona, Italy.

Altamura, Claudia. Neurology Unit, Campus Bio-Medico University, Rome, Italy.

Viticchi, Giovanna. Neurological Clinic, Marche Polytechnic University, Ancona, Italy.

Falsetti, Lorenzo. Internal and Subintensive Medicine, Ospedali Riuniti Ancona, Italy.

Luzzi, Simona. Neurological Clinic, Marche Polytechnic University, Ancona, Italy.

Provinciali, Leandro. Neurological Clinic, Marche Polytechnic University, Ancona, Italy.

Vernieri, Fabrizio. Neurology Unit, Campus Bio-Medico University, Rome, Italy.

Silvestrini, Mauro. Neurological Clinic, Marche Polytechnic University, Ancona, Italy.

MeSH Heading

Aged. Alzheimer Disease/co [Complications]. Alzheimer Disease/ep [Epidemiology]. Alzheimer Disease/px [Psychology]. *Alzheimer Disease. Biomarkers. Carotid Intima-Media Thickness. Cerebrovascular Disorders. Cognition Disorders/ep [Epidemiology]. *Cognition Disorders/et [Etiology]. Cognition Disorders/px [Psychology]. Disease Progression. Female. Humans. Logistic Models. Longitudinal Studies. Male. Neuropsychological Tests. ROC Curve. Risk Factors.

Keyword Heading

Alzheimer's disease atherosclerosis

carotid arteries

cerebral hemodynamics

mild cognitive impairment

ultrasonography.

For peer review only

Registry Number/Name of Substance

0 (Biomarkers).

Year of Publication

2015

Link to the Ovid Full Text or citation:

[Click here for full text options]

Link to the External Link Resolver:

[SFX]

51.

Association of postural instability with asymptomatic cerebrovascular damage and cognitive decline: the Japan Shimanami health promoting program study.

Tabara Y; Okada Y; Ohara M; Uetani E; Kido T; Ochi N; Nagai T; Igase M; Miki T; Matsuda F; Kohara K.

Stroke. 46(1):16-22, 2015 Jan.

[Journal Article. Research Support, Non-U.S. Gov't]

UI: 25523051

BACKGROUND AND PURPOSE: Asymptomatic cerebral small-vessel disease (cSVD) in elderly individuals are potent risk factors for stroke. In addition to common clinical risk factors, postural instability has been postulated to be associated with cSVD in older frail patients. Here, we conducted a cross-sectional study to understand the possible link between postural instability and asymptomatic cSVD further, namely periventricular hyperintensity, lacunar infarction, and microbleeds, as well as cognitive function, in a middle-aged to elderly general population (n=1387).

METHODS: Postural instability was assessed based on one-leg standing time (OLST) and posturography findings. cSVD was evaluated by brain MRI. Mild cognitive impairment was assessed using a computer-based questionnaire, and carotid intima-media thickness as an index of atherosclerosis was measured via ultrasonography.

RESULTS: Frequency of short OLST, in particular <20 s, increased linearly with severity of cSVD (lacunar infarction lesion: none, 9.7%; 1, 16.0%; >2, 34.5%; microbleeds lesion: none, 10.1%; 1, 15.3%;

For peer review only

For peer review only - http://bmjopen.bmj.com/site/about/guidelines.xhtml

>2, 30.0%; periventricular hyperintensity grade: 0, 5.7%; 1, 11.5%; >2, 23.7%). The association of short OLST with lacunar infarction and microbleeds but not periventricular hyperintensity remained significant even after adjustment for possible covariates (lacunar infarction, P=0.009; microbleeds, P=0.003; periventricular hyperintensity, P=0.601). In contrast, no significant association was found between posturographic parameters and cSVD, whereas these parameters were linearly associated with OLST. Short OLST was also significantly associated with reduced cognitive function independent of covariates, including cSVD (P=0.002).

CONCLUSIONS: Postural instability was found to be associated with early pathological changes in the brain and functional decline, even in apparently healthy subjects.

Copyright © 2014 American Heart Association, Inc.

Version ID

1

Record Owner

From MEDLINE, a database of the U.S. National Library of Medicine.

Status

MEDLINE

Authors Full Name

Tabara, Yasuharu; Okada, Yoko; Ohara, Maya; Uetani, Eri; Kido, Tomoko; Ochi, Namiko; Nagai, Tokihisa; Igase, Michiya; Miki, Tetsuro; Matsuda, Fumihiko; Kohara, Katsuhiko.

Institution

Tabara, Yasuharu. From the Center for Genomic Medicine, Kyoto University Graduate School of Medicine, Sakyo-ku, Kyoto, Japan (Y.T., T.M., F.M.); and Department of Geriatric Medicine, Ehime University Graduate School of Medicine, Toon City, Ehime, Japan (Y.T., Y.O., M.O., E.U., T.K., N.O., T.N., M.I., T.M., K.K.). tabara@genome.med.kyoto-u.ac.jp. Okada, Yoko. From the Center for Genomic Medicine, Kyoto University Graduate School of Medicine, Sakyo-ku, Kyoto, Japan (Y.T., T.M., F.M.); and Department of Geriatric Medicine, Ehime University Graduate School of Medicine, Toon City, Ehime, Japan (Y.T., Y.O., M.O., E.U., T.K., N.O., T.N., M.I., T.M., K.K.).

Ohara, Maya. From the Center for Genomic Medicine, Kyoto University Graduate School of Medicine, Sakyo-ku, Kyoto, Japan (Y.T., T.M., F.M.); and Department of Geriatric Medicine, Ehime University Graduate School of Medicine, Toon City, Ehime, Japan (Y.T., Y.O., M.O., E.U., T.K., N.O., T.N., M.I., T.M., K.K.).

Uetani, Eri. From the Center for Genomic Medicine, Kyoto University Graduate School of Medicine, Sakyo-ku, Kyoto, Japan (Y.T., T.M., F.M.); and Department of Geriatric Medicine, Ehime University Graduate School of Medicine, Toon City, Ehime, Japan (Y.T., Y.O., M.O., E.U., T.K., N.O., T.N., M.I., T.M., K.K.).

Kido, Tomoko. From the Center for Genomic Medicine, Kyoto University Graduate School of Medicine, Sakyo-ku, Kyoto, Japan (Y.T., T.M., F.M.); and Department of Geriatric Medicine, Ehime

For peer review only

University Graduate School of Medicine, Toon City, Ehime, Japan (Y.T., Y.O., M.O., E.U., T.K., N.O., T.N., M.I., T.M., K.K.).

Ochi, Namiko. From the Center for Genomic Medicine, Kyoto University Graduate School of Medicine, Sakyo-ku, Kyoto, Japan (Y.T., T.M., F.M.); and Department of Geriatric Medicine, Ehime University Graduate School of Medicine, Toon City, Ehime, Japan (Y.T., Y.O., M.O., E.U., T.K., N.O., T.N., M.I., T.M., K.K.).

Nagai, Tokihisa. From the Center for Genomic Medicine, Kyoto University Graduate School of Medicine, Sakyo-ku, Kyoto, Japan (Y.T., T.M., F.M.); and Department of Geriatric Medicine, Ehime University Graduate School of Medicine, Toon City, Ehime, Japan (Y.T., Y.O., M.O., E.U., T.K., N.O., T.N., M.I., T.M., K.K.).

Igase, Michiya. From the Center for Genomic Medicine, Kyoto University Graduate School of Medicine, Sakyo-ku, Kyoto, Japan (Y.T., T.M., F.M.); and Department of Geriatric Medicine, Ehime University Graduate School of Medicine, Toon City, Ehime, Japan (Y.T., Y.O., M.O., E.U., T.K., N.O., T.N., M.I., T.M., K.K.).

Miki, Tetsuro. From the Center for Genomic Medicine, Kyoto University Graduate School of Medicine, Sakyo-ku, Kyoto, Japan (Y.T., T.M., F.M.); and Department of Geriatric Medicine, Ehime University Graduate School of Medicine, Toon City, Ehime, Japan (Y.T., Y.O., M.O., E.U., T.K., N.O., T.N., M.I., T.M., K.K.).

Matsuda, Fumihiko. From the Center for Genomic Medicine, Kyoto University Graduate School of Medicine, Sakyo-ku, Kyoto, Japan (Y.T., T.M., F.M.); and Department of Geriatric Medicine, Ehime University Graduate School of Medicine, Toon City, Ehime, Japan (Y.T., Y.O., M.O., E.U., T.K., N.O., T.N., M.I., T.M., K.K.).

Kohara, Katsuhiko. From the Center for Genomic Medicine, Kyoto University Graduate School of Medicine, Sakyo-ku, Kyoto, Japan (Y.T., T.M., F.M.); and Department of Geriatric Medicine, Ehime University Graduate School of Medicine, Toon City, Ehime, Japan (Y.T., Y.O., M.O., E.U., T.K., N.O., T.N., M.I., T.M., K.K.).

Comments

Comment in (CIN)   Erratum in (EIN)

MeSH Heading

Aged. Asymptomatic Diseases. *Brain/pa [Pathology]. Carotid Artery Diseases/dg [Diagnostic Imaging]. *Carotid Artery Diseases/ep [Epidemiology]. Carotid Intima-Media Thickness. *Cerebral Hemorrhage/ep [Epidemiology]. Cerebral Hemorrhage/pa [Pathology]. Cerebral Small Vessel Diseases/ep [Epidemiology]. Cerebral Small Vessel Diseases/pa [Pathology]. Cerebrovascular Disorders/ep [Epidemiology]. Cerebrovascular Disorders/pa [Pathology]. Cognition Disorders/ep [Epidemiology]. Cognition Disorders/pa [Pathology]. *Cognitive Dysfunction/ep [Epidemiology]. Cognitive Dysfunction/pa [Pathology]. Cross-Sectional Studies. Female. Humans. Japan/ep

For peer review only

[Epidemiology]. Magnetic Resonance Imaging. Male. Middle Aged. *Postural Balance. *Sensation Disorders/ep [Epidemiology]. *Stroke, Lacunar/ep [Epidemiology]. Stroke, Lacunar/pa [Pathology].

Keyword Heading

lacunar stroke.

Year of Publication

2015

Link to the Ovid Full Text or citation:

Click here for full text options

Link to the External Link Resolver:

SFX

52.

Carotid plaque as a predictor of dementia in older adults: the Three-City Study.

Carcaillon L; Plichart M; Zureik M; Rouaud O; Majed B; Ritchie K; Tzourio C; Dartigues JF; Empana JP.

Alzheimer's & Dementia. 11(3):239-48, 2015 Mar.

[Journal Article. Multicenter Study. Research Support, Non-U.S. Gov't]

UI: 25510384

BACKGROUND: The contribution of carotid atherosclerosis to incident dementia remains unclear. We examined the association between carotid plaques (CP) and common carotid intima media thickness (CCA-IMT) with incident dementia and its subtypes, and their added value for dementia risk prediction.

METHODS: At baseline, 6025 dementia-free subjects aged 65-86 years underwent bilateral carotid ultrasonography measures of CP and plaque-free CCA-IMT. Subjects were followed-up over 7 years for the detection of dementia.

RESULTS: After a mean 5.4 years of follow-up, 421 subjects developed dementia including 272 Alzheimer's disease and 83 vascular/mixed dementia (VaD). Only CP were independently related to VaD (HR(>=2 sites with plaques) = 1.92; 95% confidence interval or CI = 1.13-3.22) and improved VaD risk prediction (continuous Net Reclassification Index = 30.1%; 95% CI = 8.4-51.7) beyond

For peer review only - http://bmjopen.bmj.com/site/about/guidelines.xhtml

known dementia risk factors. Accounting for stroke or competing risk by death marginally modified the results.

CONCLUSION: In older adults, CP are independent predictors of incident VaD and may improve VaD risk prediction.

Copyright © 2015 The Alzheimer's Association. Published by Elsevier Inc. All rights reserved.

Version ID

1

Record Owner

From MEDLINE, a database of the U.S. National Library of Medicine.

Status

MEDLINE

Authors Full Name

Carcaillon, Laure; Plichart, Matthieu; Zureik, Mahmoud; Rouaud, Olivier; Majed, Bilal; Ritchie, Karen; Tzourio, Christophe; Dartigues, Jean-Francois; Empana, Jean-Philippe.

Institution

Carcaillon, Laure. Inserm, CESP Centre for Research in Epidemiology and Population Health, UMR-S1018, Hormones and Cardiovascular Disease, University Paris Sud, Villejuif, France. Plichart, Matthieu. Inserm, UMR-S 970, Paris Descartes University, Sorbonne Paris Cite, Paris Cardiovascular Research Center, Paris, France; Assistance Publique - Hopitaux de Paris, Hopital Broca, Paris, France. Electronic address: matthieu.plichart@inserm.fr.

Zureik, Mahmoud. Inserm U700, Paris, France.

Rouaud, Olivier. CMMR CHU Dijon, Dijon, France.

Majed, Bilal. Inserm, UMR-S 970, Paris Descartes University, Sorbonne Paris Cite, Paris Cardiovascular Research Center, Paris, France; Epidemiology and Clinical Research Unit, Arras General Hospital, Arras, France.

Ritchie, Karen. Inserm U1061, Neuropsychiatry: Epidemiological and Clinical Research, Hopital La Colombiere, Montpellier, France; Imperial College, Faculty of Medicine, London, United Kingdom; University of Montpellier 1, Faculty of Medicine, Montpellier, France.

Tzourio, Christophe. Inserm U708, Neuroepidemiology, Bordeaux, France; University of Victor Segalen Bordeaux2, Bordeaux, France.

Dartigues, Jean-Francois. University of Victor Segalen Bordeaux2, Bordeaux, France; Inserm U897, Epidemiology and Neuropsychology of Brain Aging, Bordeaux, France.

Empana, Jean-Philippe. Inserm, UMR-S 970, Paris Descartes University, Sorbonne Paris Cite, Paris Cardiovascular Research Center, Paris, France.

MeSH Heading

Aged. Aged, 80 and over. *Carotid Arteries/dg [Diagnostic Imaging]. *Carotid Artery Diseases/dg [Diagnostic Imaging]. Carotid Artery Diseases/ep [Epidemiology]. Carotid Intima-Media Thickness.

For peer review only

*Dementia/di [Diagnosis]. *Dementia/dg [Diagnostic Imaging]. Dementia/ep [Epidemiology]. Female. Follow-Up Studies. Humans. Incidence. Male. *Plaque, Atherosclerotic/dg [Diagnostic Imaging]. Plaque, Atherosclerotic/ep [Epidemiology]. Prognosis. Prospective Studies. Risk. Risk Factors. Sensitivity and Specificity.

Keyword Heading

Aging Atherosclerosis

Dementia

Epidemiology

Risk factors.

Year of Publication

2015

Link to the Ovid Full Text or citation:

Click here for full text options

Link to the External Link Resolver:

SFX

53.

Atherosclerotic calcification is related to a higher risk of dementia and cognitive decline.

Bos D; Vernooij MW; de Bruijn RF; Koudstaal PJ; Hofman A; Franco OH; van der Lugt A; Ikram MA.

Alzheimer's & Dementia. 11(6):639-47.e1, 2015 Jun.

[Journal Article. Research Support, Non-U.S. Gov't]

UI: 25150731

BACKGROUND: Longitudinal data on the role of atherosclerosis in different vessel beds in the etiology of cognitive impairment and dementia are scarce and inconsistent.

METHODS: Between 2003-2006, 2364 nondemented persons underwent computed tomography of the coronaries, aortic arch, extracranial, and intracranial carotid arteries to quantify atherosclerotic calcification. Participants were followed for incident dementia (n = 90) until April 2012. At baseline and follow-up participants also underwent a cognitive test battery.

RESULTS: Larger calcification volume in all vessels, except in the coronaries, was associated with a higher risk of dementia. After adjustment for relevant confounders, extracranial carotid artery

For peer review only

calcification remained significantly associated with a higher risk of dementia [hazard ratio per standard deviation increase in calcification volume: 1.37 (1.05, 1.79)]. Additional analyses for Alzheimer's disease only or censoring for stroke showed similar results. Larger calcification volumes were also associated with cognitive decline.

CONCLUSIONS: Atherosclerosis, in particular in the extracranial carotid arteries, is related to a higher risk of dementia and cognitive decline.

Copyright © 2015 The Alzheimer's Association. Published by Elsevier Inc. All rights reserved.

Version ID

1

Record Owner

From MEDLINE, a database of the U.S. National Library of Medicine.

Status

MEDLINE

Authors Full Name

Bos, Daniel; Vernooij, Meike W; de Bruijn, Renee F A G; Koudstaal, Peter J; Hofman, Albert; Franco, Oscar H; van der Lugt, Aad; Ikram, M Arfan.

Institution

Bos, Daniel. Department of Radiology, Erasmus Medical Center, Rotterdam, The Netherlands; Department of Epidemiology, Erasmus Medical Center, Rotterdam, The Netherlands. Vernooij, Meike W. Department of Radiology, Erasmus Medical Center, Rotterdam, The Netherlands; Department of Epidemiology, Erasmus Medical Center, Rotterdam, The Netherlands.

de Bruijn, Renee F A G. Department of Epidemiology, Erasmus Medical Center, Rotterdam, The Netherlands; Department of Neurology, Erasmus Medical Center, Rotterdam, The Netherlands.

Koudstaal, Peter J. Department of Neurology, Erasmus Medical Center, Rotterdam, The Netherlands.

Hofman, Albert. Department of Epidemiology, Erasmus Medical Center, Rotterdam, The Netherlands.

Franco, Oscar H. Department of Epidemiology, Erasmus Medical Center, Rotterdam, The Netherlands.

van der Lugt, Aad. Department of Radiology, Erasmus Medical Center, Rotterdam, The Netherlands.

Ikram, M Arfan. Department of Radiology, Erasmus Medical Center, Rotterdam, The Netherlands; Department of Epidemiology, Erasmus Medical Center, Rotterdam, The Netherlands; Department of Neurology, Erasmus Medical Center, Rotterdam, The Netherlands. Electronic address: m.a.ikram@erasmusmc.nl.

MeSH Heading

For peer review only

Aged. Aorta, Thoracic/dg [Diagnostic Imaging]. Atherosclerosis/dg [Diagnostic Imaging]. *Atherosclerosis/ep [Epidemiology]. Atherosclerosis/px [Psychology]. Calcinosis/dg [Diagnostic Imaging]. *Calcinosis/ep [Epidemiology]. Calcinosis/px [Psychology]. Carotid Arteries/dg [Diagnostic Imaging]. Cognition Disorders/dg [Diagnostic Imaging]. *Cognition Disorders/ep [Epidemiology]. Coronary Vessels/dg [Diagnostic Imaging]. Dementia/dg [Diagnostic Imaging]. *Dementia/ep [Epidemiology]. Female. Follow-Up Studies. Humans. Longitudinal Studies. Male. Netherlands/ep [Epidemiology]. Prospective Studies. Risk. Stroke/dg [Diagnostic Imaging]. Stroke/ep [Epidemiology]. Stroke/px [Psychology]. Tomography, X-Ray Computed.

Keyword Heading

Arterial calcification Atherosclerosis

Cognitive decline

Dementia

Epidemiology

Imaging.

Year of Publication

2015

Link to the Ovid Full Text or citation:

Click here for full text options

Link to the External Link Resolver:

SFX

55.

The role of carotid intima-media thickness in predicting longitudinal cognitive function in an older adult cohort.

Frazier DT; Seider T; Bettcher BM; Mack WJ; Jastrzab L; Chao L; Weiner MW; DeCarli C; Reed BR; Mungas D; Chui HC; Kramer JH.

Cerebrovascular Diseases. 38(6):441-7, 2014.

[Journal Article. Research Support, N.I.H., Extramural]

UI: 25502351

For peer review only

BACKGROUND AND PURPOSE: Carotid atherosclerosis is a risk factor for cerebrovascular disease in older adults. Although age-related cognitive decline has been associated with cerebrovascular disease, not much is known about the consequences of carotid atherosclerosis on longitudinal cognitive function. This study examines the longitudinal relationship between atherosclerosis and cognition in a sample of non-demented older subjects using baseline measurements of carotid intima media thickness (CIMT) and annual cognitive measures of executive function (EXEC) and verbal memory (MEM).
METHODS: Baseline measurements included CIMT derived from B-mode carotid artery ultrasound, structural T1-weighted images of white matter hypointensities (WMH), white matter lesions (WML), and cerebral infarct. Hypertension, low-density lipoprotein (LDL), diabetes, and waist to hip ratios (WHR) were included as covariates in our models to control for cerebrovascular risks and central adiposity. Annual composite scores of EXEC and MEM functions were derived from item response theory. Linear mixed models were used to model longitudinal cognitive change.
RESULTS: A significant inverse relationship was found between baseline CIMT and annual EXEC score, but not annual MEM score. Subjects included in the highest 4th quartile of CIMT showed a rate of annual decline in EXEC score that was significant relative to subjects in lower quartile groups ($p<0.01$). The relationship between the 4th quartile of CIMT and annual EXEC score remained significant after independently adjusting for imaging measures of white matter injury and cerebral infarct.
CONCLUSIONS: Older adult subjects with the highest index of CIMT showed an annual decline in EXEC scores that was significant relative to subjects with lower quartile measurements of CIMT, independent of our measures of white matter injury and cerebral infarct. Our findings suggest that elevated measures of CIMT may mark an atherosclerotic state, resulting in a decline in executive function and not memory in non-demented older adults.
Copyright © 2014 S. Karger AG, Basel.
Version ID
1
Record Owner
From MEDLINE, a database of the U.S. National Library of Medicine.
Status
MEDLINE
Authors Full Name
Frazier, Darvis T; Seider, Talia; Bettcher, Brianne M; Mack, Wendy J; Jastrzab, Laura; Chao, Linda; Weiner, Michael W; DeCarli, Charles; Reed, Bruce R; Mungas, Dan; Chui, Helena C; Kramer, Joel H.
Institution
Frazier, Darvis T. Memory and Aging Center, San Francisco, Calif., USA.
MeSH Heading

For peer review only

Aged. Aged, 80 and over. Brain/pa [Pathology]. Carotid Artery Diseases/dg [Diagnostic Imaging]. *Carotid Artery Diseases/ep [Epidemiology]. *Carotid Intima-Media Thickness. *Cognition. *Cognition Disorders/ep [Epidemiology]. Cognition Disorders/pa [Pathology]. Executive Function. Female. Humans. Longitudinal Studies. Magnetic Resonance Imaging. Male. Memory. Middle Aged. Neuropsychological Tests. Prospective Studies. *White Matter/pa [Pathology].

Year of Publication

2014

Link to the Ovid Full Text or citation:

Click here for full text options

Link to the External Link Resolver:

SFX

59.

Differential impact of subclinical carotid artery disease on cerebral structure and functioning in type 1 diabetes patients with versus those without proliferative retinopathy.

van Duinkerken E; Ijzerman RG; van der Zijl NJ; Barkhof F; Pouwels PJ; Schoonheim MM; Moll AC; Boerop J; Wessels AM; Klein M; Snoek FJ; Diamant M.

Cardiovascular Diabetology. 13:58, 2014 Mar 12.

[Comparative Study. Journal Article. Research Support, Non-U.S. Gov't]

UI: 24620788

BACKGROUND: Type 1 diabetes mellitus (T1DM) is associated with cerebral compromise, typically found in patients with microangiopathy. Associations between subclinical macroangiopathy and the brain, whether or not in the presence of microangiopathy, have not been fully explored in T1DM. We hypothesized that subclinical macroangiopathy in adult T1DM may affect the brain and interacts with microangiopathy.

METHODS: In 51 asymptomatic T1DM patients with, 53 without proliferative retinopathy and 51 controls, right common carotid artery ultrasound was used to assess intima media thickness (cIMT) and distensibility (cD). Neuropsychological tests for cognitive functions, and magnetic resonance imagining for white matter integrity and functional connectivity, i.e. neuronal communication, were used.

For peer review only

RESULTS: After correction for confounders, cIMT was borderline significantly increased in all T1DM patients (P = 0.071), whereas cD was not statistically significantly altered (P = 0.45). Patients with proliferative retinopathy showed the largest increase in cIMT and decrease in cD. In all participants, after adjustment for confounders, increased cIMT was related to decreased white matter integrity (beta = -0.198 P = 0.041) and decreased functional connectivity in visual areas (beta = -0.195 P = 0.046). For cognition, there was a significant interaction between cIMT and the presence of proliferative retinopathy after adjustment for confounding factors (all P < 0.05). Increased cIMT was associated with lower general cognitive ability (beta = -0.334; P = 0.018), information processing speed (beta = -0.361; P = 0.010) and attention (beta = -0.394; P = 0.005) scores in patients without, but not in patients with proliferative retinopathy.
CONCLUSIONS: These findings suggest that subclinical macroangiopathy may be a factor in the development of diabetes-related cognitive changes in uncomplicated T1DM, whereas in patients with advanced T1DM, proliferative retinopathy may rather be the driving force of cerebral compromise.
Version ID
1
Record Owner
From MEDLINE, a database of the U.S. National Library of Medicine.
Status
MEDLINE
Authors Full Name
van Duinkerken, Eelco; Ijzerman, Richard G; van der Zijl, Nynke J; Barkhof, Frederik; Pouwels, Petra J W; Schoonheim, Menno M; Moll, Annette C; Boerop, Jeannette; Wessels, Alette M; Klein, Martin; Snoek, Frank J; Diamant, Michaela.
Institution
van Duinkerken, Eelco. Diabetes Center/Department of Internal Medicine, VU University Medical Center, De Boelelaan 1117 - Room MF-G417, 1081 HV, Amsterdam, The Netherlands. e.vanduinkerken@vumc.nl.
MeSH Heading
Adolescent. Adult. *Carotid Artery Diseases/dg [Diagnostic Imaging]. Carotid Artery Diseases/ep [Epidemiology]. Carotid Artery Diseases/pp [Physiopathology]. *Carotid Intima-Media Thickness. Cell Proliferation. *Cerebrovascular Circulation/ph [Physiology]. Cross-Sectional Studies. *Diabetes Mellitus, Type 1/dg [Diagnostic Imaging]. Diabetes Mellitus, Type 1/ep [Epidemiology]. Diabetes Mellitus, Type 1/pp [Physiopathology]. *Diabetic Retinopathy/dg [Diagnostic Imaging]. Diabetic Retinopathy/ep [Epidemiology]. Diabetic Retinopathy/pp [Physiopathology]. Female. Humans. Male. Middle Aged. Single-Blind Method. Young Adult.
Year of Publication

For peer review only - http://bmjopen.bmj.com/site/about/guidelines.xhtml

2014

Link to the Ovid Full Text or citation:

Click here for full text options

Link to the External Link Resolver:

SFX

60.

Visit-to-visit blood pressure variability in the elderly: associations with cognitive impairment and carotid artery remodeling.

Nagai M; Hoshide S; Nishikawa M; Masahisa S; Kario K.

Atherosclerosis. 233(1):19-26, 2014 Mar.

[Journal Article]

UI: 24529116

OBJECTIVE: Recently, visit-to-visit blood pressure (BP) variability has been shown to be associated with vascular remodeling and cognitive dysfunction. However, there have been no studies that focused on the relationship between visit-to-visit BP variability and cognitive dysfunction in relation to vascular remodeling. In this study, we investigated the relationships among visit-to-visit BP measures, carotid artery remodeling and cognitive function in the elderly at high risk of cardiovascular disease.

METHODS: The cognitive function was evaluated using a Mini-Mental State Examination (MMSE) and global deterioration scale (GDS) in 201 elderly subjects at high risk of cardiovascular disease (79.9 +/- 6.4 years old; female 75%). Based on 12 visits (once a month), visit-to-visit BP variability (expressed as the coefficient of variation [CV] and as delta [maximum--minimum] BP) were measured. Carotid ultrasound was performed to measure intima-media thickness (IMT) and the stiffness parameter beta.

RESULTS: The patients having both high delta systolic BP (SBP) and high IMT had significantly higher prevalence of low MMSE score than those with both low delta SBP and low IMT ($p < 0.05$), and the patients having both high delta SBP and high stiffness parameter beta also had significantly higher prevalence of low MMSE score than those with both low delta SBP and low stiffness parameter beta ($p < 0.01$). In the logistic regression analysis adjusted for age, calcium

For peer review only - http://bmjopen.bmj.com/site/about/guidelines.xhtml

channel blocker use, low density lipoprotein, average heart rate, and average SBP level, a significant interaction was found between delta SBP and stiffness parameter beta for the low MMSE score ($p < 0.05$).

CONCLUSIONS: In the high risk elderly, exaggerated visit-to-visit BP variability and advanced carotid artery remodeling have a synergetic association with cognitive dysfunction.

Copyright © 2013 Elsevier Ireland Ltd. All rights reserved.

Version ID

1

Record Owner

From MEDLINE, a database of the U.S. National Library of Medicine.

Status

MEDLINE

Authors Full Name

Nagai, Michiaki; Hoshide, Satoshi; Nishikawa, Mami; Masahisa, Shimpo; Kario, Kazuomi.

Institution

Nagai, Michiaki. Division of Cardiovascular Medicine, Department of Medicine, Jichi Medical University School of Medicine, Yakushiji 3311-1, Shimotsuke, Tochigi 329-0498, Japan. Hoshide, Satoshi. Division of Cardiovascular Medicine, Department of Medicine, Jichi Medical University School of Medicine, Yakushiji 3311-1, Shimotsuke, Tochigi 329-0498, Japan.

Nishikawa, Mami. Division of Cardiovascular Medicine, Department of Medicine, Jichi Medical University School of Medicine, Yakushiji 3311-1, Shimotsuke, Tochigi 329-0498, Japan.

Masahisa, Shimpo. Division of Cardiovascular Medicine, Department of Medicine, Jichi Medical University School of Medicine, Yakushiji 3311-1, Shimotsuke, Tochigi 329-0498, Japan.

Kario, Kazuomi. Division of Cardiovascular Medicine, Department of Medicine, Jichi Medical University School of Medicine, Yakushiji 3311-1, Shimotsuke, Tochigi 329-0498, Japan. Electronic address: kkario@jichi.ac.jp.

MeSH Heading

Aged. Aged, 80 and over. Blood Pressure/de [Drug Effects]. *Blood Pressure. Carotid Arteries/dg [Diagnostic Imaging]. *Carotid Intima-Media Thickness. Cognition Disorders/ep [Epidemiology]. *Cognition Disorders/et [Etiology]. Female. Humans. Japan/ep [Epidemiology]. Male. *Vascular Resistance/ph [Physiology].

Keyword Heading

Carotid artery remodeling   Cognitive impairment

The elderly

Visit-to-visit blood pressure variability.

Year of Publication

2014

For peer review only

Link to the Ovid Full Text or citation:

Click here for full text options

Link to the External Link Resolver:

SFX

61.

Association of cognitive dysfunction with cardiovascular disease events in elderly hypertensive patients.

Yano Y; Bakris GL; Inokuchi T; Ohba Y; Tamaki N; Nagata M; Kuwabara M; Yokota N; Eto T; Kuroki M; Shimada K; Kario K.

Journal of Hypertension. 32(2):423-31, 2014 Feb.

[Journal Article. Research Support, Non-U.S. Gov't]

UI: 24351802

OBJECTIVES: This study assesses whether presence of cognitive dysfunction can be a marker associated with the development of cardiovascular disease (CVD) events independent of ambulatory blood pressure (BP) or other indices of target organ damage (TOD) in elderly hypertensive patients.

METHODS: We recruited 585 hypertensive patients (mean age, 73 years; 41% men) who were ambulatory, lived independently, and were without clinically overt dementia. Cognitive function was assessed by Mini-Mental State Examination (MMSE) at baseline, and CVD events (coronary artery disease, stroke, congestive heart failure, and sudden death) were prospectively ascertained. Cognitive dysfunction was defined as the lowest quartile of MMSE scores (n = 183, median 24 points).

RESULTS: CVD events occurred in 42 people over an average of 2.8 years (1644 person-years). The prevalence of cognitive dysfunction was higher in patients with CVD events than those without (57 vs. 29%; both P <0.001) at baseline. Cognitive dysfunction was associated with CVD events, after adjustment for nocturnal SBP and evidence of TOD [i.e. albuminuria, cardiac hypertrophy, and carotid-artery intima-media thickness (IMT)], hazard ratio 2.5-2.9 (all P <0.01). Incorporation of MMSE in the risk model (including age, estimated glomerular filtration rate, and preexisting CVD) improved the C-statistics (from 0.691 to 0.741) and resulted in a net reclassification improvement of

For peer review only

17.6% (P = 0.02). In contrast, incorporation of albuminuria, cardiac hypertrophy, and high carotid-artery IMT added little further improvement in the risk prediction.

CONCLUSION: Cognitive dysfunction is an independent marker associated with increased risk of CVD events in elderly hypertensive patients.

Version ID

1

Record Owner

From MEDLINE, a database of the U.S. National Library of Medicine.

Status

MEDLINE

Authors Full Name

Yano, Yuichiro; Bakris, George L; Inokuchi, Takashi; Ohba, Yusuke; Tamaki, Noboru; Nagata, Masahiko; Kuwabara, Masachika; Yokota, Naoto; Eto, Takuma; Kuroki, Munetoshi; Shimada, Kazuyuki; Kario, Kazuomi.

Institution

Yano, Yuichiro. aAmerican Society of Hypertension Comprehensive Hypertension Center, Section of Endocrinology, Diabetes and Metabolism, Department of Medicine, University of Chicago Medicine, Chicago, Illinois, USA bDivision of Cardiovascular Medicine, Department of Medicine, Jichi Medical University School of Medicine, Tochigi cDepartment of Orthopedics, Chikamorikai Medical Group, Kochi dTamaki Clinic eKijo Clinic fKuwabara Clinic gYokota Naika hEto Cardiology Clinic iKuroki Naika, Miyazaki, Japan.

MeSH Heading

Aged. Aged, 80 and over. Blood Pressure. *Cardiovascular Diseases/et [Etiology]. Cardiovascular Diseases/pp [Physiopathology]. Cardiovascular Diseases/px [Psychology]. *Cognition Disorders/co [Complications]. Cognition Disorders/px [Psychology]. Cohort Studies. Female. Follow-Up Studies. Humans. *Hypertension/co [Complications]. Hypertension/pp [Physiopathology]. Hypertension/px [Psychology]. Male. Mental Status Schedule. Middle Aged. Prognosis. Proportional Hazards Models. Risk Factors.

Year of Publication

2014

Link to the Ovid Full Text or citation:

Click here for full text options

Link to the External Link Resolver:

SFX

For peer review only - http://bmjopen.bmj.com/site/about/guidelines.xhtml

63.

Correlation of cognitive function with ultrasound strain indices in carotid plaque.

Wang X; Jackson DC; Varghese T; Mitchell CC; Hermann BP; Kliewer MA; Dempsey RJ.

Ultrasound in Medicine & Biology. 40(1):78-89, 2014 Jan.

[Journal Article. Research Support, N.I.H., Extramural]

UI: 24120415

Instability in carotid vulnerable plaque can generate cerebral micro-emboli, which may be related to both stroke and eventual cognitive abnormality. Strain imaging to detect plaque vulnerability based on regions with large strain fluctuations, with arterial pulsation, may be able to determine the risk of cognitive impairment. Plaque instability may be characterized by increased strain variations over a cardiac cycle. Radiofrequency signals for ultrasound strain imaging were acquired from the carotid arteries of 24 human patients using a Siemens Antares with a VFX 13-5 linear array transducer. These patients underwent standardized cognitive assessment (Repeatable Battery for the Assessment of Neuropsychological Status [RBANS]). Plaque regions were segmented by a radiologist at end-diastole using the Medical Imaging Interaction Toolkit. A hierarchical block-matching motion tracking algorithm was used to estimate the cumulated axial, lateral and shear strains within the imaging plane. The maximum, minimum and peak-to-peak strain indices in the plaque computed from the mean cumulated strain over a small region of interest in the plaque with large deformations were obtained. The maximum and peak-to-peak mean cumulated strain indices over the entire plaque region were also computed. All strain indices were then correlated with RBANS Total performance. Overall cognitive performance (RBANS Total) was negatively associated with values of the maximum strain and the peak-to-peak for axial and lateral strains, respectively. There was no significant correlation between the RBANS Total score and shear strain and strain indices averaged over the entire identified plaque for this group of patients. However, correlation of maximum lateral strain was higher for symptomatic patients ($r = -0.650$, $p = 0.006$) than for asymptomatic patients ($r = -0.115$, $p = 0.803$). On the other hand, correlation of maximum axial strain averaged over the entire plaque region was significantly higher for asymptomatic patients ($r = -0.817$, $p = 0.016$) than for symptomatic patients ($r = -0.224$, $p = 0.402$). The results reveal a direct relationship between the maximum axial and lateral strain indices in carotid plaque and cognitive impairment.

Copyright © 2014 World Federation for Ultrasound in Medicine & Biology. Published by Elsevier Inc. All rights reserved.

For peer review only - http://bmjopen.bmj.com/site/about/guidelines.xhtml

Version ID

1

Record Owner

From MEDLINE, a database of the U.S. National Library of Medicine.

Status

MEDLINE

Authors Full Name

Wang, Xiao; Jackson, Daren C; Varghese, Tomy; Mitchell, Carol C; Hermann, Bruce P; Kliewer, Mark A; Dempsey, Robert J.

Institution

Wang, Xiao. Department of Medical Physics, University of Wisconsin-Madison, Madison, Wisconsin, USA.

MeSH Heading

Adult. Aged. *Algorithms. *Carotid Stenosis/co [Complications]. *Carotid Stenosis/dg [Diagnostic Imaging]. Carotid Stenosis/pp [Physiopathology]. *Cognition Disorders/dg [Diagnostic Imaging]. *Cognition Disorders/et [Etiology]. Cognition Disorders/pp [Physiopathology]. Elastic Modulus. Elasticity Imaging Techniques/mt [Methods]. Female. Humans. Image Enhancement/mt [Methods]. *Image Interpretation, Computer-Assisted/mt [Methods]. Male. Middle Aged. Plaque, Atherosclerotic/co [Complications]. *Plaque, Atherosclerotic/dg [Diagnostic Imaging]. Prognosis. Reproducibility of Results. Risk Assessment. Sensitivity and Specificity. Statistics as Topic. Stress, Mechanical.

Keyword Heading

Carotid plaque   Displacement

Elasticity imaging

Elastography

Motion tracking

Multi-level

Strain

Vascular cognitive dementia.

Year of Publication

2014

Link to the Ovid Full Text or citation:

Click here for full text options

Link to the External Link Resolver:

For peer review only - http://bmjopen.bmj.com/site/about/guidelines.xhtml

For peer review only

SFX

65.

Albuminuria and carotid atherosclerosis as predictors of cognitive function in a general population.

Rogne SO; Solbu MD; Arntzen KA; Herder M; Mathiesen EB; Schirmer H.

European Neurology. 70(5-6):340-8, 2013.

[Journal Article]

UI: 24158160

BACKGROUND/AIMS: Albuminuria and carotid atherosclerosis are predictors of cardiovascular disease and potential predictors of cognitive decline. Our aim was to study whether albuminuria was an early predictor of cognitive function independent of carotid atherosclerosis in a general population.

METHODS: The study population comprised 1,577 adults without self-reported stroke. In 1994 and 2007 all were screened for cardiovascular risk factors, urinary albumin-creatinine ratio (ACR), carotid intima-media thickness and carotid total plaque area (TPA). Endpoints were neuropsychological test results in 2007 from the digit symbol test, the finger-tapping test, the Mini Mental Status Examination and the 12-word test parts 1 and 2. Multivariate linear regression was used to assess associations.

RESULTS: Higher ACR, DELTAACR, intima-media thickness, TPA and DELTATPA independently predicted a lower score on the digit symbol test. Higher DELTAACR and DELTATPA predicted a lower score on the finger-tapping test. Higher TPA predicted a lower score on the 12-word test part 1 (immediate recall). Smoking predicted lower scores on the digit symbol and finger-tapping tests independent of albuminuria and carotid atherosclerosis.

CONCLUSIONS: Our results suggest that albuminuria, carotid atherosclerosis and smoking are independent predictors of executive function and motor tempo.

Copyright © 2013 S. Karger AG, Basel.

Version ID

1

Record Owner

From MEDLINE, a database of the U.S. National Library of Medicine.

Status

MEDLINE

Authors Full Name

For peer review only - http://bmjopen.bmj.com/site/about/guidelines.xhtml

Rogne, Sigbjorn O; Solbu, Marit D; Arntzen, Kjell Arne; Herder, Marit; Mathiesen, Ellisiv B; Schirmer, Henrik.
Institution
Rogne, Sigbjorn O. Department of Clinical Medicine, University of Tromso, Tromso, Norway.
MeSH Heading
Albuminuria/dg [Diagnostic Imaging]. *Albuminuria/pp [Physiopathology]. Carotid Artery Diseases/dg [Diagnostic Imaging]. *Carotid Artery Diseases/pp [Physiopathology]. Carotid Intima-Media Thickness/px [Psychology]. *Carotid Intima-Media Thickness. *Cognition/ph [Physiology]. *Cognition Disorders/di [Diagnosis]. Cognition Disorders/pp [Physiopathology]. Cognition Disorders/px [Psychology]. Female. Humans. Male. Middle Aged. Neuropsychological Tests. Predictive Value of Tests. Risk Factors.
Year of Publication
2013

Link to the Ovid Full Text or citation:
Click here for full text options

Link to the External Link Resolver:
SFX

66.

Asymptomatic cervicocerebral atherosclerosis, intracranial vascular resistance and cognition: the AsIA-neuropsychology study.
Lopez-Oloriz J; Lopez-Cancio E; Arenillas JF; Hernandez M; Jimenez M; Dorado L; Barrios M; Soriano-Raya JJ; Miralbell J; Caceres C; Fores R; Pera G; Davalos A; Mataro M.
Atherosclerosis. 230(2):330-5, 2013 Oct.
[Journal Article. Research Support, Non-U.S. Gov't]
UI: 24075765
BACKGROUND AND PURPOSE: Carotid atherosclerosis has emerged as a relevant contributor to cognitive impairment and dementia whereas the role of intracranial stenosis and vascular resistance in cognition remains unknown. This study aims to assess the association of

For peer review only - http://bmjopen.bmj.com/site/about/guidelines.xhtml

asymptomatic cervicocerebral atherosclerosis and intracranial vascular resistance with cognitive performance in a large dementia-free population.
METHODS: The Barcelona-AsIA (Asymptomatic Intracranial Atherosclerosis) Neuropsychology Study included 747 Caucasian subjects older than 50 with a moderate-high vascular risk (assessed by REGICOR score) and without history of neither symptomatic vascular disease nor dementia. Extracranial and transcranial color-coded duplex ultrasound examination was performed to assess carotid intima-media thickness (IMT), presence of carotid plaques (ECAD group), intracranial stenosis (ICAD group), and middle cerebral artery pulsatility index (MCA-PI) as a measure of intracranial vascular resistance. Neuropsychological assessment included tests in three cognitive domains: visuospatial skills and speed, verbal memory and verbal fluency.
RESULTS: In univariate analyses, carotid IMT, ECAD and MCA-PI were associated with lower performance in almost all cognitive domains, and ICAD was associated with poor performance in some visuospatial and verbal cognitive tests. After adjustment for age, sex, vascular risk score, years of education and depressive symptoms, ECAD remained associated with poor performance in the three cognitive domains and elevated MCA-PI with worse performance in visuospatial skills and speed.
CONCLUSIONS: Carotid plaques and increased intracranial vascular resistance are independently associated with low cognitive functioning in Caucasian stroke and dementia-free subjects. We failed to find an independent association of intracranial large vessel stenosis with cognitive performance.
Copyright © 2013 Elsevier Ireland Ltd. All rights reserved.
Version ID
1
Record Owner
From MEDLINE, a database of the U.S. National Library of Medicine.
Status
MEDLINE
Authors Full Name
Lopez-Oloriz, Jorge; Lopez-Cancio, Elena; Arenillas, Juan F; Hernandez, Maria; Jimenez, Marta; Dorado, Laura; Barrios, Maite; Soriano-Raya, Juan Jose; Miralbell, Julia; Caceres, Cynthia; Fores, Rosa; Pera, Guillem; Davalos, Antoni; Mataro, Maria.
Institution
Lopez-Oloriz, Jorge. Department of Psychiatry and Clinical Psychobiology, Universitat de Barcelona, 08035 Barcelona, Spain; Institute for Brain, Cognition and Behavior (IR3C), Universitat de Barcelona, 08035 Barcelona, Spain.
MeSH Heading

For peer review only

Aged. Aged, 80 and over. Carotid Arteries/dg [Diagnostic Imaging]. Carotid Arteries/pa [Pathology]. *Carotid Artery Diseases/co [Complications]. Carotid Artery Diseases/pa [Pathology]. Carotid Intima-Media Thickness. *Carotid Stenosis/co [Complications]. Carotid Stenosis/pa [Pathology]. *Cognition/ph [Physiology]. *Cognition Disorders/co [Complications]. Cognition Disorders/pa [Pathology]. Cross-Sectional Studies. Female. Humans. *Intracranial Arteriosclerosis/co [Complications]. Intracranial Arteriosclerosis/pa [Pathology]. Male. Middle Aged. Multivariate Analysis. Neuropsychological Tests. Neuropsychology. Risk Assessment. Stroke/co [Complications]. Stroke/pa [Pathology]. Ultrasonography. *Vascular Resistance.

Keyword Heading

Atherosclerosis Cognitive impairment

Intima-media thickness

Intracranial stenosis

Pulsatility index.

Year of Publication

2013

Link to the Ovid Full Text or citation:

Click here for full text options

Link to the External Link Resolver:

SFX

67.

Carotid artery atherosclerosis is correlated with cognitive impairment in an elderly urban Chinese non-stroke population.

Xiang J; Zhang T; Yang QW; Liu J; Chen Y; Cui M; Yin ZG; Li L; Wang YJ; Li J; Zhou HD.

Journal of Clinical Neuroscience. 20(11):1571-5, 2013 Nov.

[Journal Article]

UI: 23978769

Carotid artery atherosclerosis may cause increased intima-media thickness (IMT), plaque formation, and vessel stenosis or occlusion. However, the association between carotid artery atherosclerosis and cognitive impairment remains uncertain. This study explored the effects of IMT

For peer review only - http://bmjopen.bmj.com/site/about/guidelines.xhtml

For peer review only

and carotid artery stenosis on cognitive function in an elderly Chinese non-stroke population. A total of 2015 patients were recruited. The IMT of carotid arteries and the presence of plaques and stenosis in carotid arteries were assessed with B-mode ultrasound examination. Cognitive performance was evaluated with neuropsychological tests. The cross-sectional relationships between cognitive performance and carotid wall characteristics were analyzed. Carotid artery atherosclerosis (IMT>1.0) and stenosis were found in 86% and 51% of patients, respectively. Cognitive impairment was found in 356 (17.7%) patients. After adjustment for possible confounders, IMT (odds ratio [OR]=1.96; 95% confidence interval [CI] 1.23-3.16) and hyperdense plaque (OR=4.72; 95% CI 2.56-11.2) were associated with poor cognitive performance. Patients with severe (>=70%) carotid artery stenosis had a lower Mini-Mental State Examination score compared with the mild to modest (40-70%) carotid artery stenosis group. Cognitive performance differed between patients with left and right carotid artery stenosis, but no differences were observed between patients with severe left and right carotid artery stenosis. This study indicates that carotid artery atherosclerosis is correlated with cognitive impairment in the elderly Chinese population. A larger sample size across multiple centers and a longitudinal study are required to further explore the impact of carotid artery atherosclerosis on cognition in the elderly population.
Copyright © 2013 Elsevier Ltd. All rights reserved.

Version ID

1

Record Owner

From MEDLINE, a database of the U.S. National Library of Medicine.

Status

MEDLINE

Authors Full Name

Xiang, Jing; Zhang, Tao; Yang, Qing-Wu; Liu, Juan; Chen, Yong; Cui, Ming; Yin, Ze-Gang; Li, Ling; Wang, Yan-Jiang; Li, Jing; Zhou, Hua-Dong.

Institution

Xiang, Jing. Department of Neurology, Daping Hospital, The Third Military Medical University, Yangzi River Street, Yuzhong District, Chongqing 400042, China.

MeSH Heading

Aged. Aged, 80 and over. Asian Continental Ancestry Group. *Atherosclerosis/co [Complications]. Atherosclerosis/dg [Diagnostic Imaging]. Carotid Intima-Media Thickness. *Carotid Stenosis/co [Complications]. Carotid Stenosis/dg [Diagnostic Imaging]. *Cognition Disorders/co [Complications]. Cognition Disorders/dg [Diagnostic Imaging]. Female. Humans. Male. Neuropsychological Tests.

Keyword Heading

Carotid artery atherosclerosis Cognitive impairment

Intima-media thickness
Mini-mental state examination
Neuropsychological tests
Stenosis.
Year of Publication
2013

Link to the Ovid Full Text or citation:
[Click here for full text options]

Link to the External Link Resolver:
[SFX]

68.

Atherosclerosis and physical functioning in older men, a longitudinal study.
den Ouden ME; Schuurmans MJ; Arts IE; Grobbee DE; Bots ML; van den Beld AW; Lamberts SW; van der Schouw YT.
Journal of Nutrition, Health & Aging. 17(1):97-104, 2013 Jan.
[Journal Article. Research Support, Non-U.S. Gov't]
UI: 23299387
OBJECTIVE: Functional decline is a major threat to independency, progressing into functional limitations and eventually leading to disability. Chronic diseases, especially cardiovascular diseases, are important determinants of functional limitations and disability. Vascular damage exits long before it is clinically manifest and can have adverse effects on health, physical and cognitive functioning. The objective was to investigate the association between non-invasive atherosclerosis measures and physical functioning in older men.
DESIGN: Prospective cohort study.
SETTING: The study was conducted in the general community.
PARTICIPANTS: 195 independently living older men.
MEASUREMENTS: Atherosclerosis was measured by intima media thickness (CIMT) of the common carotid artery using ultrasonography and assessment for presence of atherosclerotic plaques. Physical functioning was measured by isometric handgrip strength and leg extensor strength using

For peer review only - http://bmjopen.bmj.com/site/about/guidelines.xhtml
For peer review only

a hand held dynamometer, lower extremity function using the physical performance score and ability to perform activities of daily life using the modified Stanford health assessment questionnaire. Linear regression analysis was performed to estimate the associations between CIMT or plaques and physical functioning.

RESULTS: After adjustment for confounders, higher baseline CIMT was associated with lower isometric handgrip strength at follow up (betaCIMT = -7.21, 95% CI[-13.64;-0.77]). No other associations were found between CIMT and physical functioning. In addition, no associations were found for the presence of plaques and physical functioning either at baseline, or at follow-up.

CONCLUSION: Atherosclerosis, as measured by higher CIMT, is related to a lower isometric handgrip strength at follow-up, but no further associations with physical functioning were found in this longitudinal study among independently living older men.

Version ID

1

Record Owner

From MEDLINE, a database of the U.S. National Library of Medicine.

Status

MEDLINE

Authors Full Name

den Ouden, M E M; Schuurmans, M J; Arts, I E M A; Grobbee, D E; Bots, M L; van den Beld, A W; Lamberts, S W J; van der Schouw, Y T.

Institution

den Ouden, M E M. Julius Center for Health Sciences and Primary Care, STR 6.131, University Medical Center Utrecht, P.O. Box 85500, 3508 GA Utrecht, The Netherlands.

M.E.M.denOuden@umcutrecht.nl

MeSH Heading

Aged. Aged, 80 and over. *Atherosclerosis/dg [Diagnostic Imaging]. *Atherosclerosis/pp [Physiopathology]. Carotid Artery, Common/dg [Diagnostic Imaging]. Carotid Artery, Common/pp [Physiopathology]. Carotid Intima-Media Thickness. Cognition/ph [Physiology]. Disability Evaluation. Follow-Up Studies. Hand Strength/ph [Physiology]. Humans. Independent Living. Linear Models. Longitudinal Studies. Male. *Motor Activity. Prospective Studies. Risk Factors. Surveys and Questionnaires.

Year of Publication

2013

Link to the Ovid Full Text or citation:

Click here for full text options

For peer review only - http://bmjopen.bmj.com/site/about/guidelines.xhtml

Link to the External Link Resolver:
SFX

70.

Carotid atherosclerosis and prospective risk of dementia.
Wendell CR; Waldstein SR; Ferrucci L; O'Brien RJ; Strait JB; Zonderman AB.
Stroke. 43(12):3319-24, 2012 Dec.
[Journal Article. Research Support, N.I.H., Intramural]
UI: 23103489
BACKGROUND AND PURPOSE: Although vascular risk factors have been implicated in the development of all-cause dementia and Alzheimer disease (AD), few studies have examined the association between subclinical atherosclerosis and prospective risk of dementia.
METHODS: Participants from the Baltimore Longitudinal Study of Aging (n=364; age, 60-95 years; median age, 73; 60% male; 82% white) underwent initial carotid atherosclerosis assessment and subsequently were assessed for dementia and AD annually for up to 14 years (median, 7.0). Cox proportional hazards models predicting all-cause dementia and AD were adjusted for age, sex, race, education, blood pressure, cholesterol, cardiovascular disease, diabetes mellitus, and smoking.
RESULTS: Sixty participants developed dementia, with 53 diagnosed as AD. Raw rates of future dementia and AD among individuals initially in the upper quintile of carotid intimal medial thickness or with bilateral carotid plaque were generally double the rates of individuals with intimal medial thickness in the lower quintiles or no plaque at baseline. Adjusted proportional hazards models revealed >2.5-fold increased risk of dementia and AD among individuals in the upper quintile of carotid intimal medial thickness, and approximately 2.0-fold increased risk of dementia among individuals with bilateral plaque.
CONCLUSIONS: Multiple measures of carotid atherosclerosis are associated with prospective risk of dementia. Individuals in the upper quintile of carotid intimal medial thickness or bilateral carotid plaque were at greatest risk. These findings underscore the possibility that early intervention to reduce atherosclerosis may help delay or prevent onset of dementia and AD.
Version ID
1
Record Owner

For peer review only - http://bmjopen.bmj.com/site/about/guidelines.xhtml
For peer review only

From MEDLINE, a database of the U.S. National Library of Medicine.

Status

MEDLINE

Authors Full Name

Wendell, Carrington R; Waldstein, Shari R; Ferrucci, Luigi; O'Brien, Richard J; Strait, James B; Zonderman, Alan B.

Institution

Wendell, Carrington R. Intramural Research Program, National Institute on Aging, NIH, 251 Bayview Blvd, Baltimore, MD 21224, USA. cwendell@jhmi.edu

MeSH Heading

Aged. Aged, 80 and over. Aging. *Alzheimer Disease/ep [Epidemiology]. Carotid Artery Diseases/dg [Diagnostic Imaging]. *Carotid Artery Diseases/ep [Epidemiology]. *Carotid Intima-Media Thickness/sn [Statistics & Numerical Data]. Carotid Stenosis/dg [Diagnostic Imaging]. *Carotid Stenosis/ep [Epidemiology]. *Dementia/ep [Epidemiology]. Female. Follow-Up Studies. Humans. Incidence. Longitudinal Studies. Male. Middle Aged. Predictive Value of Tests. Proportional Hazards Models. Prospective Studies. Risk Factors. Severity of Illness Index.

Year of Publication

2012

Link to the Ovid Full Text or citation:

Click here for full text options

Link to the External Link Resolver:

SFX

71.

Carotid intima-media thickness is associated with cognitive deficiency in hypertensive patients with elevated central systolic blood pressure.

Dias Eda M; Giollo LT Jr; Martinelli DD; Mazeti C; Junior HM; Vilela-Martin JF; Yugar-Toledo JC.

Cardiovascular Ultrasound. 10:41, 2012 Oct 18.

[Comparative Study. Journal Article]

UI: 23078629

For peer review only - http://bmjopen.bmj.com/site/about/guidelines.xhtml

BACKGROUND: The role of hypertension in the loss of cognitive function is controversial. Relationships between hypertension and increases in cerebral vascular resistance, diffused lesions and multiple lacunar infarcts of the white matter are well known. Thus, the objectives of this study were: to evaluate the relationship between hypertension and cognitive dysfunction (CD), identify risk factors and determine the association between early markers of vascular disease and CD in hypertensive individuals.

METHODS: Two hundred individuals aged between 40 and 80 years old were evaluated in this cross-sectional prospective study. Fifty participants were controls (CT). The remaining 150 hypertensive patients were subdivided into two groups, those with CD (HCD) and those without CD (HNCD). All participants underwent clinical evaluations and biochemical blood tests were performed. CD was investigated using the Mini Mental State Examination (MMSE) following the guidelines for its use in Brazil. The impact of hypertension on the arterial bed was assessed by identifying and measuring changes in the intima-media thickness (IMT) by vascular ultrasonography of the carotid arteries and analyses of the central blood pressure and Augmentation Index by applanation tonometry of the radial artery.

RESULTS: There were no significant differences in the total cholesterol, high-density lipoprotein cholesterol and triglycerides plasma concentrations between the three groups. The serum creatinine and estimated glomerular filtration rate were within normal ranges for all three groups. A significantly lower MMSE score was recorded for the HCD Group compared to the HNCD and CT Groups (p-value < 0.05). The IMT was significantly different between the HNCD and HCD Groups (p-value = 0.0124). A significant difference in the IMT was also observed between hypertensive patients and the CT Group (p-value < 0.0001). Age, low-density cholesterol, high-density cholesterol, triglycerides and IMT increased the Odds Ratio for cognitive dysfunction. The central systolic pressure was significantly higher in the HCD and HNCD Groups compared to CT Group (p-value < 0.0001).

CONCLUSIONS: Hypertensive patients with CD have changes in the vascular morphology characterized by an increased carotid IMT, enhanced atherosclerotic lipid profile and impaired hemodynamic functional manifested by elevated central systolic blood pressure.

Version ID

1

Record Owner

From MEDLINE, a database of the U.S. National Library of Medicine.

Status

MEDLINE

Authors Full Name

Dias, Eros da Mota; Giollo, Luiz Tadeu Jr; Martinelli, Debora Dada; Mazeti, Camila; Junior, Heitor Moreno; Vilela-Martin, Jose Fernando; Yugar-Toledo, Juan Carlos.

For peer review only - http://bmjopen.bmj.com/site/about/guidelines.xhtml

Institution

Dias, Eros da Mota. Hypertension Clinic, Department of Internal Medicine, State Medical School of Sao Jose do Rio Preto, Brazil.

MeSH Heading

Adult. Aged. Aged, 80 and over. *Blood Pressure/ph [Physiology]. Brazil/ep [Epidemiology]. *Carotid Arteries/dg [Diagnostic Imaging]. Carotid Arteries/pp [Physiopathology]. *Carotid Intima-Media Thickness. *Cognition/ph [Physiology]. Cognition Disorders/ep [Epidemiology]. *Cognition Disorders/et [Etiology]. Cognition Disorders/pp [Physiopathology]. Cross-Sectional Studies. Female. Follow-Up Studies. Humans. Hypertension/co [Complications]. *Hypertension/dg [Diagnostic Imaging]. Hypertension/pp [Physiopathology]. Incidence. Male. Middle Aged. Prognosis. Prospective Studies. Risk Factors. Severity of Illness Index. Systole.

Year of Publication

2012

Link to the Ovid Full Text or citation:

Click here for full text options

Link to the External Link Resolver:

SFX

72.

Vascular risk factors in Alzheimer's disease - preliminary report.

Stefanova E; Pavlovic A; Jovanovic Z; Veselinovic N; Despotovic I; Stojkovic T; Sternic N; Kostic V.

Journal of the Neurological Sciences. 322(1-2):166-9, 2012 Nov 15.

[Journal Article. Research Support, Non-U.S. Gov't]

UI: 22938734

BACKGROUND: The vascular risk factors are associated with an increased risk for vascular cognitive decline (VCD), but also with Alzheimer disease (AD).

OBJECTIVE: To investigate vascular risk factors in relation to AD and VCD, with a non-invasive neurosonological methods in a clinical settings.

RESULTS: A total of 296 patients with AD and 237 patients with VCD were included in the study. Hypertension, hyperlipidemia, diabetes mellitus, stroke, and white matter changes ($p<0.001$) were

For peer review only

significantly more prevalent in VCD, although they were also present in AD patients. No statistically significant differences were obtained between groups regarding coronary disease, atrial fibrillation, average degree of carotid artery stenosis and carotid intima-media thickness (cITM). However, the patients with AD had carotid artery stenosis ">50%" (p=0.007) and present plaques (p<0.001) more frequently compared to vascular group. The significant associations between robust cognitive measure and vascular factors, diabetes mellitus, carotid stenosis, cITM, and type of plaques were identified only in VCD, but not in AD group.

CONCLUSIONS: The vascular risk factors were more prevalent in VCD group, although they were also present in AD. With few treatment options available in AD, it may be important not to neglect the vascular risk factors.

Copyright © 2012 Elsevier B.V. All rights reserved.

Version ID

1

Record Owner

From MEDLINE, a database of the U.S. National Library of Medicine.

Status

MEDLINE

Authors Full Name

Stefanova, E; Pavlovic, A; Jovanovic, Z; Veselinovic, N; Despotovic, I; Stojkovic, T; Sternic, N; Kostic, V.

Institution

Stefanova, E. Faculty of Medicine, University of Belgrade, Serbia; Clinic of Neurology CCS, Serbia. steela21@gmail.com

MeSH Heading

Aged. Aged, 80 and over. Alzheimer Disease/di [Diagnosis]. *Alzheimer Disease/ep [Epidemiology]. Carotid Arteries/dg [Diagnostic Imaging]. Carotid Intima-Media Thickness. Cerebrovascular Disorders/di [Diagnosis]. *Cerebrovascular Disorders/ep [Epidemiology]. Female. Humans. Magnetic Resonance Imaging. Male. Mental Status Schedule. Middle Aged. Retrospective Studies. Risk Factors. Statistics, Nonparametric. Tomography, X-Ray Computed.

Year of Publication

2012

Link to the Ovid Full Text or citation:

Click here for full text options

Link to the External Link Resolver:

For peer review only - http://bmjopen.bmj.com/site/about/guidelines.xhtml

For peer review only

SFX

73.

Carotid atherosclerosis and 10-year changes in cognitive function.
Zhong W; Cruickshanks KJ; Schubert CR; Acher CW; Carlsson CM; Klein BE; Klein R; Chappell RJ.
Atherosclerosis. 224(2):506-10, 2012 Oct.
[Journal Article. Research Support, N.I.H., Extramural]
UI: 22854188

BACKGROUND: Carotid atherosclerosis has been suggested to be involved in cognitive decline.
METHODS: The Epidemiology of Hearing Loss Study is a longitudinal study of aging among Beaver Dam residents, WI. In 1998-2000, carotid intima-media thickness (IMT) and plaque were measured by ultrasound; cognitive function was measured by the Mini-Mental State Examination (MMSE). Follow-up examinations were conducted in 2003-2005 and 2009-2010. Incidence of cognitive impairment was defined as an MMSE score <24 or reported physician-diagnosed dementia during the follow-up. In the last examination, five additional cognitive tests were added. The associations of carotid atherosclerosis with incident cognitive impairment and cognitive test performance ten years later were evaluated.
RESULTS: A total of 1651 participants (mean age 66.8 years, 41% men) without cognitive impairment at baseline were included in the incidence analysis. IMT was associated with incidence of cognitive impairment after multiple adjustments (hazard ratio: 1.09, p = 0.02 for each 0.1 mm increase in IMT). A total of 1311 participants with atherosclerosis data at baseline had the additional cognitive tests 10 years later. Larger IMT was associated with longer time to complete the Trail-Making Test-part B after multiple adjustments (0.1 mm IMT: 2.3 s longer, p = 0.02). Plaque was not associated with incident cognitive impairment or cognitive test performance 10 years later.
CONCLUSIONS: In this population-based longitudinal study, carotid IMT was associated with a higher risk of developing cognitive impairment during the 10-year follow-up, and was associated with poorer performance in a test of executive function 10 years later.
Copyright Published by Elsevier Ireland Ltd.
Version ID
1
Record Owner
From MEDLINE, a database of the U.S. National Library of Medicine.
Status

For peer review only - http://bmjopen.bmj.com/site/about/guidelines.xhtml

MEDLINE
Authors Full Name
Zhong, Wenjun; Cruickshanks, Karen J; Schubert, Carla R; Acher, Charles W; Carlsson, Cynthia M; Klein, Barbara E K; Klein, Ronald; Chappell, Richard J.
Institution
Zhong, Wenjun. University of Wisconsin-Madison, 1036 WARF, 610 Walnut Street, WI 53726, USA. zhongwenjun80@gmail.com
MeSH Heading
Adult. Aged. Aged, 80 and over. Carotid Arteries/dg [Diagnostic Imaging]. Carotid Artery Diseases/dg [Diagnostic Imaging]. *Carotid Artery Diseases/ep [Epidemiology]. Carotid Artery Diseases/px [Psychology]. Carotid Intima-Media Thickness. *Cognition. Cognition Disorders/di [Diagnosis]. *Cognition Disorders/ep [Epidemiology]. Cognition Disorders/px [Psychology]. Female. Humans. Incidence. Linear Models. Logistic Models. Longitudinal Studies. Male. Middle Aged. Multivariate Analysis. Neuropsychological Tests. Odds Ratio. Plaque, Atherosclerotic. Predictive Value of Tests. Psychiatric Status Rating Scales. Risk Assessment. Risk Factors. Time Factors. Wisconsin/ep [Epidemiology].
Year of Publication
2012

Link to the Ovid Full Text or citation:
[Click here for full text options]

Link to the External Link Resolver:
[SFX]

74.

Impaired cognitive function in patients with atherosclerotic carotid stenosis and correlation with ultrasound strain measurements.
Rocque BG; Jackson D; Varghese T; Hermann B; McCormick M; Kliewer M; Mitchell C; Dempsey RJ.
Journal of the Neurological Sciences. 322(1-2):20-4, 2012 Nov 15.
[Journal Article. Research Support, N.I.H., Extramural]
UI: 22658531

For peer review only

INTRODUCTION: It has been postulated that up to 11 million "silent" strokes occur annually. While these patients are without classic neurologic deficits, they may exhibit cognitive decline. In this study, we examine the cognitive function of patients with carotid stenosis. Additionally, we evaluate a noninvasive measure of strain in pulsating carotid artery plaques to determine its ability to predict cognitive decline.

METHODS: We administered the Repeatable Battery for the Assessment of Neuropsychological Status (RBANS) to 44 patients with carotid stenosis. All patients had stenosis meeting NASCET or ACAS criteria for endarterectomy, and were classified as symptomatic or asymptomatic as defined by these publications. Age-adjusted scores for each of the 5 RBANS domains (immediate memory, visuospatial ability, language, attention, and delayed memory) were compared between symptomatic and asymptomatic patients. Mean score for each of the 5 domains was then compared to all other domains, regardless of symptom status. From this cohort, 23 patients underwent assessment of carotid plaque strain by tracking displacements in ultrasound radiofrequency data to estimate axial and principal strains over the cardiac cycle.

RESULTS: Thirty symptomatic and 14 asymptomatic patients were studied. Visuospatial scores were significantly lower than any other domain regardless of symptoms ($p<0.05$ for all pairwise comparisons). No other domain score was significantly different from any other. In the language domain, asymptomatic patients scored significantly higher than symptomatic patients ($p<0.05$. For all other domains, no difference was found. Asymptomatic patients showed a relationship between plaque strain and immediate memory ($r=-.61$, p=ns). Left carotid disease was associated with poorer performance across multiple cognitive domains with increasing accumulated strain. This was not seen in right carotid disease.

CONCLUSION: Patients with large carotid plaques (>70% stenosis) exhibit significant difficulties in mental status whether classically symptomatic or asymptomatic. While language deficits may be a non-specific marker for stroke symptoms, visuospatial deficits are seen before classic symptoms, suggesting that carotid disease may become symptomatic earlier and more subtly than previously suspected. Abnormal strain distribution with pulsation may be related to cognition.

Copyright © 2012 Elsevier B.V. All rights reserved.

Version ID

1

Record Owner

From MEDLINE, a database of the U.S. National Library of Medicine.

Status

MEDLINE

Authors Full Name

Rocque, Brandon G; Jackson, Daren; Varghese, Tomy; Hermann, Bruce; McCormick, Matthew; Kliewer, Mark; Mitchell, Carol; Dempsey, Robert J.

For peer review only - http://bmjopen.bmj.com/site/about/guidelines.xhtml

Institution

Rocque, Brandon G. UW-Madison School of Medicine and Public Health, Department of Neurological Surgery, 600 Highland Avenue, K4/866, Madison, WI 53792, United States.

MeSH Heading

Attention. Brain/pa [Pathology]. *Carotid Artery Diseases/co [Complications]. Carotid Artery Diseases/su [Surgery]. *Cognition Disorders/dg [Diagnostic Imaging]. *Cognition Disorders/et [Etiology]. Cohort Studies. Endarterectomy, Carotid/mt [Methods]. Female. Humans. Language. Male. Memory. Neuropsychological Tests. Space Perception. Statistics as Topic. Ultrasonography.

Year of Publication

2012

Link to the Ovid Full Text or citation:

[Click here for full text options]

Link to the External Link Resolver:

[SFX]

76.

Atherosclerotic calcification relates to cognitive function and to brain changes on magnetic resonance imaging.

Bos D; Vernooij MW; Elias-Smale SE; Verhaaren BF; Vrooman HA; Hofman A; Niessen WJ; Witteman JC; van der Lugt A; Ikram MA.

Alzheimer's & Dementia. 8(5 Suppl):S104-11, 2012 Oct.

[Journal Article. Research Support, Non-U.S. Gov't]

UI: 22537801

BACKGROUND: Increasing evidence suggests a role of atherosclerosis in the pathogenesis of cognitive impairment and dementia. Calcification volume measured with computed tomography (CT) is a valid marker of atherosclerosis. This study investigates associations of atherosclerosis (measured using CT) at four locations with cognition and brain changes on magnetic resonance imaging (MRI).

METHODS: To quantify calcification volume, 2414 nondemented people from the Rotterdam Study underwent CT of the coronary arteries, aortic arch, extracranial carotid arteries, and intracranial

For peer review only - http://bmjopen.bmj.com/site/about/guidelines.xhtml

carotid arteries. To assess global cognition and performance on memory, executive function, information processing speed, and motor speed, they also underwent neuropsychological tests. In a random subgroup of 844 participants, brain MRI was performed. Automated segmentation and quantification of brain MRI scans yielded brain tissue volumes in milliliters. Diffusion tensor imaging was used to measure the microstructural integrity of the white matter. Relationships of atherosclerotic calcification with cognition, brain tissue volumes, and diffusion tensor imaging measures were assessed with linear regression models and adjusted for relevant confounders.
RESULTS: With larger calcification volumes, lower cognitive scores were observed. When calcification volumes were larger, total brain volumes were also smaller. Specifically, larger coronary artery calcification volumes related to smaller gray matter volumes, and extracranial and intracranial carotid calcification volumes related to smaller white matter volumes. Larger calcification volume in all vessel beds was accompanied by worse microstructural integrity of the white matter.
CONCLUSIONS: Larger calcification volume is associated with worse cognitive performance. It also relates to smaller brain tissue volumes and worse white matter microstructural integrity, revealing possible mechanisms through which atherosclerosis may lead to poorer cognition.
Copyright © 2012 The Alzheimer's Association. Published by Elsevier Inc. All rights reserved.
Version ID
1
Record Owner
From MEDLINE, a database of the U.S. National Library of Medicine.
Status
MEDLINE
Authors Full Name
Bos, Daniel; Vernooij, Meike W; Elias-Smale, Suzette E; Verhaaren, Benjamin F J; Vrooman, Henri A; Hofman, Albert; Niessen, Wiro J; Witteman, Jacqueline C M; van der Lugt, Aad; Ikram, M Arfan.
Institution
Bos, Daniel. Department of Radiology, Erasmus MC, Rotterdam, The Netherlands.
MeSH Heading
Aged. Aorta, Thoracic/dg [Diagnostic Imaging]. Aorta, Thoracic/pa [Pathology]. *Atherosclerosis/co [Complications]. Atherosclerosis/dg [Diagnostic Imaging]. Atherosclerosis/pa [Pathology]. Brain/bs [Blood Supply]. Brain/dg [Diagnostic Imaging]. *Brain/pa [Pathology]. *Calcinosis/co [Complications]. Calcinosis/dg [Diagnostic Imaging]. Calcinosis/pa [Pathology]. Carotid Arteries/dg [Diagnostic Imaging]. Carotid Arteries/pa [Pathology]. Cognition Disorders/dg [Diagnostic Imaging]. *Cognition Disorders/et [Etiology]. *Cognition Disorders/pa [Pathology]. Coronary Vessels/dg [Diagnostic Imaging]. Coronary Vessels/pa [Pathology]. Female. Humans. Magnetic Resonance Imaging. Male. Neuropsychological Tests. Tomography, X-Ray Computed.

For peer review only - http://bmjopen.bmj.com/site/about/guidelines.xhtml

Year of Publication
2012

Link to the Ovid Full Text or citation:
Click here for full text options

Link to the External Link Resolver:
SFX

For peer review only

77.

Carotid artery plaque progression and cognitive decline: the Tromso Study 1994-2008.
Arntzen KA; Schirmer H; Johnsen SH; Wilsgaard T; Mathiesen EB.
European Journal of Neurology. 19(10):1318-24, 2012 Oct.
[Journal Article. Research Support, Non-U.S. Gov't]
UI: 22537454
BACKGROUND: Carotid atherosclerosis is a risk factor for stroke and cognitive decline, but knowledge on how progression of carotid atherosclerosis affects cognitive function in stroke-free individuals is scarce.
METHODS: In the population-based Tromso study, we calculated the change in ultrasound-assessed carotid plaque number and total plaque area from baseline (survey 4) to follow-up 7 years later (survey 5) in 4274 middle-aged stroke-free subjects. Cognitive function was assessed at follow-up by the verbal memory test, the digit-symbol coding test, and the tapping test and repeated after an additional 6 years in a subgroup of 2042 subjects (survey 6). Associations between the average of survey 4 and survey 5 plaque scores and the progression of plaque scores and cognitive test scores were assessed in regression analyses adjusted for baseline age, sex, education, depression, and cardiovascular risk factors.
RESULTS: Progression of total plaque area was associated with lower scores in the digit-symbol coding test (multivariable adjusted standardized beta, -0.03; 95% CI, -0.05 to -0.00; P = 0.04) and the tapping test (beta, -0.03; 95% CI, -0.06 to -0.00; P = 0.03). Similar results were seen for progression of plaque number. The average plaque scores were associated with lower scores in all cognitive tests (P-values <= 0.01). No association was found between plaque scores and cognitive decline.

For peer review only - http://bmjopen.bmj.com/site/about/guidelines.xhtml

CONCLUSIONS: The average plaque scores were associated with lower scores in all cognitive tests. Progression of plaque scores was associated with lower scores in the digit-symbol coding test and the tapping test, but not with the verbal memory test or with cognitive decline.

Copyright © 2012 The Author(s) European Journal of Neurology © 2012 EFNS.

Version ID

1

Record Owner

From MEDLINE, a database of the U.S. National Library of Medicine.

Status

MEDLINE

Authors Full Name

Arntzen, K A; Schirmer, H; Johnsen, S H; Wilsgaard, T; Mathiesen, E B.

Institution

Arntzen, K A. Department of Community Medicine, University of Tromso, Tromso, Norway. kjell.a.arntzen@uit.no

MeSH Heading

Carotid Stenosis/co [Complications]. *Carotid Stenosis/dg [Diagnostic Imaging]. *Cognition. *Cognition Disorders/ep [Epidemiology]. Cognition Disorders/et [Etiology]. Cross-Sectional Studies. Disease Progression. Female. Humans. Male. Middle Aged. Risk Factors. Ultrasonography.

Year of Publication

2012

Link to the Ovid Full Text or citation:

Click here for full text options

Link to the External Link Resolver:

SFX

79.

Carotid atherosclerosis predicts lower cognitive test results: a 7-year follow-up study of 4,371 stroke-free subjects - the Tromso study.

Arntzen KA; Schirmer H; Johnsen SH; Wilsgaard T; Mathiesen EB.

For peer review only

Cerebrovascular Diseases. 33(2):159-65, 2012.
[Journal Article. Research Support, Non-U.S. Gov't]
UI: 22222422
BACKGROUND: Carotid artery atherosclerosis is a major risk factor for stroke and subsequent cognitive impairment. Prospective population studies have shown associations between carotid intima-media thickness (IMT) and stenosis and cognitive decline and dementia in elderly stroke-free persons, whereas results in the middle-aged are conflicting.
METHODS: In this prospective population-based study, 4,371 stroke-free middle-aged participants underwent carotid ultrasound examination and assessment of vascular risk factors at baseline and were tested for cognitive function 7 years later. Associations between IMT, number of plaques and total plaque area and cognitive test scores on verbal memory test, digit symbol-coding test and tapping test were assessed in linear regression models.
RESULTS: In the multivariable analyses adjusted for sex, age, education, depression and vascular risk factors, the presence of plaques was significantly associated with lower test scores on the verbal memory test ($p = 0.01$) and on the digit symbol-coding test ($p = 0.03$). The number of plaques ($p = 0.01$) and the total plaque area ($p = 0.02$) were associated with lower scores on the verbal memory test. No significant association was seen between common carotid artery IMT and cognitive test scores. The tapping test was not associated with the carotid ultrasound variables.
CONCLUSIONS: In this middle-aged general population, subclinical carotid atherosclerosis measured as the presence of plaques, number of plaques and total plaque area were independent long-term predictors of lower cognitive test scores.
Copyright © 2012 S. Karger AG, Basel.
Version ID
1
Record Owner
From MEDLINE, a database of the U.S. National Library of Medicine.
Status
MEDLINE
Authors Full Name
Arntzen, Kjell Arne; Schirmer, Henrik; Johnsen, Stein Harald; Wilsgaard, Tom; Mathiesen, Ellisiv B.
Institution
Arntzen, Kjell Arne. Department of Community Medicine, University of Tromso, Tromso, Norway. kjell.a.arntzen@uit.no
MeSH Heading
Adult. Aged. Attention. Carotid Artery Diseases/dg [Diagnostic Imaging]. *Carotid Artery Diseases/ep [Epidemiology]. Carotid Intima-Media Thickness. *Cognition. *Cognition Disorders/ep [Epidemiology]. Cognition Disorders/px [Psychology]. Female. Follow-Up Studies. Humans. Linear

For peer review only

Models. Male. Memory. Middle Aged. Multivariate Analysis. Neuropsychological Tests. Norway/ep [Epidemiology]. Prospective Studies. Psychomotor Performance. Risk Assessment. Risk Factors. Severity of Illness Index. Time Factors. Wechsler Scales.

Year of Publication

2012

Link to the Ovid Full Text or citation:

Click here for full text options

Link to the External Link Resolver:

SFX

80.

Vascular predictors of cognitive decline in patients with mild cognitive impairment.

Viticchi G; Falsetti L; Vernieri F; Altamura C; Bartolini M; Luzzi S; Provinciali L; Silvestrini M.

Neurobiology of Aging. 33(6):1127.e1-9, 2012 Jun.

[Comparative Study. Journal Article. Research Support, Non-U.S. Gov't]

UI: 22217417

Our aim in this study was to assess the relationship between the state of cerebral vessels and the risk of conversion from mild cognitive impairment (MCI) to Alzheimer's disease (AD). We included 117 MCI patients. They underwent an ultrasonographic assessment of common carotid arteries intima-media thickness (IMT) and carotid plaque index. Cerebrovascular reactivity to hypercapnia in the middle cerebral arteries was calculated with the Breath-Holding Index (BHI). After a 12-month follow-up period, neuropsychological examinations demonstrated a progression to dementia in 21 patients. Pathological values of BHI and IMT significantly increased the risk of conversion (BHI: odds ratio, 5.80; 95% confidence interval, 1.83-18.37, $p < 0.05$; IMT: odds ratio, 3.08; 95% confidence interval, 1.02-9.33; $p < 0.05$, multinomial logistic regression analysis). Comparison between patients with all normal values and those with the simultaneous alteration of the 2 vascular indexes showed an increase in the risk of conversion from 9% to 33% (ordinal regression analysis). Our findings show that alterations of cerebral vessel functional and anatomic status increase the risk of conversion from MCI to dementia.

Copyright © 2012 IBRO. Published by Elsevier Inc. All rights reserved.

For peer review only - http://bmjopen.bmj.com/site/about/guidelines.xhtml

Version ID

1

Record Owner

From MEDLINE, a database of the U.S. National Library of Medicine.

Status

MEDLINE

Authors Full Name

Viticchi, Giovanna; Falsetti, Lorenzo; Vernieri, Fabrizio; Altamura, Claudia; Bartolini, Marco; Luzzi, Simona; Provinciali, Leandro; Silvestrini, Mauro.

Institution

Viticchi, Giovanna. Department of Experimental and Clinical Medicine, Marche Polytechnic University, Ancona, Italy.

MeSH Heading

Aged. Aged, 80 and over. *Alzheimer Disease/di [Diagnosis]. Alzheimer Disease/dg [Diagnostic Imaging]. Alzheimer Disease/ep [Epidemiology]. Carotid Artery, Common/dg [Diagnostic Imaging]. *Carotid Artery, Common/pa [Pathology]. Carotid Intima-Media Thickness/td [Trends]. Cognition Disorders/di [Diagnosis]. Cognition Disorders/dg [Diagnostic Imaging]. Cognition Disorders/ep [Epidemiology]. *Cognitive Dysfunction/di [Diagnosis]. Cognitive Dysfunction/dg [Diagnostic Imaging]. Cognitive Dysfunction/ep [Epidemiology]. *Disease Progression. Female. Follow-Up Studies. Humans. Male. Middle Cerebral Artery/dg [Diagnostic Imaging]. *Middle Cerebral Artery/pa [Pathology]. Predictive Value of Tests.

Year of Publication

2012

Link to the Ovid Full Text or citation:

Click here for full text options

Link to the External Link Resolver:

SFX

86.

For peer review only

Atherosclerosis and dementia: a cross-sectional study with pathological analysis of the carotid arteries.

Suemoto CK; Nitrini R; Grinberg LT; Ferretti RE; Farfel JM; Leite RE; Menezes PR; Fregni F; Jacob-Filho W; Pasqualucci CA; Brazilian Aging Brain Study Group.

Stroke. 42(12):3614-5, 2011 Dec.

[Journal Article. Research Support, Non-U.S. Gov't]

UI: 21940957

BACKGROUND AND PURPOSE: Previous ultrasound-based studies have shown an association between carotid artery atherosclerosis and dementia. Our aim was to investigate this association using postmortem examination.

METHODS: Postmortem morphometric measurements of carotid stenosis and intima-media thickness were performed in individuals with dementia (n=112) and control subjects (n=577). Multivariate logistic regression models were applied.

RESULTS: High-grade left internal carotid stenosis (>=70%) was associated with increased odds for dementia (OR, 2.30; 95% CI, 1.14-4.74; P=0.02). Intima-media thickness was not associated with dementia.

CONCLUSIONS: The likelihood of dementia is increased with high-grade left internal carotid artery atherosclerosis after adjusting for demographic and cardiovascular risk factors.

Version ID

1

Record Owner

From MEDLINE, a database of the U.S. National Library of Medicine.

Status

MEDLINE

Authors Full Name

Suemoto, Claudia K; Nitrini, Ricardo; Grinberg, Lea T; Ferretti, Renata E L; Farfel, Jose M; Leite, Renata E P; Menezes, Paulo R; Fregni, Felipe; Jacob-Filho, Wilson; Pasqualucci, Carlos A; Brazilian Aging Brain Study Group.

Institution

Suemoto, Claudia K. Department of Geriatrics, University of Sao Paulo Medical School, Avenida Doutor Arnaldo, 455, sala 1353, Sao Paulo, SP, Brazil. cksuemoto@usp.br

MeSH Heading

Aged. Aged, 80 and over. *Atherosclerosis/pa [Pathology]. *Carotid Arteries/pa [Pathology]. Carotid Intima-Media Thickness. *Carotid Stenosis/pa [Pathology]. Cross-Sectional Studies. *Dementia/pa [Pathology]. Female. Humans. Male. Middle Aged. Tunica Intima/pa [Pathology]. Tunica Media/pa [Pathology].

Year of Publication

For peer review only - http://bmjopen.bmj.com/site/about/guidelines.xhtml

2011

Link to the Ovid Full Text or citation:

Click here for full text options

Link to the External Link Resolver:

SFX

For peer review only

87.

Carotid atherosclerosis and cognitive function in midlife: the Beaver Dam Offspring Study.

Zhong W; Cruickshanks KJ; Huang GH; Klein BE; Klein R; Nieto FJ; Pankow JS; Schubert CR.

Atherosclerosis. 219(1):330-3, 2011 Nov.

[Journal Article. Research Support, N.I.H., Extramural]

UI: 21831374

BACKGROUND: Atherosclerosis may be associated with cognitive function; however the studies are few, especially among midlife adults.

METHODS: Participants in the beaver dam offspring study who had cognitive test data and gradable carotid artery ultrasound scans were included (n=2794, mean age: 49 years). Atherosclerosis was measured by carotid intima-media thickness (IMT) and the presence of plaque. Cognitive function was measured by the trail making test (TMT), grooved pegboard test (GPT) and mini-mental state examination (MMSE). Generalized cognitive function was defined by a summary score calculated from the TMT and GPT. Linear regression was used to evaluate the associations between carotid atherosclerosis and cognitive function tests.

RESULTS: Larger IMT was associated with lower GPT, MMSE and the summary score adjusting for multiple factors, the coefficients were: 13.8s (p<0.0001), -0.6 (p=0.007), and 0.47 (p=0.01), respectively for 1mm increase in IMT. Plaque scores were significantly associated with TMT-B, GPT, MMSE, and the summary score adjusting for age, sex and education. The associations remained statistically significant after further adjustments except for the association with TMT-B, which was attenuated and no longer significant.

CONCLUSIONS: Our results show the significant associations between markers of carotid atherosclerosis and cognitive function in a cohort of persons aged 21-84 years. Longitudinal studies are needed to further examine these associations.

Copyright A© 2011 Elsevier Ireland Ltd. All rights reserved.

Version ID

1

Record Owner

From MEDLINE, a database of the U.S. National Library of Medicine.

Status

MEDLINE

Authors Full Name

Zhong, Wenjun; Cruickshanks, Karen J; Huang, Guan-Hua; Klein, Barbara E K; Klein, Ronald; Nieto, F Javier; Pankow, James S; Schubert, Carla R.

Institution

Zhong, Wenjun. University of Wisconsin-Madison, 1036 WARF, 610 Walnut Street, Madison, WI 53726, USA. wzhong@wisc.edu

MeSH Heading

Adult. Aged. Aged, 80 and over. Carotid Artery Diseases/dg [Diagnostic Imaging]. *Carotid Artery Diseases/px [Psychology]. Carotid Intima-Media Thickness. *Cognition. Female. Humans. Male. Middle Aged. Plaque, Atherosclerotic/pa [Pathology].

Year of Publication

2011

Link to the Ovid Full Text or citation:

Click here for full text options

Link to the External Link Resolver:

SFX

96.

A review of carotid atherosclerosis and vascular cognitive decline: a new understanding of the keys to symptomology. [Review]

Dempsey RJ; Vemuganti R; Varghese T; Hermann BP.

Neurosurgery. 67(2):484-93; discussion 493-4, 2010 Aug.

[Journal Article. Research Support, N.I.H., Extramural. Review]

For peer review only

UI: 20644437

This review encourages the reader to consider cerebral vascular disease beyond the traditional clinical end points of major motor and speech strokes and to consider the possible impact of embolic cerebral vascular disease on vascular cognitive decline. This article examines the issue of "silent" strokes in the relationship between the structural stability of atherosclerotic carotid plaque and the development of nonmotor symptomatology, including cognitive decline. It addresses the question of the role of carotid emboli in silent stroke and their cognitive sequelae. In a study of endarterectomy patients, we relate plaque elasticity and its development of mechanical strain features and thinning of stabilizing fibrous cap at the point of these mechanical strain features. The possibility that microemboli from such mechanically unstable carotid plaques could contribute to silent strokes led to a study of cognitive function in such patients. A linear relationship between the process of mechanically unstable areas of carotid plaques and cognitive decline suggests a contributory role for such a process in silent strokes.

Version ID

1

Record Owner

From MEDLINE, a database of the U.S. National Library of Medicine.

Status

MEDLINE

Authors Full Name

Dempsey, Robert J; Vemuganti, Raghu; Varghese, Tomy; Hermann, Bruce P.

Institution

Dempsey, Robert J. University of Wisconsin School of Medicine and Public Health, Department of Neurological Surgery, Madison, Wisconsin 53792, USA. dempsey@neurosurg.wisc.edu

MeSH Heading

*Carotid Artery Diseases/co [Complications]. Carotid Artery Diseases/ge [Genetics]. *Carotid Artery Diseases/px [Psychology]. *Cerebrovascular Disorders/co [Complications]. *Cerebrovascular Disorders/px [Psychology]. *Cognition Disorders/et [Etiology]. *Cognition Disorders/px [Psychology]. Elasticity. Embolization, Therapeutic. Gene Expression. Humans. Intracranial Embolism/et [Etiology]. Intracranial Embolism/pa [Pathology]. Neovascularization, Pathologic/pa [Pathology]. Neuropsychological Tests. Plaque, Atherosclerotic/et [Etiology]. Plaque, Atherosclerotic/pa [Pathology]. Stroke/px [Psychology].

Year of Publication

2010

Link to the Ovid Full Text or citation:

For peer review only - http://bmjopen.bmj.com/site/about/guidelines.xhtml

Click here for full text options

Link to the External Link Resolver:

SFX

99.

Vascular characteristics of patients with dementia.

Morovic S; Jurasic MJ; Martinic Popovic I; Seric V; Lisak M; Demarin V.

Journal of the Neurological Sciences. 283(1-2):41-3, 2009 Aug 15.

[Journal Article. Research Support, Non-U.S. Gov't]

UI: 19375085

Arterial beta stiffness index is a potential risk factor for increased stroke occurrence. Vascular component appears to be significant in both Alzheimer's disease (AD) and vascular dementia (VAD). We aimed to further explore vascular characteristics of patients with both types of cognitive decline using non-invasive neurosonological methods. There were 38 patients; 16 diagnosed with AD and 22 with VAD. Vascular risk factors were assessed and ultrasound measurements on common carotid artery (CCA) were performed using Aloka ProSound ALPHA 10 with 13 MHz linear probe. Among AD patients there were 5 with arterial hypertension (AH), 3 with atrial fibrillation (AF), 2 with diabetes mellitus (DM), 6 with hyper lipidemia and 1 smoker. Nineteen VAD patients had AH, 6 had AF, 12 had hyper lipidemia and one was diabetic. We found no statistically significant differences between groups regarding average body mass index (BMI), blood pressure, pulse pressure, intima-media thickness (IMT), CCA diameter or arterial beta stiffness indices. However, the trend of BMI increase, slight blood and pulse pressure decrease, CCA diameter increase and beta stiffness index increase was noted in VAD patients. Even though there was no significant difference found among two explored subgroups of patients with dementia, there was a tendency of greater systolic and diastolic diameters noted in VAD as well as greater stiffness, especially when measured in the right CCA. This indicates that VAD patients may have more prominent vascular changes that may help differentiate the type of dementia and further monitor these individuals. Further studies on a larger number of patients are needed support this evidence.

Version ID

1

Record Owner

From MEDLINE, a database of the U.S. National Library of Medicine.

For peer review only

Status

MEDLINE

Authors Full Name

Morovic, Sandra; Jurasic, Miljenka-Jelena; Martinic Popovic, Irena; Seric, Vesna; Lisak, Marijana; Demarin, Vida.

Institution

Morovic, Sandra. University Department of Neurology, Sestre Milosrdnice University Hospital, Vinogradska 29, Zagreb, Croatia.

MeSH Heading

Aged. *Alzheimer Disease/dg [Diagnostic Imaging]. Alzheimer Disease/ep [Epidemiology]. Alzheimer Disease/pp [Physiopathology]. Atrial Fibrillation/ep [Epidemiology]. Blood Pressure. Body Mass Index. *Carotid Artery, Common/dg [Diagnostic Imaging]. Carotid Artery, Common/pa [Pathology]. Carotid Artery, Common/pp [Physiopathology]. *Dementia, Vascular/dg [Diagnostic Imaging]. Dementia, Vascular/ep [Epidemiology]. Dementia, Vascular/pp [Physiopathology]. Diabetes Mellitus/ep [Epidemiology]. Echoencephalography. Elasticity. Female. Humans. Hyperlipidemias/ep [Epidemiology]. Male. Organ Size. Risk Factors. Smoking/ep [Epidemiology].

Year of Publication

2009

Link to the Ovid Full Text or citation:

Click here for full text options

Link to the External Link Resolver:

SFX

100.

Carotid intima-media thickness as a predictor of response to cholinesterase inhibitors in Alzheimer's disease: an open-label trial.

Modrego PJ; Rios C; Perez Trullen JM; Garcia-Gomara MJ; Errea JM.

CNS Drugs. 23(3):253-60, 2009.

[Clinical Trial. Journal Article]

UI: 19320533

For peer review only - http://bmjopen.bmj.com/site/about/guidelines.xhtml

BACKGROUND: Cholinesterase inhibitors are modestly effective in treating patients with Alzheimer's disease. However, there may be important inter-individual variations ranging from no improvement at all to significant improvement and long periods of stabilization. Carotid atherosclerosis is associated with cognitive decline in elderly people.
OBJECTIVE: The objective of this study was to investigate whether carotid intima-media thickness (IMT) predicts response to cholinesterase inhibitors in Alzheimer's disease.
PATIENTS AND METHODS: A series of 54 patients with mild to moderate Alzheimer's disease were enrolled consecutively in an open-label trial. At baseline, all patients were assessed on the following clinical scales: Mini-Mental State Examination, Clinical Dementia Rating, the Hachinski Ischemic Scale, Blessed Dementia Rating Scale, Alzheimer's Disease Assessment Scale-cognitive subscale (ADAS-cog), Neuropsychiatric Inventory (NPI) and a daily-living activities scale (Disability Assessment for Dementia [DAD]). Investigations included magnetic resonance imaging of the brain and a colour echo-Doppler scan of the carotid arteries to measure the maximum IMT. Patients were then commenced on galantamine treatment for 6 months, after which scores on the ADAS-cog, NPI and DAD scales were reassessed.
RESULTS: A total of 50 patients completed the study. Their mean age was 77.78 years (SD 6.51 years); 34 patients were female. Galantamine treatment decreased the mean NPI score from 17.68 to 13.86 points, but this difference was not statistically significant (p=0.07). On the ADAS-cog scale, a modest and nonsignificant mean difference of -0.4 points (p=0.7) was observed. A weak (correlation coefficient r=0.4) but significant correlation between IMT and changes in clinical scale score was found, with low carotid IMT being shown to be a predictor of response on both the ADAS-cog (p=0.003) and NPI (p=0.006) scales; these findings were corroborated in multivariate analysis. For men, the correlation was stronger (r=0.7 and 0.8 for the ADAS-cog and NPI scales, respectively).
CONCLUSION: Although the magnitude of effect was moderate, carotid IMT could be a significant predictor of clinical response to cholinesterase inhibitors in patients with Alzheimer's disease.
Version ID
1
Record Owner
From MEDLINE, a database of the U.S. National Library of Medicine.
Status
MEDLINE
Authors Full Name
Modrego, Pedro J; Rios, Consuelo; Perez Trullen, Jose M; Garcia-Gomara, Maria J; Errea, Jose M.
Institution
Modrego, Pedro J. Department of Neurology, Hospital Universitario Miguel Servet, Zaragoza, Spain. pmodrego@salud.aragon.es

For peer review only - http://bmjopen.bmj.com/site/about/guidelines.xhtml

Comments

Comment in (CIN)

MeSH Heading

Activities of Daily Living. Aged. *Alzheimer Disease/dt [Drug Therapy]. Alzheimer Disease/pa [Pathology]. Alzheimer Disease/px [Psychology]. Atherosclerosis/co [Complications]. Atherosclerosis/dg [Diagnostic Imaging]. Atherosclerosis/pa [Pathology]. Carotid Artery Diseases/co [Complications]. *Carotid Artery Diseases/dg [Diagnostic Imaging]. *Carotid Artery Diseases/pa [Pathology]. *Cholinesterase Inhibitors/tu [Therapeutic Use]. Female. *Galantamine/tu [Therapeutic Use]. Humans. Magnetic Resonance Imaging. Male. Middle Aged. Neuropsychological Tests. Predictive Value of Tests. Psychiatric Status Rating Scales. Regression Analysis. Ultrasonography.

Registry Number/Name of Substance

0 (Cholinesterase Inhibitors). 0D3Q044KCA (Galantamine).

Year of Publication

2009

Link to the Ovid Full Text or citation:

Click here for full text options

Link to the External Link Resolver:

SFX

102.

Increased atherogenic lipoproteins are associated with cognitive impairment: effects of statins and subclinical atherosclerosis.

Carlsson CM; Nondahl DM; Klein BE; McBride PE; Sager MA; Schubert CR; Klein R; Cruickshanks KJ.

Alzheimer Disease & Associated Disorders. 23(1):11-7, 2009 Jan-Mar.

[Journal Article. Research Support, N.I.H., Extramural. Research Support, Non-U.S. Gov't]

UI: 19266697

Hypercholesterolemia increases the risk for dementia. Some studies suggest that statins may protect cognition, but findings are conflicting. Unmeasured confounders, including high-density lipoprotein (HDL) cholesterol or subclinical atherosclerosis, may have influenced prior study

For peer review only - http://bmjopen.bmj.com/site/about/guidelines.xhtml

outcomes. In older adults participating in a population-based cohort study (n=1711, aged 65 to 97 y), we investigated the relationships of total and HDL cholesterol levels, statin use, and carotid intima-media thickness with the prevalence of cognitive impairment. In adjusted models, participants in the highest quartile of non-HDL (total-HDL) cholesterol had an increased odds of cognitive impairment compared with those in the lowest quartile [odds ratio (OR): 2.06, 95% confidence interval (CI): 1.07-3.98]. Statin use was associated with lower odds of cognitive impairment in unadjusted models (OR: 0.57, 95% CI: 0.36-0.89), but this relationship was not significant after adjusting for vascular and lifestyle factors (OR: 0.84, 95% CI: 0.47-1.49). In this analysis of older adults, increased atherogenic lipoproteins were associated with impaired cognition. Statin use was related to many factors that both negatively and positively affect cognition, but was not associated with better cognitive function. These results suggest that confounding by indication may explain the contradictory findings in studies assessing the association of statins with cognition. Randomized-controlled clinical trials and longitudinal studies are necessary to determine if statins protect against cognitive decline.

Version ID

1

Record Owner

From MEDLINE, a database of the U.S. National Library of Medicine.

Status

MEDLINE

Authors Full Name

Carlsson, Cynthia M; Nondahl, David M; Klein, Barbara E K; McBride, Patrick E; Sager, Mark A; Schubert, Carla R; Klein, Ronald; Cruickshanks, Karen J.

Institution

Carlsson, Cynthia M. Section of Geriatrics and Gerontology, Department of Medicine, University of Wisconsin School of Medicine and Public Health, Madison, WI53705, USA. cmc@medicine.wisc.edu

Comments

Comment in (CIN)

MeSH Heading

Aged. Aged, 80 and over. *Atherosclerosis/co [Complications]. *Carotid Arteries/pa [Pathology]. *Cholesterol/bl [Blood]. *Cholesterol, HDL/bl [Blood]. Cognition Disorders/bl [Blood]. *Cognition Disorders/et [Etiology]. Female. Humans. *Hydroxymethylglutaryl-CoA Reductase Inhibitors/tu [Therapeutic Use]. Hypercholesterolemia/dt [Drug Therapy]. Male. Tunica Intima/pa [Pathology]. Tunica Media/pa [Pathology].

Registry Number/Name of Substance

0 (Cholesterol, HDL). 0 (Hydroxymethylglutaryl-CoA Reductase Inhibitors). 97C5T2UQ7J (Cholesterol).

For peer review only - http://bmjopen.bmj.com/site/about/guidelines.xhtml

Year of Publication

2009

Link to the Ovid Full Text or citation:

Click here for full text options

Link to the External Link Resolver:

SFX

103.

Vascular structure and function is correlated to cognitive performance and white matter hyperintensities in older hypertensive patients with subjective memory complaints.

Kearney-Schwartz A; Rossignol P; Bracard S; Felblinger J; Fay R; Boivin JM; Lecompte T; Lacolley P; Benetos A; Zannad F.

Stroke. 40(4):1229-36, 2009 Apr.

[Journal Article. Research Support, Non-U.S. Gov't]

UI: 19246701

BACKGROUND AND PURPOSE: Arterial stiffening and thickening and endothelial dysfunction may be associated with cognitive decline or white matter hyperintensities (WMH) independently of blood pressure level. We aimed to investigate, using an integrative approach, the relative contributions of structural and functional vascular factors to the degree of cognitive impairment (primary outcome) and the severity of WMH (secondary outcome) in elderly hypertensive patients with subjective memory complaints, a group prone to dementia.

METHODS: A prospective, dedicated, cross-sectional population of 198 elderly hypertensive patients (mean age 69.3+/-6.2 years) with subjective memory complaints underwent a full set of cognitive function assessments, brain MRI with semiquantification of WMH, carotid ultrasonography, carotid-femoral pulse wave velocity, brachial endothelial function, and plasma von Willebrand Factor measurements.

RESULTS: After adjustment for the usual cardiovascular risk factors, increased arterial stiffness (as assessed by pulse wave velocity) was significantly and independently associated with memory impairment in men. The severity of WMH was independently associated with increased carotid

For peer review only - http://bmjopen.bmj.com/site/about/guidelines.xhtml

intima media thickness and stiffness (as assessed by augmentation index) as well as with increased age and plasma levels of von Willebrand Factor, a biomarker of endothelial dysfunction. CONCLUSIONS: Our data suggest that vascular abnormalities, independently of blood pressure levels, may play a role in the setting of subjective memory complaints as well as of WMH in elderly hypertensive patients. Arterial thickness and stiffness as well as endothelial function should be assessed simultaneously and may represent additional targets for the prevention of subjective memory complaints and WMH.

Version ID

1

Record Owner

From MEDLINE, a database of the U.S. National Library of Medicine.

Status

MEDLINE

Authors Full Name

Kearney-Schwartz, Anna; Rossignol, Patrick; Bracard, Serge; Felblinger, Jacques; Fay, Renaud; Boivin, Jean-Marc; Lecompte, Thomas; Lacolley, Patrick; Benetos, Athanase; Zannad, Faiez.

Institution

Kearney-Schwartz, Anna. Nancy University Hospital, Clinical Investigation Centre, J d'Arc Hospital, Dommartin les Toul, France.

Comments

Comment in (CIN)

MeSH Heading

Aged. Aged, 80 and over. Carotid Artery Diseases/pa [Pathology]. Cerebrovascular Circulation. Cognition Disorders/ep [Epidemiology]. Cognition Disorders/pa [Pathology]. Cognition Disorders/pp [Physiopathology]. Cross-Sectional Studies. Endothelium, Vascular/pa [Pathology]. Endothelium, Vascular/ph [Physiology]. Female. Humans. *Hypertension/ep [Epidemiology]. *Hypertension/pa [Pathology]. Hypertension/pp [Physiopathology]. *Leukoaraiosis/ep [Epidemiology]. *Leukoaraiosis/pa [Pathology]. Leukoaraiosis/pp [Physiopathology]. Magnetic Resonance Imaging. Male. *Memory Disorders/ep [Epidemiology]. *Memory Disorders/pa [Pathology]. Memory Disorders/pp [Physiopathology]. Middle Aged. Nerve Fibers, Myelinated/pa [Pathology]. Prevalence. Prospective Studies. Pulsatile Flow. Risk Factors. Severity of Illness Index.

Year of Publication

2009

Link to the Ovid Full Text or citation:

Click here for full text options

For peer review only

For peer review only - http://bmjopen.bmj.com/site/about/guidelines.xhtml

Link to the External Link Resolver:

SFX

104.

Subclinical atherosclerosis is weakly associated with lower cognitive function in healthy hyperhomocysteinemic adults without clinical cardiovascular disease.

Gatto NM; Henderson VW; St John JA; McCleary C; Detrano R; Hodis HN; Mack WJ.

International Journal of Geriatric Psychiatry. 24(4):390-9, 2009 Apr.

[Journal Article. Randomized Controlled Trial. Research Support, N.I.H., Extramural]

UI: 18836986

OBJECTIVE: Atherosclerosis is the most common pathologic process underlying cardiovascular disease (CVD). It is not well known whether subclinical atherosclerosis is an independent risk factor for lower cognitive function among individuals without clinically evident CVD.

METHODS: We examined cross-sectional associations between subclinical atherosclerosis and cognitive function in a community-based sample of otherwise healthy adults with plasma homocysteine >or=8.5 micromol/L enrolled in the BVAIT study (n = 504, mean age 61 years). Carotid artery intima-media thickness (CIMT), coronary artery calcium (CAC) and abdominal aortic calcium (AAC) were used to measure subclinical atherosclerosis. Cognitive function was assessed with a battery of neuropsychological tests. A principal components analysis was used to extract five uncorrelated cognitive factors from scores on individual tests, and a measure of global cognition was derived. Multivariable linear regression was used to examine the association between subclinical atherosclerosis and cognitive function, adjusting for other correlates of cognition.

RESULTS: Increasing thickness of CIMT was associated with significantly lower scores on the verbal learning factor (beta = -0.07 per 0.1 mm increase CIMT [SE(beta) = 0.03], p = 0.01). CAC and AAC were not individually associated with any of the cognitive factors.

CONCLUSIONS: This study provides evidence that increasing CIMT is weakly associated with lower verbal learning abilities but not global cognition in a population of otherwise healthy middle-to-older aged adults with elevated plasma homocysteine levels but without clinically evident CVD. The association between CIMT and poor verbal learning may pertain particularly to men.

Copyright (c) 2008 John Wiley & Sons, Ltd.

Version ID

1

For peer review only - http://bmjopen.bmj.com/site/about/guidelines.xhtml

Record Owner

From MEDLINE, a database of the U.S. National Library of Medicine.

Status

MEDLINE

Authors Full Name

Gatto, Nicole M; Henderson, Victor W; St John, Jan A; McCleary, Carol; Detrano, Robert; Hodis, Howard N; Mack, Wendy J.

Institution

Gatto, Nicole M. Department of Preventive Medicine, USC Keck School of Medicine, Los Angeles, CA 90089-9010, USA.

MeSH Heading

Adult. Aged. Aged, 80 and over. *Atherosclerosis/co [Complications]. Atherosclerosis/dg [Diagnostic Imaging]. Atherosclerosis/pa [Pathology]. Carotid Arteries/dg [Diagnostic Imaging]. Carotid Arteries/pa [Pathology]. Carotid Artery Diseases/co [Complications]. Carotid Artery Diseases/pa [Pathology]. Cognition Disorders/dg [Diagnostic Imaging]. *Cognition Disorders/et [Etiology]. Cross-Sectional Studies. Female. Humans. *Hyperhomocysteinemia/co [Complications]. Hyperhomocysteinemia/dg [Diagnostic Imaging]. Hyperhomocysteinemia/pa [Pathology]. Male. Middle Aged. Neuropsychological Tests. Risk Factors. Tomography, X-Ray Computed.

Year of Publication

2009

Link to the Ovid Full Text or citation:

Click here for full text options

Link to the External Link Resolver:

SFX

105.

Carotid atherosclerosis and cognitive decline in patients with Alzheimer's disease.

Silvestrini M; Gobbi B; Pasqualetti P; Bartolini M; Baruffaldi R; Lanciotti C; Cerqua R; Altamura C; Provinciali L; Vernieri F.

Neurobiology of Aging. 30(8):1177-83, 2009 Aug.

For peer review only

For peer review only - http://bmjopen.bmj.com/site/about/guidelines.xhtml

[Journal Article. Research Support, Non-U.S. Gov't]

UI: 18077061

Aim of the study was to explore the correlation between the progression of carotid atherosclerosis and the evolution of cognitive impairment in 66 patients with Alzheimer's disease (AD). They underwent cognitive status evaluation and ultrasonography (US) to investigate carotid arteries intima-media thickness (IMT) and plaque index (PI). After a 12-month follow-up period, neuropsychological and US examinations were repeated to assess the progression of carotid atherosclerosis and of cognitive decline [in terms of changes in Mini Mental State Examination (MMSE) scores]. MMSE score changes were related to baseline IMT ($p=0.018$), changes in IMT ($p<0.001$) and PI ($p=0.006$), and "antihypertensive drug intake" ($p<0.001$). While the first three variables correlated with increased cognitive impairment, the last one was associated with a reduced extent of MMSE score decline. Results show a link between progression of carotid wall changes and of cognitive decline, and suggest a possible protective role of antihypertensive therapy. Given the potential clinical implications, our preliminary findings could stimulate further investigations into the role of vascular impairment in patients with AD.

Version ID

1

Record Owner

From MEDLINE, a database of the U.S. National Library of Medicine.

Status

MEDLINE

Authors Full Name

Silvestrini, Mauro; Gobbi, Beatrice; Pasqualetti, Patrizio; Bartolini, Marco; Baruffaldi, Roberto; Lanciotti, Chiara; Cerqua, Raffaella; Altamura, Claudia; Provinciali, Leandro; Vernieri, Fabrizio.

Institution

Silvestrini, Mauro. Neurological Clinic, Polytechnic University of Marche, Ancona, Italy.

m.silvestrini@univpm.it

MeSH Heading

Aged. *Alzheimer Disease/co [Complications]. Alzheimer Disease/dt [Drug Therapy]. Antihypertensive Agents/tu [Therapeutic Use]. Carotid Arteries/dg [Diagnostic Imaging]. Carotid Arteries/de [Drug Effects]. *Carotid Artery Diseases/co [Complications]. Carotid Artery Diseases/dg [Diagnostic Imaging]. Carotid Artery Diseases/dt [Drug Therapy]. Cognition/de [Drug Effects]. *Cognition Disorders/co [Complications]. Cognition Disorders/dt [Drug Therapy]. Disease Progression. Female. Follow-Up Studies. Humans. Linear Models. Male. Neuroprotective Agents/tu [Therapeutic Use]. Severity of Illness Index. Ultrasonography.

Registry Number/Name of Substance

0 (Antihypertensive Agents). 0 (Neuroprotective Agents).

For peer review only - http://bmjopen.bmj.com/site/about/guidelines.xhtml

Year of Publication

2009

Link to the Ovid Full Text or citation:

Click here for full text options

Link to the External Link Resolver:

SFX

107.

Socioeconomic status moderates the association between carotid intima-media thickness and cognition in midlife: evidence from the Whitehall II study.

Singh-Manoux A; Britton A; Kivimaki M; Gueguen A; Halcox J; Marmot M.

Atherosclerosis. 197(2):541-8, 2008 Apr.

[Journal Article. Research Support, N.I.H., Extramural. Research Support, Non-U.S. Gov't]

UI: 17854813

BACKGROUND: Common carotid artery intima-media thickness (IMT) is a measure of generalized atherosclerosis and has been shown to be associated with cognitive function. We examine two questions: does socioeconomic status (SES) moderate this association and is IMT more strongly associated with specific aspects of cognitive function?

METHODS: Data are drawn from the Phase 7 (2003-2004) of the Whitehall II study (N=3896). In cross-sectional analyses the association between IMT and six measures of cognition (short-term verbal memory, inductive reasoning, vocabulary, semantic and phonemic fluency and a measure of global cognitive status) was examined in analyses adjusted for previous history of coronary heart disease, health behaviours and other vascular risk measures such as blood pressure, cholesterol and body mass index.

RESULTS: The overall association between IMT and the six measures of cognition was restricted to the low SES group (p=0.02). Within this group, IMT was significantly associated with inductive reasoning (p=0.001), vocabulary (p=0.002), phonemic (p=0.006) and semantic fluency (p=0.02). The covariates examined explained about a quarter of the association between IMT and cognition in the low SES group. The associations with the measure of inductive reasoning (p=0.02), vocabulary (p=0.02) and phonemic fluency (p=0.04) remained after adjustment for all covariates.

For peer review only

CONCLUSIONS: SES is an important modifier of the association between IMT and cognition, an inverse association between the two was observed only in the low SES group. It is possible that high cognitive reserve among the high SES individuals prevents the functional manifestations of atherosclerosis. Verbal memory was not one of the cognitive domains associated with IMT.

Version ID

1

Record Owner

From MEDLINE, a database of the U.S. National Library of Medicine.

Status

MEDLINE

Authors Full Name

Singh-Manoux, Archana; Britton, Annie; Kivimaki, Mika; Gueguen, Alice; Halcox, Julian; Marmot, Michael.

Institution

Singh-Manoux, Archana. INSERM, U687-IFR69, HNSM, 14 rue du Val d'Osne, 94415 Saint-Maurice Cedex, France. Archana.Singh-Manoux@st-maurice.inserm.fr

MeSH Heading

Aged. *Atherosclerosis/co [Complications]. Atherosclerosis/ec [Economics]. Atherosclerosis/pa [Pathology]. *Carotid Artery Diseases/co [Complications]. Carotid Artery Diseases/ec [Economics]. Carotid Artery Diseases/pa [Pathology]. *Carotid Artery, Common/pa [Pathology]. *Cognition. *Cognition Disorders/pp [Physiopathology]. Cross-Sectional Studies. Female. Health Surveys. Humans. Male. Middle Aged. *Social Class. Tunica Intima/pa [Pathology]. Tunica Media/pa [Pathology].

Year of Publication

2008

Link to the Ovid Full Text or citation:

Click here for full text options

Link to the External Link Resolver:

SFX

108.

For peer review only

Carotid intima-media thickness and cognitive function in elderly women: a population-based study.

Komulainen P; Kivipelto M; Lakka TA; Hassinen M; Helkala EL; Patja K; Nissinen A; Rauramaa R.

Neuroepidemiology. 28(4):207-13, 2007.

[Comparative Study. Journal Article. Research Support, Non-U.S. Gov't]

UI: 17851259

OBJECTIVE: Several vascular risk factors have been linked to cognitive decline. However, little is known about the association between the atherosclerotic process and cognitive impairment. We investigated whether carotid intima-media thickness (IMT) predicts the risk of cognitive impairment and whether the putative impairment is specific for some cognitive domains.

METHODS: A 12-year population-based follow-up study was performed for a total of 91 women, aged 60-70 years at baseline. Ultrasonographically assessed carotid artery IMT and the Mini-Mental State Examination test were performed at baseline and 12-year follow-up. A detailed cognitive evaluation for memory and cognitive speed was performed in 2003. The mean of left and right carotid bifurcation IMT was used in the analyses for association with the risk for poor cognitive speed and memory.

RESULTS: Increased IMT at baseline was an independent predictor for poor memory (beta = -5.004, 95% confidence interval = -7.74 to -2.27; p = 0.001) and cognitive speed (beta = 2.562, 95% confidence interval = 1.19-4.94; p = 0.035) at 12-year follow-up after adjustment for age, education, depression, plasma LDL cholesterol, systolic blood pressure, cardiovascular disease, hormone replacement therapy, smoking, alcohol consumption and physical activity. The risk for poor memory (p = 0.023 for linear trend) and cognitive speed (p = 0.070 for linear trend) increased with increasing IMT tertiles.

CONCLUSIONS: Carotid IMT predicts an increased risk for cognitive impairment, particularly poor memory and cognitive speed, in elderly women.

Copyright (c) 2007 S. Karger AG, Basel.

Version ID

1

Record Owner

From MEDLINE, a database of the U.S. National Library of Medicine.

Status

MEDLINE

Authors Full Name

Komulainen, Pirjo; Kivipelto, Miia; Lakka, Timo A; Hassinen, Maija; Helkala, Eeva-Liisa; Patja, Kristiina; Nissinen, Aulikki; Rauramaa, Rainer.

Institution

For peer review only

Komulainen, Pirjo. Kuopio Research Institute of Exercise Medicine, Kuopio, Finland.
pirjo.komulainen@uku.fi

MeSH Heading

Aged. Aged, 80 and over. Atherosclerosis/co [Complications]. Atherosclerosis/pa [Pathology]. *Carotid Artery, Common/pa [Pathology]. Cognition/ph [Physiology]. *Cognition Disorders/et [Etiology]. *Cognition Disorders/pa [Pathology]. Female. Follow-Up Studies. Humans. Middle Aged. Risk Factors. *Tunica Intima/pa [Pathology]. *Tunica Media/pa [Pathology].

Year of Publication

2007

Link to the Ovid Full Text or citation:

[Click here for full text options]

Link to the External Link Resolver:

[SFX]

109.

Cerebrovascular risk factors, vascular disease, and neuropsychological outcomes in adults with major depression.

Smith PJ; Blumenthal JA; Babyak MA; Hoffman BM; Doraiswamy PM; Waugh R; Hinderliter A; Sherwood A.

Psychosomatic Medicine. 69(6):578-86, 2007 Jul-Aug.

[Journal Article. Research Support, N.I.H., Extramural]

UI: 17634564

OBJECTIVE: To investigate the relationship of cerebrovascular risk factors (CVRFs), endothelial function, carotid artery intima medial thickness (IMT), and neuropsychological performance in a sample of 198 middle-aged and older individuals with major depressive disorder (MDD). Neuropsychological deficits are common among adults with MDD, particularly among those with CVRFs and potentially persons with subclinical vascular disease.

METHODS: CVRFs were indexed by the Framingham Stroke Risk Profile (FSRP) and serum cholesterol levels obtained by medical history and physical examination. Patients completed a neuropsychological test battery including measures of executive functioning, working memory,

For peer review only - http://bmjopen.bmj.com/site/about/guidelines.xhtml

and verbal recall. Vascular function was indexed by carotid artery IMT and brachial artery flow mediated dilation (FMD). Hierarchical multiple regression analyses were used to investigate the association between CVRFs, vascular disease, and neurocognitive performance.

RESULTS: Greater FSRP scores were associated with poorer executive functioning (b = -0.86; p = .041) and working memory (b = -0.90; p = .024). Lower high-density lipoprotein levels also were associated with poorer executive functioning (b = 1.03; p = .035). Higher IMT (b = -0.83; p = .028) and lower FMD (b = 1.29; p = .032) were associated with poorer executive functioning after controlling for CVRFs. Lower FMD was also associated with poorer working memory (b = 1.58; p = .015).

CONCLUSIONS: Greater CVRFs were associated with poorer neuropsychological performance. Vascular dysfunction also was associated with neuropsychological decrements independent of traditional CVRFs.

Version ID

1

Record Owner

From MEDLINE, a database of the U.S. National Library of Medicine.

Status

MEDLINE

Authors Full Name

Smith, Patrick J; Blumenthal, James A; Babyak, Michael A; Hoffman, Benson M; Doraiswamy, P Murali; Waugh, Robert; Hinderliter, Alan; Sherwood, Andrew.

Institution

Smith, Patrick J. Department of Psychiatry and Behavioral Sciences, Duke University Medical Center, Durham, NC 27710, USA. Smith562@mc.duke.edu

MeSH Heading

Atherosclerosis/ep [Epidemiology]. *Atherosclerosis/pp [Physiopathology]. Cerebrovascular Disorders/ep [Epidemiology]. *Cerebrovascular Disorders/pp [Physiopathology]. *Cognition. Cross-Sectional Studies. Depressive Disorder, Major/ep [Epidemiology]. *Depressive Disorder, Major/pp [Physiopathology]. *Endothelium, Vascular/pp [Physiopathology]. Factor Analysis, Statistical. Female. Humans. Male. Middle Aged. Multivariate Analysis. Regression Analysis. Risk Factors. United States/ep [Epidemiology].

Year of Publication

2007

Link to the Ovid Full Text or citation:

Click here for full text options

For peer review only - http://bmjopen.bmj.com/site/about/guidelines.xhtml

Link to the External Link Resolver:
SFX

111.

Atherosclerosis and risk for dementia.
van Oijen M; de Jong FJ; Witteman JC; Hofman A; Koudstaal PJ; Breteler MM.
Annals of Neurology. 61(5):403-10, 2007 May.
[Journal Article. Research Support, Non-U.S. Gov't]
UI: 17328068
OBJECTIVE: Atherosclerosis has been implicated in the development of dementia and its major subtypes, Alzheimer's disease and vascular dementia. However, support for this association mainly comes from cross-sectional studies. We investigated the association of atherosclerosis with dementia and subtypes of dementia during long follow-up, with various noninvasive measures of atherosclerosis.
METHODS: This study was based on 6,647 participants in the Rotterdam Study, a population-based prospective cohort study among 7,983 elderly subjects. At baseline (1990-1993) and at the third survey (1997-1999), common carotid intima media thickness, carotid plaques, and peripheral arterial disease (measured as ankle-brachial index) were measured. During follow-up (mean, 9.0 years), 678 subjects developed dementia. We estimated the associations of different measures of atherosclerosis with risk for dementia and subtypes of dementia by means of Cox proportional hazard models. Analyses were repeated and stratified on duration of follow-up. To evaluate competing risk for mortality, we examined the association between measures of atherosclerosis and risk for dementia or mortality by combining the two in a single outcome measure.
RESULTS: We found that atherosclerosis, predominantly carotid atherosclerosis, was associated with an increased risk for dementia during short follow-up. This association attenuated with longer follow-up, likely because of the strong association between atherosclerosis and mortality. The associations did not differ across apolipoprotein E genotypes.
INTERPRETATION: Our findings suggest that atherosclerosis is associated with an increased risk for dementia. Stronger associations between atherosclerosis and mortality may attenuate the association between atherosclerosis and dementia in prospective cohort studies with long follow-up periods.
Version ID

For peer review only - http://bmjopen.bmj.com/site/about/guidelines.xhtml

1

Record Owner

From MEDLINE, a database of the U.S. National Library of Medicine.

Status

MEDLINE

Authors Full Name

van Oijen, Marieke; de Jong, Frank Jan; Witteman, Jacqueline C M; Hofman, Albert; Koudstaal, Peter J; Breteler, Monique M B.

Institution

van Oijen, Marieke. Department of Epidemiology and Biostatistics, Erasmus Medical Center, Rotterdam, the Netherlands.

Comments

Comment in (CIN) Comment in (CIN)

MeSH Heading

Aged. Apolipoproteins E/ge [Genetics]. *Atherosclerosis/co [Complications]. Atherosclerosis/dg [Diagnostic Imaging]. *Atherosclerosis/ep [Epidemiology]. Carotid Arteries/dg [Diagnostic Imaging]. Cohort Studies. Data Interpretation, Statistical. Dementia/di [Diagnosis]. *Dementia/ep [Epidemiology]. Dementia/px [Psychology]. Female. Follow-Up Studies. Genotype. Humans. Male. Netherlands/ep [Epidemiology]. Neuropsychological Tests. Population. Prospective Studies. Risk. Risk Factors. Ultrasonography.

Registry Number/Name of Substance

0 (Apolipoproteins E).

Year of Publication

2007

Link to the Ovid Full Text or citation:

Click here for full text options

Link to the External Link Resolver:

SFX

112.

For peer review only

Carotid artery intima-media thickness and cognition in cardiovascular disease.

Haley AP; Forman DE; Poppas A; Hoth KF; Gunstad J; Jefferson AL; Paul RH; Ler AS; Sweet LH; Cohen RA.

International Journal of Cardiology. 121(2):148-54, 2007 Oct 01.

[Comparative Study. Journal Article. Research Support, N.I.H., Extramural]

UI: 17196687

BACKGROUND: Increased carotid artery intima-media thickness (IMT) is a non-invasive marker of systemic arterial disease. Increased IMT has been associated with atherosclerosis, abnormal arterial mechanics, myocardial infarction, and stroke. Given evidence of a relationship between cardiovascular health and attention-executive-psychomotor functioning, the purpose of this study was to examine IMT in relation to neuropsychological test performance in patients with a variety of cardiovascular diagnoses.

METHODS: One hundred and nine participants, ages 55 to 85, underwent neuropsychological assessment and B-mode ultrasound of the left common carotid artery. IMT was calculated using an automated algorithm based on a validated edge-detection technique. The relationship between IMT and measures of language, memory, visual-spatial abilities and attention-executive-psychomotor functioning was modeled using hierarchical linear regression analyses adjusted for age, education, sex, cardiovascular risk, current systolic blood pressure, and history of coronary artery disease (CAD).

RESULTS: Increased IMT was associated with significantly lower performance in the attention-executive-psychomotor domain (IMT beta=-0.26, p<.01), independent of age, education, sex, cardiovascular risk, current systolic blood pressure, and CAD (F(10,100)=3.61, p<.001). IMT was not significantly related to language, memory, or visual-spatial abilities.

CONCLUSIONS: Our findings suggest that, in patients with cardiovascular disease, IMT may be associated with the integrity of frontal subcortical networks responsible for attention-executive-psychomotor performance. Future studies are needed to clarify the mechanisms by which IMT affects cognition and examine potential interactions between increased IMT and other measures of cardiovascular health such as blood pressure variability, cardiac systolic performance, and systemic perfusion.

Version ID

1

Record Owner

From MEDLINE, a database of the U.S. National Library of Medicine.

Status

MEDLINE

Authors Full Name

For peer review only - http://bmjopen.bmj.com/site/about/guidelines.xhtml

Haley, Andreana P; Forman, Daniel E; Poppas, Athena; Hoth, Karin F; Gunstad, John; Jefferson, Angela L; Paul, Robert H; Ler, Albert S H; Sweet, Lawrence H; Cohen, Ronald A.

Institution

Haley, Andreana P. Department of Psychiatry and Human Behavior, Brown Medical School, Providence, RI, United States. aphaley@brown.edu <aphaley@brown.edu>

MeSH Heading

Aged. Aged, 80 and over. Cardiovascular Diseases/co [Complications]. *Cardiovascular Diseases/pa [Pathology]. Cardiovascular Diseases/px [Psychology]. Carotid Artery Diseases/co [Complications]. *Carotid Artery Diseases/pa [Pathology]. Carotid Artery Diseases/px [Psychology]. *Carotid Artery, Common/pa [Pathology]. *Cognition. Cognition Disorders/et [Etiology]. Cognition Disorders/pa [Pathology]. Cognition Disorders/px [Psychology]. Female. Humans. Male. Middle Aged. Neuropsychological Tests. *Tunica Intima/pa [Pathology].

Year of Publication

2007

Link to the Ovid Full Text or citation:

Click here for full text options

Link to the External Link Resolver:

SFX

114.

Cognitive impairment and carotid atherosclerosis in a general Italian midlife and old population.

Prati P; Casaroli M; Bignamini A; Scotti S; Canciani L; Ruscio M; Balestrieri M; Bornstein N; Zanetti O; Tosetto A; Castellani S; Pantoni L; Touboul PJ; Inzitari D.

Neuroepidemiology. 27(1):33-8, 2006.

[Journal Article. Research Support, Non-U.S. Gov't]

UI: 16804332

The authors describe the design and the general, ultrasonographic, neuropsychological methodology of an observational epidemiological population survey, named REMEMBER (Registry Evaluation Memory in Buttrio e Remanzacco) conducted in the northeast of Italy in a randomized stratified sample of 1,026 subjects (554 F and 472 M) aged 55-98 years. The study was planned as

For peer review only

cross-sectional and longitudinal survey of cognitive impairment, cardiovascular risk factors, carotid atherosclerosis in a midlife and older Italian population sample. The objectives of the first phase are to assess the prevalence of the different types of dementia, the cognitive impairment non-dementia, the cardiovascular risk factors, the carotid intima-media thickness and arterial distensibility, and of depression. The conclusions of this study will make it possible to organize preventive and interventional strategies for these epidemic conditions.

Version ID

1

Record Owner

From MEDLINE, a database of the U.S. National Library of Medicine.

Status

MEDLINE

Authors Full Name

Prati, P; Casaroli, M; Bignamini, A; Scotti, S; Canciani, L; Ruscio, M; Balestrieri, M; Bornstein, N; Zanetti, O; Tosetto, A; Castellani, S; Pantoni, L; Touboul, P J; Inzitari, D.

Institution

Prati, P. Department of Neurology, Gervasutta Hospital, Udine, Italy. patrizio.prati@tin.it

MeSH Heading

Aged. Aged, 80 and over. *Alzheimer Disease/ep [Epidemiology]. Carotid Artery Diseases/dg [Diagnostic Imaging]. *Carotid Artery Diseases/ep [Epidemiology]. *Cognition Disorders/ep [Epidemiology]. Female. Humans. Incidence. Italy/ep [Epidemiology]. Male. Mass Screening/mt [Methods]. Middle Aged. Prevalence. Risk Factors. Surveys and Questionnaires. Ultrasonography.

Year of Publication

2006

Link to the Ovid Full Text or citation:

Click here for full text options

Link to the External Link Resolver:

SFX

119.

For peer review only - http://bmjopen.bmj.com/site/about/guidelines.xhtml

Carotid intima-media thickness and cognitive decline: what does it mean for prevention of dementia?.
Spence JD.
Journal of the Neurological Sciences. 223(2):103-5, 2004 Aug 30.
[Comment. Editorial]
UI: 15337609
Version ID
1
Record Owner
From MEDLINE, a database of the U.S. National Library of Medicine.
Status
MEDLINE
Authors Full Name
Spence, J David.
Comments
Comment on (CON)
MeSH Heading
Aged. Aged, 80 and over. Angiotensin-Converting Enzyme Inhibitors/tu [Therapeutic Use]. Carotid Artery, Common/pa [Pathology]. Cognition Disorders/dt [Drug Therapy]. *Cognition Disorders/pa [Pathology]. Dementia/co [Complications]. *Dementia/pa [Pathology]. *Dementia/pc [Prevention & Control]. Humans. Tunica Intima/de [Drug Effects]. *Tunica Intima/pa [Pathology]. Tunica Media/de [Drug Effects]. *Tunica Media/pa [Pathology].
Registry Number/Name of Substance
0 (Angiotensin-Converting Enzyme Inhibitors).
Year of Publication
2004

Link to the Ovid Full Text or citation:
Click here for full text options

Link to the External Link Resolver:
SFX

For peer review only

121.

Small dense low-density lipoprotein and carotid atherosclerosis in relation to vascular dementia.
Watanabe T; Koba S; Kawamura M; Itokawa M; Idei T; Nakagawa Y; Iguchi T; Katagiri T.
Metabolism: Clinical & Experimental. 53(4):476-82, 2004 Apr.
[Comparative Study. Journal Article]
UI: 15045695
Vascular dementia (VaD) and Alzheimer's disease (AD) are the most common causes of dementia in the elderly. The aim of this study was to investigate carotid atherosclerosis, serum lipid profiles, and atherogenic hormone levels in nondiabetic Japanese men with VaD or AD. Carotid artery intima-media thickness (IMT) and plaque, serum lipid and lipoprotein profiles, including low-density lipoprotein (LDL) particle size, as well as insulin-like growth factor-I (IGF-I, somatomedin C) and testosterone levels, were determined in 34 patients with AD, 37 patients with VaD, and 63 healthy male controls. Age, body mass index, systolic and diastolic blood pressure, and fasting plasma glucose, hemoglobin A(1c) (HbA(1c)), triglyceride, high-density lipoprotein (HDL)-cholesterol, and apolipoproteins (apo) A-I, B, and E levels did not differ significantly among the 3 groups. However, the mean value of carotid IMT, the frequency of atherosclerotic plaque deposition, the serum levels of LDL-cholesterol, lipoprotein(a), and lipid peroxides, and the incidence of small dense LDL (particle diameter </= 25.5 nm) were increased significantly in VaD patients compared with AD patients or controls. VaD patients had a close reverse correlation between carotid IMT and LDL particle diameter, which were statistically proven independent risk factors for VaD. In contrast, AD patients had significantly lower serum levels of IGF-I and testosterone than either VaD patients or controls. Our results indicate that VaD is associated with atherogenic dyslipidemia, in particular, small dense LDL and carotid atherosclerosis, whereas AD is associated with hyposomatomedinemia and hypogonadism rather than atherosclerosis.
Version ID
1
Record Owner
From MEDLINE, a database of the U.S. National Library of Medicine.
Status
MEDLINE
Authors Full Name
Watanabe, Takuya; Koba, Shinji; Kawamura, Mitsuharu; Itokawa, Masashi; Idei, Tsunenori; Nakagawa, Yukiko; Iguchi, Takashi; Katagiri, Takashi.
Institution
Watanabe, Takuya. Third Department of Internal Medicine, Showa University School of Medicine, Division of Internal Medicine, Showa University Karasuyama Hospital, Tokyo, Japan.

For peer review only

MeSH Heading
Aged. Aged, 80 and over. Alzheimer Disease/bl [Blood]. Alzheimer Disease/me [Metabolism]. *Carotid Artery Diseases/bl [Blood]. Carotid Artery Diseases/dg [Diagnostic Imaging]. *Dementia, Vascular/bl [Blood]. Dementia, Vascular/ep [Epidemiology]. Dementia, Vascular/me [Metabolism]. Humans. Insulin-Like Growth Factor I/me [Metabolism]. Lipid Peroxides/bl [Blood]. Lipoprotein(a)/bl [Blood]. *Lipoproteins, LDL/bl [Blood]. Male. Middle Aged. Particle Size. Risk Factors. Testosterone/bl [Blood]. Tunica Media/me [Metabolism]. Tunica Media/pa [Pathology]. Ultrasonography.
Registry Number/Name of Substance
0 (Lipid Peroxides). 0 (Lipoprotein(a)). 0 (Lipoproteins, LDL). 3XMK78S47O (Testosterone). 67763-96-6 (Insulin-Like Growth Factor I).
Year of Publication
2004

Link to the Ovid Full Text or citation:
Click here for full text options

Link to the External Link Resolver:
SFX

124.

Ultrasonographic assessment of carotid wall characteristics and cognitive functions in a community sample of 59- to 71-year-olds. The EVA Study Group.
Auperin A; Berr C; Bonithon-Kopp C; Touboul PJ; Ruelland I; Ducimetiere P; Alperovitch A.
Stroke. 27(8):1290-5, 1996 Aug.
[Journal Article]
UI: 8711788
BACKGROUND AND PURPOSE: This study was aimed at analyzing cross-sectional relationships between cognitive performance and ultrasonographic assessment of carotid wall characteristics. METHODS: A cohort of 1279 subjects (men, 41%) aged 59 to 71 years was recruited from the electoral rolls of the city of Nantes (western France). Cognitive performances were evaluated with the Mini-Mental State Examination (MMSE) and seven neuropsychological tests assessing

For peer review only - http://bmjopen.bmj.com/site/about/guidelines.xhtml

attention, psychomotor rapidity, verbal abilities, memory, and visuospatial perception. For each test, subjects were classified into three performance levels with a quartile distribution: 25% highest, 25% lowest, and 50% middle. The intima-media thickness of common carotid arteries and the presence of plaques in the carotid arteries were assessed with B-mode ultrasound examination.
RESULTS: Only 28% of men and 17% of women had carotid plaques inducing moderate stenosis of the lumen (< 40%). After adjustment for possible confounders, odds ratios for poor cognitive performance associated with plaques were above 1 for all cognitive tests in men. This association was statistically significant for the MMSE and another test assessing attention skills. There was a slight association between increase of the common carotid intima-media thickness and poor cognitive scores in men with plaques. In women, no association was found between cognitive functions and presence of plaques or intima-media thickness.
CONCLUSIONS: This study indicated a moderate association between atherosclerosis of the carotid arteries and poor cognitive functioning in men aged 59 to 71 years. In view of these moderate cross-sectional results, further studies are required to better assess the relationship between carotid atherosclerosis and cognitive impairment.
Version ID
1
Record Owner
From MEDLINE, a database of the U.S. National Library of Medicine.
Status
MEDLINE
Authors Full Name
Auperin, A; Berr, C; Bonithon-Kopp, C; Touboul, P J; Ruelland, I; Ducimetiere, P; Alperovitch, A.
Institution
Auperin, A. INSERM U360, Hopital de la Salpetriere, Nantes, France.
MeSH Heading
Aged. *Aging/ph [Physiology]. Arteriosclerosis/dg [Diagnostic Imaging]. Arteriosclerosis/ep [Epidemiology]. Arteriosclerosis/pa [Pathology]. *Carotid Arteries/dg [Diagnostic Imaging]. Carotid Arteries/pa [Pathology]. *Cognition/ph [Physiology]. Cross-Sectional Studies. Female. Humans. Longitudinal Studies. Male. Middle Aged. Multivariate Analysis. Prevalence. Sex Factors. Ultrasonography.
Year of Publication
1996

Link to the Ovid Full Text or citation:
Click here for full text options

For peer review only - http://bmjopen.bmj.com/site/about/guidelines.xhtml

Link to the External Link Resolver:

SFX

For peer review only

For peer review only - http://bmjopen.bmj.com/site/about/guidelines.xhtml

Eligibility Form: IS ASYMPTOMATIC ATHEROSCLEROSIS IN CAROTID ARTERIES ASSOCIATED WITH COGNITIVE FUNCTION?

A SYSTEMATIC REVIEW AND META-ANALYSIS

**1. Reference details**					
Reference citation					
First author					
Year of publication					
Title of the paper					
Assessor's identifier	1 ☐	2 ☐			
**2. Study eligibility**					
Inclusion of the study	Yes ☐ √	No ☐ √	Unclear r ☐		
**Eligibility criteria**	**Reason for exclusion (if excluded)**			Yes	No
ALL studies except : review articles, conference abstract, letters to the editor, case reports; pilot study,	☐ no MMSE cognitive test			☐	☐
cIMT measured by ultrasound	☐ No IMT Assessment			√	☐
	☐ Other imaging modality not ultrasound			☐	☐
	☐ review articles, conference abstract, letters to the editor, case reports			☐	☐
Notes:	☐ Duplicate			☐	☐

**Additional information**
Notes:

DO NOT PROCEED IF PAPER EXCLUDED FROM REVIEW

Data Extraction Form

3. Study details				
Study (cohort) name	The EVA Study			
Study design	longitudinal study			
Region/country	Nantes (western France).			
Sample size	1279			
Mean (range) age	Mean ± SD :<br>mean age, 65.0+/-3.0 years		Range (IQR):	
Sex	Male, n (%):<br>526 men		Female, n (%): 753 women	
Follow-up duration				
Clinical variables (n, %)				
Hypertension	n./a			
Diabetes mellitus	n/a			
**4. Exposure details**				
Hardware: Ultrasound Machine	an Aloka SSD-650 with a 7.5-MHz transducer.			
Method and site	The ultrasound examination involved scanning the common carotid arteries, the carotid bifurcations, and the first 2 cm of the internal carotid arteries. The IMT (distance between the media-adventitia interface and the lumen-intima interface) was automatically measured twice on longitudinal B-mode images of the far wall of each common carotid artery at plaque-free sites. The common carotid IMT was defined as the mean of four measurements. All segments of the carotid arteries were scanned longitudinally and transversely to assess the presence of plaques, and a localized echo-structure protruding into the lumen was considered to be a plaque if the distance between the media-adventitia interface and the internal side of the lesion was >or=to1 mm. The total number of plaques was recorded. For each, maximum thickness was measured perpendicular to the vessel wall. When several plaques were present in the same carotid segment (ie, common carotid artery or bifureation/origin of the internal carotid artery), plaque thickness measurement was made only for that with the greatest protrusion into the lumen. On the basis of transverse views, the degree of carotid stenosis was defined as (1-residual area/vessel area) times 100%.			

For peer review

Notes:

5. Outcomes' details	
Type has of outcomes used in this review	Outcomes reported in this study (n)
**Primary outcome(s)**<br>Global cognitive function	neuropsychological battery that included seven tests assessing different cognitive functions and a global test (MMSE) of 18 items roughly assessing various cognitive skills with scores ranging from 0 to 30.
Notes:	

6. Available number of participants					
					Number
Baseline sample size	Yes	☐	No	☐	
follow-up	Yes	☐	No	☐	
Total number included in the analysis	Yes	☐	No	☐	
All subjects accounted for	Yes	☐	No	☐	
Notes:					

7. Statistical analysis	
Statistical method used	Nominal polychotomous logistic regressions [13] (BMDP statistical software) were used to analyze the relationships between each cognitive score distribution of the eight neuropsychological tests and carotid characteristics (presence of plaques, IMT), adjusting for age, education, depressive symptomatology, systolic blood pressure, body mass index, smoking, and alcohol consumption.

For peer review only

For peer review only

A Modified Newcastle-Ottawa Quality Assessment for Observational Studies

The quality of included studies was assessed using a modified seven-point criteria derived from Newcastle-Ottawa scale (Wells et al., 2000). Each of the following criteria (A to G) was assigned 1 point if met.

For peer review only

Title and reference :				
**Criteria**				
**Sample**	**Yes**	**No**	**NR**	**Note**
A) Representativeness of the target sample<br>Yes = sampling designed to ensure adequate representativeness<br>No = inappropriately selected sample, or no description of sample selection				
B) Non-response satisfactorily dealt with<br>Yes = comparability between respondent and non-respondent characteristics established and response rate is satisfactory<br>No = the response rate is unsatisfactory, or the comparability between respondents and non-respondents is unsatisfactory, or no description of the response rate or the characteristics of the responders and the non-responders.				
**Exposure**	**Yes**	**No**	**NR**	**Note**
C) Validated measurement tool<br>Yes = high-resolution ultrasound system with linear ultrasound transducers at frequencies >7 MHz and measurement protocol for cIMT and plaque in accord* with American Society Echocardiography/Mannheim Consensus Guidelines. Data analysis performed independently and blinded using validated analysis system<br>No = inadequate ultrasound system, protocol, or analysis process, or not described				

* 'In accord' needs to be interpreted reasonably since some studies will precede these guidelines and others, while deviating slightly may not deviate sufficiently to be out of accord with the guidelines.

For peer review only

D) Relevant confounders measured† Yes = sex, age, smoking, hypertension/blood pressure, diabetes, hyperlipidemia/blood lipids/body mass index No = potential confounders not measured or not reported				
**Outcome**	**Yes**	**No**	**NR**	**Note**
E) Assessment of outcome Yes = independent blinded assessment of cognitive status using a validated instrument No = unvalidated instrument, or not described				
F) Statistical analysis Yes = statistical analysis clearly described and appropriate: estimate of the central tendency of the outcome with a measure of precision is provided (e.g. mean/median (95% confidence interval/standard deviation/range)). No = the statistical analysis is inappropriate, missing or inadequately described				
G) Missing data Yes = extent of missing data reported and the methods for addressing missing data described No = extent of missing data not reported and/or no description of how missing data were dealt with				
TOTAL (maximum 7)				

† relevant confounders listed here were selected on the basis of various components of the Framingham Cardiovascular risk score (https://framinghamheartstudy.org/fhs-risk-functions/cardiovascular-disease-10-year-risk/).

For peer review only

Title and reference :				
**Criteria**				
**Sample**	**Yes**	**No**	**NR**	**Note**
A) Representativeness of the target sample<br>Yes = sampling designed to ensure adequate representativeness<br>No = inappropriately selected sample, or no description of sample selection				
B) Non-response satisfactorily dealt with<br>Yes = comparability between respondent and non-respondent characteristics established and response rate is satisfactory<br>No = the response rate is unsatisfactory, or the comparability between respondents and non-respondents is unsatisfactory, or no description of the response rate or the characteristics of the responders and the non-responders.				
**Exposure**	**Yes**	**No**	**NR**	**Note**
C) Validated measurement tool<br>Yes = high-resolution ultrasound system with linear ultrasound transducers at frequencies >7 MHz and measurement protocol for cIMT and plaque in accord‡ with American Society Echocardiography/Mannheim Consensus Guidelines. Data analysis performed independently and blinded using validated analysis system<br>No = inadequate ultrasound system, protocol, or analysis process, or not described				

‡ 'In accord' needs to be interpreted reasonably since some studies will precede these guidelines and others, while deviating slightly may not deviate sufficiently to be out of accord with the guidelines.

For peer review only

	Yes	No	NR	Note
D) Relevant confounders measured[§]<br>Yes = sex, age, smoking, hypertension/blood pressure, diabetes, hyperlipidemia/blood lipids/body mass index<br>No = potential confounders not measured or not reported				
**Outcome**	**Yes**	**No**	**NR**	**Note**
E) Assessment of outcome<br>Yes = independent blinded assessment of cognitive status using a validated instrument<br>No = unvalidated instrument, or not described				
F) Statistical analysis<br>Yes = statistical analysis clearly described and appropriate: estimate of the central tendency of the outcome with a measure of precision is provided (e.g. mean/median (95% confidence interval/standard deviation/range)).<br>No = the statistical analysis is inappropriate, missing or inadequately described				
G) Missing data<br>Yes = extent of missing data reported and the methods for addressing missing data described<br>No = extent of missing data not reported and/or no description of how missing data were dealt with				
TOTAL (maximum 7)				

[§] relevant confounders listed here were selected on the basis of various components of the Framingham Cardiovascular risk score (https://framinghamheartstudy.org/fhs-risk-functions/cardiovascular-disease-10-year-risk/).

For peer review only

For peer review only

**Title and reference :**				
**Criteria**				
**Sample**	**Yes**	**No**	**NR**	**Note**
A) Representativeness of the target sample<br>Yes = sampling designed to ensure adequate representativeness<br>No = inappropriately selected sample, or no description of sample selection				
B) Non-response satisfactorily dealt with<br>Yes = comparability between respondent and non-respondent characteristics established and response rate is satisfactory<br>No = the response rate is unsatisfactory, or the comparability between respondents and non-respondents is unsatisfactory, or no description of the response rate or the characteristics of the responders and the non-responders.				
**Exposure**	**Yes**	**No**	**NR**	**Note**
C) Validated measurement tool<br>Yes = high-resolution ultrasound system with linear ultrasound transducers at frequencies >7 MHz and measurement protocol for cIMT and plaque in accord[**] with American Society Echocardiography/Mannheim Consensus Guidelines. Data analysis performed independently and blinded using validated analysis system<br>No = inadequate ultrasound system, protocol, or analysis process, or not described				
D) Relevant confounders measured[††]<br>Yes = sex, age, smoking, hypertension/blood pressure, diabetes, hyperlipidemia/blood lipids/body mass index<br>No = potential confounders not measured or not reported				

[**] 'In accord' needs to be interpreted reasonably since some studies will precede these guidelines and others, while deviating slightly may not deviate sufficiently to be out of accord with the guidelines.

[††] relevant confounders listed here were selected on the basis of various components of the Framingham Cardiovascular risk score (https://framinghamheartstudy.org/fhs-risk-functions/cardiovascular-disease-10-year-risk/).

For peer review only

Outcome	Yes	No	NR	Note
E) Assessment of outcome<br>Yes = independent blinded assessment of cognitive status using a validated instrument<br>No = unvalidated instrument, or not described				
F) Statistical analysis<br>Yes = statistical analysis clearly described and appropriate: estimate of the central tendency of the outcome with a measure of precision is provided (e.g. mean/median (95% confidence interval/standard deviation/range)).<br>No = the statistical analysis is inappropriate, missing or inadequately described				
G) Missing data<br>Yes = extent of missing data reported and the methods for addressing missing data described<br>No = extent of missing data not reported and/or no description of how missing data were dealt with				
TOTAL (maximum 7)				

For peer review only

**Title and reference :**				
**Criteria**				
**Sample**	**Yes**	**No**	**NR**	**Note**
A) Representativeness of the target sample<br>Yes = sampling designed to ensure adequate representativeness<br>No = inappropriately selected sample, or no description of sample selection				
B) Non-response satisfactorily dealt with<br>Yes = comparability between respondent and non-respondent characteristics established and response rate is satisfactory<br>No = the response rate is unsatisfactory, or the comparability between respondents and non-respondents is unsatisfactory, or no description of the response rate or the characteristics of the responders and the non-responders.				
**Exposure**	**Yes**	**No**	**NR**	**Note**
C) Validated measurement tool<br>Yes = high-resolution ultrasound system with linear ultrasound transducers at frequencies >7 MHz and measurement protocol for cIMT and plaque in accord[‡‡] with American Society Echocardiography/Mannheim Consensus Guidelines. Data analysis performed independently and blinded using validated analysis system<br>No = inadequate ultrasound system, protocol, or analysis process, or not described				
D) Relevant confounders measured[§§]<br>Yes = sex, age, smoking, hypertension/blood pressure, diabetes, hyperlipidemia/blood lipids/body mass index<br>No = potential confounders not measured or not reported				

[‡‡] 'In accord' needs to be interpreted reasonably since some studies will precede these guidelines and others, while deviating slightly may not deviate sufficiently to be out of accord with the guidelines.

[§§] relevant confounders listed here were selected on the basis of various components of the Framingham Cardiovascular risk score (https://framinghamheartstudy.org/fhs-risk-functions/cardiovascular-disease-10-year-risk/).

For peer review only

Outcome	Yes	No	NR	Note
E) Assessment of outcome<br>Yes = independent blinded assessment of cognitive status using a validated instrument<br>No = unvalidated instrument, or not described				
F) Statistical analysis<br>Yes = statistical analysis clearly described and appropriate: estimate of the central tendency of the outcome with a measure of precision is provided (e.g. mean/median (95% confidence interval/standard deviation/range)).<br>No = the statistical analysis is inappropriate, missing or inadequately described				
G) Missing data<br>Yes = extent of missing data reported and the methods for addressing missing data described<br>No = extent of missing data not reported and/or no description of how missing data were dealt with				
TOTAL (maximum 7)				

For peer review only

For peer review only

**Title and reference :**				
**Criteria**				
**Sample**	**Yes**	**No**	**NR**	**Note**
A) Representativeness of the target sample<br>Yes = sampling designed to ensure adequate representativeness<br>No = inappropriately selected sample, or no description of sample selection				
B) Non-response satisfactorily dealt with<br>Yes = comparability between respondent and non-respondent characteristics established and response rate is satisfactory<br>No = the response rate is unsatisfactory, or the comparability between respondents and non-respondents is unsatisfactory, or no description of the response rate or the characteristics of the responders and the non-responders.				
**Exposure**	**Yes**	**No**	**NR**	**Note**
C) Validated measurement tool<br>Yes = high-resolution ultrasound system with linear ultrasound transducers at frequencies >7 MHz and measurement protocol for cIMT and plaque in accord[***] with American Society Echocardiography/Mannheim Consensus Guidelines. Data analysis performed independently and blinded using validated analysis system<br>No = inadequate ultrasound system, protocol, or analysis process, or not described				
D) Relevant confounders measured[†††]<br>Yes = sex, age, smoking, hypertension/blood pressure, diabetes, hyperlipidemia/blood lipids/body mass index<br>No = potential confounders not measured or not reported				

[***] 'In accord' needs to be interpreted reasonably since some studies will precede these guidelines and others, while deviating slightly may not deviate sufficiently to be out of accord with the guidelines.
[†††] relevant confounders listed here were selected on the basis of various components of the Framingham Cardiovascular risk score (https://framinghamheartstudy.org/fhs-risk-functions/cardiovascular-disease-10-year-risk/).

For peer review only

Outcome	Yes	No	NR	Note
E) Assessment of outcome<br>Yes = independent blinded assessment of cognitive status using a validated instrument<br>No = unvalidated instrument, or not described				
F) Statistical analysis<br>Yes = statistical analysis clearly described and appropriate: estimate of the central tendency of the outcome with a measure of precision is provided (e.g. mean/median (95% confidence interval/standard deviation/range)).<br>No = the statistical analysis is inappropriate, missing or inadequately described				
G) Missing data<br>Yes = extent of missing data reported and the methods for addressing missing data described<br>No = extent of missing data not reported and/or no description of how missing data were dealt with				
TOTAL (maximum 7)				

For peer review only

For peer review only

Title and reference :				
**Criteria**				
**Sample**	**Yes**	**No**	**NR**	**Note**
A) Representativeness of the target sample<br>Yes = sampling designed to ensure adequate representativeness<br>No = inappropriately selected sample, or no description of sample selection				
B) Non-response satisfactorily dealt with<br>Yes = comparability between respondent and non-respondent characteristics established and response rate is satisfactory<br>No = the response rate is unsatisfactory, or the comparability between respondents and non-respondents is unsatisfactory, or no description of the response rate or the characteristics of the responders and the non-responders.				
**Exposure**	**Yes**	**No**	**NR**	**Note**
C) Validated measurement tool<br>Yes = high-resolution ultrasound system with linear ultrasound transducers at frequencies >7 MHz and measurement protocol for cIMT and plaque in accord[‡‡‡] with American Society Echocardiography/Mannheim Consensus Guidelines. Data analysis performed independently and blinded using validated analysis system<br>No = inadequate ultrasound system, protocol, or analysis process, or not described				
D) Relevant confounders measured[§§§]<br>Yes = sex, age, smoking, hypertension/blood pressure, diabetes, hyperlipidemia/blood lipids/body mass index<br>No = potential confounders not measured or not reported				

[‡‡‡] ‘In accord’ needs to be interpreted reasonably since some studies will precede these guidelines and others, while deviating slightly may not deviate sufficiently to be out of accord with the guidelines.
[§§§] relevant confounders listed here were selected on the basis of various components of the Framingham Cardiovascular risk score (https://framinghamheartstudy.org/fhs-risk-functions/cardiovascular-disease-10-year-risk/).

For peer review only

Outcome	Yes	No	NR	Note
E) Assessment of outcome<br>Yes = independent blinded assessment of cognitive status using a validated instrument<br>No = unvalidated instrument, or not described				
F) Statistical analysis<br>Yes = statistical analysis clearly described and appropriate: estimate of the central tendency of the outcome with a measure of precision is provided (e.g. mean/median (95% confidence interval/standard deviation/range)).<br>No = the statistical analysis is inappropriate, missing or inadequately described				
G) Missing data<br>Yes = extent of missing data reported and the methods for addressing missing data described<br>No = extent of missing data not reported and/or no description of how missing data were dealt with				
TOTAL (maximum 7)				

For peer review only

For peer review only

Title and reference :				
**Criteria**				
**Sample**	**Yes**	**No**	**NR**	**Note**
A) Representativeness of the target sample<br>Yes = sampling designed to ensure adequate representativeness<br>No = inappropriately selected sample, or no description of sample selection				
B) Non-response satisfactorily dealt with<br>Yes = comparability between respondent and non-respondent characteristics established and response rate is satisfactory<br>No = the response rate is unsatisfactory, or the comparability between respondents and non-respondents is unsatisfactory, or no description of the response rate or the characteristics of the responders and the non-responders.				
**Exposure**	**Yes**	**No**	**NR**	**Note**
C) Validated measurement tool<br>Yes = high-resolution ultrasound system with linear ultrasound transducers at frequencies >7 MHz and measurement protocol for cIMT and plaque in accord[****] with American Society Echocardiography/Mannheim Consensus Guidelines. Data analysis performed independently and blinded using validated analysis system<br>No = inadequate ultrasound system, protocol, or analysis process, or not described				
D) Relevant confounders measured[††††]<br>Yes = sex, age, smoking, hypertension/blood pressure, diabetes, hyperlipidemia/blood lipids/body mass index<br>No = potential confounders not measured or not reported				

[****] 'In accord' needs to be interpreted reasonably since some studies will precede these guidelines and others, while deviating slightly may not deviate sufficiently to be out of accord with the guidelines.
[††††] relevant confounders listed here were selected on the basis of various components of the Framingham Cardiovascular risk score (https://framinghamheartstudy.org/fhs-risk-functions/cardiovascular-disease-10-year-risk/).

For peer review only

Outcome	Yes	No	NR	Note
E) Assessment of outcome<br>Yes = independent blinded assessment of cognitive status using a validated instrument<br>No = unvalidated instrument, or not described				
F) Statistical analysis<br>Yes = statistical analysis clearly described and appropriate: estimate of the central tendency of the outcome with a measure of precision is provided (e.g. mean/median (95% confidence interval/standard deviation/range)).<br>No = the statistical analysis is inappropriate, missing or inadequately described				
G) Missing data<br>Yes = extent of missing data reported and the methods for addressing missing data described<br>No = extent of missing data not reported and/or no description of how missing data were dealt with				
TOTAL (maximum 7)				

For peer review only

For peer review only

Title and reference :				
**Criteria**				
**Sample**	**Yes**	**No**	**NR**	**Note**
A) Representativeness of the target sample<br>Yes = sampling designed to ensure adequate representativeness<br>No = inappropriately selected sample, or no description of sample selection				
B) Non-response satisfactorily dealt with<br>Yes = comparability between respondent and non-respondent characteristics established and response rate is satisfactory<br>No = the response rate is unsatisfactory, or the comparability between respondents and non-respondents is unsatisfactory, or no description of the response rate or the characteristics of the responders and the non-responders.				
**Exposure**	**Yes**	**No**	**NR**	**Note**
C) Validated measurement tool<br>Yes = high-resolution ultrasound system with linear ultrasound transducers at frequencies >7 MHz and measurement protocol for cIMT and plaque in accord[‡‡‡‡] with American Society Echocardiography/Mannheim Consensus Guidelines. Data analysis performed independently and blinded using validated analysis system<br>No = inadequate ultrasound system, protocol, or analysis process, or not described				
D) Relevant confounders measured[§§§§]<br>Yes = sex, age, smoking, hypertension/blood pressure, diabetes, hyperlipidemia/blood lipids/body mass index<br>No = potential confounders not measured or not reported				

[‡‡‡‡] ‘In accord’ needs to be interpreted reasonably since some studies will precede these guidelines and others, while deviating slightly may not deviate sufficiently to be out of accord with the guidelines.

[§§§§] relevant confounders listed here were selected on the basis of various components of the Framingham Cardiovascular risk score (https://framinghamheartstudy.org/fhs-risk-functions/cardiovascular-disease-10-year-risk/).

For peer review only

Outcome	Yes	No	NR	Note
E) Assessment of outcome<br>Yes = independent blinded assessment of cognitive status using a validated instrument<br>No = unvalidated instrument, or not described				
F) Statistical analysis<br>Yes = statistical analysis clearly described and appropriate: estimate of the central tendency of the outcome with a measure of precision is provided (e.g. mean/median (95% confidence interval/standard deviation/range)).<br>No = the statistical analysis is inappropriate, missing or inadequately described				
G) Missing data<br>Yes = extent of missing data reported and the methods for addressing missing data described<br>No = extent of missing data not reported and/or no description of how missing data were dealt with				
TOTAL (maximum 7)				

For peer review only

For peer review only

**Title and reference :**				
**Criteria**				
**Sample**	**Yes**	**No**	**NR**	**Note**
A) Representativeness of the target sample<br>Yes = sampling designed to ensure adequate representativeness<br>No = inappropriately selected sample, or no description of sample selection				
B) Non-response satisfactorily dealt with<br>Yes = comparability between respondent and non-respondent characteristics established and response rate is satisfactory<br>No = the response rate is unsatisfactory, or the comparability between respondents and non-respondents is unsatisfactory, or no description of the response rate or the characteristics of the responders and the non-responders.				
**Exposure**	**Yes**	**No**	**NR**	**Note**
C) Validated measurement tool<br>Yes = high-resolution ultrasound system with linear ultrasound transducers at frequencies >7 MHz and measurement protocol for cIMT and plaque in accord[*****] with American Society Echocardiography/Mannheim Consensus Guidelines. Data analysis performed independently and blinded using validated analysis system<br>No = inadequate ultrasound system, protocol, or analysis process, or not described				
D) Relevant confounders measured[†††††]<br>Yes = sex, age, smoking, hypertension/blood pressure, diabetes, hyperlipidemia/blood lipids/body mass index<br>No = potential confounders not measured or not reported				

[*****] 'In accord' needs to be interpreted reasonably since some studies will precede these guidelines and others, while deviating slightly may not deviate sufficiently to be out of accord with the guidelines.

[†††††] relevant confounders listed here were selected on the basis of various components of the Framingham Cardiovascular risk score (https://framinghamheartstudy.org/fhs-risk-functions/cardiovascular-disease-10-year-risk/).

Outcome	Yes	No	NR	Note
E) Assessment of outcome<br>Yes = independent blinded assessment of cognitive status using a validated instrument<br>No = unvalidated instrument, or not described				
F) Statistical analysis<br>Yes = statistical analysis clearly described and appropriate: estimate of the central tendency of the outcome with a measure of precision is provided (e.g. mean/median (95% confidence interval/standard deviation/range)).<br>No = the statistical analysis is inappropriate, missing or inadequately described				
G) Missing data<br>Yes = extent of missing data reported and the methods for addressing missing data described<br>No = extent of missing data not reported and/or no description of how missing data were dealt with				
TOTAL (maximum 7)				

References

Wells, G. et al. (2000) The Newcastle-Ottawa Scale (NOS) for assessing the quality of nonrandomised studies in meta-analyses, Ottawa, ON: Ottawa Hospital Research Institute.

Title and reference :				
**Criteria**				
**Sample**	**Yes**	**No**	**NR**	**Note**
A) Representativeness of the target sample<br>Yes = sampling designed to ensure adequate representativeness<br>No = inappropriately selected sample, or no description of sample selection				
B) Non-response satisfactorily dealt with<br>Yes = comparability between respondent and non-respondent characteristics established and response rate is satisfactory<br>No = the response rate is unsatisfactory, or the comparability between respondents and non-respondents is unsatisfactory, or no				

description of the response rate or the characteristics of the responders and the non-responders.				
**Exposure**	**Yes**	**No**	**NR**	**Note**
C) Validated measurement tool<br>Yes = high-resolution ultrasound system with linear ultrasound transducers at frequencies >7 MHz and measurement protocol for cIMT and plaque in accord[‡‡‡‡‡] with American Society Echocardiography/Mannheim Consensus Guidelines. Data analysis performed independently and blinded using validated analysis system<br>No = inadequate ultrasound system, protocol, or analysis process, or not described				
D) Relevant confounders measured[§§§§§]<br>Yes = sex, age, smoking, hypertension/blood pressure, diabetes, hyperlipidemia/blood lipids/body mass index<br>No = potential confounders not measured or not reported				
**Outcome**	**Yes**	**No**	**NR**	**Note**
E) Assessment of outcome<br>Yes = independent blinded assessment of cognitive status using a validated instrument<br>No = unvalidated instrument, or not described				
F) Statistical analysis<br>Yes = statistical analysis clearly described and appropriate: estimate of the central tendency of the outcome with a measure of precision is provided (e.g. mean/median (95% confidence interval/standard deviation/range)).<br>No = the statistical analysis is inappropriate, missing or inadequately described				

[‡‡‡‡‡] 'In accord' needs to be interpreted reasonably since some studies will precede these guidelines and others, while deviating slightly may not deviate sufficiently to be out of accord with the guidelines.
[§§§§§] relevant confounders listed here were selected on the basis of various components of the Framingham Cardiovascular risk score (https://framinghamheartstudy.org/fhs-risk-functions/cardiovascular-disease-10-year-risk/).

For peer review only

G) Missing data<br>Yes = extent of missing data reported and the methods for addressing missing data described<br>No = extent of missing data not reported and/or no description of how missing data were dealt with				
TOTAL (maximum 7)				

**Title and reference :**				
**Criteria**				
**Sample**	**Yes**	**No**	**NR**	**Note**
A) Representativeness of the target sample<br>Yes = sampling designed to ensure adequate representativeness<br>No = inappropriately selected sample, or no description of sample selection				
B) Non-response satisfactorily dealt with<br>Yes = comparability between respondent and non-respondent characteristics established and response rate is satisfactory<br>No = the response rate is unsatisfactory, or the comparability between respondents and non-respondents is unsatisfactory, or no description of the response rate or the characteristics of the responders and the non-responders.				
**Exposure**	**Yes**	**No**	**NR**	**Note**
C) Validated measurement tool<br>Yes = high-resolution ultrasound system with linear ultrasound transducers at frequencies >7 MHz and measurement protocol for cIMT and plaque in accord[******] with American Society Echocardiography/Mannheim Consensus Guidelines. Data analysis performed independently and blinded using validated analysis system<br>No = inadequate ultrasound system, protocol, or analysis process, or not described				

[******] 'In accord' needs to be interpreted reasonably since some studies will precede these guidelines and others, while deviating slightly may not deviate sufficiently to be out of accord with the guidelines.

For peer review only - http://bmjopen.bmj.com/site/about/guidelines.xhtml

D) Relevant confounders measured[††††††]<br>Yes = sex, age, smoking, hypertension/blood pressure, diabetes, hyperlipidemia/blood lipids/body mass index<br>No = potential confounders not measured or not reported				
**Outcome**	**Yes**	**No**	**NR**	**Note**
E) Assessment of outcome<br>Yes = independent blinded assessment of cognitive status using a validated instrument<br>No = unvalidated instrument, or not described				
F) Statistical analysis<br>Yes = statistical analysis clearly described and appropriate: estimate of the central tendency of the outcome with a measure of precision is provided (e.g. mean/median (95% confidence interval/standard deviation/range)).<br>No = the statistical analysis is inappropriate, missing or inadequately described				
G) Missing data<br>Yes = extent of missing data reported and the methods for addressing missing data described<br>No = extent of missing data not reported and/or no description of how missing data were dealt with				
TOTAL (maximum 7)				

**Title and reference :**				
**Criteria**				
**Sample**	**Yes**	**No**	**NR**	**Note**
A) Representativeness of the target sample<br>Yes = sampling designed to ensure adequate representativeness<br>No = inappropriately selected sample, or no description of sample selection				
B) Non-response satisfactorily dealt with				

[††††††] relevant confounders listed here were selected on the basis of various components of the Framingham Cardiovascular risk score (https://framinghamheartstudy.org/fhs-risk-functions/cardiovascular-disease-10-year-risk/).

For peer review only

Yes = comparability between respondent and non-respondent characteristics established and response rate is satisfactory<br>No = the response rate is unsatisfactory, or the comparability between respondents and non-respondents is unsatisfactory, or no description of the response rate or the characteristics of the responders and the non-responders.				
**Exposure**	**Yes**	**No**	**NR**	**Note**
C) Validated measurement tool<br>Yes = high-resolution ultrasound system with linear ultrasound transducers at frequencies >7 MHz and measurement protocol for cIMT and plaque in accord[‡‡‡‡‡‡] with American Society Echocardiography/Mannheim Consensus Guidelines. Data analysis performed independently and blinded using validated analysis system<br>No = inadequate ultrasound system, protocol, or analysis process, or not described				
D) Relevant confounders measured[§§§§§§]<br>Yes = sex, age, smoking, hypertension/blood pressure, diabetes, hyperlipidemia/blood lipids/body mass index<br>No = potential confounders not measured or not reported				
**Outcome**	**Yes**	**No**	**NR**	**Note**
E) Assessment of outcome<br>Yes = independent blinded assessment of cognitive status using a validated instrument<br>No = unvalidated instrument, or not described				

[‡‡‡‡‡‡] 'In accord' needs to be interpreted reasonably since some studies will precede these guidelines and others, while deviating slightly may not deviate sufficiently to be out of accord with the guidelines.
[§§§§§§] relevant confounders listed here were selected on the basis of various components of the Framingham Cardiovascular risk score (https://framinghamheartstudy.org/fhs-risk-functions/cardiovascular-disease-10-year-risk/).

For peer review only

F) Statistical analysis<br>Yes = statistical analysis clearly described and appropriate: estimate of the central tendency of the outcome with a measure of precision is provided (e.g. mean/median (95% confidence interval/standard deviation/range)).<br>No = the statistical analysis is inappropriate, missing or inadequately described				
G) Missing data<br>Yes = extent of missing data reported and the methods for addressing missing data described<br>No = extent of missing data not reported and/or no description of how missing data were dealt with				
TOTAL (maximum 7)				

**Title and reference :**				
**Criteria**				
**Sample**	**Yes**	**No**	**NR**	**Note**
A) Representativeness of the target sample<br>Yes = sampling designed to ensure adequate representativeness<br>No = inappropriately selected sample, or no description of sample selection				
B) Non-response satisfactorily dealt with<br>Yes = comparability between respondent and non-respondent characteristics established and response rate is satisfactory<br>No = the response rate is unsatisfactory, or the comparability between respondents and non-respondents is unsatisfactory, or no description of the response rate or the characteristics of the responders and the non-responders.				
**Exposure**	**Yes**	**No**	**NR**	**Note**

For peer review only

C) Validated measurement tool<br>Yes = high-resolution ultrasound system with linear ultrasound transducers at frequencies >7 MHz and measurement protocol for cIMT and plaque in accord[*******] with American Society Echocardiography/Mannheim Consensus Guidelines. Data analysis performed independently and blinded using validated analysis system<br>No = inadequate ultrasound system, protocol, or analysis process, or not described				
D) Relevant confounders measured[†††††††]<br>Yes = sex, age, smoking, hypertension/blood pressure, diabetes, hyperlipidemia/blood lipids/body mass index<br>No = potential confounders not measured or not reported				
**Outcome**	**Yes**	**No**	**NR**	**Note**
E) Assessment of outcome<br>Yes = independent blinded assessment of cognitive status using a validated instrument<br>No = unvalidated instrument, or not described				
F) Statistical analysis<br>Yes = statistical analysis clearly described and appropriate: estimate of the central tendency of the outcome with a measure of precision is provided (e.g. mean/median (95% confidence interval/standard deviation/range)).<br>No = the statistical analysis is inappropriate, missing or inadequately described				

[*******] ‘In accord’ needs to be interpreted reasonably since some studies will precede these guidelines and others, while deviating slightly may not deviate sufficiently to be out of accord with the guidelines.
[†††††††] relevant confounders listed here were selected on the basis of various components of the Framingham Cardiovascular risk score (https://framinghamheartstudy.org/fhs-risk-functions/cardiovascular-disease-10-year-risk/).

For peer review only

G) Missing data<br>Yes = extent of missing data reported and the methods for addressing missing data described<br>No = extent of missing data not reported and/or no description of how missing data were dealt with				
TOTAL (maximum 7)				

**Title and reference :**				
**Criteria**				
**Sample**	**Yes**	**No**	**NR**	**Note**
A) Representativeness of the target sample<br>Yes = sampling designed to ensure adequate representativeness<br>No = inappropriately selected sample, or no description of sample selection				
B) Non-response satisfactorily dealt with<br>Yes = comparability between respondent and non-respondent characteristics established and response rate is satisfactory<br>No = the response rate is unsatisfactory, or the comparability between respondents and non-respondents is unsatisfactory, or no description of the response rate or the characteristics of the responders and the non-responders.				
**Exposure**	**Yes**	**No**	**NR**	**Note**
C) Validated measurement tool<br>Yes = high-resolution ultrasound system with linear ultrasound transducers at frequencies >7 MHz and measurement protocol for cIMT and plaque in accord[‡‡‡‡‡‡‡] with American Society Echocardiography/Mannheim Consensus Guidelines. Data analysis performed independently and blinded using validated analysis system<br>No = inadequate ultrasound system, protocol, or analysis process, or not described				

[‡‡‡‡‡‡‡] ‘In accord’ needs to be interpreted reasonably since some studies will precede these guidelines and others, while deviating slightly may not deviate sufficiently to be out of accord with the guidelines.

For peer review only

D) Relevant confounders measured[§§§§§§§]<br>Yes = sex, age, smoking, hypertension/blood pressure, diabetes, hyperlipidemia/blood lipids/body mass index<br>No = potential confounders not measured or not reported				
**Outcome**	**Yes**	**No**	**NR**	**Note**
E) Assessment of outcome<br>Yes = independent blinded assessment of cognitive status using a validated instrument<br>No = unvalidated instrument, or not described				
F) Statistical analysis<br>Yes = statistical analysis clearly described and appropriate: estimate of the central tendency of the outcome with a measure of precision is provided (e.g. mean/median (95% confidence interval/standard deviation/range)).<br>No = the statistical analysis is inappropriate, missing or inadequately described				
G) Missing data<br>Yes = extent of missing data reported and the methods for addressing missing data described<br>No = extent of missing data not reported and/or no description of how missing data were dealt with				
TOTAL (maximum 7)				

For peer review only

**Title and reference :**				
**Criteria**				
**Sample**	**Yes**	**No**	**NR**	**Note**
A) Representativeness of the target sample<br>Yes = sampling designed to ensure adequate representativeness<br>No = inappropriately selected sample, or no description of sample selection				
B) Non-response satisfactorily dealt with				

[§§§§§§§] relevant confounders listed here were selected on the basis of various components of the Framingham Cardiovascular risk score (https://framinghamheartstudy.org/fhs-risk-functions/cardiovascular-disease-10-year-risk/).

Yes = comparability between respondent and non-respondent characteristics established and response rate is satisfactory<br>No = the response rate is unsatisfactory, or the comparability between respondents and non-respondents is unsatisfactory, or no description of the response rate or the characteristics of the responders and the non-responders.				
**Exposure**	**Yes**	**No**	**NR**	**Note**
C) Validated measurement tool<br>Yes = high-resolution ultrasound system with linear ultrasound transducers at frequencies >7 MHz and measurement protocol for cIMT and plaque in accord[********] with American Society Echocardiography/Mannheim Consensus Guidelines. Data analysis performed independently and blinded using validated analysis system<br>No = inadequate ultrasound system, protocol, or analysis process, or not described				
D) Relevant confounders measured[††††††††]<br>Yes = sex, age, smoking, hypertension/blood pressure, diabetes, hyperlipidemia/blood lipids/body mass index<br>No = potential confounders not measured or not reported				
**Outcome**	**Yes**	**No**	**NR**	**Note**
E) Assessment of outcome<br>Yes = independent blinded assessment of cognitive status using a validated instrument<br>No = unvalidated instrument, or not described				

[********] 'In accord' needs to be interpreted reasonably since some studies will precede these guidelines and others, while deviating slightly may not deviate sufficiently to be out of accord with the guidelines.

[††††††††] relevant confounders listed here were selected on the basis of various components of the Framingham Cardiovascular risk score (https://framinghamheartstudy.org/fhs-risk-functions/cardiovascular-disease-10-year-risk/).

For peer review only

F) Statistical analysis<br>Yes = statistical analysis clearly described and appropriate: estimate of the central tendency of the outcome with a measure of precision is provided (e.g. mean/median (95% confidence interval/standard deviation/range)).<br>No = the statistical analysis is inappropriate, missing or inadequately described				
G) Missing data<br>Yes = extent of missing data reported and the methods for addressing missing data described<br>No = extent of missing data not reported and/or no description of how missing data were dealt with				
TOTAL (maximum 7)				

**Title and reference :**				
**Criteria**				
**Sample**	**Yes**	**No**	**NR**	**Note**
A) Representativeness of the target sample<br>Yes = sampling designed to ensure adequate representativeness<br>No = inappropriately selected sample, or no description of sample selection				
B) Non-response satisfactorily dealt with<br>Yes = comparability between respondent and non-respondent characteristics established and response rate is satisfactory<br>No = the response rate is unsatisfactory, or the comparability between respondents and non-respondents is unsatisfactory, or no description of the response rate or the characteristics of the responders and the non-responders.				
**Exposure**	**Yes**	**No**	**NR**	**Note**

For peer review only

For peer review only - http://bmjopen.bmj.com/site/about/guidelines.xhtml

C) Validated measurement tool<br>Yes = high-resolution ultrasound system with linear ultrasound transducers at frequencies >7 MHz and measurement protocol for cIMT and plaque in accord[‡‡‡‡‡‡‡‡] with American Society Echocardiography/Mannheim Consensus Guidelines. Data analysis performed independently and blinded using validated analysis system<br>No = inadequate ultrasound system, protocol, or analysis process, or not described				
D) Relevant confounders measured[§§§§§§§§]<br>Yes = sex, age, smoking, hypertension/blood pressure, diabetes, hyperlipidemia/blood lipids/body mass index<br>No = potential confounders not measured or not reported				
**Outcome**	**Yes**	**No**	**NR**	**Note**
E) Assessment of outcome<br>Yes = independent blinded assessment of cognitive status using a validated instrument<br>No = unvalidated instrument, or not described				
F) Statistical analysis<br>Yes = statistical analysis clearly described and appropriate: estimate of the central tendency of the outcome with a measure of precision is provided (e.g. mean/median (95% confidence interval/standard deviation/range)).<br>No = the statistical analysis is inappropriate, missing or inadequately described				

For peer review only

‡‡‡‡‡‡‡‡ 'In accord' needs to be interpreted reasonably since some studies will precede these guidelines and others, while deviating slightly may not deviate sufficiently to be out of accord with the guidelines.
§§§§§§§§ relevant confounders listed here were selected on the basis of various components of the Framingham Cardiovascular risk score (https://framinghamheartstudy.org/fhs-risk-functions/cardiovascular-disease-10-year-risk/).

G) Missing data<br>Yes = extent of missing data reported and the methods for addressing missing data described<br>No = extent of missing data not reported and/or no description of how missing data were dealt with				
TOTAL (maximum 7)				

**Title and reference :**				
**Criteria**				
**Sample**	**Yes**	**No**	**NR**	**Note**
A) Representativeness of the target sample<br>Yes = sampling designed to ensure adequate representativeness<br>No = inappropriately selected sample, or no description of sample selection				
B) Non-response satisfactorily dealt with<br>Yes = comparability between respondent and non-respondent characteristics established and response rate is satisfactory<br>No = the response rate is unsatisfactory, or the comparability between respondents and non-respondents is unsatisfactory, or no description of the response rate or the characteristics of the responders and the non-responders.				
**Exposure**	**Yes**	**No**	**NR**	**Note**
C) Validated measurement tool<br>Yes = high-resolution ultrasound system with linear ultrasound transducers at frequencies >7 MHz and measurement protocol for cIMT and plaque in accord********* with American Society Echocardiography/Mannheim Consensus Guidelines. Data analysis performed independently and blinded using validated analysis system<br>No = inadequate ultrasound system, protocol, or analysis process, or not described				

For peer review only

********* ‘In accord’ needs to be interpreted reasonably since some studies will precede these guidelines and others, while deviating slightly may not deviate sufficiently to be out of accord with the guidelines.

For peer review only - http://bmjopen.bmj.com/site/about/guidelines.xhtml

D) Relevant confounders measured[†††††††††]<br>Yes = sex, age, smoking, hypertension/blood pressure, diabetes, hyperlipidemia/blood lipids/body mass index<br>No = potential confounders not measured or not reported				
**Outcome**	**Yes**	**No**	**NR**	**Note**
E) Assessment of outcome<br>Yes = independent blinded assessment of cognitive status using a validated instrument<br>No = unvalidated instrument, or not described				
F) Statistical analysis<br>Yes = statistical analysis clearly described and appropriate: estimate of the central tendency of the outcome with a measure of precision is provided (e.g. mean/median (95% confidence interval/standard deviation/range)).<br>No = the statistical analysis is inappropriate, missing or inadequately described				
G) Missing data<br>Yes = extent of missing data reported and the methods for addressing missing data described<br>No = extent of missing data not reported and/or no description of how missing data were dealt with				
TOTAL (maximum 7)				

**Title and reference :**				
**Criteria**				
**Sample**	**Yes**	**No**	**NR**	**Note**
A) Representativeness of the target sample<br>Yes = sampling designed to ensure adequate representativeness<br>No = inappropriately selected sample, or no description of sample selection				
B) Non-response satisfactorily dealt with				

[†††††††††] relevant confounders listed here were selected on the basis of various components of the Framingham Cardiovascular risk score (https://framinghamheartstudy.org/fhs-risk-functions/cardiovascular-disease-10-year-risk/).

For peer review only

Yes = comparability between respondent and non-respondent characteristics established and response rate is satisfactory<br>No = the response rate is unsatisfactory, or the comparability between respondents and non-respondents is unsatisfactory, or no description of the response rate or the characteristics of the responders and the non-responders.				
**Exposure**	**Yes**	**No**	**NR**	**Note**
C) Validated measurement tool<br>Yes = high-resolution ultrasound system with linear ultrasound transducers at frequencies >7 MHz and measurement protocol for cIMT and plaque in accord[‡‡‡‡‡‡‡‡‡] with American Society Echocardiography/Mannheim Consensus Guidelines. Data analysis performed independently and blinded using validated analysis system<br>No = inadequate ultrasound system, protocol, or analysis process, or not described				
D) Relevant confounders measured[§§§§§§§§§]<br>Yes = sex, age, smoking, hypertension/blood pressure, diabetes, hyperlipidemia/blood lipids/body mass index<br>No = potential confounders not measured or not reported				
**Outcome**	**Yes**	**No**	**NR**	**Note**
E) Assessment of outcome<br>Yes = independent blinded assessment of cognitive status using a validated instrument<br>No = unvalidated instrument, or not described				

[‡‡‡‡‡‡‡‡‡] ‘In accord’ needs to be interpreted reasonably since some studies will precede these guidelines and others, while deviating slightly may not deviate sufficiently to be out of accord with the guidelines.

[§§§§§§§§§] relevant confounders listed here were selected on the basis of various components of the Framingham Cardiovascular risk score (https://framinghamheartstudy.org/fhs-risk-functions/cardiovascular-disease-10-year-risk/).

For peer review only

F) Statistical analysis<br>Yes = statistical analysis clearly described and appropriate: estimate of the central tendency of the outcome with a measure of precision is provided (e.g. mean/median (95% confidence interval/standard deviation/range)).<br>No = the statistical analysis is inappropriate, missing or inadequately described				
G) Missing data<br>Yes = extent of missing data reported and the methods for addressing missing data described<br>No = extent of missing data not reported and/or no description of how missing data were dealt with				
TOTAL (maximum 7)				

**Title and reference :**				
**Criteria**				
**Sample**	**Yes**	**No**	**NR**	**Note**
A) Representativeness of the target sample<br>Yes = sampling designed to ensure adequate representativeness<br>No = inappropriately selected sample, or no description of sample selection				
B) Non-response satisfactorily dealt with<br>Yes = comparability between respondent and non-respondent characteristics established and response rate is satisfactory<br>No = the response rate is unsatisfactory, or the comparability between respondents and non-respondents is unsatisfactory, or no description of the response rate or the characteristics of the responders and the non-responders.				
**Exposure**	**Yes**	**No**	**NR**	**Note**

For peer review only

C) Validated measurement tool<br>Yes = high-resolution ultrasound system with linear ultrasound transducers at frequencies >7 MHz and measurement protocol for cIMT and plaque in accord[**********] with American Society Echocardiography/Mannheim Consensus Guidelines. Data analysis performed independently and blinded using validated analysis system<br>No = inadequate ultrasound system, protocol, or analysis process, or not described				
D) Relevant confounders measured[††††††††††]<br>Yes = sex, age, smoking, hypertension/blood pressure, diabetes, hyperlipidemia/blood lipids/body mass index<br>No = potential confounders not measured or not reported				
**Outcome**	**Yes**	**No**	**NR**	**Note**
E) Assessment of outcome<br>Yes = independent blinded assessment of cognitive status using a validated instrument<br>No = unvalidated instrument, or not described				
F) Statistical analysis<br>Yes = statistical analysis clearly described and appropriate: estimate of the central tendency of the outcome with a measure of precision is provided (e.g. mean/median (95% confidence interval/standard deviation/range)).<br>No = the statistical analysis is inappropriate, missing or inadequately described				

[**********] 'In accord' needs to be interpreted reasonably since some studies will precede these guidelines and others, while deviating slightly may not deviate sufficiently to be out of accord with the guidelines.

[††††††††††] relevant confounders listed here were selected on the basis of various components of the Framingham Cardiovascular risk score (https://framinghamheartstudy.org/fhs-risk-functions/cardiovascular-disease-10-year-risk/).

For peer review only - http://bmjopen.bmj.com/site/about/guidelines.xhtml

G) Missing data<br>Yes = extent of missing data reported and the methods for addressing missing data described<br>No = extent of missing data not reported and/or no description of how missing data were dealt with				
TOTAL (maximum 7)				

**Title and reference :**				
**Criteria**				
**Sample**	**Yes**	**No**	**NR**	**Note**
A) Representativeness of the target sample<br>Yes = sampling designed to ensure adequate representativeness<br>No = inappropriately selected sample, or no description of sample selection				
B) Non-response satisfactorily dealt with<br>Yes = comparability between respondent and non-respondent characteristics established and response rate is satisfactory<br>No = the response rate is unsatisfactory, or the comparability between respondents and non-respondents is unsatisfactory, or no description of the response rate or the characteristics of the responders and the non-responders.				
**Exposure**	**Yes**	**No**	**NR**	**Note**
C) Validated measurement tool<br>Yes = high-resolution ultrasound system with linear ultrasound transducers at frequencies >7 MHz and measurement protocol for cIMT and plaque in accord[‡‡‡‡‡‡‡‡‡‡] with American Society Echocardiography/Mannheim Consensus Guidelines. Data analysis performed independently and blinded using validated analysis system<br>No = inadequate ultrasound system, protocol, or analysis process, or not described				

[‡‡‡‡‡‡‡‡‡‡] 'In accord' needs to be interpreted reasonably since some studies will precede these guidelines and others, while deviating slightly may not deviate sufficiently to be out of accord with the guidelines.

For peer review only

D) Relevant confounders measured[§§§§§§§§§§§]<br>Yes = sex, age, smoking, hypertension/blood pressure, diabetes, hyperlipidemia/blood lipids/body mass index<br>No = potential confounders not measured or not reported				
**Outcome**	**Yes**	**No**	**NR**	**Note**
E) Assessment of outcome<br>Yes = independent blinded assessment of cognitive status using a validated instrument<br>No = unvalidated instrument, or not described				
F) Statistical analysis<br>Yes = statistical analysis clearly described and appropriate: estimate of the central tendency of the outcome with a measure of precision is provided (e.g. mean/median (95% confidence interval/standard deviation/range)).<br>No = the statistical analysis is inappropriate, missing or inadequately described				
G) Missing data<br>Yes = extent of missing data reported and the methods for addressing missing data described<br>No = extent of missing data not reported and/or no description of how missing data were dealt with				
TOTAL (maximum 7)				

**Title and reference :**				
**Criteria**				
**Sample**	**Yes**	**No**	**NR**	**Note**
A) Representativeness of the target sample<br>Yes = sampling designed to ensure adequate representativeness<br>No = inappropriately selected sample, or no description of sample selection				
B) Non-response satisfactorily dealt with				

[§§§§§§§§§§§] relevant confounders listed here were selected on the basis of various components of the Framingham Cardiovascular risk score (https://framinghamheartstudy.org/fhs-risk-functions/cardiovascular-disease-10-year-risk/).

For peer review only

	Yes	No	NR	Note
Yes = comparability between respondent and non-respondent characteristics established and response rate is satisfactory<br>No = the response rate is unsatisfactory, or the comparability between respondents and non-respondents is unsatisfactory, or no description of the response rate or the characteristics of the responders and the non-responders.				
**Exposure**	**Yes**	**No**	**NR**	**Note**
C) Validated measurement tool<br>Yes = high-resolution ultrasound system with linear ultrasound transducers at frequencies >7 MHz and measurement protocol for cIMT and plaque in accord[***********] with American Society Echocardiography/Mannheim Consensus Guidelines. Data analysis performed independently and blinded using validated analysis system<br>No = inadequate ultrasound system, protocol, or analysis process, or not described				
D) Relevant confounders measured[†††††††††††]<br>Yes = sex, age, smoking, hypertension/blood pressure, diabetes, hyperlipidemia/blood lipids/body mass index<br>No = potential confounders not measured or not reported				
**Outcome**	**Yes**	**No**	**NR**	**Note**
E) Assessment of outcome<br>Yes = independent blinded assessment of cognitive status using a validated instrument<br>No = unvalidated instrument, or not described				

[***********] 'In accord' needs to be interpreted reasonably since some studies will precede these guidelines and others, while deviating slightly may not deviate sufficiently to be out of accord with the guidelines.

[†††††††††††] relevant confounders listed here were selected on the basis of various components of the Framingham Cardiovascular risk score (https://framinghamheartstudy.org/fhs-risk-functions/cardiovascular-disease-10-year-risk/).

For peer review only

F) Statistical analysis<br>Yes = statistical analysis clearly described and appropriate: estimate of the central tendency of the outcome with a measure of precision is provided (e.g. mean/median (95% confidence interval/standard deviation/range)).<br>No = the statistical analysis is inappropriate, missing or inadequately described				
G) Missing data<br>Yes = extent of missing data reported and the methods for addressing missing data described<br>No = extent of missing data not reported and/or no description of how missing data were dealt with				
TOTAL (maximum 7)				

**Title and reference :**				
**Criteria**				
**Sample**	**Yes**	**No**	**NR**	**Note**
A) Representativeness of the target sample<br>Yes = sampling designed to ensure adequate representativeness<br>No = inappropriately selected sample, or no description of sample selection				
B) Non-response satisfactorily dealt with<br>Yes = comparability between respondent and non-respondent characteristics established and response rate is satisfactory<br>No = the response rate is unsatisfactory, or the comparability between respondents and non-respondents is unsatisfactory, or no description of the response rate or the characteristics of the responders and the non-responders.				
**Exposure**	**Yes**	**No**	**NR**	**Note**

For peer review only

C) Validated measurement tool<br>Yes = high-resolution ultrasound system with linear ultrasound transducers at frequencies >7 MHz and measurement protocol for cIMT and plaque in accord[‡‡‡‡‡‡‡‡‡‡‡‡] with American Society Echocardiography/Mannheim Consensus Guidelines. Data analysis performed independently and blinded using validated analysis system<br>No = inadequate ultrasound system, protocol, or analysis process, or not described				
D) Relevant confounders measured[§§§§§§§§§§§§]<br>Yes = sex, age, smoking, hypertension/blood pressure, diabetes, hyperlipidemia/blood lipids/body mass index<br>No = potential confounders not measured or not reported				
**Outcome**	**Yes**	**No**	**NR**	**Note**
E) Assessment of outcome<br>Yes = independent blinded assessment of cognitive status using a validated instrument<br>No = unvalidated instrument, or not described				
F) Statistical analysis<br>Yes = statistical analysis clearly described and appropriate: estimate of the central tendency of the outcome with a measure of precision is provided (e.g. mean/median (95% confidence interval/standard deviation/range)).<br>No = the statistical analysis is inappropriate, missing or inadequately described				

For peer review only

[‡‡‡‡‡‡‡‡‡‡‡‡] 'In accord' needs to be interpreted reasonably since some studies will precede these guidelines and others, while deviating slightly may not deviate sufficiently to be out of accord with the guidelines.

[§§§§§§§§§§§§] relevant confounders listed here were selected on the basis of various components of the Framingham Cardiovascular risk score (https://framinghamheartstudy.org/fhs-risk-functions/cardiovascular-disease-10-year-risk/).

G) Missing data<br>Yes = extent of missing data reported and the methods for addressing missing data described<br>No = extent of missing data not reported and/or no description of how missing data were dealt with				
TOTAL (maximum 7)				

**Title and reference :**				
**Criteria**				
**Sample**	**Yes**	**No**	**NR**	**Note**
A) Representativeness of the target sample<br>Yes = sampling designed to ensure adequate representativeness<br>No = inappropriately selected sample, or no description of sample selection				
B) Non-response satisfactorily dealt with<br>Yes = comparability between respondent and non-respondent characteristics established and response rate is satisfactory<br>No = the response rate is unsatisfactory, or the comparability between respondents and non-respondents is unsatisfactory, or no description of the response rate or the characteristics of the responders and the non-responders.				
**Exposure**	**Yes**	**No**	**NR**	**Note**
C) Validated measurement tool<br>Yes = high-resolution ultrasound system with linear ultrasound transducers at frequencies >7 MHz and measurement protocol for cIMT and plaque in accord[************] with American Society Echocardiography/Mannheim Consensus Guidelines. Data analysis performed independently and blinded using validated analysis system<br>No = inadequate ultrasound system, protocol, or analysis process, or not described				

[************] 'In accord' needs to be interpreted reasonably since some studies will precede these guidelines and others, while deviating slightly may not deviate sufficiently to be out of accord with the guidelines.

For peer review only - http://bmjopen.bmj.com/site/about/guidelines.xhtml

D) Relevant confounders measured[††††††††††††]<br>Yes = sex, age, smoking, hypertension/blood pressure, diabetes, hyperlipidemia/blood lipids/body mass index<br>No = potential confounders not measured or not reported				
**Outcome**	**Yes**	**No**	**NR**	**Note**
E) Assessment of outcome<br>Yes = independent blinded assessment of cognitive status using a validated instrument<br>No = unvalidated instrument, or not described				
F) Statistical analysis<br>Yes = statistical analysis clearly described and appropriate: estimate of the central tendency of the outcome with a measure of precision is provided (e.g. mean/median (95% confidence interval/standard deviation/range)).<br>No = the statistical analysis is inappropriate, missing or inadequately described				
G) Missing data<br>Yes = extent of missing data reported and the methods for addressing missing data described<br>No = extent of missing data not reported and/or no description of how missing data were dealt with				
TOTAL (maximum 7)				

**Title and reference :**				
**Criteria**				
**Sample**	**Yes**	**No**	**NR**	**Note**
A) Representativeness of the target sample<br>Yes = sampling designed to ensure adequate representativeness<br>No = inappropriately selected sample, or no description of sample selection				
B) Non-response satisfactorily dealt with				

[††††††††††††] relevant confounders listed here were selected on the basis of various components of the Framingham Cardiovascular risk score (https://framinghamheartstudy.org/fhs-risk-functions/cardiovascular-disease-10-year-risk/).

For peer review only

Yes = comparability between respondent and non-respondent characteristics established and response rate is satisfactory<br>No = the response rate is unsatisfactory, or the comparability between respondents and non-respondents is unsatisfactory, or no description of the response rate or the characteristics of the responders and the non-responders.				
**Exposure**	**Yes**	**No**	**NR**	**Note**
C) Validated measurement tool<br>Yes = high-resolution ultrasound system with linear ultrasound transducers at frequencies >7 MHz and measurement protocol for cIMT and plaque in accord[‡‡‡‡‡‡‡‡‡‡‡‡‡] with American Society Echocardiography/Mannheim Consensus Guidelines. Data analysis performed independently and blinded using validated analysis system<br>No = inadequate ultrasound system, protocol, or analysis process, or not described				
D) Relevant confounders measured[§§§§§§§§§§§§]<br>Yes = sex, age, smoking, hypertension/blood pressure, diabetes, hyperlipidemia/blood lipids/body mass index<br>No = potential confounders not measured or not reported				
**Outcome**	**Yes**	**No**	**NR**	**Note**
E) Assessment of outcome<br>Yes = independent blinded assessment of cognitive status using a validated instrument<br>No = unvalidated instrument, or not described				

[‡‡‡‡‡‡‡‡‡‡‡‡‡] 'In accord' needs to be interpreted reasonably since some studies will precede these guidelines and others, while deviating slightly may not deviate sufficiently to be out of accord with the guidelines.
[§§§§§§§§§§§§] relevant confounders listed here were selected on the basis of various components of the Framingham Cardiovascular risk score (https://framinghamheartstudy.org/fhs-risk-functions/cardiovascular-disease-10-year-risk/).

For peer review only

F) Statistical analysis<br>Yes = statistical analysis clearly described and appropriate: estimate of the central tendency of the outcome with a measure of precision is provided (e.g. mean/median (95% confidence interval/standard deviation/range)).<br>No = the statistical analysis is inappropriate, missing or inadequately described				
G) Missing data<br>Yes = extent of missing data reported and the methods for addressing missing data described<br>No = extent of missing data not reported and/or no description of how missing data were dealt with				
TOTAL (maximum 7)				

For peer review only

Supplementary Table S1A. Longitudinal studies

Reference	Location	Sampling frame / Health conditions	Sample size	Age (years±SD)	Female (%)	Presence of Plaque (n)	cIMT measurement (mm±SD)	MMSE (score±SD)	Major relevant finding
Auperin, et al. [1]	France	Community-based sample	Male: 521 Female: 753	65±3	60	Male: Yes (358) Female: Yes (217)	Male: 0.69±0.14 Female: 0.65±0.11	Male: 28.3±2 Female: 27.9±2.3	No evidence of an association between cIMT and MMSE in either sex. Association between presence of carotid plaques and impaired MMSE in men. No evidence of an association in women

For peer review only

Watanabe, et al. [2]	Japan	Patients with vascular dementia (VaD) and age-matched controls	Control: 63<br>VaD: 37<br>AD: 34	C: 72±11<br>VaD: 75±8<br>AD: 76±9	0	Control: Yes (15)<br>VaD: Yes (28)<br>AD: Yes (18)	Control : 0.68±0.11<br>VaD: 0.94±0.2<br>AD: 0.85±0.14	Control : 26±3<br>VaD: 13±6<br>AD: 10±5	Comparing VaD patients with controls. MMSE was lower, cIMT was greater and carotid plaques more frequent
Haley, et al. [3]	USA	Cardiology patients and volunteers	109	69.18±7.43	43	N/A	0.88±0.13	28.55±1.59	cIMT was not independently related to performance on measures of global cognitive functioning. Increased cIMT was significantly associated with poorer performan

For peer review only

										ce in the attention-executive-psychomotor domain independent of risk factors.
Muller, et al. [4]	Netherlands	Community-based sample, age-stratified selection and further stratified by cardiovascular disease status	No CVD: 217 Subclinical CVD: 125 Prevalent CVD: 54	No CVD: 54±10.3 Subclinical CVD: 66.8±8.1 Prevalent CVD: 67.7±8.8	0	N/A	No CVD: 0.77±0.01 Subclinical CVD: 0.89±0.01 Prevalent CVD: 0.89±0.02	No CVD: >28 (n=100, 63%) Subclinical CVD: >28 (n=49, 31%) Prevalent CVD: >28 (n=11, 7%)	No CVD: ≤28 (n=117, 50%) Subclinical CVD: ≤28 (n=73, 31%) Prevalent CVD: ≤28 (n=43, 18%)	Association of cIMT with MMSE not presented. Thicker cIMT was associated with lower scores on memory functioning.
Singh-Manoux, et al. [5]	UK	Occupational sample excluding individuals with stroke	High SES, Male: 1190 High SES, Female: 185 Intermediate SES, Male: 1477 Intermediate SES, Female:490	High SES, Male: 62.32±5.61 High SES, Female: 59.8±5.49 Intermediate SES, Male: 60±71 Intermediate SES, Female: 60±5.93	High SES: 17 Intermediate SES: 45 Low SES: 38	N/A	N/A	High SES, Male: 0.8±0.16 High SES, Female: 0.76±0.13 Intermediate SES, Male: 0.79±0.16 Intermediate SES, Female: 0.77±0.13	High SES, Male: 28.9±1.19 High SES, Female: 29.16±1.04 Intermediate SES, Male: 28.71±1.31 Intermediate SES,	An inverse association between cIMT and cognition was observed only in the low SES group. Evidence that SES

For peer review only

			Low SES, Male: 141 Low SES, F: 413	Low SES, Male: 60.84±6.51 Low SES, Female: 62±5.73				Low SES, Male: 0.82±0.18 Low SES, Female: 0.79±0.14	Female: 28.88±1.24 Low SES, Male: 27.86±1.7 Low SES, Female: 28.18±1.55	modifies the association between cIMT and cognition.
Carlsson, et al. [6]	USA	Population-based sample	No cognitive impairment: 1358 Cognitive impairment: 180	No cognitive impairment: 74.8±6.6 Cognitive impairment:80 ±7.1	No cognitive impairment: 62 Cognitive impairment: 54	N/A	N/A	No cognitive impairment: 0.95±0.23 Cognitive impairment: 1.00 ± 0.25	No cognitive impairment: 27.6 ± 1.7 Cognitive impairment: 21.1 ± 2.9	cIMT higher in people with cognitive impairment defined as MMSE <24 or proxy-reported dementia
El-Kattan, et al. [7]	Egypt	Vascular surgery patients and age- sex-matched healthy controls / PAD (66.6%)	Control: 10 PAD without CVD: 10 PAD with CVD: 10	Control: 53.9±9.55 PAD without CVD: 54.8±8.96 PAD with CVD: 56.1±9.62	Control: 20 PAD without CVD: 10 PAD with CVD: 20	N/A	Control: No (0) PAD without CVD: Yes (1) PAD with CVD: Yes (5)	Control: 0.7±0.1 PAD without CVD: 0.9±0.2 PAD with CVD: 1.07±0.13	Control: 30±0 PAD without CVD: 28±1.4 PAD with CVD: 26±1.5	Patients with peripheral arterial disease had higher cIMT and lower MMSE compared to healthy controls

For peer review only

Kearney-Schwartz, et al. [8]	France	Hypertensive patients with subjective memory complaints but excluding individuals with MMSW ≤24	198	69.3±6.2	53	N/A	Yes (50)	0.68±0.09	28.3±1.4	No evidence of an association between cIMT and impaired memory function implied, although data for MMSE or cognition not presented.
Zhong, et al. [9]	USA	Population-based sample	2794	49±9.8	54	N/A	Yes (2665)	0.65±0.15	28.7±1.3	Higher Carotid IMT and plaque were associated with lower MMSE score
Dias, et al. [10]	Brazil	HTN patients and non-HTN, non-cognitively impaired controls identified from	Controls: 48 HTN without cognitive impairment: 108 HTN with cognitive	Controls: 44.5±7.9 HTN without cognitive impairment: 55.75±8.6 HTN with cognitive	C 62.5 HTN without 53.7 HTN with 60.5	N/A		Controls: 0.69±1 HTN without cognitive impairment: 0.89±0.2 HTN with cognitive	Controls: 27.77±4.4 HTN without cognitive impairment: 28.44±1.3 HTN with cognitive	Higher cIMT was associated with higher odds ratio of MMSE score ≤24

For peer review only

		patients referred for investigation of high blood pressure. People with carotid plaques excluded.	impairment: 42	impairment: 63.3±9.3				impairment: 0.99±0.2	impairment: 22.02±2.4	
Stefanova, et al. [11]	Serbia	Patients with VCD or AD / VCD: Hyperlipidemia (36%) HTN (29%) DM (11%) AD: Hyperlipidemia (62%) HTN (88%) DM (28%)	VCD: 237 AD: 197	VCD: 67.4±8.18 AD: 68.5±9.36	VCD: 52.3 AD: 49	N/A N/A	VCD: Yes (120) AD: Yes (155)	VCD: 1.11±0.2 AD: 1.15±0.18	VCD: 23.81±2.33 AD: 16±5.9	Higher cIMT and plaque type was correlated with poorer MMSE in VCD. No evidence of a correlation between MMSE and cIMT or plaque in AD group.
**Rogne, et al.** [12]	Norway	Population-based sample / DM (1%)	1,577	Median (range): 57 (52-61)	48	N/A	N/A	Median (range): 0.78 (0.69–0.89)	N/A	No evidence of association between

		CHD (5.3%)								cIMT and MMSE.
**Xiang, et al.** [13]	China	Neurology patients / HTN (55%) DM (35%) CHD (10%)	No cognitive impairment : 1659 Cognitive impairment : 356	No cognitive impairment: 68.1±7.2 Cognitive impairment: 73.2±7.8	No cognitive impairment : 48 Cognitive impairment : 52	N/A N/A	Yes (1377) Yes (299)	No cognitive impairment: 0.76±0.14 Cognitive Impairment: 1.57±0.15	No cognitive impairment : ≥24 Cognitive impairment : <24	cIMT was associated with a higher odds ratio of cognitive impairmen t (MMSE <24)
**Nagai, et al.** [14]	Japan	Patients at high risk of cardiovascu lar disease / HTN (75.9%) DM (13.9%)	201	79.9±6.4	75	N/A	N/A	1.03±0.3	25.8±4.69	Compared with those with both low blood pressure variability and low IMT, patients with high blood pressure variability and high IMT had lower MMSE score or higher

For peer review only - http://bmjopen.bmj.com/site/about/guidelines.xhtml

										prevalence of cognitive impairment (based on a MMSE score ≤24)
Yano, et al. [15]	Japan	Hypertensive patients / HTN (100%)	587	73±8.1	59.0	N/A	N/A	NCD, median(range): 0.87(0.75-1.01) CD, median(range): 0.96 (0.82-1.06)	NCD, median(range): 28(27-29) CD, median(range): 24(22-24)	cIMT higher in patients with cognitive impairment (defined as MMSE score ≤ 24)
Liu, et al. [16]	China	Community-based sample / HTN (71%) DM (13%)	384	84.65±2.3	67	N/A	N/A	1.45 ± 0.30	Median (range): 24 (22-24)	cIMT inversely related to MMSE score.
Alhusaini, et al. [17]	Scotland	Population-based cohort HTN (47.3%) DM (10.1%)	518	72.7±0.73	46.6	N/A	Yes (77)	0.84±0.17	28.8±1.3	Data relating cIMT to MMSE not presented. Carotid stenosis was related to lower fluid

For peer review only

										intelligence.
Matsumoto, et al. [18]	Japan	Patients undergoing health screening	176	No cognitive impairment: 64.6 ±9.6 Cognitive impairment: 67.7±12.3	No cognitive impairment: 59.1 Cognitive impairment: 37	N/A	N/A	No cognitive impairment:1.7 ±0.7 Cognitive impairment: 2.0±1	N/A	No evidence of an association between cIMT and MMSE in a model adjusted for sex, age and years of education. cIMT higher in patients with cognitive impairment.
Muela, et al. [19]	Brazil	Patients. Individuals with cerebrovascular disease excluded / HTN (67%)	Normotensive: 69 HTN stage 1: 83 HTN stage 2: 59	Normotensive: 52.1±13.9 HTN stage 1: 52.1±13 HTN stage 2: 51.3±10.1	Normotensive: 55.1 HTN stage 1: 55.8 HTN stage 2: 53.6	N/A	N/A	Normotensive: 0.7±0.1 HTN stage 1: 0.8±0.1 HTN stage 2: 0.8±0.1	Normotensive: 28.03±1.92 HTN stage 1: 27.43±2.01	cIMT showed a weak negative correlation with MMSE consistent

For peer review only

									HTN stage 2: 26.66±2.07	with null. Negative correlations of cIMT with other cognitive measures were observed.
Mworozi, et al. [20]	Uganda	Community-based study HTN (25%) DM (5%)	210	69.9±7.76	71.4	N/A	Yes (45)	0.9±0.2	N/A	Presence of carotid artery plaque was associated with abnormal cognitive function defined as MMSE ≤24.

For peer review only

Supplementary Table S1B. Longitudinal studies

Reference	Location	Sampling frame / Health conditions	Sample size	Age, year (mean±SD)	Female, %	Mean follow-up (years)	Presence of Plaque (n)	cIMT measurement (mm±SD)	MMSE (score±SD)	Major relevant finding
Silvestrini, et al. [21]	Italy	Dementia clinic outpatients	66	72.7±6.1	55	1	Yes (52)	1.1±0.3	18.1±3	Higher baseline cIMT was associated with greater decline in MMSE scores
Viticchi, et al. [22]	Italy	Patients referred to Dementia clinic / Hyperlipidemia (55.6%) HTN (65%) DM (29.1%)	Cognitively stable: 96 Developed dementia: 21	Cognitively stable: 75±6 Developed dementia: 77.2±4.28	Cognitively stable:44.8 Developed dementia: 66.7	1	Cognitively stable: Yes (30) Developed dementia: Yes (9)	Cognitively stable : 0.92±0.15 Developed dementia: 1.04±0.16	Cognitively stable 27.14±1.76 Developed dementia: 27±1.56	Patients with abnormal cIMT (>1mm) had higher odds ratio of progressing from mild cognitive impairment to AD dementia
Zhong, et al. [23]	USA	Population-based sample	1311	66.8	59	5 and 10	Yes (682)	0.86±0.21	N/A	Higher cIMT was associated with higher risk of incident

										cognitive impairment (MMSE <24). Plaque was not associated with incident cognitive impairment or cognitive test performance.
Buratti, et al. [24]	Italy	Patients with high vascular risk	159 Normal cIMT: 68 Pathological cIMT: 91)	Normal cIMT:69.7±3.6 PcIMT:70.2±3.8	NcIMT:39.7 PcIMT:37.4	3	Normal cIMT:Yes (68) PcIMT:Yes (91)	Normal cIMT: < 1 PcIMT: ≥ 1	Normal cIMT baseline: 26.75±1.05 Normal cIMT follow-up:24.86±1.85 Pathological cIMT baseline: 27±1.25 Pathological cIMT follow-up: 25.27±1.86	No evidence of an association between cIMT and change in MMSE score

For peer review only

Buratti, et al. [25]	Italy	Patients referred to Dementia clinic	MCI: 300<br>AD: 106	MCI, meadian (range): 72 (67-76)<br>AD, meadian (range): 72 (68-76)	MCI: 37<br>AD: 43	1	MCI: Yes (114)<br>AD: Yes (53)	N/A	MCI: 27±1<br>AD: 27±1	cIMT >1mm and plaque associated with increased odds ratio of developme nt of Alzheimer disease over 12-month follow-up
Wendell, et al. [26]	USA	Community-based sample	1696	46.9±9.3	55	4	N/A	0.69±0.13	N/A	Weak negative association between cIMT and MMSE in fully adjusted models, consistent with null.

For peer review only

Falsetti, et al. [27]	Italy	Patients from neurological clinic with mild to moderate cognitive impairment	310	76.86±7.49	62.9	2	N/A	pNVAF(-) : 0.97±0.2 pNVAF(+) : 1.11±0.16	pNVAF(-) : 18.62±5.22 pNVAF(+): 15.8±5.17	cIMT weakly associated with progression to probably Alzheimer Disease but results compatible with null. Patients with non-valvular atrial fibrillation (pNVAF**(+)**) had lower MMSE and higher cIMT compared to pNVAF**(-)**.

For peer review only

Rouch, et al. [28]	N/A	Consecutive patients attending a memory clinic	363	75.2±7	65.6	4.5	No conversion to dementia: Yes (50) Conversion to dementia: Yes (40)	No conversion to dementia: 0.85±0.13 Conversion to dementia : 0.82±0.13	No conversion to dementia: 28.3±1.6 Conversion to dementia: 26.6±2.6	Higher cIMT and plaque associated with progression from MCI to dementia based on MMSE score.

For peer review only

Supplementary Table S1C. Cross-sectional plus longitudinal studies

Reference	Location	Sampling frame / Health conditions	Sample size	Age (years±SD)	Female (%)	Follow-up (years)	Presence of Plaque (n)	cIMT measurement (mm±SD)	MMSE (score±SD)	Major relevant finding
Komulainen, et al. [29]	Finland	Population-based sample	91 (CS) 47 (LT)	63.5±3.1 (CS) 75±3.2 (LT)	100	12	N/A	1.02±0.26 (Cross-sectional) 1.25±0.33 (LTl)	28.9±1.6 (Cross-sectional) 26.4±2.01 (LT)	There was no evidence of an association between IMT and MMSE score cross-sectionally or after 12-year follow-up, although confidence intervals of estimates were very wide. Associations between high cIMT and poor memory were seen both cross-sectionally and longitudinally.

For peer review only

Carcaillon, et al. [30]	France	Population-based sample	5798	73.4±4.8	60.5	7	Yes (3038)	0.71±0.12	N/A	Association between baseline carotid plaques and incident dementia. No association between cIMT and incident dementia
Kawasaki, et al. [31]	Japan	Population-based sample	494	Median (range): 87.2 (86.1–88.7)	55	3	Yes (328)	N/A	N/A	Presence of higher carotid artery plaque score was associated with lower MMSE scores. Weak evidence of an association between plaque score and increase rate of decline in MMSE compatible with null.

Abbreviations: AD: Alzheimer's disease, cIMT: carotid intima-media thickness, CD: cognitive dysfunction, CHD: coronary heart disease, CS: cross-sectional, CVD: cardiovascular disease, DM: Diabetes mellitus, HTN: Hypertensive, ICA: internal carotid artery, LT: longitudinal, MMSE: Mini Mental State Examination, MCI: mild cognitive impairment, N/A: not available, PAD: peripheral arterial disease, pNVAF: permanent non-valvular atrial fibrillation, SES; socioeconomic status, VaD: vascular dementia, VCD: vascular cognitive decline.

For peer review only

Supplementary Table S2. Exposure measures of the included papers.

Reference	IMT Measurement sites	Scanning techniques	Intra-observer reproducibility, blinded
Auperin, et al. [1]	Bilateral CCA, including the carotid bifurcation, and the first 2 cm of the ICA	Scanned longitudinally and transversely to assess the presence of plaques	Inter-reader agreement regarding the presence of plaques was excellent (κ coefficient=.90).
Watanabe, et al. [2]	Bilateral CCA including the carotid bifurcation and from 10 mm below the bifurcation,	Anterolateral and posterolateral angles of CCA with beam focused on the far wall	All measurements were determined by the same examiner, who was blinded to clinical history or risk factor profile.
Komulainen, et al. [29]	Bilateral far wall of CCA and bifurcation	N/A	sonographers were blinded to the randomization status of the study participants
Haley, et al. [3]	Left, far wall CCA 1 cm proximal to the carotid bulb	N/A	N/A
Muller, et al. [4]	Bilateral distal CCA	N/A	the intra-class correlation coefficient (ICC) for repeated IMT-measurement was 84%
Singh-Manoux, et al. [5]	Bilateral CCA	Longitudinal images triggered on the R-wave of the ECG	N/A
Silvestrini, et al. [21]	Bilateral CCA	of IMT taken as the thickest plaque-free region on the near and far walls in longitudinal images	N/A
Carlsson, et al. [6]	Bilateral, distal CCA, bifurcation, and the proximal portion of ICA	near and far walls scanned in each vessel segment (total of 12 sites)	N/A

For peer review only

El-Kattan, et al. [7]	No details provided	Arteries assessed using longitudinal views and checked for the state of arterial wall and the presence of thrombi	N/A
Kearney-Schwartz, et al. [8]	Bilateral CCA	Arteries assessed using longitudinal views	N/A
Zhong, et al. [9]	Bilateral, near and far walls of CCA, bifurcation and ICA	N/A	The reproducibility of IMT and plaque assessment was good. In a 10% sample (n=280) of participant scans that were re-graded the mean difference in IMT was 0.0019 mm and kappa statistics for plaque assessment ranged from 0.58 (ICA) to 0.71(bifurcation) with 97.3% agreement within ±1 for the number of sites with plaque.
Dias, et al. [10]	Bilateral CCA	The intima-media thickness was measured in near and far walls over a 1-cm segment of the artery located approximately 0.5 cm below the carotid-artery bulb and considered not to contain any plaque	The variability between IMT measurements less than 2%,; analysis performed by a physician blinded to the patient's clinical data
Zhong, et al. [23]	Bilateral CCA	Near and far walls	The reproducibility of IMT and plaque assessment was good. The mean inter-grader difference in IMT was 0.03 mm; and for plaque, the kappa coefficient was 0.76 and percent agreement was 90%

For peer review only

Viticchi, et al. [22]	Bilateral, distal segment of CCA	Measurement of near and far wall IMT on longitudinal image of 1.5cm segment of CCA that precedes the carotid bifurcation. Measurement made with an automated system at the thickest point where there were no plaques	N/A
Stefanova, et al. [11]	Far wall of CCA, ICA during diastole	during diastole measurements were done in a supine position with head elevated up to 45°, and tilted to the either side for 30°, depending on the side examined	The inter-rater correlation reliability assessed for 50 randomly selected patients from both groups was excellent (0.932), Physicians that performed ultrasound examinations were blinded to clinical data
Rogne, et al. [12]	Right, far and near wall CCA, bulb	measurement of IMT was performed in 10-mm segments of the far and near wall of the common carotid artery in the most proximal 10-mm segment of the bulb. The CCA, the bifurcation and the internal carotid artery were examined for plaque presence.	N/A
Xiang, et al. [13]	Bilateral, far wall CCA	Measurement of far wall cIMT in longitudinal B-mode images of the CCA, the carotid bifurcations, and the first 2 cm of the ICA at plaque-free sites	N/A
Nagai, et al. [14]	Bilateral, far wall CCA	CCA scanned bilaterally in longitudinal and transverse projections. The image was focused on the far wall of the artery	N/A
Yano, et al. [15]	Bilaterally at CCA, the bulb, and ICA	CCA, the bulb, and ICA measured from both transverse and longitudinal orientations, Region with the thickest IMT measured.	Coefficient of variation within 10%. Scan performed blind to patient's data
Buratti, et al. [24]	Bilateral CCA	N/A	inter-reader correlation coefficient of 0.88
Buratti, et al. [25]	Bilateral CCA	A longitudinal image of the distal segment of common carotid arteries was taken, and the measurement was obtained with an automatic system at the thickest point where there were no plaques on the proximal and distal wall	N/A

For peer review only

Carcaillon, et al. [30]	Bilateral CCA, bulb, and ICA	scanned longitudinally and transversally to detect plaques.	N/A
Kawasaki, et al. [31]	Bilateral CCA, ICA	N/A	All examinations were performed by a single physician blinded to the subject's clinical information
Liu, et al. [16]	Bilateral CCA	Three B-mode images were obtained using anterior, lateral, and medial angles. Maximum IMT in the right or left CCA used.	Scanning done by certified ultrasonographer who was unaware of the subjects' clinical details
Wendell, et al. [26]	Left CCA	Far wall IMT measured over a region 1.5 cm proximal to the carotid bifurcation	Intraobserver correlation between repeated carotid IMT measurements on 10 participants was 0.96 ($p < 0.001$).
Rouch, et al. [28]	Bilateral CCA	Near and far wall IMT measured in longitudinal images. Longitudinal and transverse images examined for plaques	N/A
Alhusaini, et al. [17]	Far walls of CCA, and bulb*	Far wall cIMT measured as the mean of 3 caliper measurements over a 1cm-long segment of the CCA and carotid bulb	N/A
Matsumoto, et al. [18]	Bilateral, far wall CCA, ICA	far wall maximum cIMT of the bilateral CCA,ICA measured at end-diastole in longitudinal images	Certified sonographers who were blinded with cognitive test results carried out the carotid ultrasonography
Muela, et al. [19]	Left CCA	Near and far wall cIMT was measured at the thickest point of the distal CCA, not including plaques using a computer program	an experienced observer who were blinded with Clinical condition carried out the carotid ultrasonography
Falsetti, et al. [27]	Bilateral CCA	cIMT measurements made on 1.5 cm segment of CCA artery preceding carotid bifurcation in a longitudinal image using a semiautomatic system	N/A
Mworozi, et al. [20]	CCA, ICA*	Participants scanned in both supine and semi recumbent positions, with the head slightly hyperextended and rotated 45 degrees away from the side being examined	N/A

For Peer Review Only

Abbreviations: (ICA) internal carotid artery, (ECA) the external carotid artery, (CCA) common carotid artery , (CIMT) carotid intima-media thickness , (N/A) not applicable. *not clear whether measured bilaterally.

For peer review only

Supplementary Table S3. The quality of included studies assessed using a modified seven-point criteria derived from Newcastle-Ottawa scale [32].

Reference	Quality score Newcastle-Ottawa scale (0-7)
Auperin, et al. [1]	3
Watanabe, et al. [2]	5
Komulainen, et al. [29]	4
Haley, et al. [3]	4
Muller, et al. [4]	4
Singh-Manoux, et al. [5]	4
Silvestrini, et al. [21]	4
Carlsson, et al. [6]	4
El-Kattan, et al. [7]	4
Kearney-Schwartz, et al. [8]	4
Zhong, et al. [9]	4
Dias, et al. [10]	4
Zhong, et al. [23]	4
Viticchi, et al. [22]	6
Stefanova, et al. [11]	4
Rogne, et al. [12]	4
Xiang, et al. [13]	4
Nagai, et al. [14]	4
Yano, et al. [15]	3

For peer review only

Buratti, et al. [24]	5
Buratti, et al. [25]	4
Carcaillon, et al. [30]	5
Kawasaki, et al. [31]	5
Liu, et al. [16]	4
Wendell, et al. [26]	4
Rouch, et al. [28]	5
Alhusaini, et al. [17]	4
Matsumoto, et al. [18]	2
Muela, et al. [19]	3
Falsetti, et al. [27]	4
Mworozi, et al. [20]	3

For peer review only

References

1. Auperin A, Berr C, Bonithon-Kopp C, et al. Ultrasonographic assessment of carotid wall characteristics and cognitive functions in a community sample of 59- to 71-year-olds. The EVA Study Group. *Stroke* 1996;27(8):1290-5. doi: 10.1161/01.str.27.8.1290 [published Online First: 1996/08/01]
2. Watanabe T, Koba S, Kawamura M, et al. Small dense low-density lipoprotein and carotid atherosclerosis in relation to vascular dementia. *Metabolism, clinical and experimental* 2004;53(4):476-82. doi: 10.1016/j.metabol.2003.11.020
3. Haley AP, Forman DE, Poppas A, et al. Carotid artery intima-media thickness and cognition in cardiovascular disease. *International Journal of Cardiology* 2007;121(2):148-54.
4. Muller M, Grobbee DE, Aleman A, et al. Cardiovascular disease and cognitive performance in middle-aged and elderly men. *Atherosclerosis* 2007;190(1):143-49. doi: 10.1016/j.atherosclerosis.2006.01.005
5. Singh-Manoux A, Britton A, Kivimaki M, et al. Socioeconomic status moderates the association between carotid intima-media thickness and cognition in midlife: Evidence from the Whitehall II study. *Atherosclerosis* 2008;197(2):541-48.
6. Carlsson CM, Nondahl DM, Klein BE, et al. Increased atherogenic lipoproteins are associated with cognitive impairment: effects of statins and subclinical atherosclerosis. *Alzheimer Dis Assoc Disord* 2009;23(1):11-7. doi: 10.1097/wad.0b013e3181850188 [published Online First: 2009/03/10]
7. El-Kattan MM, Zakaria YA, El-Fayomy N, et al. Peripheral arterial disease and cognition. *Egyptian Journal of Neurology, Psychiatry and Neurosurgery* 2009;46(2):311-22.
8. Kearney-Schwartz A, Rossignol P, Bracard S, et al. Vascular Structure and Function Is Correlated to Cognitive Performance and White Matter Hyperintensities in Older Hypertensive Patients With Subjective Memory Complaints. *Stroke* 2009;40(4):1229-36.
9. Zhong W, Cruickshanks KJ, Huang GH, et al. Carotid atherosclerosis and cognitive function in midlife: the Beaver Dam Offspring Study. *Atherosclerosis* 2011;219(1):330-3. doi: 10.1016/j.atherosclerosis.2011.07.013 [published Online First: 2011/08/13]
10. Dias EDM, Giollo LT, Martinelli DD, et al. Carotid intima-media thickness is associated with cognitive deficiency in hypertensive patients with elevated central systolic blood pressure. *Cardiovascular Ultrasound* 2012;10(1)
11. Stefanova E, Pavlovic A, Jovanovic Z, et al. Vascular risk factors in Alzheimer's disease - preliminary report. *J Neurol Sci* 2012;322(1-2):166-9. doi: 10.1016/j.jns.2012.07.065 [published Online First: 2012/09/04]
12. Rogne SO, Solbu MD, Arntzen KA, et al. Albuminuria and carotid atherosclerosis as predictors of cognitive function in a general population. *European Neurology* 2013;70(5-6):340-8.
13. Xiang J, Zhang T, Yang QW, et al. Carotid artery atherosclerosis is correlated with cognitive impairment in an elderly urban Chinese non-stroke population. *Journal of Clinical Neuroscience* 2013;20(11):1571-75.
14. Nagai M, Hoshide S, Nishikawa M, et al. Visit-to-visit blood pressure variability in the elderly: associations with cognitive impairment and carotid artery remodeling. *Atherosclerosis* 2014;233(1):19-26. doi: 10.1016/j.atherosclerosis.2013.11.071 [published Online First: 2014/02/18]

For peer review only

15. Yano Y, Bakris GL, Inokuchi T, et al. Association of cognitive dysfunction with cardiovascular disease events in elderly hypertensive patients. *J Hypertens* 2014;32(2):423-31. doi: 10.1097/HJH.0000000000000025 [published Online First: 2013/12/20]
16. Liu Z, Zhao Y, Wang X, et al. Low carotid artery wall shear stress is independently associated with brain white-matter hyperintensities and cognitive impairment in older patients. *Atherosclerosis* 2016;247:78-86. doi: 10.1016/j.atherosclerosis.2016.02.003 [published Online First: 2016/02/13]
17. Alhusaini S, Karama S, Nguyen TV, et al. Association between carotid atheroma and cerebral cortex structure at age 73 years. *Ann Neurol* 2018;84(4):576-87. doi: 10.1002/ana.25324 [published Online First: 2018/09/05]
18. Matsumoto L, Suzuki K, Mizuno Y, et al. Association of subclinical carotid atherosclerosis with immediate memory and other cognitive functions. *Geriatrics & gerontology international* 2018;18(1):65-71.
19. Muela HCS, Costa-Hong VA, Yassuda MS, et al. Higher arterial stiffness is associated with lower cognitive performance in patients with hypertension. *Journal of Clinical Hypertension* 2018;20(1):22-30.
20. Mworozi K, Ameda F, Byanyima RK, et al. Carotid artery plaque detected on ultrasound is associated with impaired cognitive state in the elderly: A population-based study in Wakiso district, Uganda. *Journal of Clinical Neuroscience* 2019;68:194-200.
21. Silvestrini M, Gobbi B, Pasqualetti P, et al. Carotid atherosclerosis and cognitive decline in patients with Alzheimer's disease. *Neurobiology of Aging* 2009;30(8):1177-83.
22. Viticchi G, Falsetti L, Vernieri F, et al. Vascular predictors of cognitive decline in patients with mild cognitive impairment. *Neurobiology of Aging* 2012;33(6):1127e1-27e9.
23. Zhong W, Cruickshanks KJ, Schubert CR, et al. Carotid atherosclerosis and 10-year changes in cognitive function. *Atherosclerosis* 2012;224(2):506-10. doi: 10.1016/j.atherosclerosis.2012.07.024 [published Online First: 2012/08/03]
24. Buratti L, Balucani C, Viticchi G, et al. Cognitive deterioration in bilateral asymptomatic severe carotid stenosis. *Stroke* 2014;45(7):2072-77.
25. Buratti L, Balestrini S, Altamura C, et al. Markers for the Risk of Progression from Mild Cognitive Impairment to Alzheimer's Disease. *Journal of Alzheimer's Disease* 2015;45(3):883-90.
26. Wendell CR, Waldstein SR, Evans MK, et al. Subclinical carotid atherosclerosis and neurocognitive function in an urban population. *Atherosclerosis* 2016;249:125-31. doi: 10.1016/j.atherosclerosis.2016.04.009 [published Online First: 2016/04/20]
27. Falsetti L, Viticchi G, Buratti L, et al. Interactions between Atrial Fibrillation, Cardiovascular Risk Factors, and ApoE Genotype in Promoting Cognitive Decline in Patients with Alzheimer's Disease: A Prospective Cohort Study. *Journal of Alzheimer's Disease* 2018;62(2):713-25.
28. Rouch L, Cestac P, Sallerin B, et al. Pulse wave velocity is associated with greater risk of dementia in mild cognitive impairment patients. *Journal of Hypertension* 2018;36 (Supplement 3):e340.
29. Komulainen P, Kivipelto M, Lakka TA, et al. Carotid intima-media thickness and cognitive function in elderly women: A population-based study. *Neuroepidemiology* 2007;28(4):207-13.
30. Carcaillon L, Plichart M, Zureik M, et al. Carotid plaque as a predictor of dementia in older adults: the Three-City Study. *Alzheimer's & Dementia* 2015;11(3):239-48.
31. Kawasaki M, Arai Y, Hirata T, et al. Carotid atherosclerosis, cytomegalovirus infection, and cognitive decline in the very old: a community-based prospective cohort study. *Age* 2016;38(2):1-13.

For peer review only

32. Wells G, Shea B, O'Connell D, et al. The Newcastle–Ottawa Scale (NOS) for Assessing the Quality of Non-Randomized Studies in Meta-Analysis. , 2000.

For peer review only

PRISMA 2020 Checklist

Section and Topic	Item #	Checklist item	Location where item is reported
**TITLE**			
Title	1	Identify the report as a systematic review.	Page1<br>line 1
**ABSTRACT**			
Abstract	2	See the PRISMA 2020 for Abstracts checklist.	P2+3
**INTRODUCTION**			
Rationale	3	Describe the rationale for the review in the context of existing knowledge.	P 4 line 2-19
Objectives	4	Provide an explicit statement of the objective(s) or question(s) the review addresses.	P 4 line 2-19
**METHODS**			
Eligibility criteria	5	Specify the inclusion and exclusion criteria for the review and how studies were grouped for the syntheses.	P5 +6+7
Information sources	6	Specify all databases, registers, websites, organisations, reference lists and other sources searched or consulted to identify studies. Specify the date when each source was last searched or consulted.	P5<br>24-28
Search strategy	7	Present the full search strategies for all databases, registers and websites, including any filters and limits used.	Supplementary file 1+2
Selection process	8	Specify the methods used to decide whether a study met the inclusion criteria of the review, including how many reviewers screened each record and each report retrieved, whether they worked independently, and if applicable, details of automation tools used in the process.	P6+7<br>Data extraction
Data collection process	9	Specify the methods used to collect data from reports, including how many reviewers collected data from each report, whether they worked independently, any processes for obtaining or confirming data from study investigators, and if applicable, details of automation tools used in the process.	P6 Line 26-34<br>P7 Line1-9<br>Data extraction
Data items	10a	List and define all outcomes for which data were sought. Specify whether all results that were compatible with each outcome domain in each study were sought (e.g. for all measures, time points, analyses), and if not, the methods used to decide which results to collect.	P6 Line 26-34<br>P7 Line1-9<br>Data extraction
	10b	List and define all other variables for which data were sought (e.g. participant and intervention characteristics, funding sources). Describe any assumptions made about any missing or unclear information.	P6 Line 26-34<br>P7 Line1-9<br>Data extraction
Study risk of bias assessment	11	Specify the methods used to assess risk of bias in the included studies, including details of the tool(s) used, how many reviewers assessed each study and whether they worked independently, and if applicable, details of automation tools used in the process.	P10<br>Egger`s test
Effect measures	12	Specify for each outcome the effect measure(s) (e.g. risk ratio, mean difference) used in the synthesis or presentation of results.	P8<br>Line 10-22
Synthesis methods	13a	Describe the processes used to decide which studies were eligible for each synthesis (e.g. tabulating the study intervention characteristics and comparing against the planned groups for each synthesis (item #5)).	Meta-analysis<br>P7<br>Line 13-22
	13b	Describe any methods required to prepare the data for presentation or synthesis, such as handling of missing summary statistics, or data conversions.	P7 line 7-9
	13c	Describe any methods used to tabulate or visually display results of individual studies and syntheses.	Meta-analysis<br>P7<br>Line 13-22

For peer review only - http://bmjopen.bmj.com/site/about/guidelines.xhtml

PRISMA 2020 Checklist

Section and Topic	Item #	Checklist item	Location where item is reported
	13d	Describe any methods used to synthesize results and provide a rationale for the choice(s). If meta-analysis was performed, describe the model(s), method(s) to identify the presence and extent of statistical heterogeneity, and software package(s) used.	Meta-analysis P7 Line 13-22
	13e	Describe any methods used to explore possible causes of heterogeneity among study results (e.g. subgroup analysis, meta-regression).	Quality Assessment P7 line 25-31
	13f	Describe any sensitivity analyses conducted to assess robustness of the synthesized results.	N/A
Reporting bias assessment	14	Describe any methods used to assess risk of bias due to missing results in a synthesis (arising from reporting biases).	Quality Assessment P7 line 25-31
Certainty assessment	15	Describe any methods used to assess certainty (or confidence) in the body of evidence for an outcome.	Quality Assessment P7 line 25-31
**RESULTS**			
Study selection	16a	Describe the results of the search and selection process, from the number of records identified in the search to the number of studies included in the review, ideally using a flow diagram.	P8+9 10 +fig 1 Tables 1A+b1+c1
	16b	Cite studies that might appear to meet the inclusion criteria, but which were excluded, and explain why they were excluded.	flowchart fig 1
Study characteristics	17	Cite each included study and present its characteristics.	Tables 1A+b1+c1
Risk of bias in studies	18	Present assessments of risk of bias for each included study.	(Figure 4 A&B).
Results of individual studies	19	For all outcomes, present, for each study: (a) summary statistics for each group (where appropriate) and (b) an effect estimate and its precision (e.g. confidence/credible interval), ideally using structured tables or plots.	Tables 1A+b1+c1
Results of syntheses	20a	For each synthesis, briefly summarise the characteristics and risk of bias among contributing studies.	(Figure 4 A&B). Tables 1A+b1+c1
	20b	Present results of all statistical syntheses conducted. If meta-analysis was done, present for each the summary estimate and its precision (e.g. confidence/credible interval) and measures of statistical heterogeneity. If comparing groups, describe the direction of the effect.	(Figure 2). (Figure 3).
	20c	Present results of all investigations of possible causes of heterogeneity among study results.	(Figure 4 A&B).
	20d	Present results of all sensitivity analyses conducted to assess the robustness of the synthesized results.	(Figure 2). (Figure 3).
Reporting biases	21	Present assessments of risk of bias due to missing results (arising from reporting biases) for each synthesis assessed.	(Figure 4 A&B). + supp 3
Certainty of evidence	22	Present assessments of certainty (or confidence) in the body of evidence for each outcome assessed.	P8 25-33 P9 1-24

For peer review only

PRISMA 2020 Checklist

Section and Topic	Item #	Checklist item	Location where item is reported
**DISCUSSION**			
Discussion	23a	Provide a general interpretation of the results in the context of other evidence.	P10 25-32<br>P11 1-22
	23b	Discuss any limitations of the evidence included in the review.	P10 25-32<br>P11 1-22
	23c	Discuss any limitations of the review processes used.	P12 line2-15
	23d	Discuss implications of the results for practice, policy, and future research.	P12 line2-15
**OTHER INFORMATION**			
Registration and protocol	24a	Provide registration information for the review, including register name and registration number, or state that the review was not registered.	supp
	24b	Indicate where the review protocol can be accessed, or state that a protocol was not prepared.	was not prepared.
	24c	Describe and explain any amendments to information provided at registration or in the protocol.	N/A
Support	25	Describe sources of financial or non-financial support for the review, and the role of the funders or sponsors in the review.	P 36
Competing interests	26	Declare any competing interests of review authors.	P36
Availability of data, code and other materials	27	Report which of the following are publicly available and where they can be found: template data collection forms; data extracted from included studies; data used for all analyses; analytic code; any other materials used in the review.	Supplementary files

From: Page MJ, McKenzie JE, Bossuyt PM, Boutron I, Hoffmann TC, Mulrow CD, et al. The PRISMA 2020 statement: an updated guideline for reporting systematic reviews. BMJ 2021;372:n71. doi: 10.1136/bmj.n71

For more information, visit: http://www.prisma-statement.org/

For peer review only
